# Cell fate simulation reveals cancer cell features in the tumor microenvironment

**DOI:** 10.1016/j.jbc.2024.107697

**Published:** 2024-08-20

**Authors:** Sachiko Sato, Ann Rancourt, Masahiko S. Satoh

**Affiliations:** 1 Glycobiology and Bioimaging Laboratory of Research Center for Infectious Diseases and Axe of Infectious and Immunological Diseases, Research Centre of CHU de Quebec, Faculty of Medicine, Laval University, Quebec, Canada; 2 Laboratory of DNA Damage Responses and Bioimaging, Research Centre of CHU de Quebec, Faculty of Medicine, Laval University, Quebec, Canada

**Keywords:** single-cell tracking, live-cell imaging, cell fate simulation, tumor microenvironment, 3-dimensional tumor microenvironment simulation, cancer cell heterogeneity, stemness, *Sambucus nigra* lectin, α2-6 sialic acid modification on glycans, cervical cancer, pancreatic cancer, cancer cell lines

## Abstract

To elucidate the dynamic evolution of cancer cell characteristics within the tumor microenvironment (TME), we developed an integrative approach combining single-cell tracking, cell fate simulation, and 3D TME modeling. We began our investigation by analyzing the spatiotemporal behavior of individual cancer cells in cultured pancreatic (MiaPaCa2) and cervical (HeLa) cancer cell lines, with a focus on the α2-6 sialic acid (α2-6Sia) modification on glycans, which is associated with cell stemness. Our findings revealed that MiaPaCa2 cells exhibited significantly higher levels of α2-6Sia modification, correlating with enhanced reproductive capabilities, whereas HeLa cells showed less prevalence of this modification. To accommodate the *in vivo* variability of α2-6Sia levels, we employed a cell fate simulation algorithm that digitally generates cell populations based on our observed data while varying the level of sialylation, thereby simulating cell growth patterns. Subsequently, we performed a 3D TME simulation with these deduced cell populations, considering the microenvironment that could impact cancer cell growth. Immune cell landscape information derived from 193 cervical and 172 pancreatic cancer cases was used to estimate the degree of the positive or negative impact. Our analysis suggests that the deduced cells generated based on the characteristics of MiaPaCa2 cells are less influenced by the immune cell landscape within the TME compared to those of HeLa cells, highlighting that the fate of cancer cells is shaped by both the surrounding immune landscape and the intrinsic characteristics of the cancer cells.

Cancer cell populations exhibit a range of phenotypical characteristics stemming from genetic sequence alterations and chromosomal variations (1, 2, 3, 4, 5, 6, 7, 8). Notably, missense mutations in *p53* gene are prevalent in over 50% of human cancer cases (9, 10, 11, 12, 13, 14, 15). Aneuploidy, characterized by an abnormal number of chromosomes, arises through mechanisms such as DNA damage, impaired mitotic phase checkpoints (16, 17), and multipolar cell division (MD), which is frequently observed in cancer tissues (18, 19, 20). Moreover, cancer cells often retain stemness-like properties to varying degrees within a cancer cell population (21, 22, 23, 24), contributing to the phenotypical diversity that accumulates during cancer development.

These heterogeneous cancer cell populations coexist within the tumor microenvironment (TME), comprising various cell types such as infiltrated immune cells, tumor-associated fibroblasts, endothelial cells, and the cancer cells themselves (25, 26, 27, 28). This ecosystem is characterized by conditions like hypoxia and abnormal levels of cytokines, growth factors, and metabolites (25, 26, 27, 28). Insights into the cellular composition of the TME in primary tumors have been provided by The Cancer Genome Atlas Program through genomic, epigenetic, and protein-level molecular profiling (29, 30, 31, 32, 33), depicting a snapshot of the TME's landscape at a specific stage in cancer development. However, both the landscape and the attributes of cancer cells within the TME are subject to evolution, influencing the fate of cancer.

To gain insight into the spatiotemporal dynamics of cancer cell evolution and its interplay with immune cells in the TME, we developed an approach with a 3D TME simulation. This simulation positions cancer and immune cells to investigate the fate of cancer cells within the TME, considering the spatiotemporal aspects of their interactions. Our approach began with acquiring spatiotemporal data on individual cell behavior through empirical single-cell tracking of established cultured cancer cell lines (20, 34). We developed a computerized single-cell tracking system to generate data suitable for bioinformatics analysis, involving long-term live-cell imaging, video generation, individual cell tracking, and cell lineage creation. Our focus was particularly on aspects related to stemness, notably the α2-6 sialic acid structure (α2-6Sia) (35, 36, 37, 38, 39, 40), and MD leading to altered chromosome numbers (18, 19, 20). Our findings revealed that MiaPaCa2 cells derived from pancreatic cancer exhibit higher α2-6Sia expression, correlating with enhanced reproductive capacity. In contrast, a smaller subset of cervical cancer–derived cells (HeLa cells) shows elevated α2-6Sia expression, highlighting distinct characteristics from MiaPaCa2 cells. Subsequently, using single-cell data from cancer cell lines and considering the *in vivo* variation of α2-6Sia expression levels (41, 42, 43, 44), we generated deduced cell populations digitally using a cell fate simulation algorithm (20). These populations, embodying the attributes of cancer cells in cell lines, reflect specific α2-6Sia expression levels observed *in vivo*. These populations were then integrated into the 3D TME simulation to simulate the fate of each cancer cell within the TME. We define the surrounding microenvironment of a cancer cell as suppressive, permissive, or lethal to cancer cells. Immune cells, cancer-associated fibroblasts, and endothelial cells are categorized based on their contribution to creating these microenvironments. The impact of these microenvironments on cancer cells was estimated using immune cell landscapes characteristic of 193 cervical and 172 pancreatic cancers.

We obtained results indicating that the fate of deduced cells generated based on MiaPaCa2 characteristics (such as a higher level of α2-6Sia, a stem glycosylation marker, and a high survival rate of cells after multipolar division) is less influenced by the immune cell landscape within the TME, compared to those of HeLa cells. These findings suggest that the fate of cancer cells within the TME is affected not only by the immune cell landscape but also by the intrinsic characteristics of the cancer cells themselves, underscoring the importance of understanding the spatiotemporal context of interactions between immune cells and cancer cells with diverse characteristics within the TME.

## Results

### Overview of single-cell tracking and 3D TME simulation

**Figure 1:**
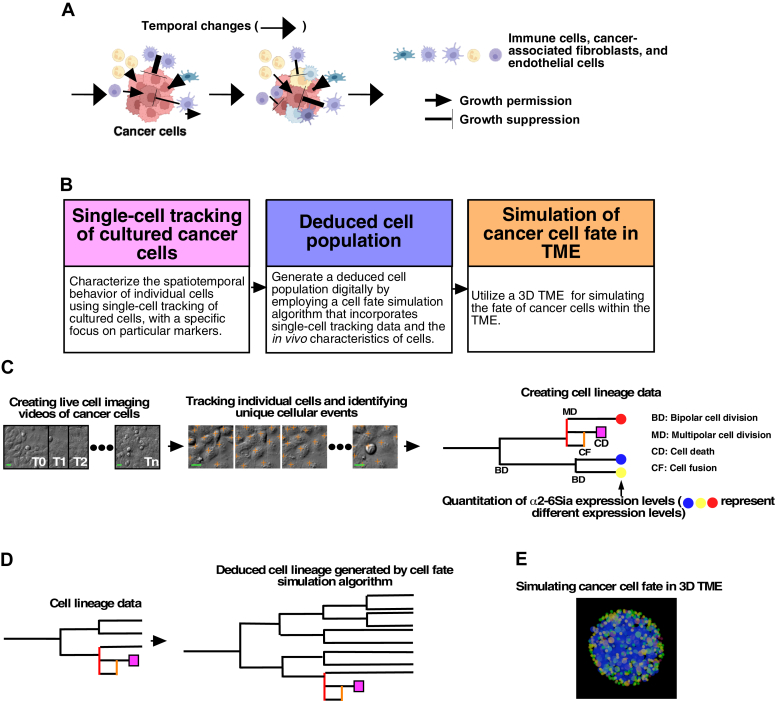
**Conceptual workflow for performing a 3D TME simulation.***A*, the cancer cell population within the TME exhibits inherent phenotypic diversity and dynamically evolves over time. *B*, to uncover the temporal dynamics of cancer cells within the TME, a conceptual workflow for performing a 3D TME simulation was developed. It starts with the acquisition of spatiotemporal information about individual cancer cells using a computerized single-cell tracking system. Subsequently, deduced cell populations that signify particular attributes of cells *in vivo* are generated using a cell fate simulation algorithm, followed by the execution of a 3D TME simulation. *C*, single-cell tracking is performed using established cancer cell lines. This involves creating live-cell imaging videos of cultured cancer cells and tracking individual cells. The resulting data resources include cell lineage information. Quantitative analysis of α2-6Sia expression levels is conducted at the end of imaging and integrated into the cell lineage data. The scale bar indicates 10 μm. *D*, utilizing the cell lineage data, deduced cell populations are created by focusing on specific characteristics of cancer cells using a cell fate simulation algorithm. *E*, the deduced cancer cell population, along with cells that create a suppressive, permissive, or lethal microenvironment for cancer cell growth, is introduced into a 3D TME generated by artificial intelligence for the simulation. In this simulation, deduced cancer cells (*depicted in blue*) coexist with cells involved in a suppressive, permissive, and lethal microenvironment (*depicted in yellow, green, and red,* respectively) within a *sphere*. α2-6Sia, α2-6 sialic acid; TME, tumor microenvironment.

The cancer cell population comprises cells with diverse phenotypic characteristics that evolve over time (Fig. 1*A*). The fate of these cells is influenced by cells residing in the TME, including immune cells. These immune cells can either promote or counteract the proliferation of cancer cells (25, 26, 27, 28). To simulate the fate of cancer cells within the TME, we required detailed information about the spatiotemporal behavior of individual cancer cells as well as the heterogeneity alteration.

To accomplish this, we conducted single-cell tracking analysis (20, 34) on established cancer cell lines (Fig. 1*B*). In order to generate data suitable for bioinformatic analysis, we devised a computerized single-cell tracking system (Fig. S1, see Text S1 for Legends). This procedure involved live-cell imaging using near-infrared (NIR)-illuminated differential interference contrast (DIC) microscopy (20, 34), which allowed us to create grayscale live-cell imaging videos without introducing any phototoxicity related to excitation light and fluorochromes. Then, we developed an algorithm to segment the grayscale DIC images with a wide range of cell densities, from sparse to densely populated populations (Fig. S2, *A* and *B*). This approach allowed us to track individual cell behaviors within these videos, enhancing our understanding of cellular dynamics. Subsequently, automated cell tracking was executed using an algorithm that analyzes the surrounding areas of the tracking-target region. It identifies the tracking-target area in the subsequent image frame by considering information from the surrounding areas (Fig. S3). Through this tracking process, various cellular events, including MD, can be detected. This results in the establishment of accurate cell lineage data, including the spatiotemporal behaviors of a cell that commence with the tracking process and encompass all of its progeny (Fig. 1*C*). After live-cell imaging, cells can be labeled with specific antibodies or lectins targeting biomolecules or posttranslational modifications, such as oligosaccharides bound to membrane proteins, for fluorescent imaging. The quantified fluorescent values associated with each cell were then integrated into the cell lineage data (Fig. 1*C*).

Because cancer cell lines are derived from a single cell within the original cancer cell population, they may only partially represent the characteristics of the original population. To address this, we employed a cell fate simulation algorithm (20) (Fig. 1, *B* and *D*). This algorithm calculates the probabilistic likelihood of a specific cellular event occurring subsequent to a preceding event, considering the duration between these events (20). For instance, it can assess the likelihood of MD occurring after bipolar cell division (BD), along with the associated duration. Consequently, the algorithm generates cell lineage data based on these probabilistic values. The growth dynamics of each cell population can be defined by combining these probabilistic values. Therefore, the cell fate simulation algorithm has the capability to simulate the growth of various cell populations when the requisite information to define these probabilistic values is available. For example, if we possess knowledge that the expression of α2-6Sia is notably high in highly reproductive cells, the algorithm can generate a deduced cell population in which reproductive cells exhibit a high level of α2-6Sia expression. Similarly, if we know the levels of variation of α2-6Sia expression *in vivo*, a digitally generated cell population (deduced cell population) that can reflect the attributes of growth patterns of cancer cells *in vivo* can be created. Subsequently, this deduced cell population was introduced into a 3D TME (Fig. 1, *B* and *E*). Cells that could impact the fate of the deduced cell population were categorized into three types: suppressive, permissive, and lethal. The simulation was performed by taking into account the characteristics of each cell, to determine how local suppressive, permissive, and lethal microenvironments affect the fate of individual deduced cells. This simulation allows us to investigate the fate of these cells within the TME.

### Distinct characteristics of cell lines derived from cervical (HeLa) and pancreatic (MiaPaCa2) cancer

**Figure 2:**
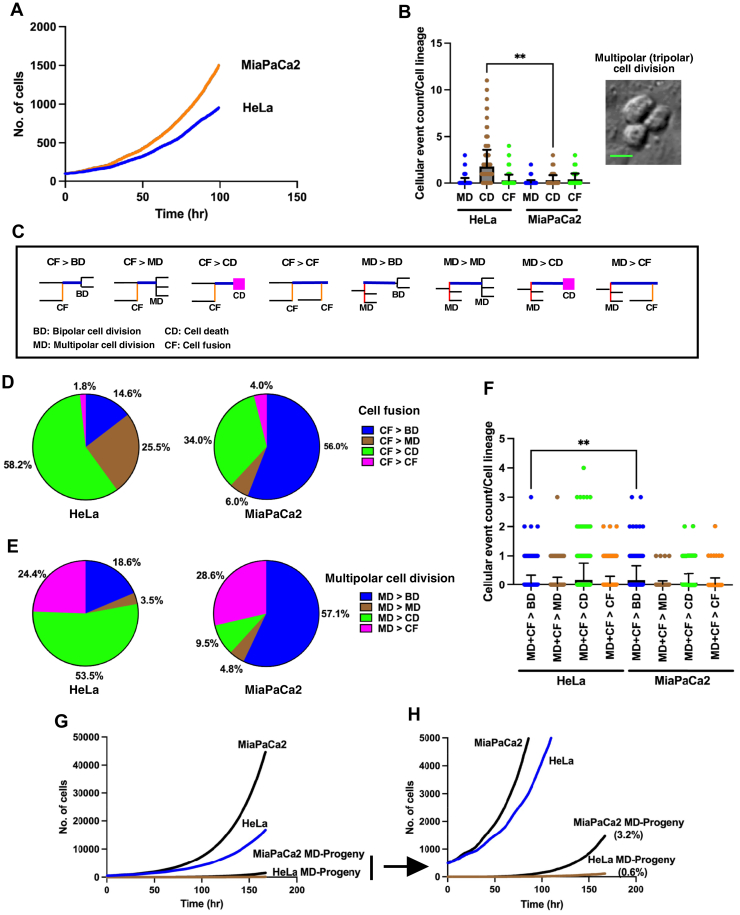
**Characteristics of cervical cancer cell line (HeLa) and pancreatic cancer cell line (MiaPaCa2).***A*, single-cell tracking was conducted with HeLa and MiaPaCa2 cells, plotting the number of cells at each time point to determine cell population expansion curves. The number of cells (referred to as progenitor cells) was adjusted to 100 cells at time point 1 based on the results using 420 and 230 progenitor cells for HeLa and MiaPaCa2, respectively. *B*, the counts of multipolar cell divisions (MDs), cell deaths (CDs), and cell fusions (CFs) occurring within cell lineages were recorded. Statistical analysis was performed using ordinary one-way ANOVA (Tukey’s), with ∗∗*p* < 0.01 indicating statistical significance. An example of a cell undergoing tripolar cell division is illustrated in a DIC image. The scale bar indicates 8 μm. Mean ± SD are shown. HeLa: n = 419, MiaPaca2: n = 230. *C*, the fate of cells resulting from cell fusion and the progeny produced by multipolar cell division were explored, with possible patterns illustrated (*e.g.*, “CF > BD” indicates bipolar cell division (BD) occurs following CF). *D*, the fate of cells resulting from cell fusion is detailed. *E*, the fate of progeny produced through multipolar cell division is presented. *F*, the total numbers of BDs, MDs, CDs, and CFs that occurred following CF or MD were determined, with statistical analysis conducted using ordinary one-way ANOVA (Tukey’s) and ∗∗*p* < 0.01 indicating statistical significance. Mean ± SD are shown. HeLa: n = 419, MiaPaca2: n = 230. Data is based on the MD + CF > BD, MD + CF > MD, MD + CF > CD, and MD + CF > CF counts of each cell lineage. *G*, cell fate simulation, based on single-cell tracking data of HeLa and MiaPaCa2 cells, determined the number of reproductive progeny derived from cells produced through multipolar cell division. Simulations were started with 500 progenitor cells. *H*, a magnified view of the reproductive progeny results is shown, with “MD-progeny” referring to reproductive progeny produced through multipolar cell division. The percentage in parentheses indicates the proportion of reproductive progeny within the total HeLa or MiaPaCa2 cell population. *A*, *B*, *D*, *E*, and *F*, cell lineage databases for HeLa and MiaPaCa2 are used for these analyses. DIC, differential interference contrast.

We conducted single-cell tracking using HeLa and MiaPaCa2 cell lines. The cell population size at each time point was determined through single-cell tracking data (Fig. 2*A*). Additionally, Data S1, Data S2, Data S3 (video), and Data S4 (video) provide cell lineage maps and videos illustrating the tracking processes. Our results reveal an increase in the population size of HeLa and MiaPaCa2 cells, with average cell doubling times of 26.6 and 22.7 h, respectively.

Next, we assessed the occurrence of MDs, cell death (CD), and cell fusion (CF) in HeLa and MiaPaCa2 cells (Fig. 2*B*). Notably, MiaPaCa2 cells exhibited a lower frequency of MDs, CD, and CF than HeLa cells. Subsequently, we delved into the cellular events following MDs and CF. In Figure 2*C*, we have listed the patterns we analyzed. For instance, “CF > BD” illustrates that BD occurs following CF. In the case of HeLa cells, over 50% of the progeny arising from MD and cells resulting from CF ultimately underwent CD (Fig. 2, *D* and *E*, HeLa). In contrast, among MiaPaCa2 cells generated through MD or CF, more than 50% retained the capability to undergo BD (Fig. 2, *D* and *E*, MiaPaCa2). This difference between HeLa and MiaPaCa2 cells suggests that while MiaPaCa2 cells undergo MD and CF less frequently, the progeny resulting from these events have a higher probability of survival than HeLa cells (Fig. 2*F*, MD + CF > BD, HeLa *versus* MiaPaCa2). Consequently, MiaPaCa2 cells have a greater chance of accumulating cells with altered chromosome numbers due to MD and CF, potentially making up 3.2% of the total cell population of MiaPaCa2 cells after 170 h of growth (Fig. 2, *G* and *H*).

### Expression of α2-6Sia in HeLa and MiaPaCa2 cell lines

**Figure 3:**
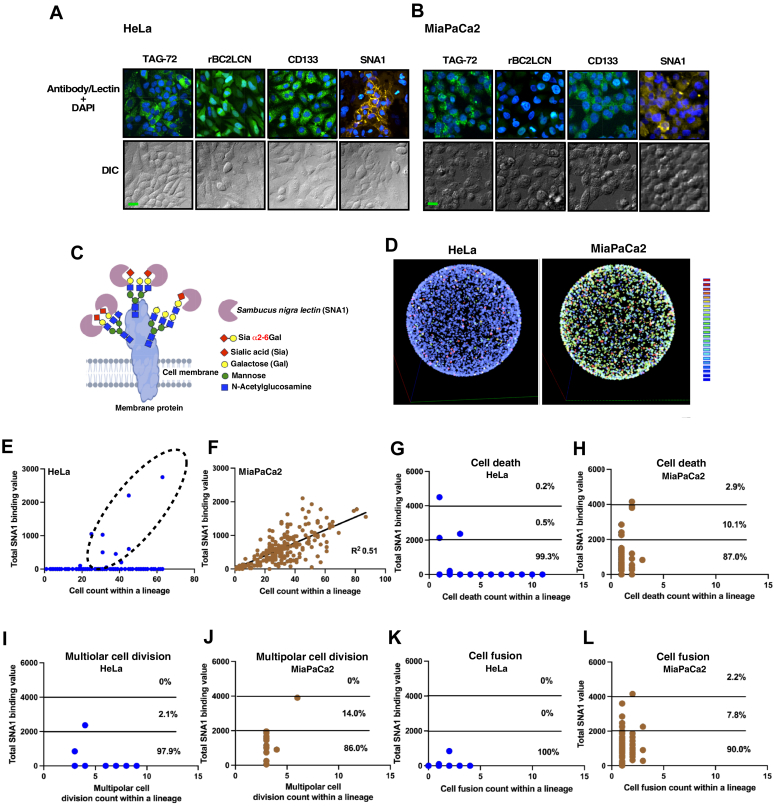
**Analysis of α2-6Sia expression using SNA1.** Fluorescence-tagged rBC2LCN, SNA1, anti-CD133, and anti-TAG-72 staining was performed on HeLa (*A*) and MiaPaCa2 (*B*) cells, followed by Alexa 488–labeled secondary antibody visualization and fluorescence imaging. Nuclei were counterstained with 4′,6-diamidino-2-phenylindole (*blue*), and DIC images were included. *A* and *B*, scale bars indicate 20 μm. *C*, illustration of membrane proteins with sialic acid α2-6 galactose (α2-6Sia) termini oligosaccharides, recognized by *Sambucus nigra* lectin (SNA1), which can bind to α2-6Sia with higher affinity than other oligosaccharide structures. *D*, visualization of α2-6Sia expression in individual cells arranged on a *spherical surface*, color-coded by a heat map scale. *E* and *F*, cell counts within HeLa (*E*) and MiaPaCa2 (*F*) cell lineages, respectively, with total SNA1 binding values highlighted by a *dotted circle* for lineages with higher values. Statistical analysis used simple linear regression (R^2^ = 0.51). *G*-*H*, cell death counts within HeLa (*G*) and MiaPaCa2 (*H*) lineages plotted against total SNA1 binding, categorized into groups by SNA1 values (<2000, 2000–3999, >4000) with percentages. *I*-*J*, multipolar cell division counts within HeLa (I) and MiaPaCa2 (*J*) lineages against SNA1 binding, categorized similarly with percentages. *K*-*L*, cell fusion counts within HeLa (*K*) and MiaPaCa2 (*L*) lineages against SNA1 binding are also categorized with percentages shown. *E*-*L*, HeLa: n = 420, MiaPaca2: n = 230. Cell lineage databases for HeLa and MiaPaCa2 are used for these analyses. DIC, differential interference contrast.

HeLa and MiaPaCa2 cell lines were subjected to immunostaining for the cancer marker TAG-72 (45, 46, 47), the stem cell marker CD133 (48, 49, 50), and the stem cell glycan marker fucose α1-2galactose α1-3 structure (40), recognized by the rBC2LCN bacterial lectin (Fig. 3, *A* and *B*, respectively). The majority of HeLa cells exhibited broad expression of these markers, with most MiaPaCa2 cells also displaying these markers, except for the rBC2LCN lectin staining.

The oligosaccharide structure attached to membrane proteins and terminated by α2-6Sia structure, recognized by *Sambucus nigra* lectin (Fig. 3*C*; SNA1), is suggested to be associated with the maintenance of stemness features and malignancy (35, 36, 37, 38, 39, 40, 43). Interestingly, only a subset of HeLa cells expressed α2-6Sia (Fig. 3*A*, SNA1 staining, and Data S1; the right side of the lineage maps), indicating that α2-6Sia–related stemness differs from other markers, such as CD133. Conversely, the majority of MiaPaCa2 cells displayed positive α2-6Sia expression (Fig. 3*B*, SNA1 staining, and Data S2; the right side of the lineage maps). We employed single-cell tracking data linked to the SNA1 binding levels of individual cells to create a visual heat map representation of these levels. In this visualization (Fig. 3*D*), individual cells were positioned on the surface of a sphere, illustrating that MiaPac2 cells exhibit elevated levels of α2-6Sia–related stemness compared to HeLa cells.

In Figure 3*E* (HeLa) and F (MiaPaCa2), we plotted the total number of cells in a cell lineage on the horizontal axis, where a higher value represents a cell's ability to produce a larger number of progeny (indicative of reproductive ability), and the total SNA1 binding levels of progeny within the cell lineage on the vertical axis. Consequently, if a cell lineage comprises a large number of cells, all expressing high levels of α2-6Sia, it will be positioned toward the upper-right quadrant of the graph. Notably, only a subset of the HeLa cell population expressed α2-6Sia, and these cells exhibited a higher reproductive ability, while α2-6Sia-negative cells showed varied reproductive ability (Fig. 3*E*). In contrast, the majority of MiaPaCa2 cells expressed α2-6Sia, and the levels of expression displayed a linear relationship with their reproductive ability (Fig. 3*F*). We also conducted similar analyses by plotting the CD count (Fig. 3*G* for HeLa and Fig. 3*H* for MiaPaCa2), MD count (Fig. 3*I* for HeLa and Fig. 3*J* for MiaPaCa2), and CF count (Fig. 3*K* for HeLa and Fig. 3*L* for MiaPaCa2). In all instances, it was observed that CD, MD, and CF predominantly occurred in cells exhibiting lower α2-6Sia expression levels (below 2000 of total SNA1 binding). These findings not only underscore the connection between α2-6Sia expression and the maintenance of cellular reproductive ability but also highlight its role in reducing the likelihood of CD, MD, and CF. Taken together, these results suggest that only a subset of HeLa cells expressing α2-6Sia retain their stemness, contributing to a higher reproductive ability to maintain the cell population (Fig. 3*E*), consistent with our previous observation (34). The presence of α2-6Sia–negative cells increases the likelihood of CD, MD, and CF (Fig. 3, *G*, *I* and *K*). In contrast, the majority of MiaPaCa2 cells maintain α2-6Sia–related stemness (Fig. 3*F*), resulting in fewer occurrences of CD, MD, and CF (Fig. 3, *H*, *J*, and *L*). This implies that α2-6Sia–related stemness plays a role in preserving the integrity of the cell population.

### Generation of deduced cell populations and designing the 3D TME simulation

**Figure 4:**
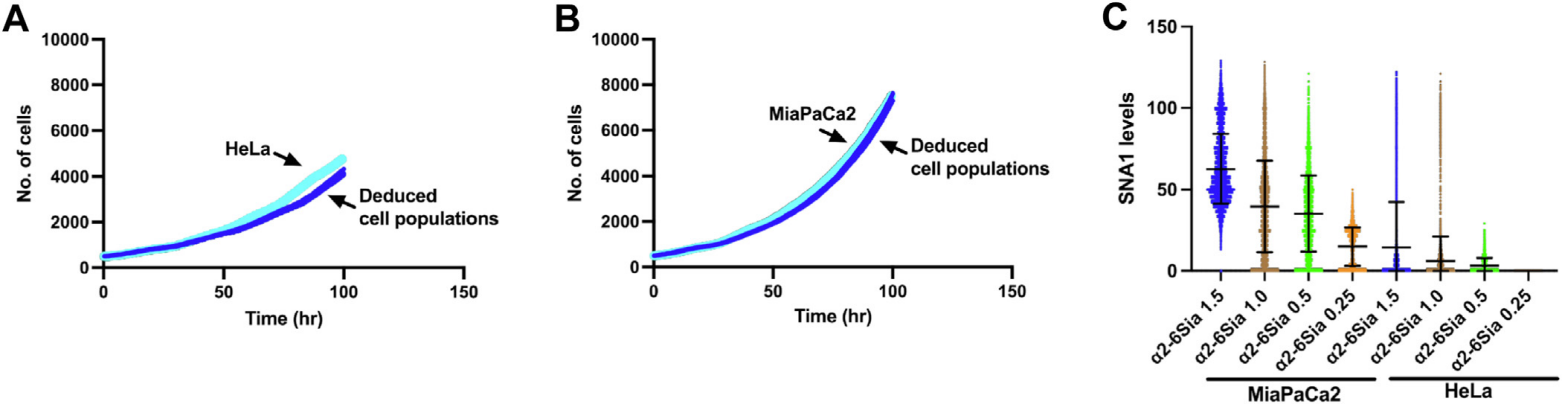
**Generation of deduced cell populations.***A*, HeLa (empirical data); *light blue line* and HeLa α2-6Sia; *blue lines* and (*B*) MiaPaCa2 (empirical data); *light blue line* and MiaPaCa2 α2-6Sia; *blue lines*. Deduced cell populations (500 cell lineages) were generated using a cell fate simulation algorithm by varying α2-6Sia expression levels. The cell population expansion curves for these cells are shown. *C*, the expression levels of α2-6Sia in the deduced cell population are presented. As the cell fate simulation algorithm incorporated the recorded α2-6Sia expression patterns from cell lineage data, the deduced cell population's α2-6Sia expression levels reflect those of HeLa and MiaPaCa2 cells. These cell populations were designated as HeLa α2-6Sia 1.5, 1.0, 0.5, and 0.25, and MiaPaCa2 α2-6Sia 1.5, 1.0, 0.5, and 0.25. Mean ± SD are shown. The number of data points is 15,053, 15,342, 14,686, 14,718, 8725, 8973, 8932, and 9393, respectively. α2-6Sia, α2-6 sialic acid.

Our findings thus far indicate distinct patterns of α2-6Sia expression in HeLa and MiaPaCa2 cells, which could be related to the reproductive capability of these cells and their role in maintaining the integrity of the cell population. Specifically, our results suggest that α2-6Sia expression reduces the occurrence of CF and MD, events that can lead to the production of cells with altered chromosome numbers. In cancer tissues, the expression levels of α2-6Sia among cancer cells vary (41, 42, 43, 44), implying that each cancer cell within a tissue may possess a unique reproductive ability and a chance to produce cells with altered chromosome numbers. This variation falls within the range of 10 to 200% of the average expression levels (41, 42, 43, 44). To explore the effects of varying levels of α2-6Sia expression on cancer cell fate, we created deduced cell populations with 1.5, 1.0, 0.5, and 0.25-fold α2-6Sia expression relative to the levels found in HeLa and MiaPaCa2 cells, using the cell fate simulation algorithm (20) with some modifications. An overview of the deduced cell population generation is provided in Figure S4 and Experimental procedures. The created cell populations are designated as follows: HeLa α2-6Sia 1.5, 1.0, 0.5, and 0.25, and MiaPaCa2 α2-6Sia 1.5, 1.0, 0.5, and 0.25. In essence, the cell fate simulation algorithm constructs a digitally generated cell population (deduced cell population), where events that occur within each cell composing the population and its progeny—such as cell division, CD, and CF—are determined. This allows for the generation of cell lineage maps that visually represent the fate and genealogical relationships of each cell within the population. Cell lineage maps of HeLa α2-6Sia 1.5 and MiaPaCa2 α2-6Sia 1.5 are provided in Data S5 and Data S6, respectively. Figure 4*A* (HeLa) and Figure 4*B* (MiaPaCa2) depict the cell population expansion curves, which include empirical tracking results (light blue line) and deduced cell populations (blue lines). The deduced cell populations generated using single-cell tracking data of HeLa cells show a ∼5% slower rate of population expansion than actual HeLa cells, as the cell fate simulation algorithm reflects the averaged proliferation pattern of the given cell population. In Figure 4*C*, the levels of α2-6Sia in the deduced cell populations are depicted. We used the deduced cell populations for 3D TME simulation, carried out by positioning these cells and immune cells within a 3D space (sphere) (see details in Experimental procedures).

### Categorization of TME-resident cells for 3D TME simulation

**Figure 5:**
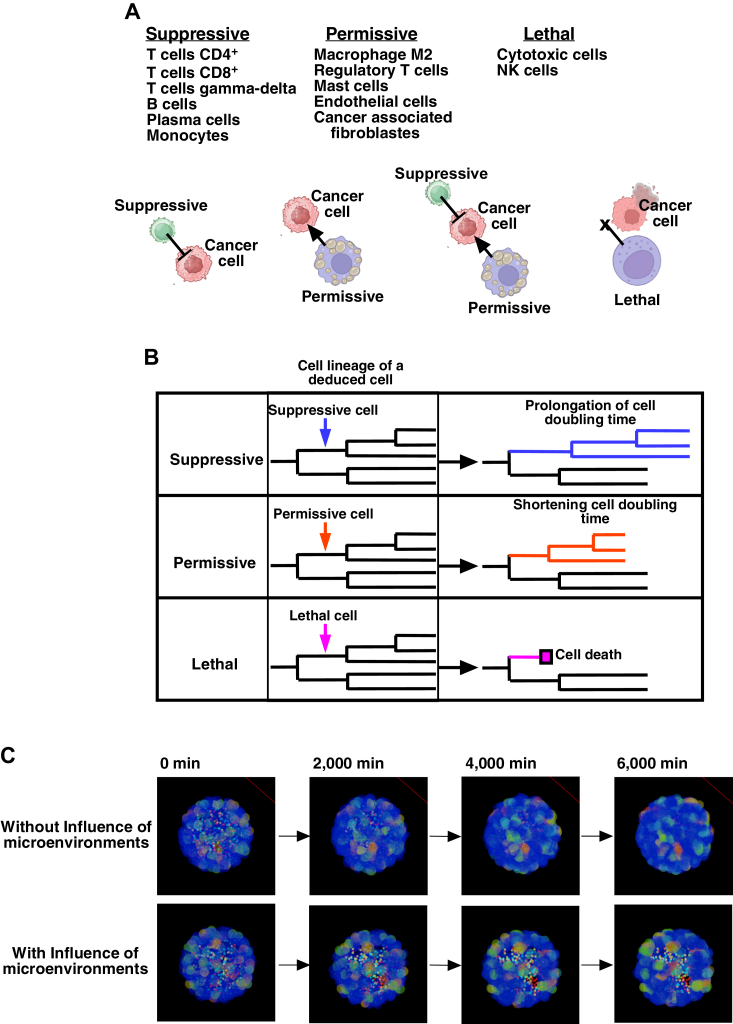
**Categorization of immune cells.***A*, immune cells within the TME (the ConsensusTME dataset in the tumor immune micro-environment cell composition database) were classified into suppressive, permissive, and lethal categories. *B*, in the 3D TME simulation, each deduced cell undergoes bipolar cell division, multipolar cell division, cell fusion, and cell death according to the cell lineage data generated by the cell fate simulation algorithm. When a cancer cell is located within a defined spatial distance of a suppressive microenvironment, it experiences an extended doubling time. The doubling time of its progeny is also extended. When a cancer cell is within a specified spatial distance of a permissive microenvironment, it experiences a shortened doubling time. The doubling time of its progeny is also shortened. When both suppressive and permissive microenvironments are within a specified distance, their impact on cancer cells depends on their respective distances from the cancer cell. When a cancer cell is within a specified spatial distance of a lethal microenvironment, cell death is marked to the cell, and its progeny are removed from the cell lineage database. *C*, an example of a 3D TME simulation is shown. The suppressive, permissive, and lethal microenvironments are represented by *red, light blue,* and *yellow spheres*, respectively (denoted as *smaller spheres*), and cancer cells are shown by *larger spheres*. The expression levels of α2-6Sia were visualized using a heat map scale, ranging from *blue* to *red*. Without the influence of microenvironments, cancer cells expand within the space. With the influence of microenvironments, particularly lethal environments, fewer cancer cells remained after 6000 min of simulation. TME, tumor microenvironment; α2-6Sia, α2-6 sialic acid.

Within the TME, cancer cells can be influenced by immune cells, cancer-associated fibroblasts, and endothelial cells through mechanisms such as supplying nutrients, releasing growth factors, and modulating immune cell functions by releasing cytokines (25, 26, 27, 28). Each type of cell contributes to generating a microenvironment around a cancer cell that can either favor or suppress its growth. One approach to developing the 3D TME simulation is to define the contribution of each type of cell, although quantitative information to define the degree of contribution of each cell to create a microenvironment is not yet clear. Thus, rather than focusing on each type of cell, we consider three types of microenvironments that could impact cancer cell proliferation: suppressive, permissive, and lethal environments (Fig. 5*A*), and calculate the total percentage of cells that belong to each category to perform a 3D TME simulation. We categorize cells using the ConsensusTME dataset in the tumor immune micro-environment cell composition database (TIMEDB) (51). Suppressive includes T cell CD4^+^, T cell CD8^+^, T cell gamma-delta, B cells, plasma cells, and monocyte. Although some T cell CD8^+^ could have a lethal effect on cancer cells, there are various subsets (52), so we categorize them as suppressive. Permissive includes macrophage M2, regulatory T cells, mast cells, endothelial cells, and cancer-associated fibroblasts. Lethal includes cytotoxic cells and natural killer cells. Some cell types, such as M1 macrophages and neutrophils, can exhibit dual roles, either suppressing or promoting cancer cell growth depending on the context (25, 26, 27, 28). Due to this complexity, we chose not to include these cells in the 3D TME simulation. This approach, focusing on three categories of microenvironments, contributed to streamlining the development of the algorithm, and we tested whether unique cancer characteristics can be highlighted under the simulation conditions.

To facilitate our analysis, we collectively refer to cells that create these microenvironments as suppressive, permissive, and lethal cells. Thus, in a 3D space, if a cancer cell is located near a suppressive cell, it implies that the cancer cell is close to the suppressive microenvironment. Within the actual 3D TME simulation, suppressive, permissive, and lethal cells were placed alongside deduced cancer cells that were generated digitally. These cancer cells undergo cell division, CD, or CF following cell lineage data generated by the cell fate simulation algorithm (Fig. 5*B*, and Data S5 and Data S6). In scenarios where immune cells do not influence cancer cells, the latter proliferate in 3D space according to their cell lineage map data (Fig. 5*B*). The effects of encountering suppressive, permissive, or lethal cells are recorded by modifying the data. When a deduced cancer cell encounters a suppressive cell, the doubling times for the cell and its progenies are lengthened, slowing their proliferation. Conversely, when a deduced cell encounters a permissive cell, the doubling times of the deduced cells and their progenies are reduced, enhancing proliferation rates, although the shortest cell doubling time is limited to the minimum doubling time observed in the cell population.

Interactions with a lethal cell result in the removal of the deduced cell and its progenies from the simulation (see details in Experimental procedures).

To perform the 3D TME simulation, several parameters are considered, including [1] the resistance levels of deduced α2-6Siaexpressing cells against suppressive and lethal cells, [2] cell ratios: establishing the relative numbers of suppressive, permissive, and lethal cells in relation to deduced cancer cells, and [3] impact strength: the strength of the influence exerted by suppressive, permissive, and lethal cells on deduced cancer cells. Details for setting these parameters, along with control data, are provided in Text S2 (Pseudocode), and Fig. S5. Graphical representations of the 3D TME, depicting views at 0, 2000, 4000, and 6000 min, are shown in Figure 5*C*. The simulation was conducted with HeLa α2-6Sia 1.5 cells under Control conditions or the influence of lethal cells.

### Simulations with pancreatic and cervical cancer TME

**Figure 6:**
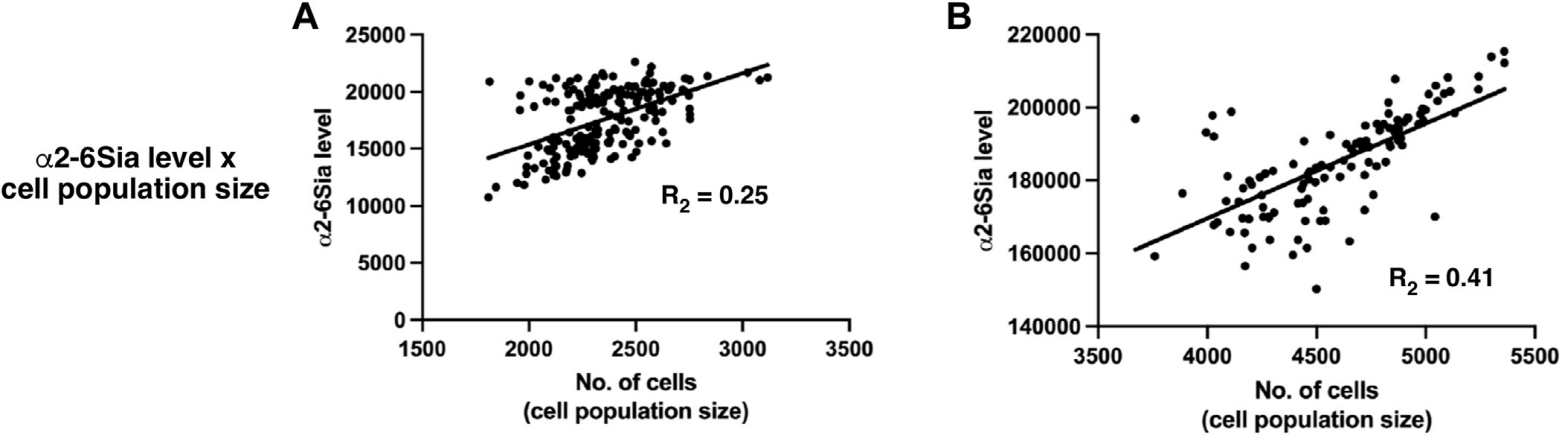
**Relationships of simulation results with α2-6Sia expression.** Simulation results are presented for HeLa α2-6Sia 1.0 (*A*) and MiaPaCa2 α2-6Sia 1.0 (*B*). The simulated cell population size is plotted against α2-6Sia expression levels. The resulting R^2^ values were shown. Each *dot* signifies the outcome of simulations using the TME cell landscape data of each cancer case. The number of data points for HeLa α2-6Sia 1.0 and MiaPaCa2 α2-6Sia 1.0 are 193 and 172, respectively. α2-6Sia, α2-6 sialic acid; TME, tumor microenvironment.

**Figure 7:**
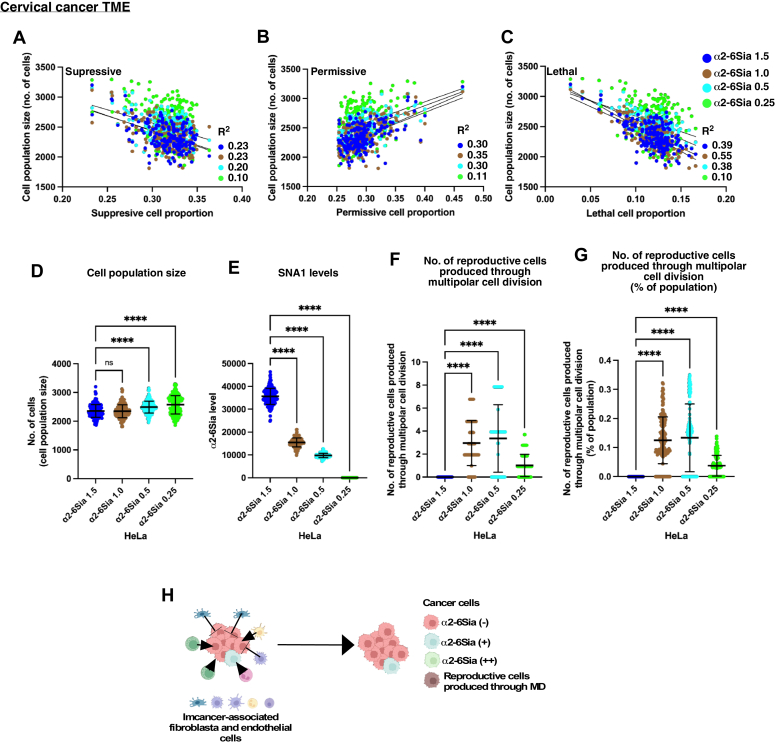
**Characteristics of HeLa α2-6Sia cells within the TME.** Four different populations of HeLa α2-6Sia cells: α2-6Sia 1.5, α2-6Sia 1.0, α2-6Sia 0.5, and α2-6Sia 0.25, were employed in 3D TME simulations using TME cell landscape data from 193 cervical cancer cases. *A*–*C*, the simulated population size of HeLa α2-6Sia cell populations is plotted against the initial proportion of suppressive cells (*A*), permissive cells (*B*), and lethal cells (*C*) within TME. Variation of each data relative to the linear regression line was calculated. The resulting R^2^ values were shown. *D*-*G*, cell population sizes (*D*), α2-6Sia expression levels (*E*), and the number of reproductive cells produced through multipolar cell division (*F*) of HeLa α2-6Sia (1.5, 1.0, 0.5, and 0.25) were analyzed. The percentage of the number of reproductive cells produced through multipolar cell division relative to the total cell population is also shown *G*, statistical analysis was conducted using ordinary one-way ANOVA (Tukey’s), and significance is denoted as ∗∗∗∗*p* < 0.0001, while "ns" indicates no significance. Mean ± SD are shown. The results of the analysis are shown between HeLa α2-6Sia 1.5 and the other populations. *D*-*G*, the number of data points is 193 for each of HeLa α2-6Sia 1.5, 1.0, 0.5, and 0.25. *H*, the summarized characteristics of HeLa α2-6Sia cells within the TME context are presented. Each *dot* signifies the outcome of simulations using the TME cell landscape data of each cancer case. α2-6Sia, α2-6 sialic acid; MD, multipolar cell division; TME, tumor microenvironment.

**Figure 8:**
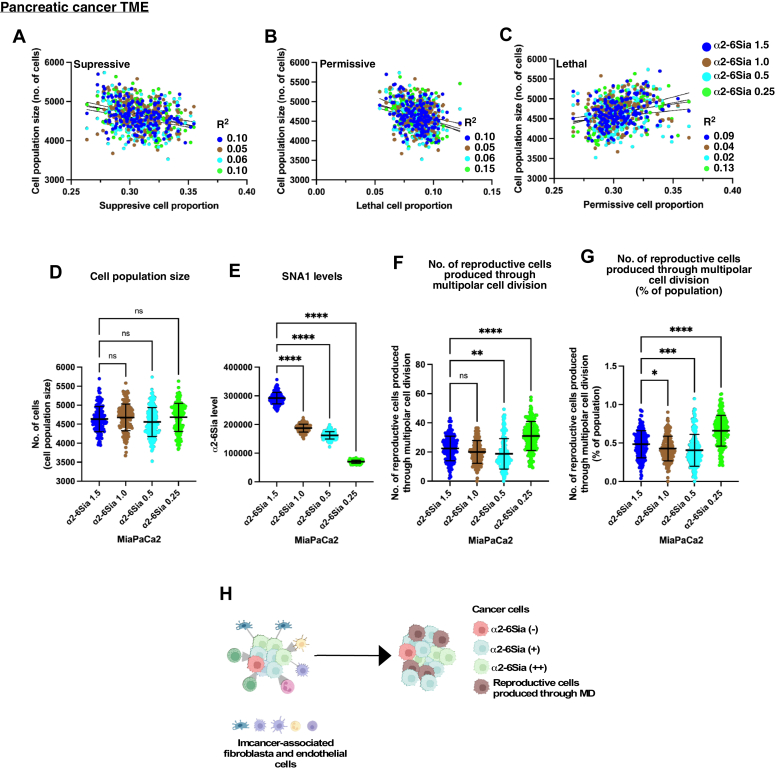
**Characteristics of MiaPaCa2 α2-6Sia cells within the TME.** Four different populations of MiaPaCa2 α2-6Sia cells: α2-6Sia 1.5, α2-6Sia 1.0, α2-6Sia 0.5, and α2-6Sia 0.25, were employed in 3D TME simulations using TME cell landscape data from 172 pancreatic cancer cases. *A*–*C*, the simulated population size of MiaPaCa2 α2-6Sia cell populations is plotted against the initial proportion of suppressive cells (*A*), permissive cells (*B*), and lethal cells (*C*). Variation of each data relative to the linear regression line was calculated. The resulting R^2^ values were shown. *D*–*G*, cell population sizes (*D*), α2-6Sia expression levels (*E*), and the number of reproductive cells produced through multipolar cell division (*F*) of MiaPaCa2 α2-6Sia (1.5, 1.0, 0.5, and 0.25) were analyzed. The percentage of the number of reproductive cells produced through multipolar cell division relative to the total cell population is also shown (*G*). Statistical analysis was conducted using ordinary one-way ANOVA (Tukey’s), and significance is denoted as ∗∗*p* < 0.01 and ∗∗∗∗*p* < 0.0001, while "ns" indicates no significance. Mean ± SD are shown. The results of the analysis are shown between MiaPaCa2 α2-6Sia 1.5 and the other populations. *D*-*G*, the number of data points is 172 for each of MiaPaCa2 α2-6Sia 1.5, 1.0, 0.5, and 0.25. *H*, the summarized characteristics of MiaPaCa2 α2-6Sia cells within the TME context are presented. Each *dot* signifies the outcome of simulations using the TME cell landscape data of each cancer case. α2-6Sia, α2-6 sialic acid; MD, multipolar cell division; TME, tumor microenvironment.

To estimate the impact of suppressive, permissive, and lethal microenvironments on cancer cells and conduct 3D TME simulations, we used TME cell landscape data from 193 cervical and 172 pancreatic cancer patients sourced from the TIMEDB (the ConsensusTME dataset) (51). Using the percentage of each type of cell provided by the TIMEDB, we calculated the total percentage of each category and determined the number of cells to be placed in the 3D TME based on percentages unique to each cancer case (refer to Data S7 for the calculated percentages). For example, if the suppressive cell percentage was determined to be 45%, we placed 45% of suppressive cells relative to cancer cells. Thus, if 500 deduced cancer cells are used in the simulation, 225 suppressive cells are placed in the 3D TME. We selected specific setting values for suppressive, permissive, and lethal strength, confirmed to impact the population expansion of deduced cancer cells, as detailed in Fig. S5. Four deduced cell populations, with varying levels of α2-6Sia (1.5, 1.0, 0.5, and 0.25), were employed for both HeLa and MiaPaCa2 cancers. Accordingly, simulations were conducted using percentage data obtained from the 193 cervical and 172 pancreatic cancer cases. In Figure 6, Figure 7, Figure 8, each dot represents the outcome of simulations using the TME cell landscape data from each cancer case. Specifically, in Figures 7 and 8, the blue, brown, light blue, and green dots indicate results obtained with α2-6Sia 1.5, 1.0, 0.5, and 0.25, respectively, illustrating the impact of varying α2-6Sia expression levels on simulation outcomes.

### Relationship between simulated cell population size and α2-6Sia expression levels

To examine the relationship between simulated cell population size and α2-6Sia expression levels, we performed a comparative analysis using HeLa α2-6Sia 1.0 cells and MiaPaCa2 α2-6Sia 1.0 cells within TME cell landscapes. We observed a positive correlation between the simulated cell population size and α2-6Sia expression levels, as shown in Figure 6*A* (R^2^ = 0.25 for HeLa α2-6Sia) and Figure 6*B* (R^2^ = 0.41 for MiaPaCa2 α2-6Sia). This finding suggests the significant role of α2-6Sia expression in maintaining the cell population.

### Characteristics of HeLa α2-6Sia cells within the TME

We investigated the relationship between the simulated population size of HeLa α2-6Sia cells and the proportions of suppressive (Fig. 7*A*), permissive (Fig. 7*B*), or lethal (Fig. 7*C*) cells, based on the cell landscape data of cervical cancer TME from the TIMEDB (51). The results revealed correlations between the simulated cell population size of HeLa α2-6Sia cells and the proportions of suppressive, permissive, and lethal cells within the TME of cervical cancer (Fig. 7, *A*–*C*; R^2^ > 0.1–0.55, where each dot represents the outcome of a simulation for each cancer case, see Data S7 for the proportions). Notably, negative linear correlations were observed with the proportions of suppressive (Fig. 7*A*) and lethal (Fig. 7*C*) cells, while a positive correlation was found with the proportion of permissive cells (Fig. 7*B*). These findings suggest that the proliferation of HeLa α2-6Sia cells is significantly influenced by the composition of immune cells within the TME for each cancer case. Among the HeLa α2-6Sia cell populations, the average cell population size of HeLa α2-6Sia 0.25 was observed to be 10% larger than that of HeLa α2-6Sia 1.5 (Fig. 7*D*). As expected, HeLa α2-6Sia 1.5 cells exhibited the highest levels of α2-6Sia expression, which gradually decreased in the HeLa α2-6Sia 1.0, 0.5, and 0.25 populations (Fig. 7*E*). The contribution of reproductive progeny produced through MD was marginal (Fig. 7*F*), with such progeny accounting for less than 0.4% of the HeLa α2-6Sia cell population (Fig. 7*G*).

These findings underscore the distinctive characteristics of HeLa α2-6Sia cells within the TME, highlighting the significant influence of the immune cell landscape on the proliferation of cell populations maintained by a small number of cells with stem cell–like characteristics (Fig. 7*H*). If HeLa cells represent some characteristics of cervical cancer, the immune cell landscape of the cervical cancer TME would have similar effects on cells in cervical cancer tissue.

### Characteristics of MiaPaCa2 α2-6Sia cells within the TME

We then explored the relationship between the simulated population size of MiaPaCa2 α2-6Sia cells and the proportions of suppressive (Fig. 8*A*), permissive (Fig. 8*B*), or lethal (Fig. 8*C*) cells. Unlike with HeLa α2-6Sia cells, we found almost no significant correlations (R^2^ < 0.15, where each dot represents the outcome of a simulation for each cancer case, see Data S7 for the proportions) for MiaPaCa2 α2-6Sia cells. This suggests that the proliferation of MiaPaCa2 α2-6Sia cells was relatively unaffected by the immune cell composition within the TME. Among different MiaPaCa2 α2-6Sia cell populations, we observed no significant differences in population size (Fig. 8*D*). MiaPaCa2 α2-6Sia 1.5 cells exhibited the highest levels of α2-6Sia expression, which gradually decreased in the MiaPaCa2 α2-6Sia 1.0, 0.5, and 0.25 populations, as expected (Fig. 8*E*). Concerning the number of reproductive cells produced through MD, there was a reduction in the number for MiaPaCa2 α2-6Sia 0.5 cells but an increase for the MiaPaCa2 α2-6Sia 0.25 cell population compared to MiaPaCa2 α2-6Sia 1.5 cells (Fig. 8, *F* and *G*, averaging from 0.48 in MiaPaCa2 α2-6Sia 1.5 cells to 0.66 in MiaPaCa2 α2-6Sia 0.25 cells). This suggests that although varying levels of α2-6Sia did not substantially impact the simulated cell population size, cells with altered chromosome numbers expanded within the MiaPaCa2 α2-6Sia cell population, particularly in those with 0.25-fold levels of α2-6Sia expression.

These results underscore the unique characteristics of MiaPaCa2 α2-6Sia cells (Fig. 8*H*). The majority of MiaPaCa2 α2-6Sia cells expressing α2-6Sia seemed to be less influenced by the TME cell landscape. In addition, cells with altered chromosome compositions accumulated in populations expressing lower levels of α2-6Sia, introducing genetic diversity within the MiaPaCa2 cell population.

## Discussion

In this study, we empirically obtained data related to the spatiotemporal behavior of individual cells using a computerized single-cell tracking system with two established cancer cell lines. We then deduced cell populations, signifying particular characteristics of cancer cells *in vivo*, using the cell fate simulation algorithm we previously developed (20). Additionally, we used a 3D TME simulation with data regarding TME-resident cells of cervical and pancreatic cancer patients to analyze the impact of microenvironments on these deduced cells, focusing on the role of α2-6Sia–related stemness and the reproductive cells produced through MD. To date, extensive data have been accumulated at the gene, protein, cell, and tissue levels regarding cancer cells and the TME. However, these data often represent the status of cancer tissue or cells at a specific moment within the long process of cancer development. Thus, we believe that incorporating a temporal context based on empirical data, coupled with simulations that account for the heterogeneous nature of the cancer cell population, will provide a more comprehensive understanding. Although the 3D TME simulation needs to be developed further, our approach will contribute to a deeper understanding of the dynamics of cancer development.

In the study on HeLa α2-6Sia cells, our results suggest that the status of the TME significantly influences the fate of the cells, mainly due to the lack of α2-6Sia expression, which is associated with stemness (35, 36, 37, 38, 39, 40). Although HeLa cells may only reflect some characteristics of cervical cancer cells, it is likely that some cervical cancer cell populations lacking α2-6Sia–related stemness may exhibit a similar fate as HeLa α2-6Sia cells. Accumulating more data on α2-6Sia–related stemness and the spatiotemporal fate of cervical cancer cells would thus be useful for accurate prediction of their fate. Unlike HeLa α2-6Sia cells, our study reveals that the fate of MiaPaCa2 α2-6Sia cells is relatively less influenced by the TME. This difference can be attributed to the fact that the majority of MiaPaCa2 cells express α2-6Sia, which is known to be related to resistance to cell killing (53, 54, 55). Consequently, cells with high α2-6Sia expression levels show reduced sensitivity to the TME. It has been reported that the glycan modification status with α2-6Sia is subject to dynamic regulation. For example, circulating β-galactoside α2-6Sialyltransferase 1 can modify cell surface glycoproteins with α2-6Sia when CMP-sialic acid is released from either inflammation-activated platelets (56) or tumor-induced platelet aggregation, which has long been known to be involved in tumor progression in several types of cancers, including lung, colon, breast, pancreatic, ovarian, and brain (57, 58, 59, 60, 61) This implies that cancer cells not expressing α2-6Sia can be converted into α2-6Sia–expressing cells through this inflammation or cancer-induced, platelet-related mechanism. Thus, if α2-6Sia expression plays a role in the resistance of pancreatic cancer cells in general, the levels of α2-6Sia expression on pancreatic cancer cells may be subject to various regulatory mechanisms.

With regard to α2-6Sia expression, we found that a high level of expression contributes to maintaining the integrity of cells by preventing them from undergoing CD, CF, and MD. In addition, cells expressing α2-6Sia exhibit stable growth, suggesting that once such a population is established, it can proliferate stably. This stable proliferation may, in turn, be recognized clinically as malignant cancer. However, while the accumulation of abnormalities through aberrant events is characteristic of cancer, high levels of stemness may counteract these events. Thus, the coexistence of high stemness and malignant characteristics presents a paradox. Perhaps cells with initially high stemness undergo a phase of reduced integrity in cell proliferation, which may be related to the reduction of surface α2-6Sia as indicated in our results. This reduction may allow for the acquisition of abnormalities, or cells with moderate stemness—sufficient to permit aberrant events but not prevent them—may be pivotal in cancer progression. If such cells regain high expression of α2-6Sia, it is possible that cells with higher malignancy would regain some attributes of stemness. As discussed above, in the circulation, β-galactoside α2-6sialyltransferase 1 is present and cancer cells activate platelets. One such scenario would be when cancer cells penetrate into the circulation, where sialyltransferase and platelets carrying CMP-Sia are present (57, 58, 59, 60, 61). Indeed, it has been demonstrated that invasive cancer cells can arise from populations with reduced α2-6Sia expression levels (62). Therefore, identifying cancer cells that balance the propensity for change with maintaining integrity may be crucial for understanding cancer progression.

Finally, we have showcased the framework of the 3D TME simulation using the suppressive, permissive, and lethal categories, which collectively represent the characteristics of cells that could influence the fate of cancer cells. The 3D TME simulation is carried out by defining the status of each cell and determining their interactions, allowing for flexible design of simulation conditions. Thus, when accurate information regarding the spatial location and quantitative biological effect of each cell type in the TME is available, the suppressive, permissive, and lethal categories can be replaced with specific cell types. While the current study only outlined the process of connecting cancer cell lines, their spatiotemporal changes, *in vivo* variation of cancer cell characteristics, and cell fate simulation, we believe that extending and refining this framework will enable a research approach that incorporates spatiotemporal aspects into the study of complex biological systems.

## Experimental procedures

### Cell culture and cell plating

HeLa cells were purchased from the American Type Culture Collection and cultured in Dulbecco’s modified Eagle’s medium supplemented with 10% fetal bovine serum in a humidified atmosphere with 5% CO^2^. MiaPaCa2 cells were also purchased from the American Type Culture Collection and maintained in Dulbecco’s modified Eagle’s medium supplemented with 10% fetal bovine serum and 2% horse serum under a 5% CO_2_-humidified atmosphere. *Mycoplasma* tests were routinely carried out. To ensure the authentication of these cells, morphological characteristics were recorded using live-cell imaging, and the videos were archived. For cell plating, approximately 3500 cells in 50 μl of cell suspension were added to the center of each well of a Lab-Tek II 8 Chamber Slide. Subsequently, 0.75 ml of culture medium was gently added to each well. The Lab-Tek II 8 Chamber Slide was placed on a microscope stage 24 h after plating.

### Culture conditions on the microscope stage

Cells were maintained in an environmental chamber (Live Cell Instruments) set at 37 °C with 80% relative humidity. To prevent an increase in medium pH within the Lab-TekII 8-well chamber, the CO_2_ concentration was controlled at 7.5%. To minimize medium evaporation, a NIR-DIC optimal glass lid (Live Cell Instruments) was placed on the Lab-Tek II 8 Chamber Slide. The typical medium evaporation rate was 10 μl/24 h, and as a result, 700 to 800 μl of the medium was added per well, ensuring cells could be cultured for at least 1 week without the need for medium changes.

For monitoring pH during long-term live-cell imaging, phenol red was included in the medium. Importantly, this inclusion did not interfere with NIR-DIC imaging.

### Live-cell imaging microscopy

A custom microscope was constructed using an Olympus IX81 microscope frame (Quorum Technologies). NIR-DIC imaging was chosen to minimize phototoxicity and reduce sensitivity to light distortion caused by plastic partitions and surface irregularities in the culture medium. The microscope featured dual imaging capabilities, combining NIR-DIC and fluorescent imaging for both live and fixed samples. Two distinct light paths were established within the microscope. For NIR-DIC imaging, NIR light was generated using light-emitting diodes and passed through a polarizer, Nomarski prism, and condenser before illuminating the cells. The returning light passed through an objective lens and Nomarski prism, ultimately reaching the first charge-coupled device (CCD) camera (Camera 1, Hamamatsu Photonics, Image EM, 512 × 512 pixels). In the case of fluorescent imaging, laser light was directed onto cells stained with fluorescence-conjugated antibodies or proteins. This was accomplished by passing the laser light through a Nipkow disk (Yokokawa-Quorum Technologies) and an objective lens to excite fluorophores. The emitted light was collected by the Nipkow disk and captured using the second CCD camera (Camera 2, Hamamatsu, Image EM, 512 × 512 pixels). The system allowed the use of ×10–×40 objectives. In this study, an Olympus ×10 dry objective (UPlanSApo, 10×/0.40 NA, α/0.17/FN26.5) or ×20 dry objective (UPlanSApo, 20×/0.75 NA, α/0.17/FN2G.5) was employed. To achieve equivalent magnifications of ×15 and ×30, respectively, a ×1.5 coupler (Quorum Technologies) was inserted into the light path leading to the CCD cameras. A precision piezo XY stage with absolute measurement capabilities was utilized. An environmental chamber (Live Cell Instrument) was mounted on the piezo stage. The entire microscope system, along with the piezo XY stage, was controlled using MetaMorph software (Quorum Technologies) on a Windows computer. Images were acquired using MetaMorph’s multidimensional acquisition (MDA) mode. For NIR-DIC imaging, a 34 ms exposure time was used. The exposure time for fluorescence imaging was adjusted based on the fluorescence intensity. Typically, 20 to 80 z-planes at 1 μm intervals were acquired for NIR-DIC imaging. The resulting z-plane NIR-DIC images, captured by camera 1, were saved as 512 × 512 pixel multilayer TIFF files. For fluorescence imaging, z-plane NIR-DIC and fluorescence images generated by CCD cameras 1 and 2, respectively, were merged using a macro program in MetaMorph. This produced 512 × 1024 pixel multilayer TIFF files. To manage data, computer 1 (Windows computer) was connected to computer 2 (Macintosh) *via* an Ethernet cable. Computer 2 was responsible for creating live-cell videos. Image files generated by computer 1 were promptly transferred to computer 2 using an in-house *file transfer* software program, with subsequent deletion of the image files from computer 1.

### Developed software for a single-cell tracking system

Image files produced by MetaMorph underwent processing through a series of in-house software tools (Fig. S1). The computerized single-cell lineage tracking analysis system consisted of two computers: computer 1 (Windows) controlled the microscope, while computer 2 (Macintosh) handled image processing, single-cell tracking, and data analysis tasks. Computer 1 was equipped with commercially available image acquisition software (MetaMorph) to oversee microscope control and image file creation. Computer 2 utilized several in-house software programs (italic names indicate the in-house software, DOI: 10.5281/zenodo.12988726). *Image processing controller* managed other software programs. *File transfer* and *map* facilitated communication between computers 1 and 2. *File converter* imported images generated by other microscopes. *Name assignment*, *focal image selection*, and *contrast set* were responsible for creating live-cell videos. *Data backup* controlled file archiving and backup procedures. *Outline drawing* performed image segmentation and established the object segmentation library. *Movie viewer* played movies and fine-tuned image quality. *Object tracking controller*, in conjunction with *automatic object tracking*, created the cell lineage database and facilitated data verification. *Data analysis* offered various options for data analysis. The data generated through this process was subsequently used for cell fate simulation and 3D TME simulation.

### Setting fields of views and adjustment of focus

Cells cultured in each well of the Lab-Tek II 8 Chamber Slide were simultaneously monitored. To cover the area of interest with multiple fields of view (FOVs), 2D image acquisition arrays were established in each well (20). The configuration of FOVs for these arrays and the necessary focus adjustments were executed using *map* software in conjunction with MetaMorph. *Map* software was specifically designed to coordinate and share information on the xyz positions between MetaMorph on computer 1 and software on computer 2. To initiate this coordination, the objective lens was initially positioned at the left corner of well 2 of the Lab-Tek II 8 Chamber Slide, and this position was registered in *map* software. Subsequently, the outline of a Lab-Tek II 8 Chamber Slide was traced on the screen. The objective lens was then directed to a FOV of interest within a well using the outline as a guide. To view the cells in the surrounding area, 5 × 5 FOV dimensions were captured using MetaMorph, which generated xyz position data for each FOV. A suitable area for live-cell imaging was searched by repeating this process. When the suitable area was found, xyz position data saved on computer 1 was transferred to computer 2 using *map* software. Then, the selection of the area to be imaged was finalized by setting the 2D image acquisition arrays that define each position of FOV using *map* software. Next, the optimal objective z position for each FOV was determined, and the xyz position information created by *map* software was compiled into a MDA file format. This MDA file was then used by MetaMorph to acquire an image of each FOV. The complied MDA file, created by *map* software, was transmitted to computer 1 and uploaded to MetaMorph. Subsequently, MetaMorph performed MDA image acquisition to generate images for the corresponding FOVs.

### Starting image acquisition

The first round of image acquisition was initiated by MetaMorph through MDA image acquisition (Fig. S1). Subsequently, *file transfer* transmitted the multilayer TIFF files (512 × 512 pixels) generated by MetaMorph (computer 1) to computer 2. To facilitate file archiving, each multilayer TIFF file was assigned a specific file name using *name assignment*. The image files underwent processing using *focus image selection*, which involved selecting a focal image from among the images within a multilayer TIFF file or generating an all-in-focus image. The all-in-focus image was constructed by selecting the optimal grayscale value from the *z*-axis data for each pixel. Following this, the contrast of the resulting images was adjusted, and image backgrounds were corrected using *contrast set*. Since the background patterns varied for each FOV, custom background patterns were generated using data from single-layer TIFF files. Each of these background patterns was then applied to the corresponding FOVs. Ultimately, stitched images were created using *contrast set* after contrast adjustment, background correction, and fine-tuning of the position for each FOV.

### Automated long-term live-cell imaging video creation

Following the initial setup, automated image acquisition commenced. MetaMorph captured images of each FOV at 10-min intervals. Subsequently, after each round of image acquisition, the process involving *file transfer*, *name assignment*, *focus image selection*, and *contrast set* was coordinated by *image processing controller*. The status of the images could be monitored using *image viewer*, and *data backup* generated backup files as needed. This process continued until the completion of live-cell imaging. Live-cell imaging was performed at least three times for HeLa and MiaPaCa2 cells, and one video from each was selected for single-cell tracking (20).

### Staining of cells with SNAI-TRITC

Fluorescent imaging was conducted at the end of the live-cell imaging process. Before removing the Lab-Tek II 8 Chamber Slide from the microscope stage, a snapshot was captured using *map* software to document the position of a unique object, such as a cell with a distinct shape. Subsequently, the chamber slide was removed from the stage, and the cells underwent three washes with PBS. Following this, the cells were fixed with 3.7% paraformaldehyde for 15 min at room temperature and then washed three additional times with PBS. Next, the cells were treated with Carbo-Free Blocking Solution (×1) (Vector) for 1 h at room temperature and subsequently incubated with tetramethylrhodamine (TRITC)-labeled SNAI (E-Y Laboratories) diluted with Carbo-Free Blocking Solution (×1) to a concentration of 50 μg/ml for 1 h at 4 °C. Following this incubation, the cells were washed with PBS three times and exposed to 4′,6-diamidino-2-phenylindole (DAPI). This was performed by diluting two drops of NucBlue Fixed Cell ReadyProbes reagent with 1 ml Carbo-Free Blocking Solution (×1) for 15 min at room temperature, followed by three additional washes with PBS. The Lab-Tek II 8 Chamber Slide was then returned to the microscope stage, and its position was adjusted using the snapshot, which was taken before removing the chamber slide from the microscope stage. As returning it to the identical position as during live-cell imaging proved to be challenging, the shift in the x and y position from the live-cell imaging setup was calculated, and a new MDA file, accounting for this shift in position, was generated. Following the upload of this new MDA file, fluorescent image acquisition was conducted by MetaMorph. Fluorescent imaging was conducted with a 250 ms exposure using lasers with wavelengths of 403 nm and 491 nm for DAPI and TRITC, respectively.

### Segmentation of NIR-DIC images

Grayscale live-cell imaging videos were then subjected to image segmentation. To this end, we developed a method referred to as stepwise area expansion, which was performed by *outline drawing* software (Fig. S2). Four images were created from the original grayscale image by applying four different threshold (TH) values (TH1–4, Fig. S2*A*). First, pixels above the TH value (*e.g.* 200, 170, 140, and 110 of 0–255 grayscale for TH1, 2, 3, and 4, respectively) were extracted from the grayscale image. Segmentation was then performed step by step, starting from the TH1 image (Fig. S2*B*). Connectivity analysis was performed on the TH1 image to identify groups of pixels that were attached and an edge circle that surrounded the group of pixels was then determined (Fig. S2*B*, Panel a, pink circles). The TH1 edge circles were overlaid on the corresponding grayscale image (Fig. S2*B*, Panel b). The pixels composing the circles were referred to as the original pixels. Using the overlaid grayscale image as a reference, the software examined the pixel values for each original pixel. The values of pixels located in a circle from 12 o’clock from the original pixels were examined (Fig. S2*B*, panel b and line extension (magnified)). The edges of the pixels were extended until the values became either 100 (representing the grayscale background) or 50 above or below the original pixel, as long as those extensions were toward the outside of the edge circles. The final pixel positions were determined by this technique. Similar examinations were performed for the other three directions (Fig. S2*B*, Panel b: 3, 6, and 9 o’clock directions; yellow, light blue, and blue lines, respectively), and the final pixel positions were then linked to create four directional lines corresponding to the 12, 3, 6, and 9 o’clock directions (Fig. S2*B*, Panel c and edge linking (magnified), L 12′, L3′, L6′, and L9′). Finally, the four lines were linked to make an edge circle (Fig. S2*B*, Panel d, green circles), which completed the initial segmentation for the first TH1 image. The TH1 edge circles were then overlaid on the TH2 image and another connectivity analysis was performed, except for the TH1 edge circle areas (Fig. S2*B*, Panel e, green circles). Two types of edge circles (Fig. S2*B*, Panel e, green and pink circles) were thus created at this stage. The pink circles that emerged in the TH2 image were overlaid on the corresponding grayscale image, the pixel value of each original pixel in the pink circles was examined, the final pixel positions were determined (Fig. S2*B*, Panel f), and the positions were linked to create four lines (Fig. S2*B*, Panel g) and a new edge circle was created (Fig. S2*B*, Panel h), as described for TH1. The software applied a different expansion approach for the green edge circles that had been determined during TH1 processing (Fig. S2*B*, Panel e, green circles): the location of each pixel on the edge of the circles (Fig. S2*B*, Panel k) was moved by 1 to 4 pixels outside the original (Fig. S2*B*, Panel l, edge expansion (magnified)) to create the red circles (Fig. S2*B*, Panel l). Both the green TH2 (Fig. S2*B*, Panel h) and red TH2 circles (Fig. S2*B*, Panel l) were then overlaid onto the TH2 image (Fig. S2*B*, Panel i), and the circle overlaps were removed to create new edge circles (Fig. S2*B*, Panel j). Those new edge circles were then applied to TH3, and the above-mentioned processes were repeated for TH3 and TH4 images. Areas surrounded by edge circles were numbered.

This approach proves valuable in scenarios where cell segmentation encounters challenges. For instance, when a flat cell coexists with larger, bright cells that are easily distinguishable in the NIR-DIC image, the flat cell may become obscured by the brightness of the larger cells, making segmentation difficult. In the stepwise area expansion method, the initial step involves defining the boundaries of the brighter objects within the TH1 image. Since the TH1 image typically does not encompass the darker flat cell, there is a possibility that the flat cell lies outside this boundary. In subsequent steps utilizing images created with lower TH levels, the darker flat cell can be included in the image. By excluding the area within the previously determined boundary during segmentation, the region corresponding to the flat cell can be segmented. This approach facilitates accurate segmentation of grayscale images that contain cells with varying brightness levels and shapes. Fig. S2*A* (depicted by blue lines) provides examples of segmentation outcomes using both high- and low-density HeLa cell cultures.

### Assignment of cell lineage number and cell number

To assign cell lineage numbers and cell identifiers to the cells within the time 1 image, a verification and correction process was carried out for the segmentation results using the *object tracking controller* software. In cases where multiple segments were incorrectly associated with a single cell, one segment was chosen to represent the cell, and segments were merged as needed. Conversely, if multiple cells were grouped within a single segment, the segment was divided to accurately represent each individual cell. The cells identified within the time 1 image were classified as progenitor cells, and a unique cell lineage number was assigned to each progenitor cell. Progenitor cell numbers were designated as 0. Data was then recorded in the cell lineage database.

### Automated object tracking (single-cell tracking)

After assigning cell lineage numbers to segmented areas, the automatic cell tracking process was initiated using the *automatic cell tracking* software. Fig. S3 provides an overview of the automatic tracking process for the segmented area, outlined by green lines. In Fig. S3*A* (time A), orange characters represent segmented areas identified as cell representations. The blue area with a white asterisk denotes the cell being tracked, while the yellow and magenta areas indicate segmented areas representing neighboring cells. In the subsequent time point (Fig. S3*B*, time A + 1), the positions of cells and segmentation patterns changed. To perform single-cell tracking, it was essential to determine the segmented area corresponding to the cell being tracked. To achieve this, the blue area from time A was overlaid onto the time A + 1 image (Fig. S3*C*). However, the blue segmented area from time A did not exist in time A + 1; instead, it overlapped with the light blue and white segmented areas. The *automatic cell tracking* software could not ascertain which area corresponded to the tracked cell. To resolve this, the yellow and magenta areas were overlaid on the time A + 1 image (Fig. S3*D*). This process revealed that the light blue area was related to the yellow area. Furthermore, the magenta area overlapped with the white area, but the larger portion of the white area overlapped with the blue area. Consequently, the *automatic cell tracking* software determined that the white area represented the cell being tracked (Fig. S3*E*). This process was systematically repeated for all cells recorded in the database.

### Tracking data verification

Since achieving 100% accuracy in automatic single-cell tracking is not always feasible in practice, the tracking data underwent verification using the interactive features of the *object tracking controller* software. If errors were identified, the single-cell tracking data were corrected, ultimately enabling the generation of nearly 100% accurate single-cell tracking data.

### Data analysis software

Bioinformatics analysis was conducted using the *data analysis* software, which encompasses a range of functions such as generating cell lineage maps, calculating the individual cell doubling time, and determining cell population expansion curves. Notably, cell fate simulation is one of the features offered by this software.

### Staining of cells with antibodies against a stem cell marker

HeLa and MiaPaCa2 cells were fixed with 3.7% paraformaldehyde for 15 min at room temperature, followed by three additional washes with PBS. Subsequently, the cells underwent treatment with Carbo-Free Blocking Solution (×1) (Vector) for 1 h at room temperature. After this blocking step, the cells were incubated with the following primary antibodies or lectin: Anti-CD133 antibody (Developmental Studies Hybridoma Bank) at a dilution of 1:50, which corresponds to 5 μg/ml, in Carbo-Free Blocking Solution (×1) for 30 min at room temperature, anti-TAG-72 antibody (B72.3) at a 1:50 dilution in Carbo-Free Blocking Solution (×1) for 1 h at room temperature, or FITC-labeled rBC2LCN (Wako) at a 1:100 dilution in Carbo-Free Blocking Solution (×1) for 30 min at room temperature. Following the primary antibody or FITC-labeled rBC2LCN incubation, the cells were washed three times with PBS. For cells incubated with primary antibodies (anti-CD133 and anti-TAG-72), a secondary antibody (Invitrogen, goat anti-mouse IgG Alexa Fluor 488) diluted 1:1000 was applied for 1 h at room temperature. After the secondary antibody incubation, the cells were subjected to three additional washes with PBS. Subsequently, the cells were stained with DAPI and underwent three final washes with PBS. Fluorescent imaging was conducted with a 250 ms exposure using lasers with wavelengths of 403 nm for DAPI and 491 nm for Alexa Fluor 488 and FITC.

### Generation of deduced cell populations with varied α2-6Sia expression levels

The initial step in generating the deduced cell population involved creating a α2-6Sia expression list (Fig. S4*A*). In this list, we considered examples from lineage 1 and lineage 2. Indirect immunofluorescence using TRITC-tagged SNA1 was performed at the end of live-cell imaging, allowing us to determine the SNA1 binding levels of cells present at the end of the imaging period. Lineage1 exhibited no expression of α2-6Sia in any of the cells present at the end of live-cell imaging, while lineage 2 showed various levels of α2-6Sia expression in its cells. Based on the α2-6Sia expression levels of cells at the end of live-cell imaging, we traced back the α2-6Sia expression levels along the cell lineage map to determine the expression levels of their parent cells. This was done by calculating the average expression of daughter cells, thus estimating the evolution of α2-6Sia expression levels within a cell lineage. Subsequently, we compiled a α2-6Sia expression list that included the α2-6Sia expression levels of all tracked HeLa or MiaPaCa2 cells. For the lineages 1 and 2 examples, the list contained 17 data items with a value of 0 and 17 data items with varying levels of expression values.

Next, we generated the deduced cell population using the cell fate simulation algorithm (20), as illustrated in Fig. S4*B*. This algorithm analyzed cell lineage data and calculated the probability of certain types of cell events occurring following a given event, along with the time intervals between events. The combinations of events that the algorithm analyzed was previously described (20). The cell fate simulation algorithm employed these probabilistic values to generate a deduced cell population. Initially, the algorithm assigned the length of time to the first event (the event that occurred in the progenitor cell) to the progenitors. In the actual process, 500 progenitors were generated, each at a different stage of the cell cycle, resulting in varying time intervals until the first event.

Subsequently, a cellular event was assigned to a progenitor cell based on the probabilistic values. If a BD was assigned, two daughter cells were created. In example 1, a longer time interval until the next event was assigned than example 2. After the assignment, the algorithm referred to the cell doubling time (the time between BDs) of α2-6Sia–expressing cells. As 80% of the cell doubling times for α2-6Sia–expressing cells fell within ±10% of the average cell doubling time for these cells, the algorithm checked whether any of the α2-6Sia–expressing cells had cell doubling times within ±10% of the time assigned to daughter cells. If no such cells were found, α2-6Sia expression levels were not assigned to the daughter cells (example 1). However, if some cells met this criterion, α2-6Sia expression levels were assigned to the daughter cells (example 2). This approach allowed us to generate deduced cells with reproductive abilities similar to those of α2-6Sia–expressing cells.

Subsequently, the algorithm assigned α2-6Sia expression levels to the daughter cells by referring to the α2-6Sia expression level list (Fig. S4*C*). A random value was generated to select one value from the list, which was then assigned to one of the daughter cells. In the case of the lineage 1 and 2 examples (Fig. S4*A*), there was a 50% chance of selecting value 0. As the variation in α2-6Sia expression levels typically fell within ±15%, the algorithm determined the α2-6Sia expression level of the second daughter cell based on the first daughter cell's level, accounting for the ±15% variation. Finally, α2-6Sia expression levels were modified to create deduced cell populations with various levels of α2-6Sia expression. This was achieved by multiplying the value of the α2-6Sia expression by factors such as 1.5, 0.5, and 0.25. In cases where the calculated value exceeded the highest α2-6Sia expression value observed in the HeLa or MiaPaCa2 cell population, the value was capped at the highest observed level.

### The 3D TME simulation methodology

The outline of the 3D TME simulation was presented in text S2 (Pseudocode). The codes for the 3D TME were developed using C, C++, and objective-C with the utilization of SceneKit (macOS 12). To execute the 3D TME simulation, various parameters pertaining to graphic display and simulation execution were configured. Graphic display parameters for both cancer cells and immune cells included options such as cell shape (sphere, box, or capsule), displayed cell size, color, alpha value, motility of cells, nucleus size, and nucleus color. These parameters could be individually adjusted for cancer cells, cells representing suppressive, permissive, and lethal environments. Additionally, for fluorescence display reflecting the quantitated fluorescent value of each cancer cell, the level of expression could be visualized using either a heat map scale (ranging from blue to red, indicating low to high expression) or manually set colors.

Concerning suppressive, permissive, and lethal cells, the ratio of each cell type relative to cancer cells can be specified. In a typical simulation, 500 progenitors of the deduced cell population were employed, and the numbers of suppressive, permissive, and lethal cells were determined based on the specified ratio relative to the total of 500 cells. For instance, if the ratio was set to 1:0.5 (cancer cells to either suppressive, permissive, or lethal cells), and then 250 of the chosen cell types were placed accordingly. These ratios could be customized independently for each category of cells, and the range of the ratio as well as the increment could be set. For instance, the 3D TME simulation could be automatically conducted by accounting for the defined range and increment. As an example, if the range was set from 1:0 to 1:1 for each category with an increment of 0.1, this would result in 1331 simulations being carried out automatically.

The strength of the impact could also be configured. In the case of suppressive and permissive cells, the setting values, such as 10, represented the percentage for prolonging or shortening the cell doubling time. For example, if a deduced cell had a doubling time of 25 h and encountered a suppressive cell, the doubling time would be extended by 10%, making it 27.5 h. In the case of encountering permissive cells, the cell doubling time for deduced cells and their progeny would be reduced by 10%. However, this reduced cell doubling time could not fall below the shortest cell doubling time observed within the deduced cell population, and in such cases, the shortest time was assigned.

As for lethal cells, the setting value represented the probability of inducing CD. For instance, a setting of 100 indicated a 100% chance of inducing CD when cancer cells encountered a lethal cell. When CD was triggered for a cancer cell, all its progeny were removed from the cell lineage database. The range of these setting values, along with the increment, could be customized, and the 3D TME simulation could be automatically executed, considering the specified increment value. The resistance of cancer cells to suppressive and lethal effects could also be set. For example, if the maximum resistance level was defined as 5-fold, then cancer cells subjected to suppressive or lethal effects would have a 20% chance (one in five chance) of experiencing these effects.

These cells were positioned within spheres, each with a radius of 50 pixels. Cells were randomly distributed within the space, typically with a radius of 6 pixels from the cancer cell to search for suppressive, permissive, and lethal cells. Under this setting, neighboring cancer cells were found within an 80% chance within the 6-pixel radius. Multiple cell lineage data of the deduced cell population can be loaded and the primary simulation process commenced with cancer cells identified at time 1 and involved searching within a specified radius. If suppressive, permissive, and/or lethal cells were discovered, the two nearest cells were selected. If the nearest cell was a lethal cell, the resistance level of a cancer cell was initially considered, followed by the strength factor. If a cancer cell was determined to be killed, the CD process was executed. If the nearest cell was a suppressive cell, and the second nearest cell was not a permissive cell, the resistance level of a cancer cell was initially taken into account, followed by the strength factor. If the cancer cell doubling time was prolonged, the process for prolongation was executed. If the nearest cell was a permissive cell, and the second nearest cell was not a suppressive cell, the strength factor was taken into account. If the cancer cell doubling time was determined to be reduced, the process for reduction was executed. If the nearest cell was a suppressive cell, and the second nearest cell was a permissive cell, the distances of both cells from a cancer cell were calculated, and the suppressive strength was adjusted, considering the presence of permissive cells. For instance, if the initial strength of both suppressive and permissive cells was set to 50 and the distance of suppressive and permissive cells from a cancer cell was 3 and 6 pixels, respectively, the effect of permissive strength was considered as half of the suppressive strength, resulting in an adjusted suppressive strength of 25 (50 (suppressive) −25 (permissive)). If the nearest cell was a permissive cell, and the second nearest cell was a suppressive cell, a similar adjustment process was applied. These processes were iteratively carried out for each cancer cell identified at each time point and continued until reaching the final time point.

### Parameters for the methodology of 3D TME simulation

In the 3D TME simulation, the motility of cancer cells, suppressive cells, permissive cells, and lethal cells was restricted to a maximum displacement of 2 pixels from their initial positions within the 3D TME sphere. This constraint was crucial to confine cancer cells and immune cells within the simulation sphere. However, this constraint also fixed the relative positions of cancer cells concerning suppressive, permissive, and lethal cells. For example, once a cancer cell was positioned within the 3D TME, the spatial relationship between that cancer cell and nearby suppressive, permissive, or lethal cells remained constant throughout the cell's simulation. As a result, if suppressive, permissive, and/or lethal cells located within 6 pixels of a cancer cell were monitored at each time point, the same combination of suppressive, permissive, and/or lethal cells would persist. This implied that if a suppressive cell was initially the closest to a cancer cell, it would always remain the closest cell throughout the cancer cell's lifetime.

During the simulation, when a cancer cell experienced a suppressive or permissive effect, it did not receive additional suppressive or permissive effects. In other words, if a cell encountered a suppressive cell and had the effect induced, it would not undergo another suppressive effect. This rule applied uniformly to all progeny cells. However, these progeny cells could subsequently encounter additional suppressive, permissive, or lethal effects.

When cancer cells produced daughter cells, the locations of these daughter cells within the 3D space were randomly determined within 6 pixels from the parent cancer cell. This randomization altered the relative positions of suppressive, permissive, and lethal cells concerning the daughter cells compared to their parent cell. Consequently, if a suppressive effect was applied to a daughter cell due to its parent cell's previous interaction with a suppressive cell, and the daughter cell encountered a permissive cell, the daughter cell would be subject to the permissive effect. Similarly, daughter cells could receive a suppressive or lethal effect if either of these cells became the closest to a daughter cell.

### Other parameters for 3D TME simulation methodology

Upon placing suppressive, permissive, and lethal cells in the 3D TME, individual cell lineages were established, each characterized by its specific attribute (*e.g.*, suppressing, permissive, or lethal). The 3D TME simulation algorithm executed simulation processes by referencing these attributes. This flexibility allowed the creation of comprehensive simulation scenarios by modifying attributes, including alterations at specific stages of the simulation to reflect changes in cell behavior.

Each cancer cell was associated with attributes, such as α2-6 Sia expression levels, and by incorporating multiple attributes, the simulation could encompass various characteristics of cancer cells. Thus, the 3D TME simulation, based on lineage data and associated attributes for each cell, facilitated the execution of complex simulations and provided that the biological implications of each cell's attributes were defined.

### Statistical analysis

Statistical analyses were conducted using Prism 10 software (GraphPad Software).

## Data availability

All data are included in the article and/or supporting information. The software was deposited to GitHub; DOI 10.5281/zenodo.12988726. Any questions regarding data availability can be directed to the corresponding author.

## Supporting information

This article contains supporting information (53, 54, 55).

## Conflict of interests

The authors declare that they have no conflicts of interest with the contents of this article.
