# Supplementary material for: Cell fate simulation reveals cancer cell features in the tumor microenvironment
Source: J Biol Chem. 2024 Aug 20;300(9):107697. doi: 10.1016/j.jbc.2024.107697 (PMC11419826; doi:10.1016/j.jbc.2024.107697)
Supplement: Supplementary Text 2 [file mmc2.docx]

**Supplementary Text 2**

**Pseudocode**

Setting simulation parameters:

Display and setting parameters (cancer cells):

* Cell Shape

* Size

* Motility

* Alpha value

* Fluorescent display mode (heat map or manual color selection):

* Nucleus size

* Nucleus color

Display and setting parameters (TME cells):

* Cell Shape

* Size

* Motility

* Alpha value

* Nucleus size

* Nucleus color

*Color for Suppressive, Permissive, and Lethal microenvironments.

* Ratio of microenvironments to place for Suppressive, Permissive, and Lethal

microenvironments relative to cancer cells; Each ratio can vary.

* Impact strength of Suppressive, Permissive, and Lethal microenvironments on cancer

cells; Each value can vary.

* Cancer cell resistance to suppressive and lethal cells based on stemness.

General parameters:

* Diameter of spheres

* Search distance

Place TME cells in the sphere.

Place cancer cells in the sphere.

For x = simulation time:

* Calculate the distance of a cancer cell to Suppressive, Permissive, and Lethal

microenvironments in 3D TME.

* Select the two nearest TME cells within the search distance.

* Identify the type of cells: Suppressive, Permissive, and Lethal microenvironments.

* If a Lethal cell is the nearest cell:

* Calculate the resistance of cancer cells to the Lethal effect (based on the stemness level)

* Determine whether the cell death procedure is executed

* If Yes, obtain the Lethal strength

* Determine whether the cell death procedure is executed

* If Yes, execute the cell death procedure

* If a Suppressive microenvironment is the nearest and the second nearest

microenvironment is not a Permissive cell:

* Calculate the resistance of cancer cells to the Suppressive effect (based on the stemness

level)

* Determine whether the cell doubling time prolongation procedure is executed

* If Yes, obtain the Suppressive strength

* Determine whether the cell doubling time prolongation procedure is executed

* If Yes, execute the prolongation procedure

* If a Permissive microenvironment is the nearest and the second nearest microenvironment is

not a Suppressive cell:

* Determine whether the cell doubling time shortening procedure is executed

* If Yes, execute the shortening

* If a Suppressive microenvironment is the nearest and the second nearest microenvironment

is a Permissive cell or vice versa:

* Obtain the Suppressive strength and Permissive Strength

* Calculate the distance from a cancer cell to Suppressive and Permissive

microenvironments

* Adjust Suppressive or Permissive strength

* Execute the prolongation or the shortening procedure

Repeat

Export results

Display results in 3D

End

**Legends for Supplementary Text 2.**

To perform the 3D TME simulation, several parameters needed to be defined. These parameters included: [1] Resistance levels of α2-6Sia-expressing Cells: Determining the resistance levels of cells expressing α2-6Sia to suppressive and lethal effects, [2] Cell ratios: Establishing the relative numbers of Suppressive, Permissive, and Lethal cells in relation to cancer cells, and [3] Impact strength: The strength of the influence exerted by Suppressive, Permissive, and Lethal microenvironments on cancer cells.

As for [1], cells expressing α2-6Sia demonstrate approximately 2 to 4.5-fold resistance to TNF, cisplatin, and gemcitabine compared to non-expressing cells (1-3). To take into account this, we assigned a 5-fold resistance factor to cancer cells expressing the highest level of α2-6Sia when subjected to Suppressive or Lethal effects, relative to non-expressing cells. In this scenario, when a cancer cell encounters, for instance, a Lethal cell, there is a 20% chance of the cancer cell being eliminated (a 1 in 5 chance). Conversely, if a cancer cell lacks α2-6Sia expression and encounters a Lethal cell, it is eliminated with a 100% probability. The degree of resistance for other cells was calculated based on their α2-6Sia expression levels.

Regarding parameter [2], we conducted a 3D TME simulation involving cancer cells and either Suppressive, Permissive, or Lethal microenvironments to determine the ratio that could effectively observe the impact of these cells on cancer cells (Fig. S5 *A-H*, using HeLa α2-6Sia 1.0 and MiaPaCa2 α2-6Sia 1.0 cells). Within the ratios of deduced cancer cells to Suppressive microenvironments, ranging from 1:0.1 to 1:0.9 (Fig. S5 *A* and *E*) and the ratios of cancer cells to Lethal cells, ranging from 1:0.1 to 1:0.46 (Fig. S5 *C* and *G*), we observed a decrease in the expansion of the cancer cell population, as expected. This reduction was primarily due to the prolongation of cell doubling time or cell elimination, with Lethal cells exerting a stronger effect compared to Suppressive microenvironments. However, when we performed the simulation by varying the ratio of Permissive microenvironments, it did not have a significant impact on the expansion of the cell population. This observation can be attributed to the fact that the minimal cell doubling time was restricted from falling below the shortest doubling time observed within the deduced cell population (Fig. S5 *B* and *F*). Deduced cancer cells, which had a doubling time close to the shortest observed, may have experienced a comparatively lower influence from the presence of Permissive microenvironments. Consequently, we conducted the 3D TME simulation with a fixed ratio of 1:0.2 for deduced cancer cells to Suppressive cells and a varying ratio of Permissive microenvironments (cancer cells: Suppressive cells: Permissive microenvironments; 1:0.2:0.2 to 1:0.2:0.5, Fig. S5 *D* and *H*). This approach allowed the effects of Permissive microenvironments to counteract the prolongation of cell doubling time caused by Suppressive microenvironments, resulting in the reduction of cell doubling time. As anticipated, we observed that Permissive microenvironments effectively mitigated the reduction in population expansion induced by Suppressive microenvironments. These results indicate that the effects of Suppressive, Permissive, and Lethal microenvironments can be observed within the ratio range of 1:0.1 to 1:0.9 for Suppressive and Permissive microenvironments and of 1:0.1 to 1:0.46 for Lethal microenvironments.

Regarding parameter [3], we performed a 3D TME simulation to determine the range of strength for Suppressive, Permissive, and Lethal microenvironments' effects on cancer cells (Fig. S5 *I* and *P*). For Suppressive cells, when the strength was set to 10, the cell doubling time of the cells encountering the Suppressive microenvironment and its progeny was extended by 10% (for instance, if the doubling time was 25 hours, it was extended to 27.5 hours). Similarly, with a strength setting of 10 for Permissive microenvironments, the cell doubling time of the encountering cell and its progeny was reduced by 10%. If the reduced doubling time fell below the shortest doubling time within the deduced cell population, it was capped at the shortest time. For Lethal microenvironments, the chance of inducing cell death was set. For example, setting values of 20 implied that cell death occurred with a 20% chance when cancer cells encountered a Lethal microenvironment. We conducted 3D TME simulations by varying these setting values and found a similar trend for Suppressive, Permissive, Lethal, and the combination of Suppressive and Permissive microenvironments, as analyzed by cancer cell to immune cell ratio (Fig. S5 *I-P*). These results suggest that the effects of Suppressive, Permissive, and Lethal microenvironments can be analyzed within the range of 10% to 100% for Suppressive and Permissive microenvironments and a 10% to 50% chance for Lethal microenvironments.

The 3D TME simulation process involved the identification of Suppressive, Permissive, and Lethal microenvironments within a specified spatial range around a cancer cell. At each time point, Suppressive, Permissive, and/or Lethal microenvironments within this spatial range were located, and the nearest cell to the cancer cell was selected. If the selected cell was a Lethal microenvironment, first, the resistance levels related to the α2-6Sia expression on cancer cells were considered, followed by the Lethal strength. If cell death was determined to be induced, the process of eliminating the cancer cell and its progeny was executed. If the nearest cell was a Suppressive cell, the cell doubling time was extended, after considering α2-6Sia expression levels followed by the Suppressive strength. When the nearest cell was a Permissive microenvironment, the cell doubling time was reduced, accounting for the Permissive strength. In cases where the nearest and second nearest cells were a combination of Suppressive and Permissive microenvironments, or vice versa, the relative distance between both cells was factored into the calculation of strength. This process was applied to each deduced cancer cell, and the iterative simulation continued until reaching the final time point. In Fig. 5*B*, a graphical representation of the 3D TME is presented, depicting views at 0, 2000, 4000, and 6000 minutes. The simulation was performed with HeLa α2-6Sia 1.5 cells in the presence of Suppressive, Permissive, and Lethal microenvironments, with the influence strength of each category set to 0 (Control) or Lethal strength 100.

**References**

1. A.T. Holdbrooks, C.M. Britain and S.L. Bellis, ST6Gal-I sialyltransferase promotes tumor necrosis factor (TNF)-mediated cancer cell survival via sialylation of the TNF receptor 1 (TNFR1) death receptor, *J. Biol. Chem.*, **293**, 2018, 1610–1622.

2. A. Chakraborty, K.A. Dorsett, H.Q. Trummell, E.S. Yang, P.G. Oliver, J.A. Bonner, et al., ST6Gal-I sialyltransferase promotes chemoresistance in pancreatic ductal adenocarcinoma by abrogating gemcitabine-mediated DNA damage, *J. Biol. Chem.*, **293**, 2018, 984–994.

3. M.J. Schultz, A.F. Swindall, J.W. Wright, E.S. Sztul, C.N. Landen and S.L. Bellis, ST6Gal-I sialyltransferase confers cisplatin resistance in ovarian tumor cells, *J. Ovarian Res.*, **6**, 2013, 25.
