# Supplementary material for: Cell fate simulation reveals cancer cell features in the tumor microenvironment
Source: J Biol Chem. 2024 Aug 20;300(9):107697. doi: 10.1016/j.jbc.2024.107697 (PMC11419826; doi:10.1016/j.jbc.2024.107697)

Analysis: HeLa, Treat.: HeLa, Cell: HeLa

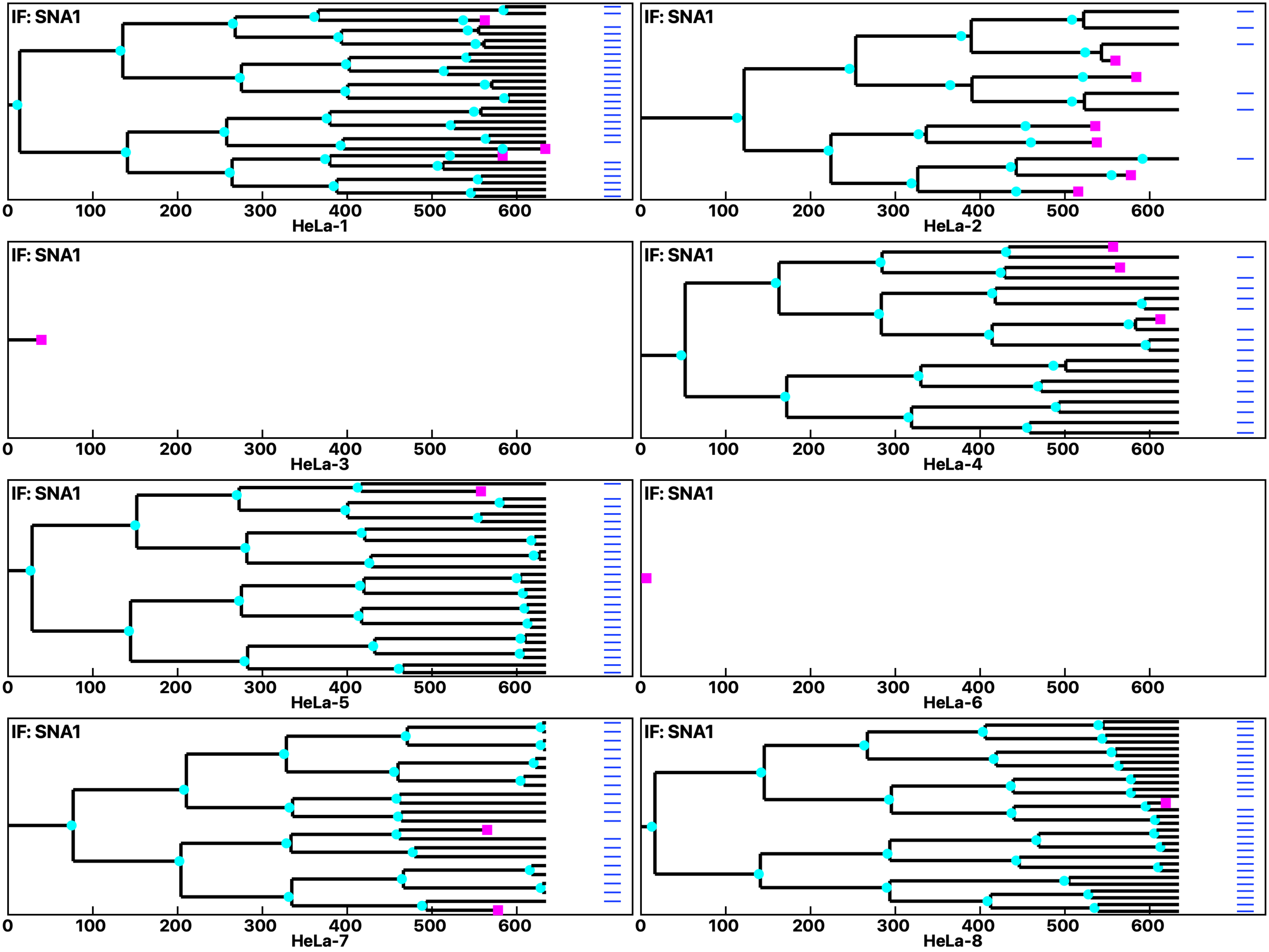

Analysis: HeLa, Treat.: HeLa, Cell: HeLa

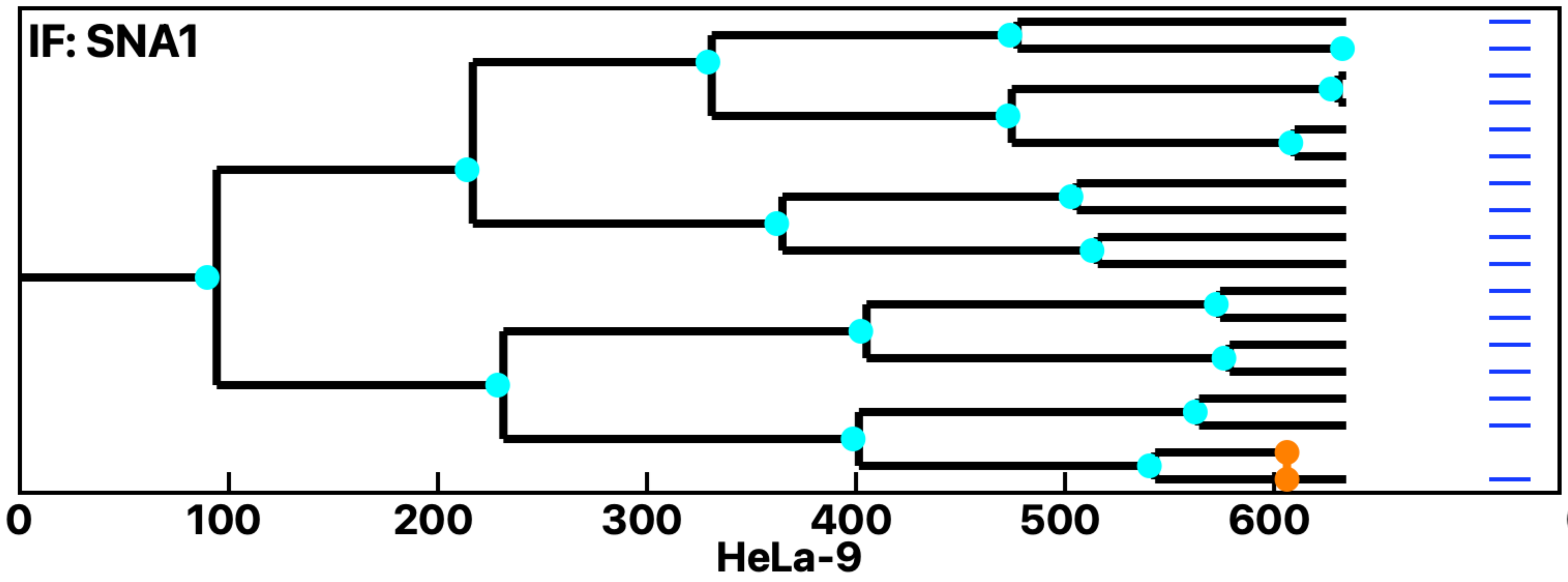

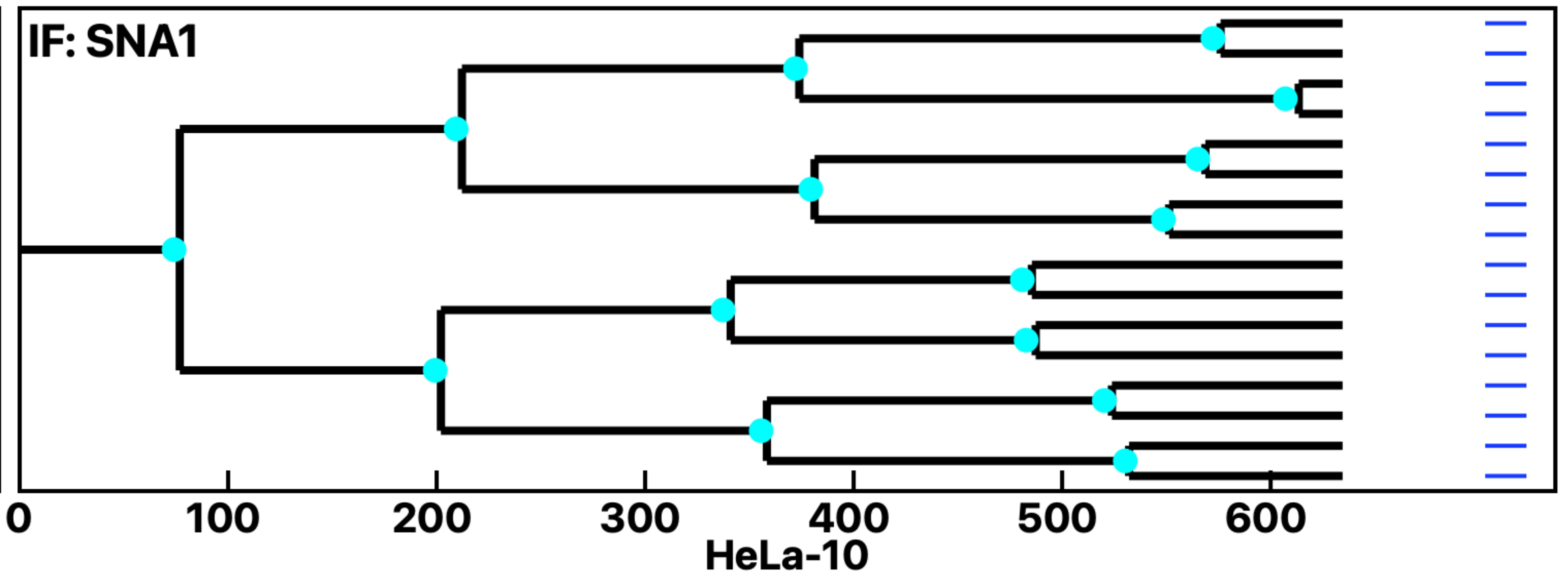

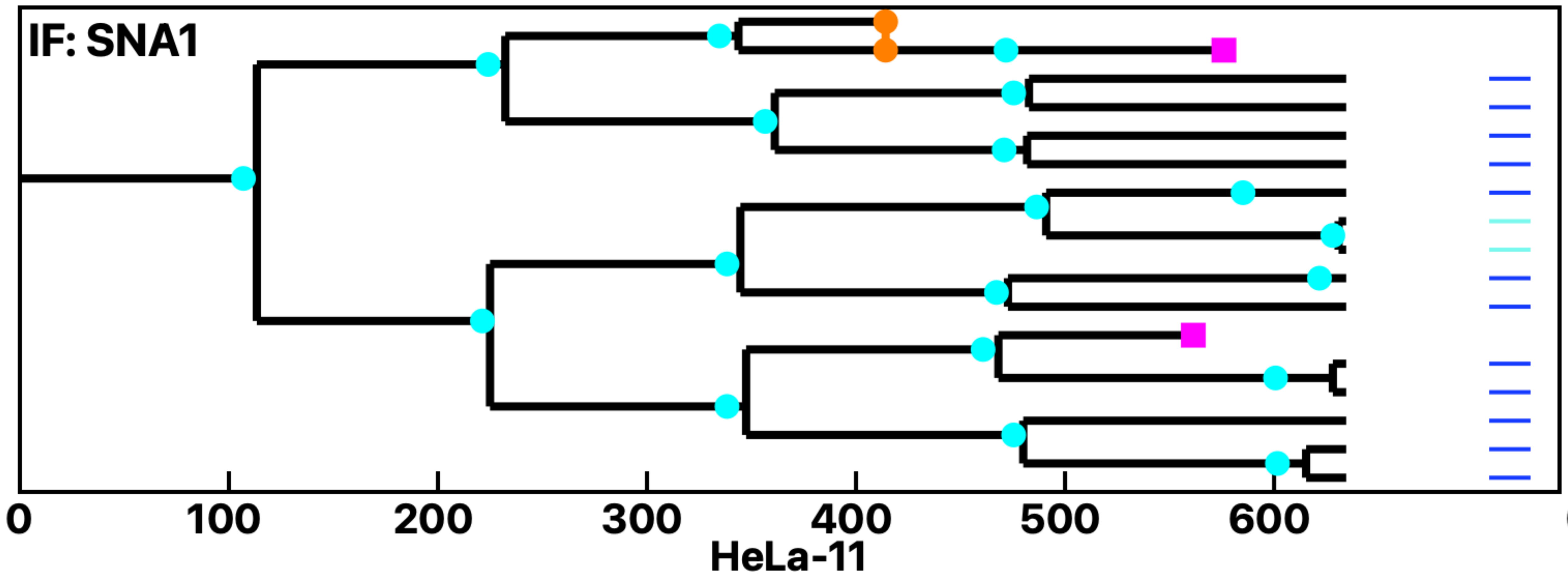

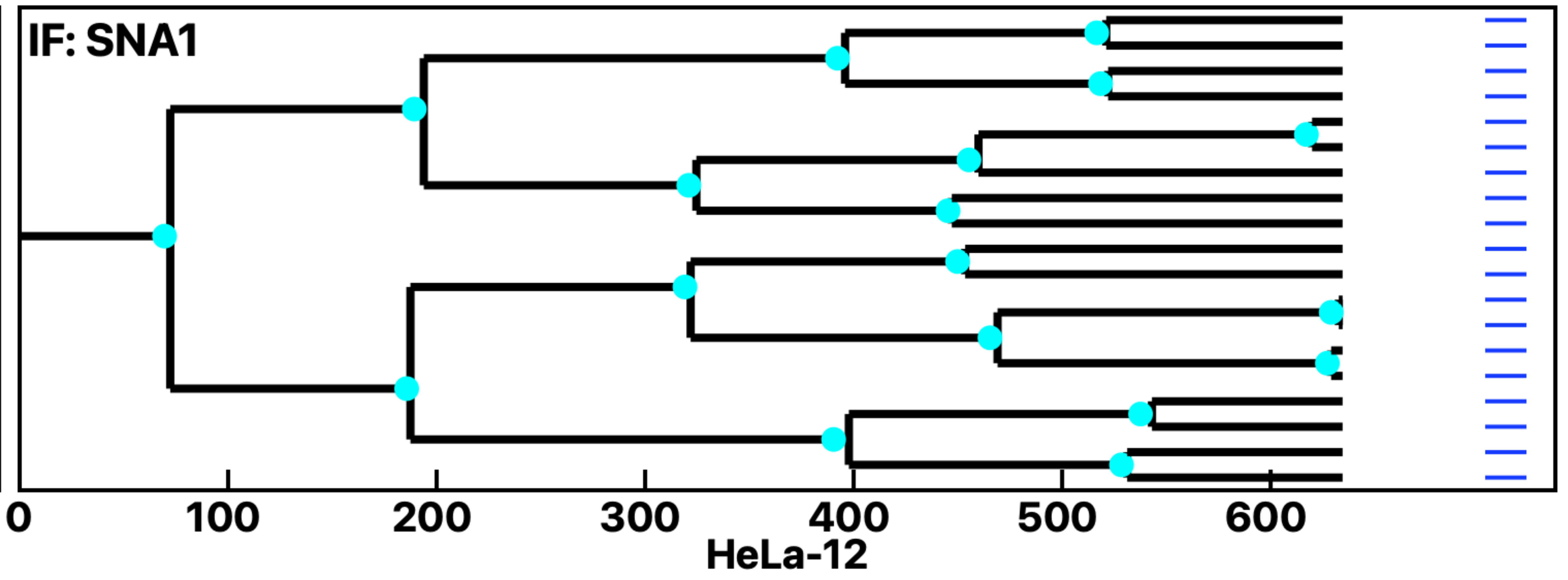

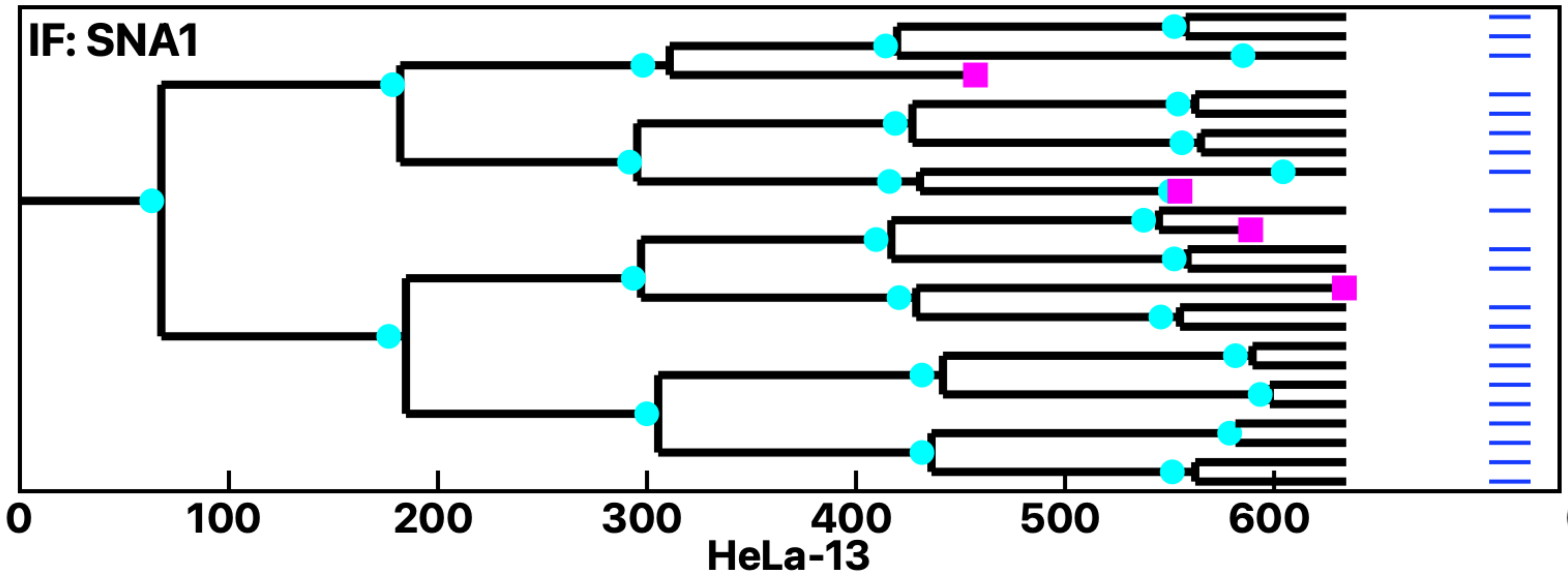

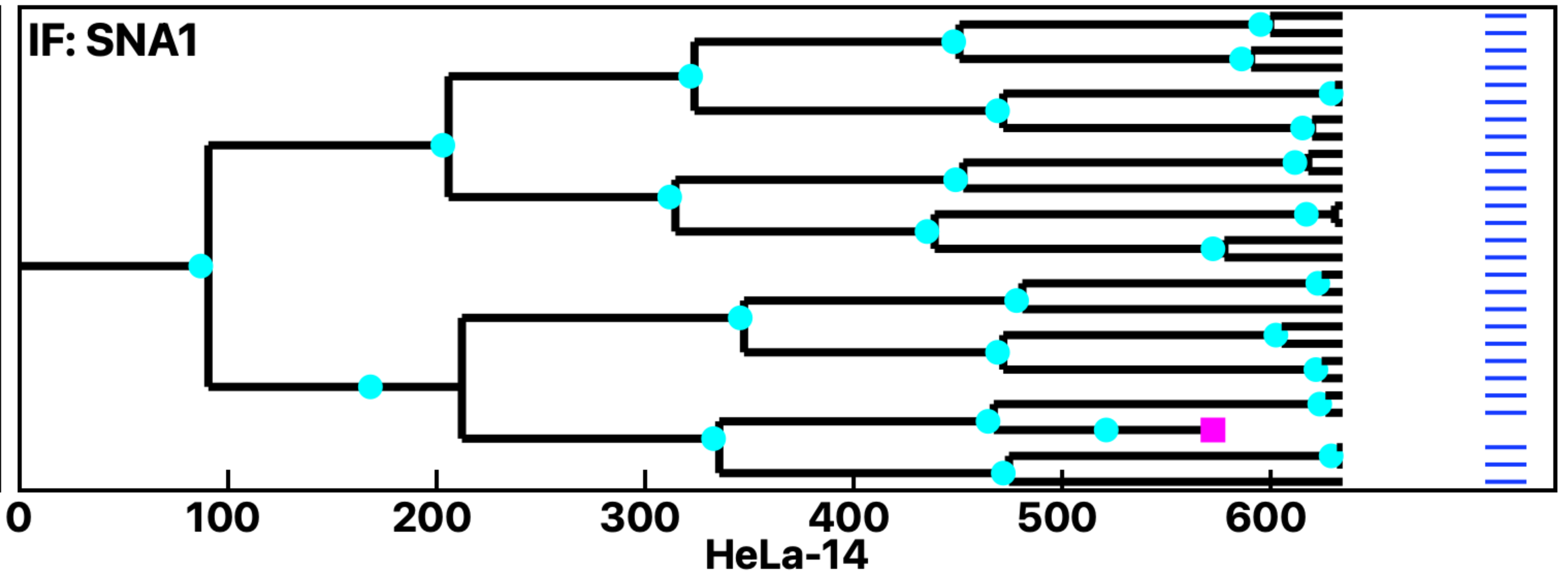

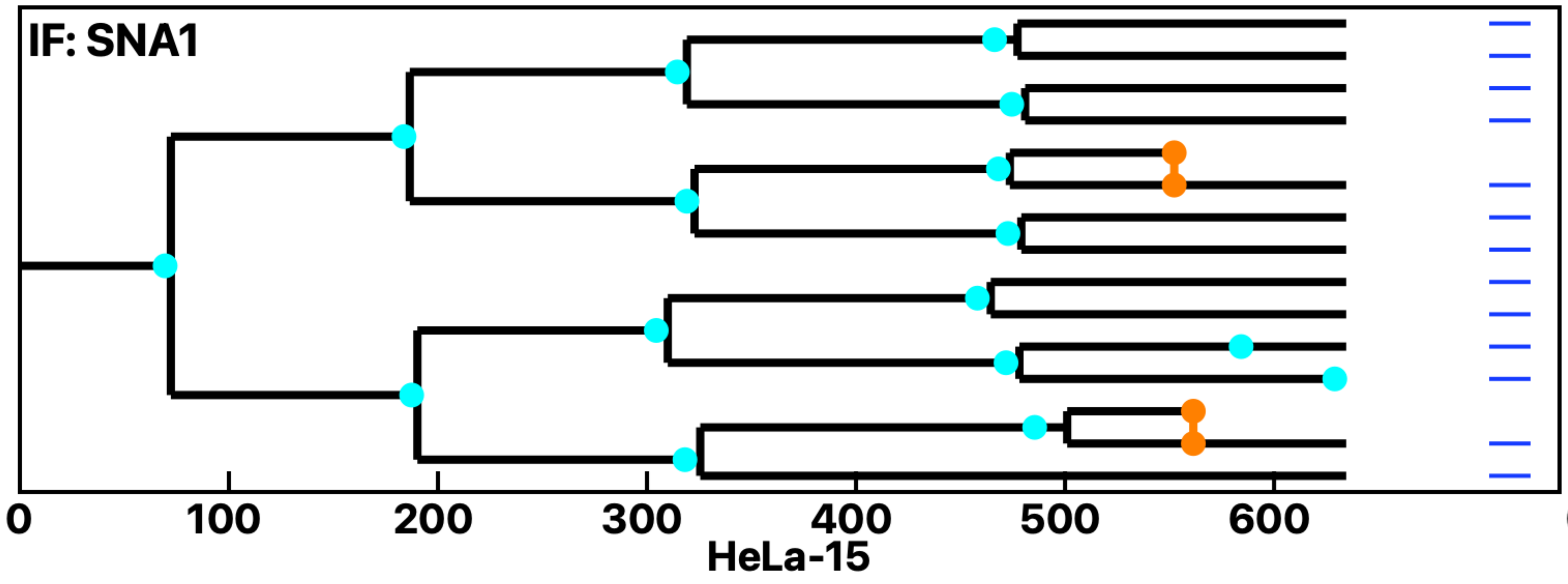

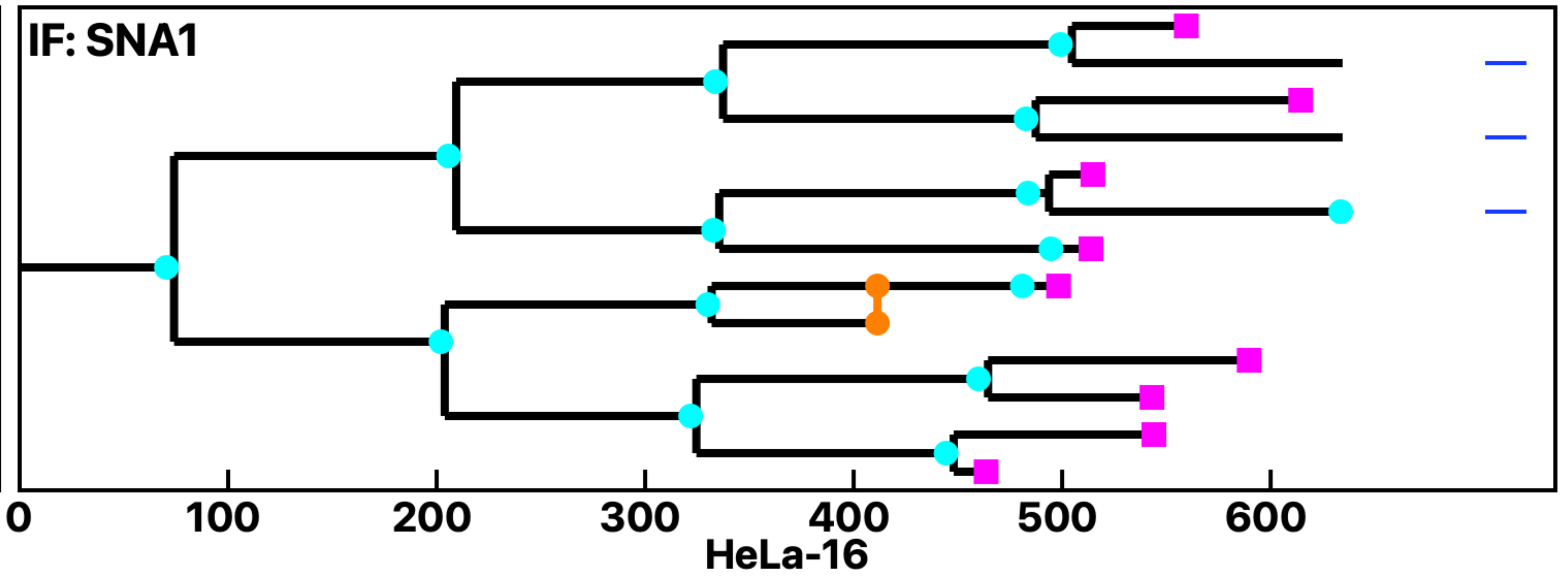

Analysis: HeLa, Treat.: HeLa, Cell: HeLa

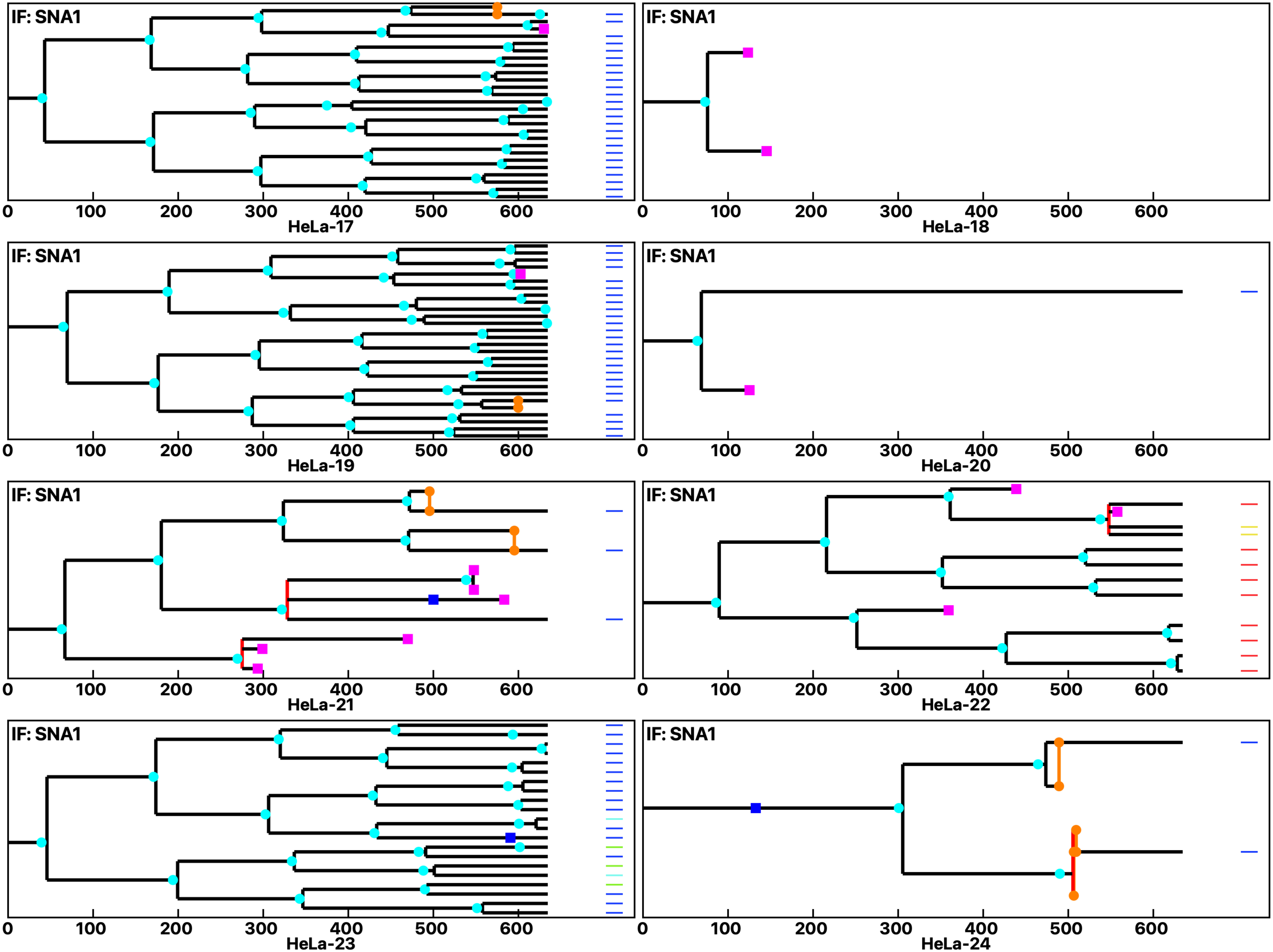

Analysis: HeLa, Treat.: HeLa, Cell: HeLa

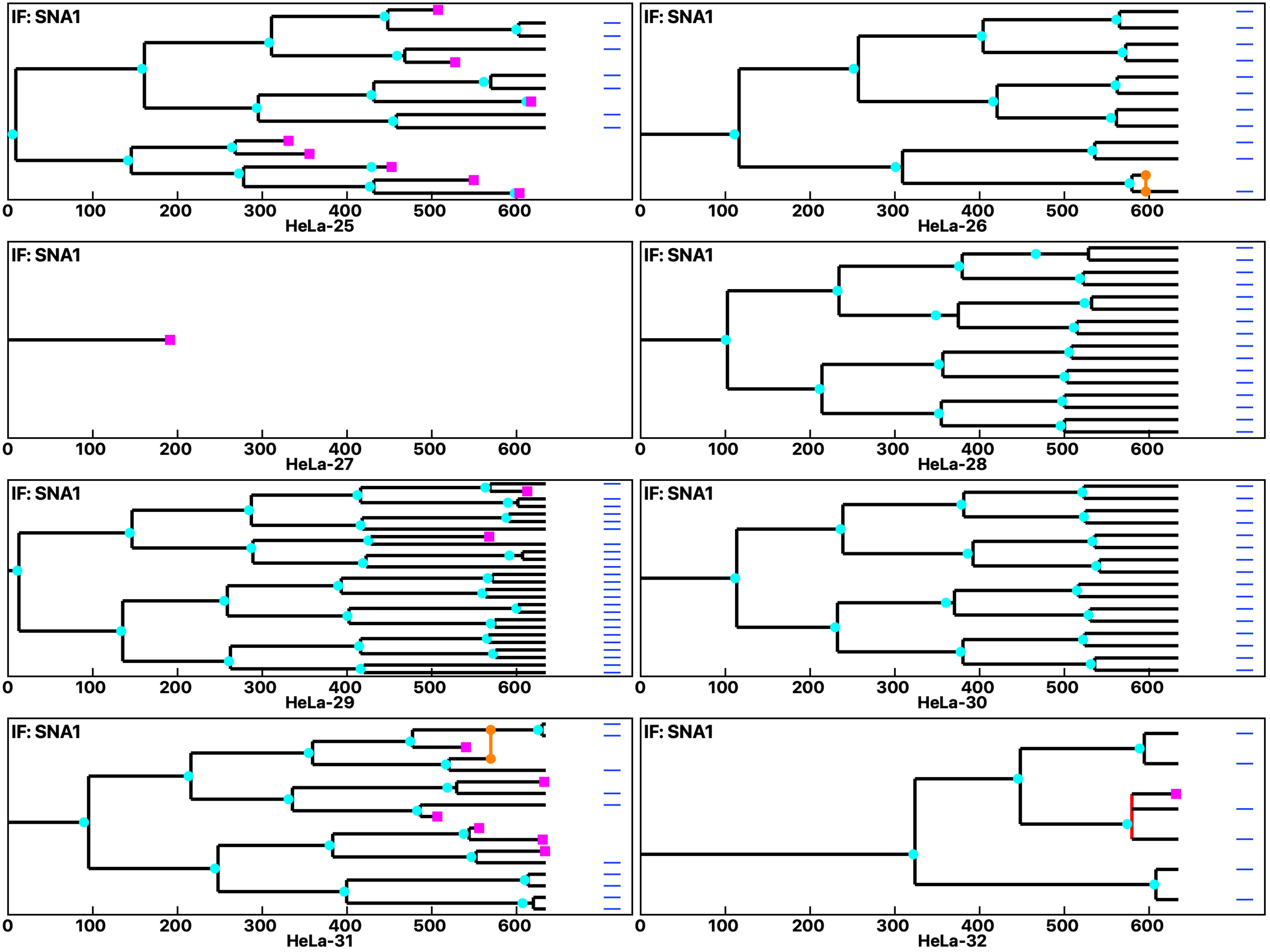

Analysis: HeLa, Treat.: HeLa, Cell: HeLa

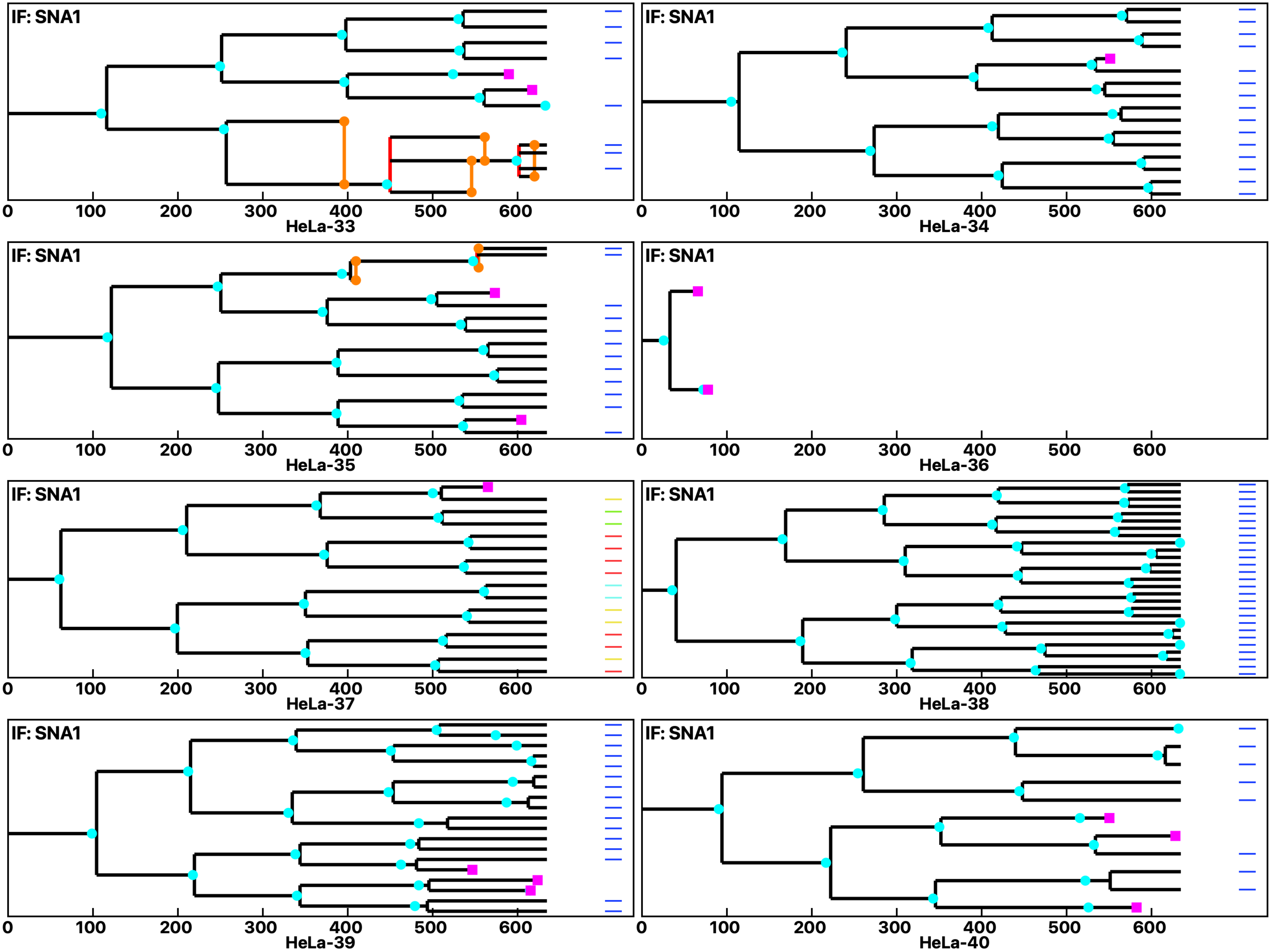

Analysis: HeLa, Treat.: HeLa, Cell: HeLa

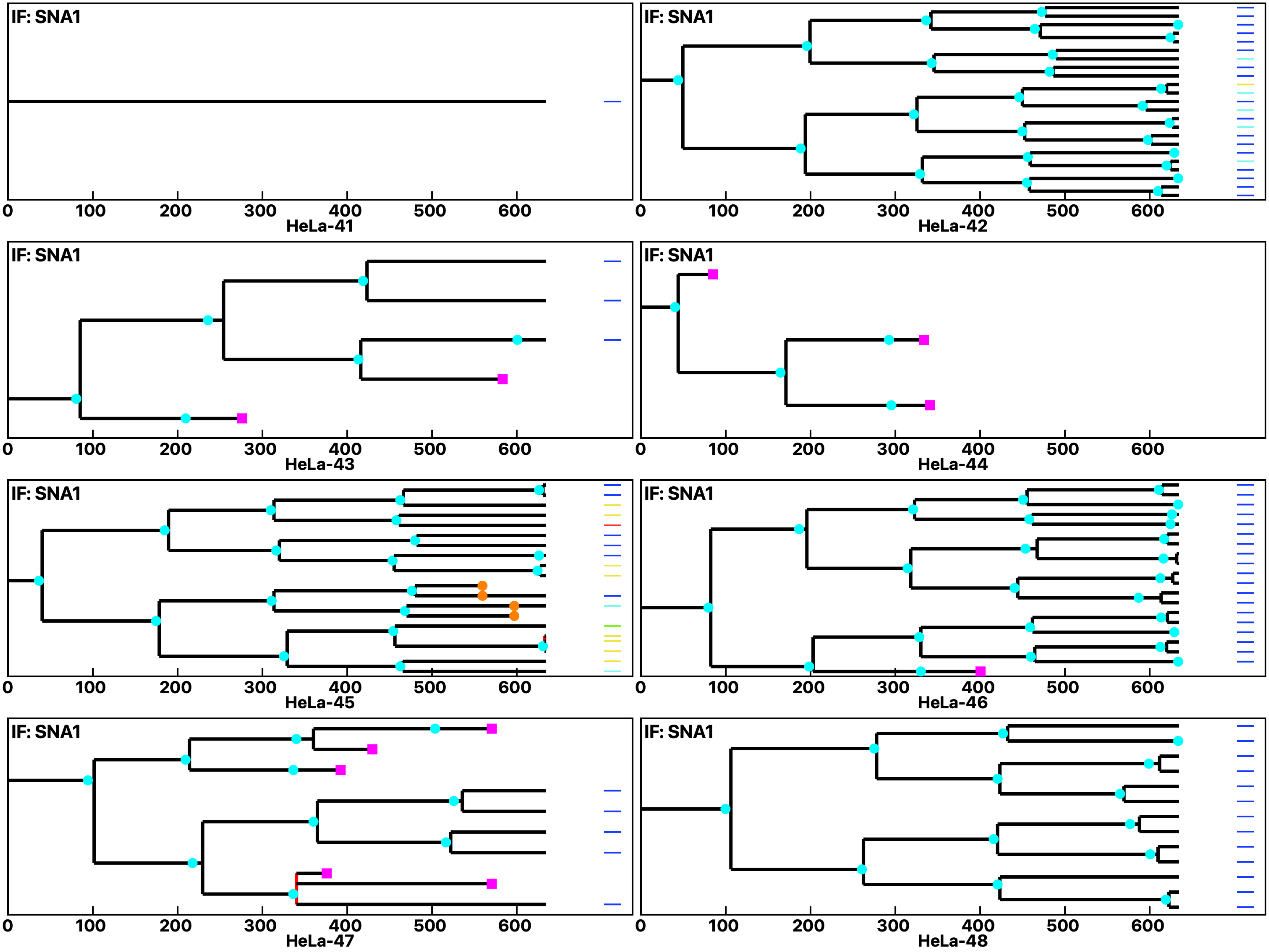

Analysis: HeLa, Treat.: HeLa, Cell: HeLa

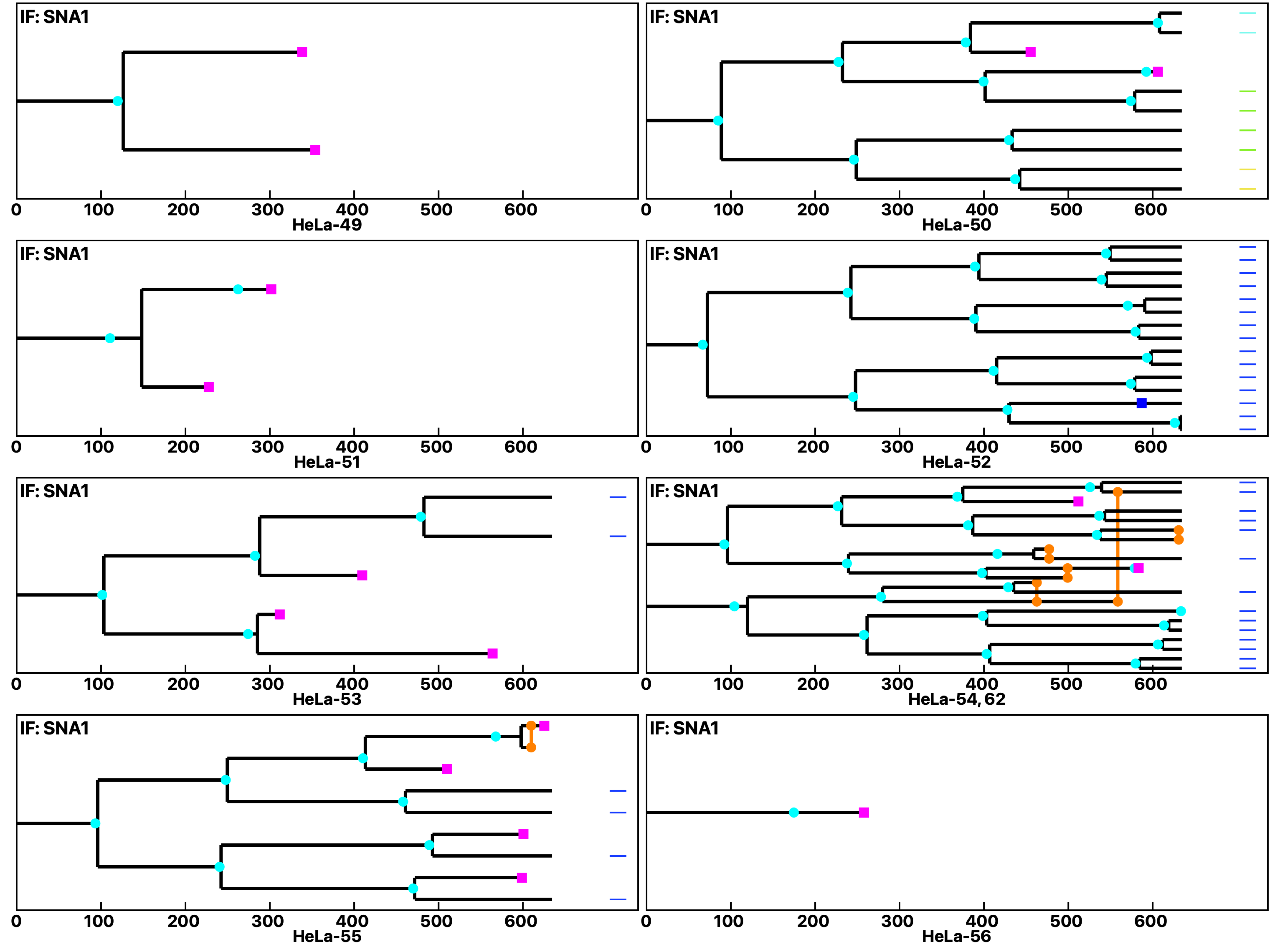

Analysis: HeLa, Treat.: HeLa, Cell: HeLa

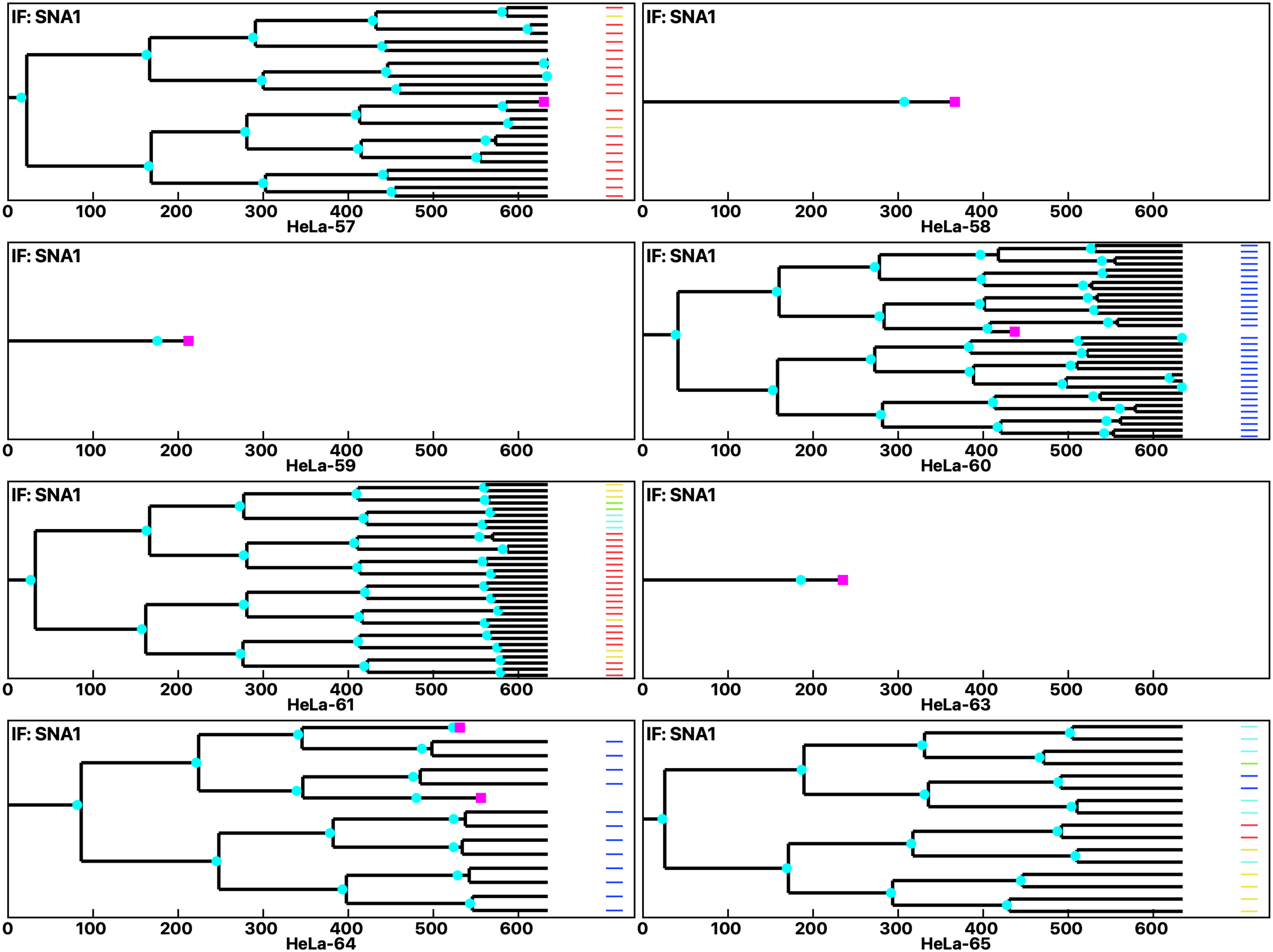

Analysis: HeLa, Treat.: HeLa, Cell: HeLa

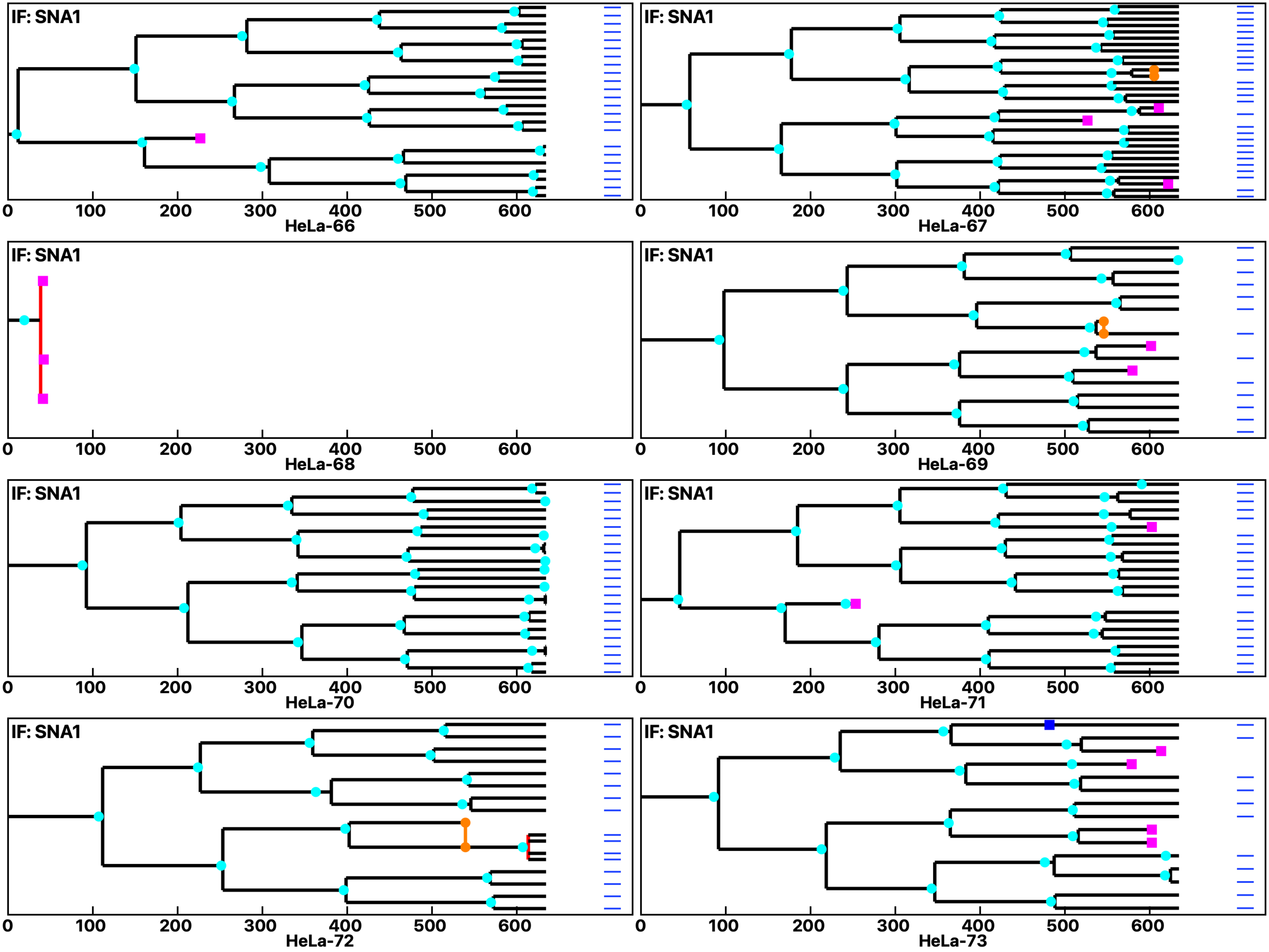

Analysis: HeLa, Treat.: HeLa, Cell: HeLa

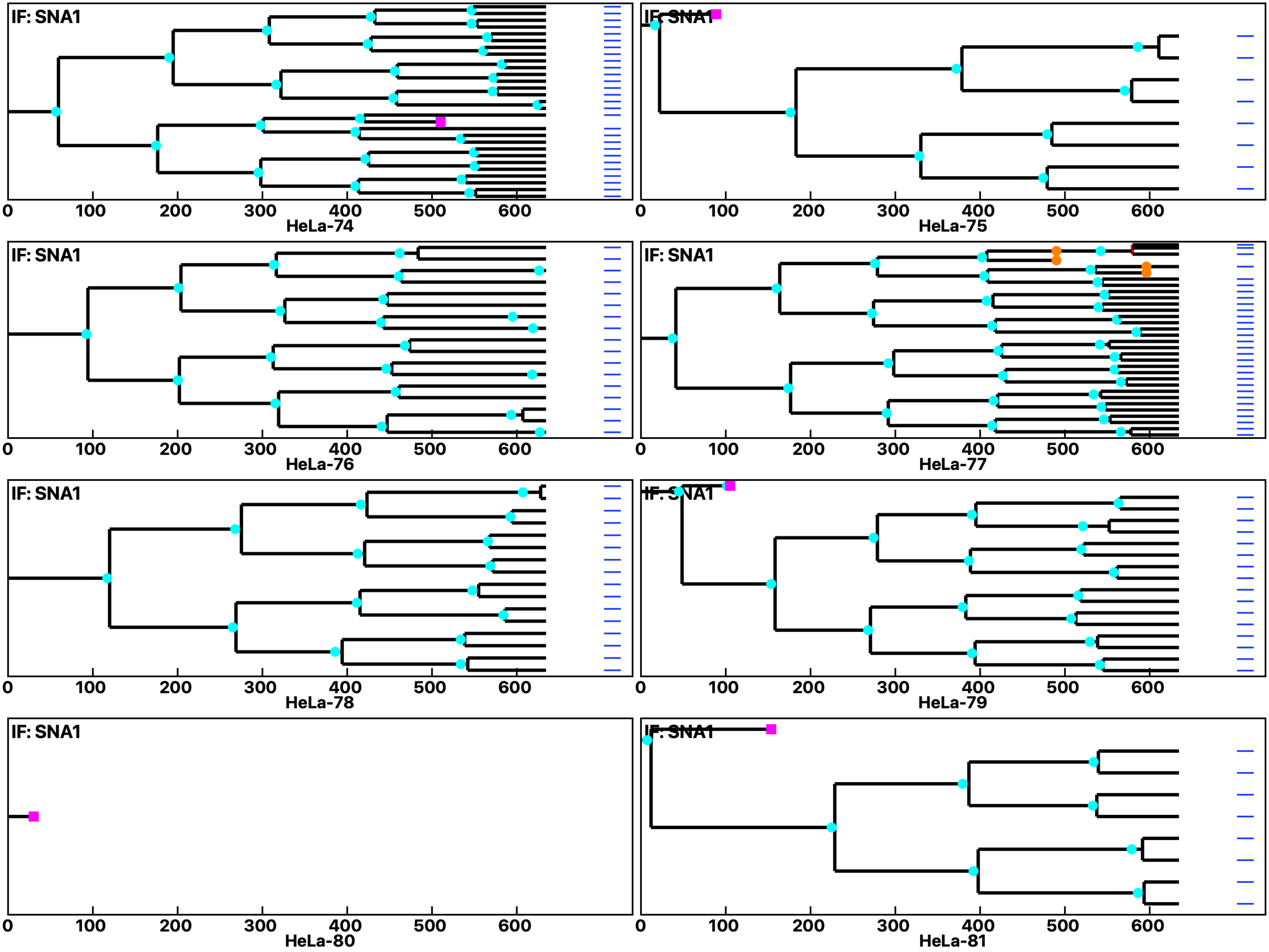

Analysis: HeLa, Treat.: HeLa, Cell: HeLa

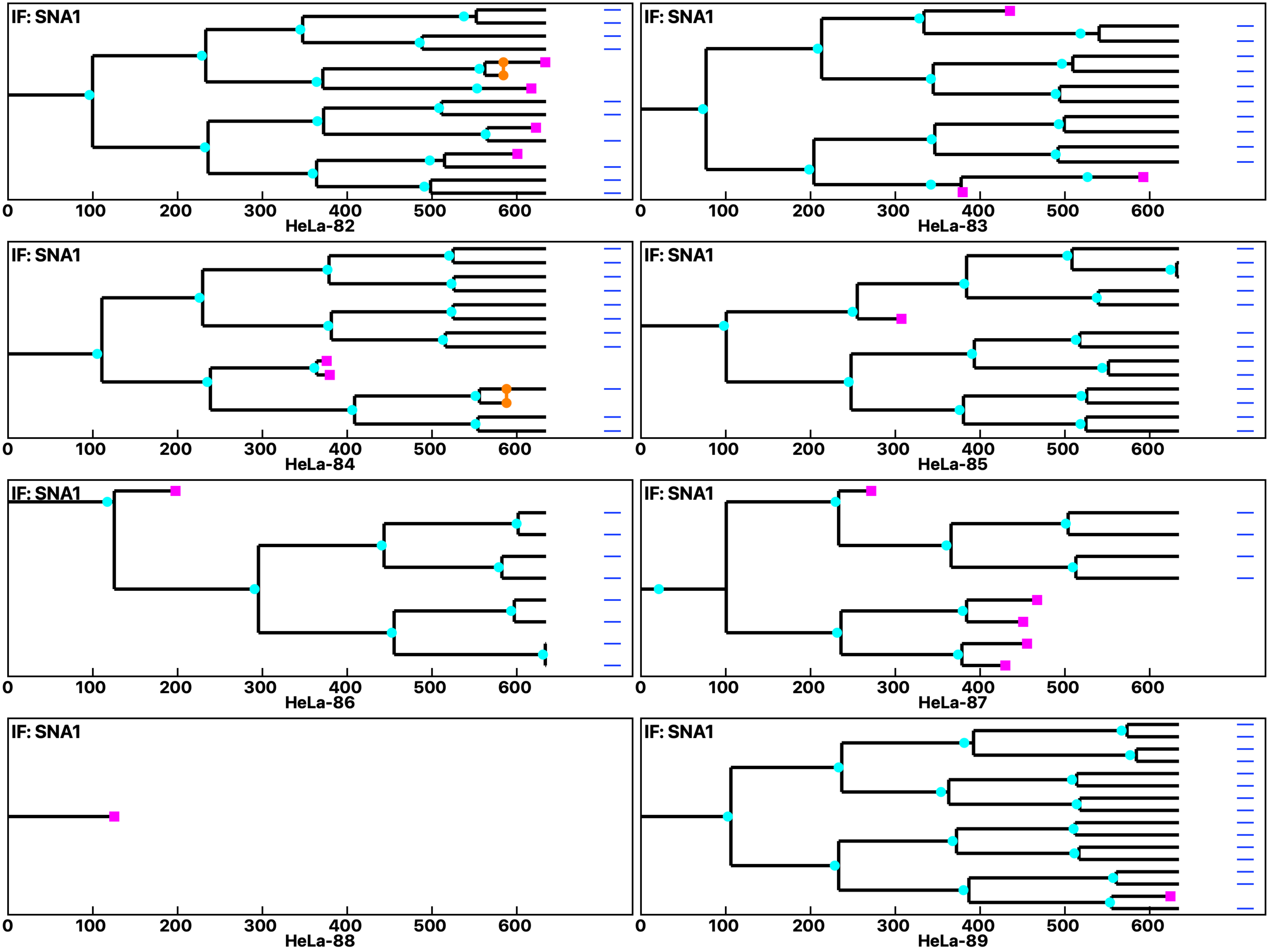

Analysis: HeLa, Treat.: HeLa, Cell: HeLa

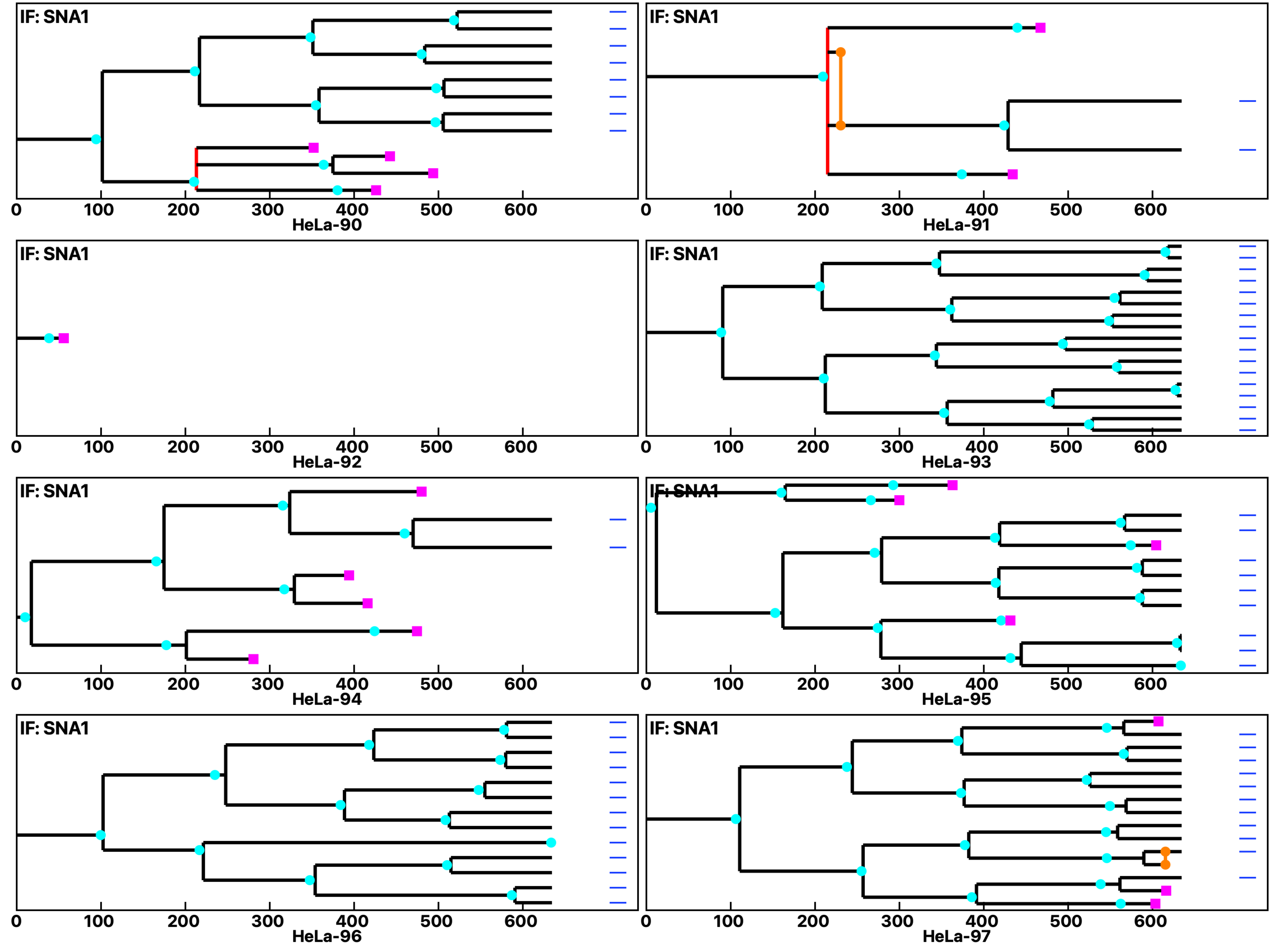

Analysis: HeLa, Treat.: HeLa, Cell: HeLa

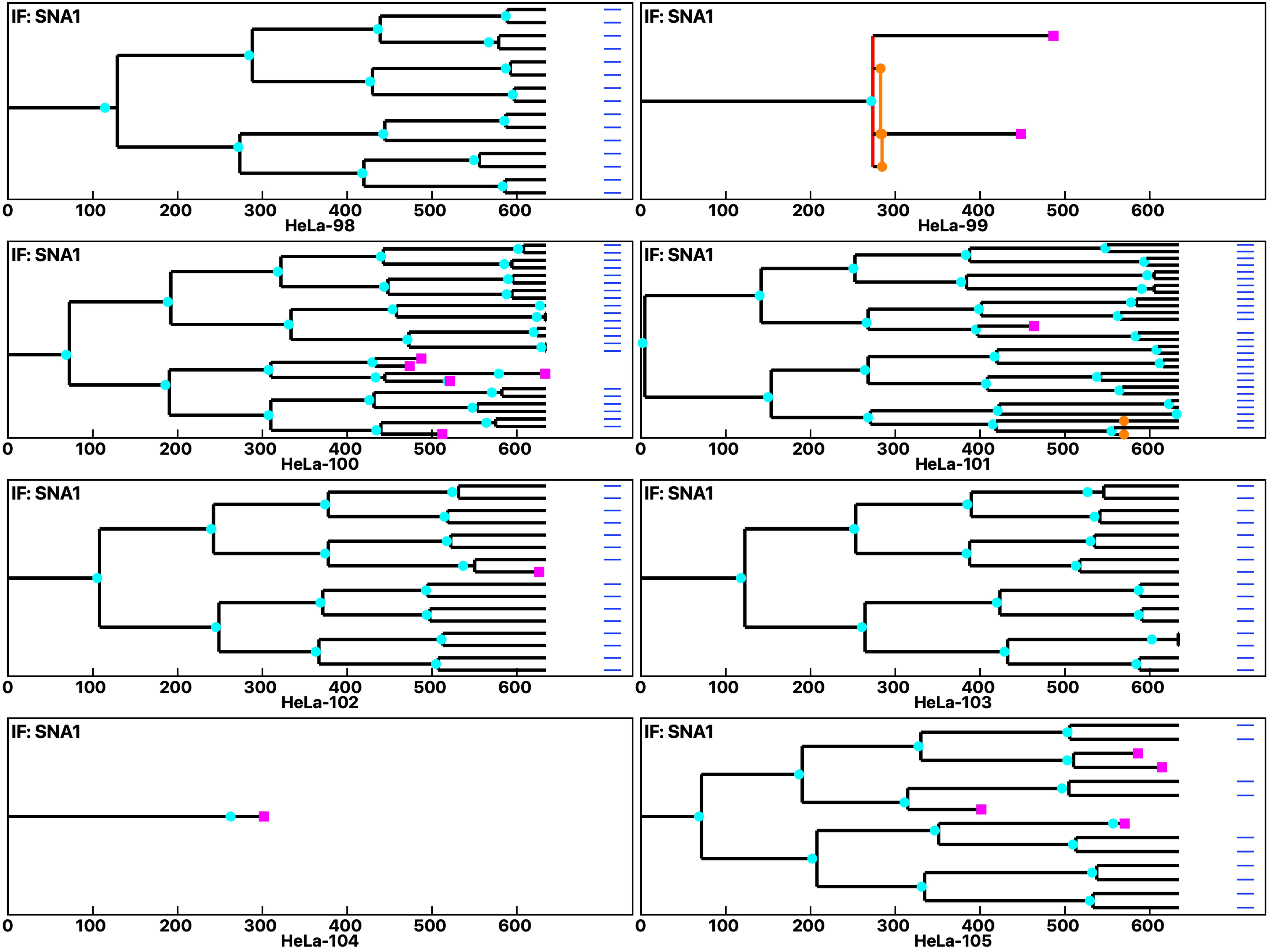

Analysis: HeLa, Treat.: HeLa, Cell: HeLa

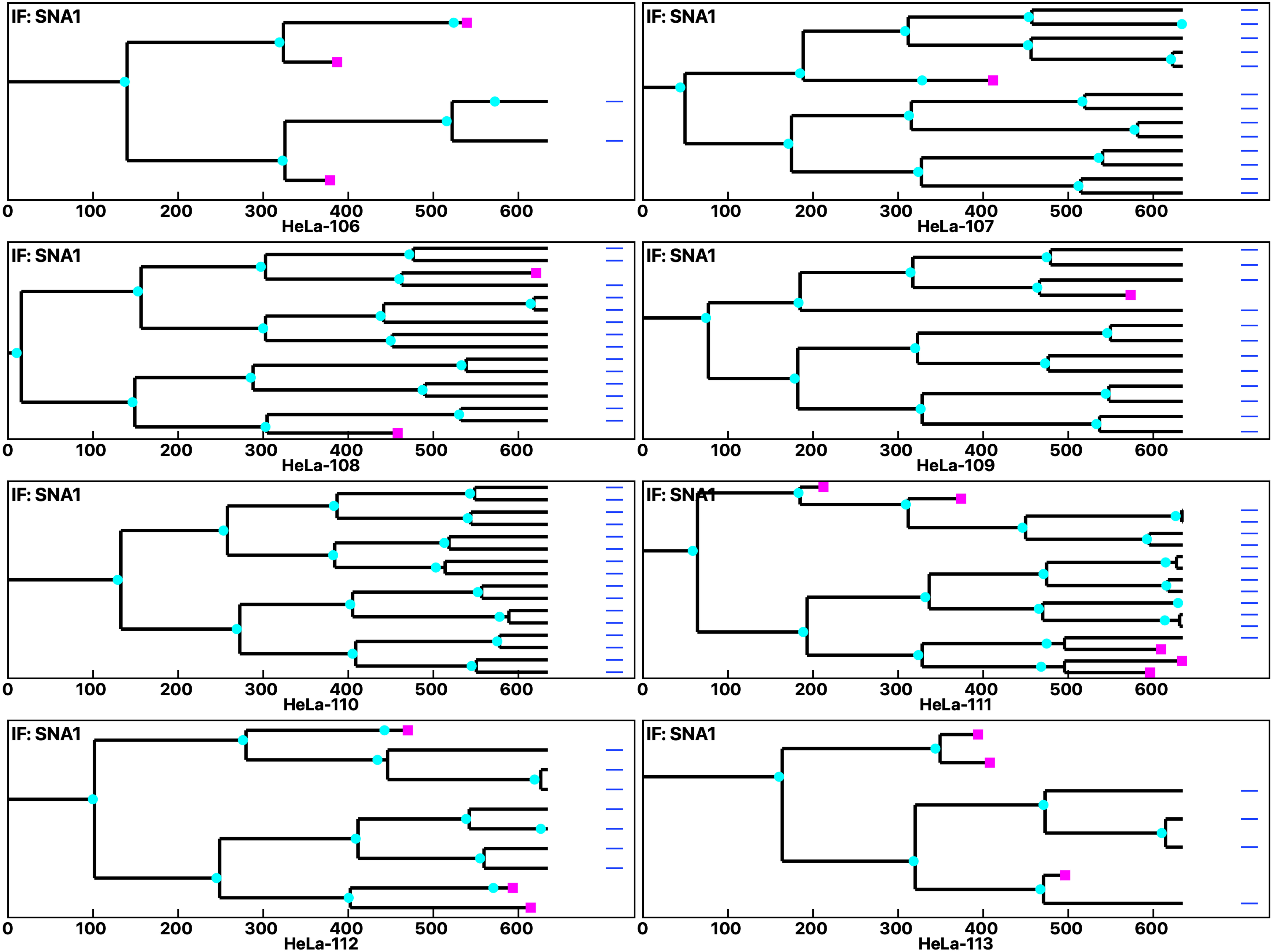

Analysis: HeLa, Treat.: HeLa, Cell: HeLa

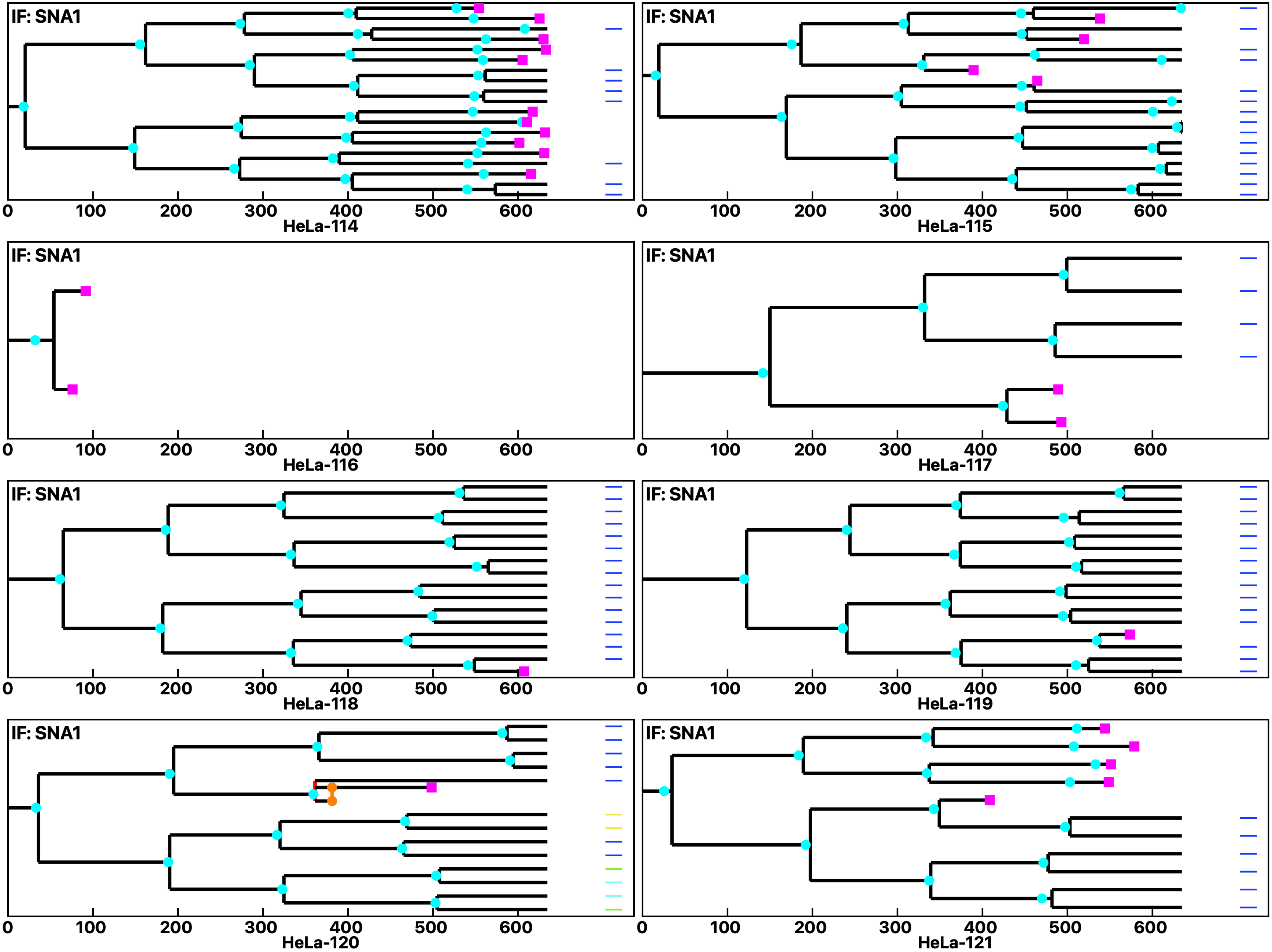

Analysis: HeLa, Treat.: HeLa, Cell: HeLa

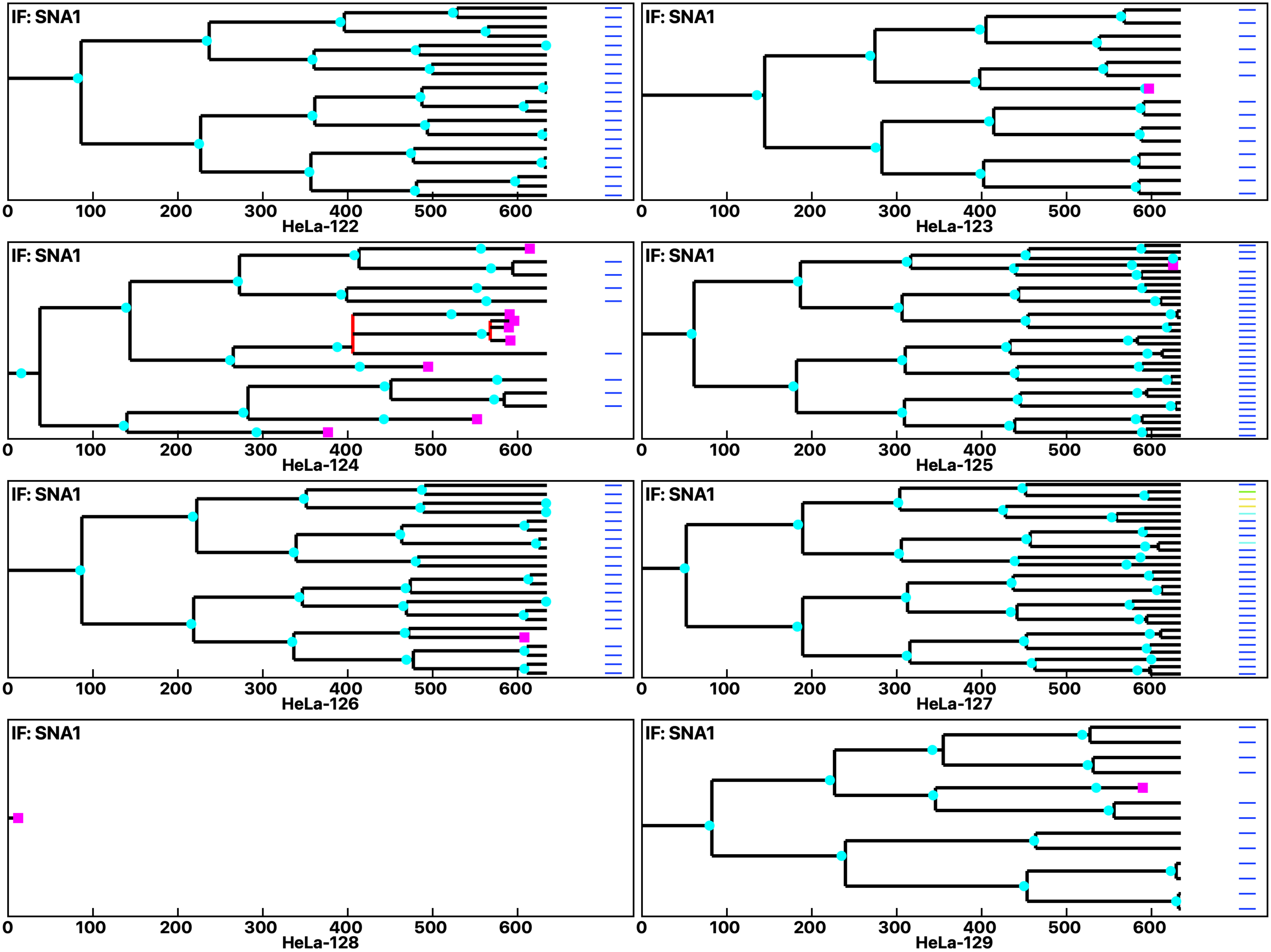

Analysis: HeLa, Treat.: HeLa, Cell: HeLa

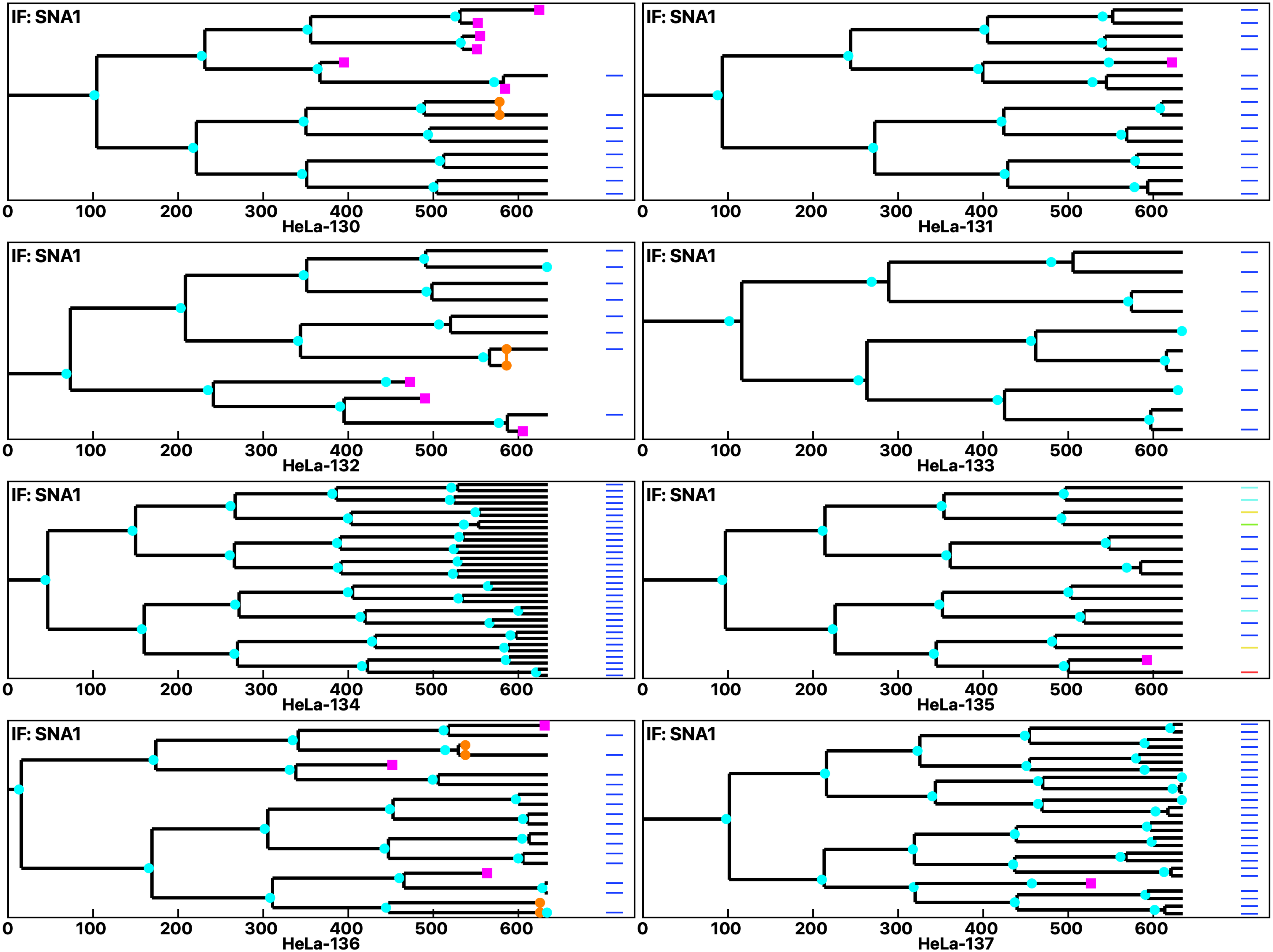

Analysis: HeLa, Treat.: HeLa, Cell: HeLa

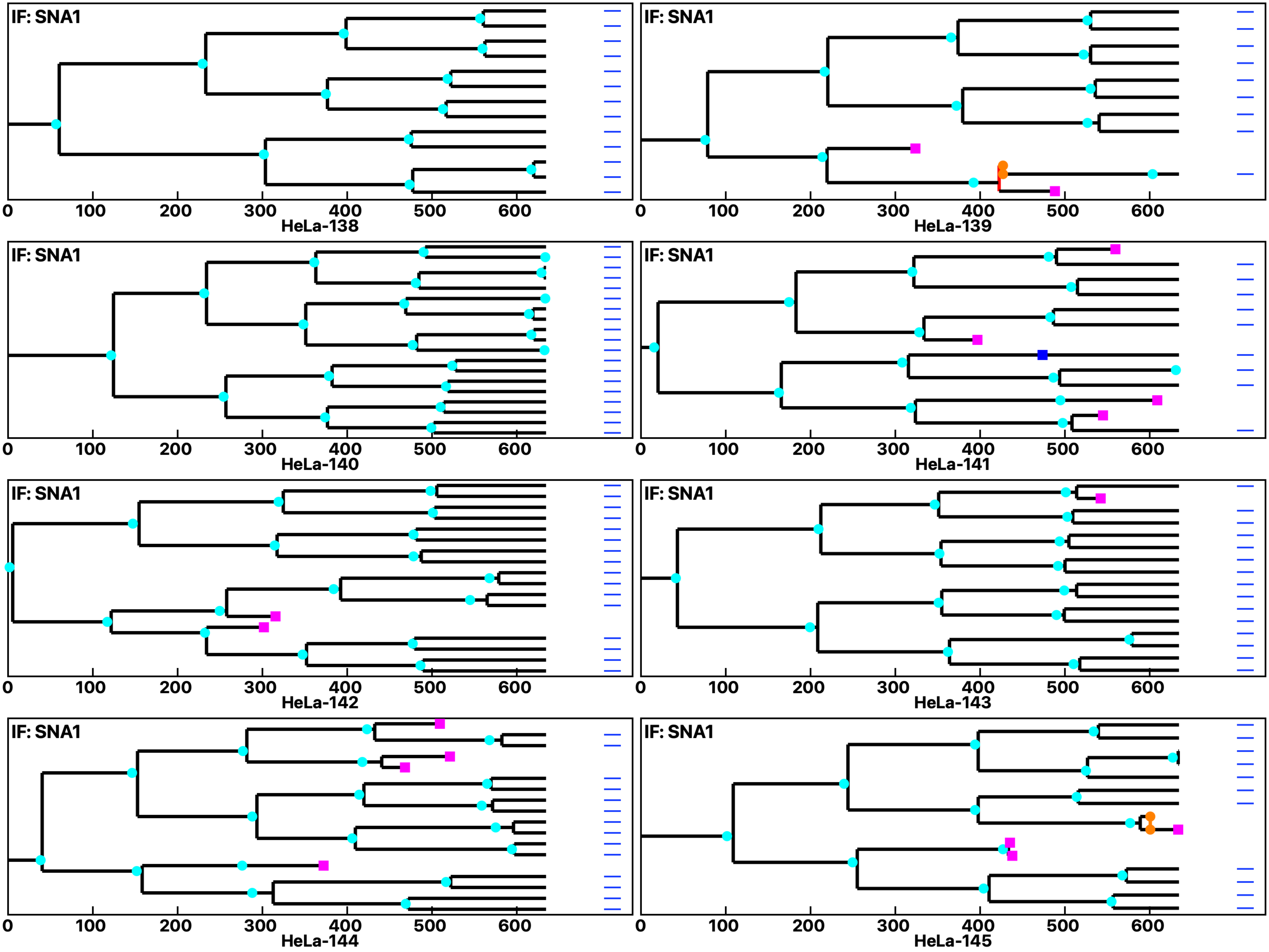

Analysis: HeLa, Treat.: HeLa, Cell: HeLa

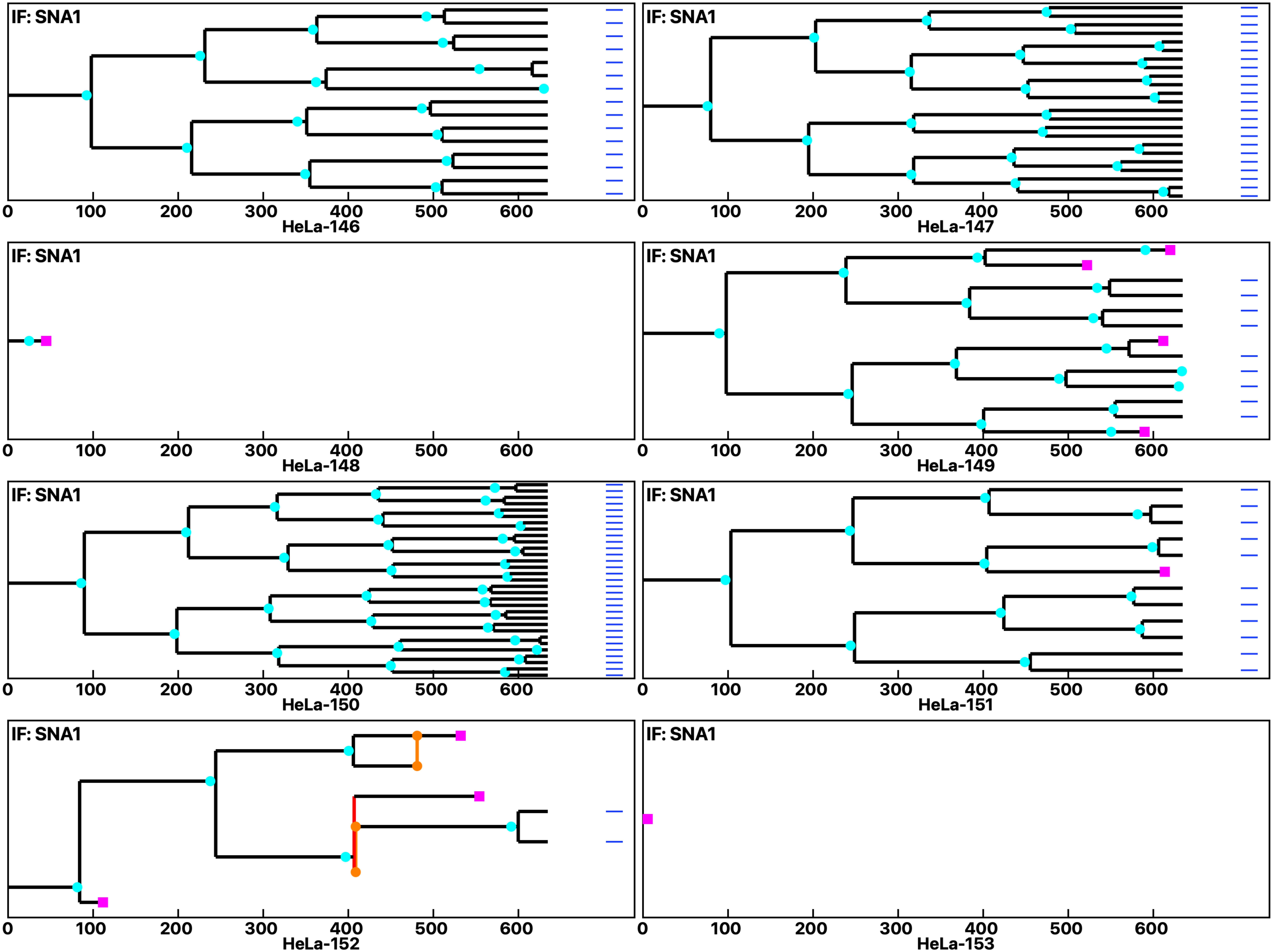

Analysis: HeLa, Treat.: HeLa, Cell: HeLa

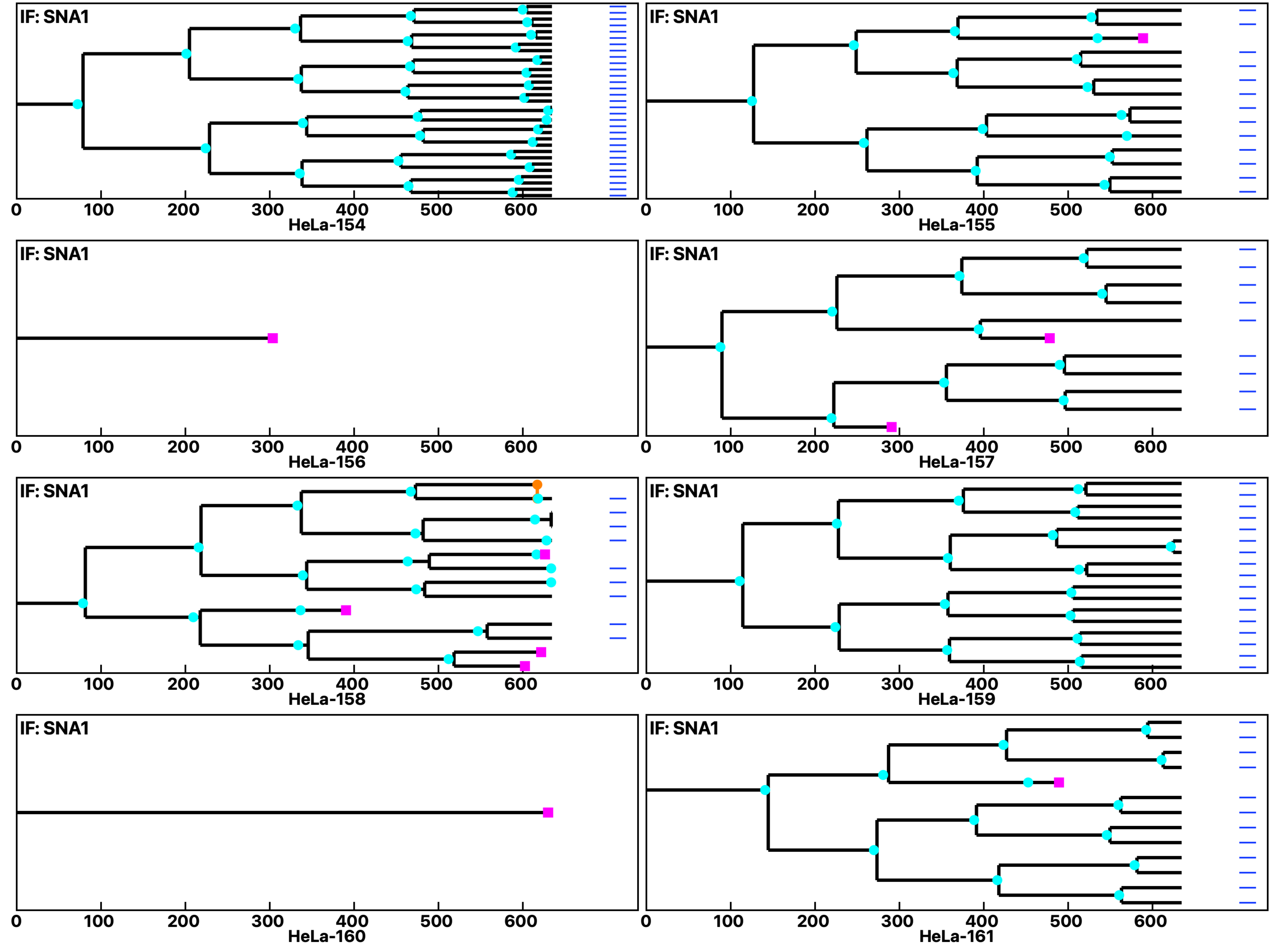

Analysis: HeLa, Treat.: HeLa, Cell: HeLa

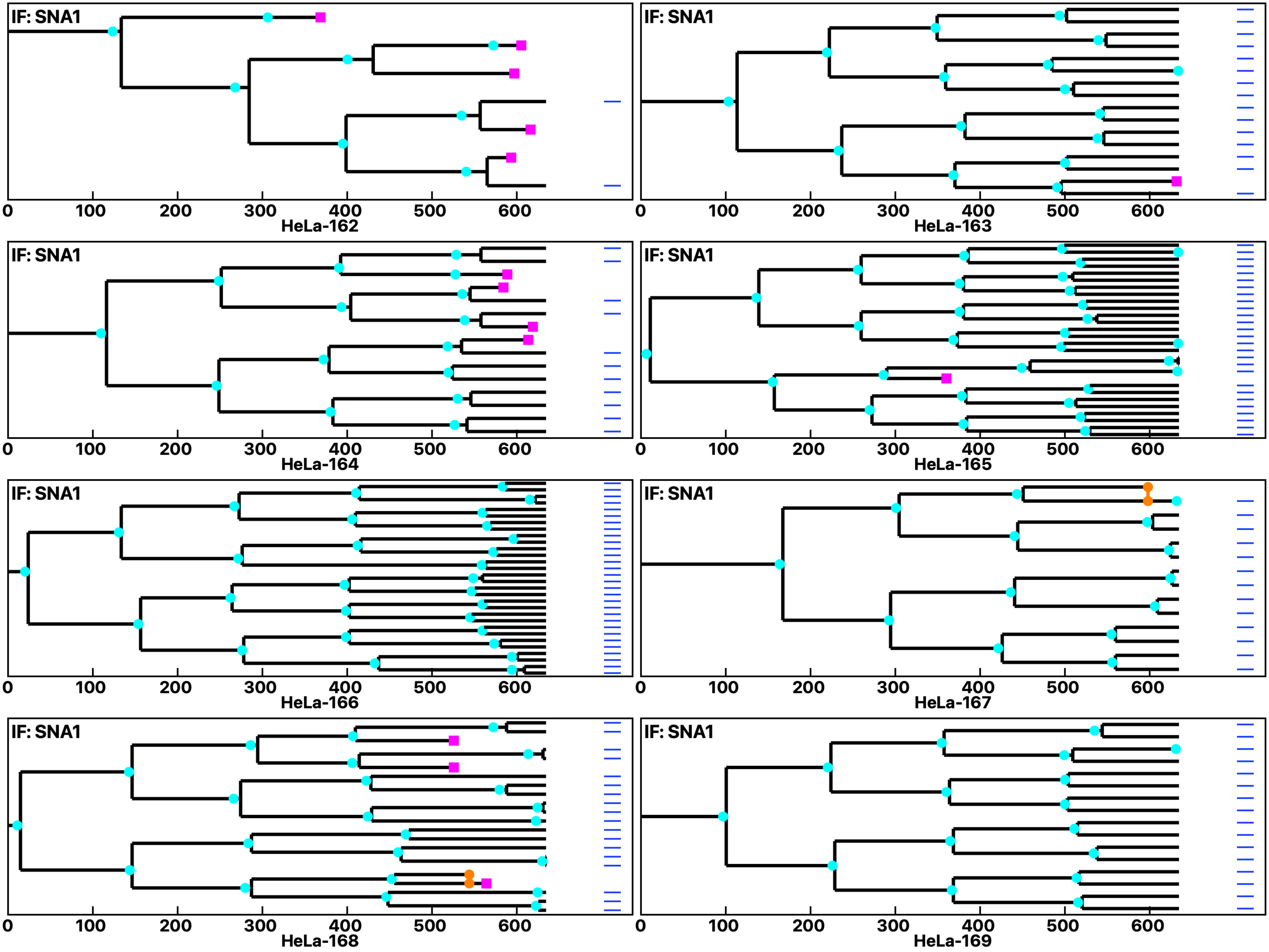

Analysis: HeLa, Treat.: HeLa, Cell: HeLa

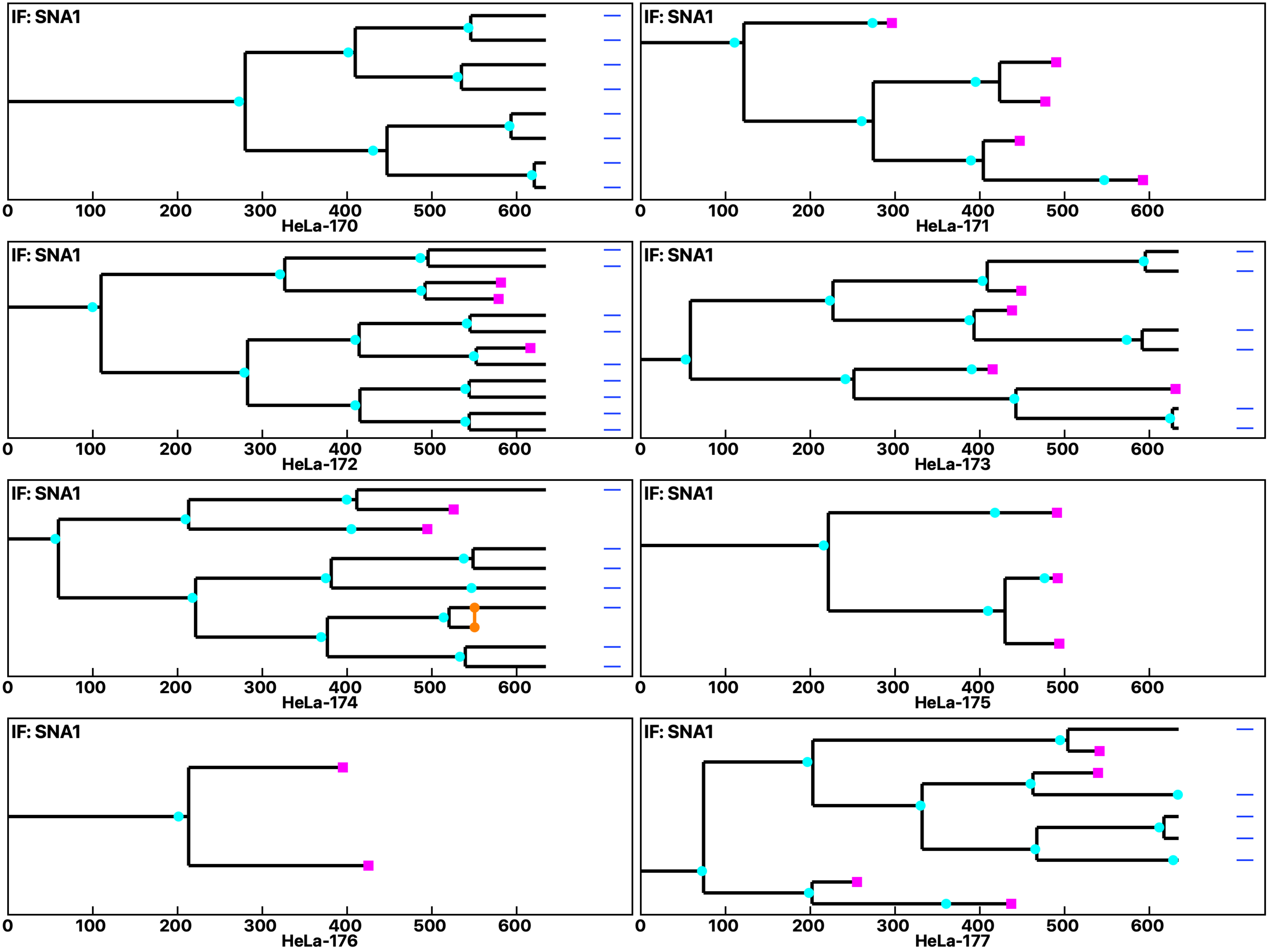

Analysis: HeLa, Treat.: HeLa, Cell: HeLa

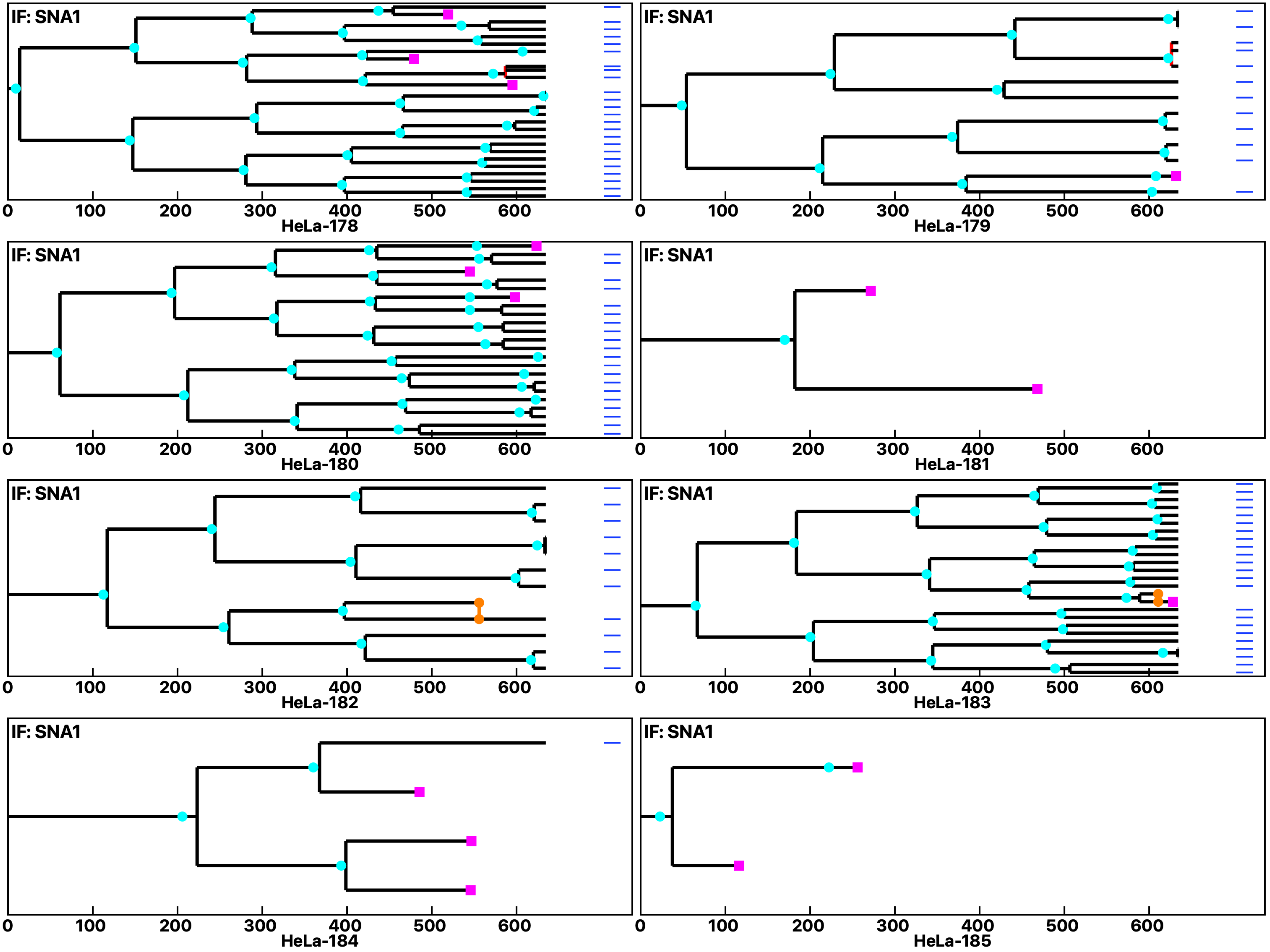

Analysis: HeLa, Treat.: HeLa, Cell: HeLa

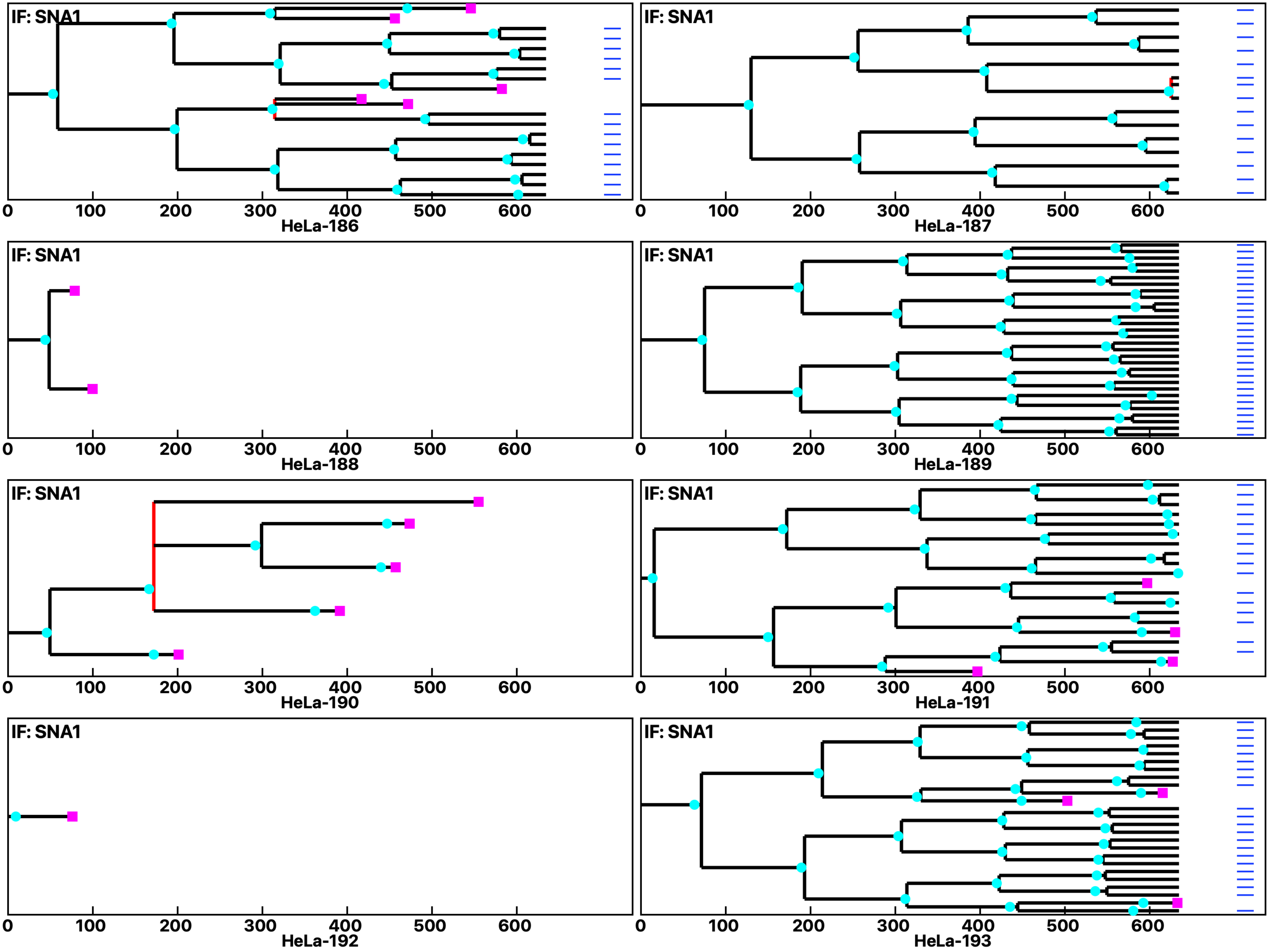

Analysis: HeLa, Treat.: HeLa, Cell: HeLa

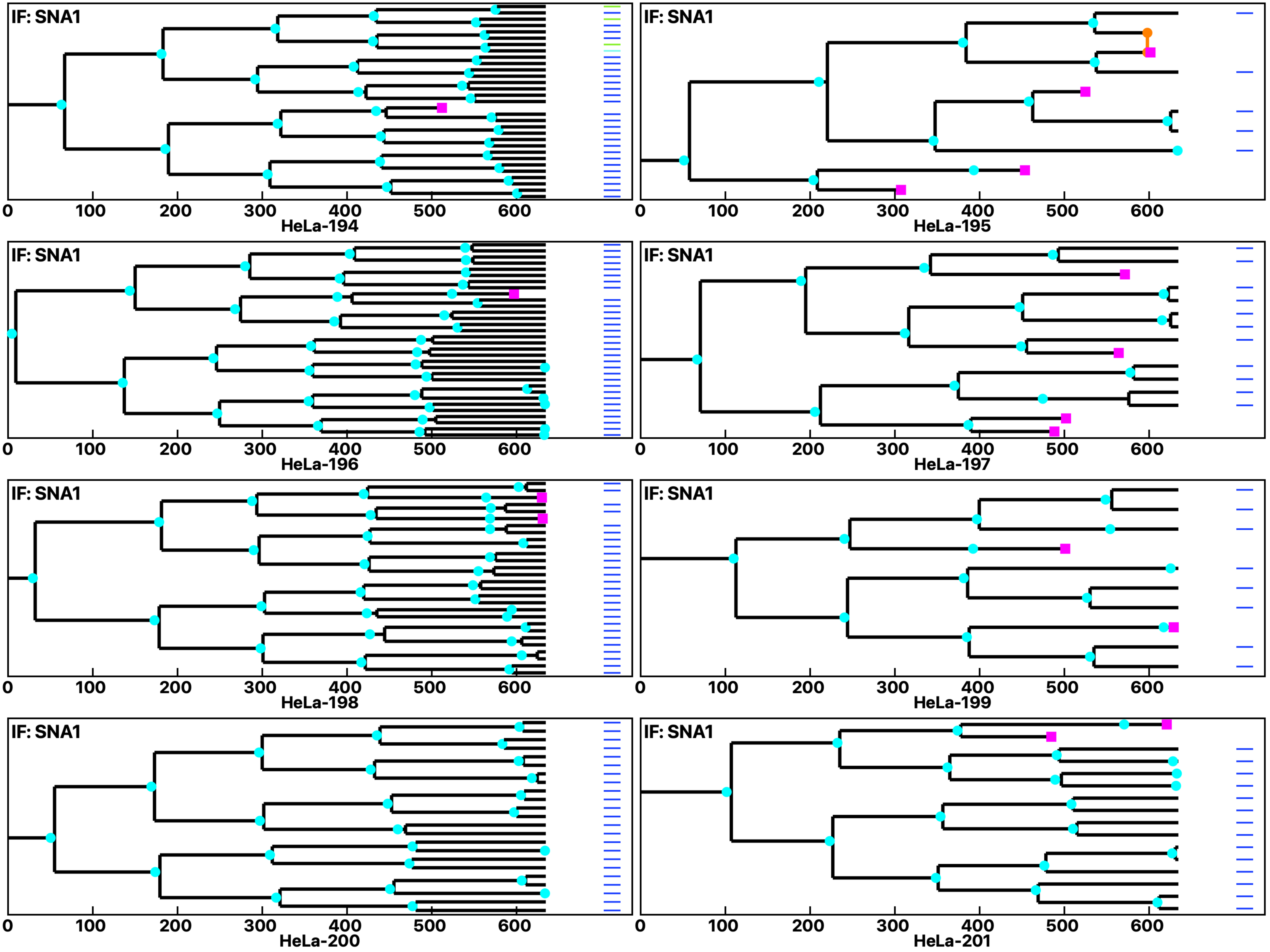

Analysis: HeLa, Treat.: HeLa, Cell: HeLa

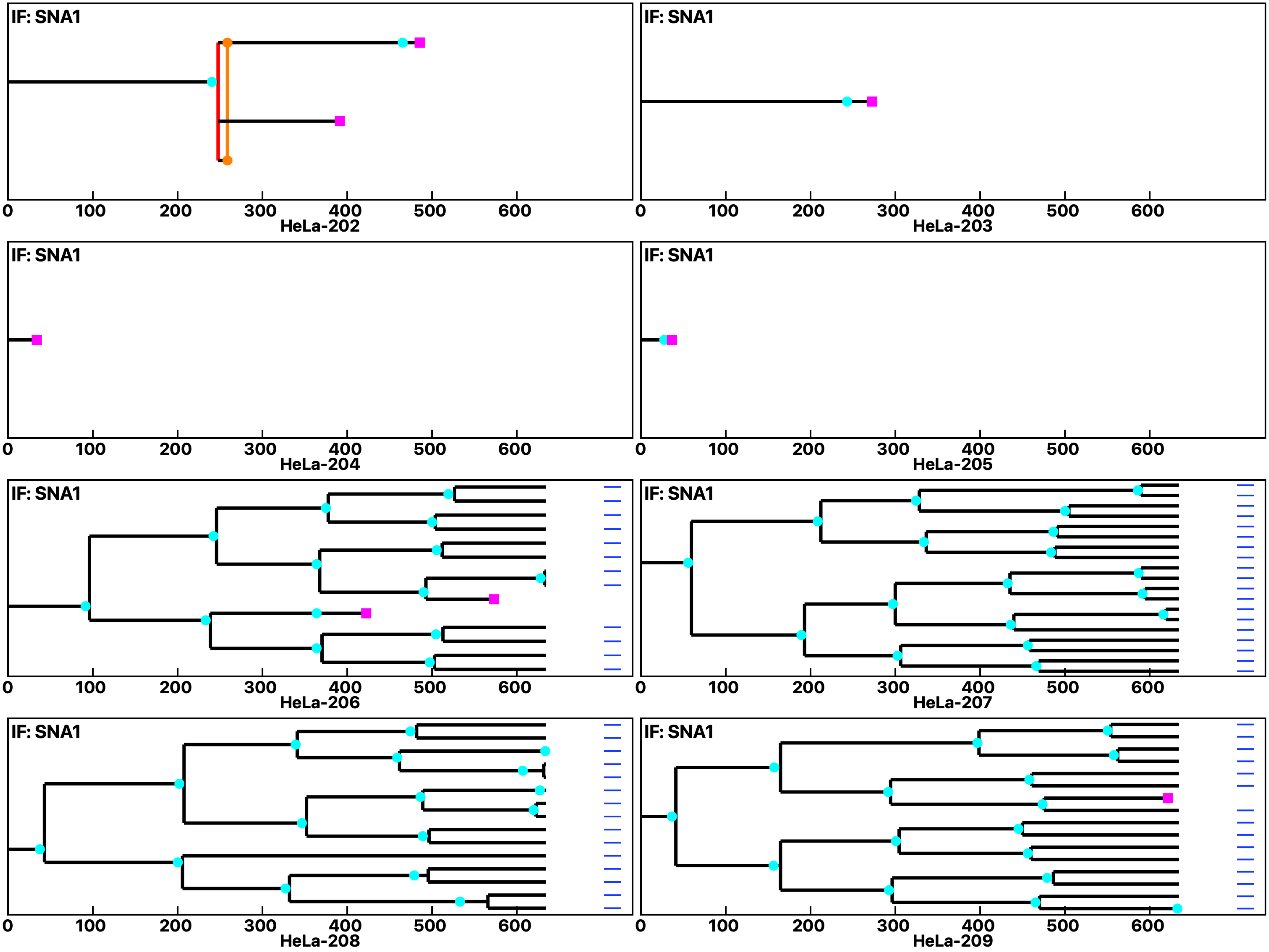

Analysis: HeLa, Treat.: HeLa, Cell: HeLa

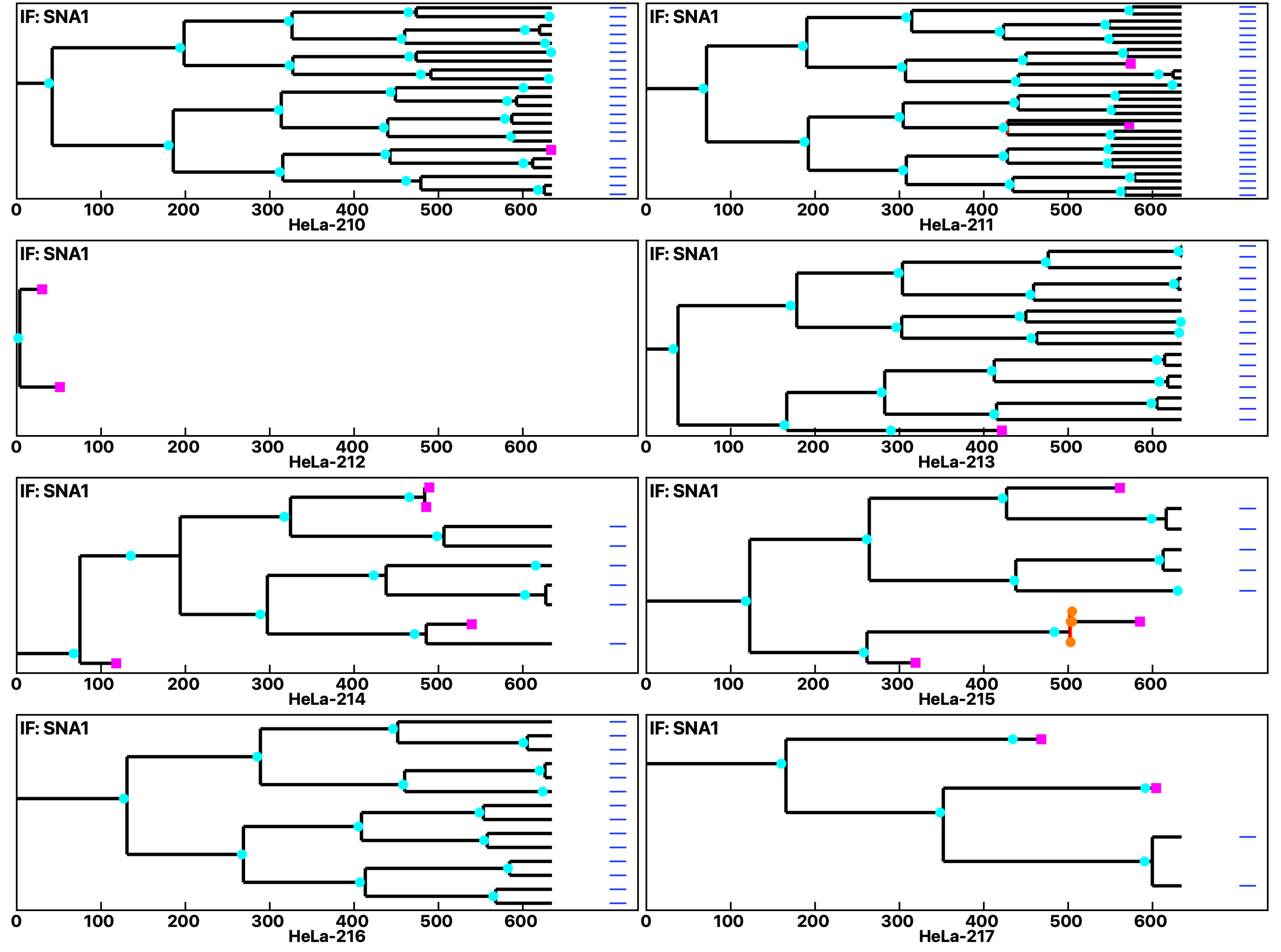

Analysis: HeLa, Treat.: HeLa, Cell: HeLa

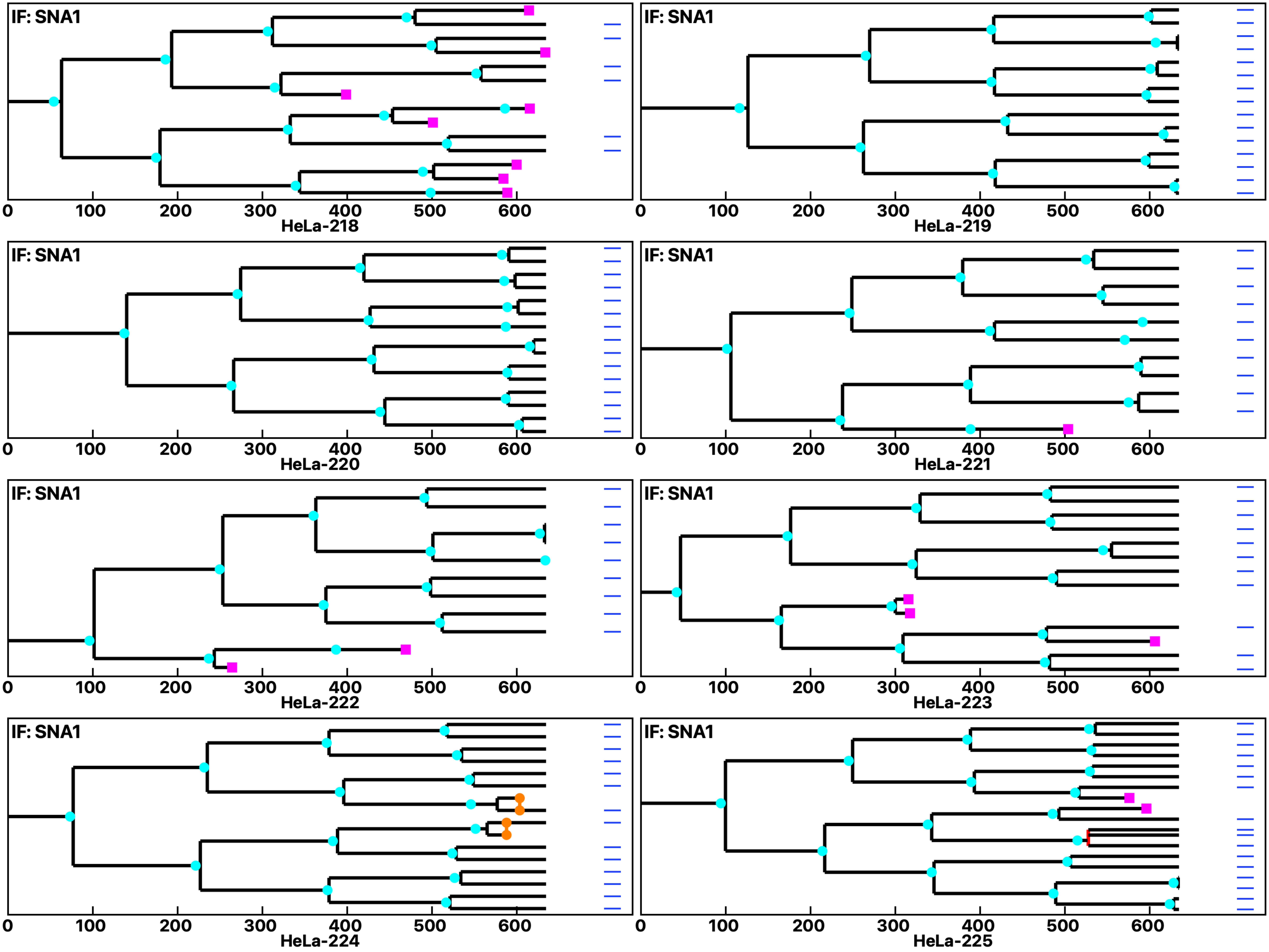

Analysis: HeLa, Treat.: HeLa, Cell: HeLa

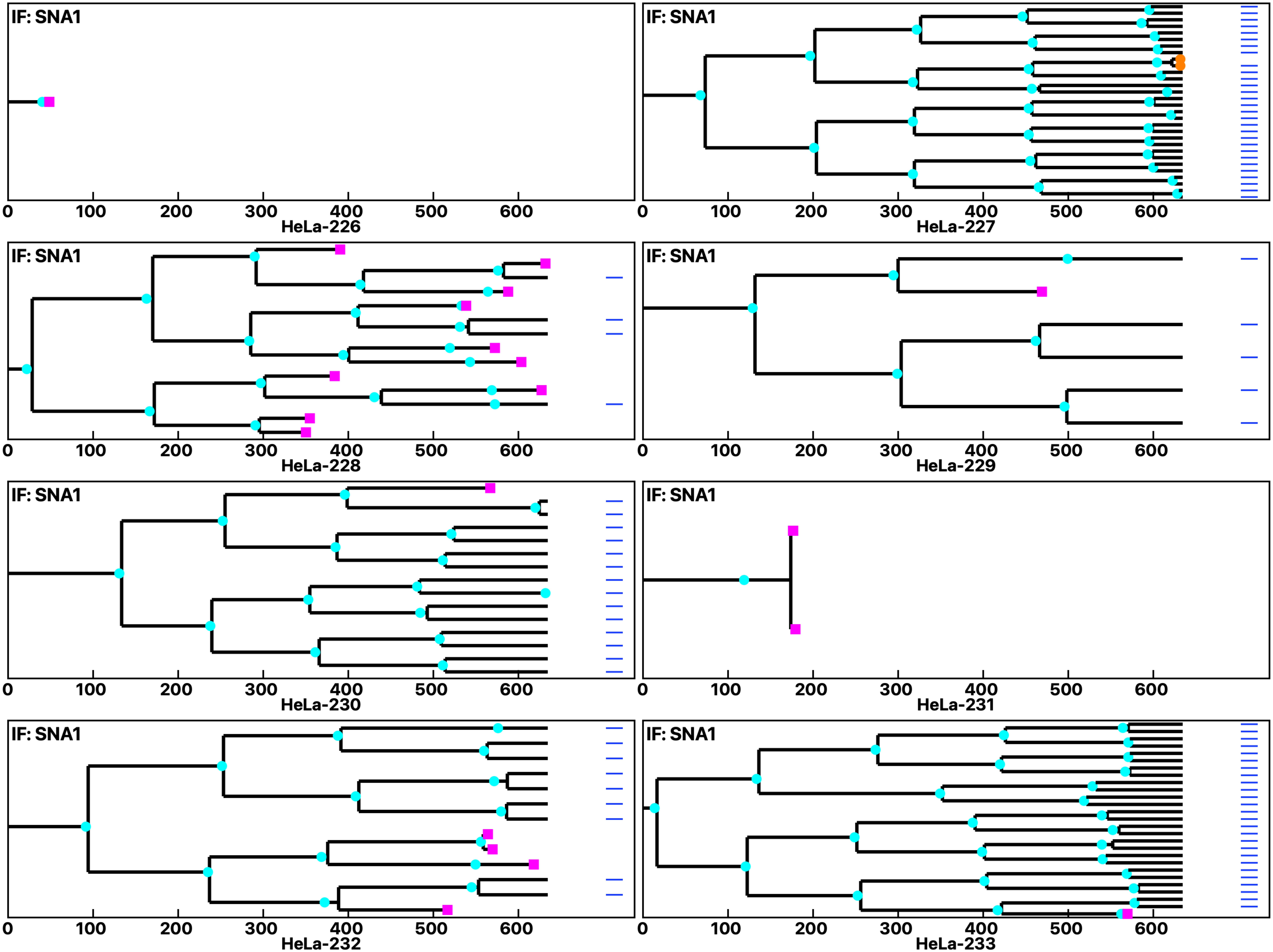

Analysis: HeLa, Treat.: HeLa, Cell: HeLa

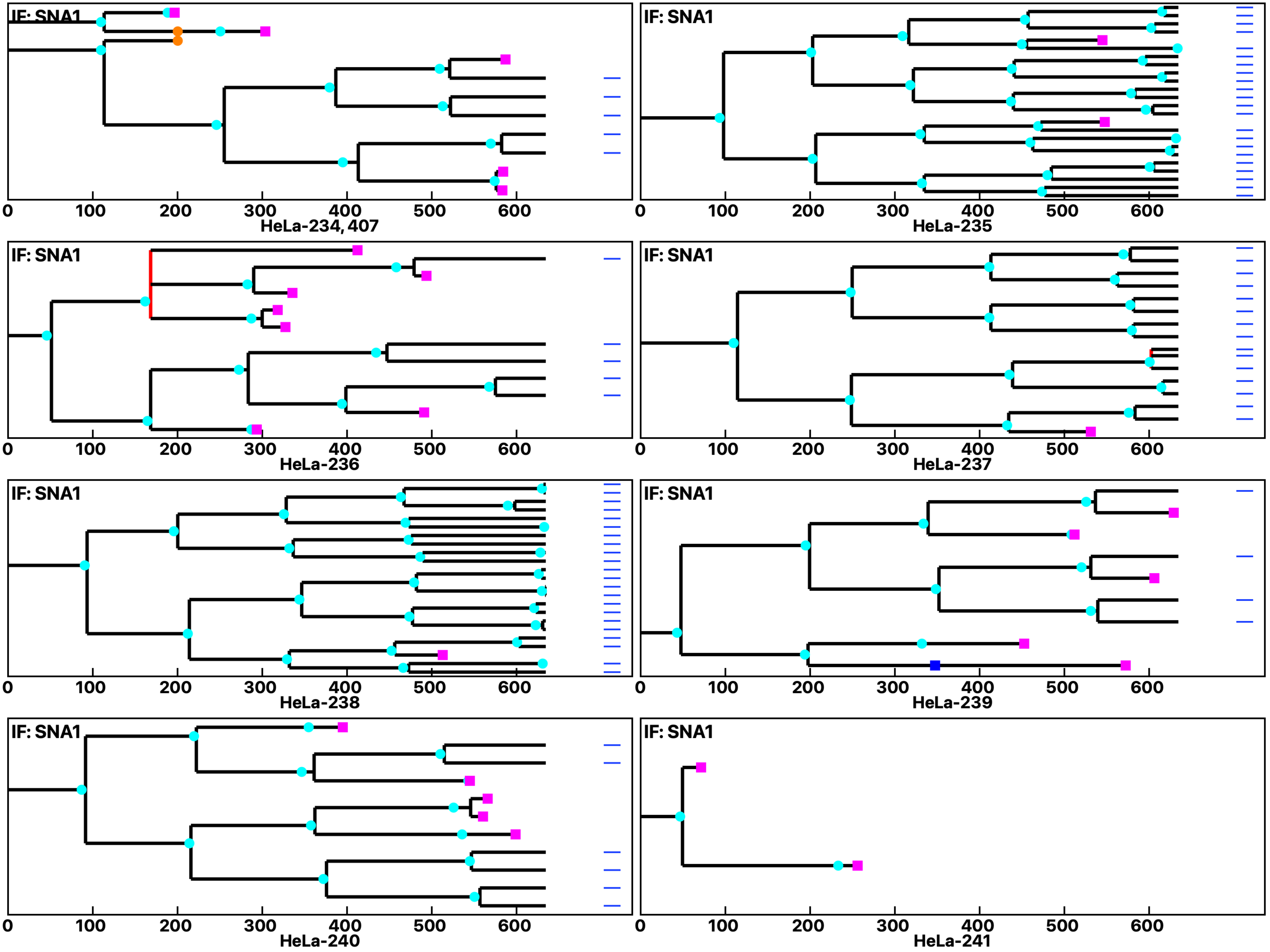

Analysis: HeLa, Treat.: HeLa, Cell: HeLa

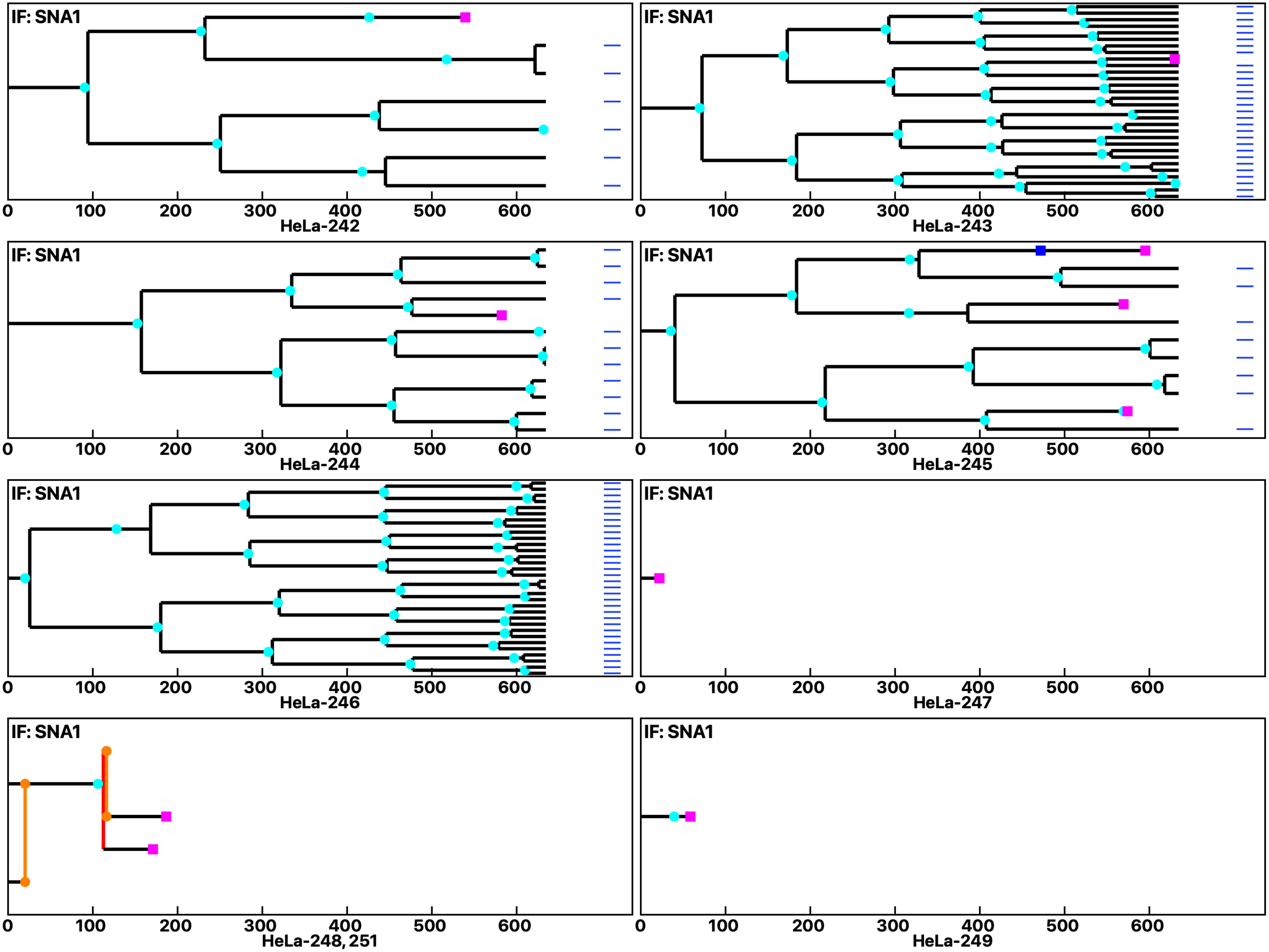

Analysis: HeLa, Treat.: HeLa, Cell: HeLa

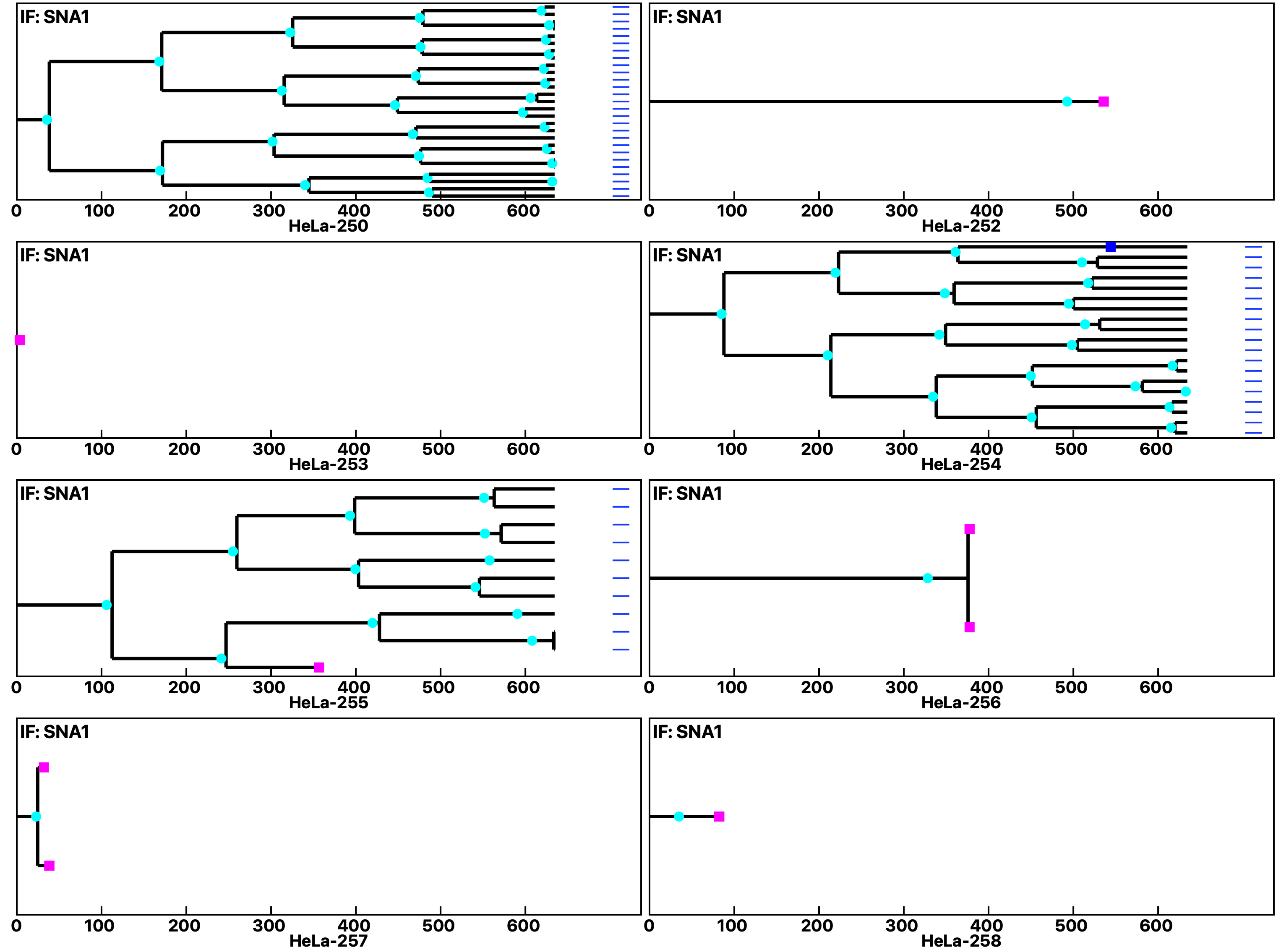

Analysis: HeLa, Treat.: HeLa, Cell: HeLa

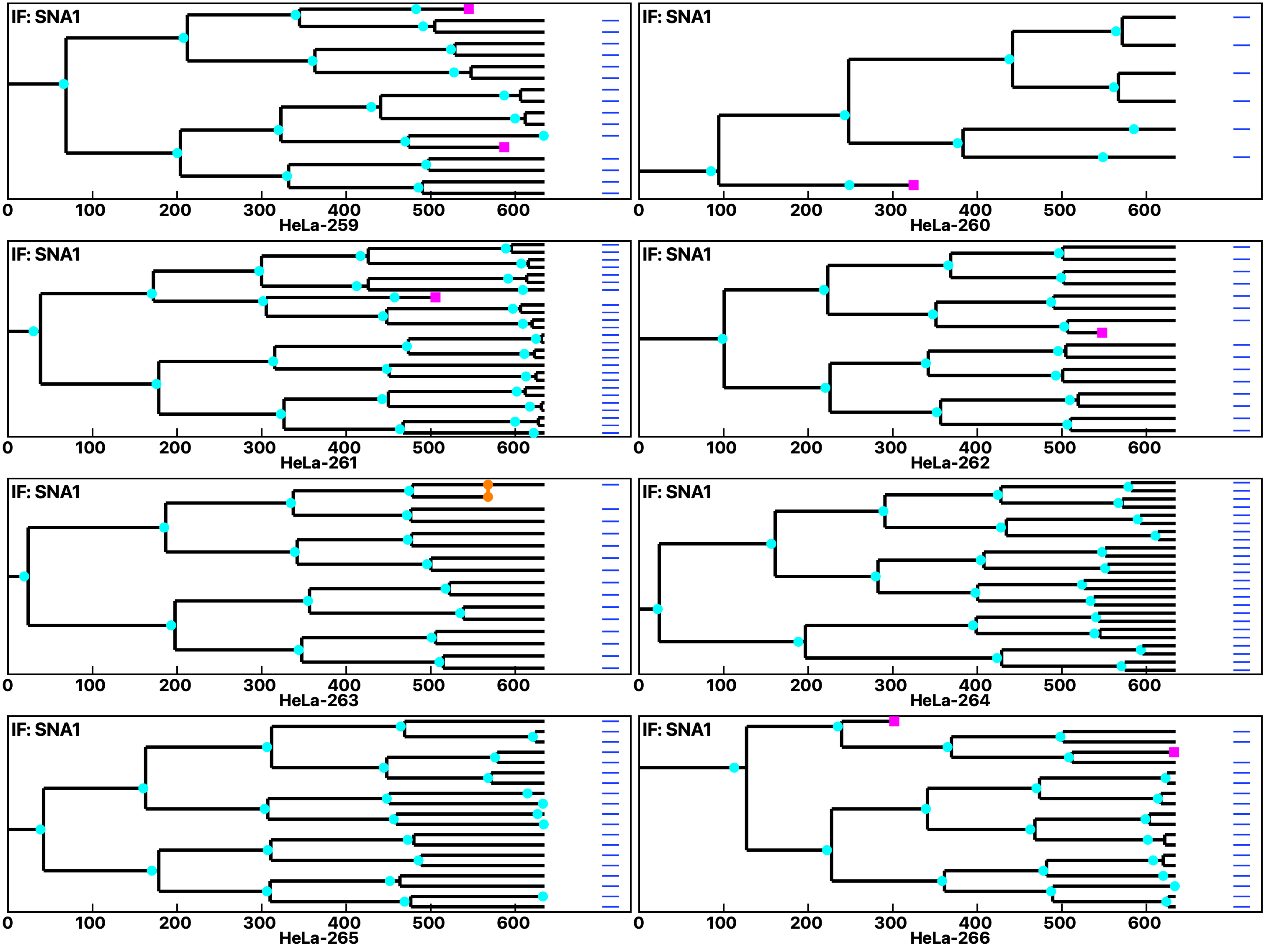

Analysis: HeLa, Treat.: HeLa, Cell: HeLa

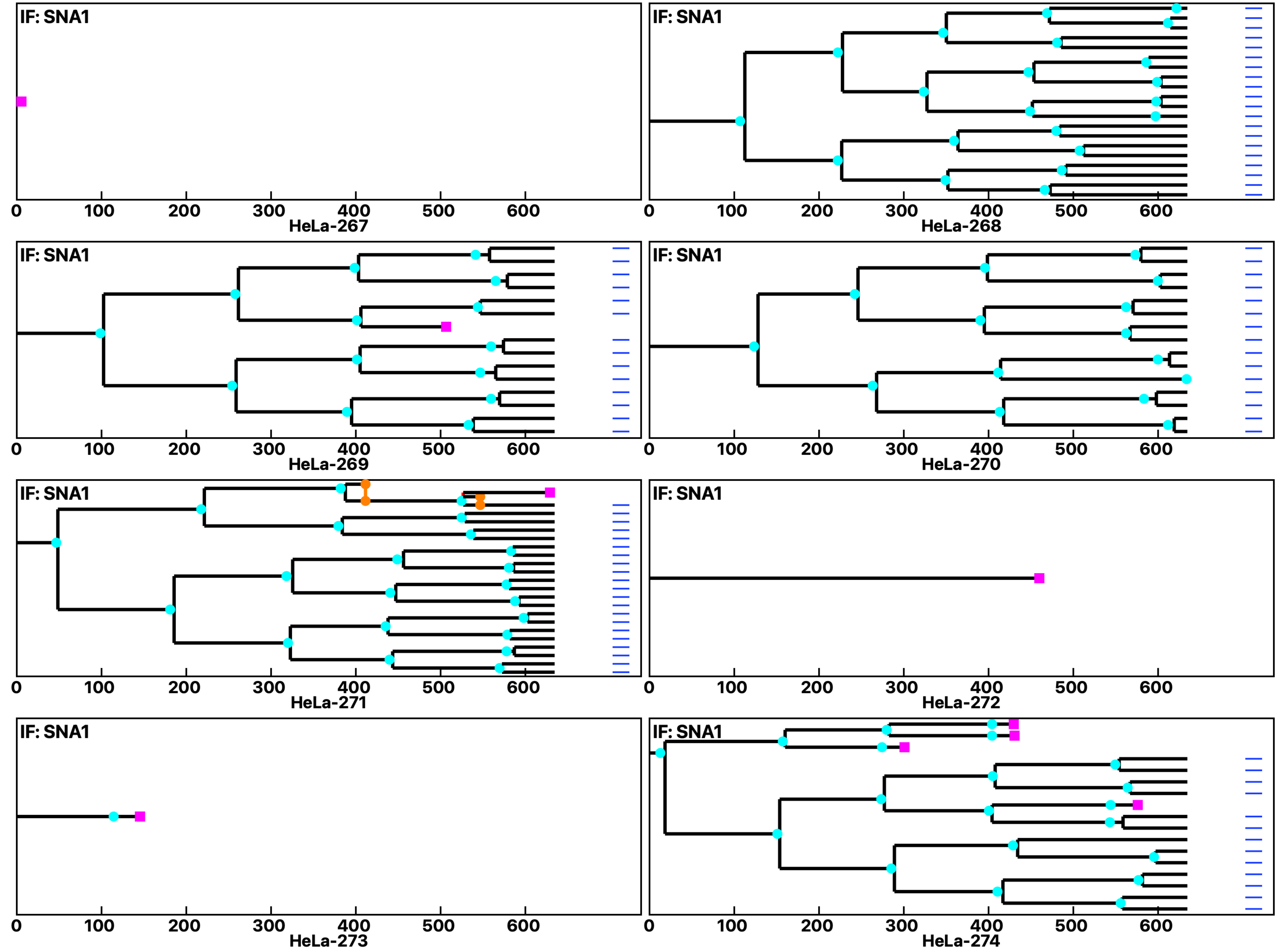

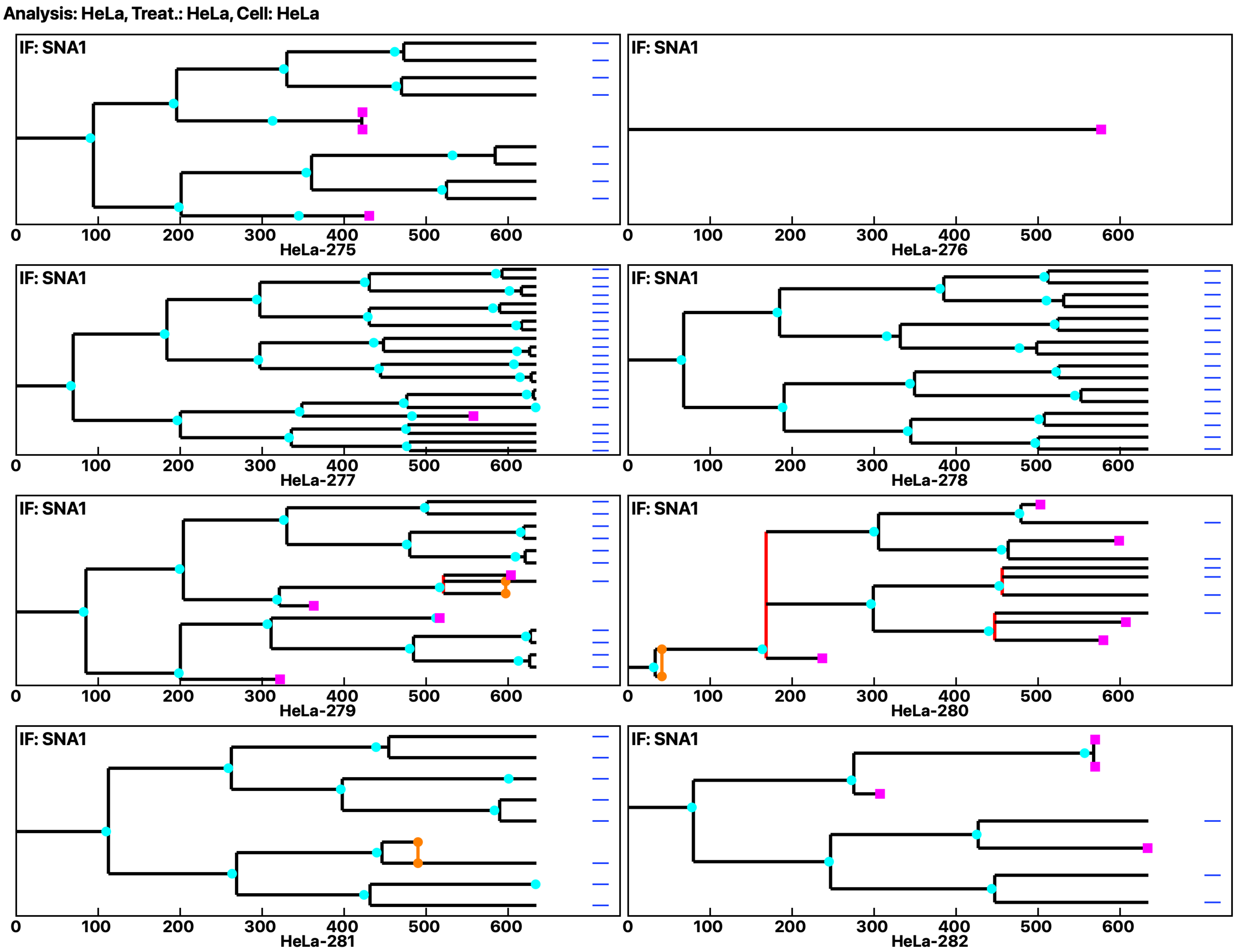

Analysis: HeLa, Treat.: HeLa, Cell: HeLa
IF: SNA1
0
100
200
300
400
500
600
HeLa-275
HeLa-276
HeLa-277
HeLa-278
HeLa-279
HeLa-280
HeLa-281
HeLa-282

Analysis: HeLa, Treat.: HeLa, Cell: HeLa

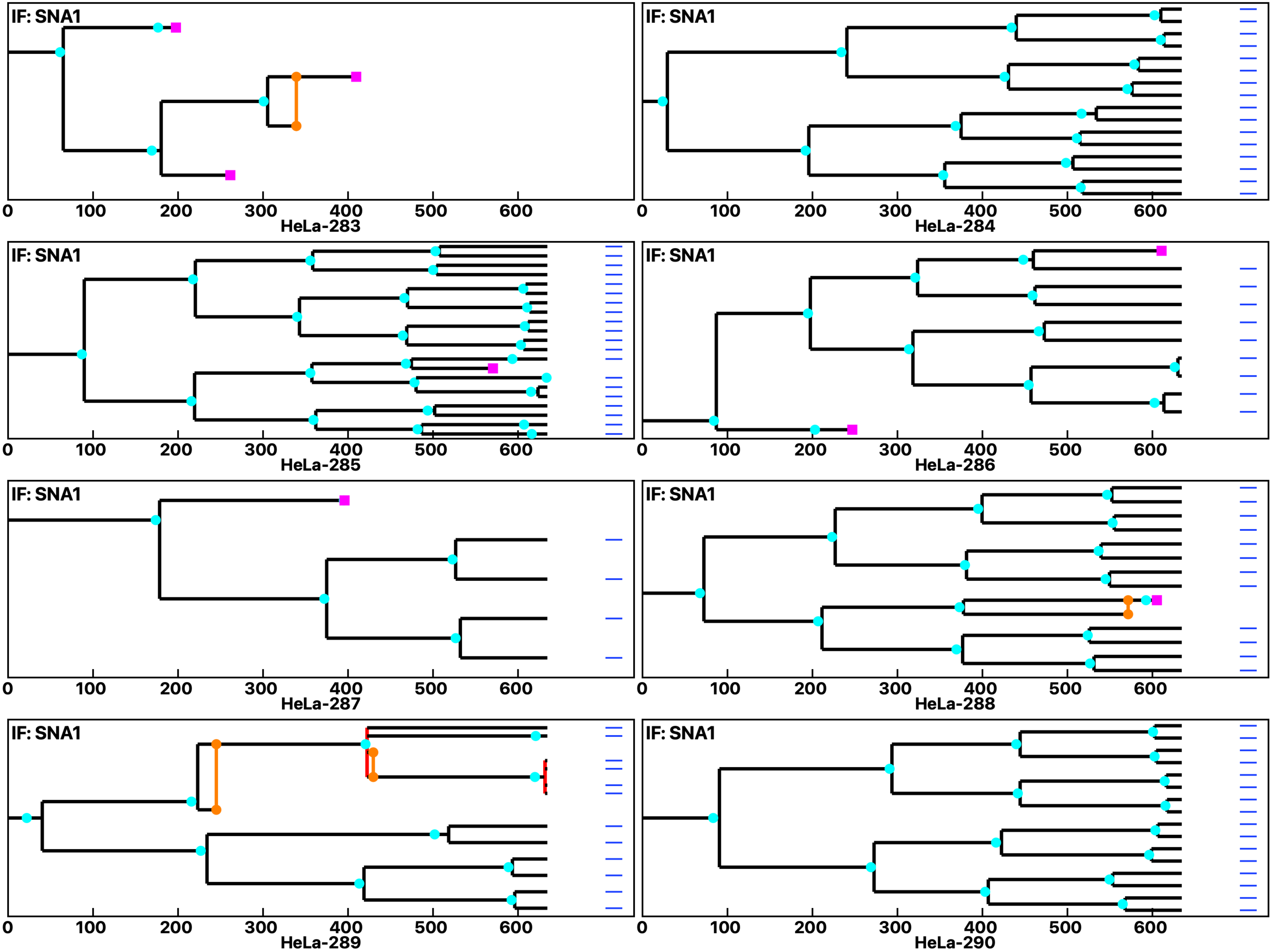

Analysis: HeLa, Treat.: HeLa, Cell: HeLa

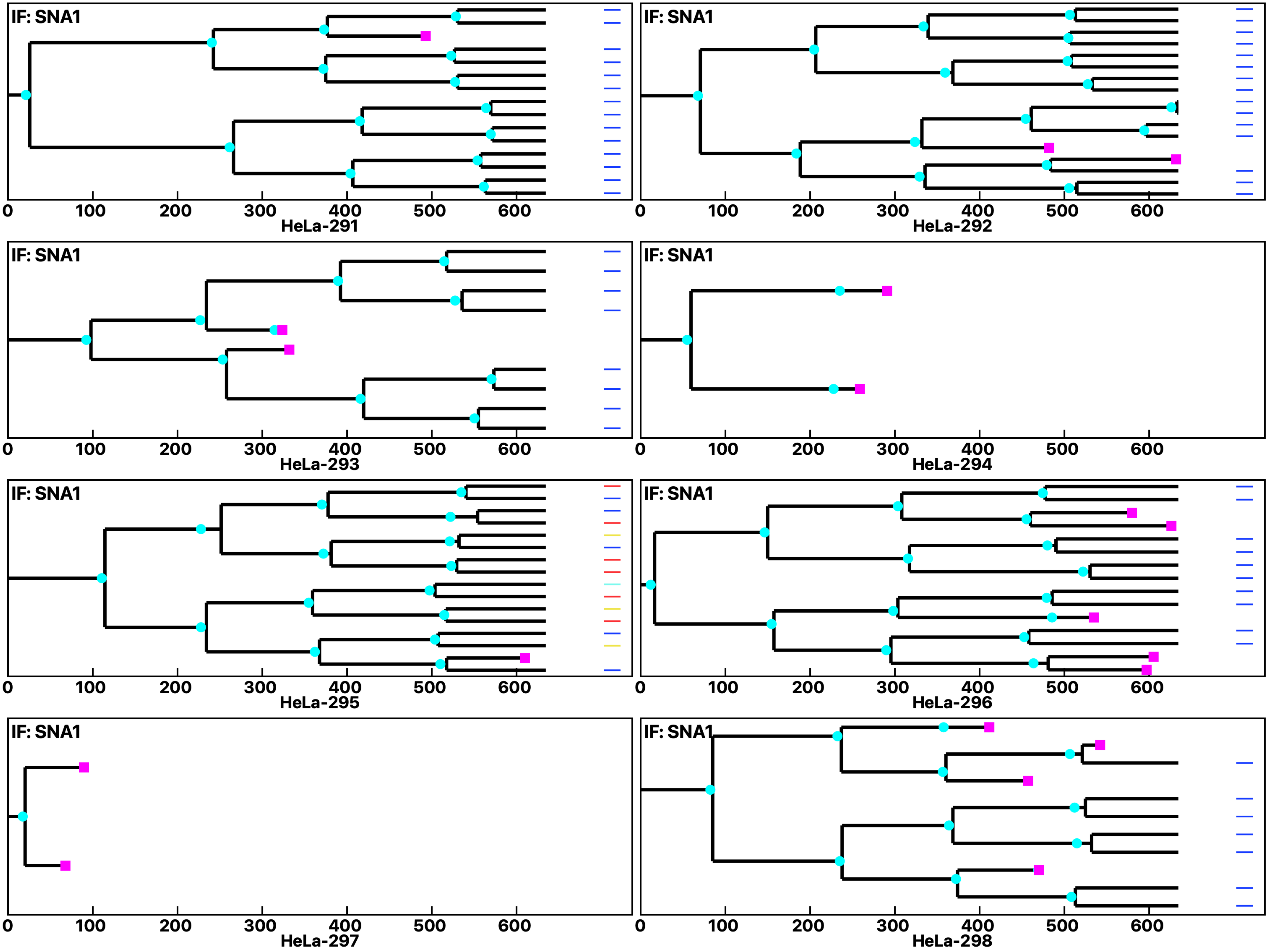

Analysis: HeLa, Treat.: HeLa, Cell: HeLa

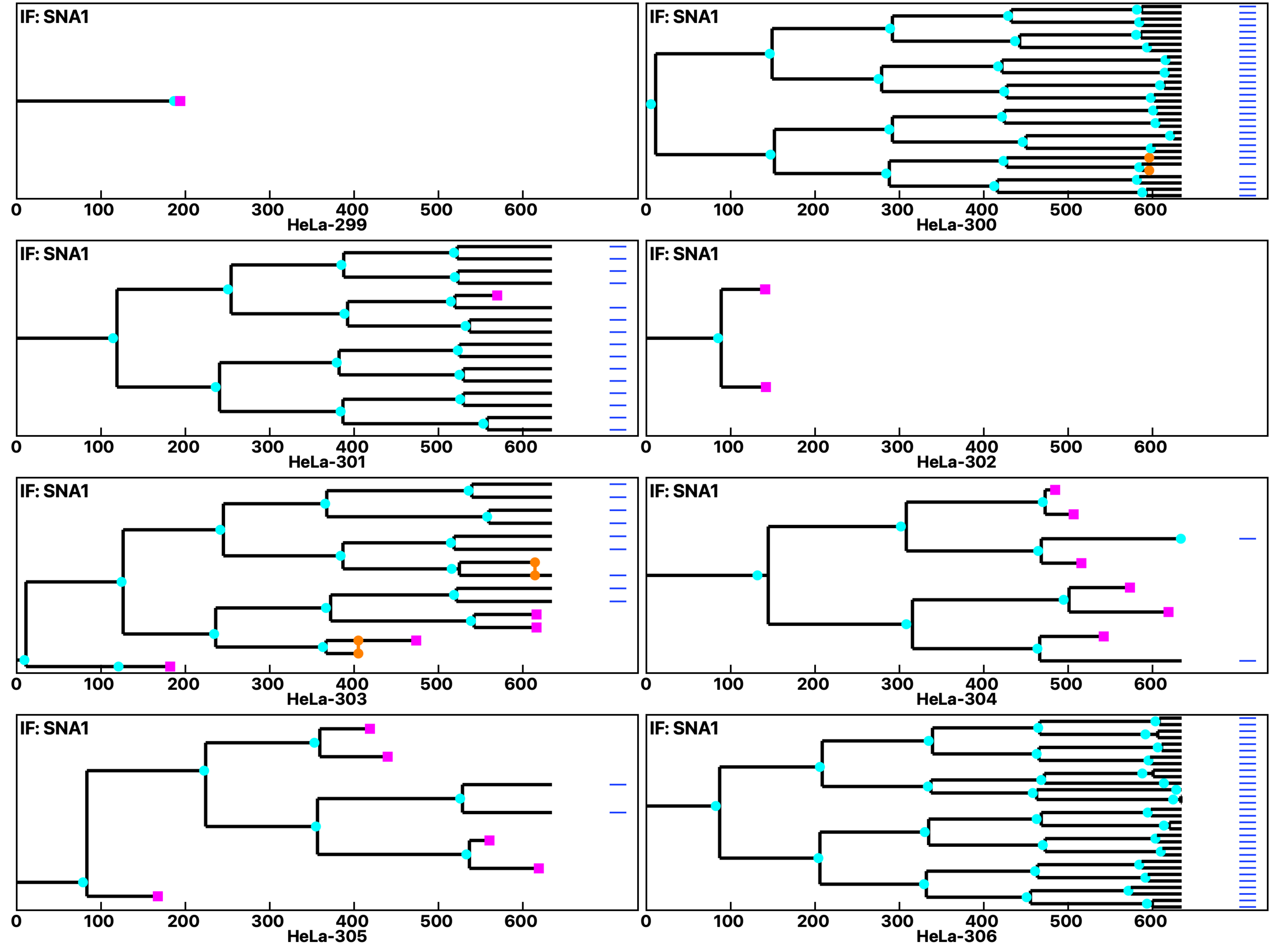

Analysis: HeLa, Treat.: HeLa, Cell: HeLa

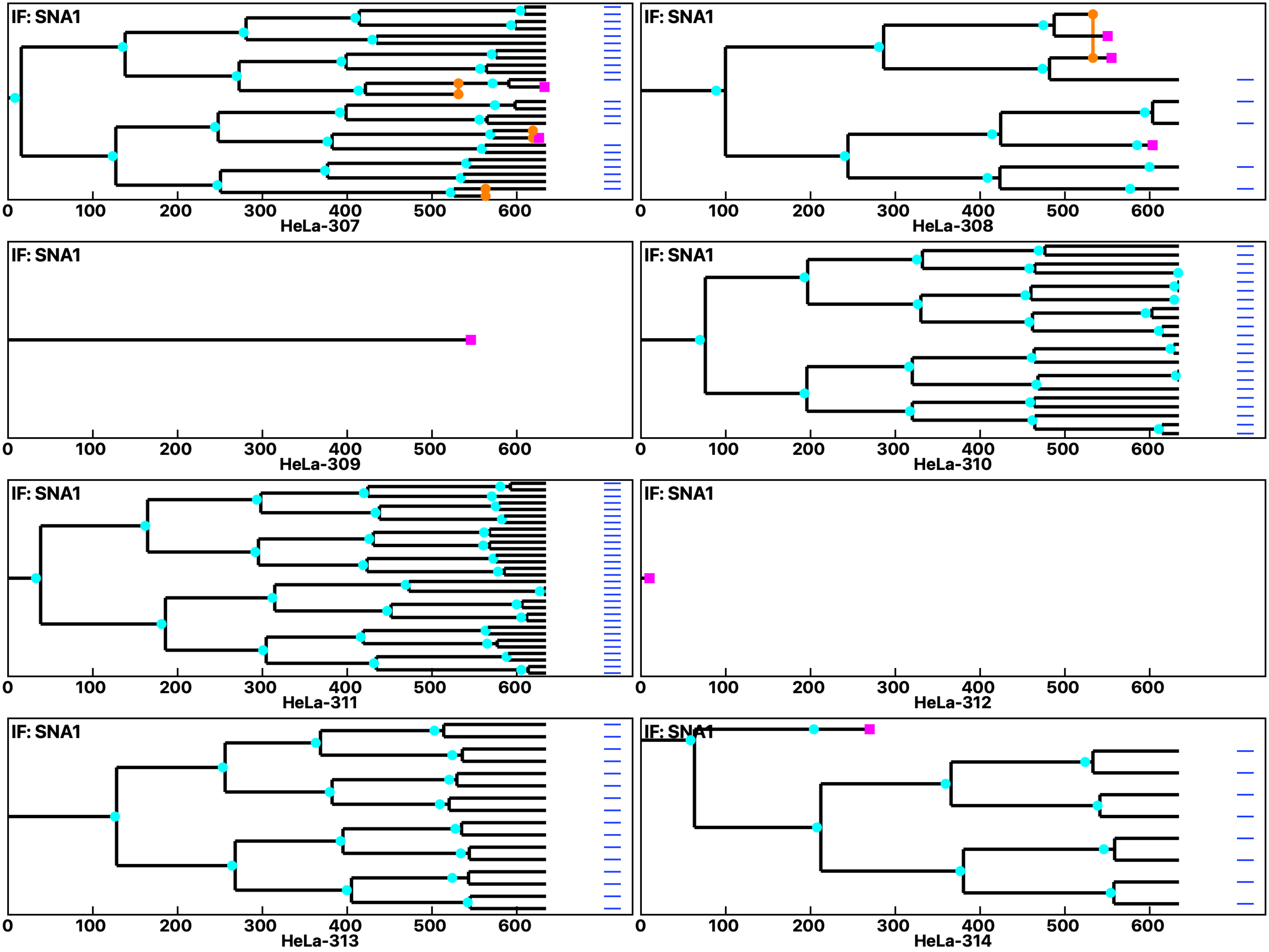

Analysis: HeLa, Treat.: HeLa, Cell: HeLa

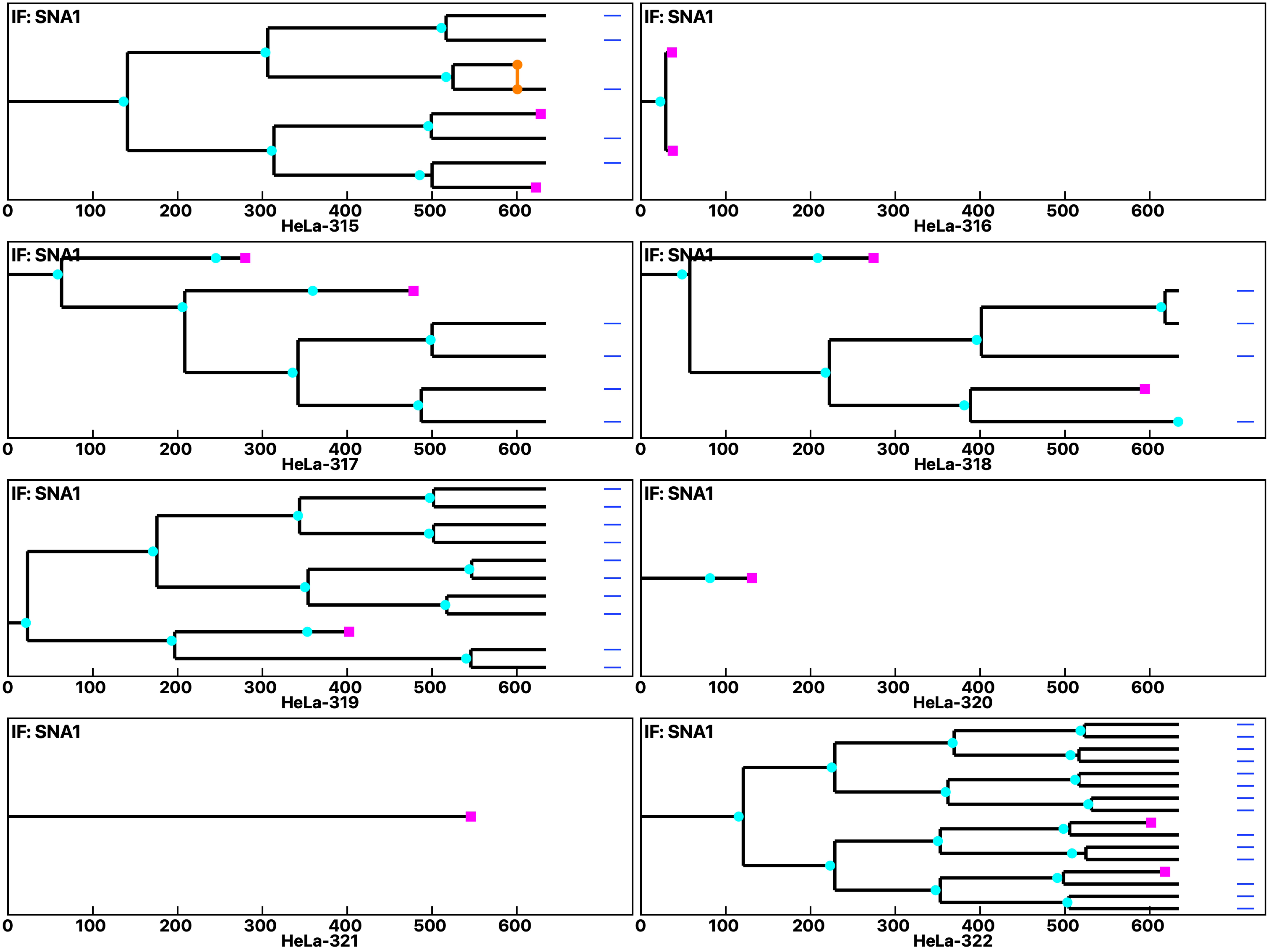

Analysis: HeLa, Treat.: HeLa, Cell: HeLa

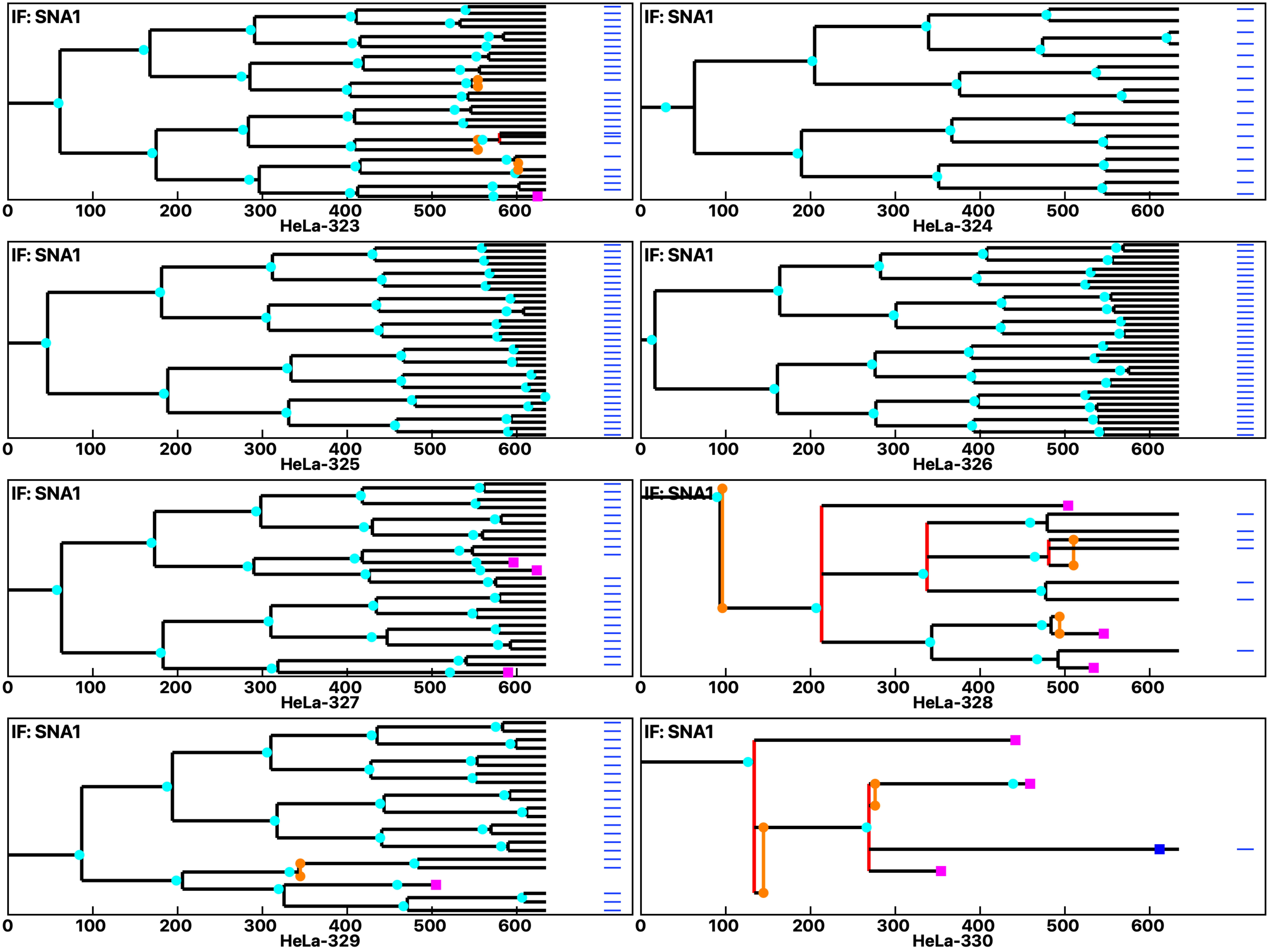

Analysis: HeLa, Treat.: HeLa, Cell: HeLa

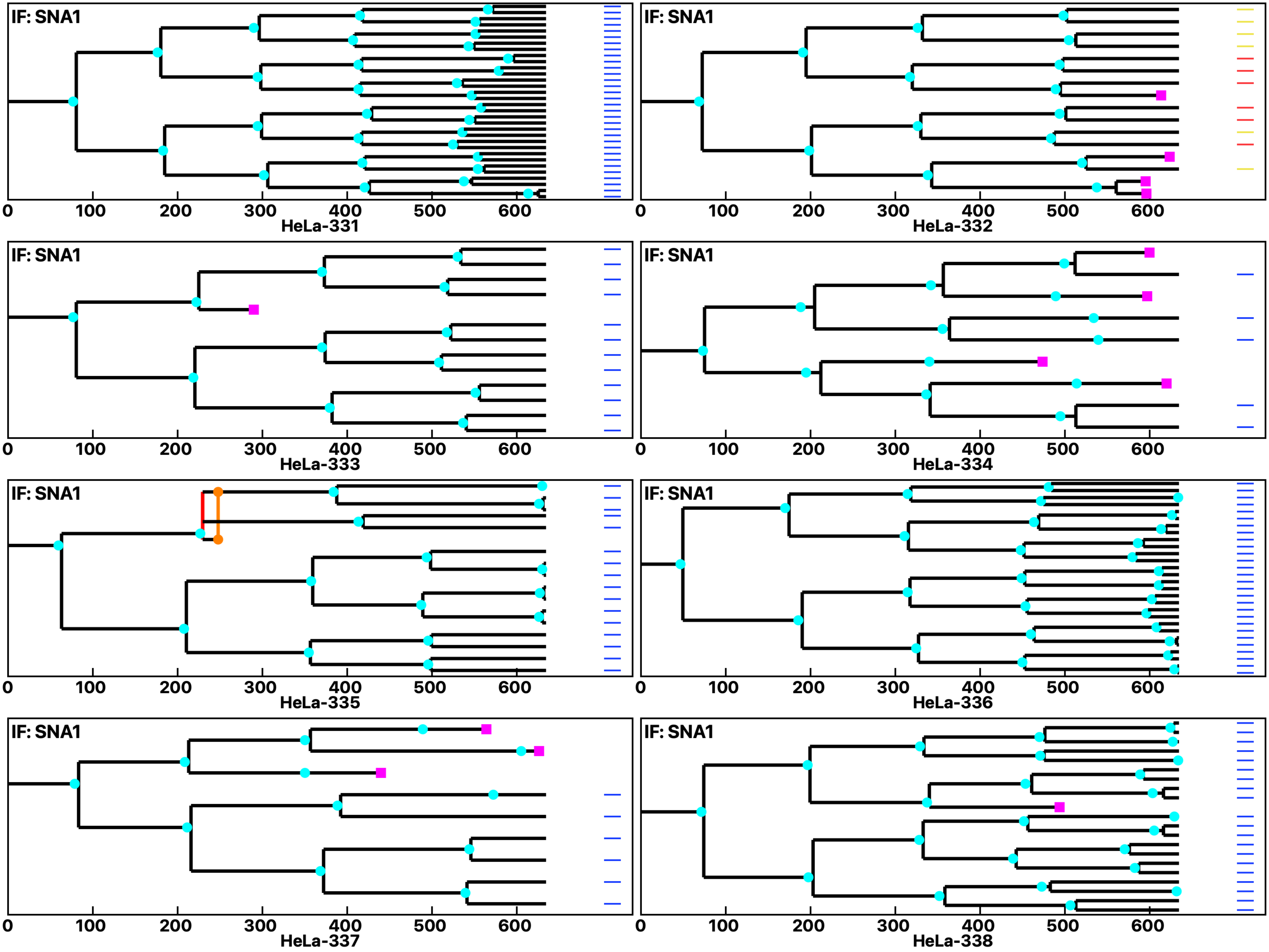

Analysis: HeLa, Treat.: HeLa, Cell: HeLa

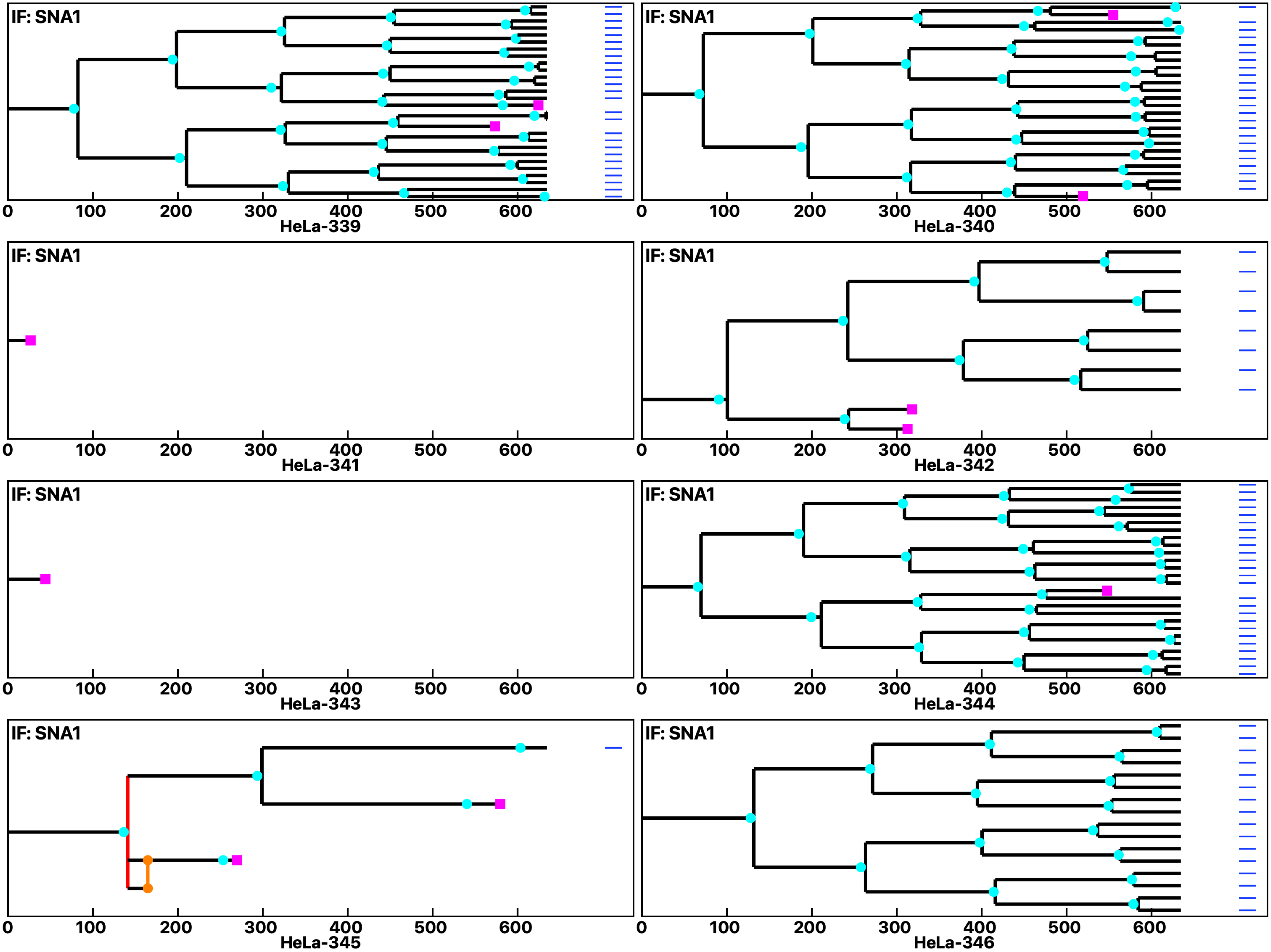

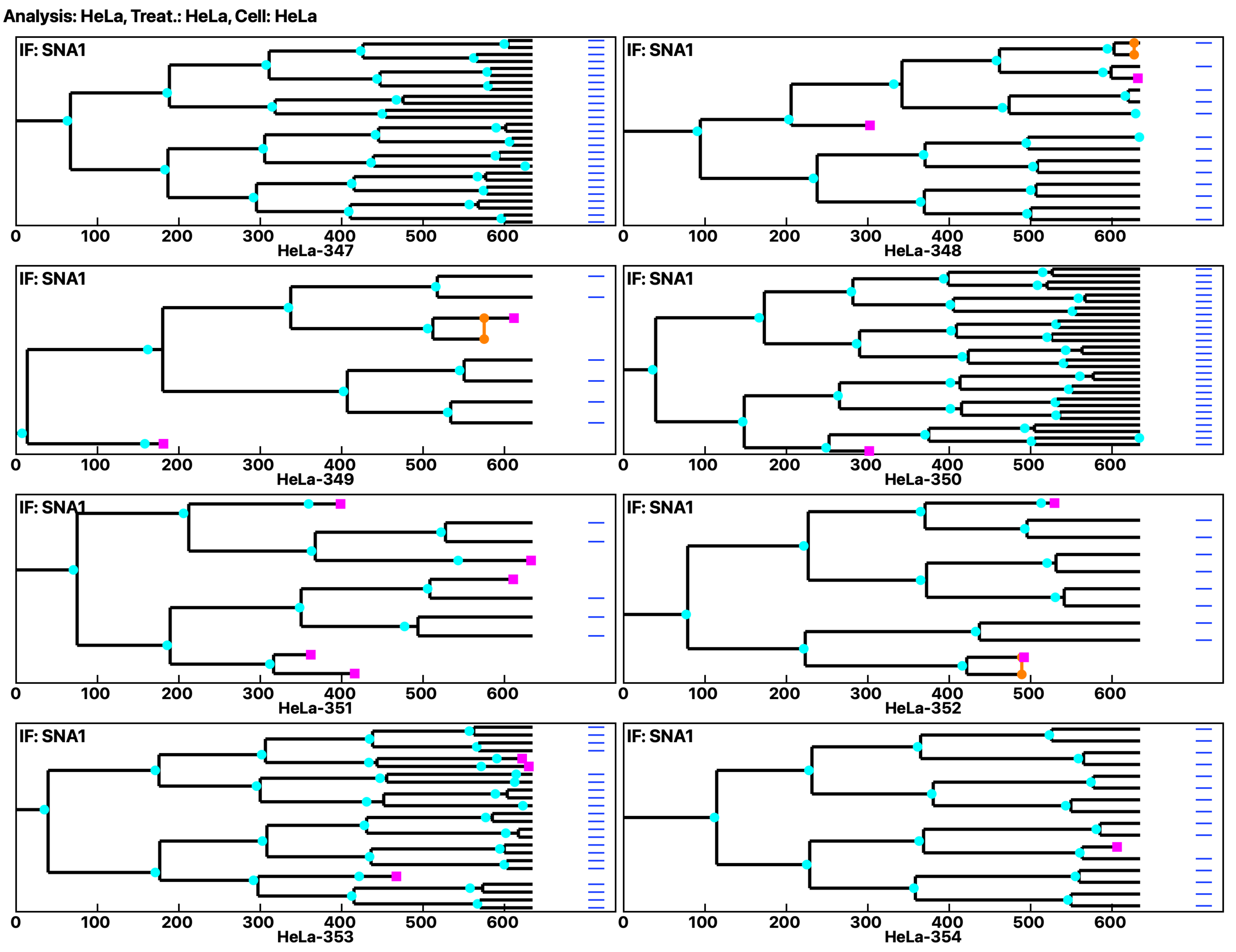
Analysis: HeLa, Treat.: HeLa, Cell: HeLa
IF: SNA1
0
100
200
300
400
500
600
HeLa-347
HeLa-348
HeLa-349
HeLa-350
HeLa-351
HeLa-352
HeLa-353
HeLa-354

Analysis: HeLa, Treat.: HeLa, Cell: HeLa

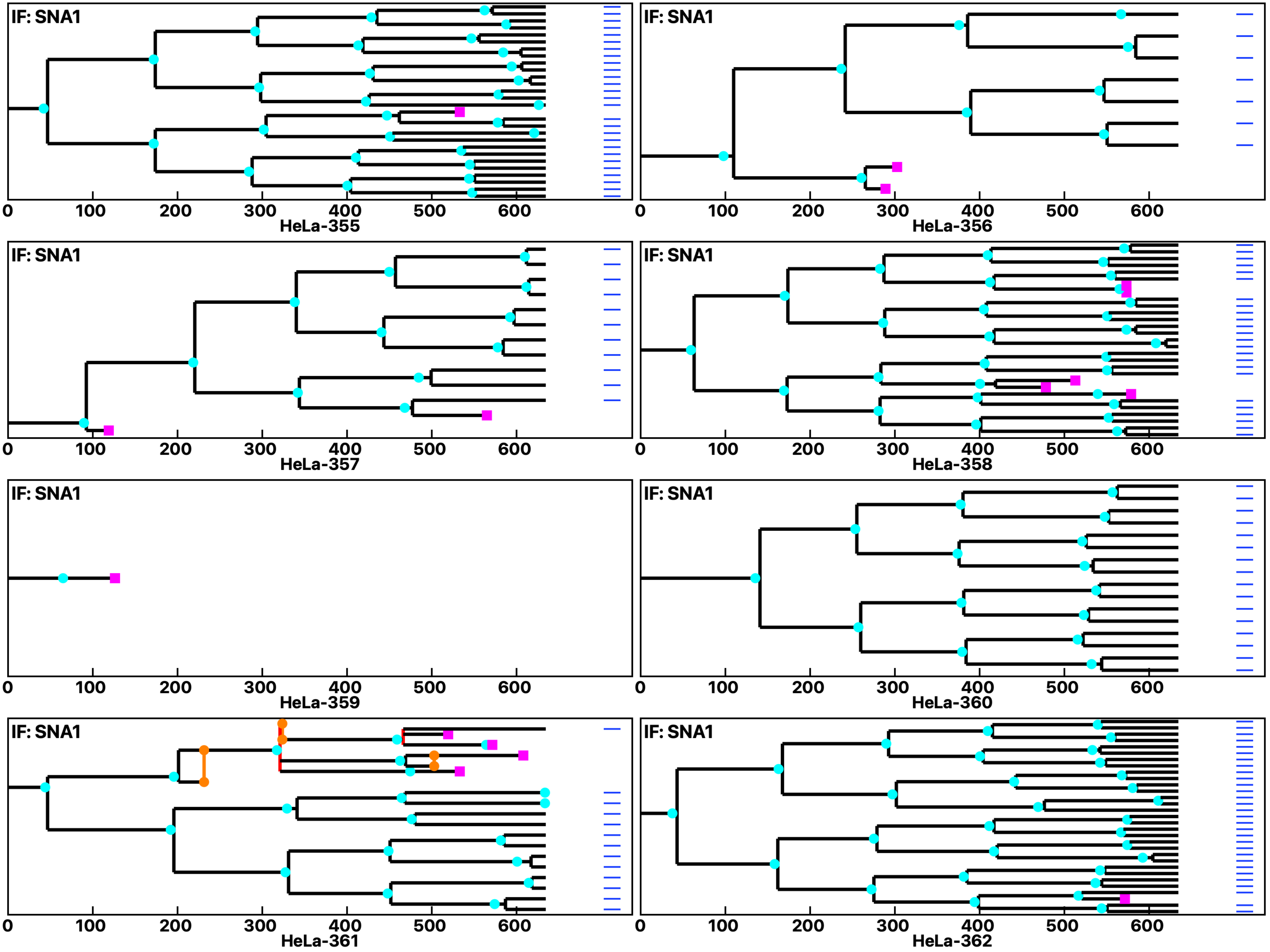

Analysis: HeLa, Treat.: HeLa, Cell: HeLa

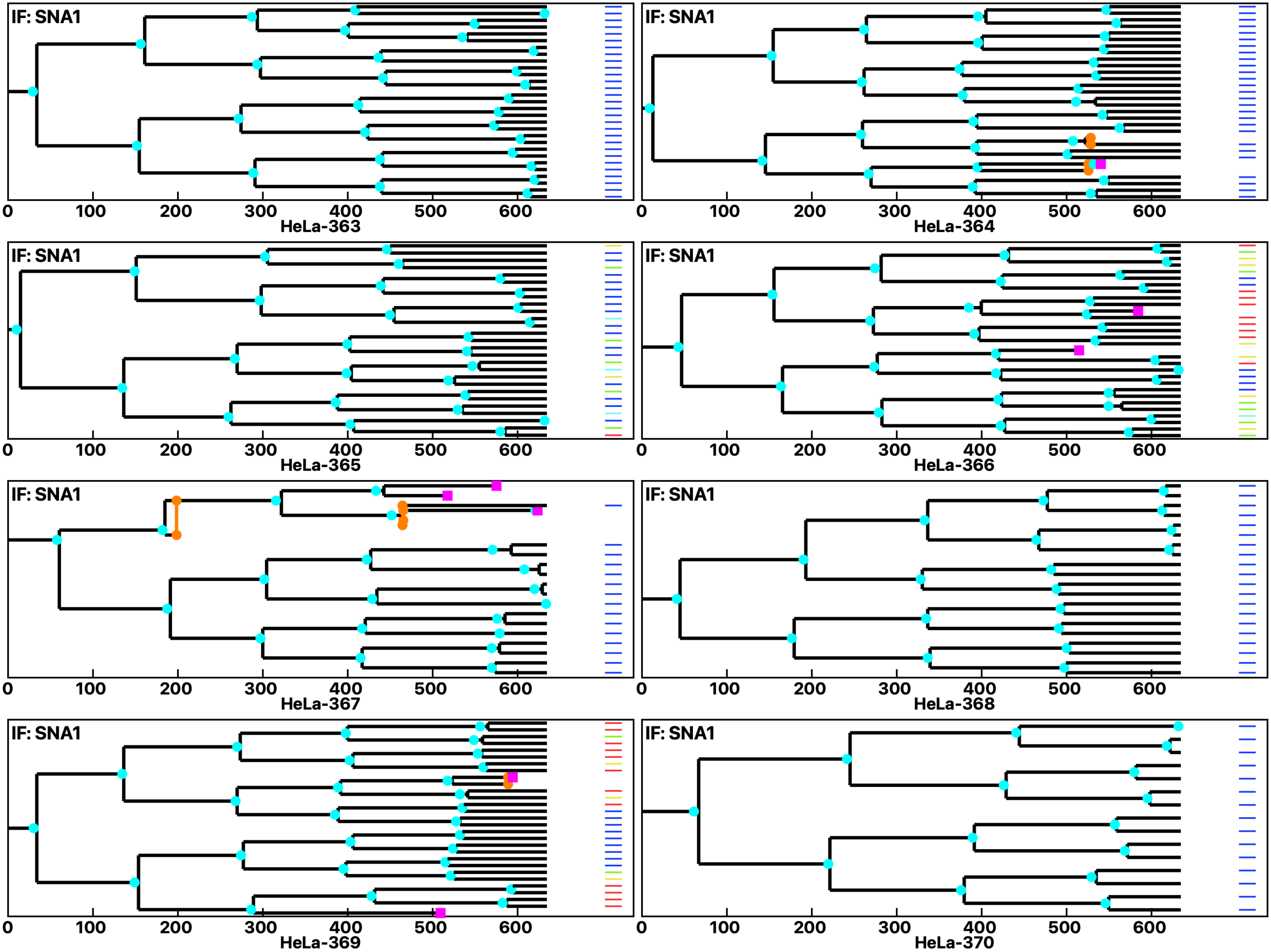

Analysis: HeLa, Treat.: HeLa, Cell: HeLa

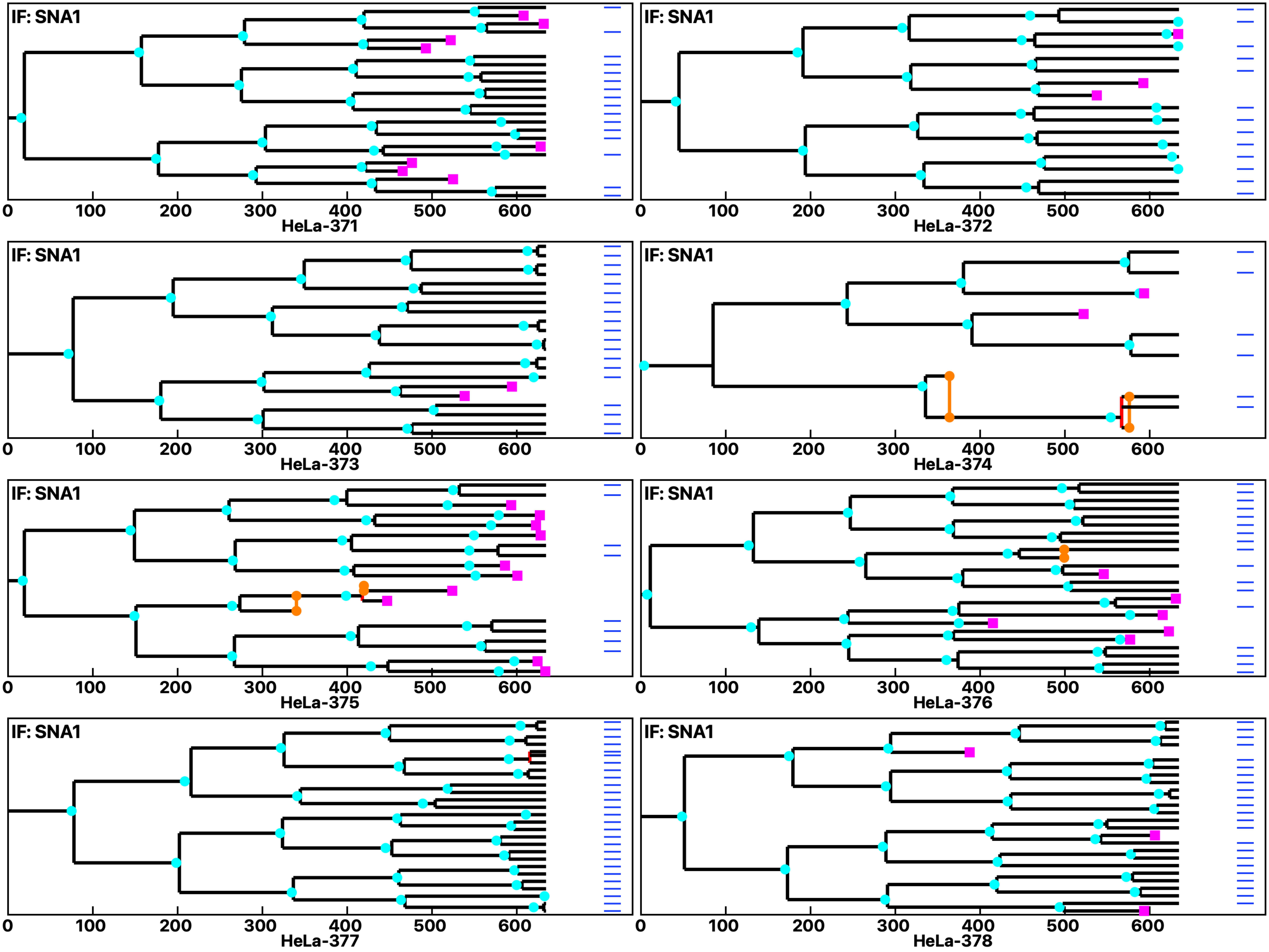

Analysis: HeLa, Treat.: HeLa, Cell: HeLa

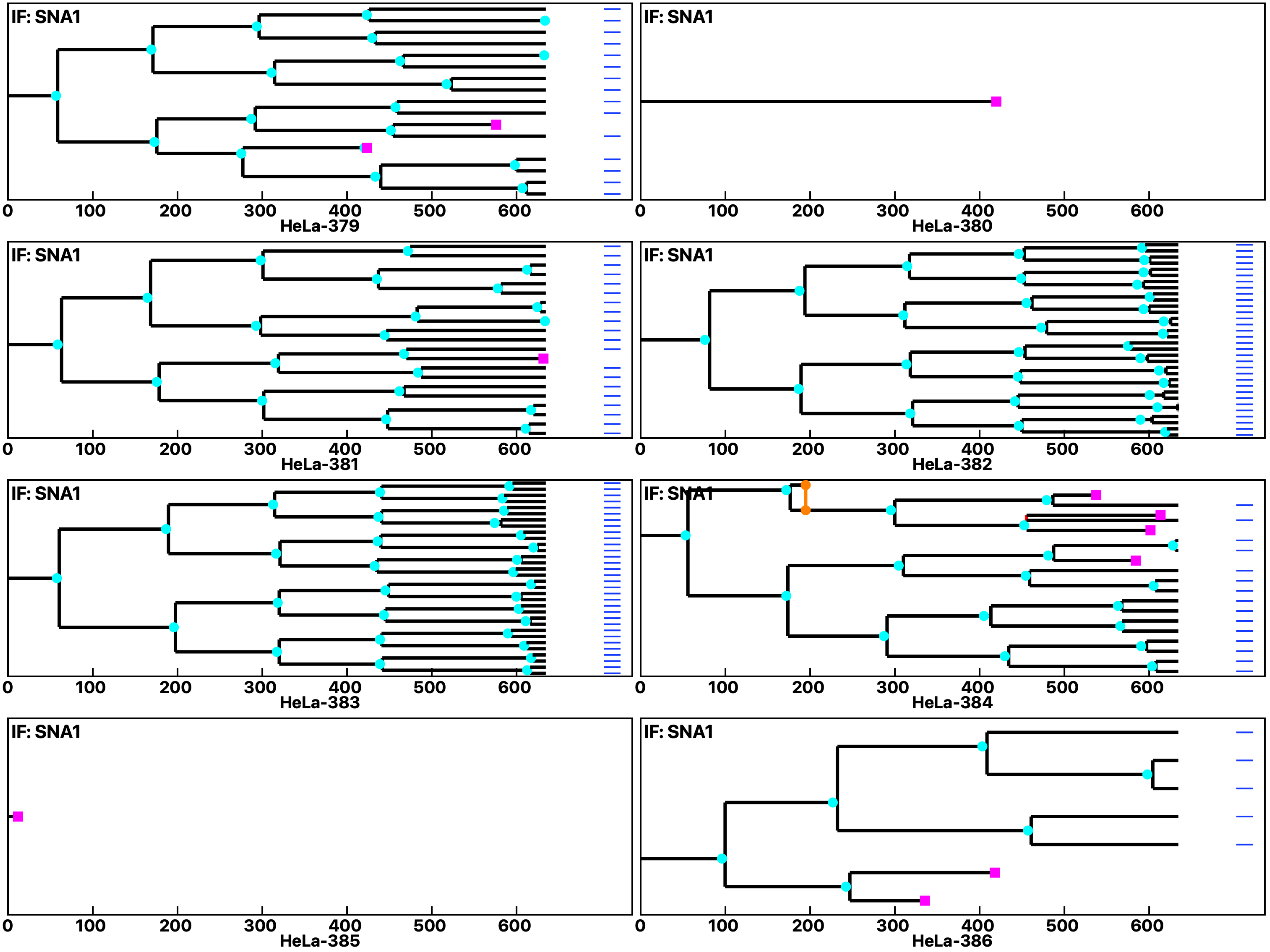

Analysis: HeLa, Treat.: HeLa, Cell: HeLa

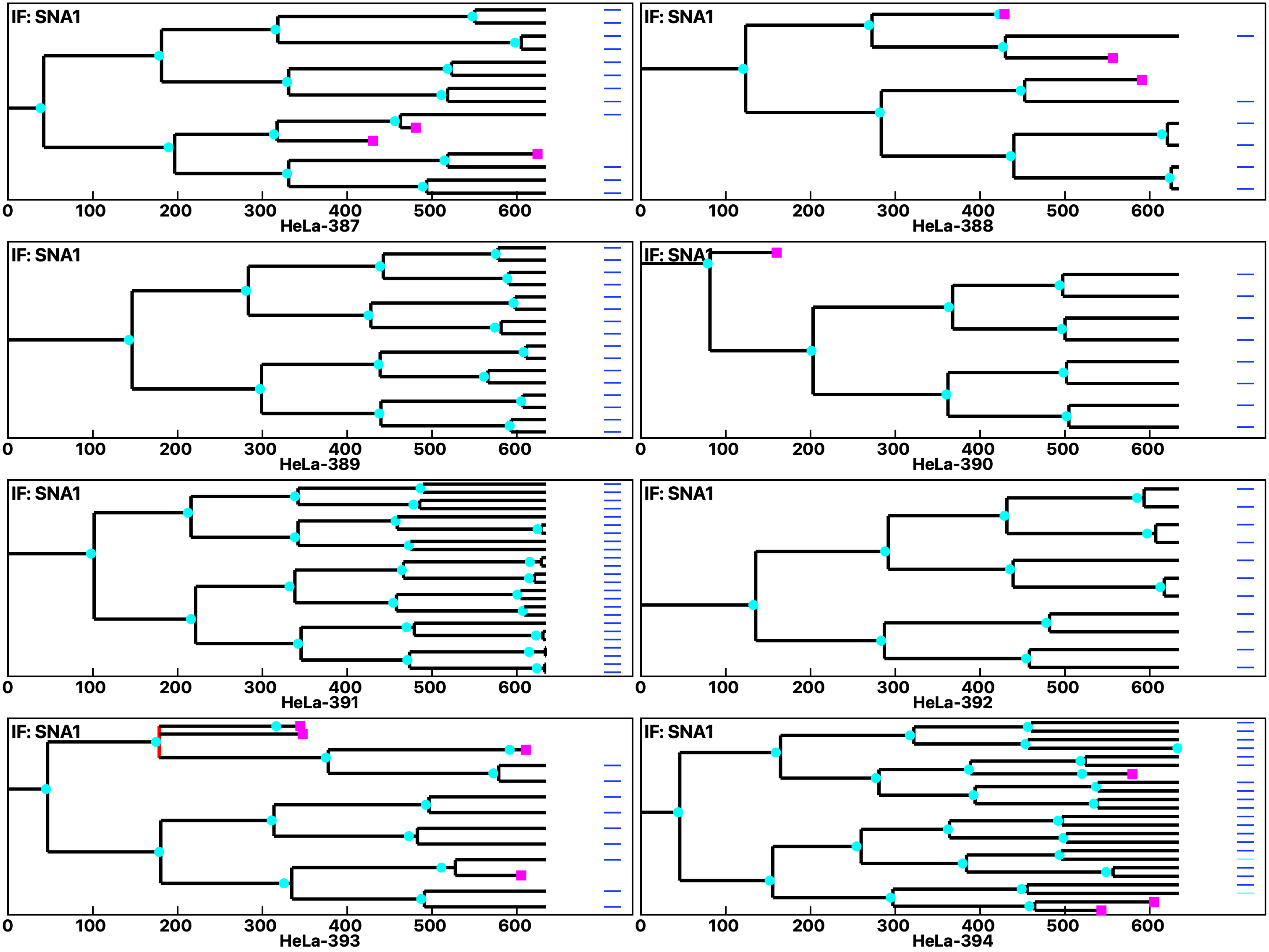

Analysis: HeLa, Treat.: HeLa, Cell: HeLa

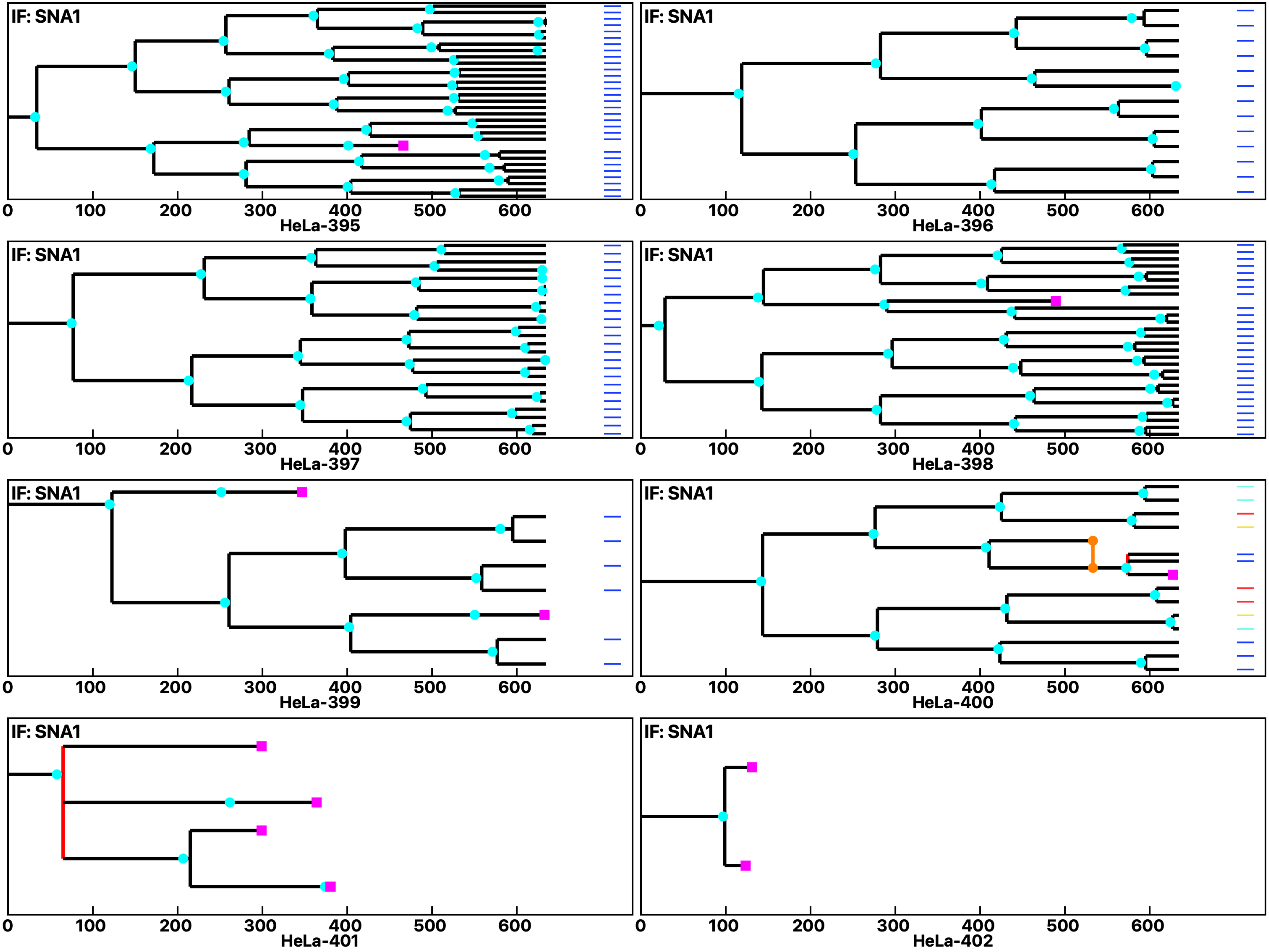

Analysis: HeLa, Treat.: HeLa, Cell: HeLa

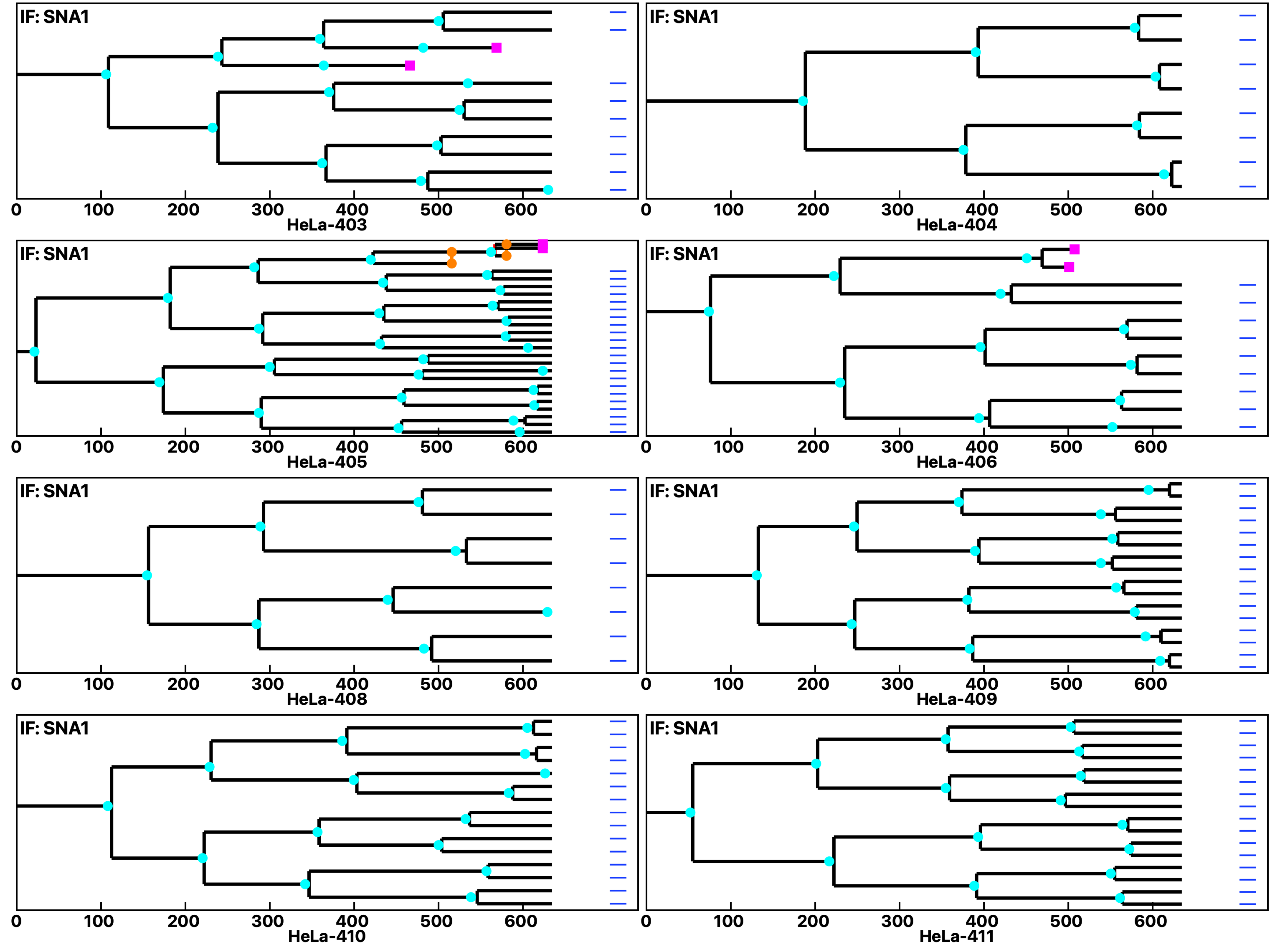

Analysis: HeLa, Treat.: HeLa, Cell: HeLa

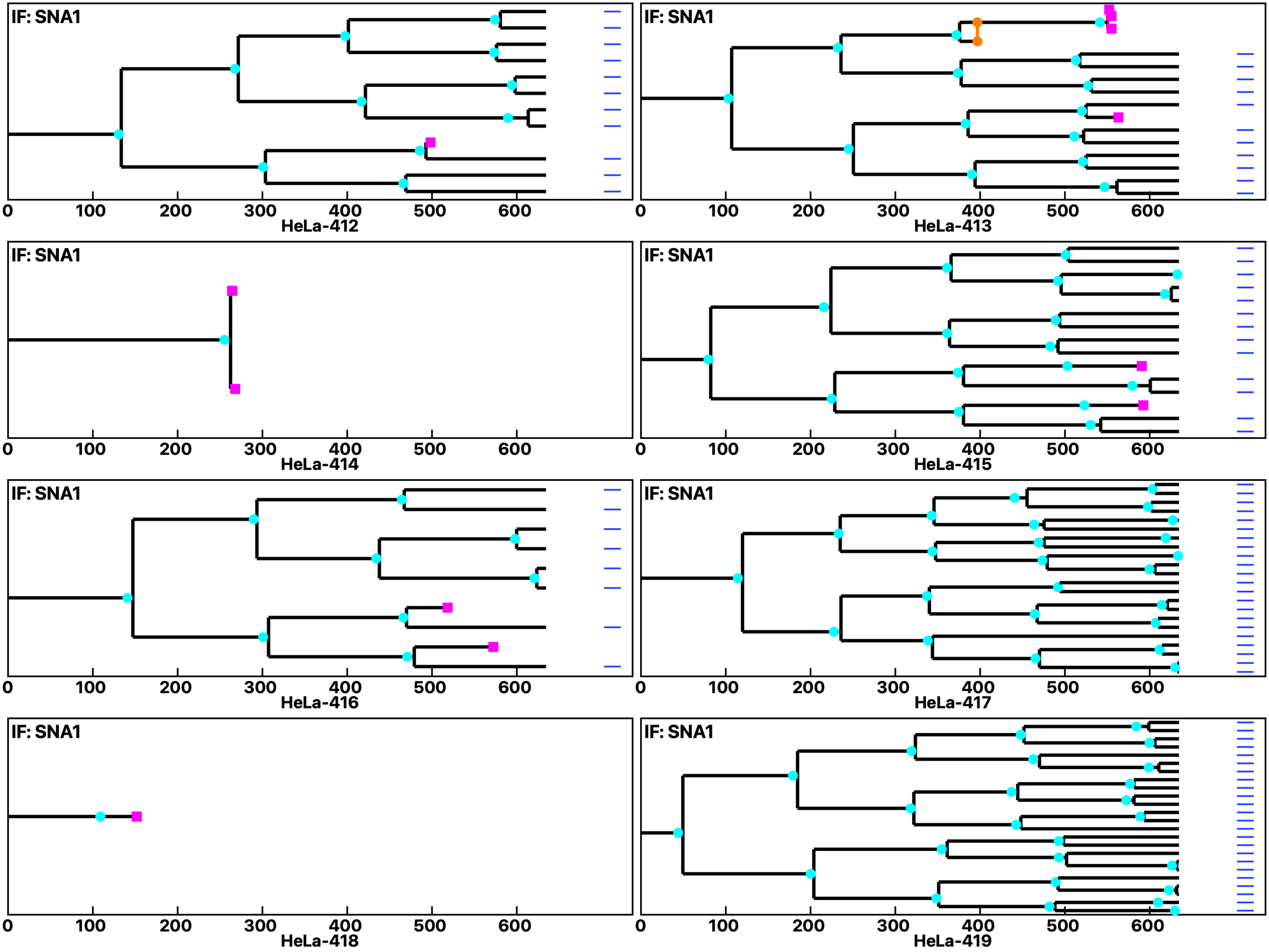

Analysis: HeLa, Treat.: HeLa, Cell: HeLa

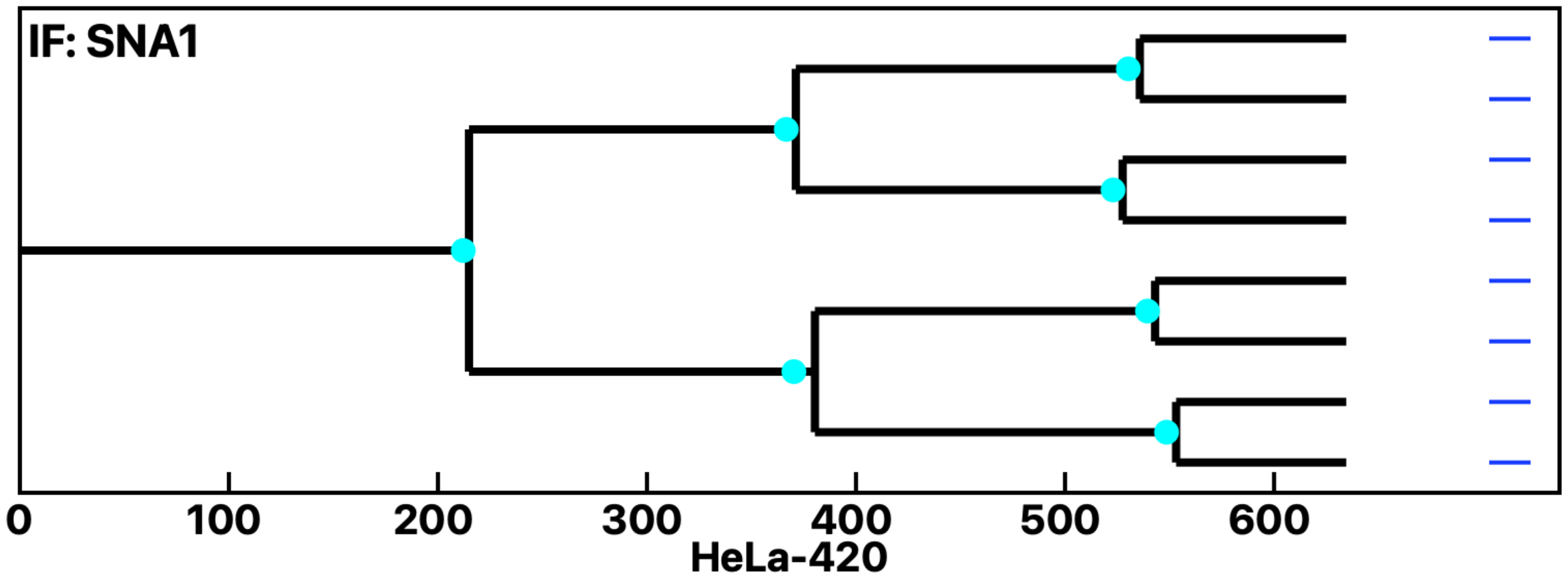

Supplement: Data S1 [file mmc3.pdf]
