# Supplementary figures and images for: Cell fate simulation reveals cancer cell features in the tumor microenvironment
Source: J Biol Chem. 2024 Aug 20;300(9):107697. doi: 10.1016/j.jbc.2024.107697 (PMC11419826; doi:10.1016/j.jbc.2024.107697)

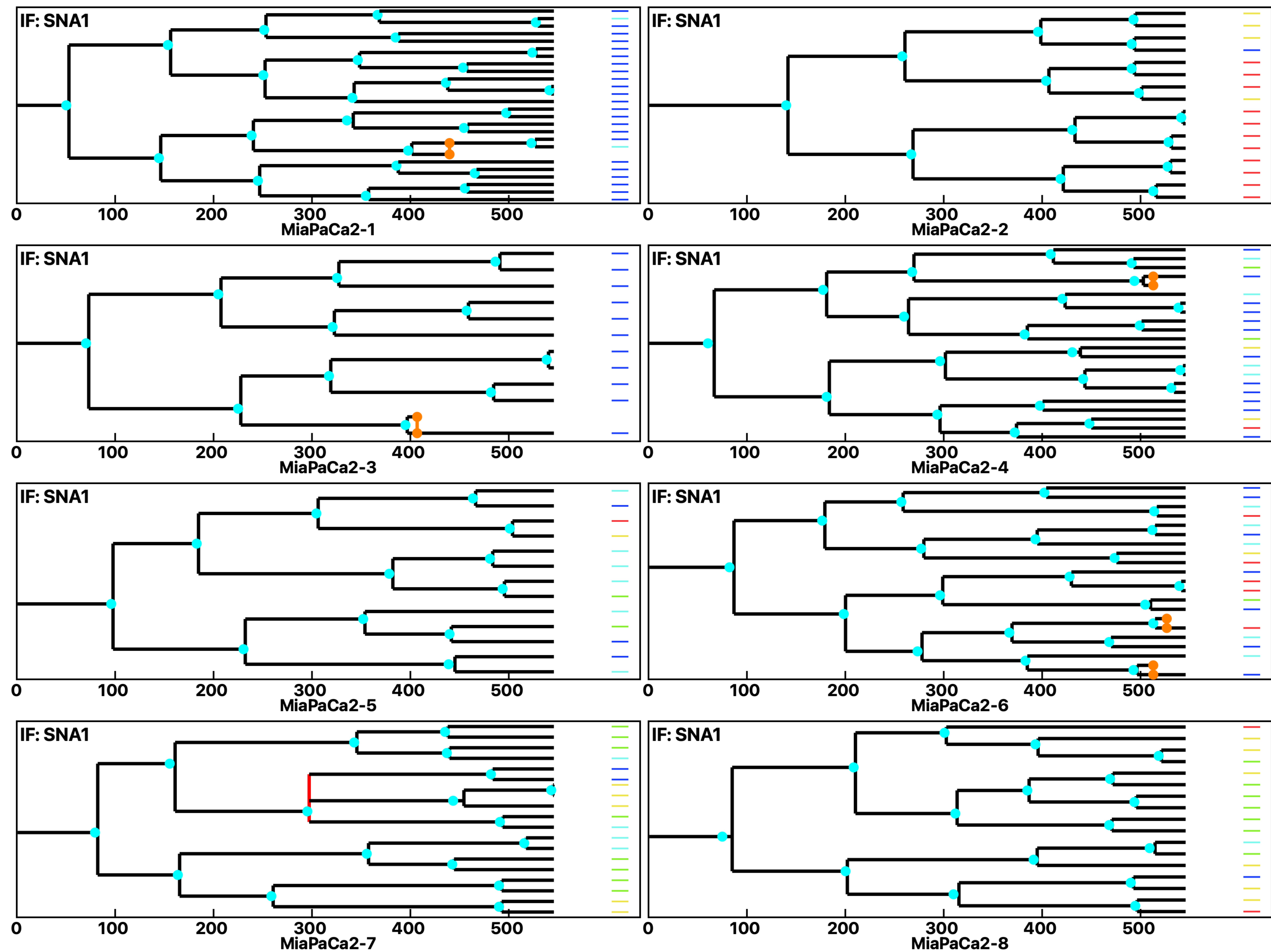

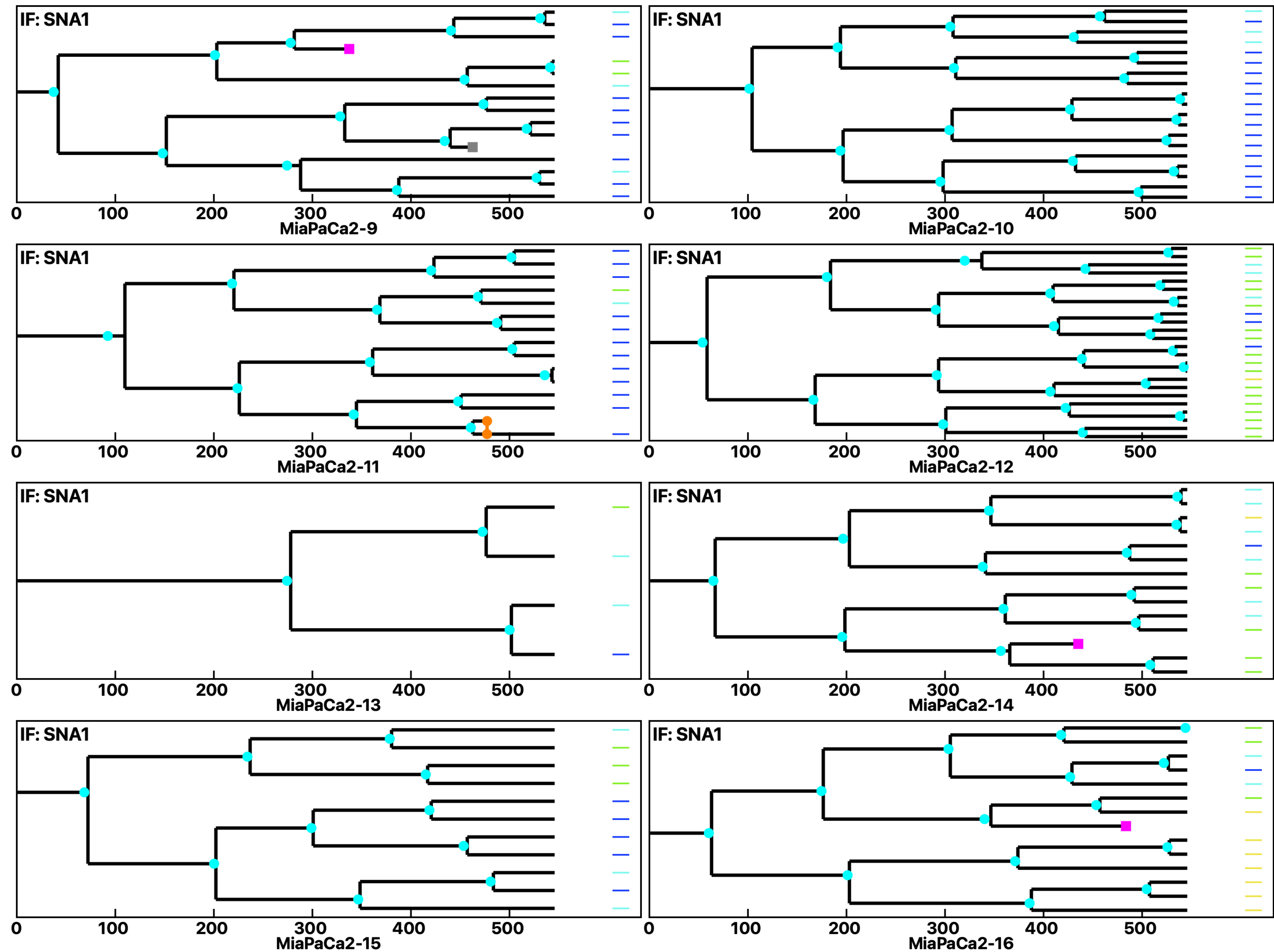

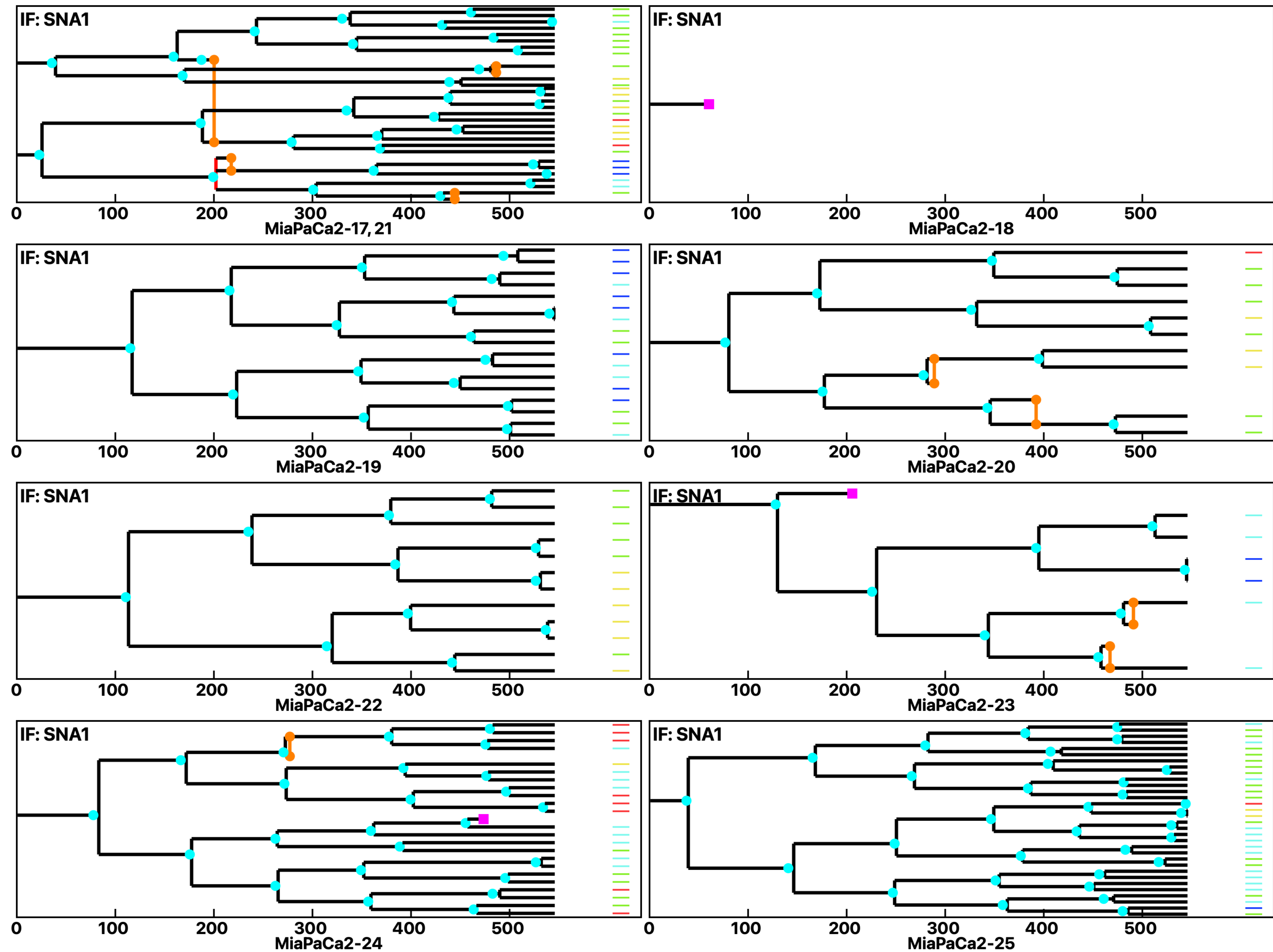

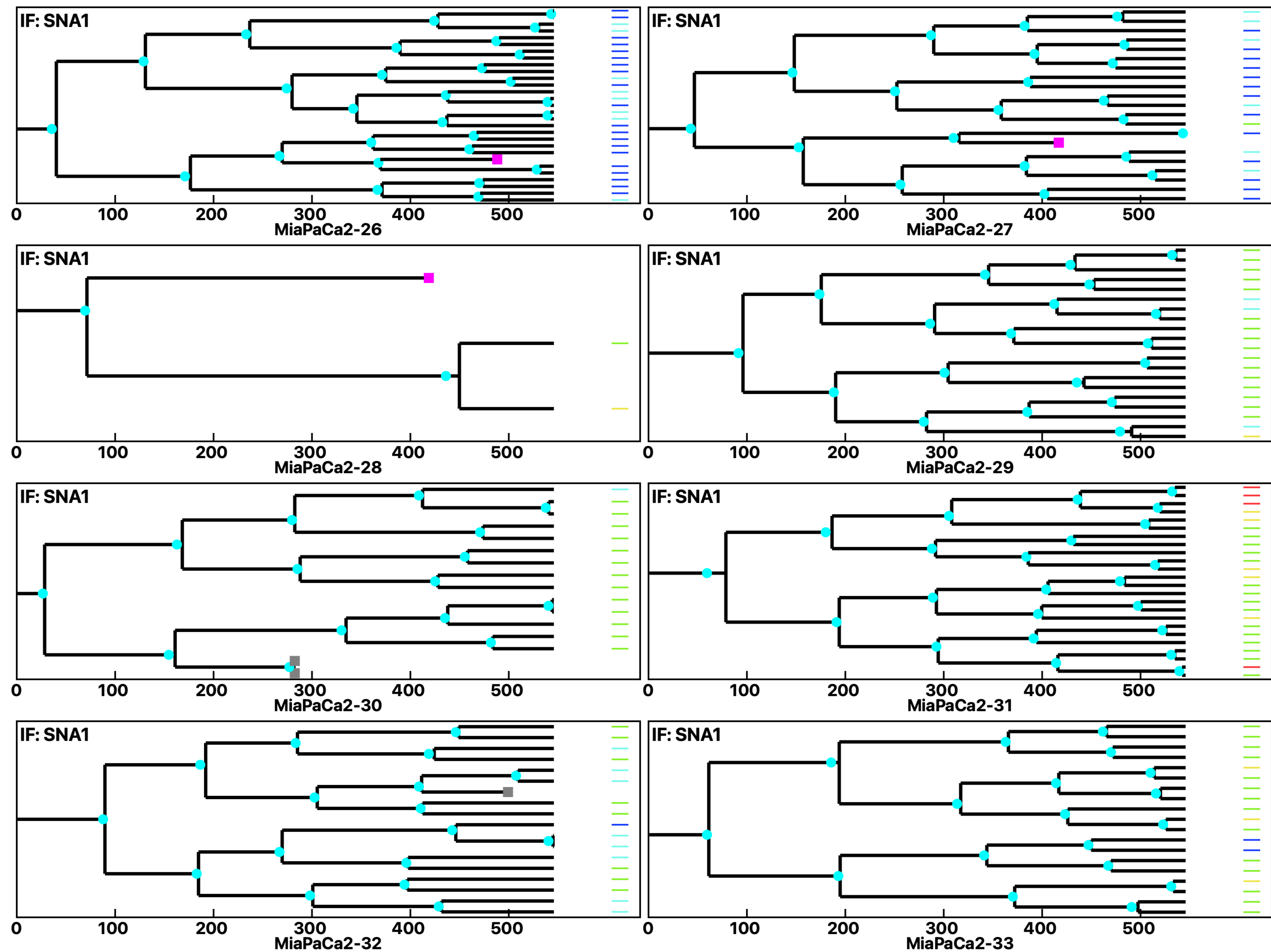

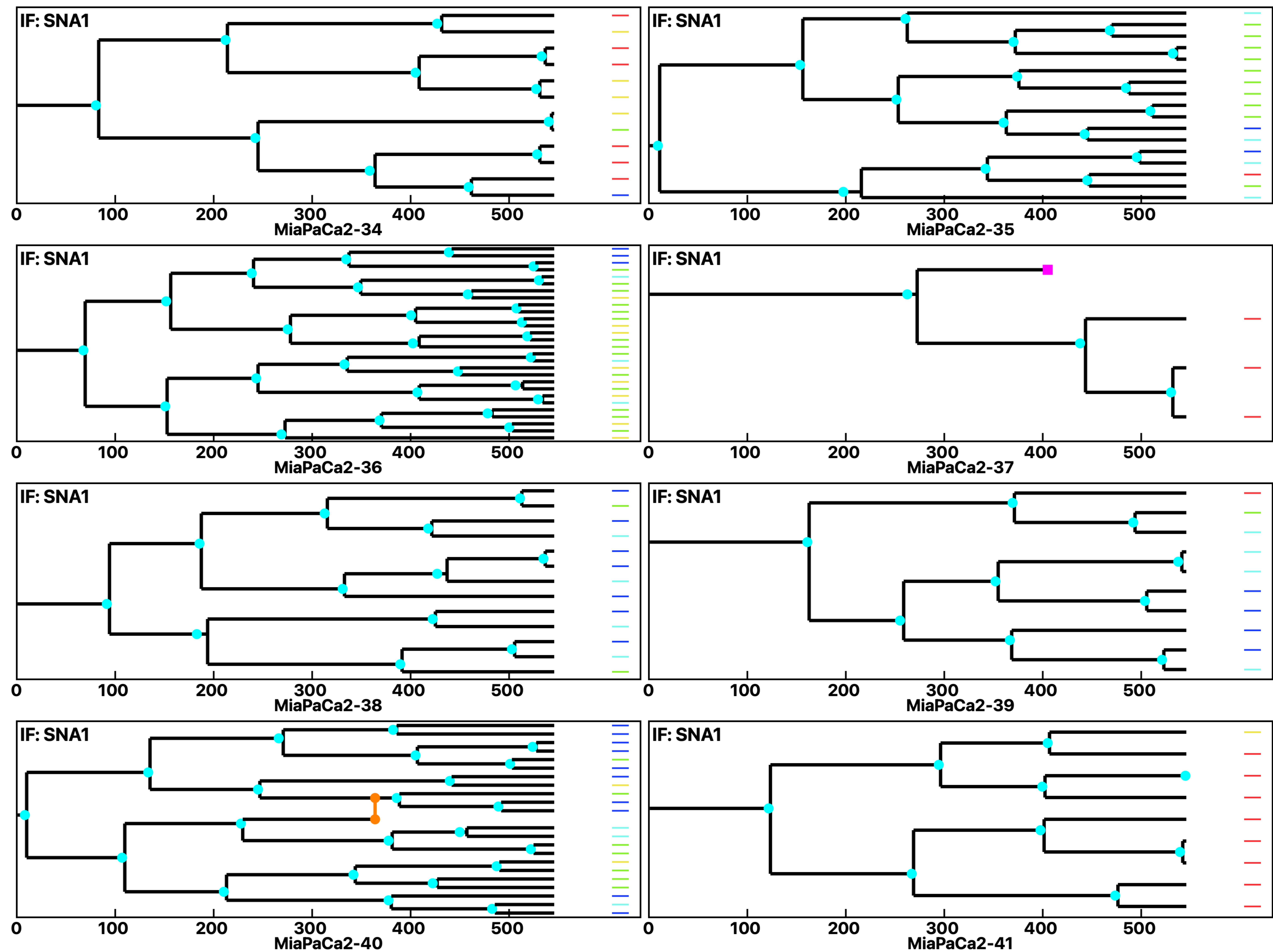

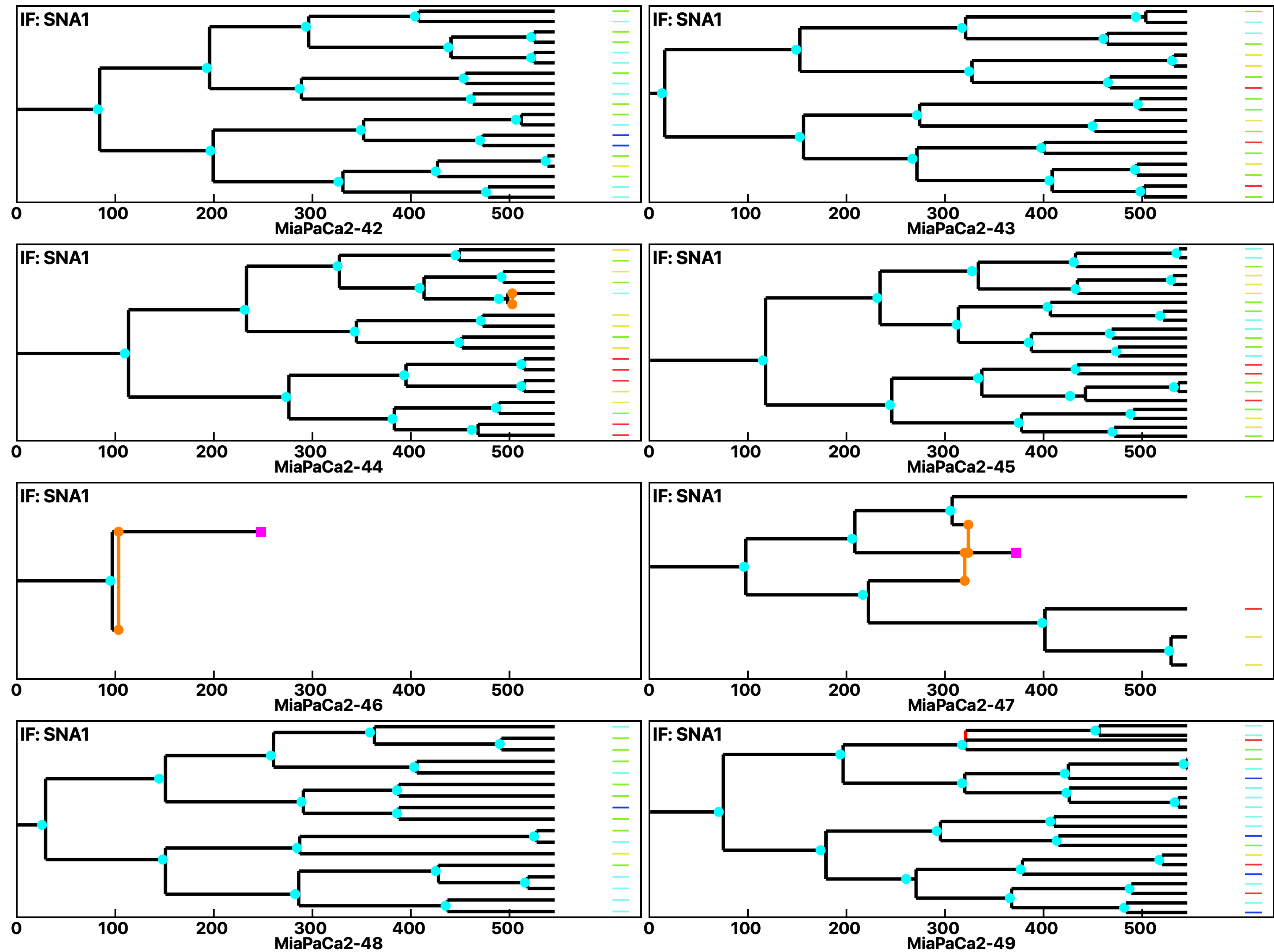

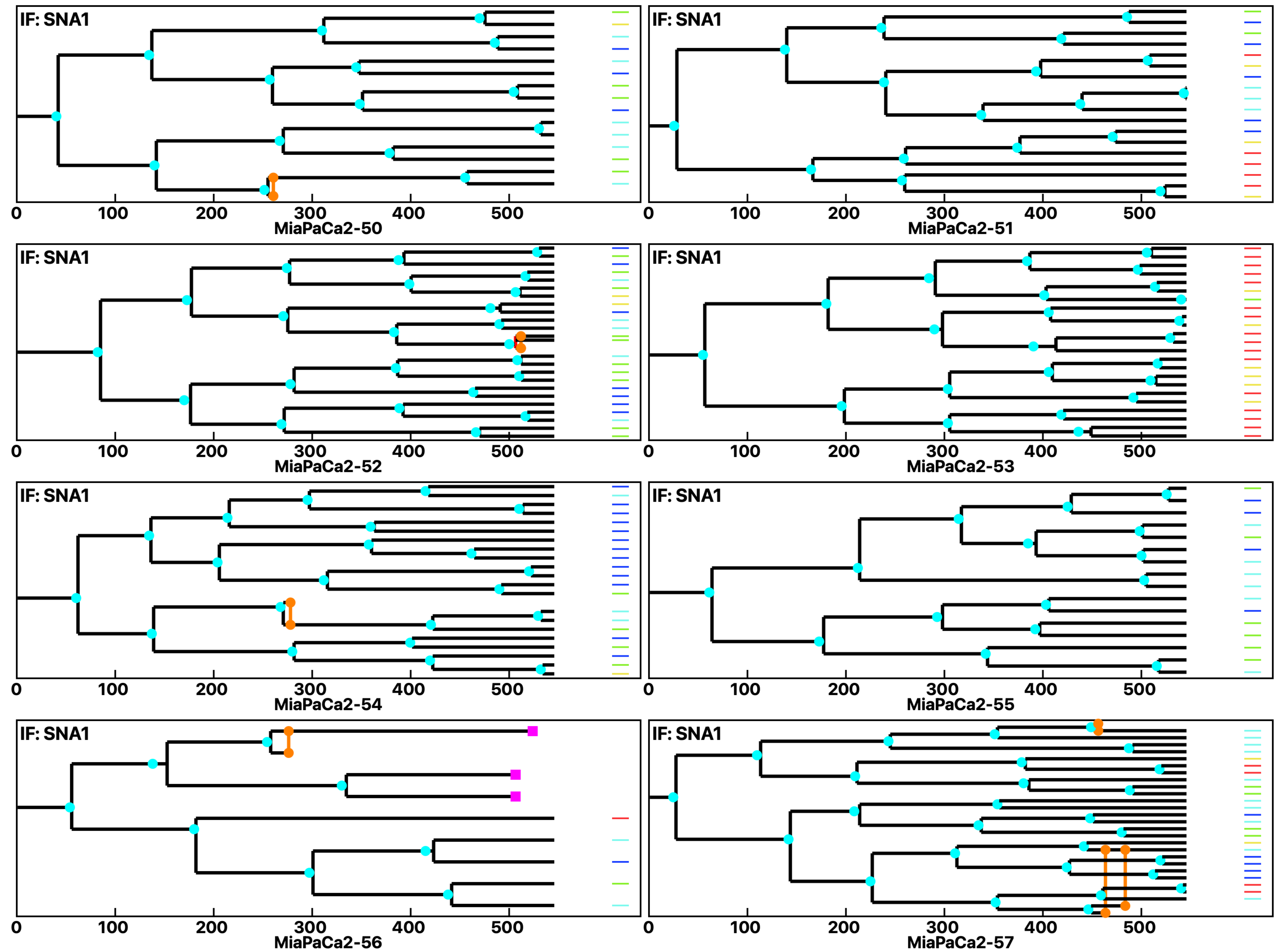

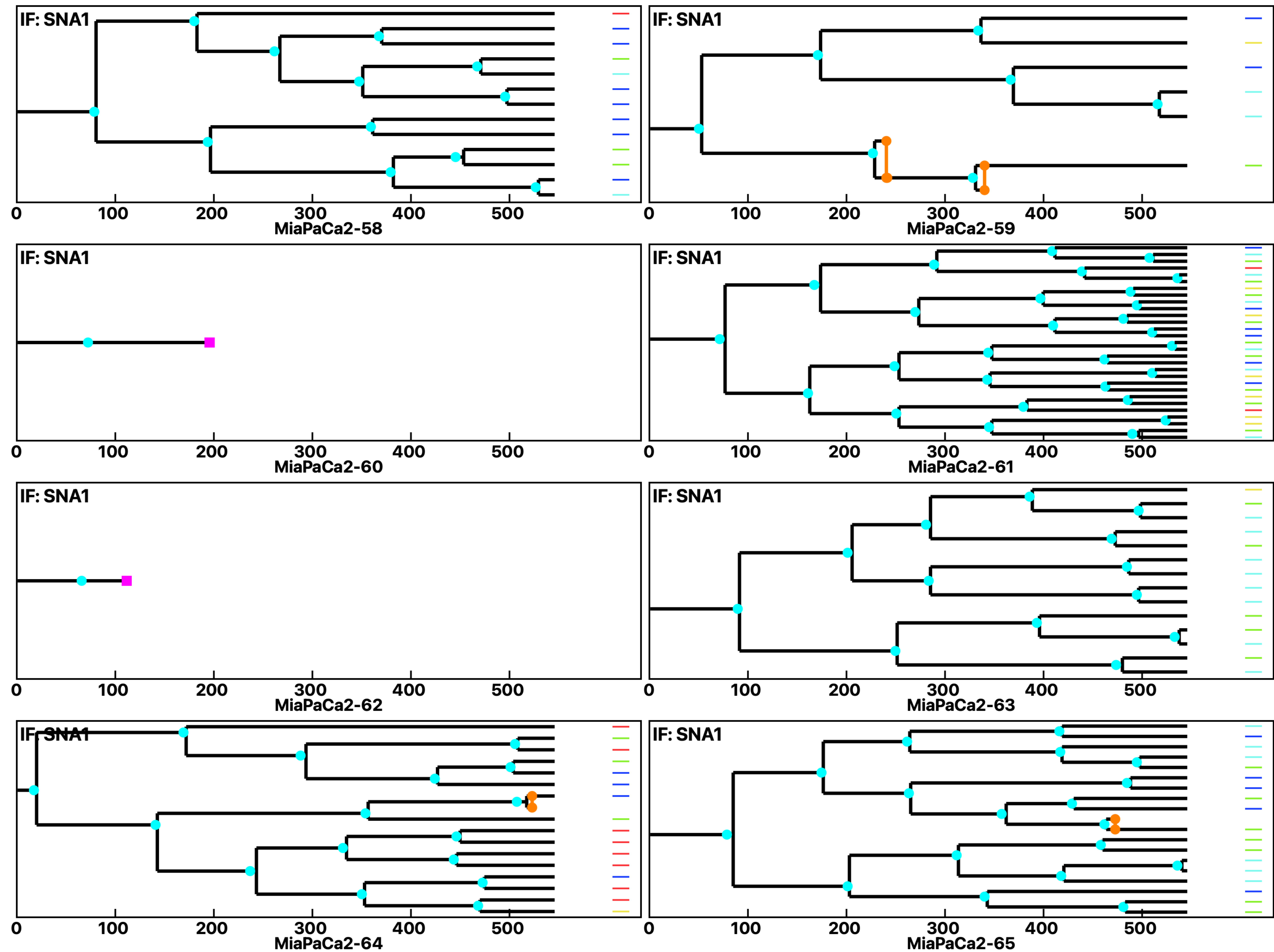

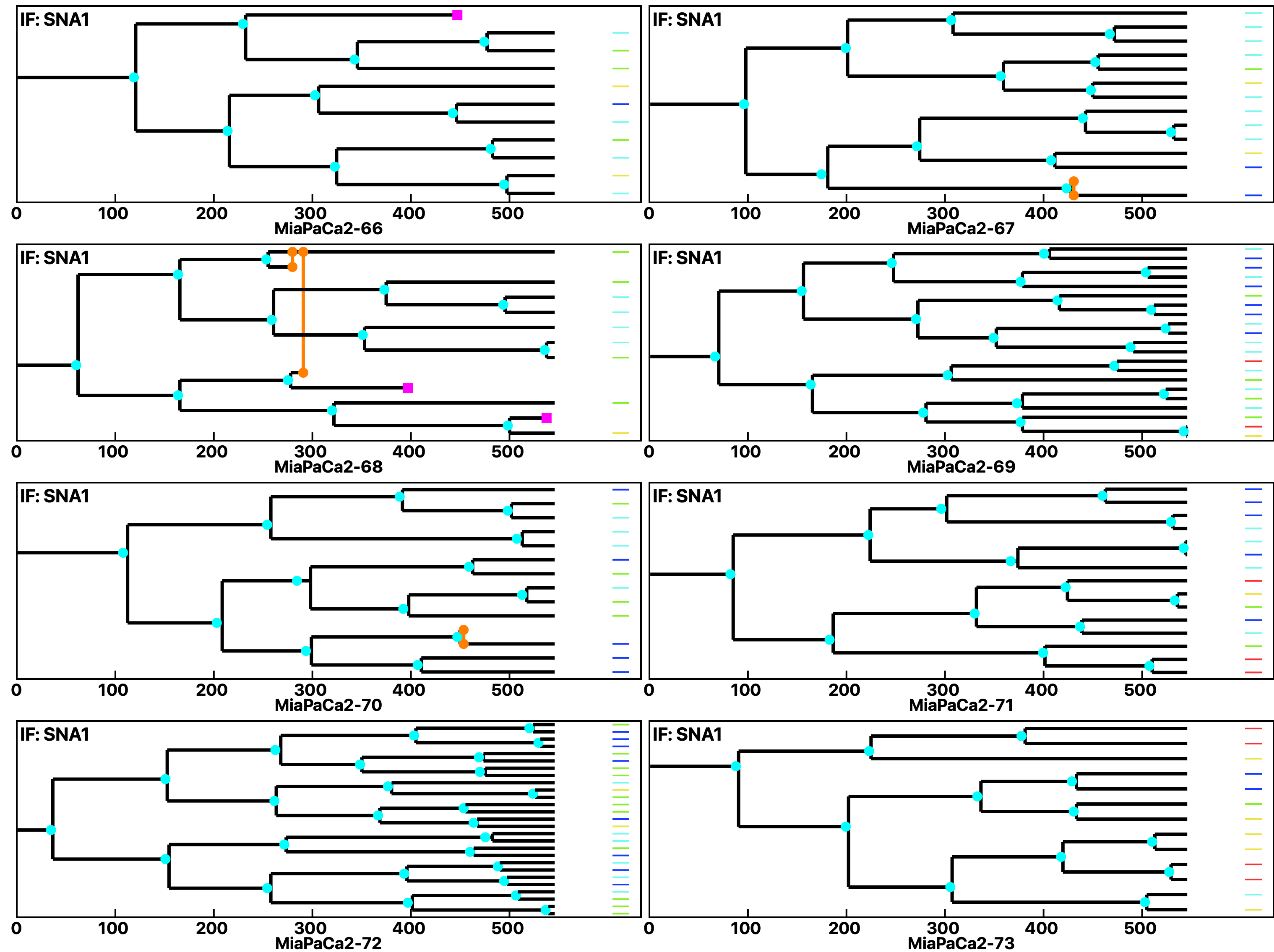

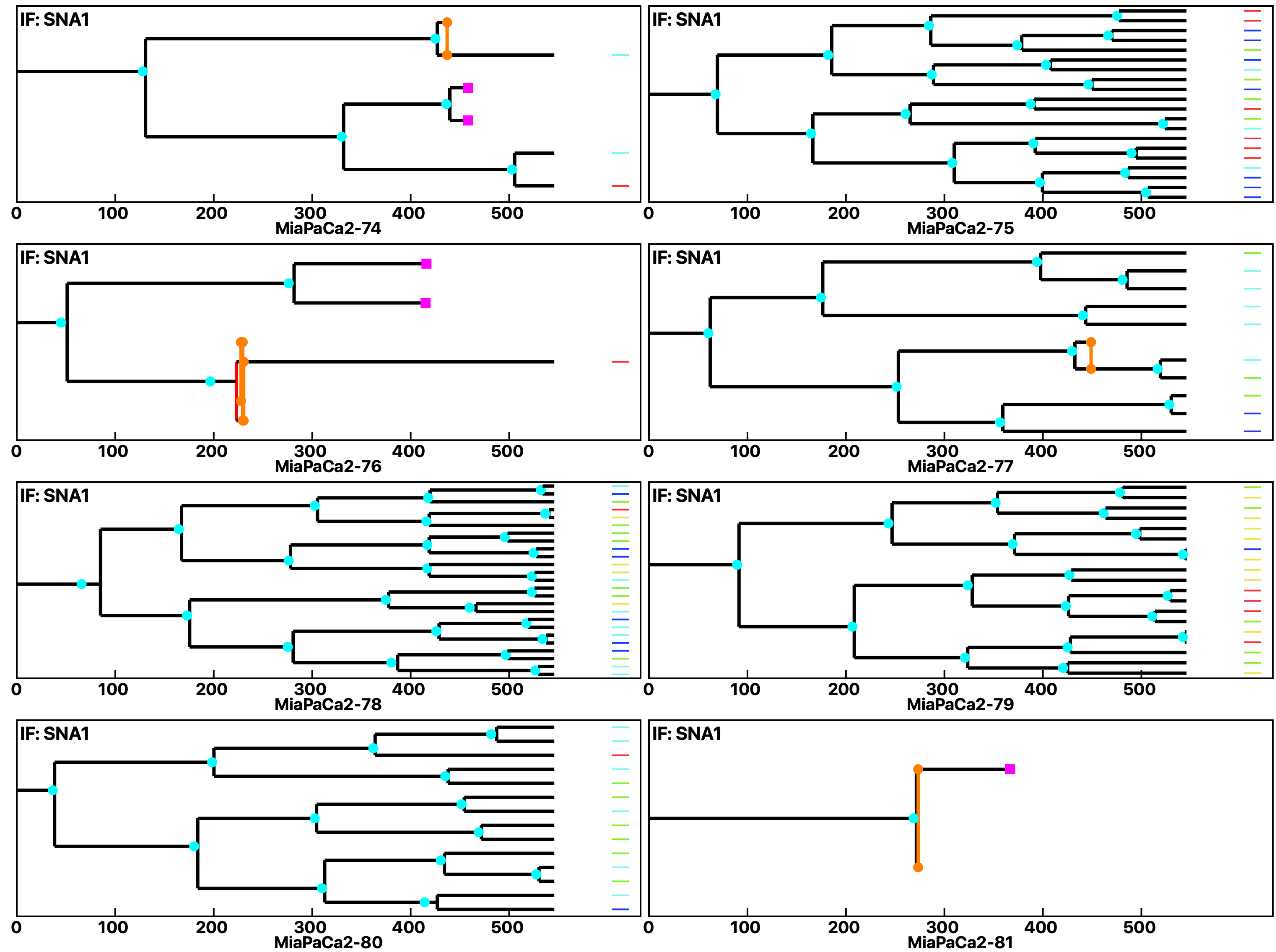

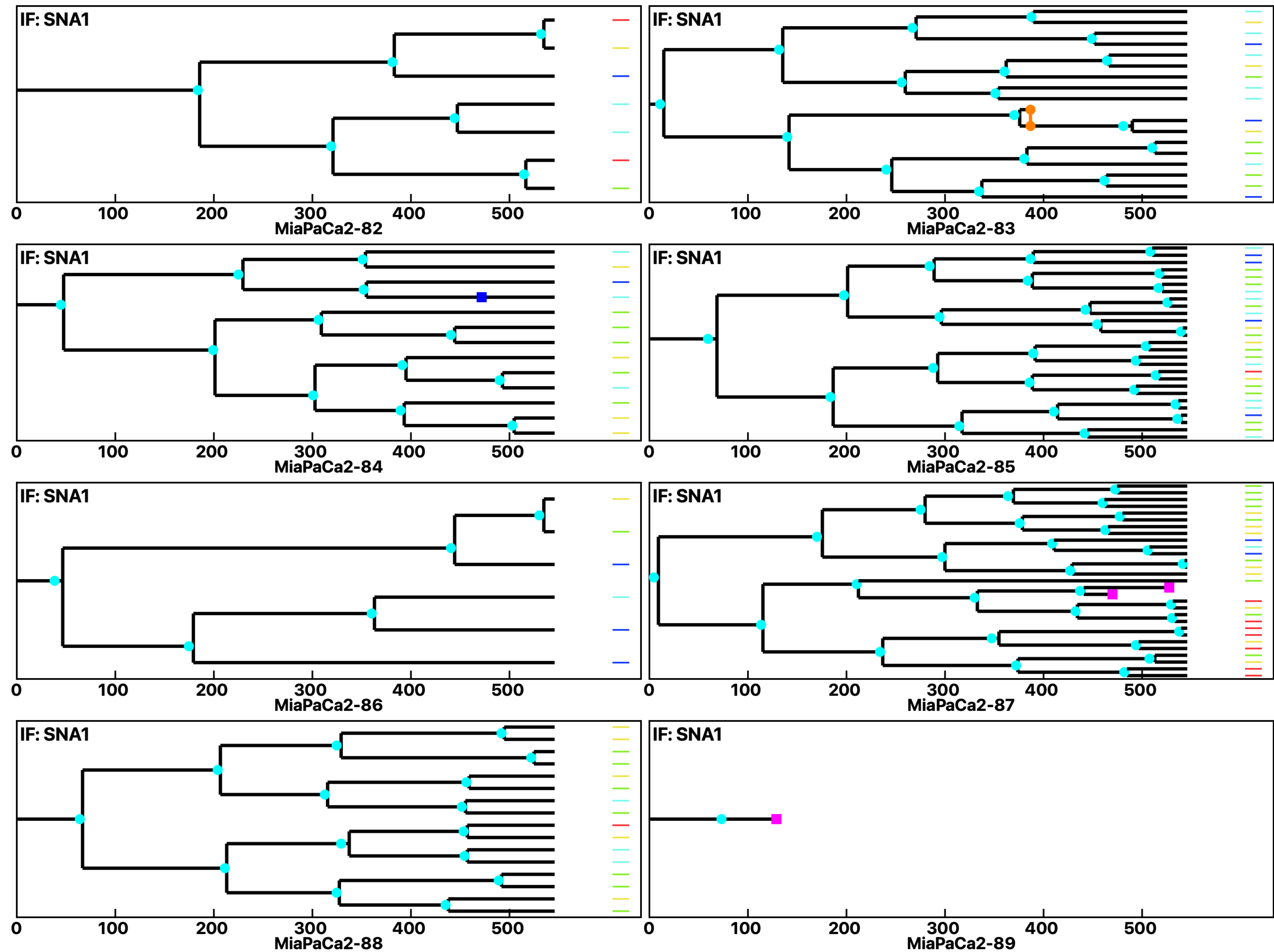

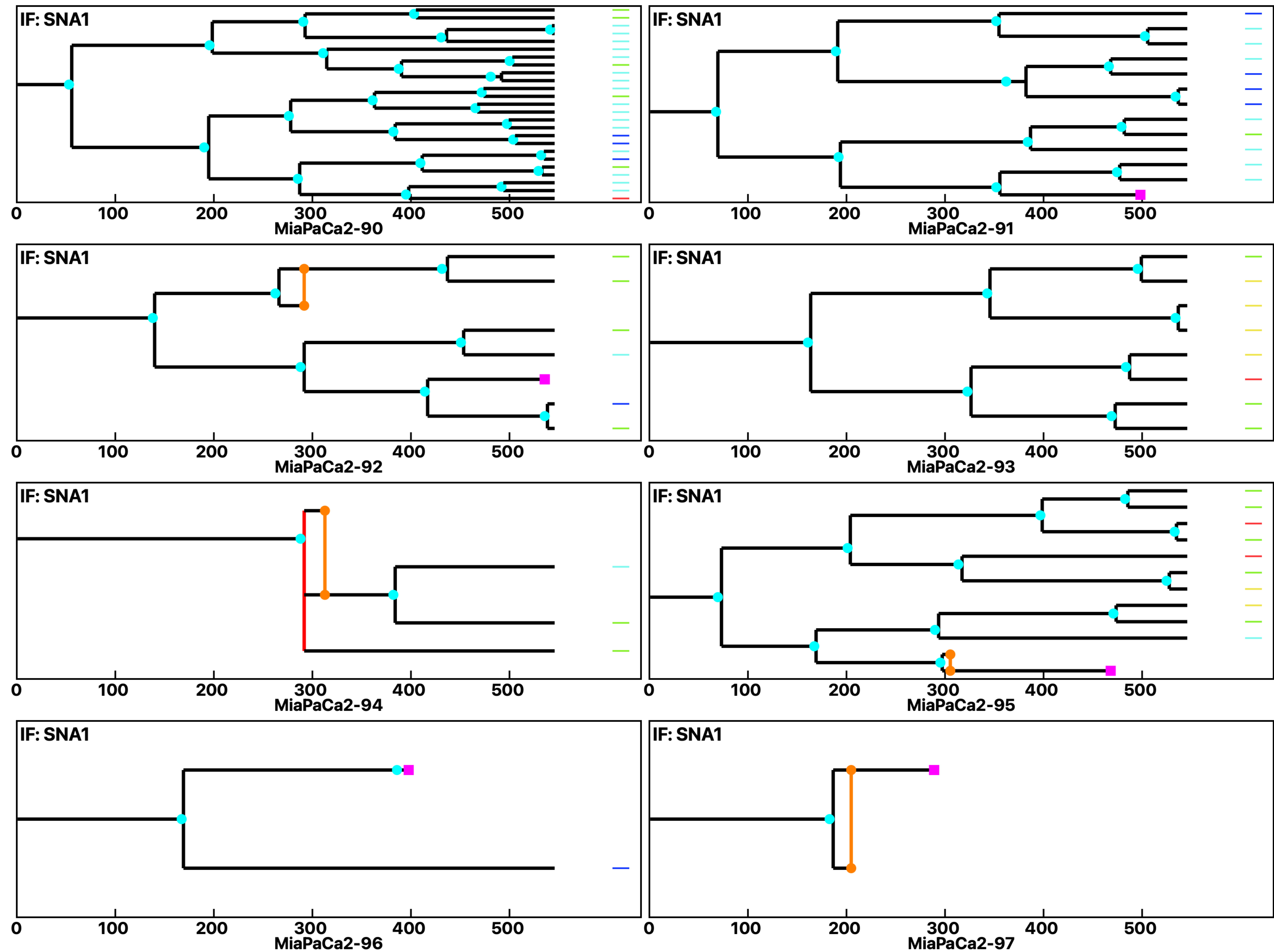

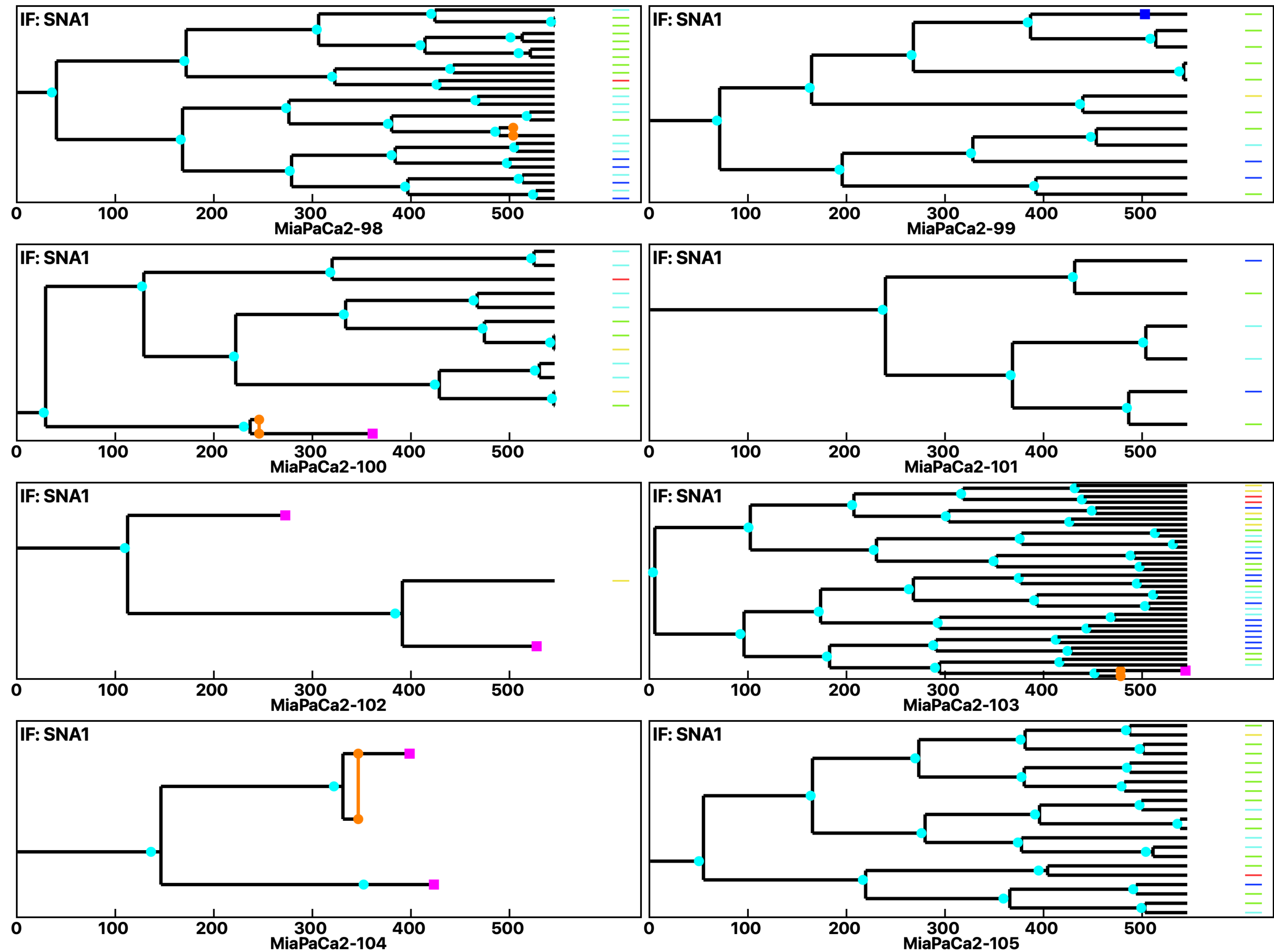

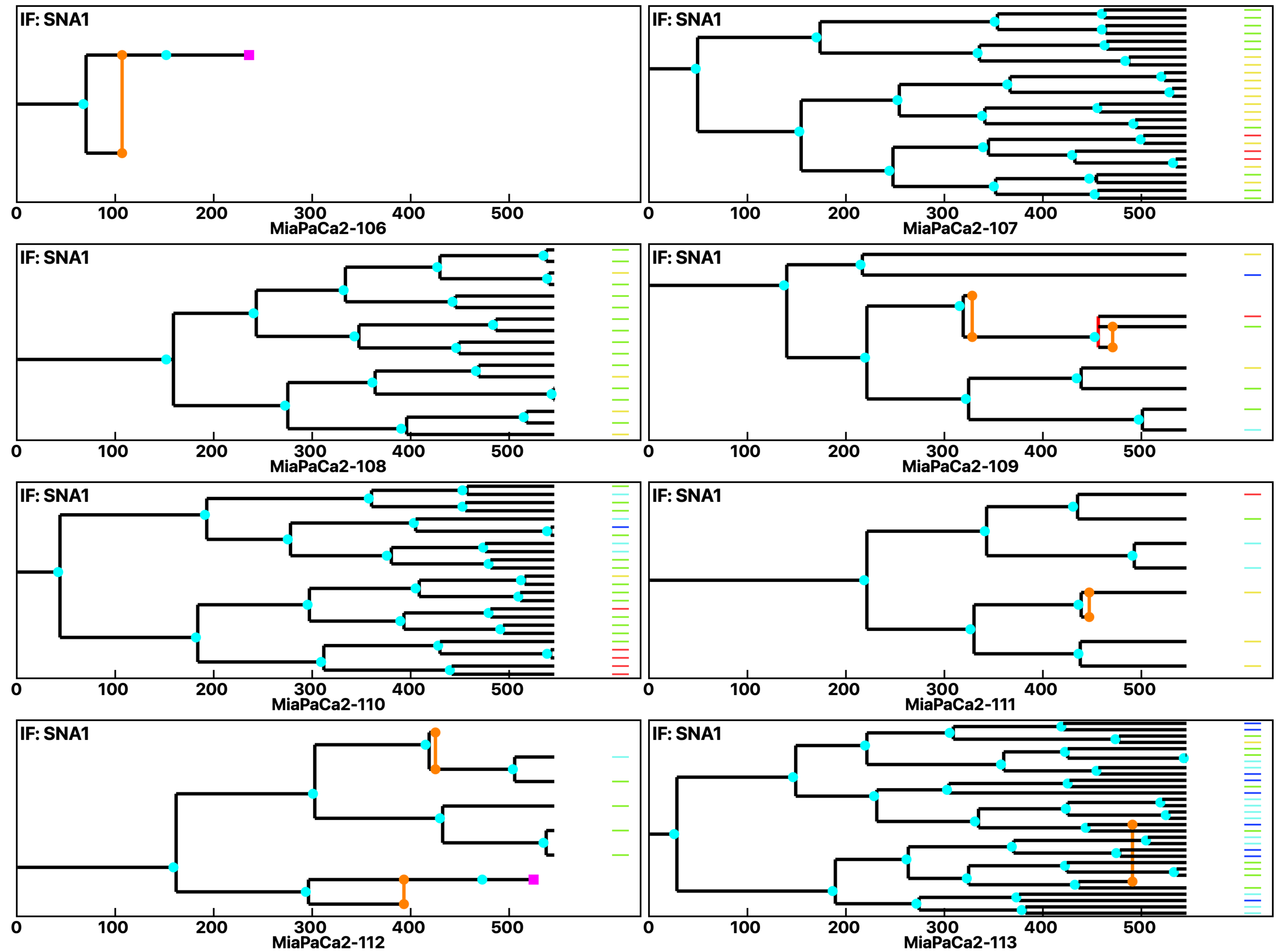

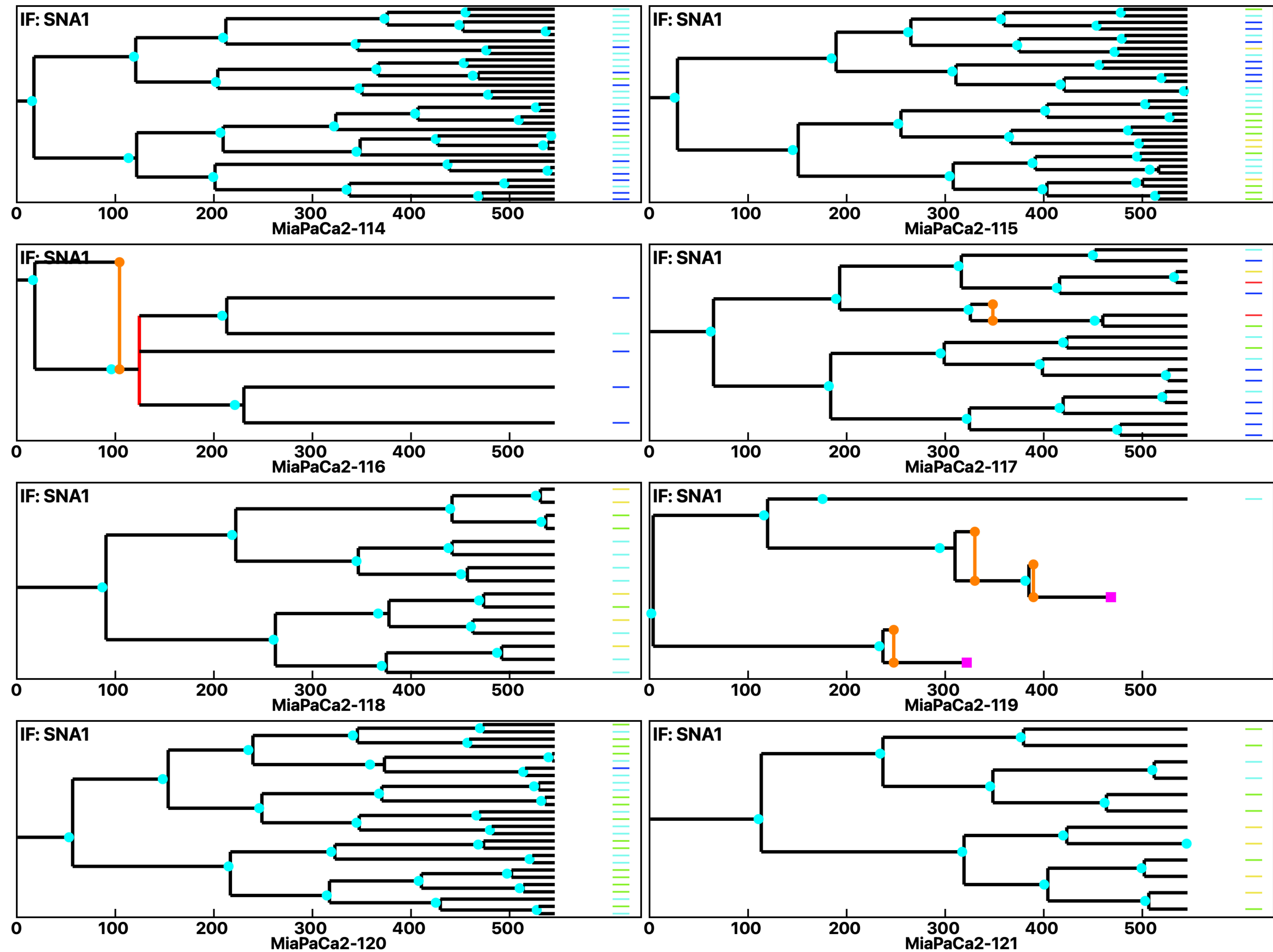

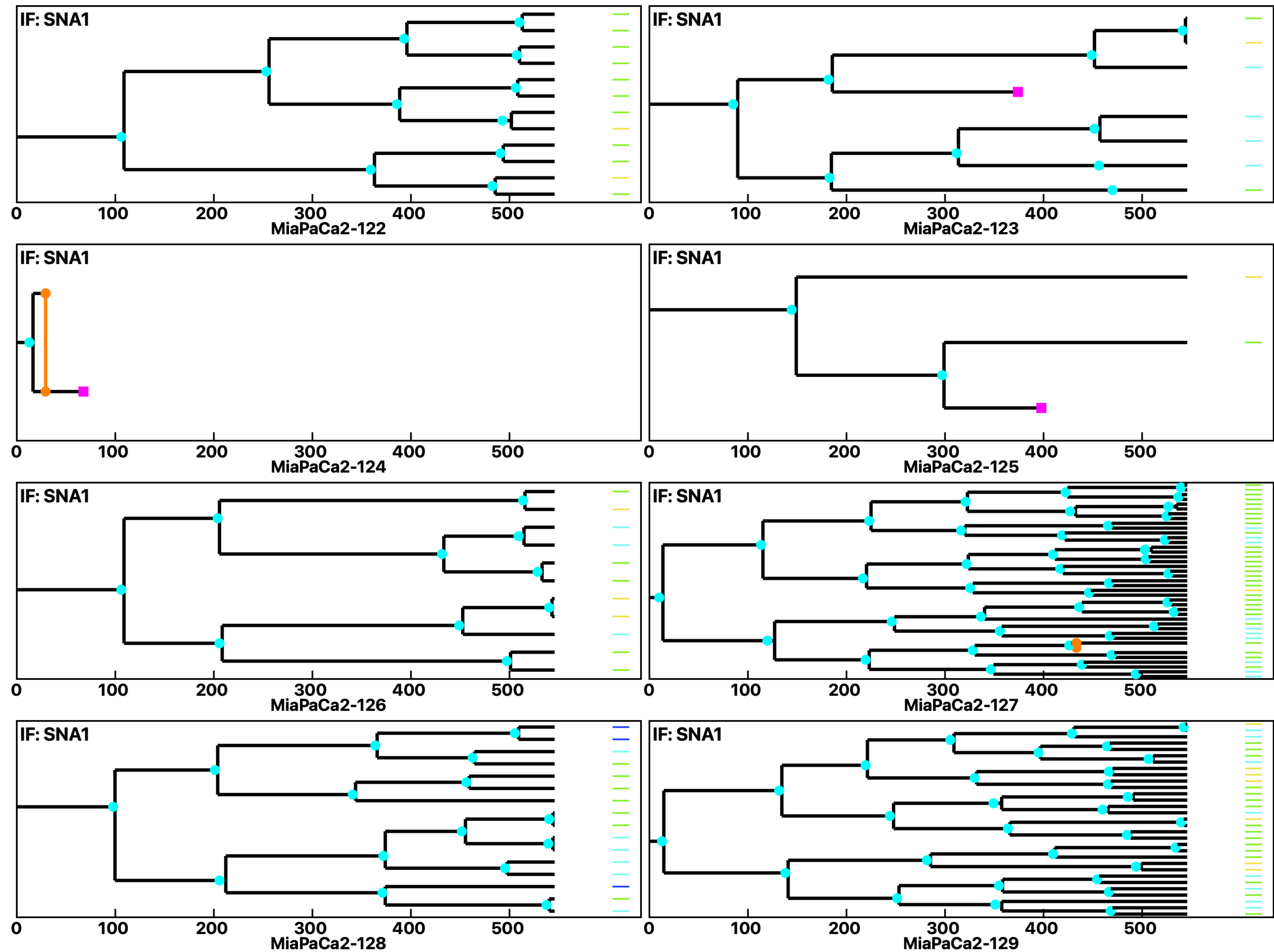

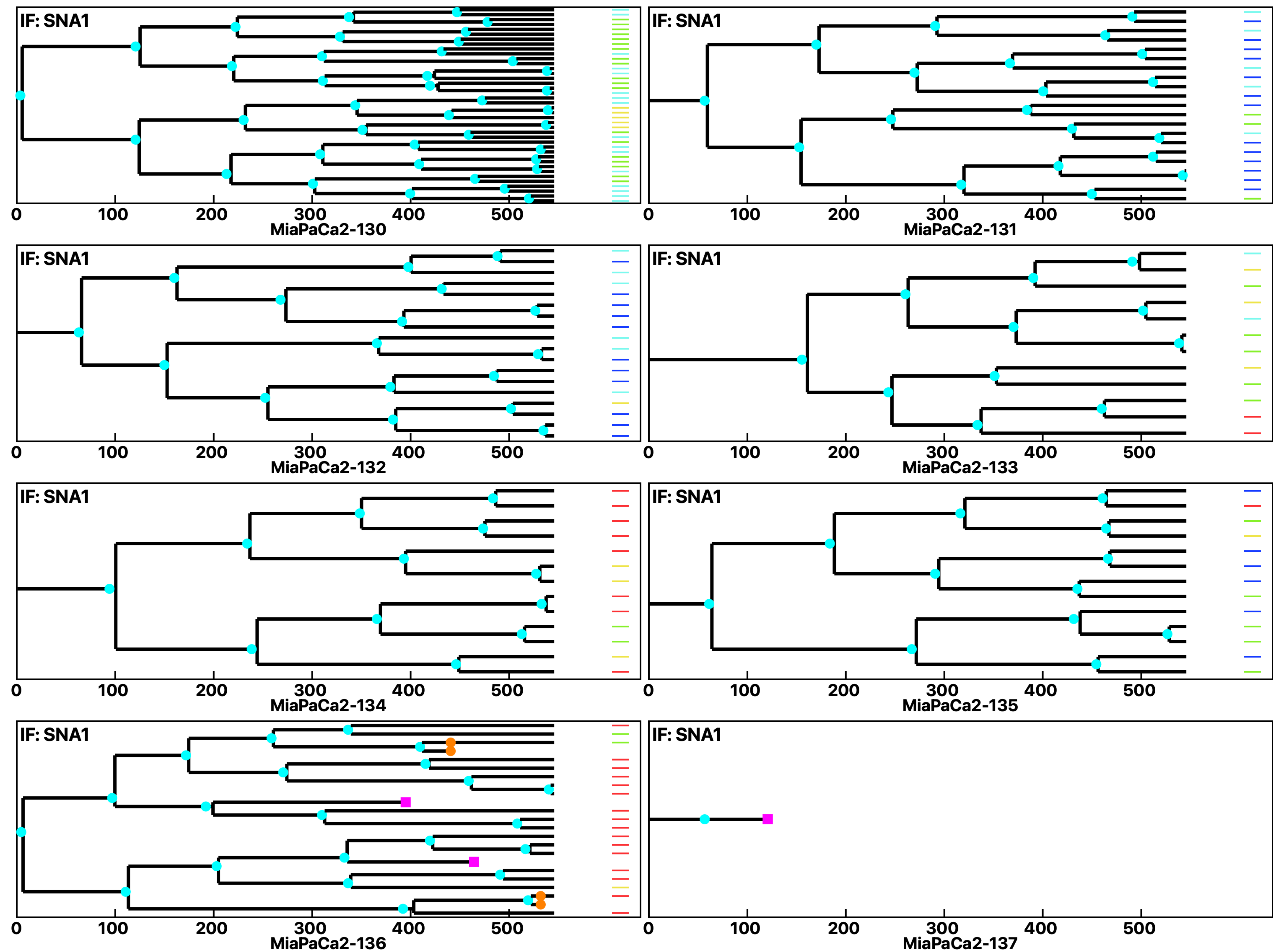

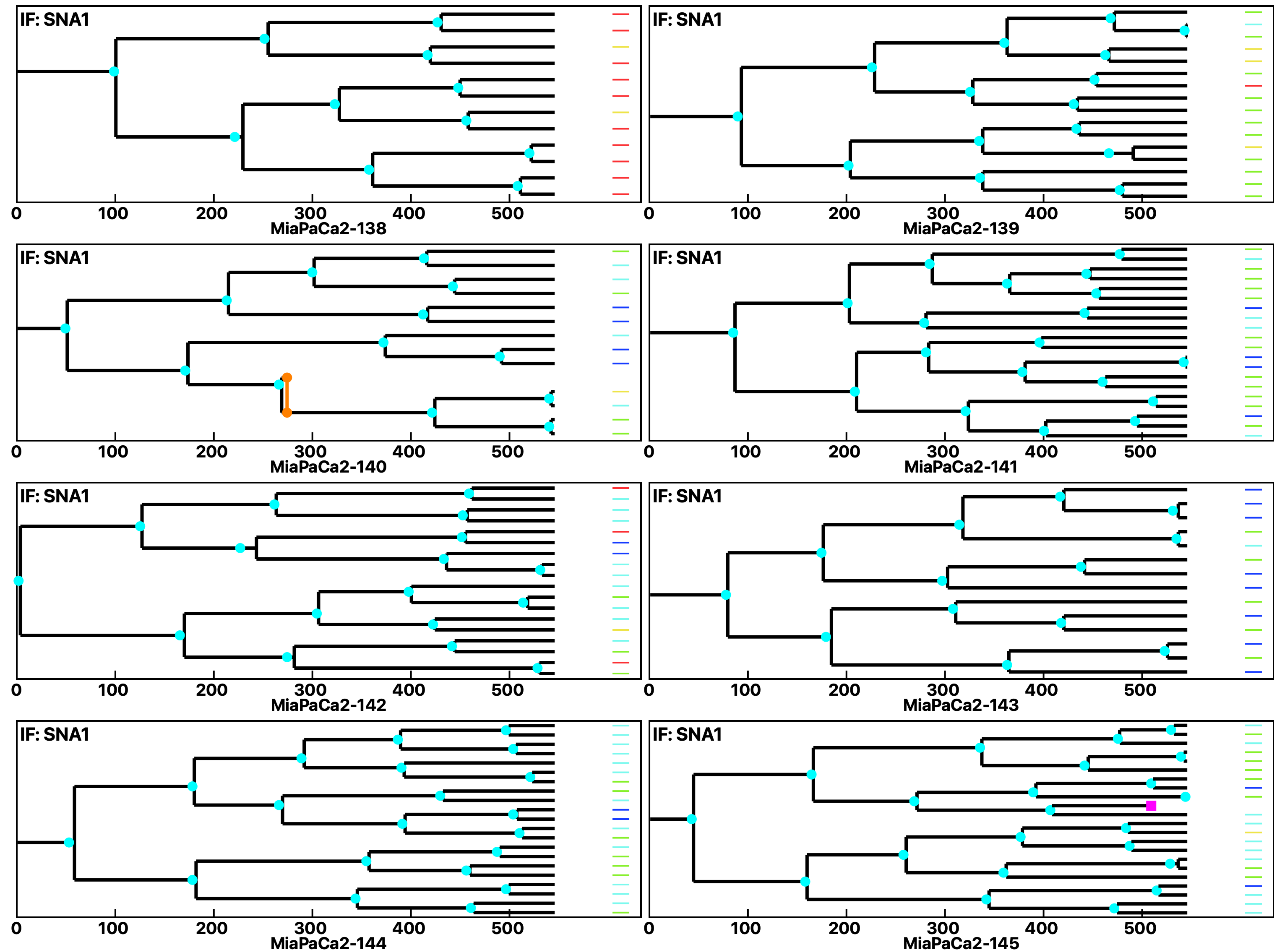

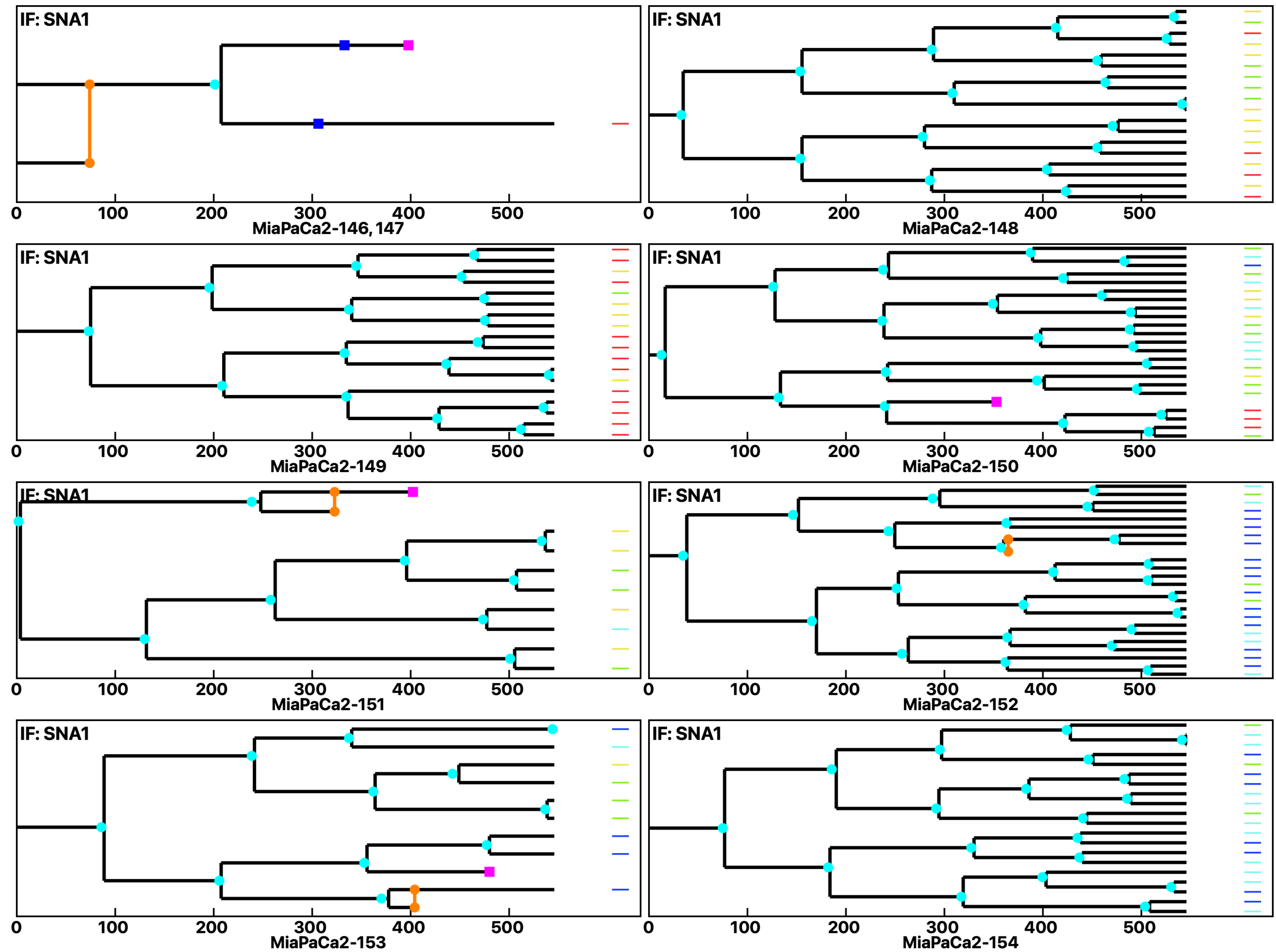

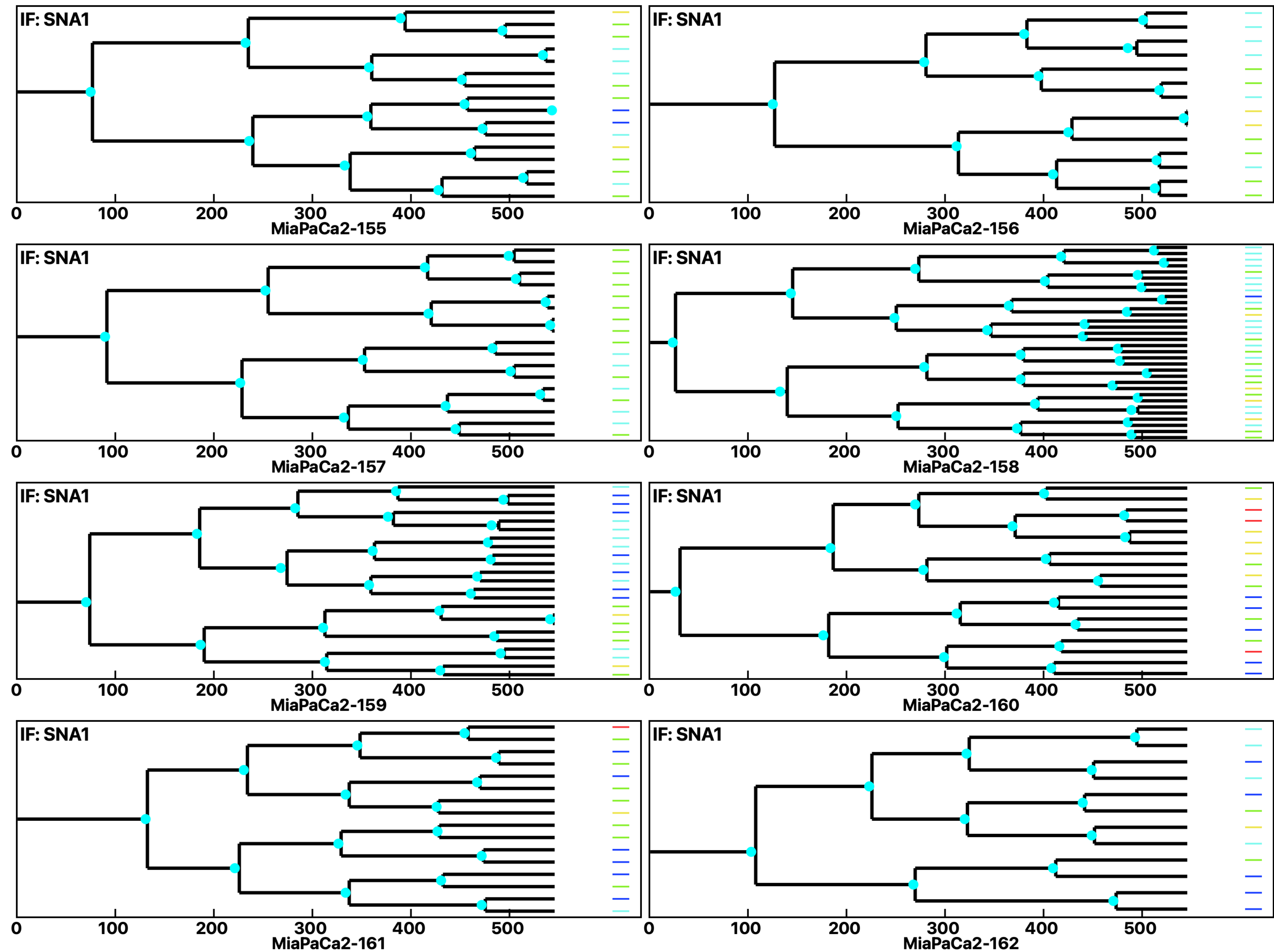

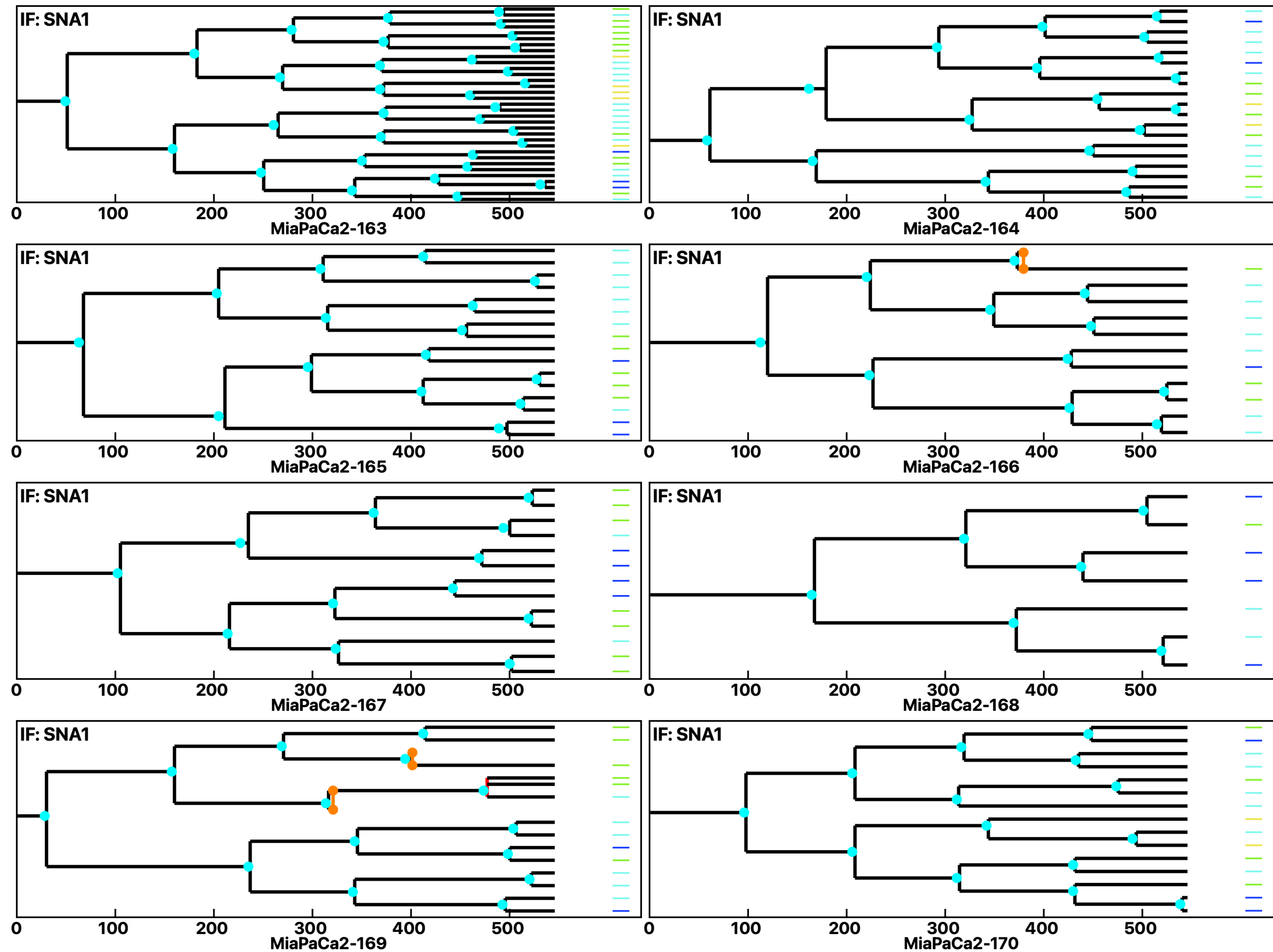

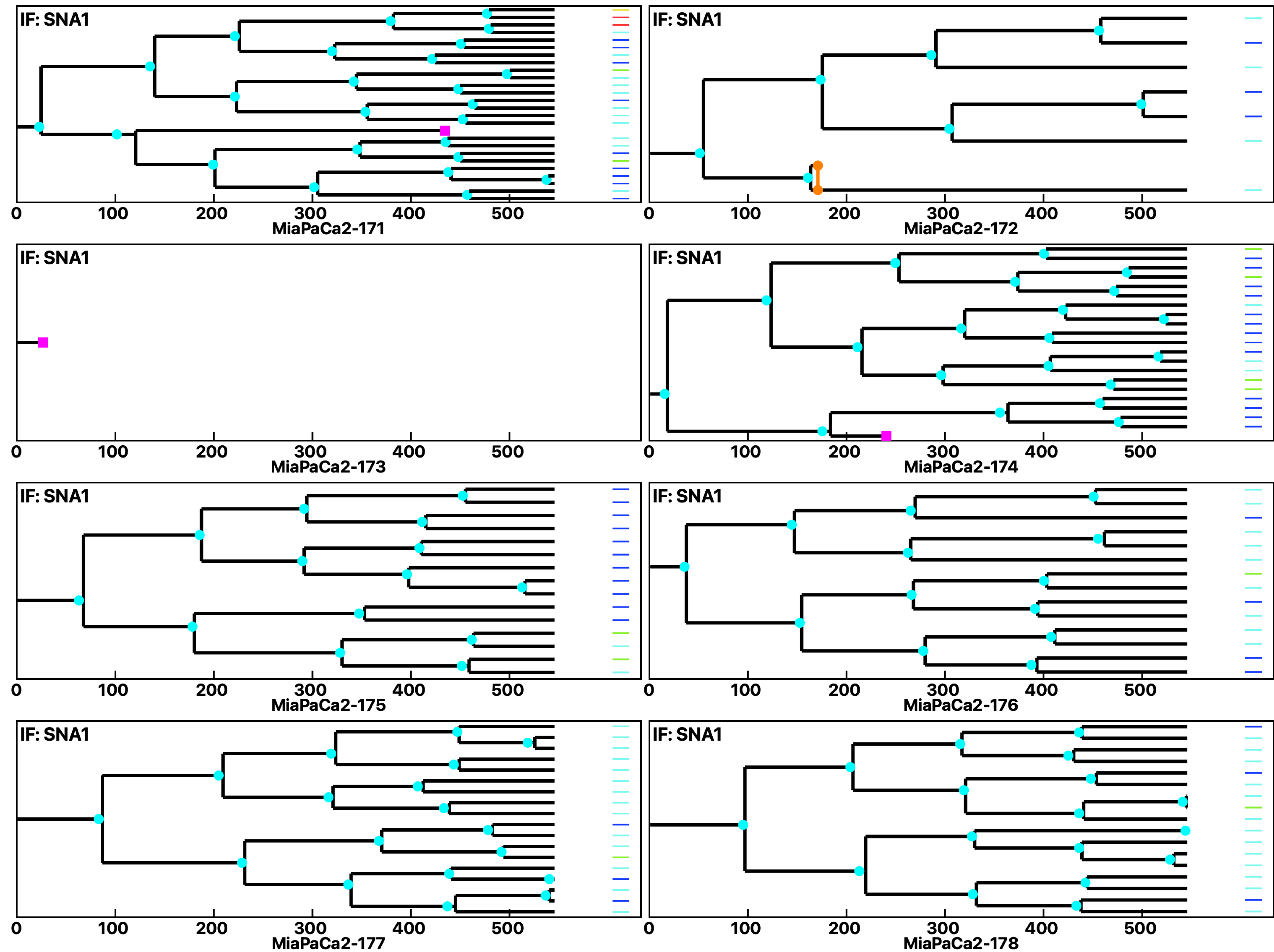

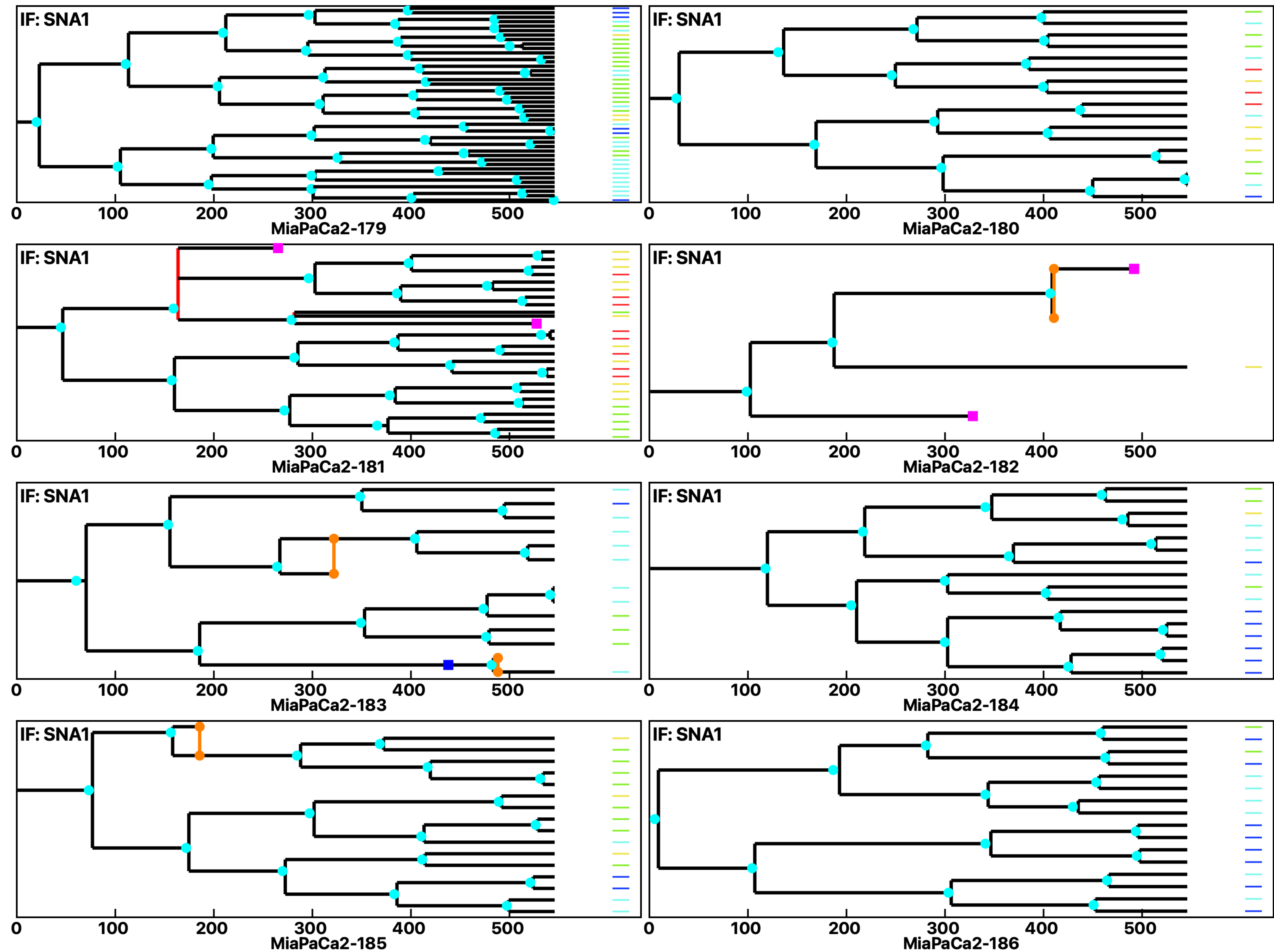

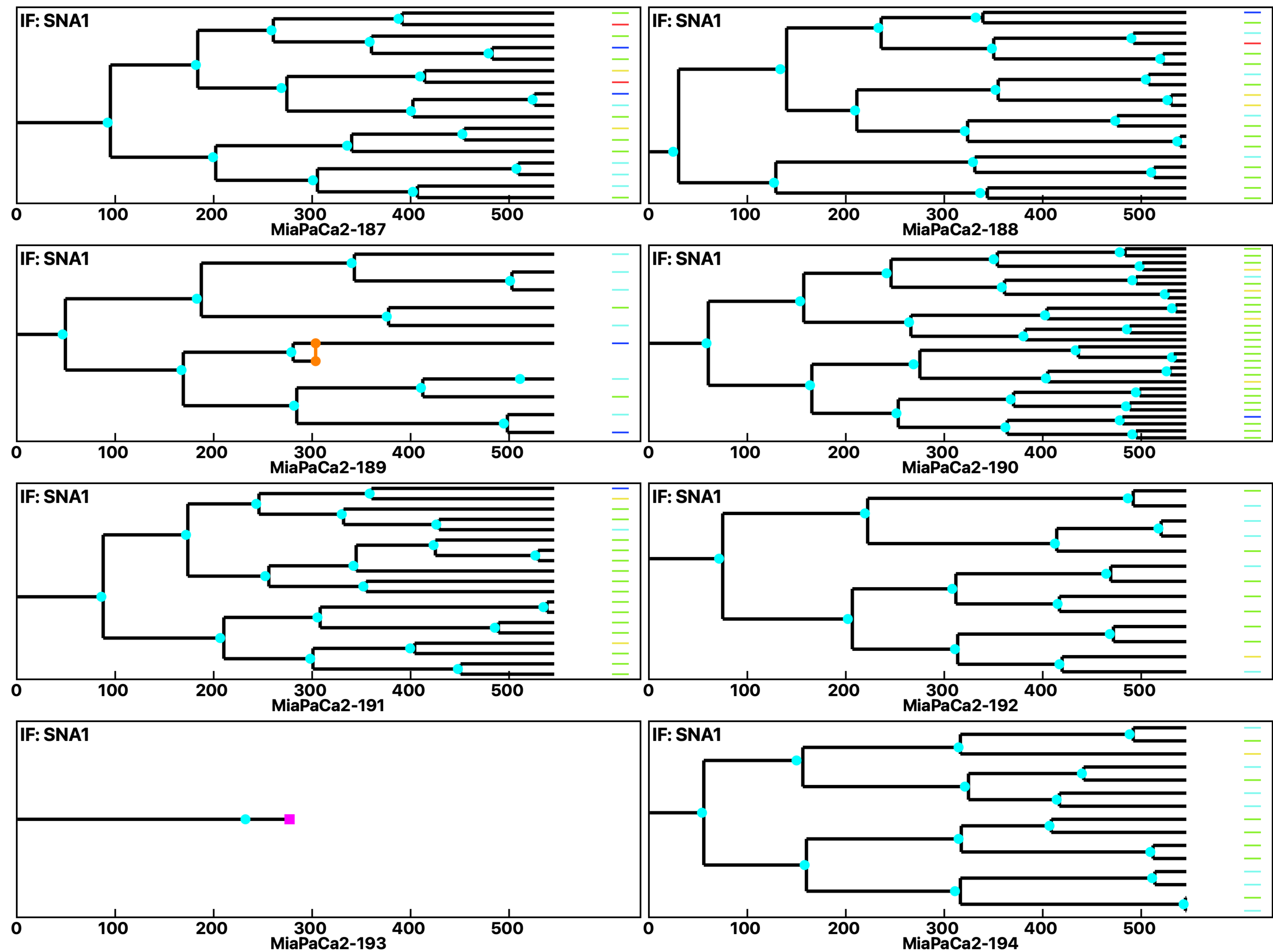

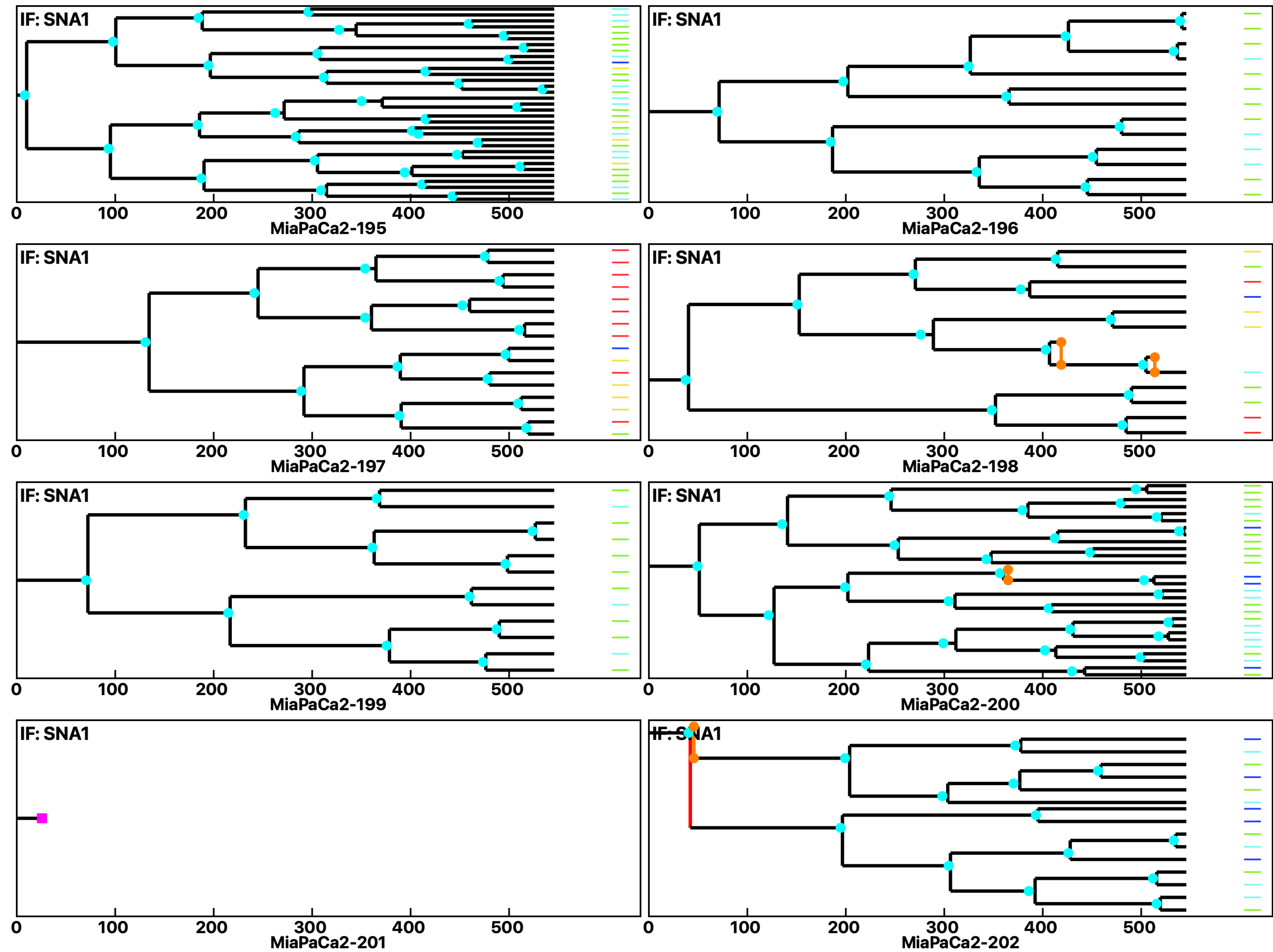

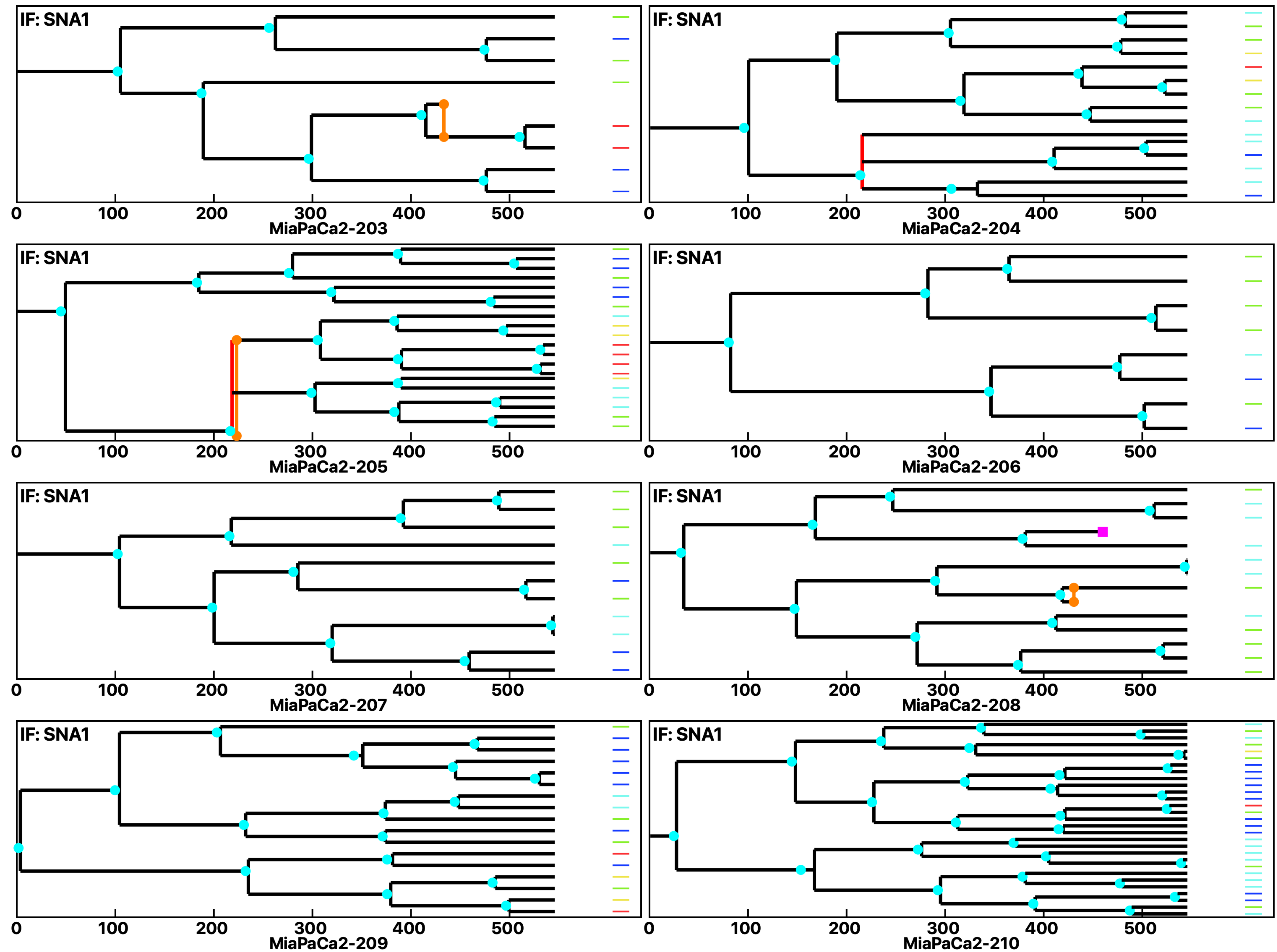

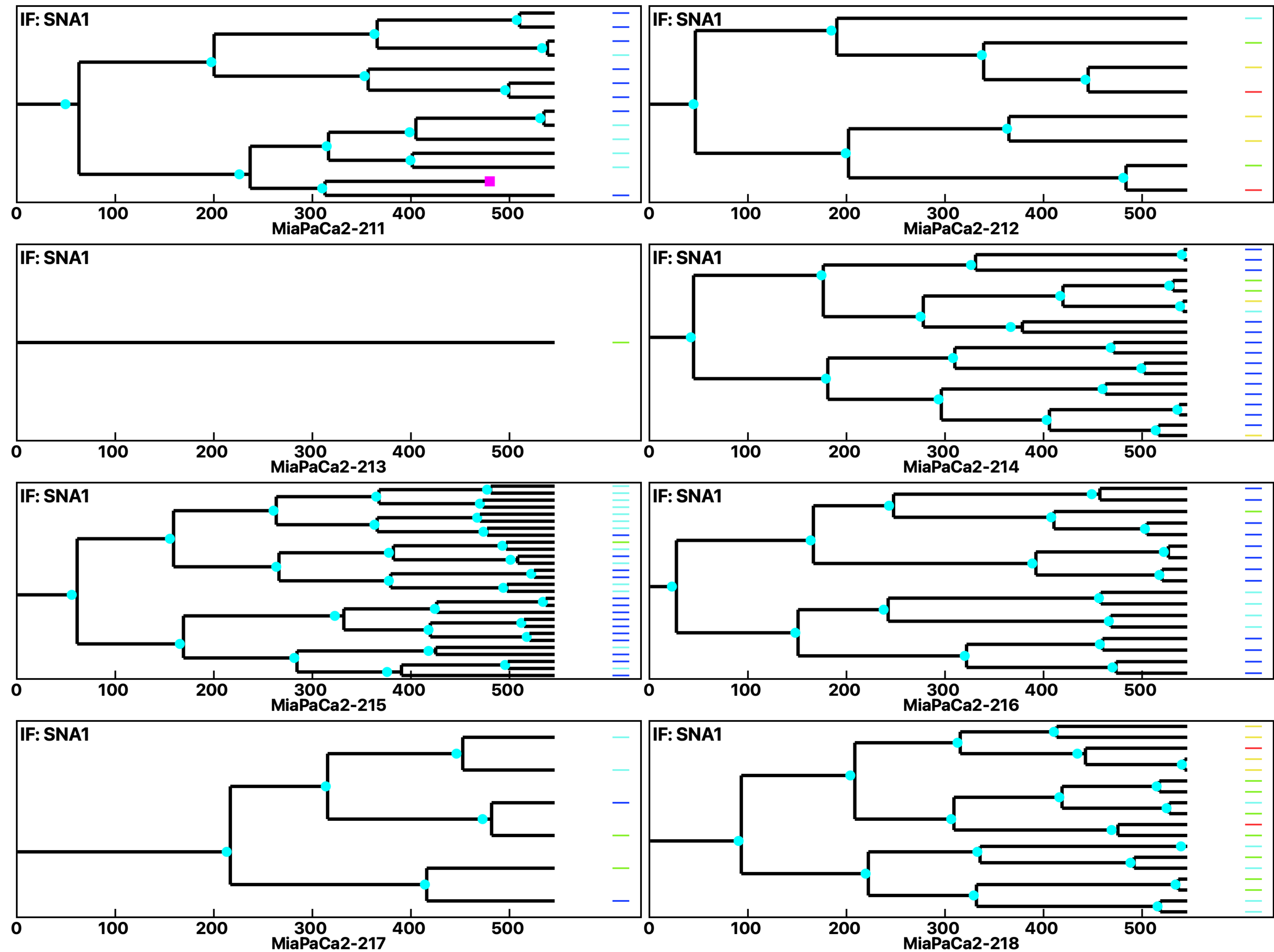

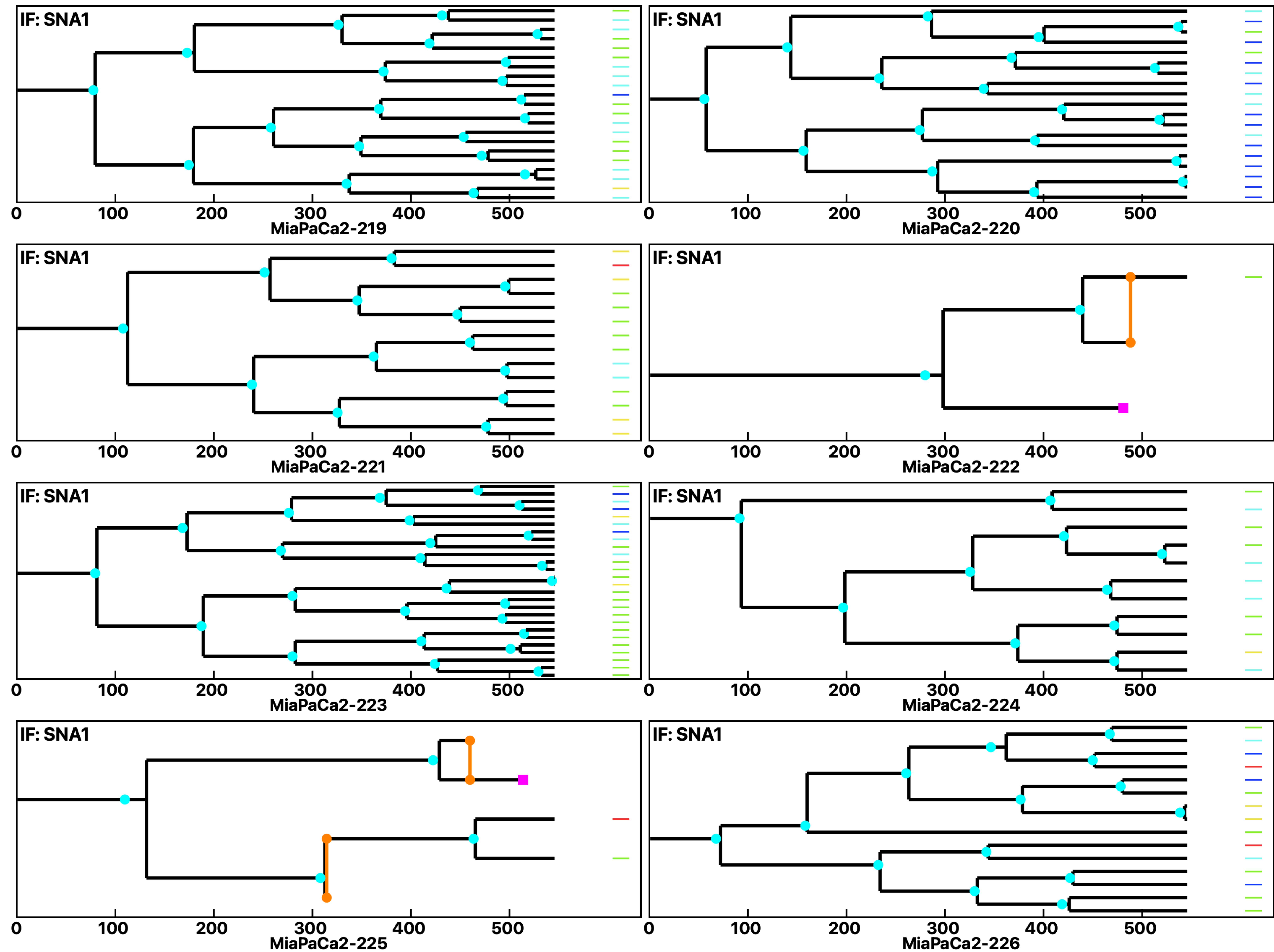

**Analysis: MiaPaCa2, Treat.: MiaPaCa2, Cell: MiaPaCa2**

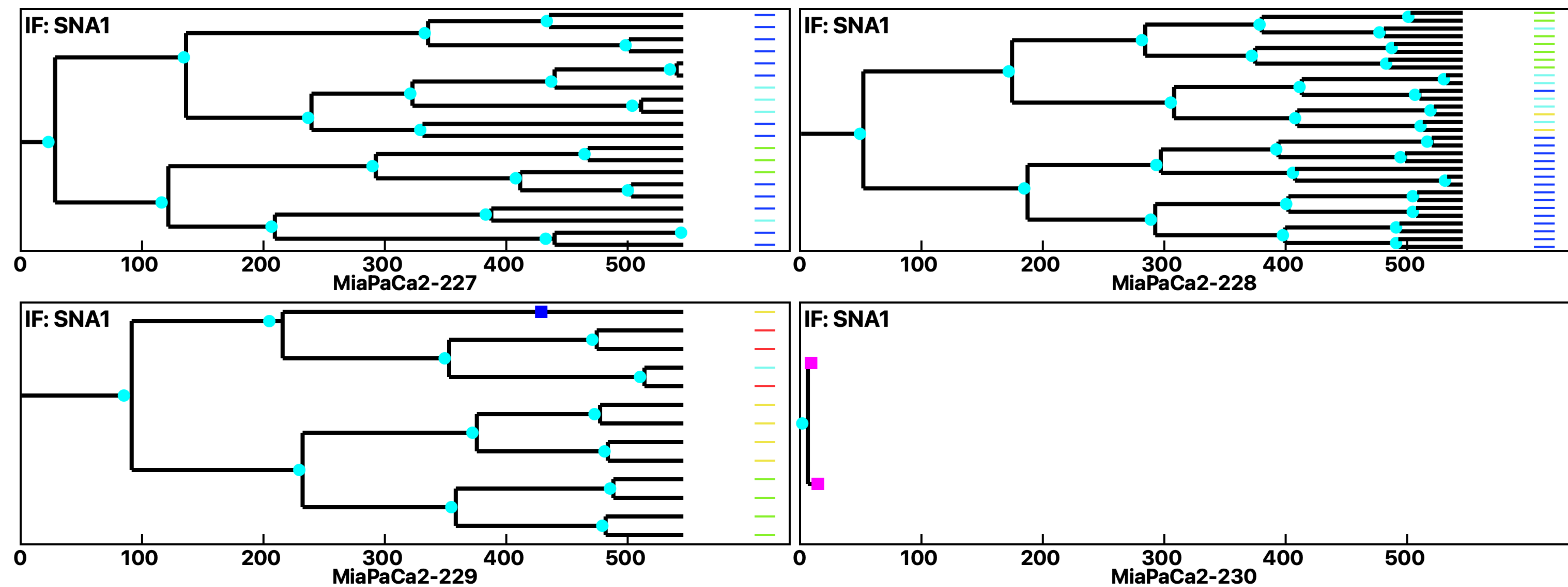

Supplement: Data S2 [file mmc4.pdf]

Fig. S1

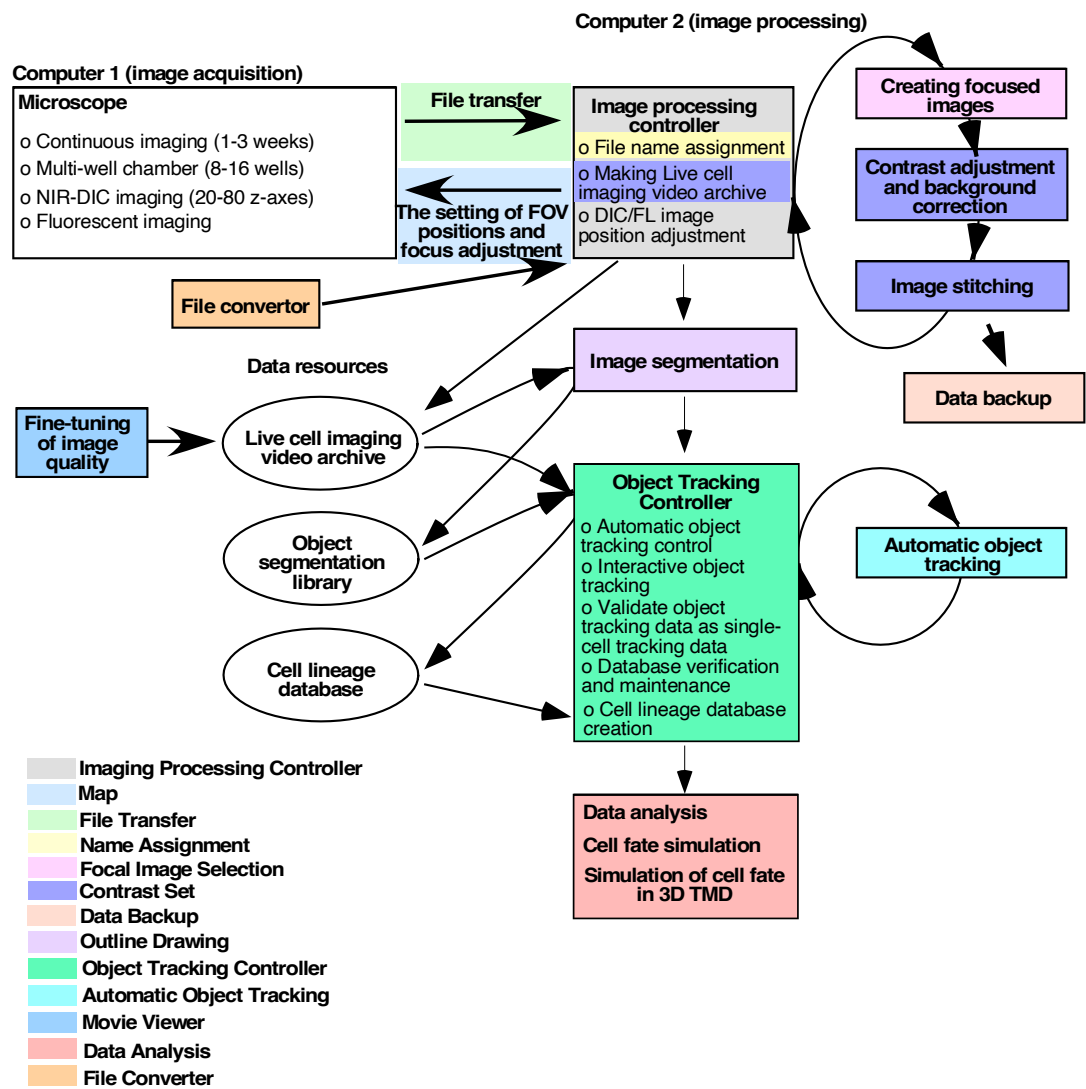

Supplement: Supplementary Figure S1 [file mmc10.pdf]

Fig. S2

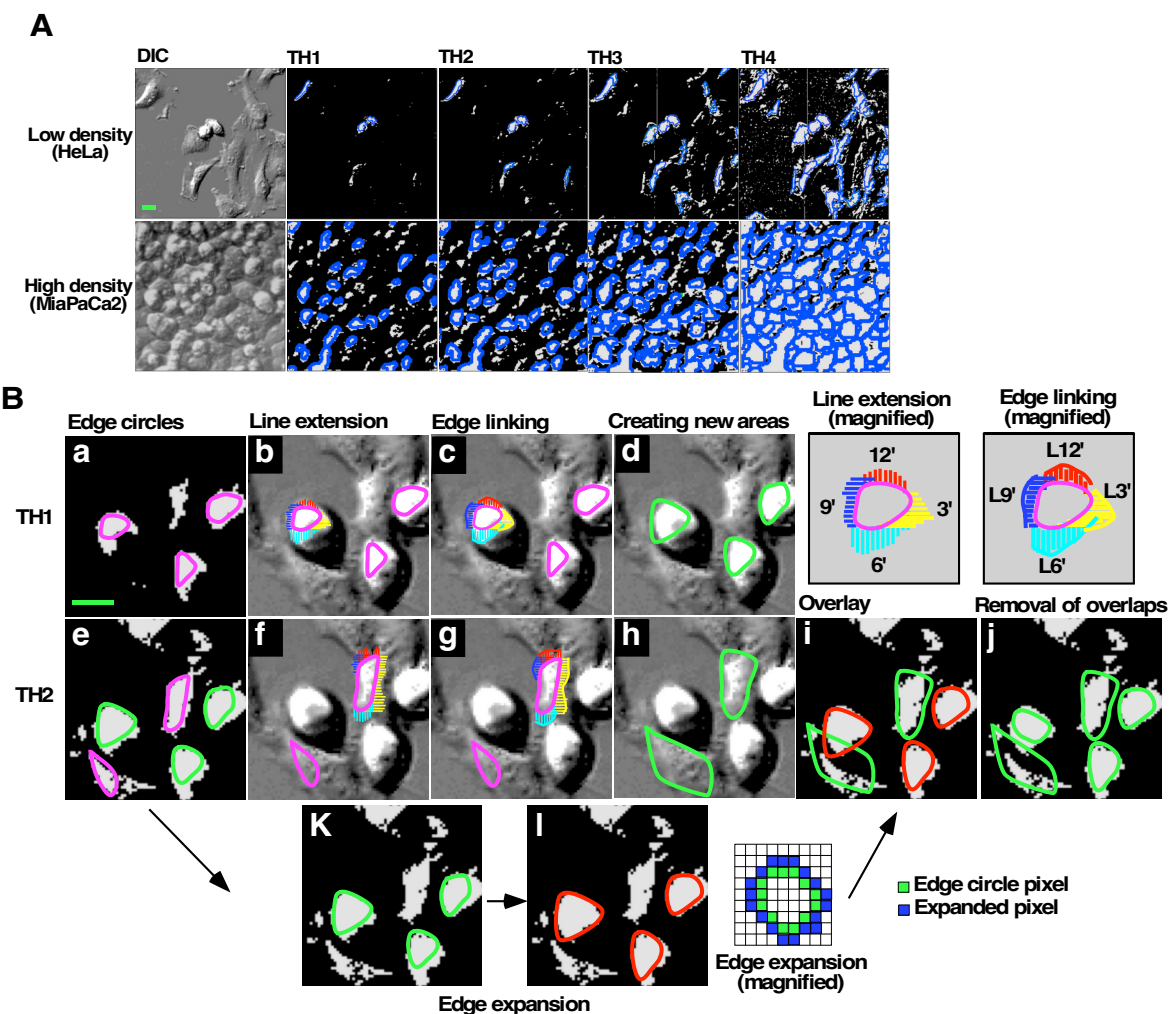

Supplement: Supplementary Figure S2 [file mmc11.pdf]

Fig. S3

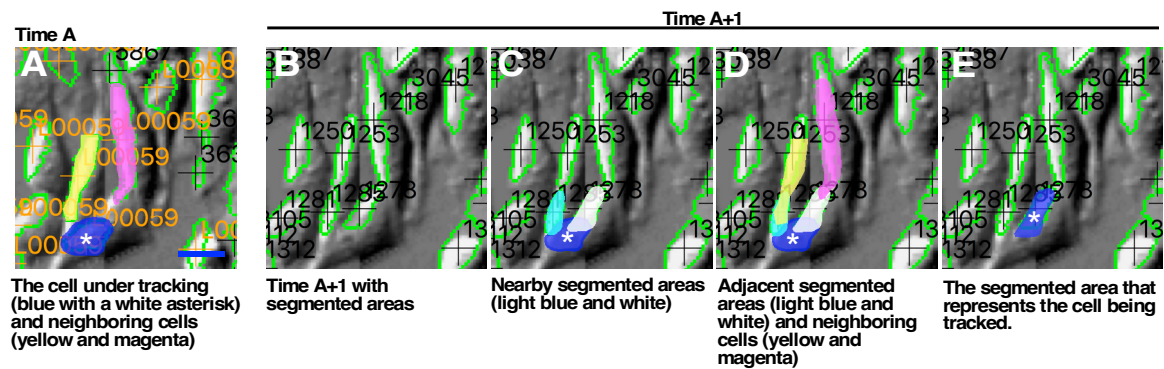

Supplement: Supplementary Figure S3 [file mmc12.pdf]

Fig. S4

A

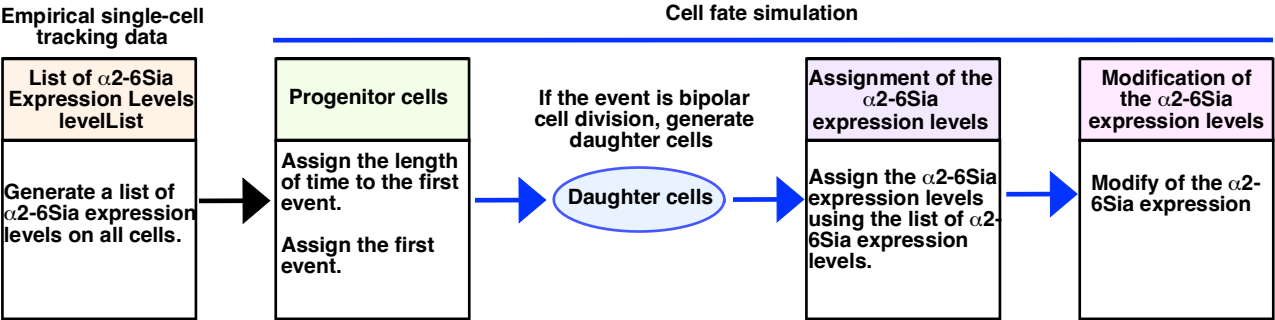

B

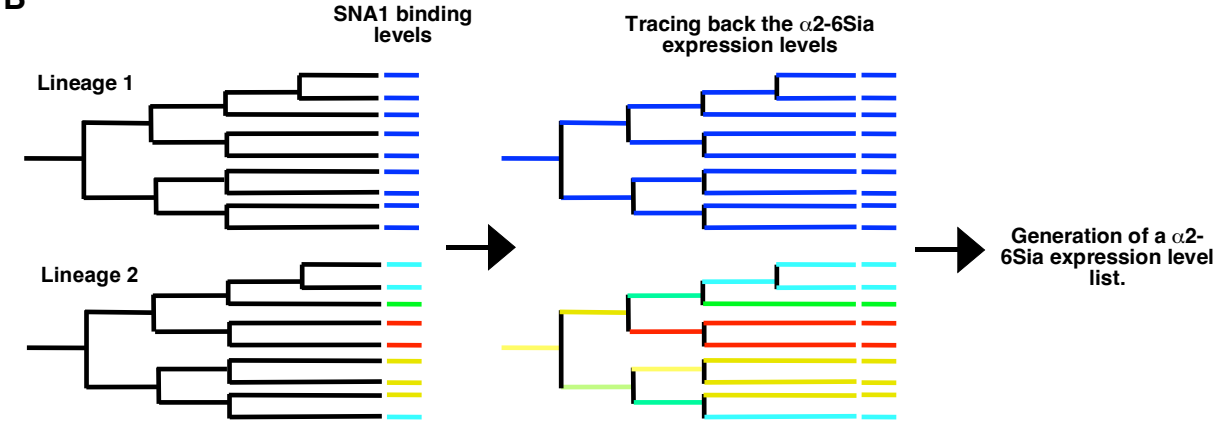

C

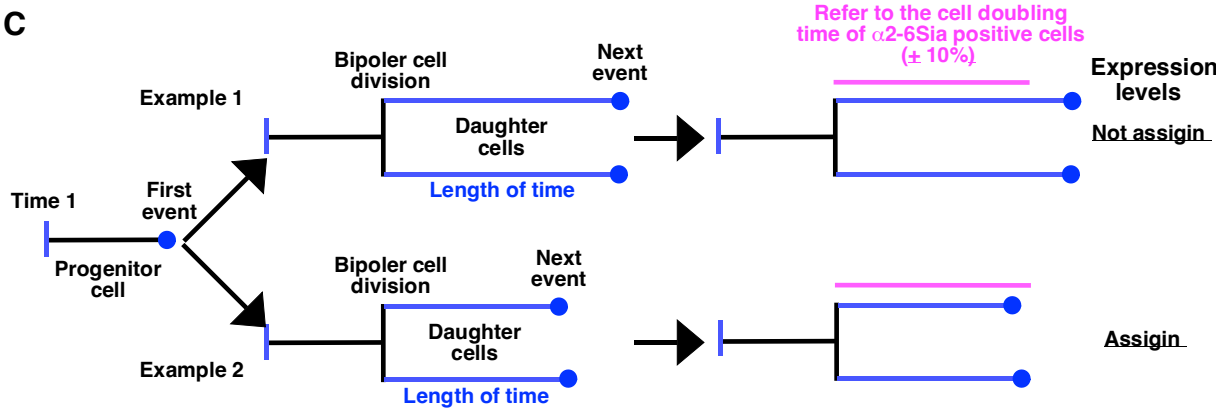

D

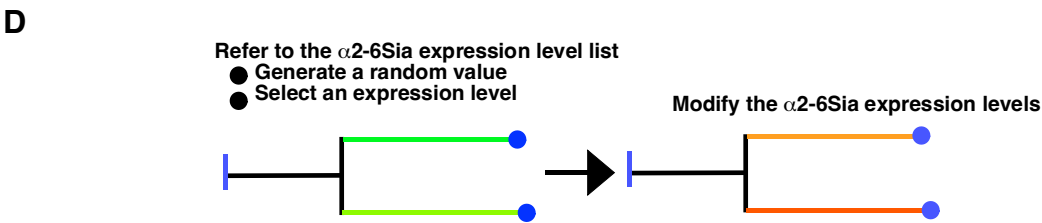

Supplement: Supplementary Figure S4 [file mmc13.pdf]

Fig. S5

Cancer cell to immune cell ratio

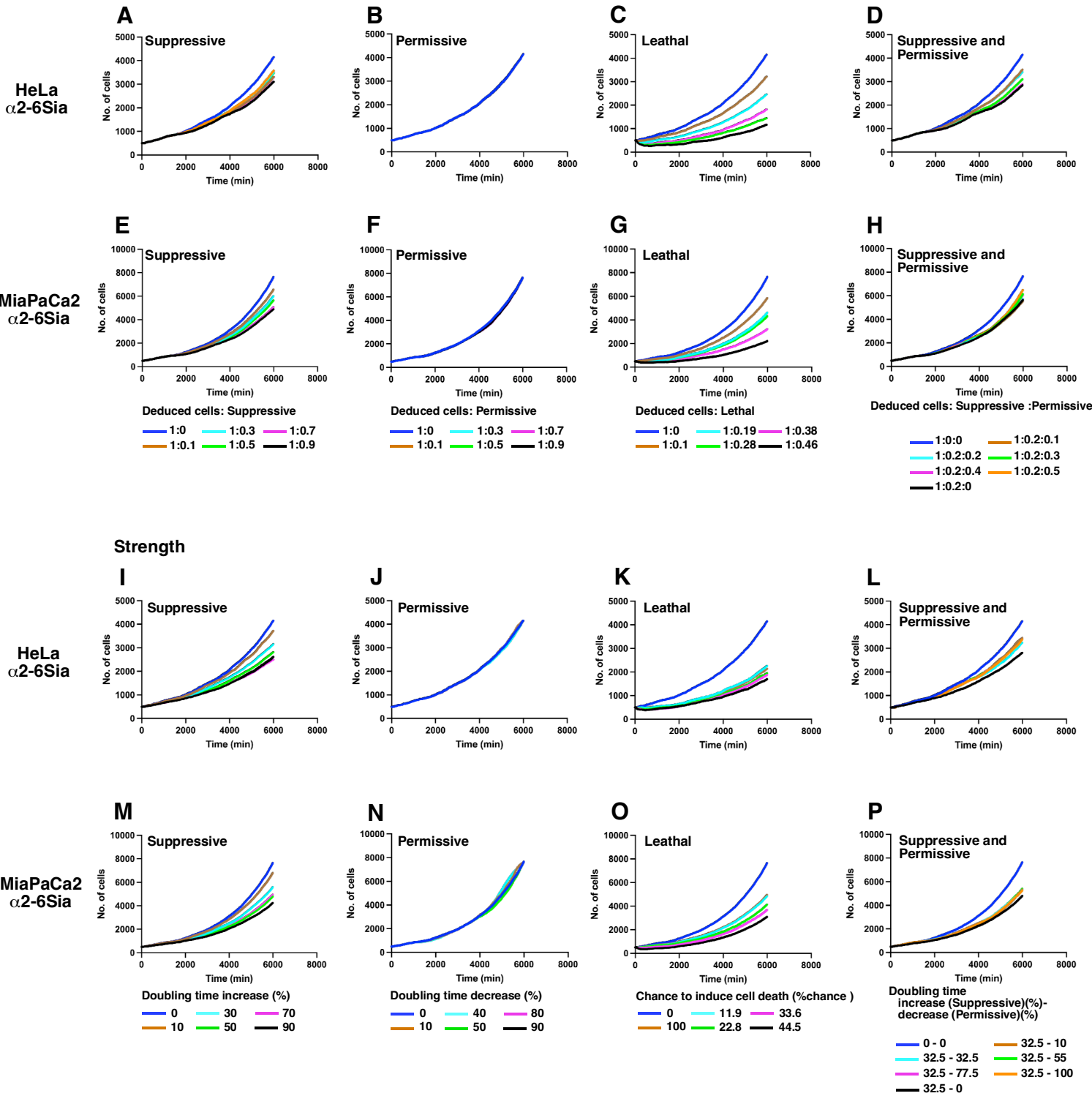

Supplement: Supplementary Figure S5 [file mmc14.pdf]
