# Supplementary material for: Cell fate simulation reveals cancer cell features in the tumor microenvironment
Source: J Biol Chem. 2024 Aug 20;300(9):107697. doi: 10.1016/j.jbc.2024.107697 (PMC11419826; doi:10.1016/j.jbc.2024.107697)

Analysis: Simulation, Treat.: HeLa a2-6Sia 1.5, Cell: HeLa-Simulation

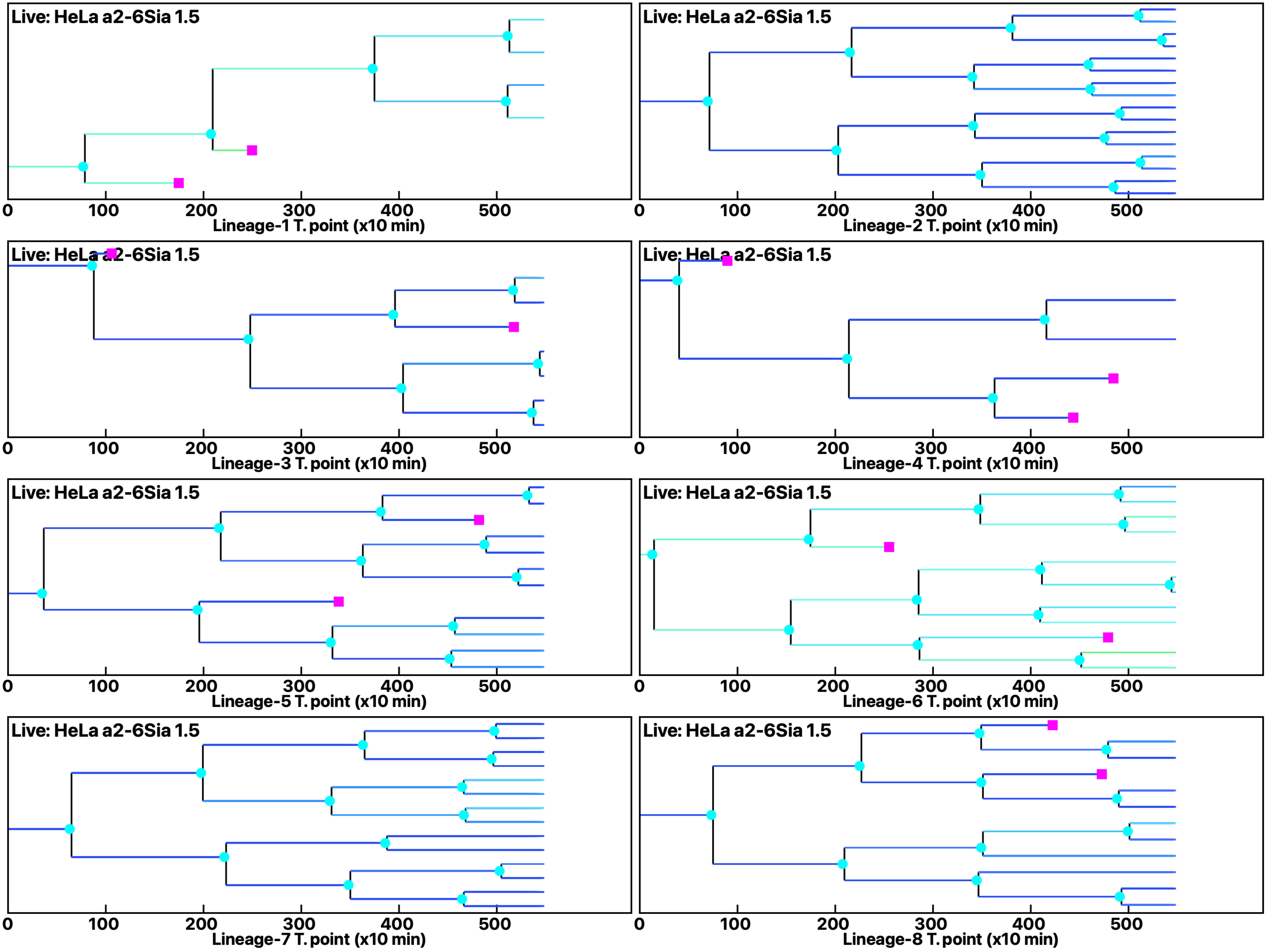

Analysis: Simulation, Treat.: HeLa a2-6Sia 1.5, Cell: HeLa-Simulation

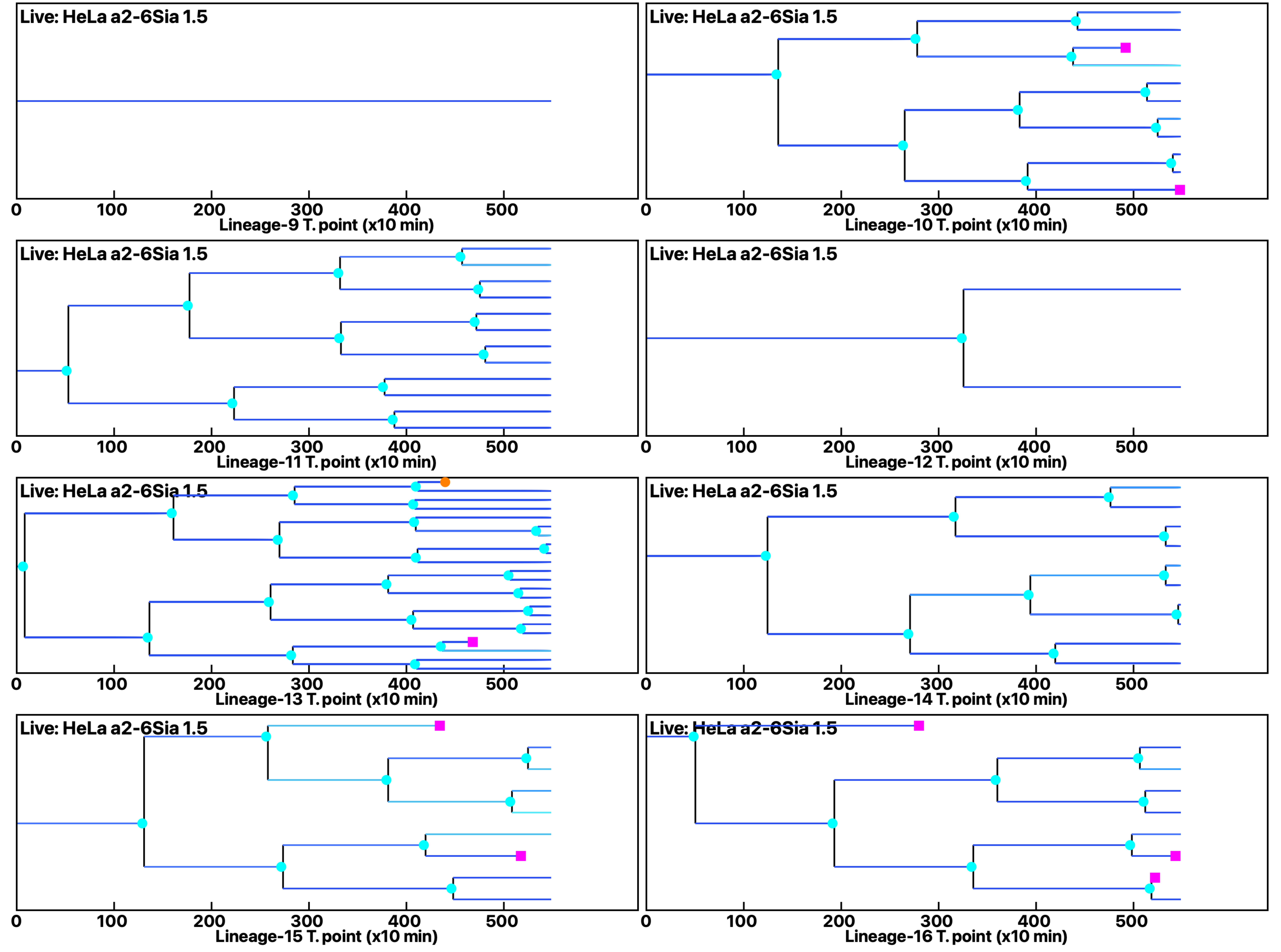

Analysis: Simulation, Treat.: HeLa a2-6Sia 1.5, Cell: HeLa-Simulation

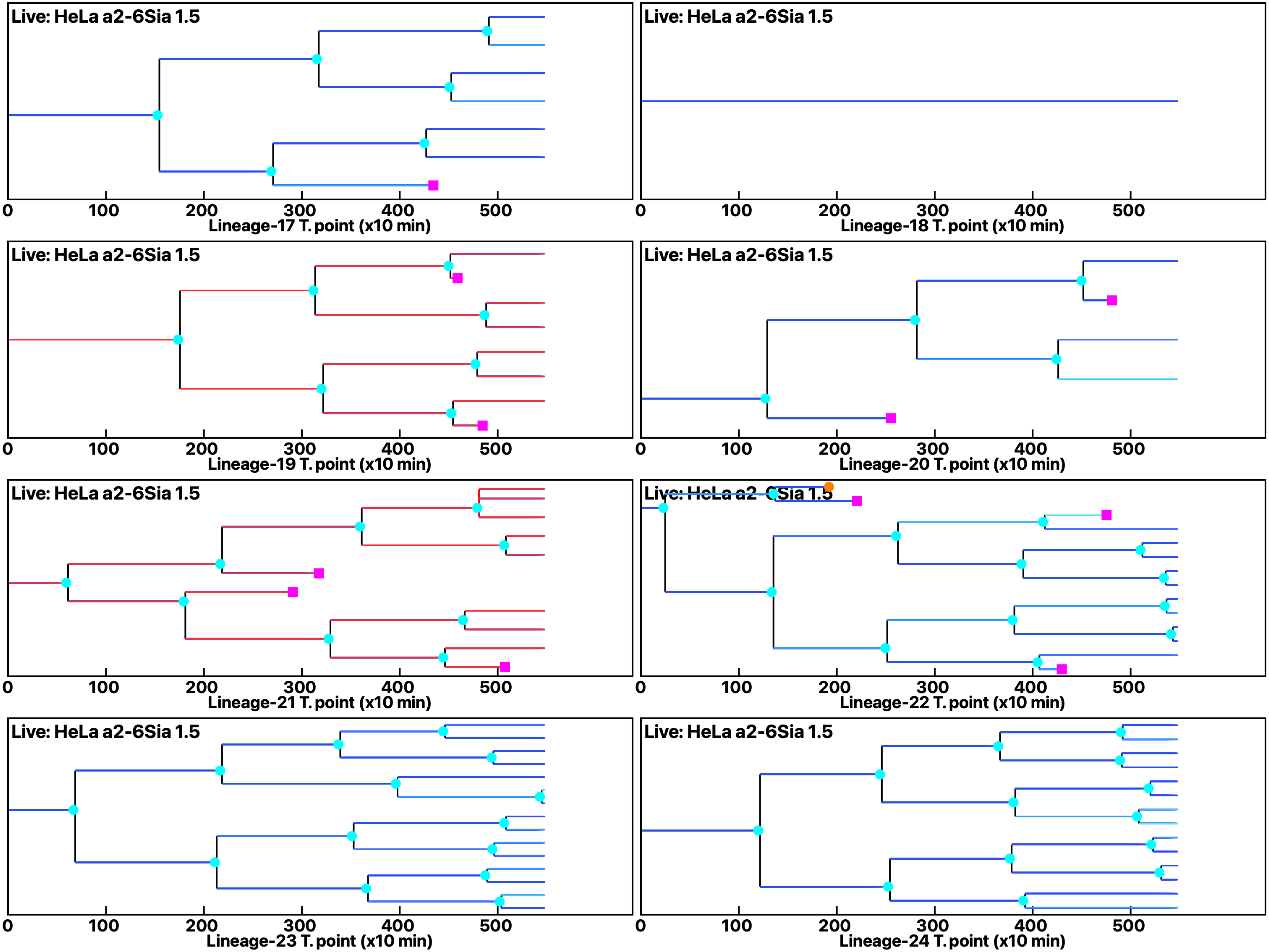

Analysis: Simulation, Treat.: HeLa a2-6Sia 1.5, Cell: HeLa-Simulation

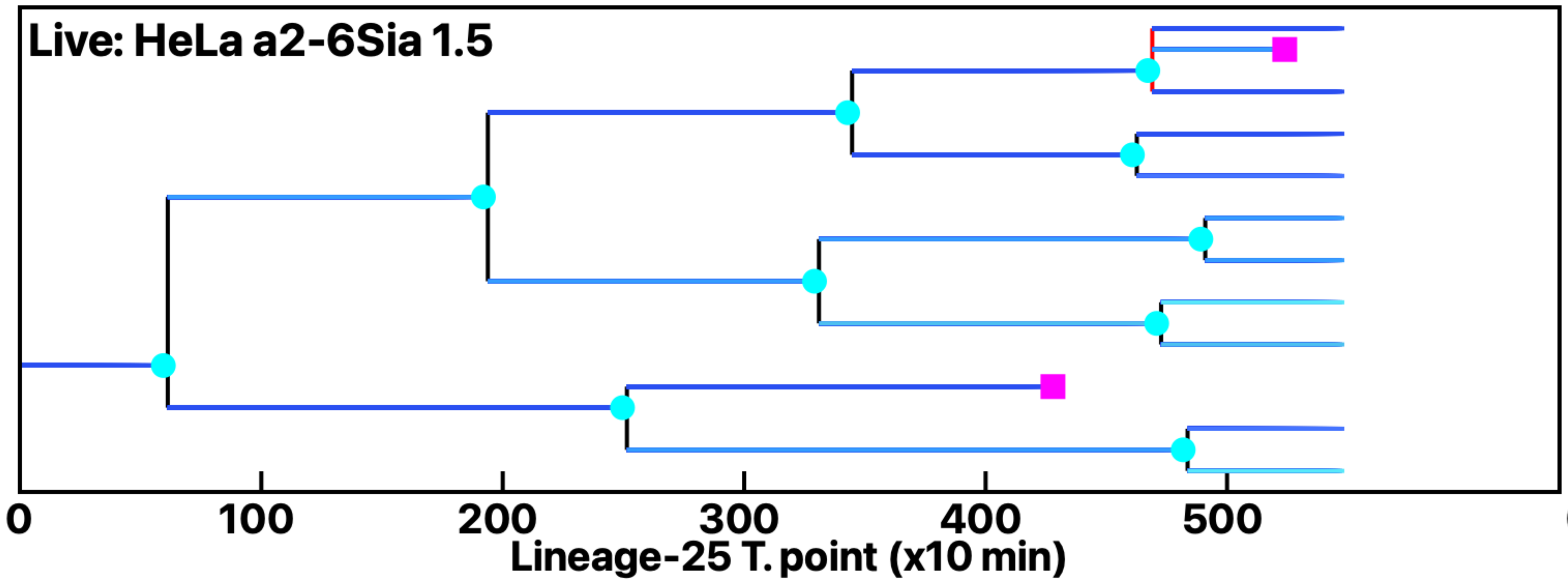

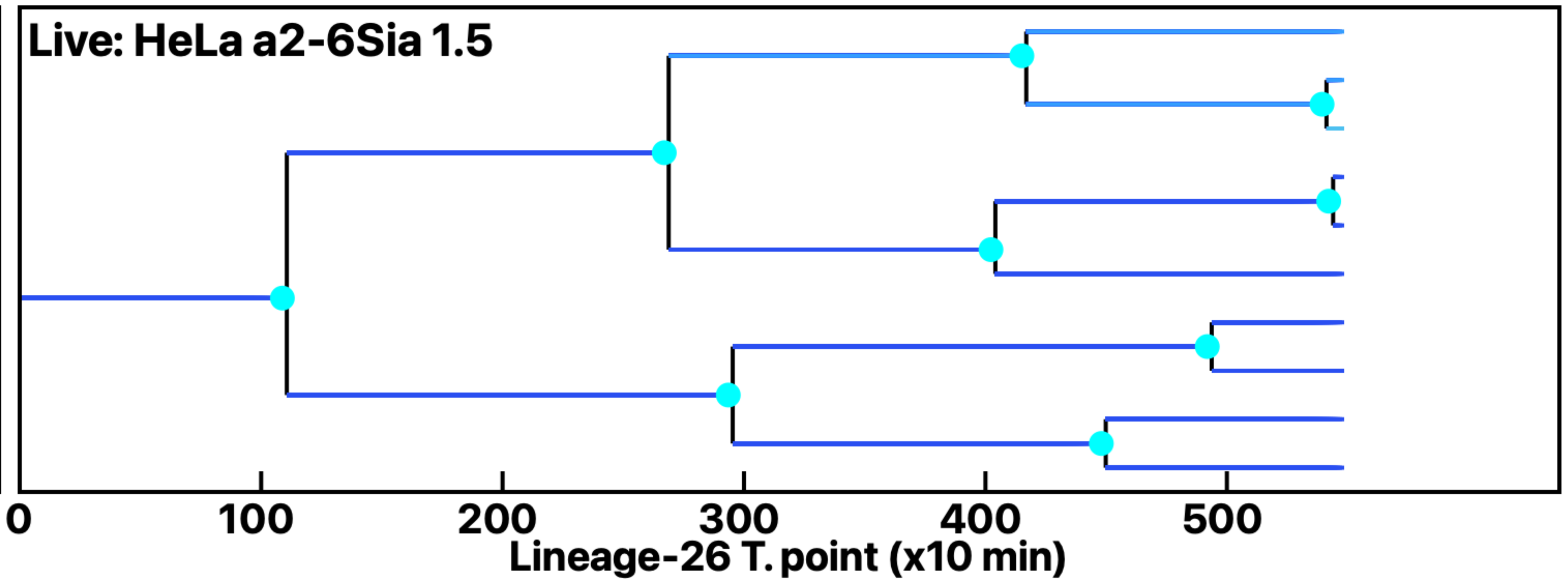

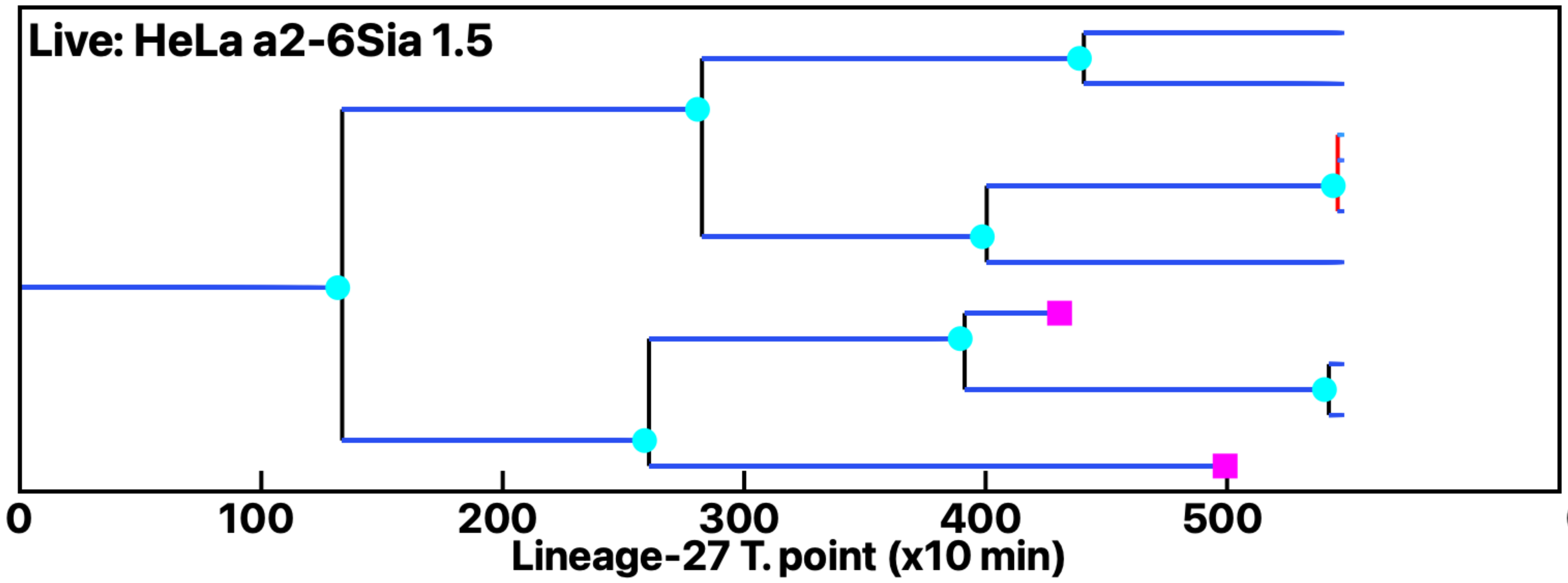

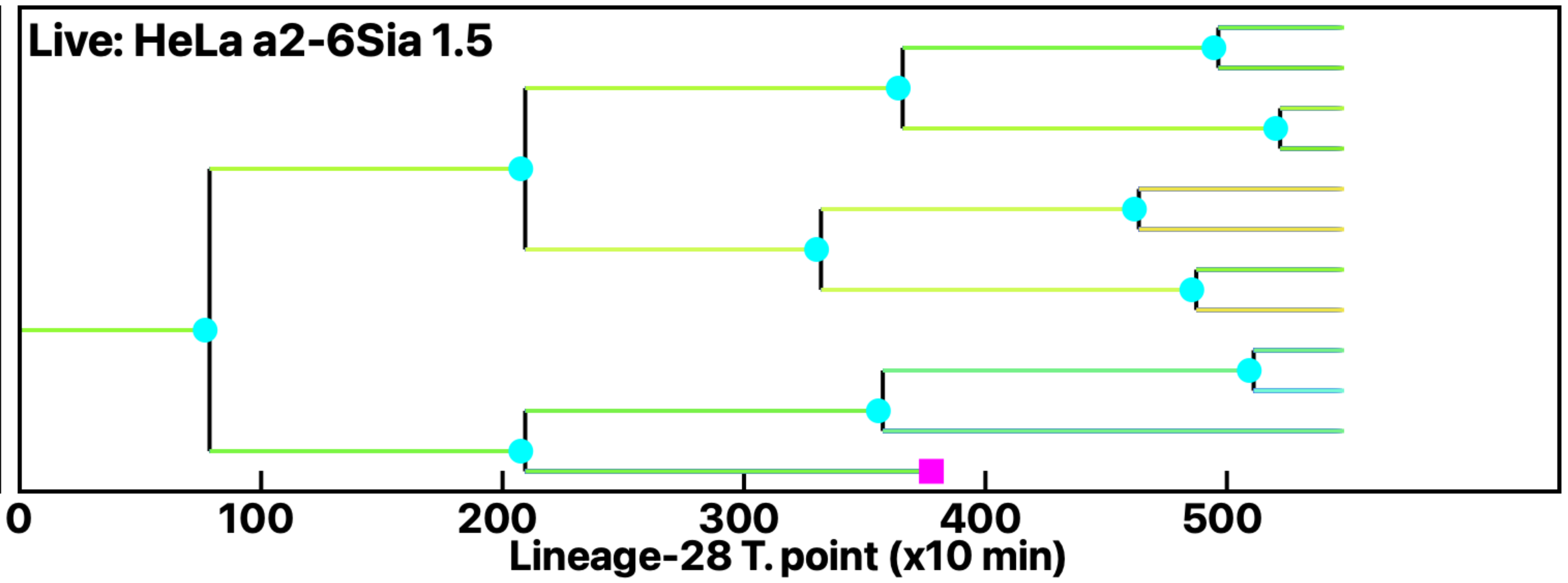

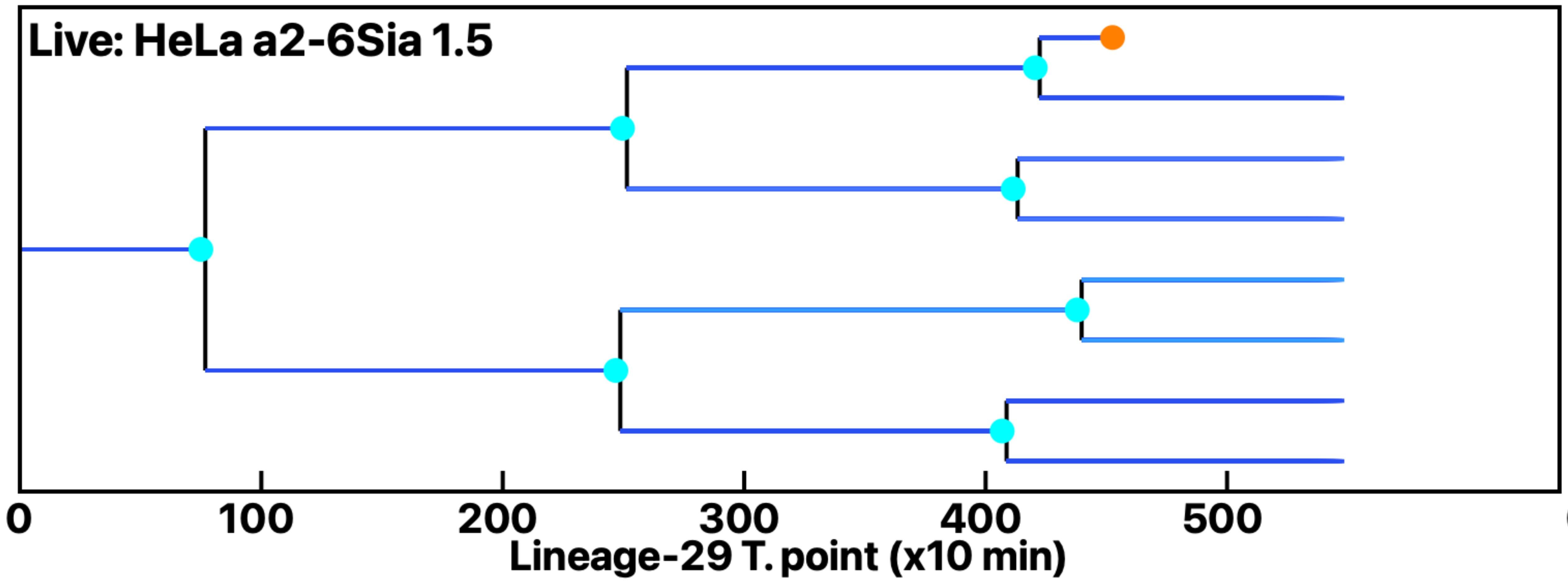

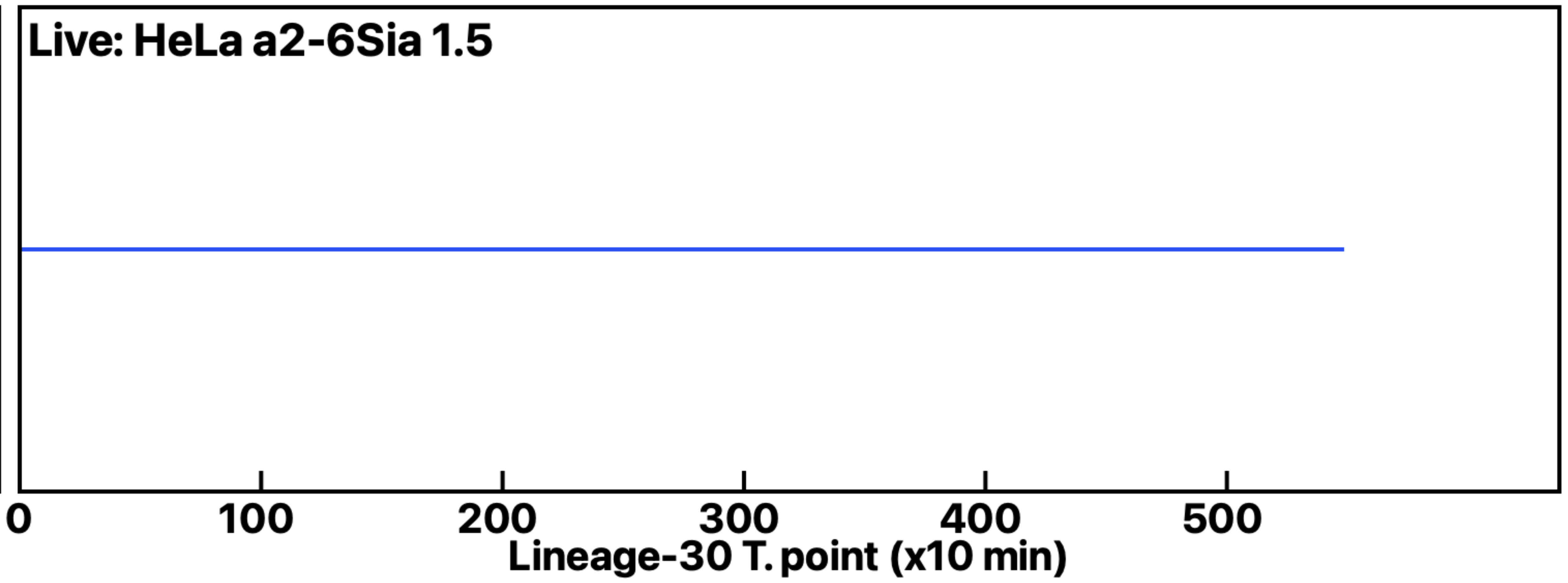

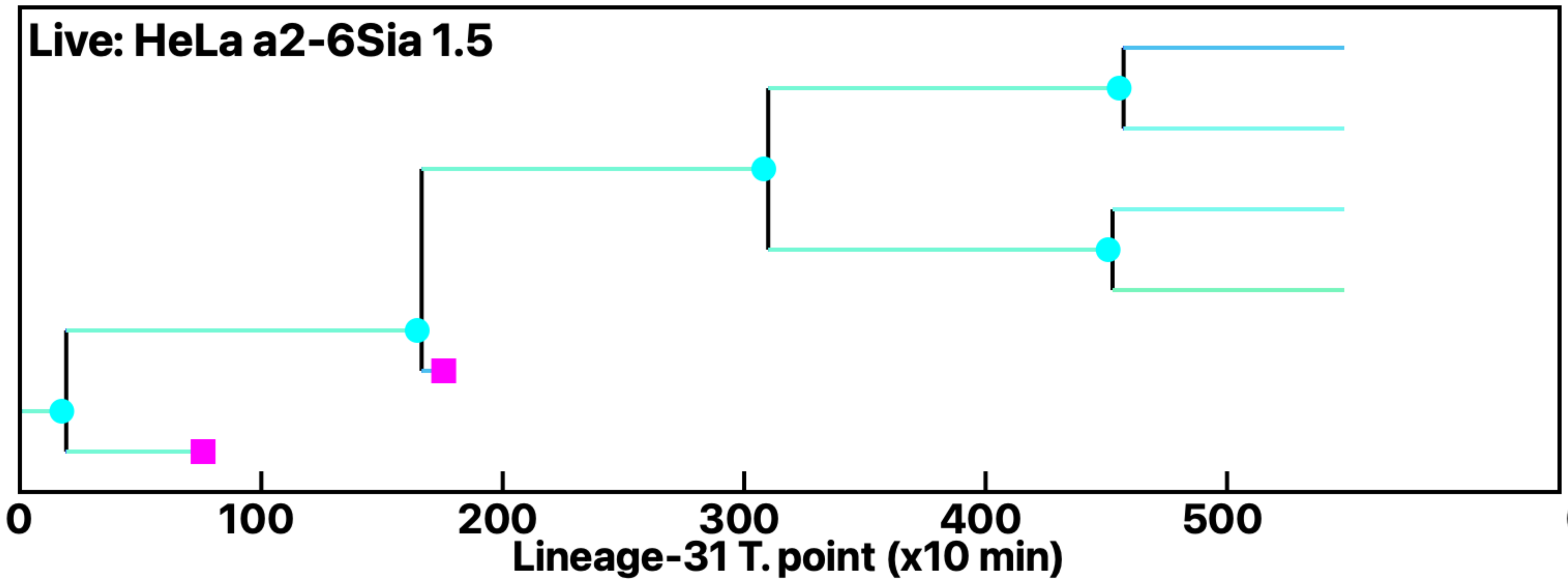

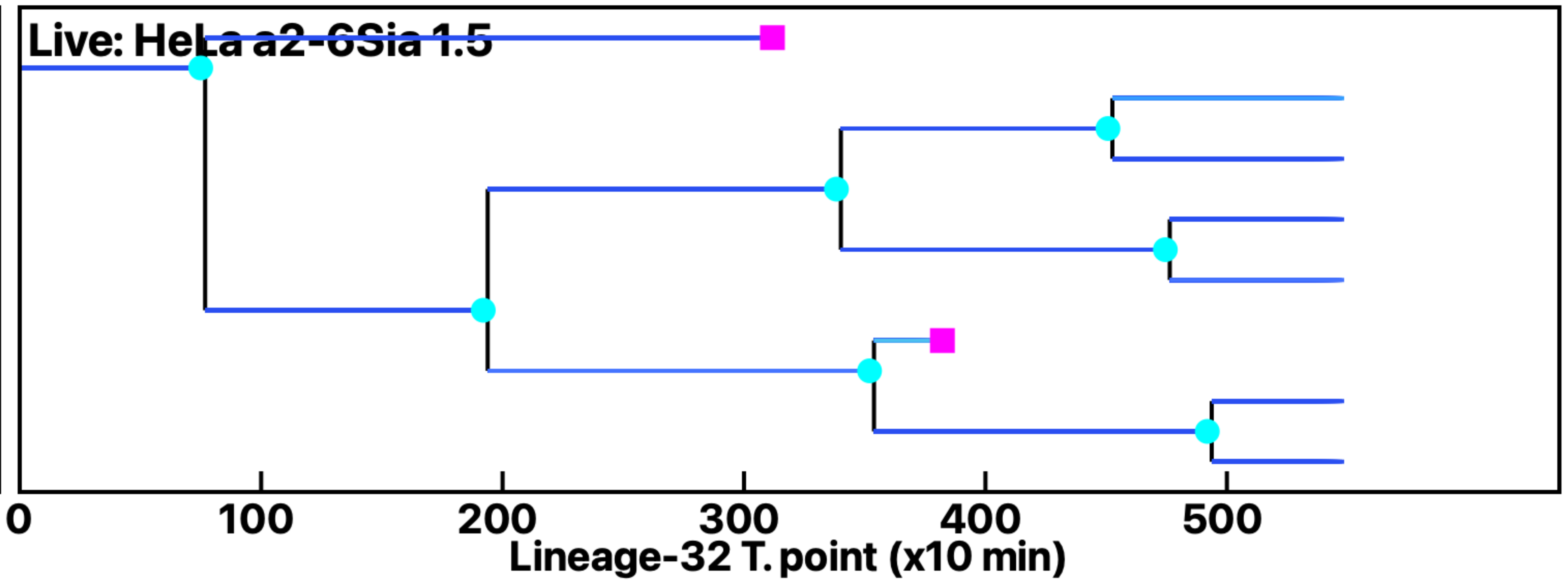

Analysis: Simulation, Treat.: HeLa a2-6Sia 1.5, Cell: HeLa-Simulation

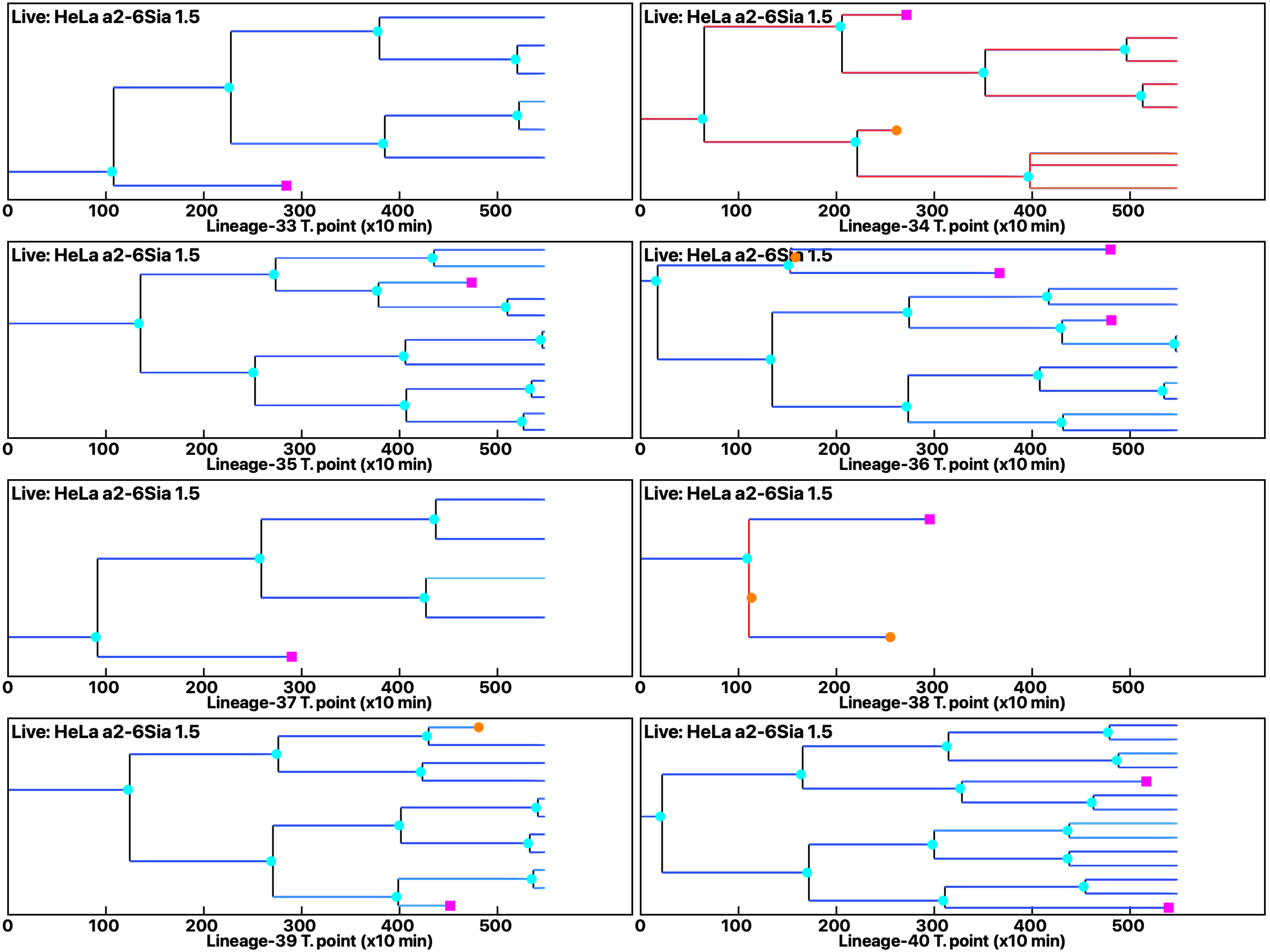

Analysis: Simulation, Treat.: HeLa a2-6Sia 1.5, Cell: HeLa-Simulation

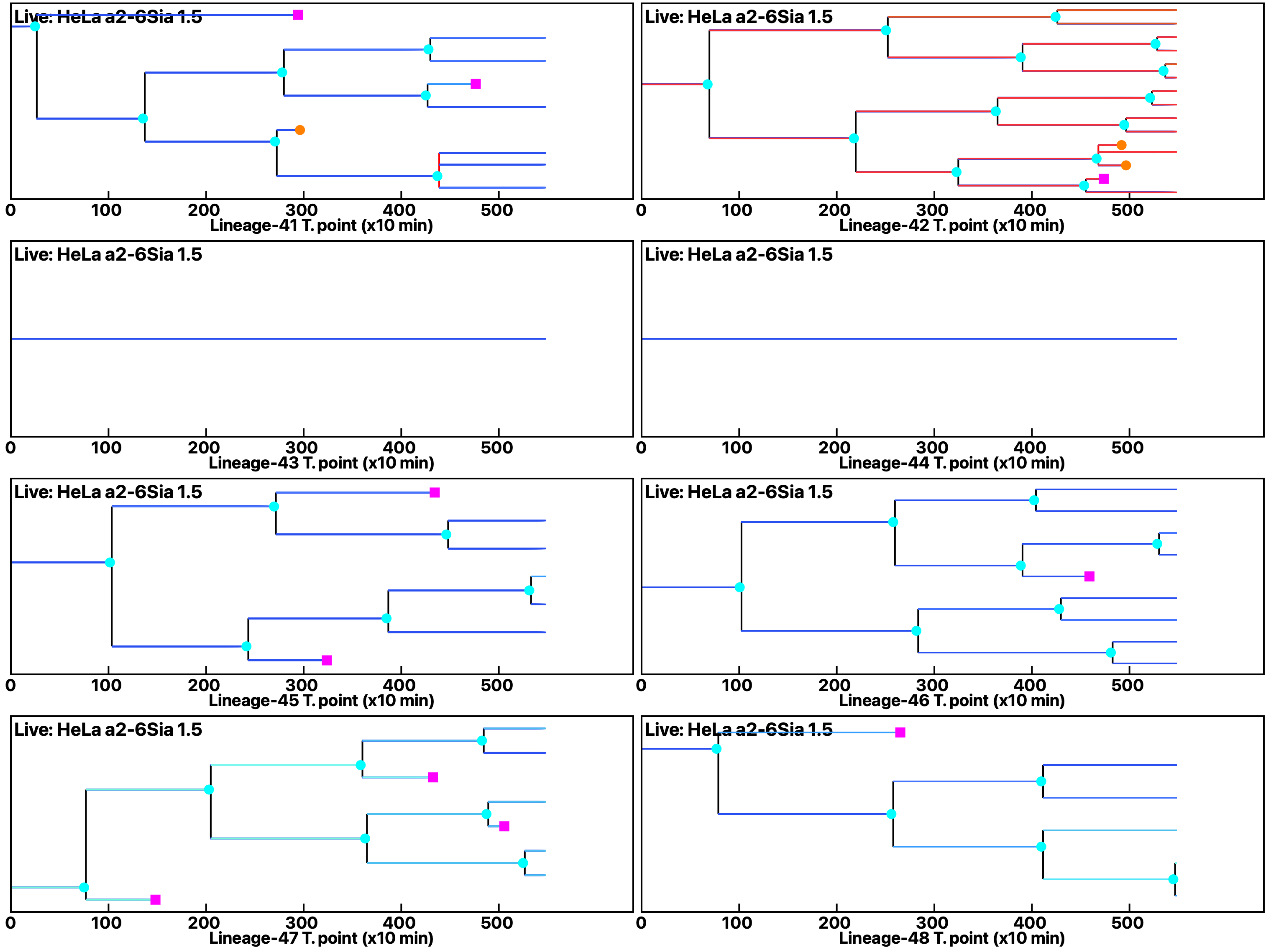

Analysis: Simulation, Treat.: HeLa a2-6Sia 1.5, Cell: HeLa-Simulation

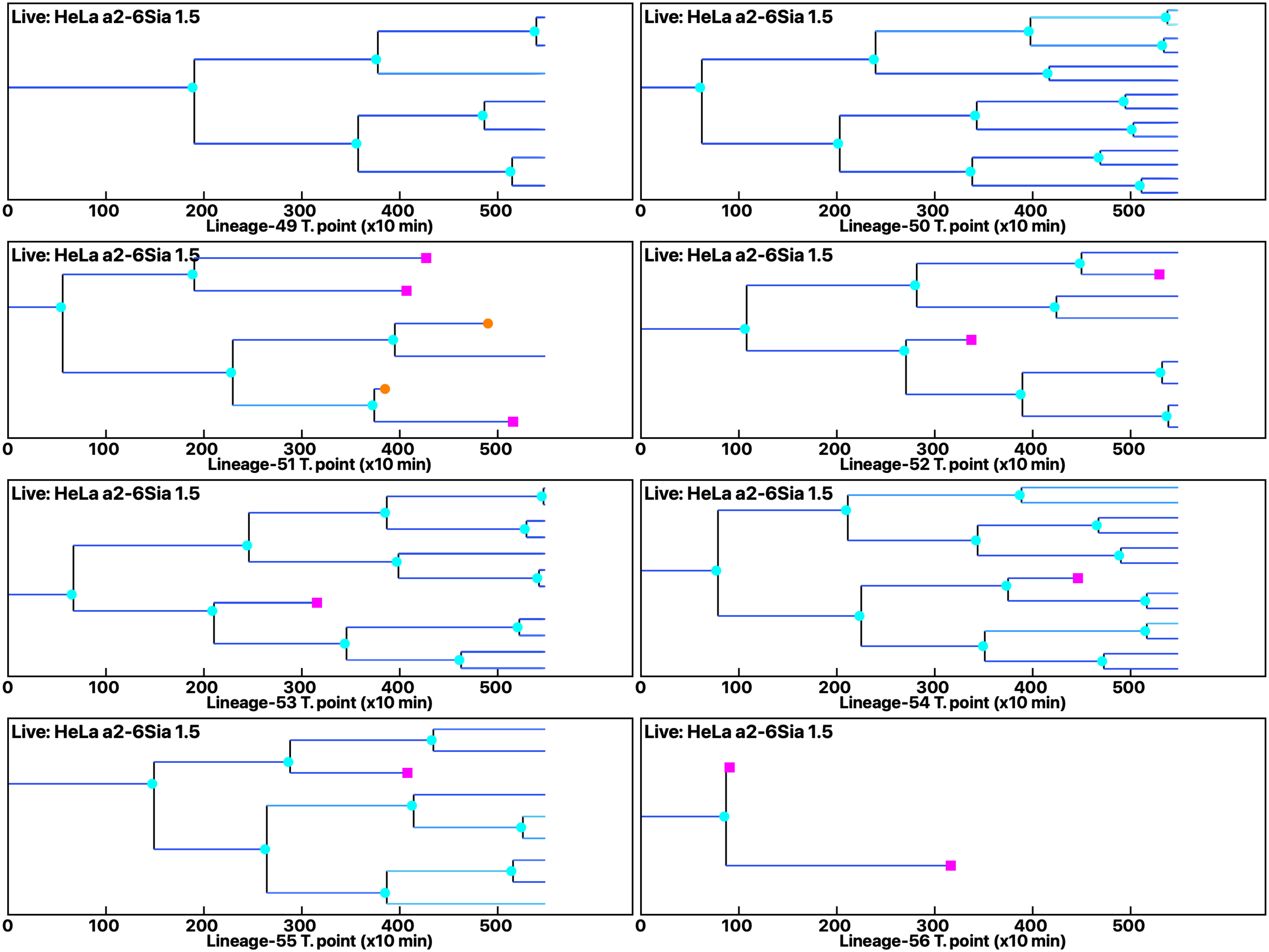

Analysis: Simulation, Treat.: HeLa a2-6Sia 1.5, Cell: HeLa-Simulation

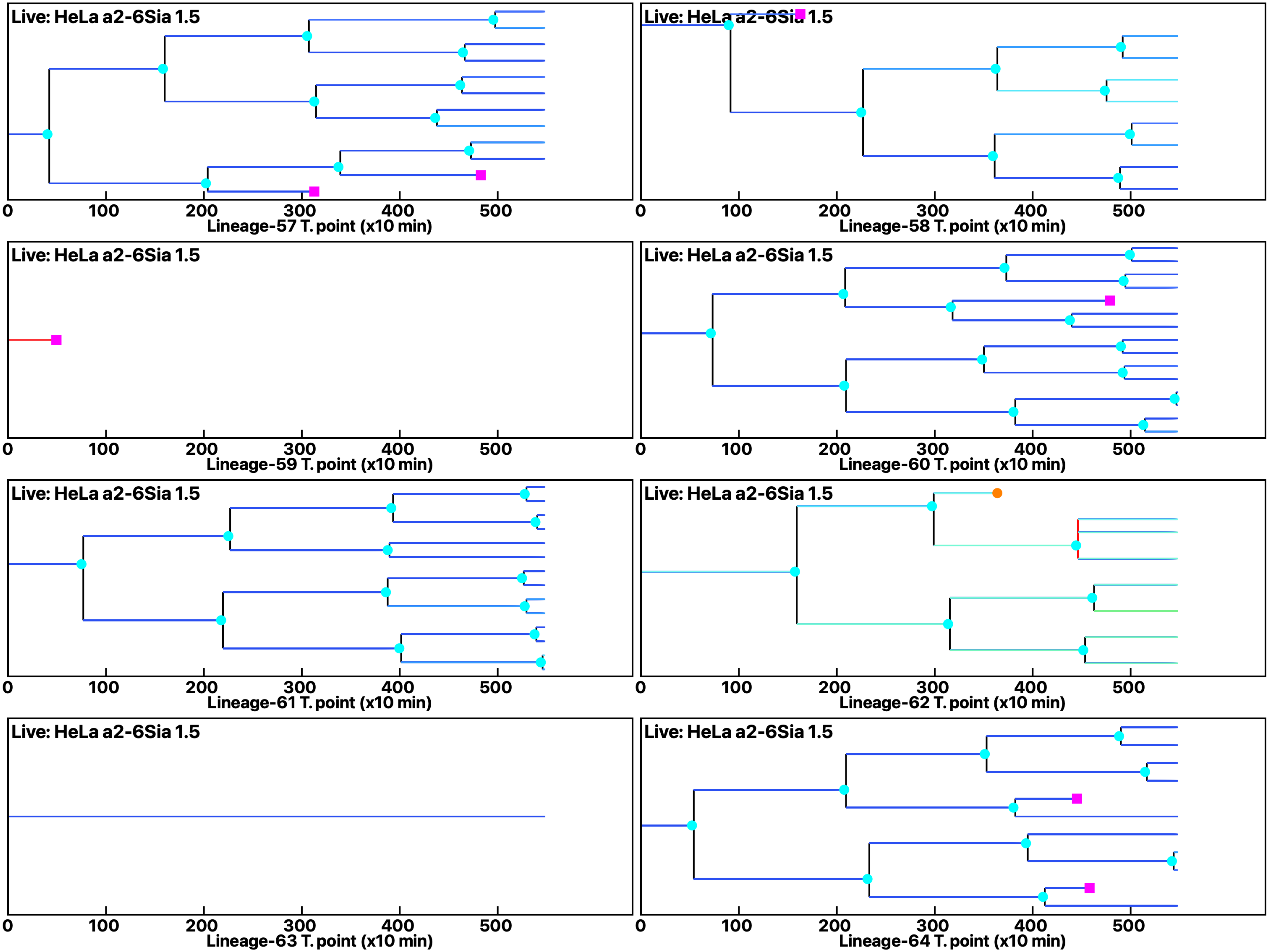

Analysis: Simulation, Treat.: HeLa a2-6Sia 1.5, Cell: HeLa-Simulation

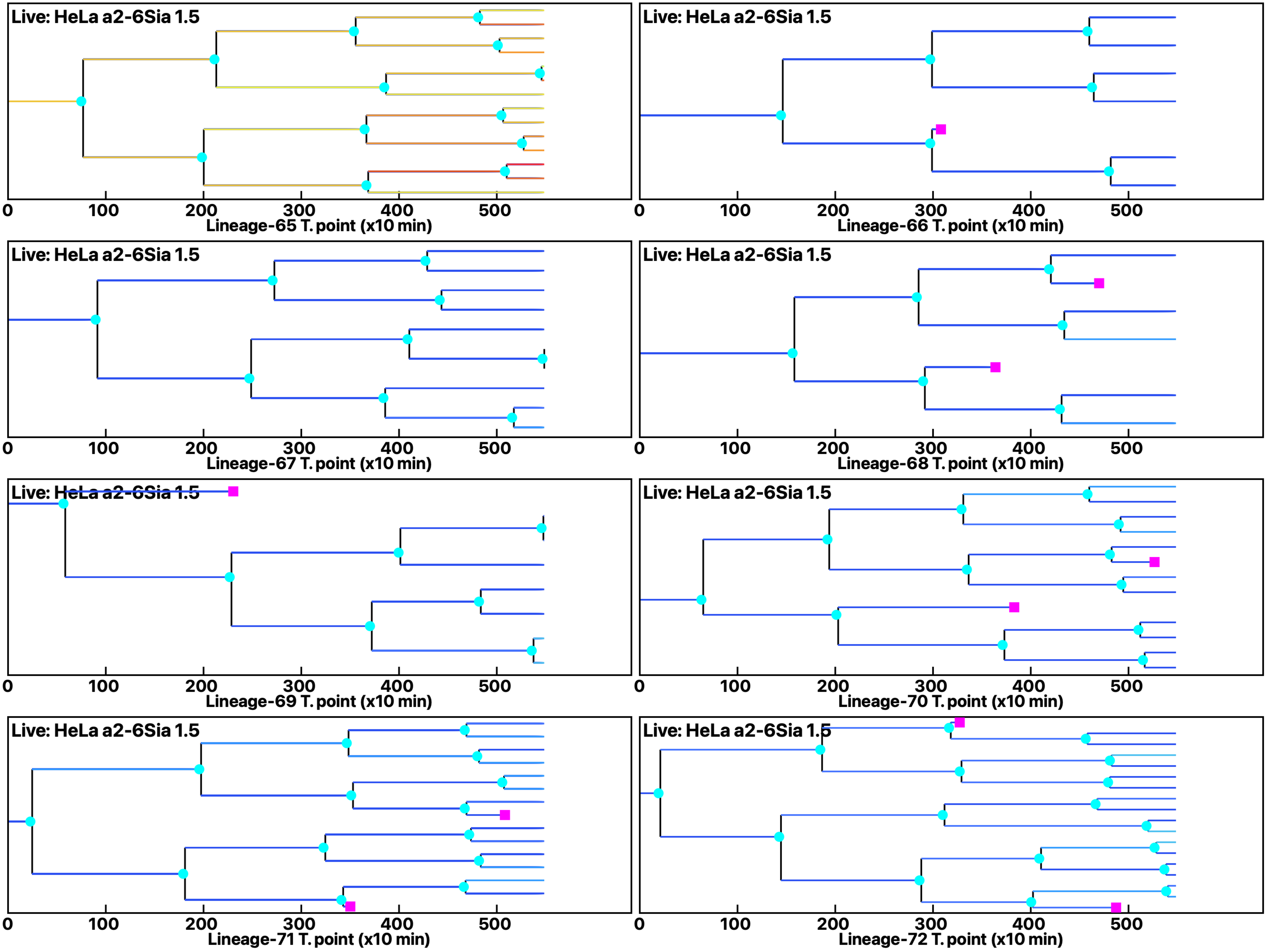

Analysis: Simulation, Treat.: HeLa a2-6Sia 1.5, Cell: HeLa-Simulation

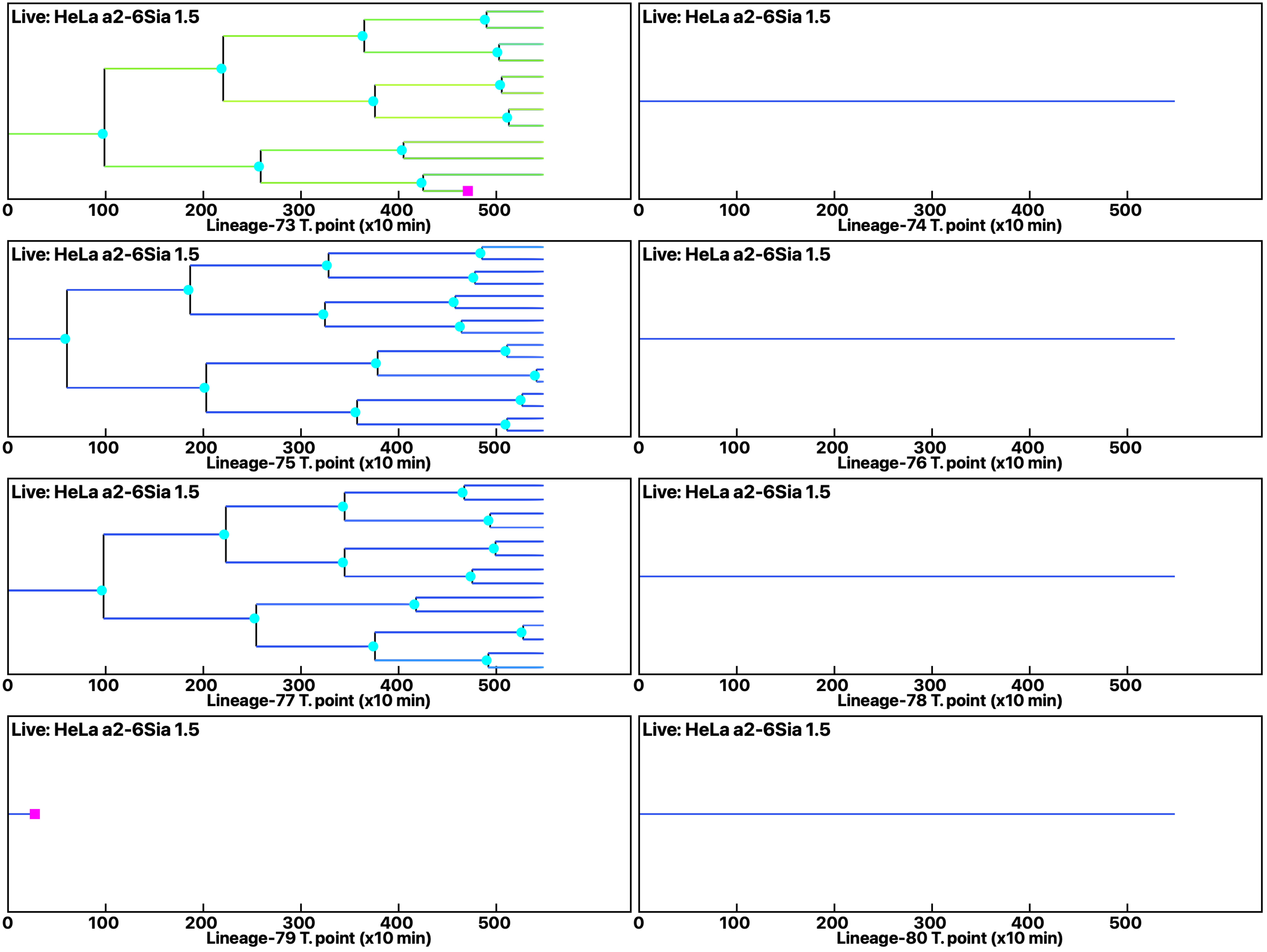

Analysis: Simulation, Treat.: HeLa a2-6Sia 1.5, Cell: HeLa-Simulation

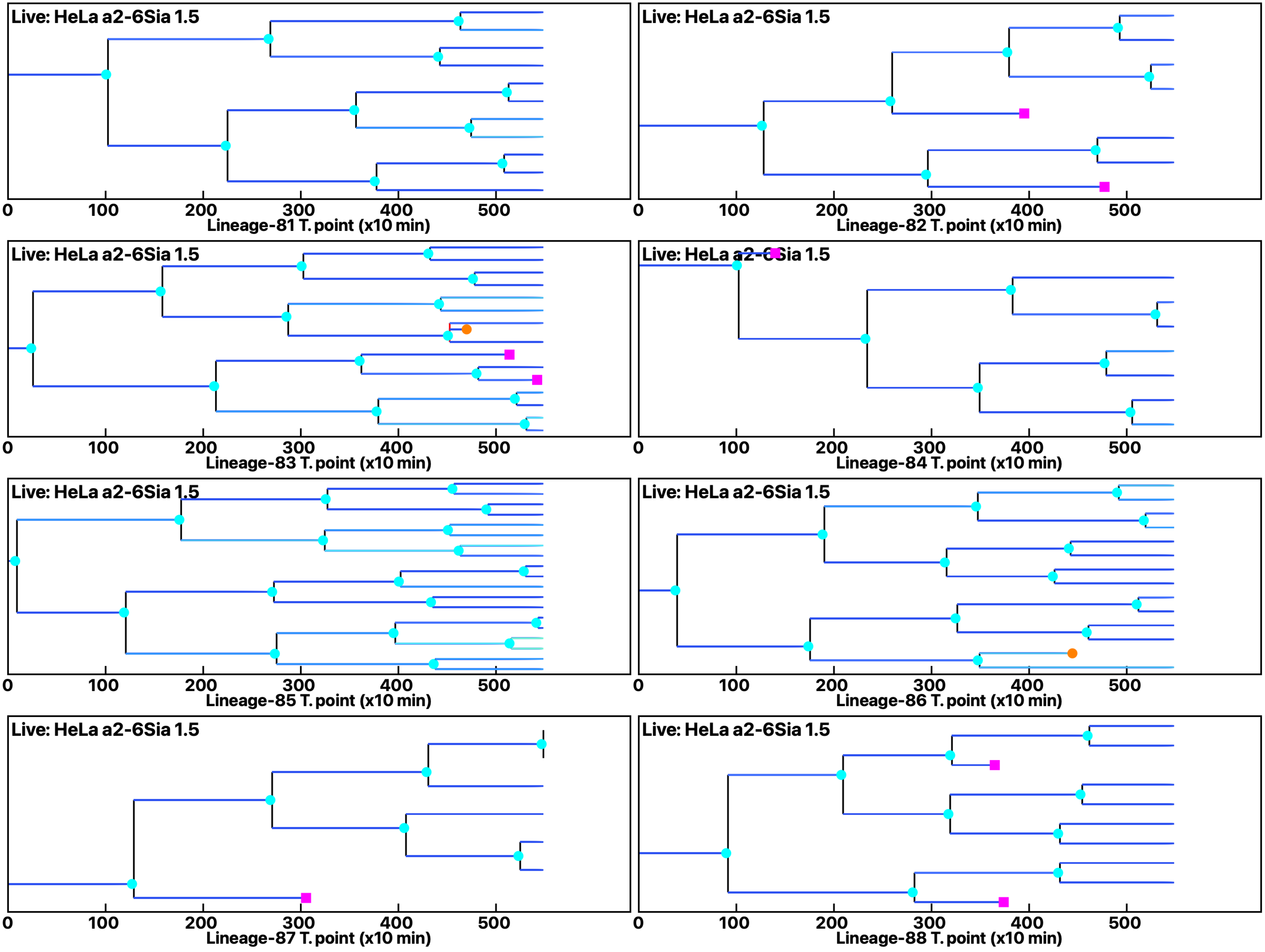

Analysis: Simulation, Treat.: HeLa a2-6Sia 1.5, Cell: HeLa-Simulation

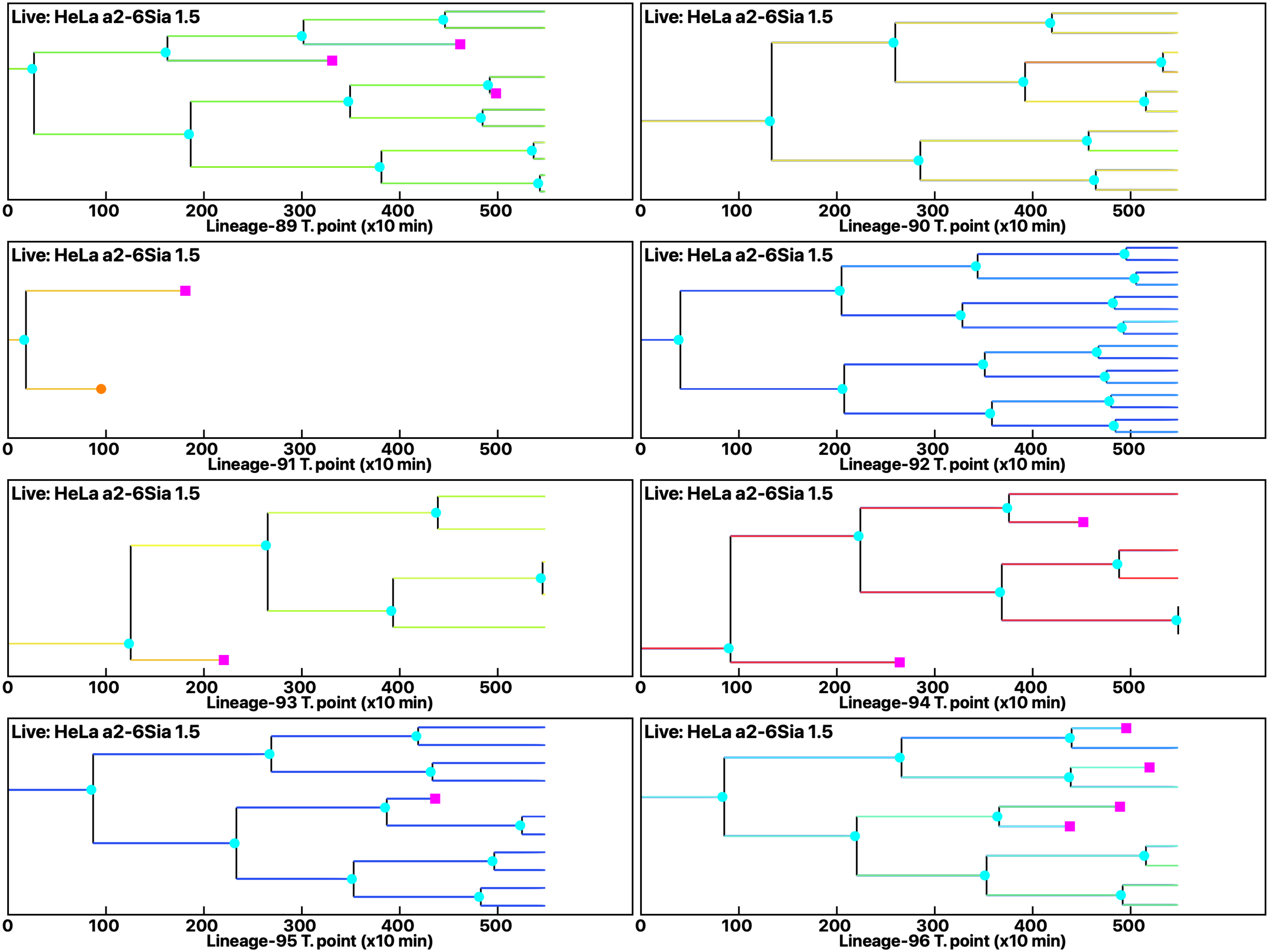

Analysis: Simulation, Treat.: HeLa a2-6Sia 1.5, Cell: HeLa-Simulation

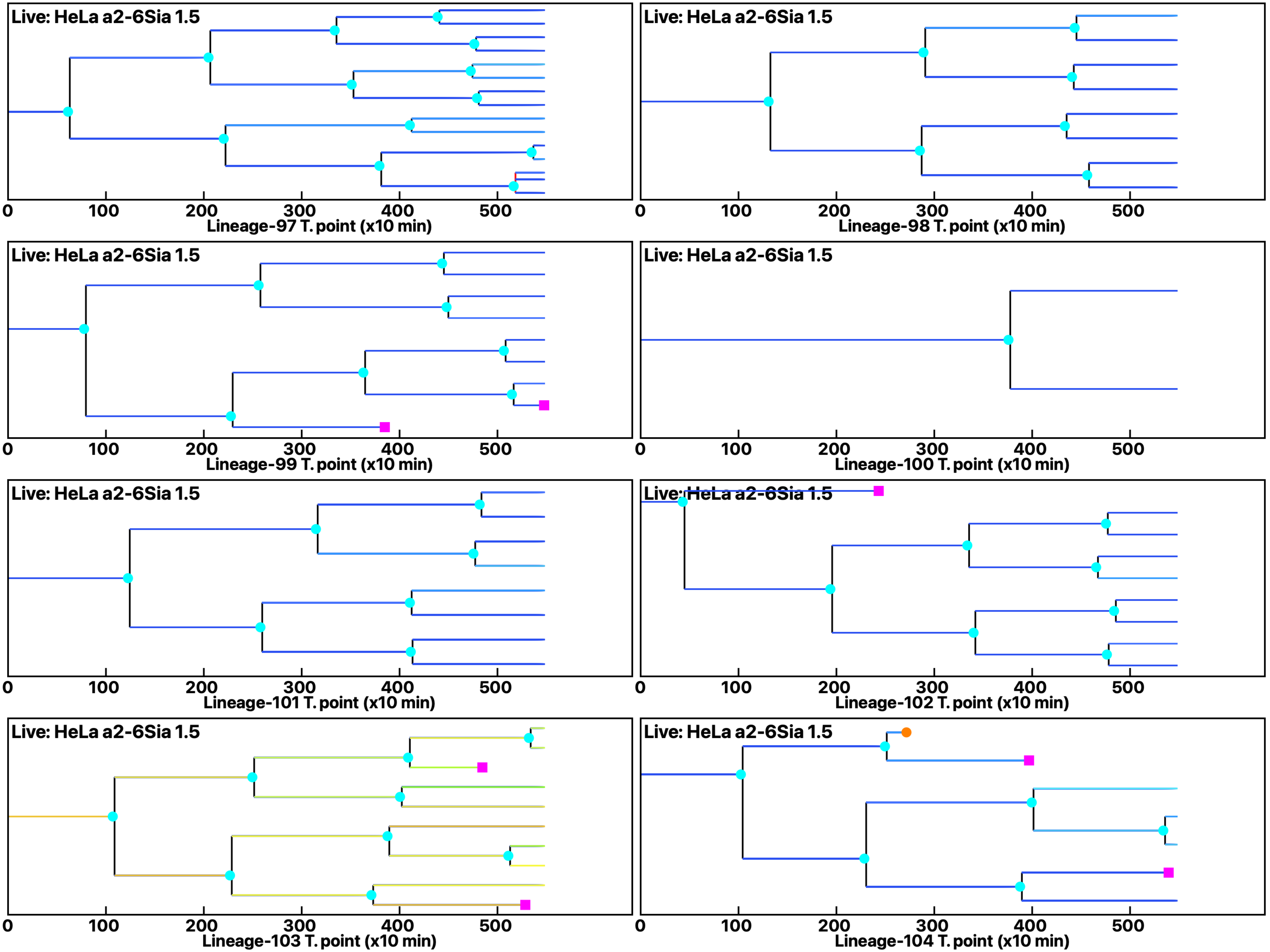

Analysis: Simulation, Treat.: HeLa a2-6Sia 1.5, Cell: HeLa-Simulation

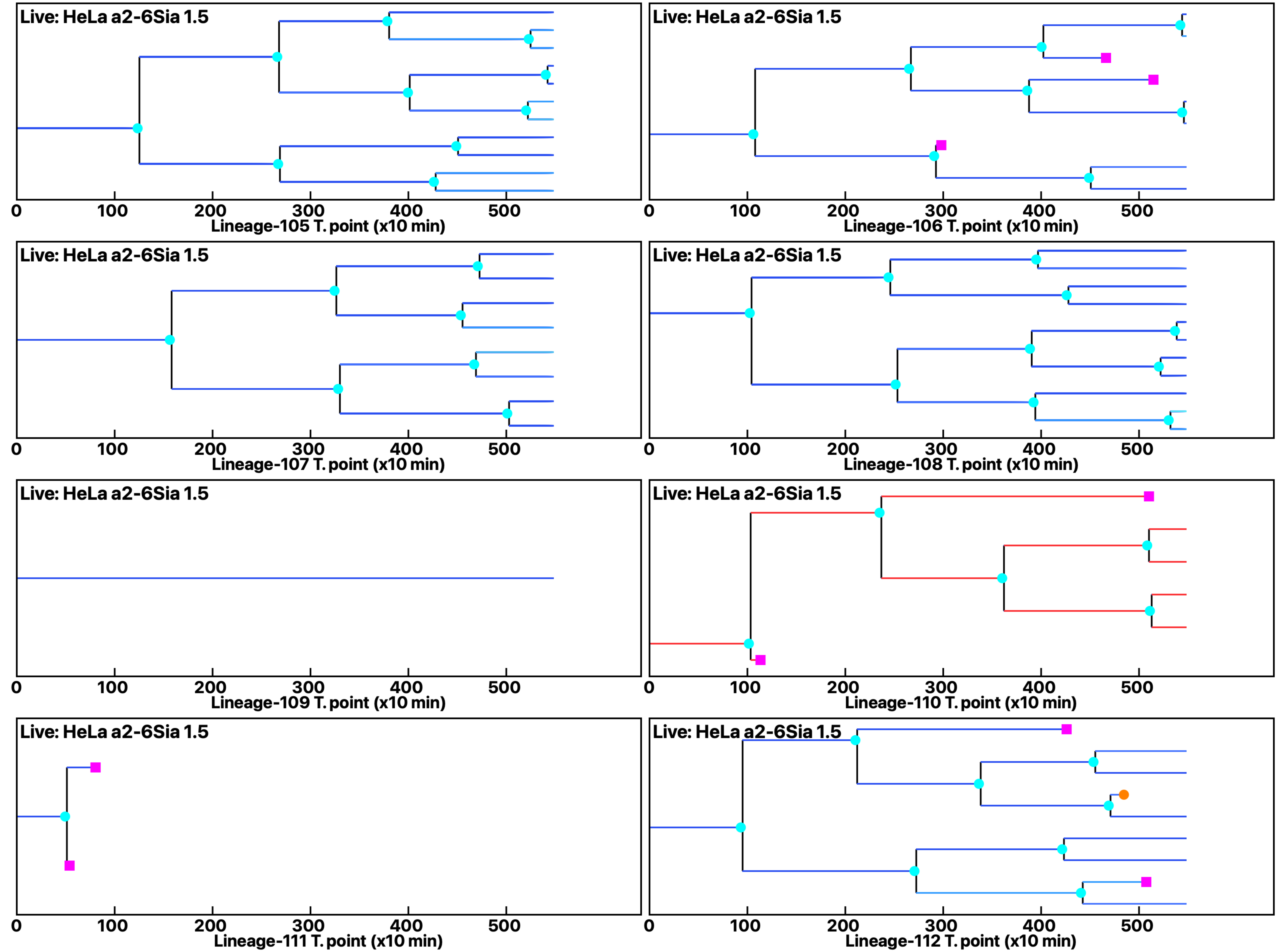

Analysis: Simulation, Treat.: HeLa a2-6Sia 1.5, Cell: HeLa-Simulation

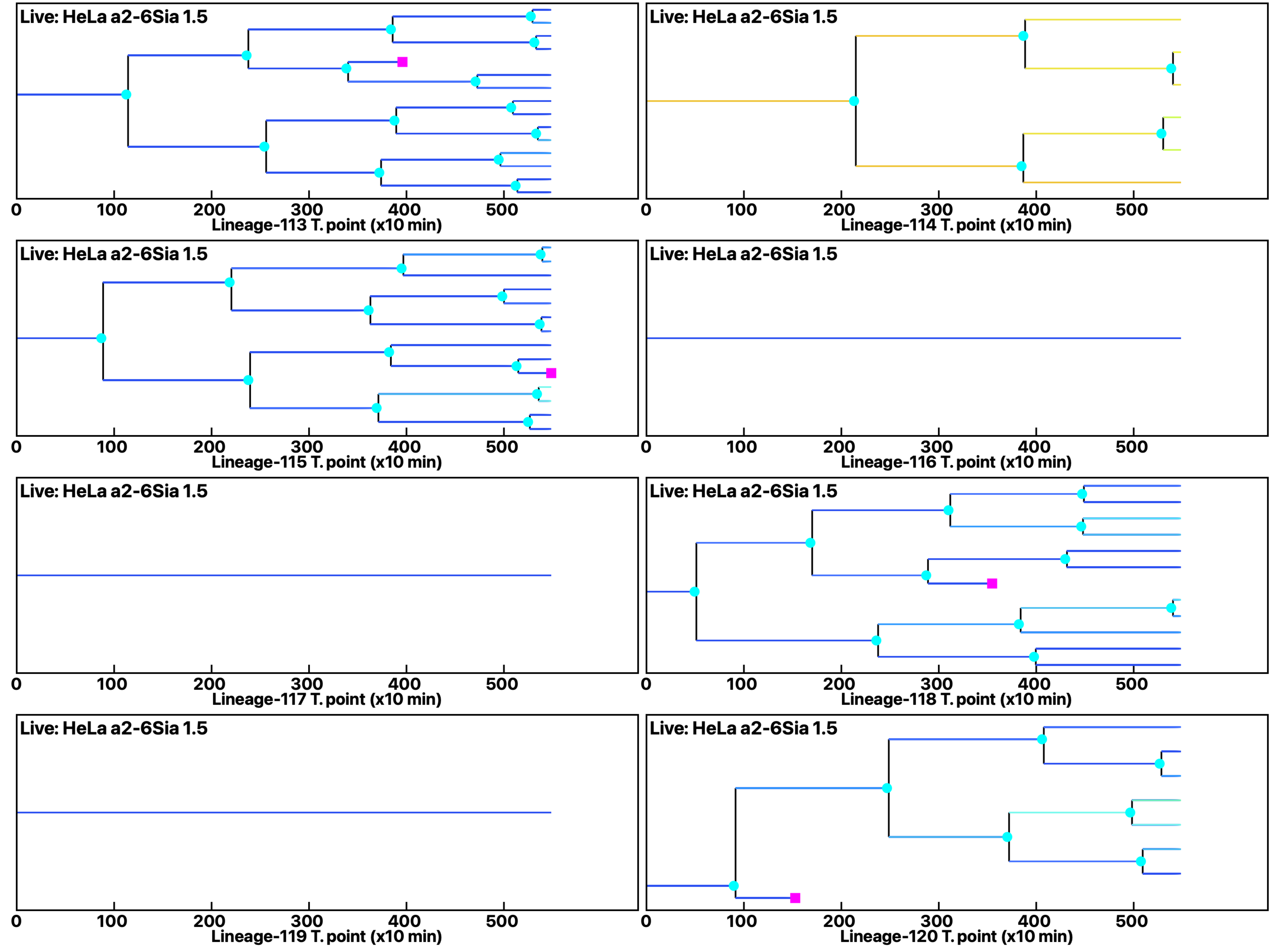

Analysis: Simulation, Treat.: HeLa a2-6Sia 1.5, Cell: HeLa-Simulation

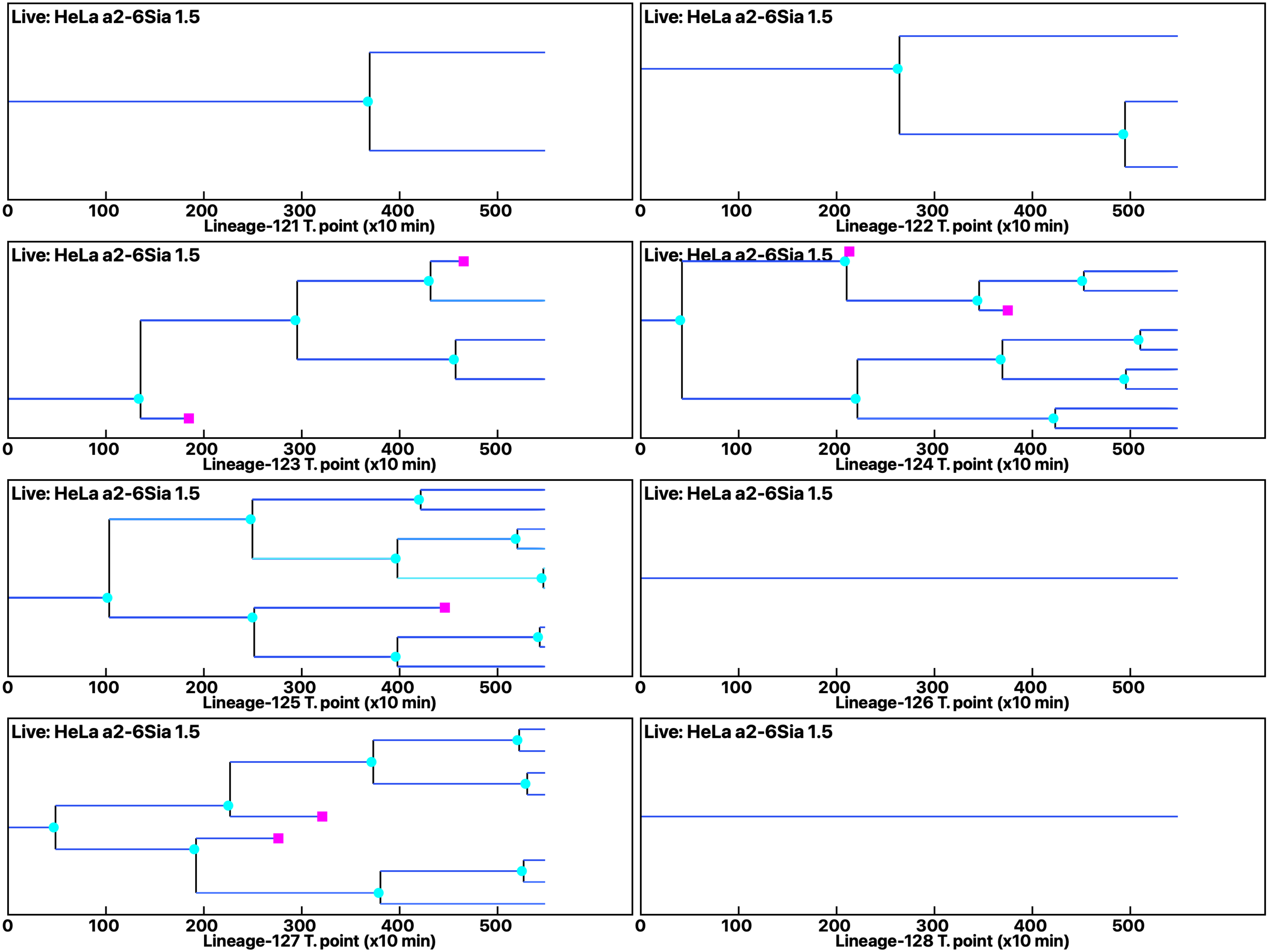

Analysis: Simulation, Treat.: HeLa a2-6Sia 1.5, Cell: HeLa-Simulation

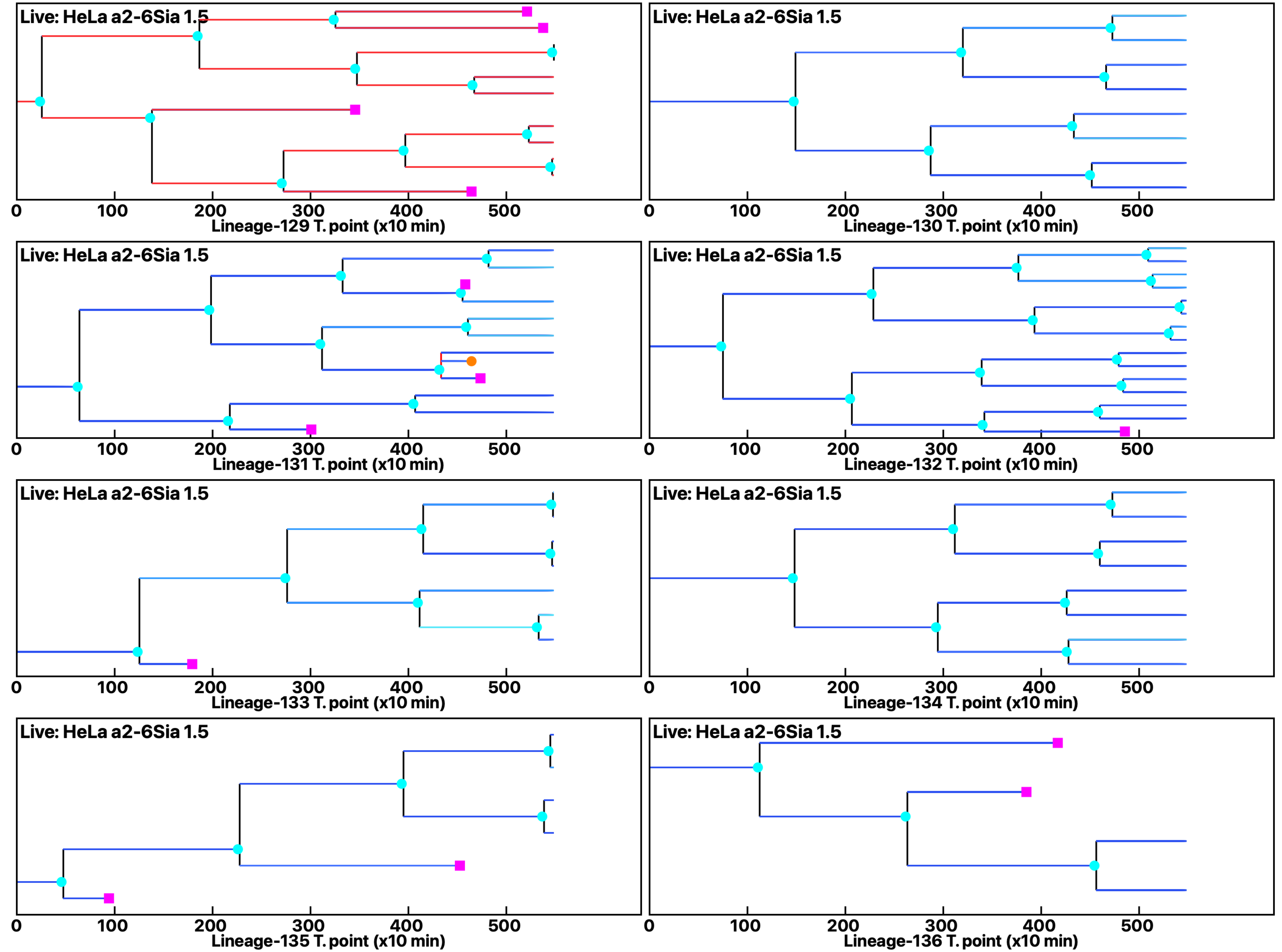

Analysis: Simulation, Treat.: HeLa a2-6Sia 1.5, Cell: HeLa-Simulation

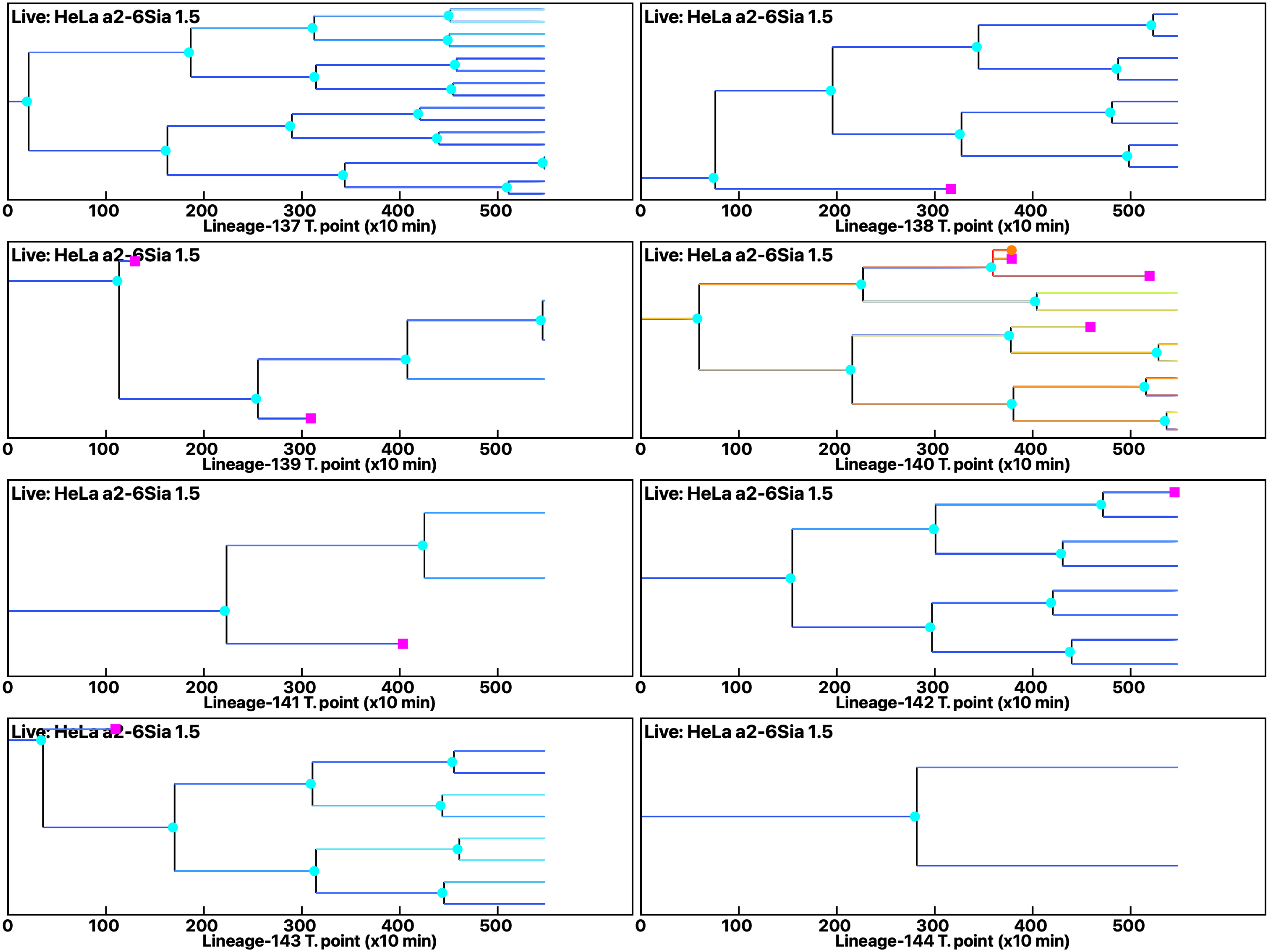

Analysis: Simulation, Treat.: HeLa a2-6Sia 1.5, Cell: HeLa-Simulation

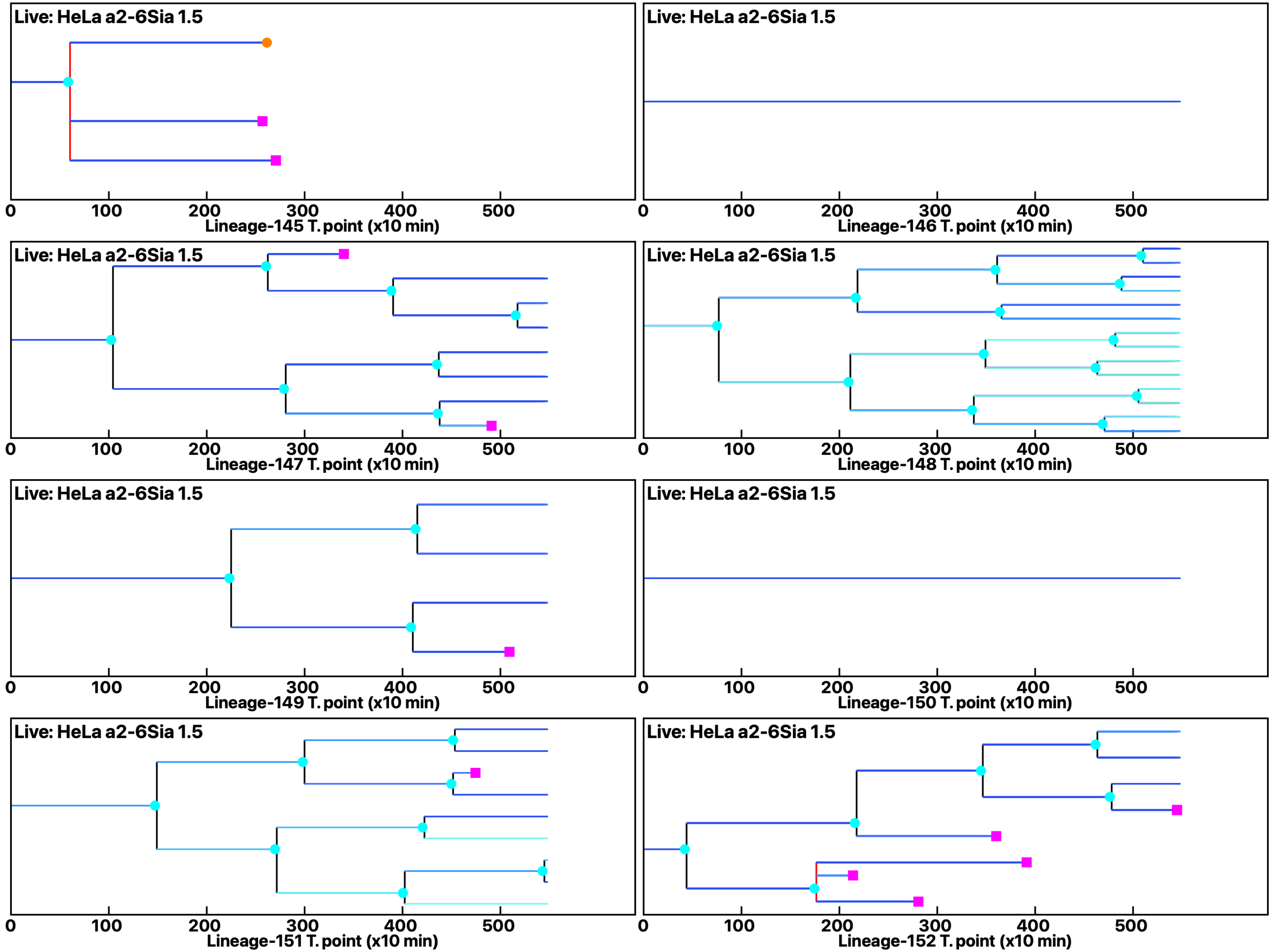

Analysis: Simulation, Treat.: HeLa a2-6Sia 1.5, Cell: HeLa-Simulation

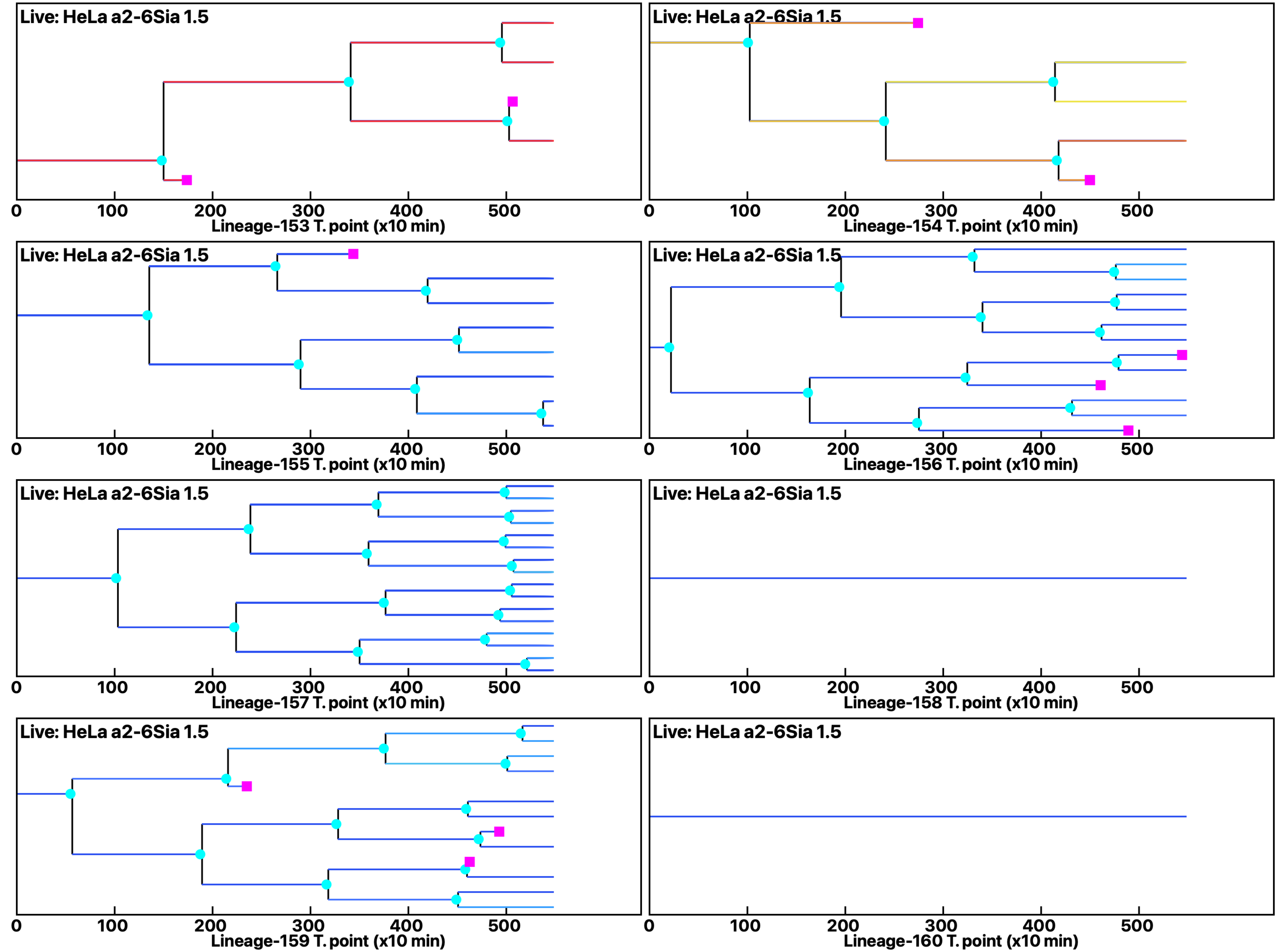

Analysis: Simulation, Treat.: HeLa a2-6Sia 1.5, Cell: HeLa-Simulation

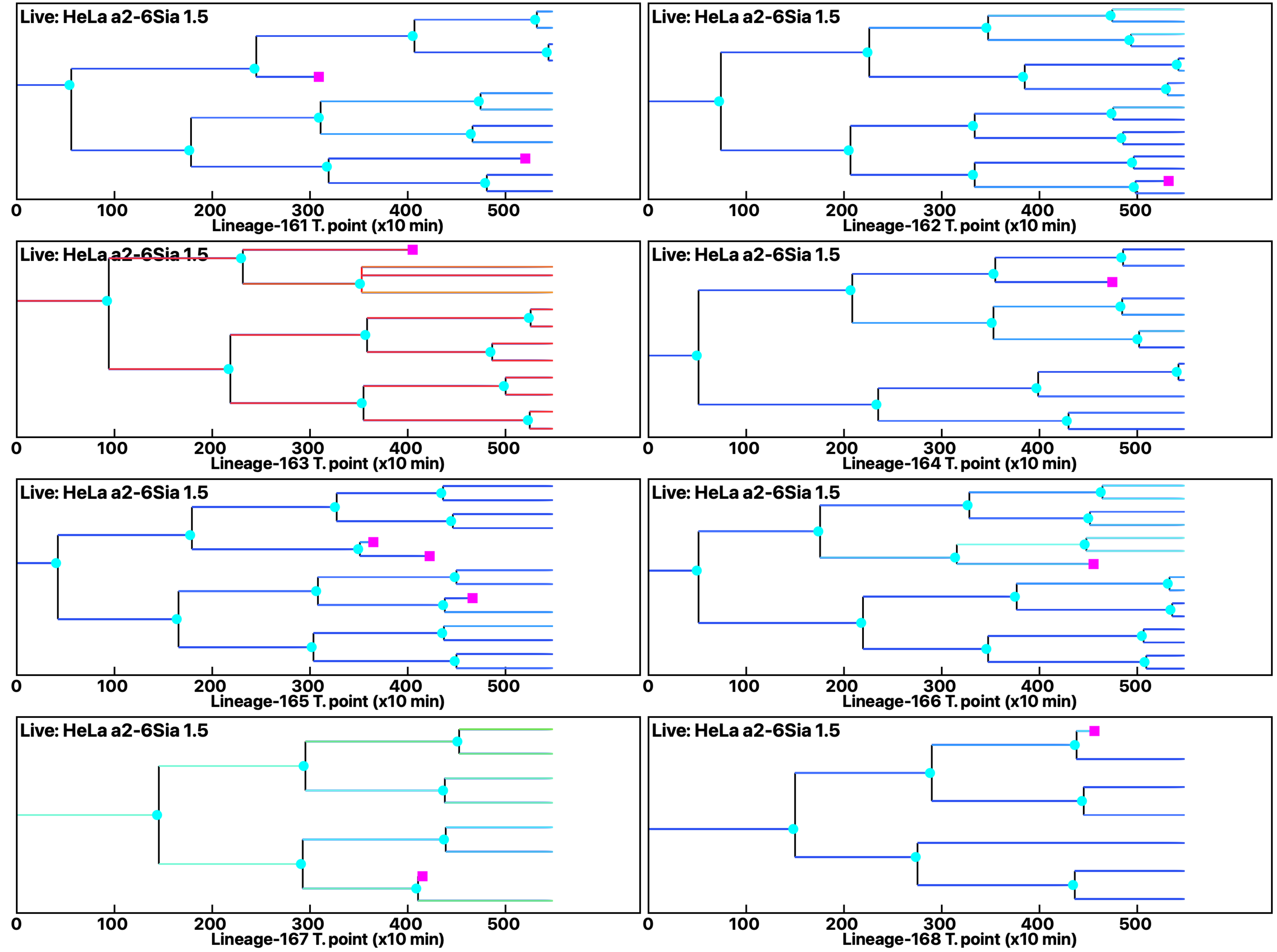

Analysis: Simulation, Treat.: HeLa a2-6Sia 1.5, Cell: HeLa-Simulation

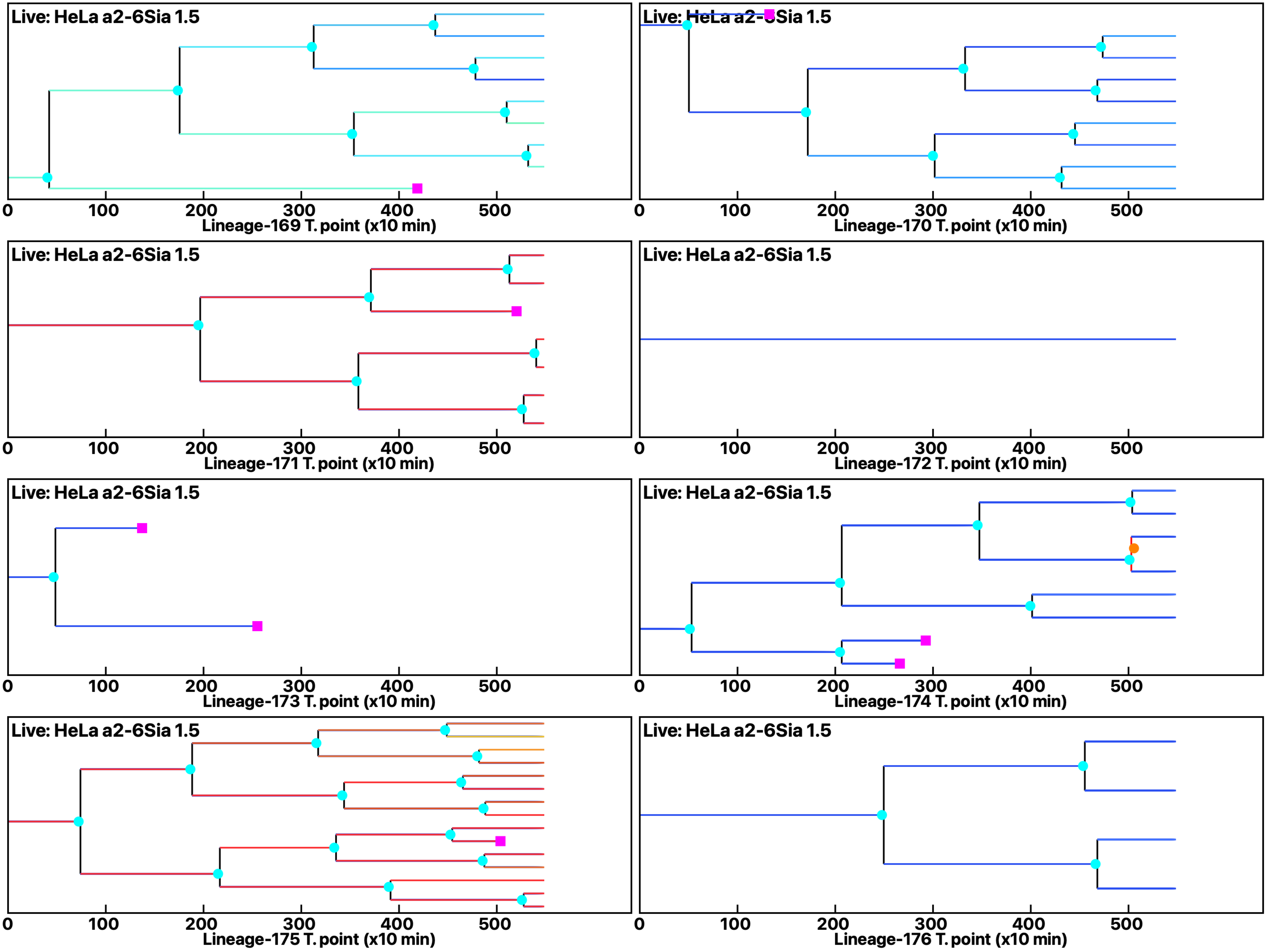

Analysis: Simulation, Treat.: HeLa a2-6Sia 1.5, Cell: HeLa-Simulation

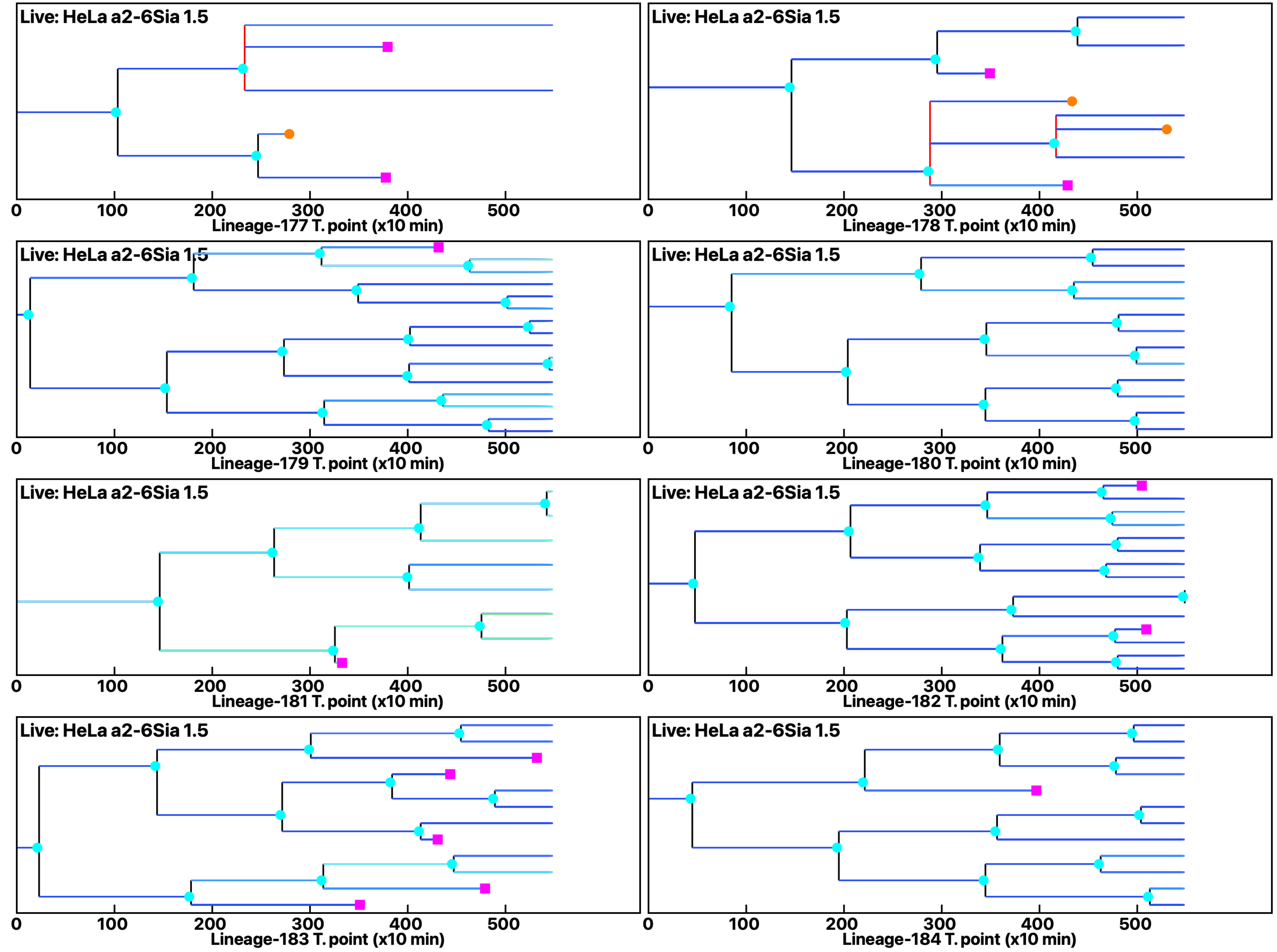

Analysis: Simulation, Treat.: HeLa a2-6Sia 1.5, Cell: HeLa-Simulation

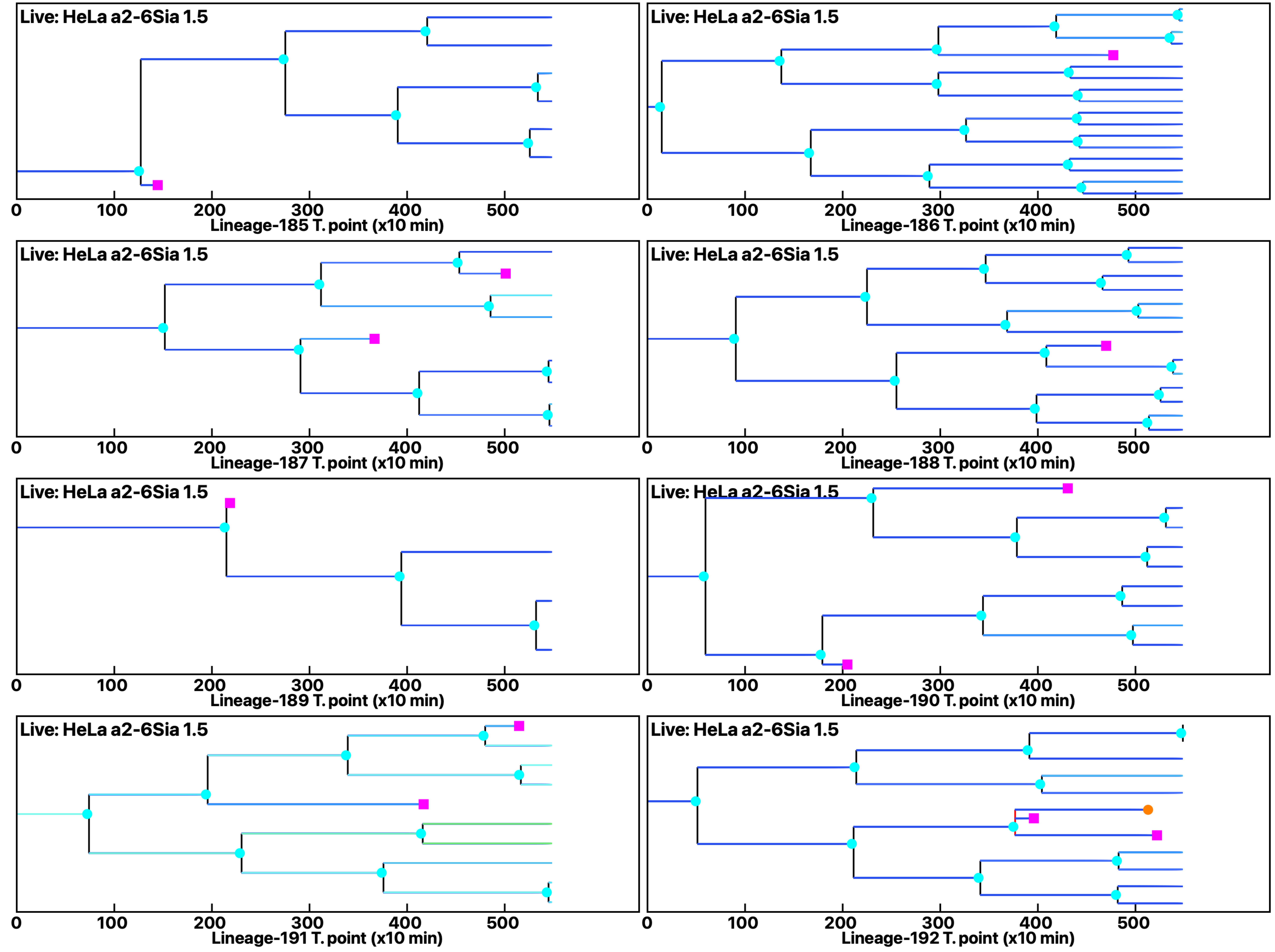

Analysis: Simulation, Treat.: HeLa a2-6Sia 1.5, Cell: HeLa-Simulation

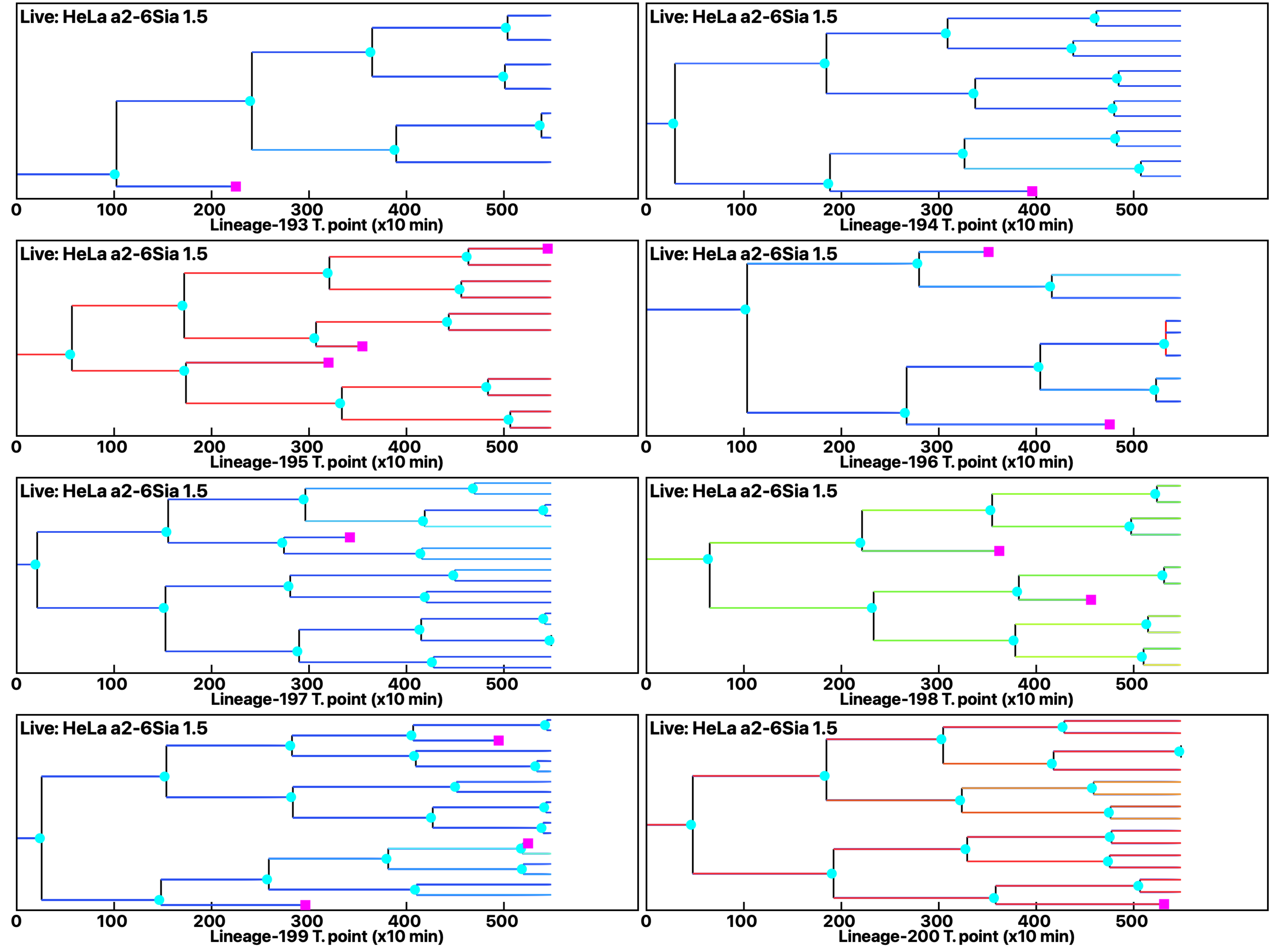

Analysis: Simulation, Treat.: HeLa a2-6Sia 1.5, Cell: HeLa-Simulation

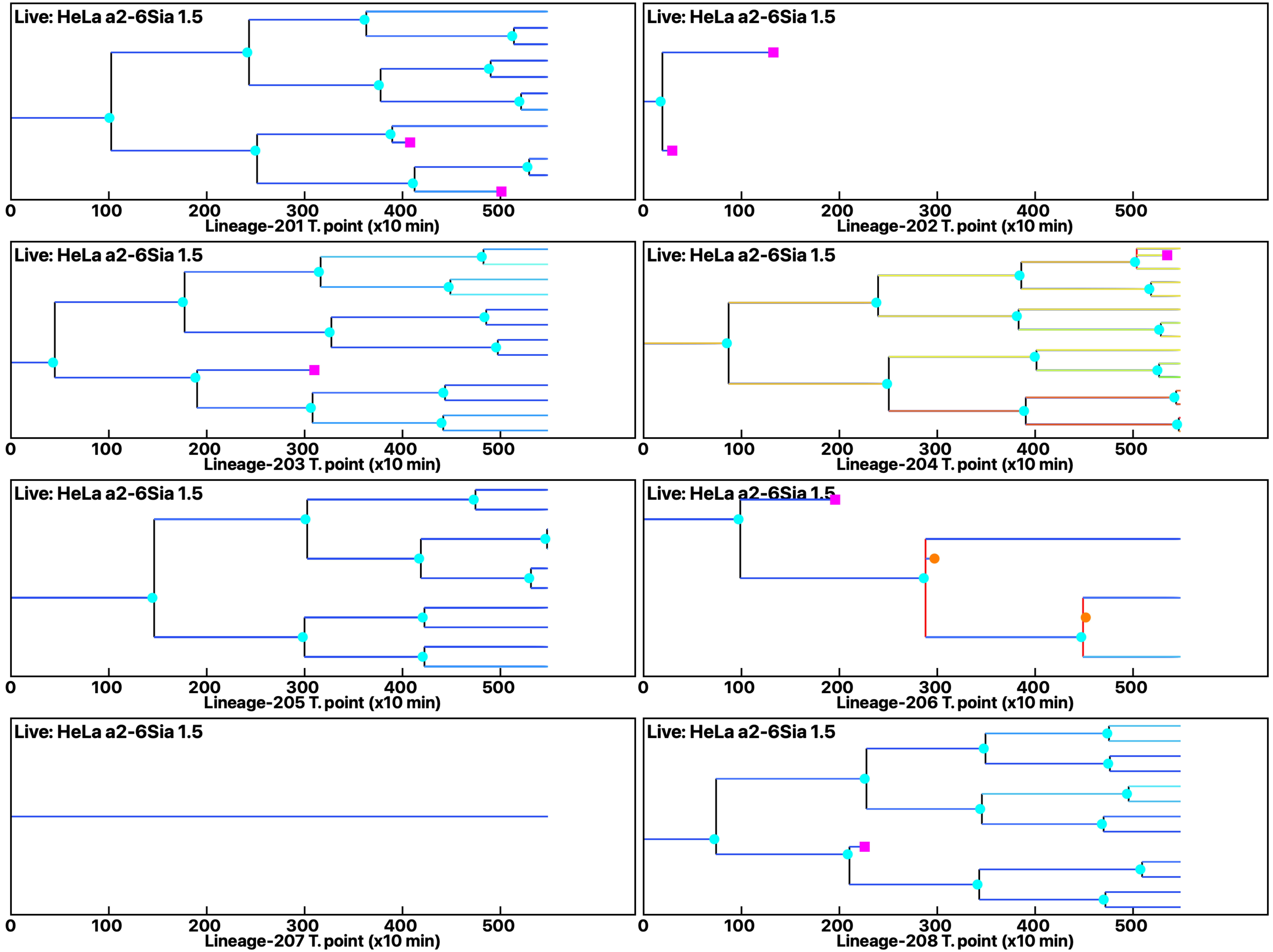

Analysis: Simulation, Treat.: HeLa a2-6Sia 1.5, Cell: HeLa-Simulation

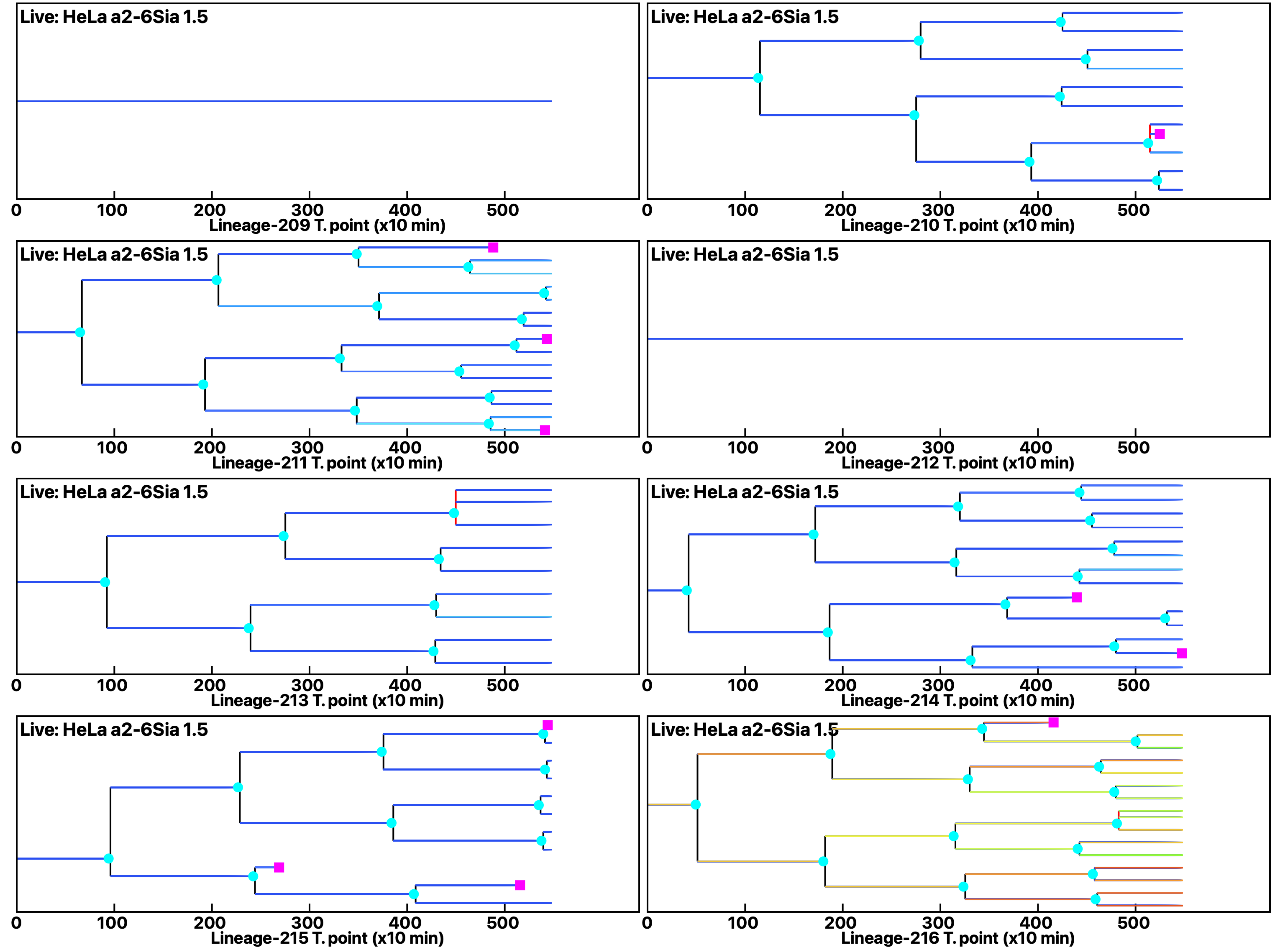

Analysis: Simulation, Treat.: HeLa a2-6Sia 1.5, Cell: HeLa-Simulation

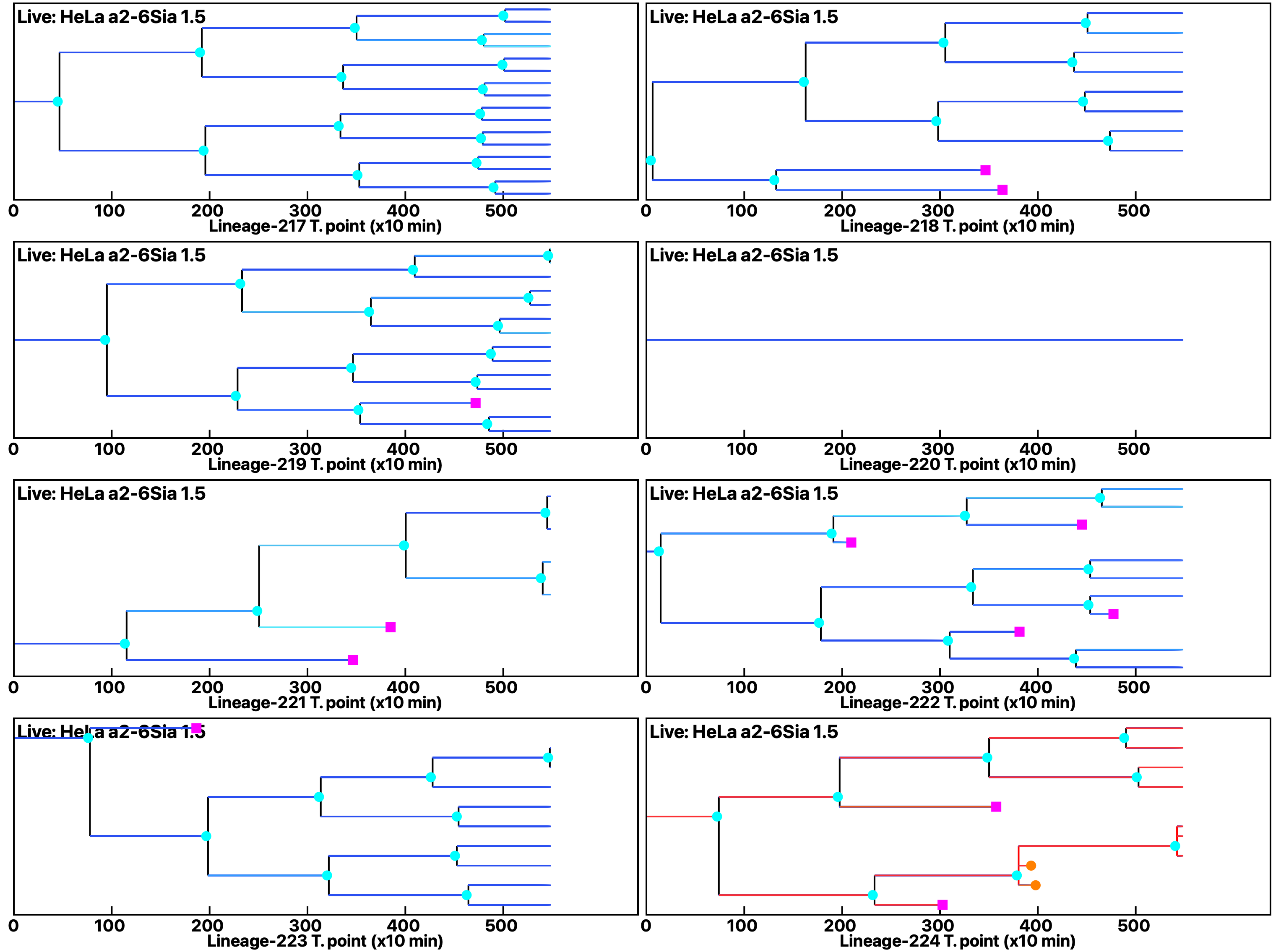

Analysis: Simulation, Treat.: HeLa a2-6Sia 1.5, Cell: HeLa-Simulation

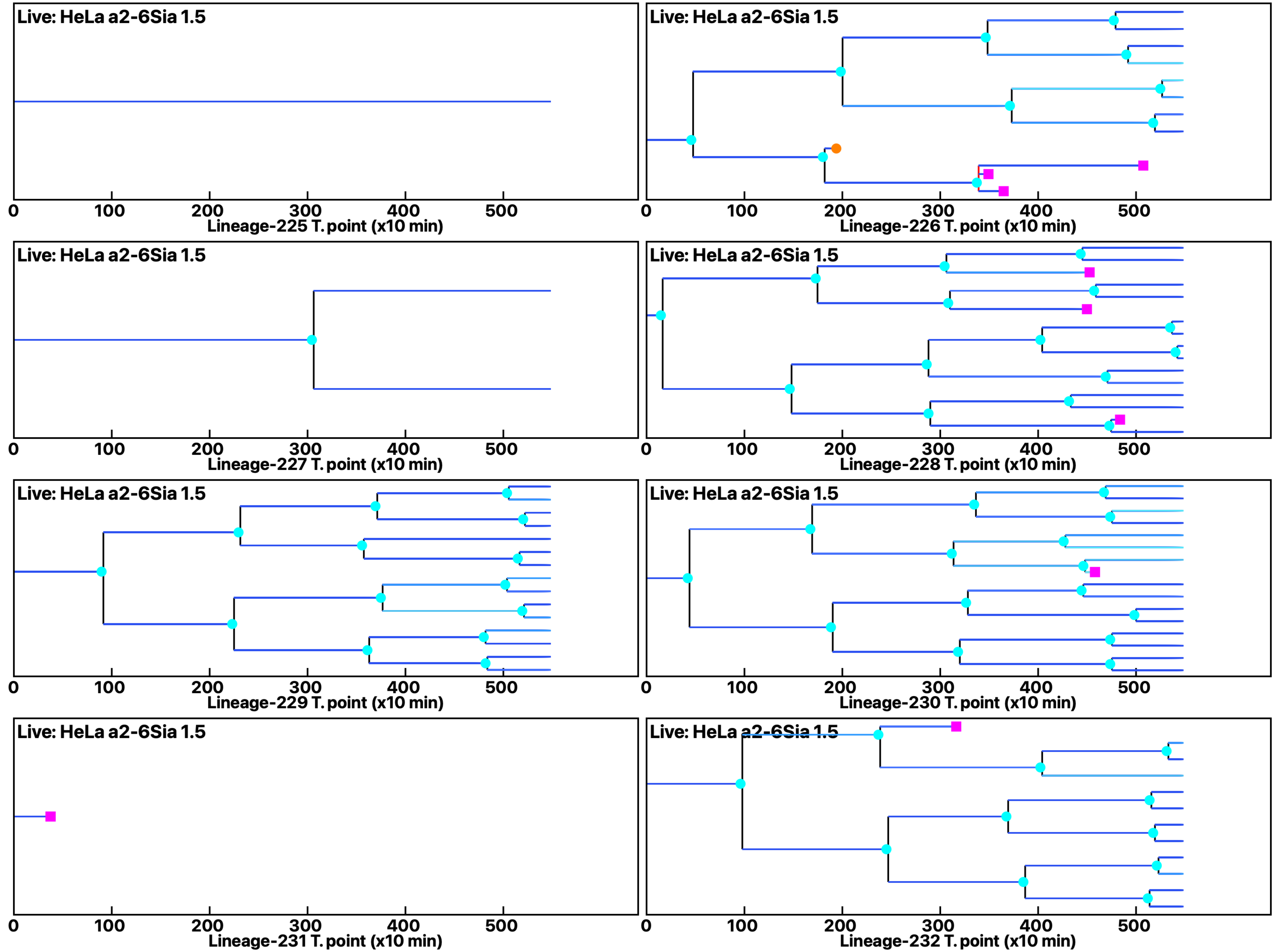

Analysis: Simulation, Treat.: HeLa a2-6Sia 1.5, Cell: HeLa-Simulation

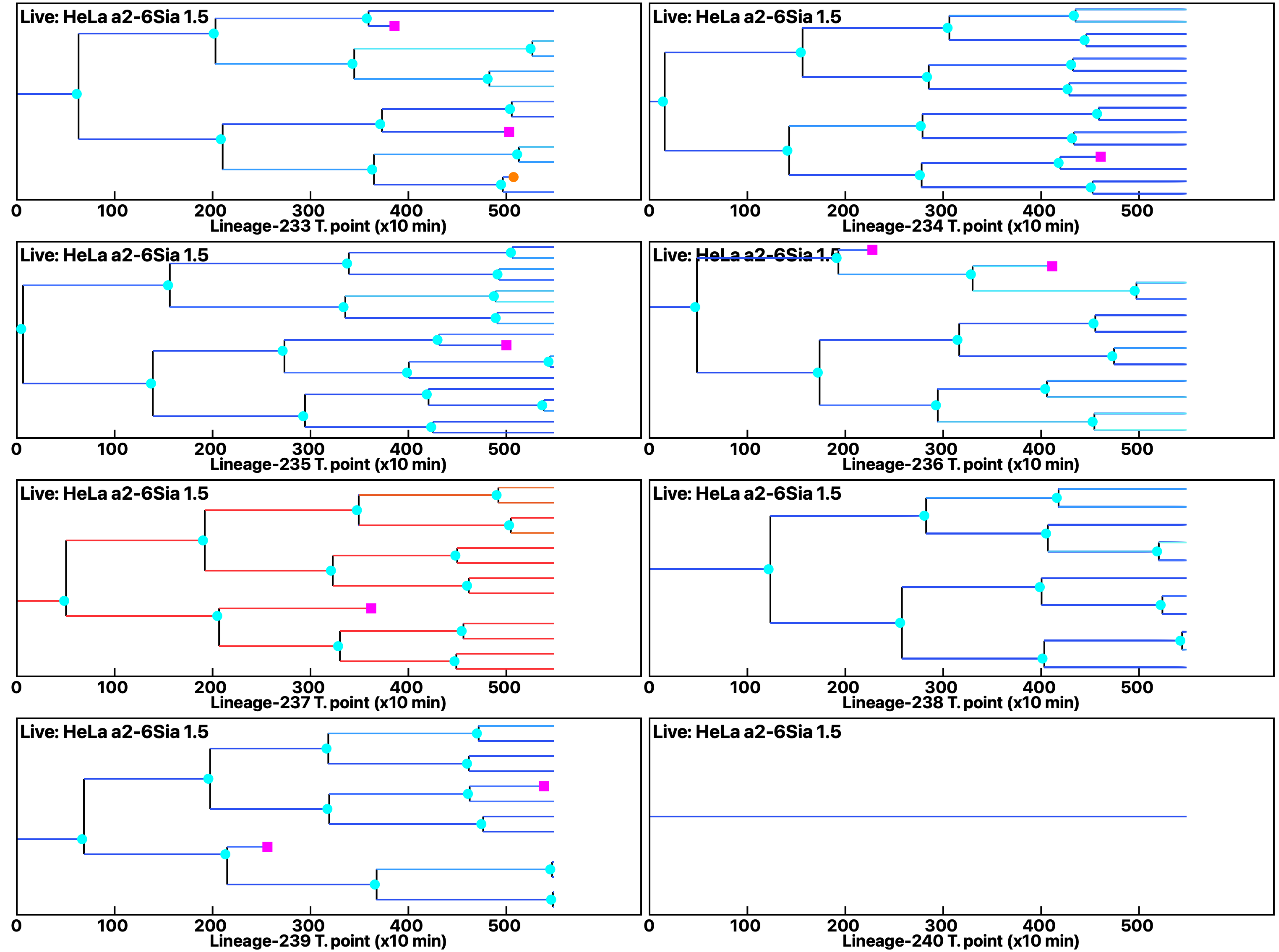

Analysis: Simulation, Treat.: HeLa a2-6Sia 1.5, Cell: HeLa-Simulation

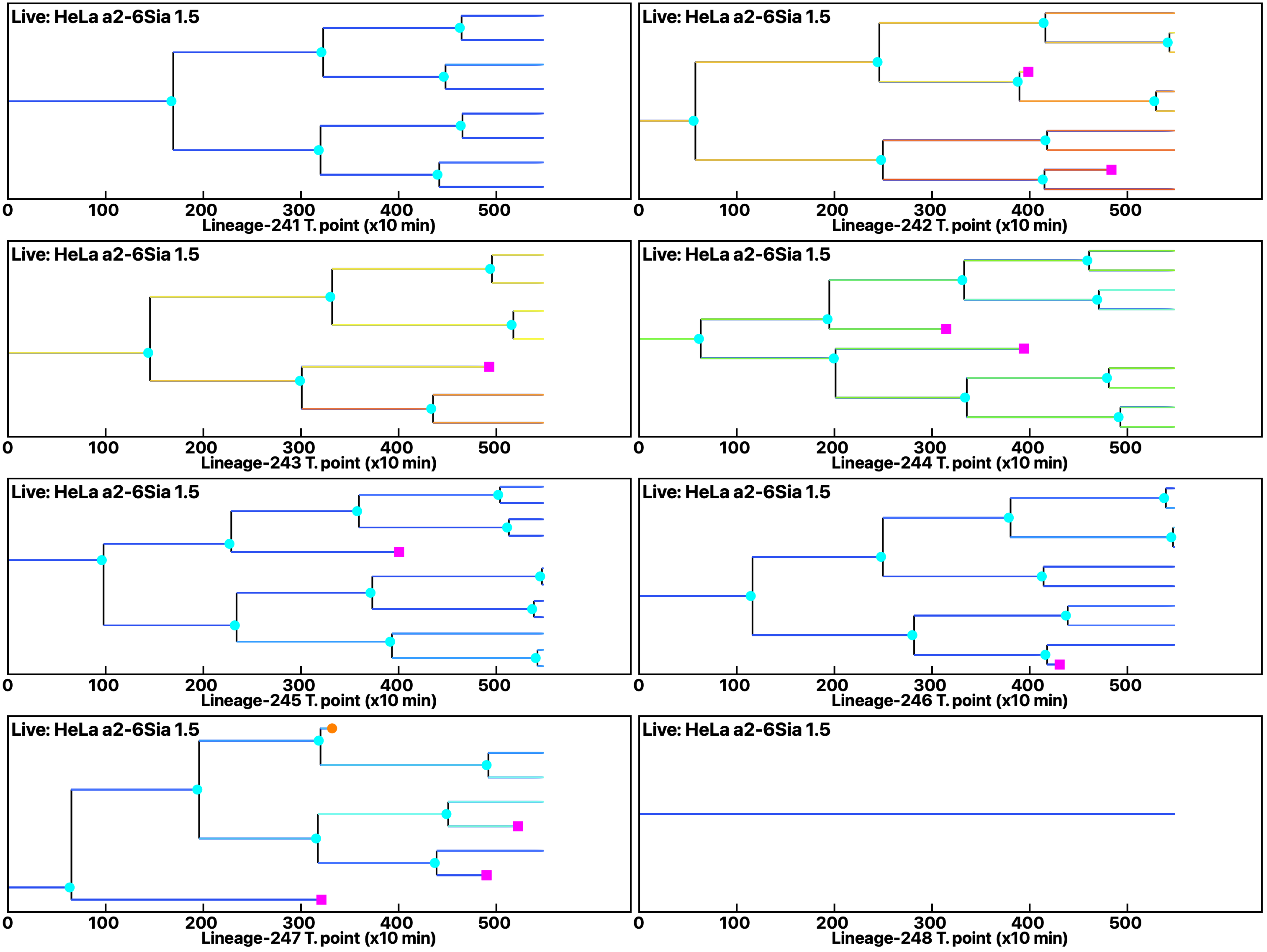

Analysis: Simulation, Treat.: HeLa a2-6Sia 1.5, Cell: HeLa-Simulation

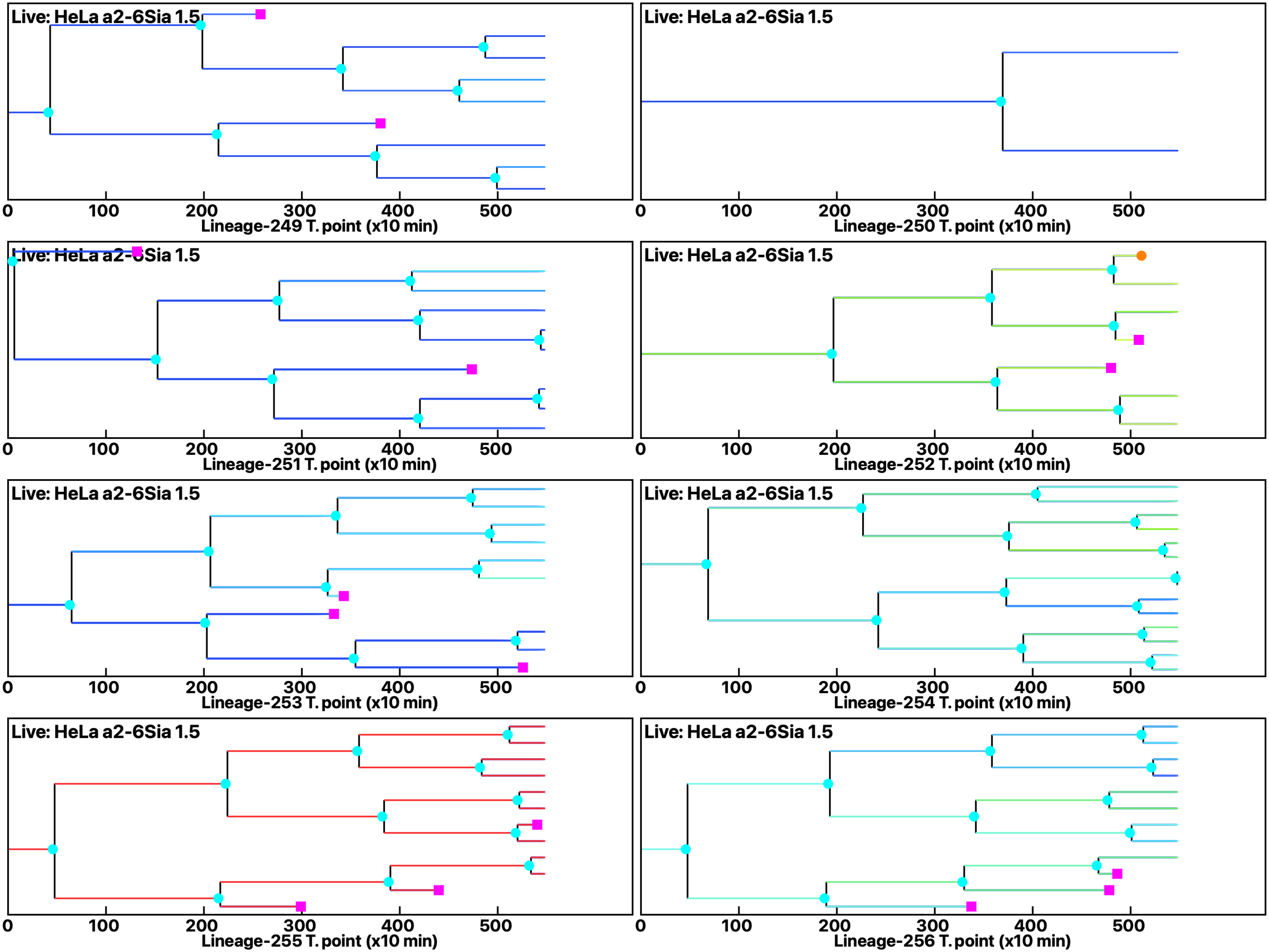

Analysis: Simulation, Treat.: HeLa a2-6Sia 1.5, Cell: HeLa-Simulation

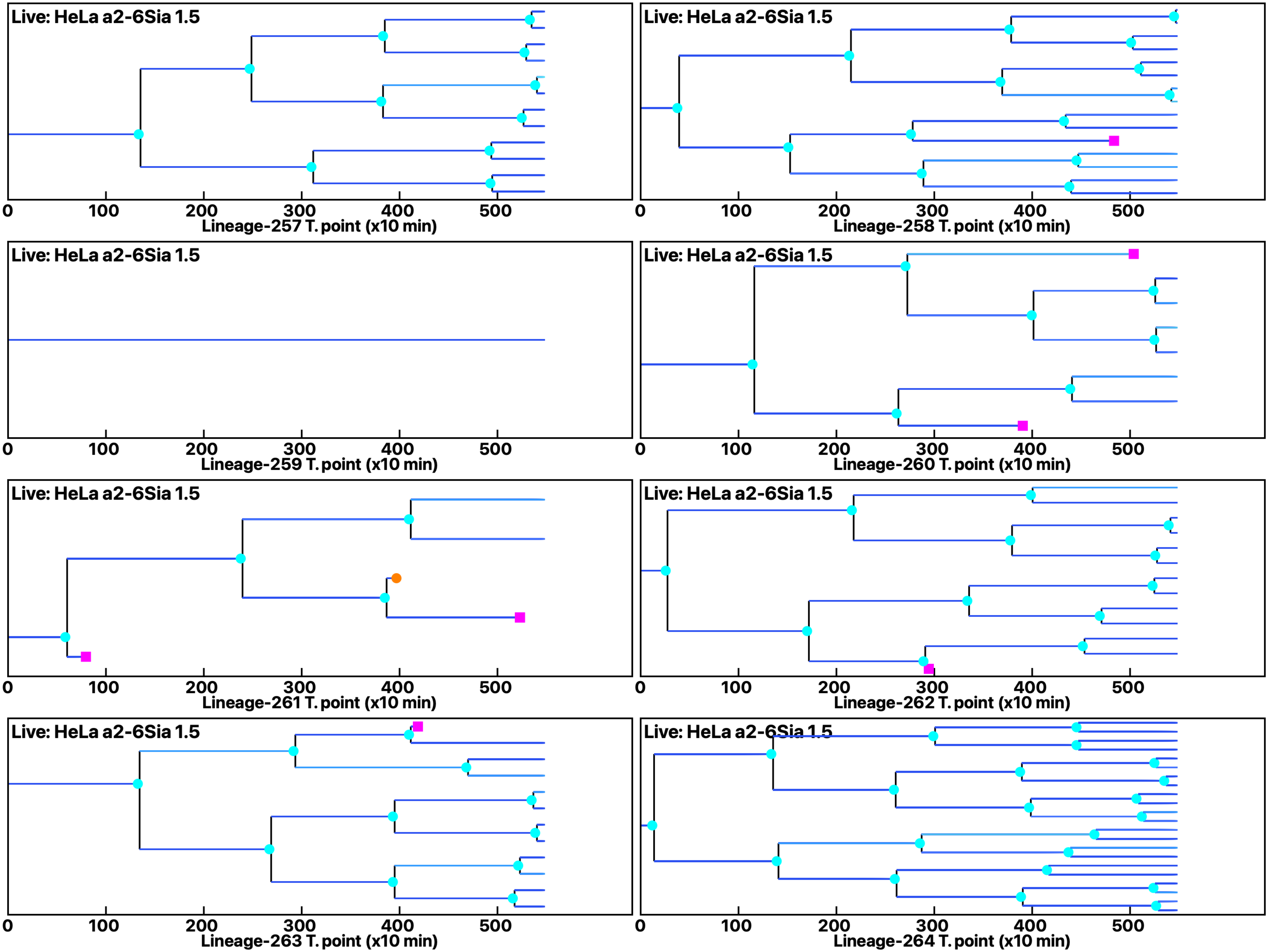

Analysis: Simulation, Treat.: HeLa a2-6Sia 1.5, Cell: HeLa-Simulation

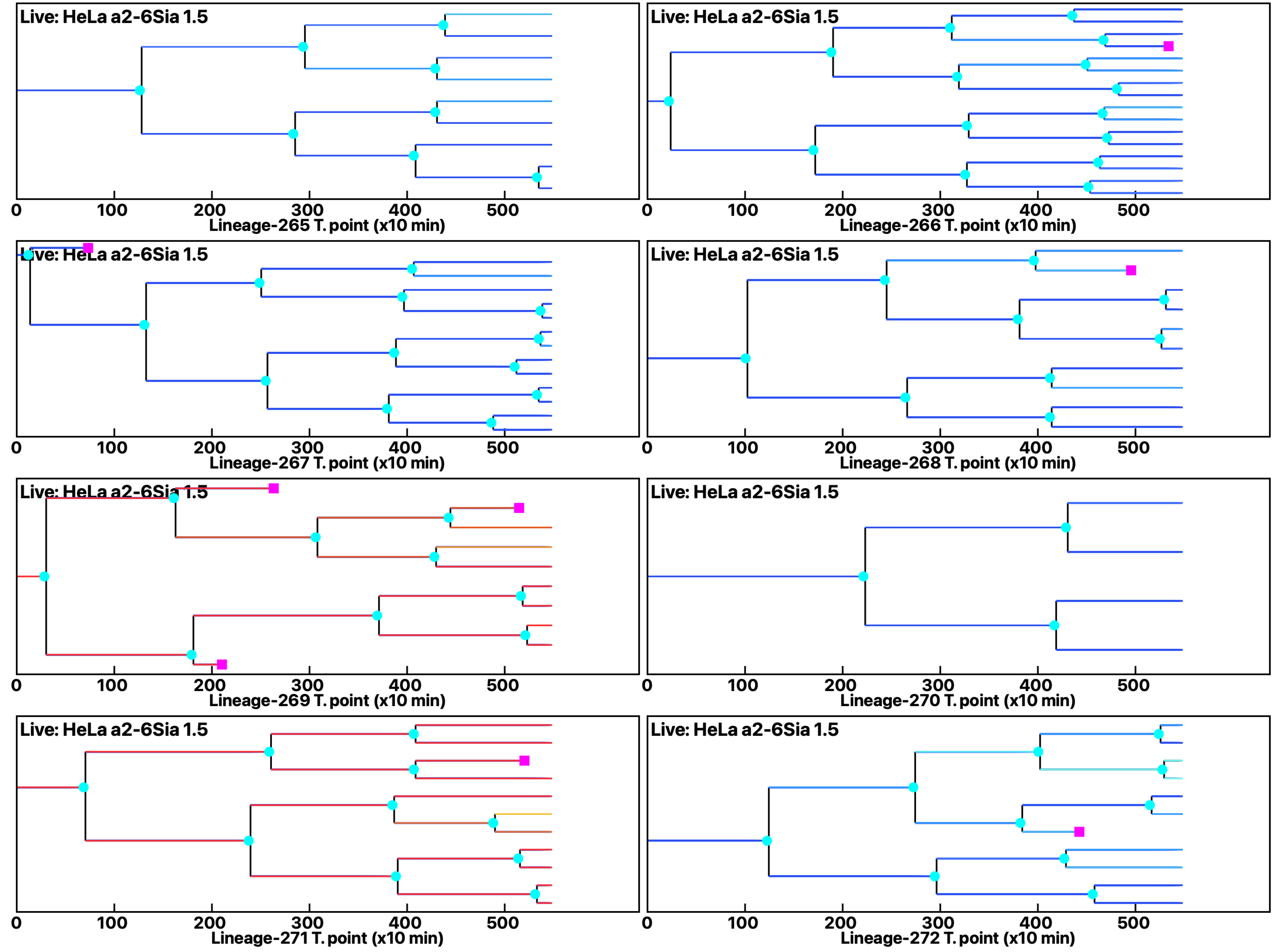

Analysis: Simulation, Treat.: HeLa a2-6Sia 1.5, Cell: HeLa-Simulation

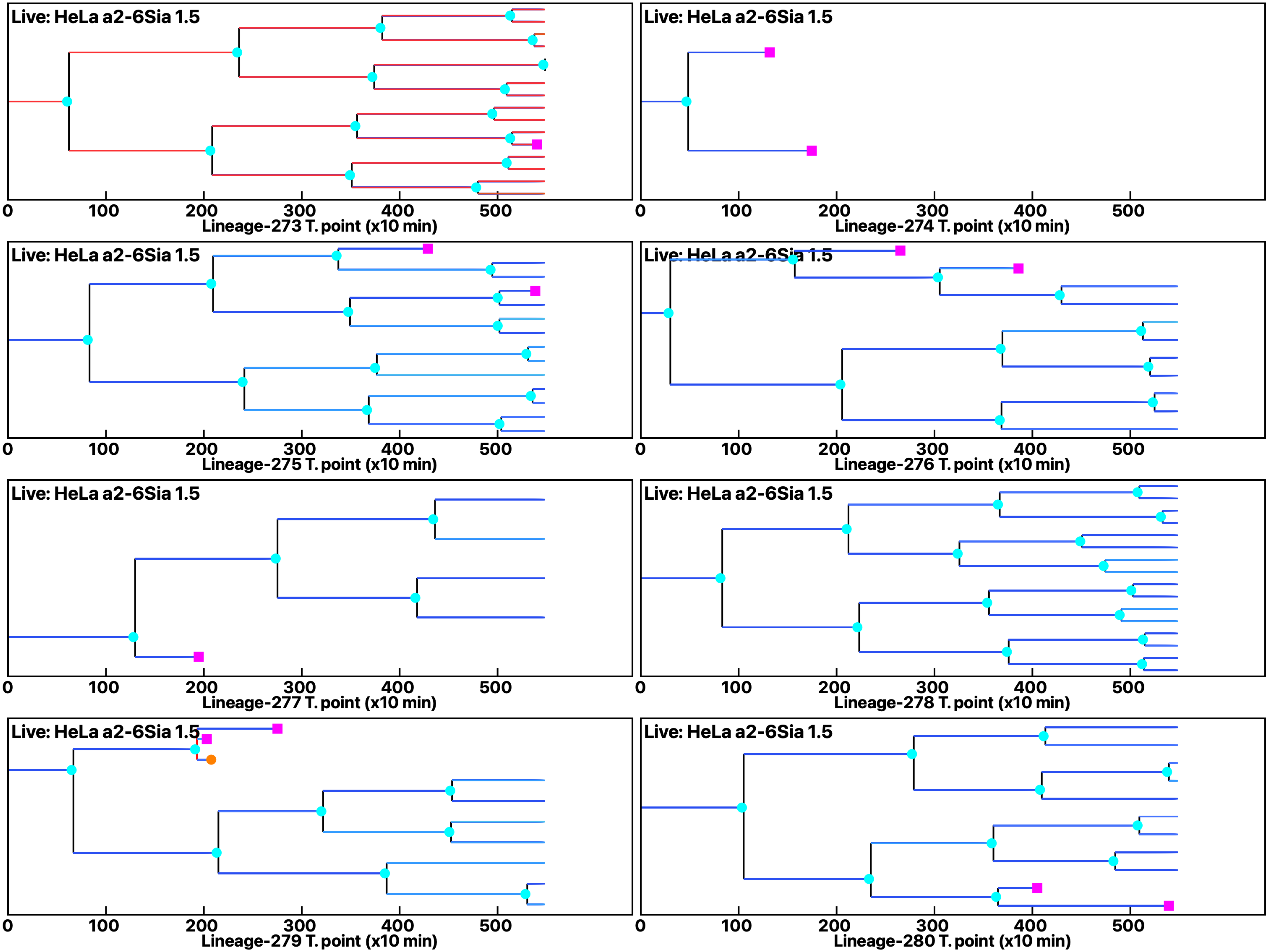

Analysis: Simulation, Treat.: HeLa a2-6Sia 1.5, Cell: HeLa-Simulation

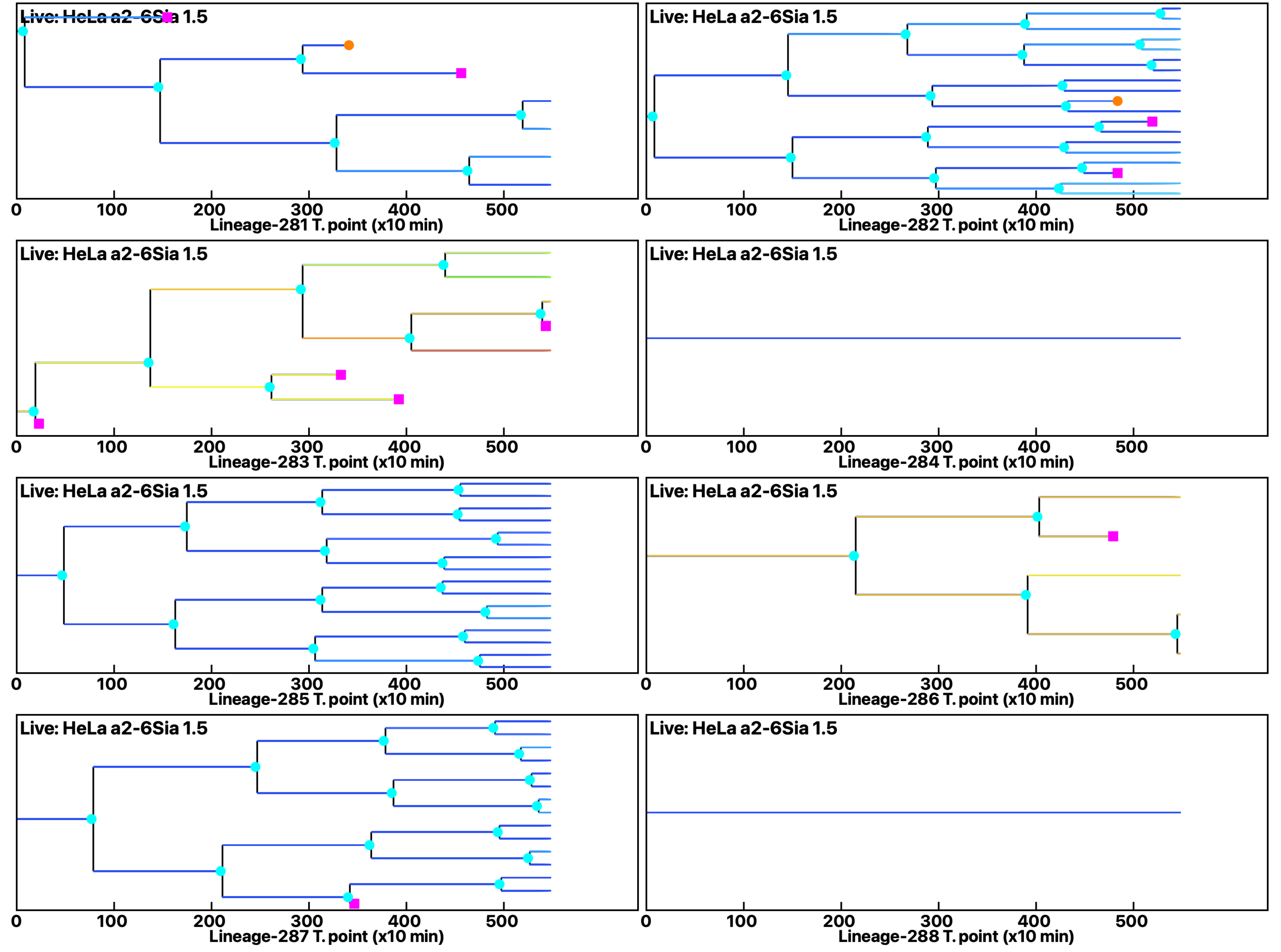

Analysis: Simulation, Treat.: HeLa a2-6Sia 1.5, Cell: HeLa-Simulation

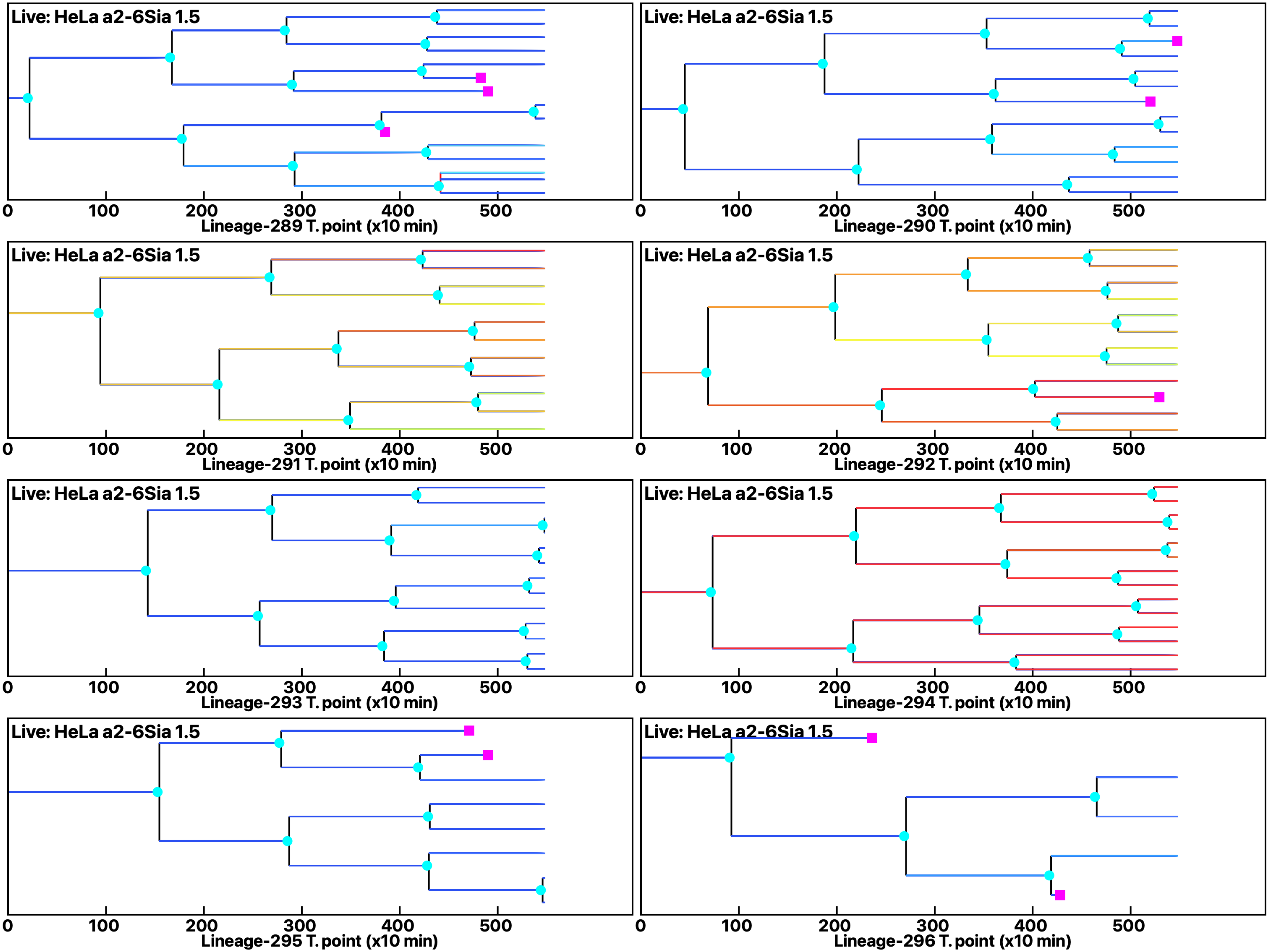

Analysis: Simulation, Treat.: HeLa a2-6Sia 1.5, Cell: HeLa-Simulation

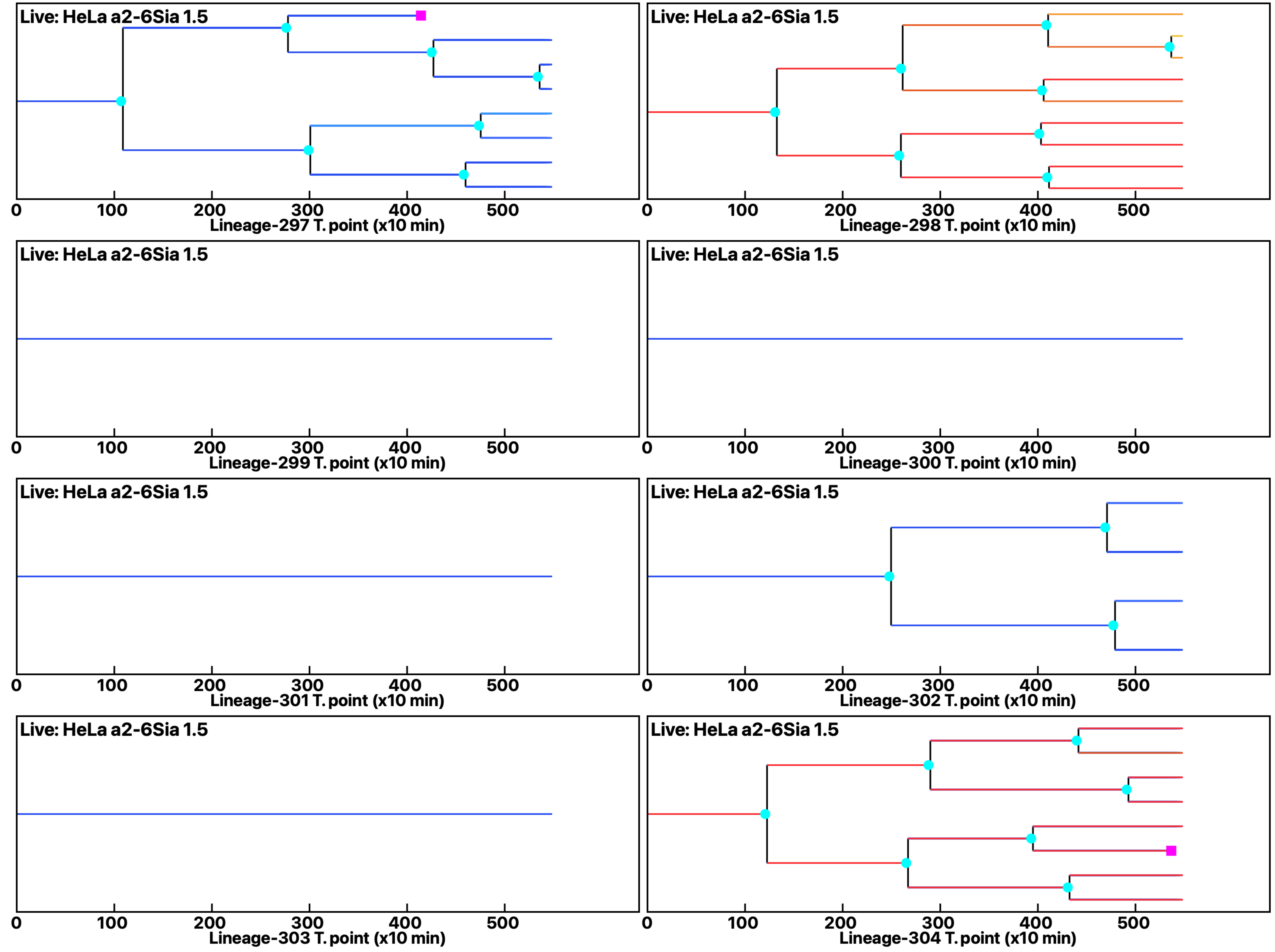

Analysis: Simulation, Treat.: HeLa a2-6Sia 1.5, Cell: HeLa-Simulation

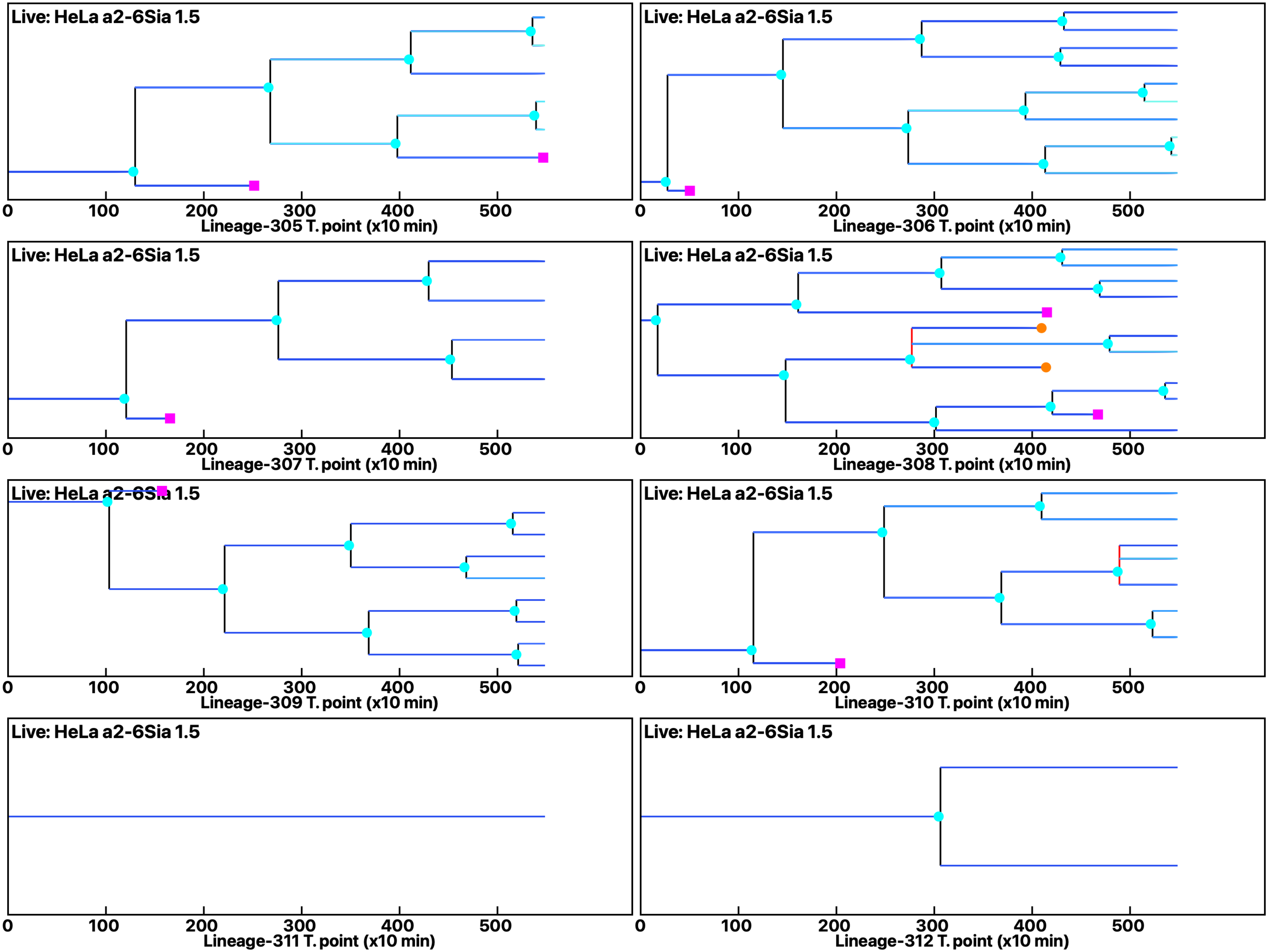

Analysis: Simulation, Treat.: HeLa a2-6Sia 1.5, Cell: HeLa-Simulation

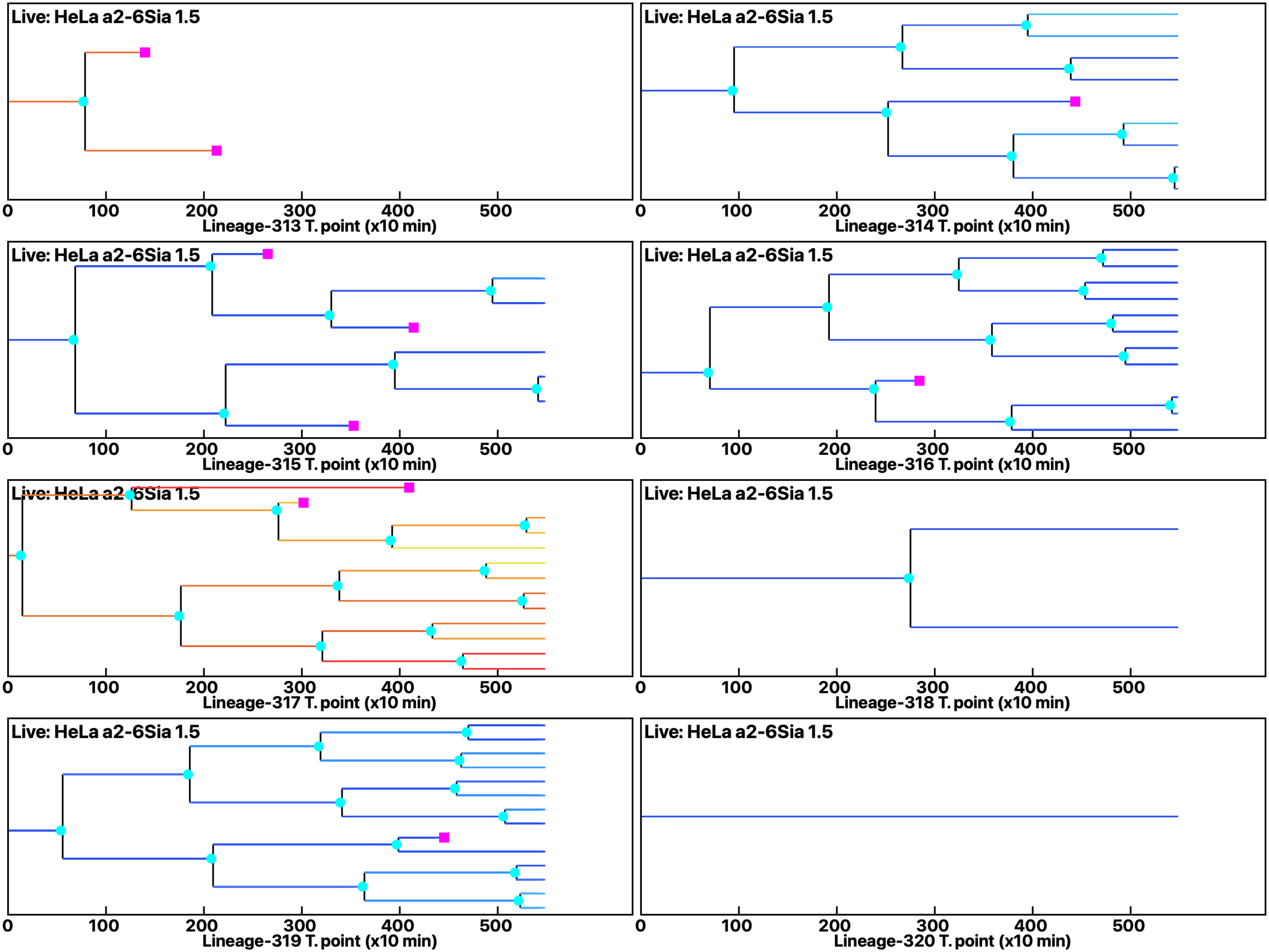

Analysis: Simulation, Treat.: HeLa a2-6Sia 1.5, Cell: HeLa-Simulation

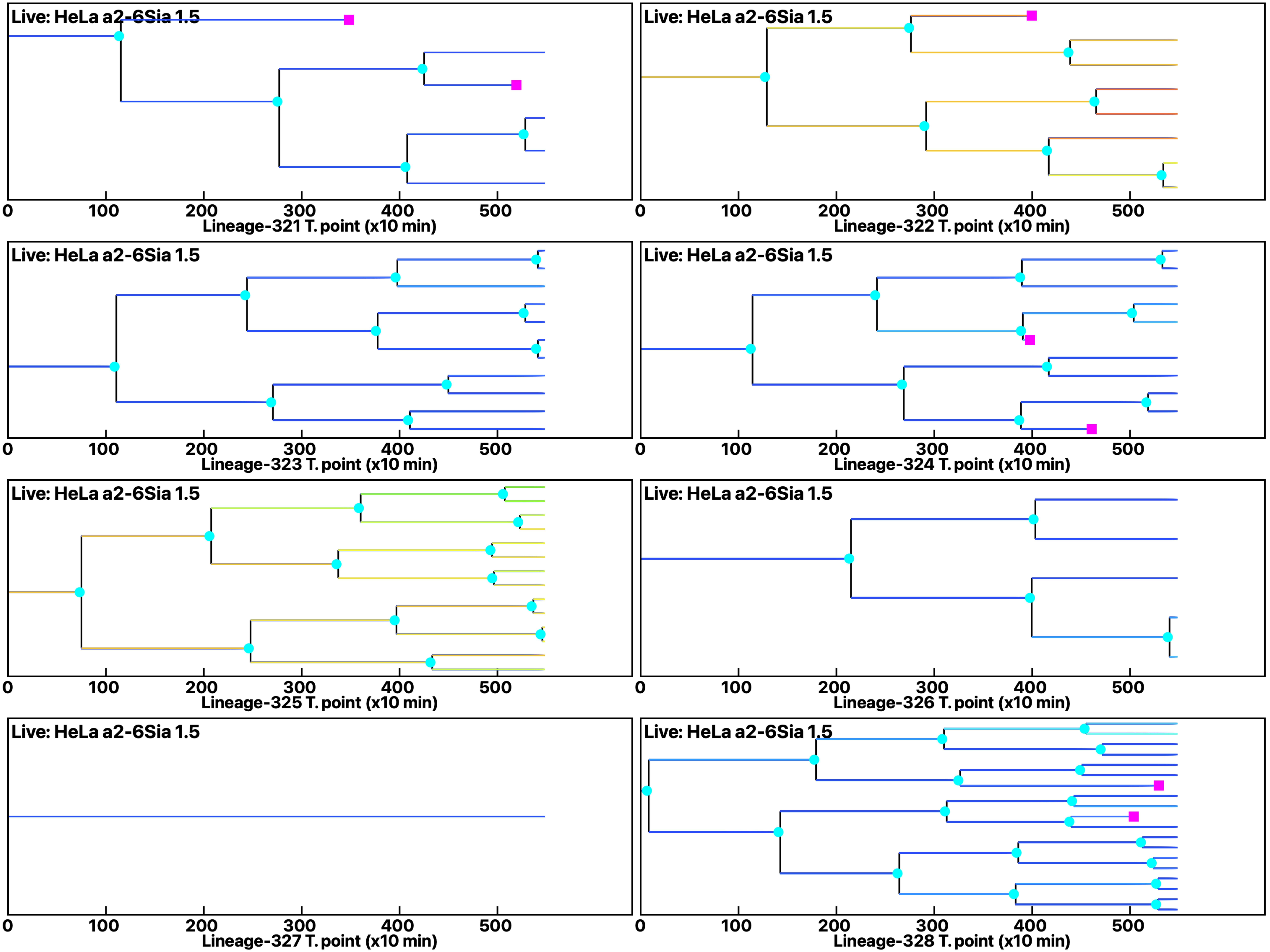

Analysis: Simulation, Treat.: HeLa a2-6Sia 1.5, Cell: HeLa-Simulation

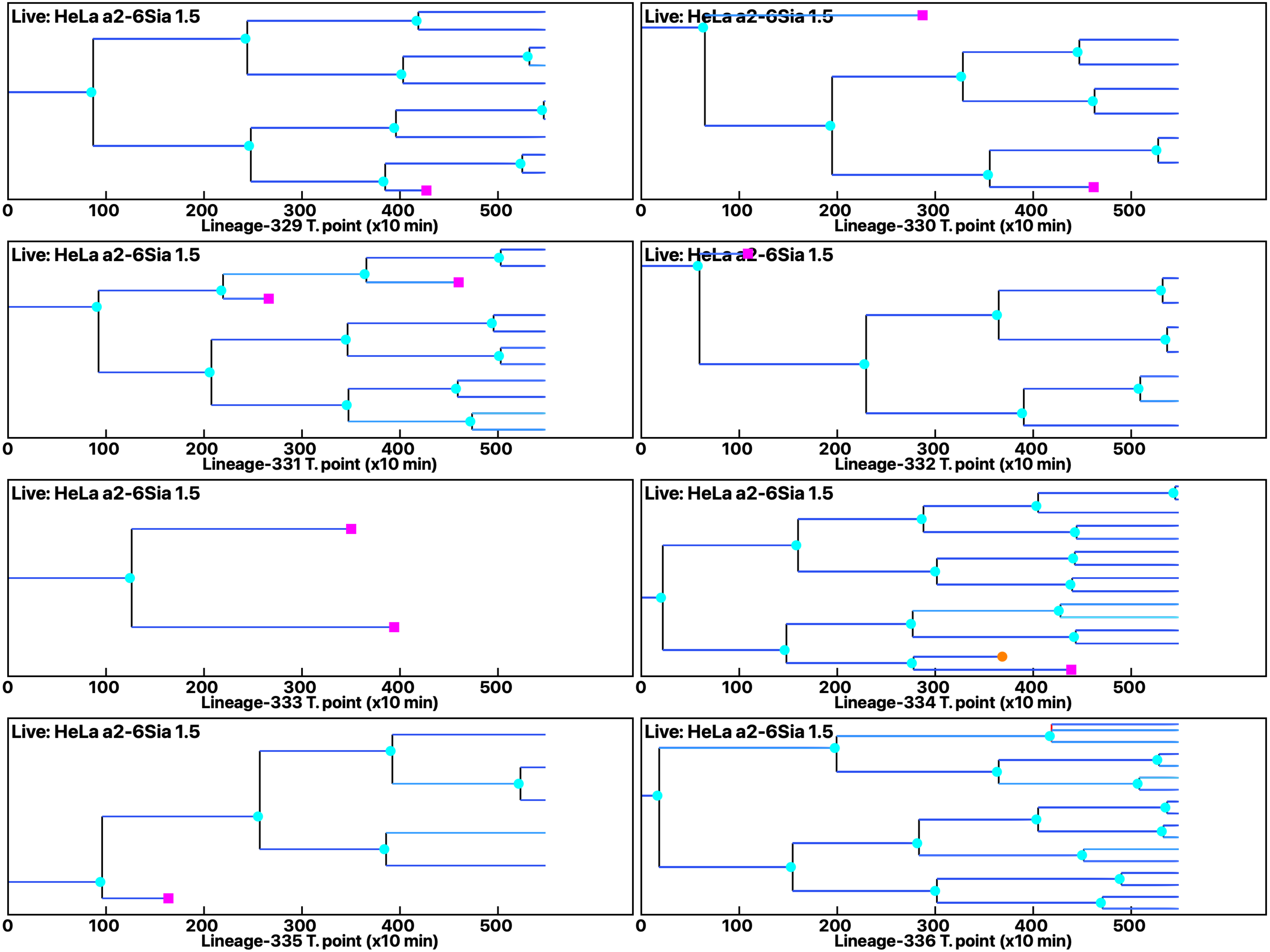

Analysis: Simulation, Treat.: HeLa a2-6Sia 1.5, Cell: HeLa-Simulation

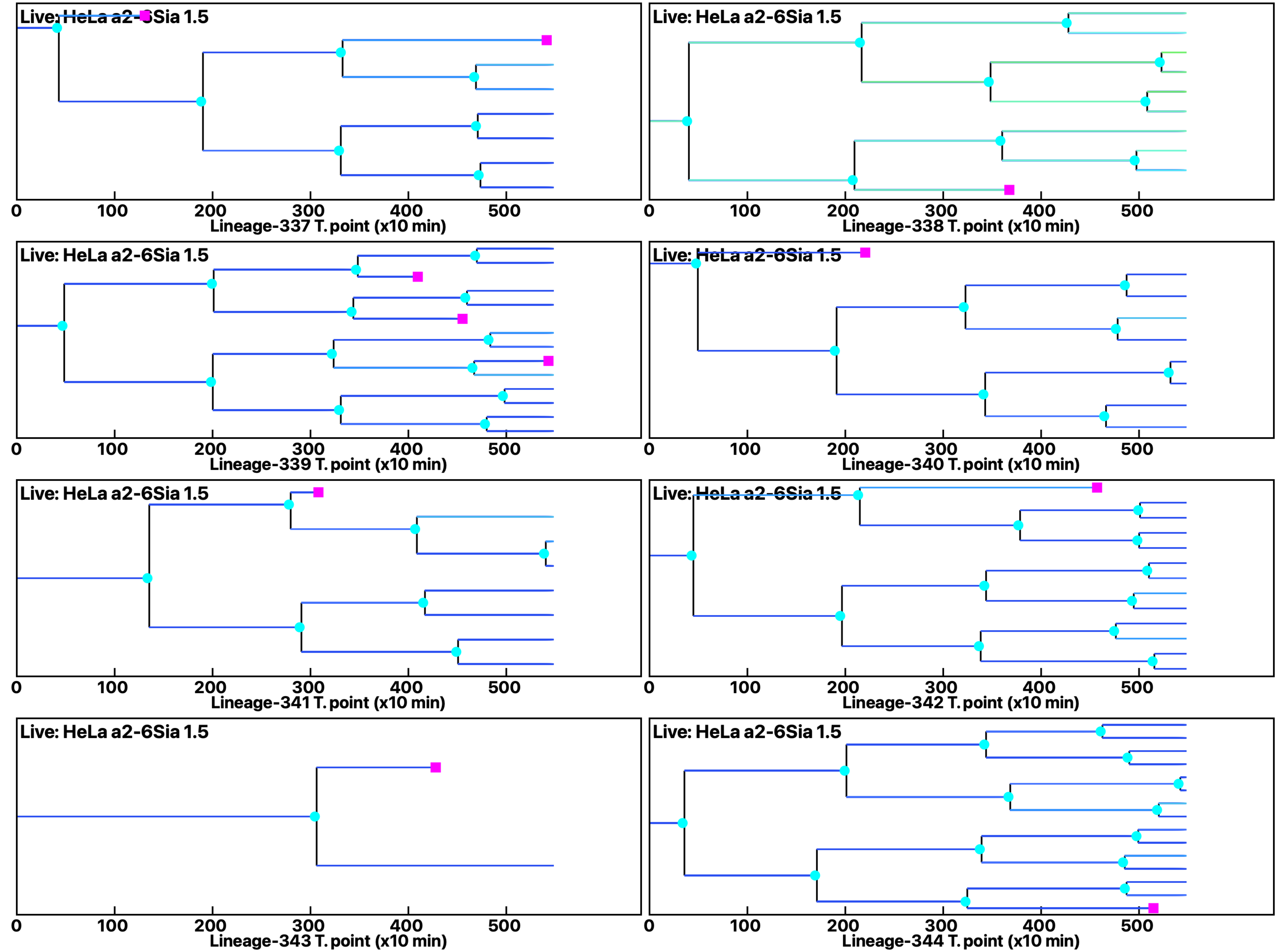

Analysis: Simulation, Treat.: HeLa a2-6Sia 1.5, Cell: HeLa-Simulation

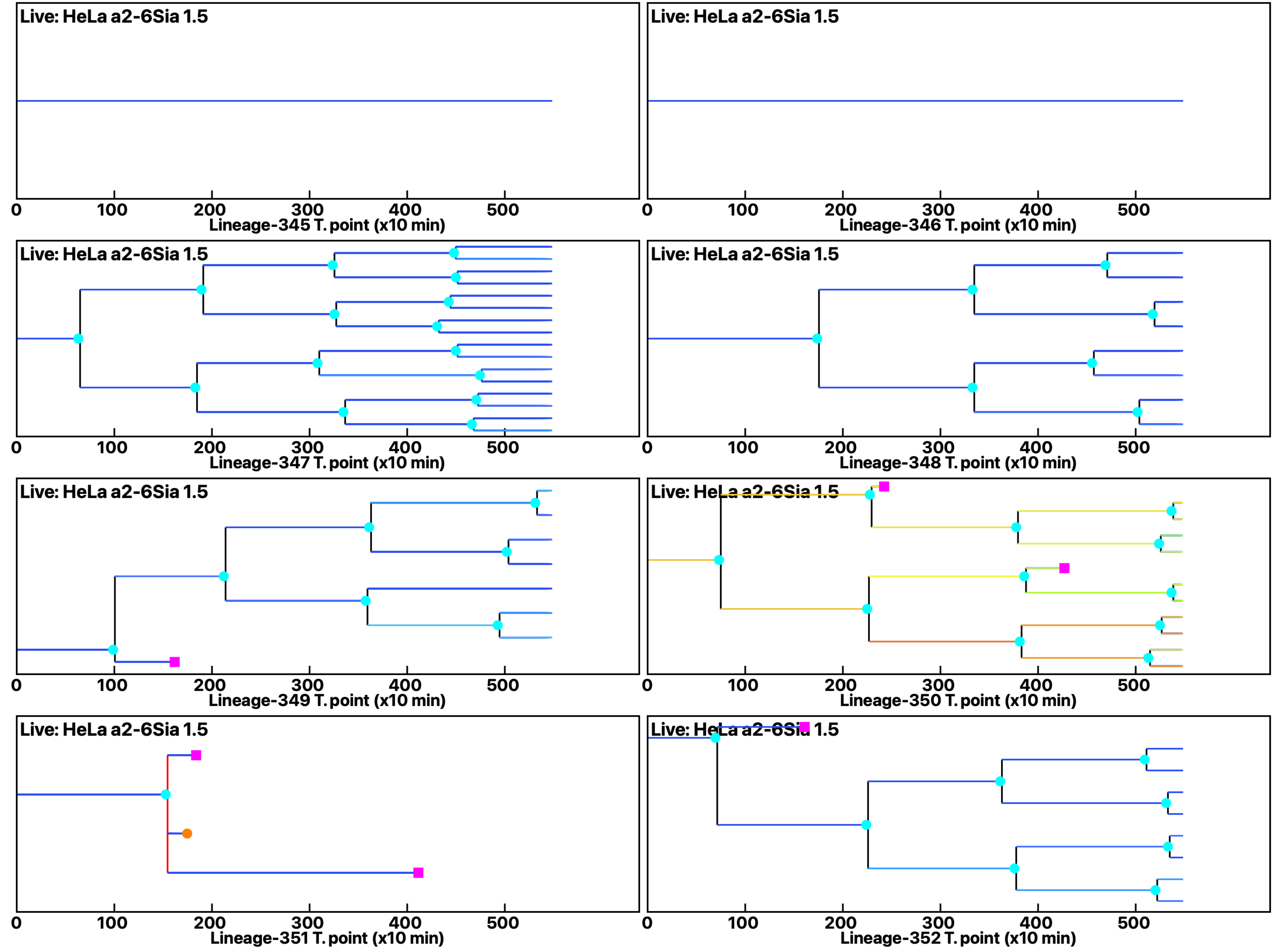

Analysis: Simulation, Treat.: HeLa a2-6Sia 1.5, Cell: HeLa-Simulation

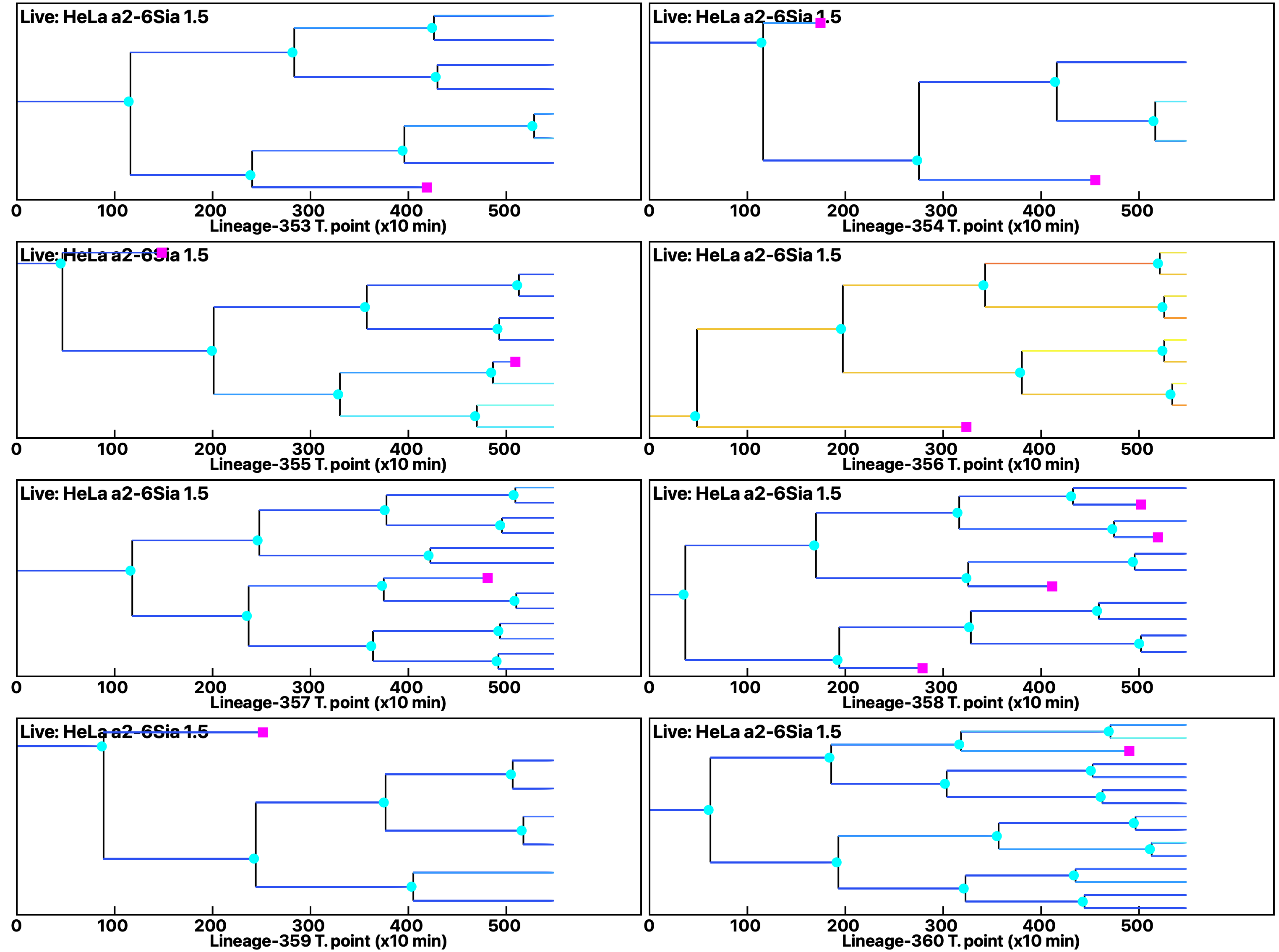

Analysis: Simulation, Treat.: HeLa a2-6Sia 1.5, Cell: HeLa-Simulation

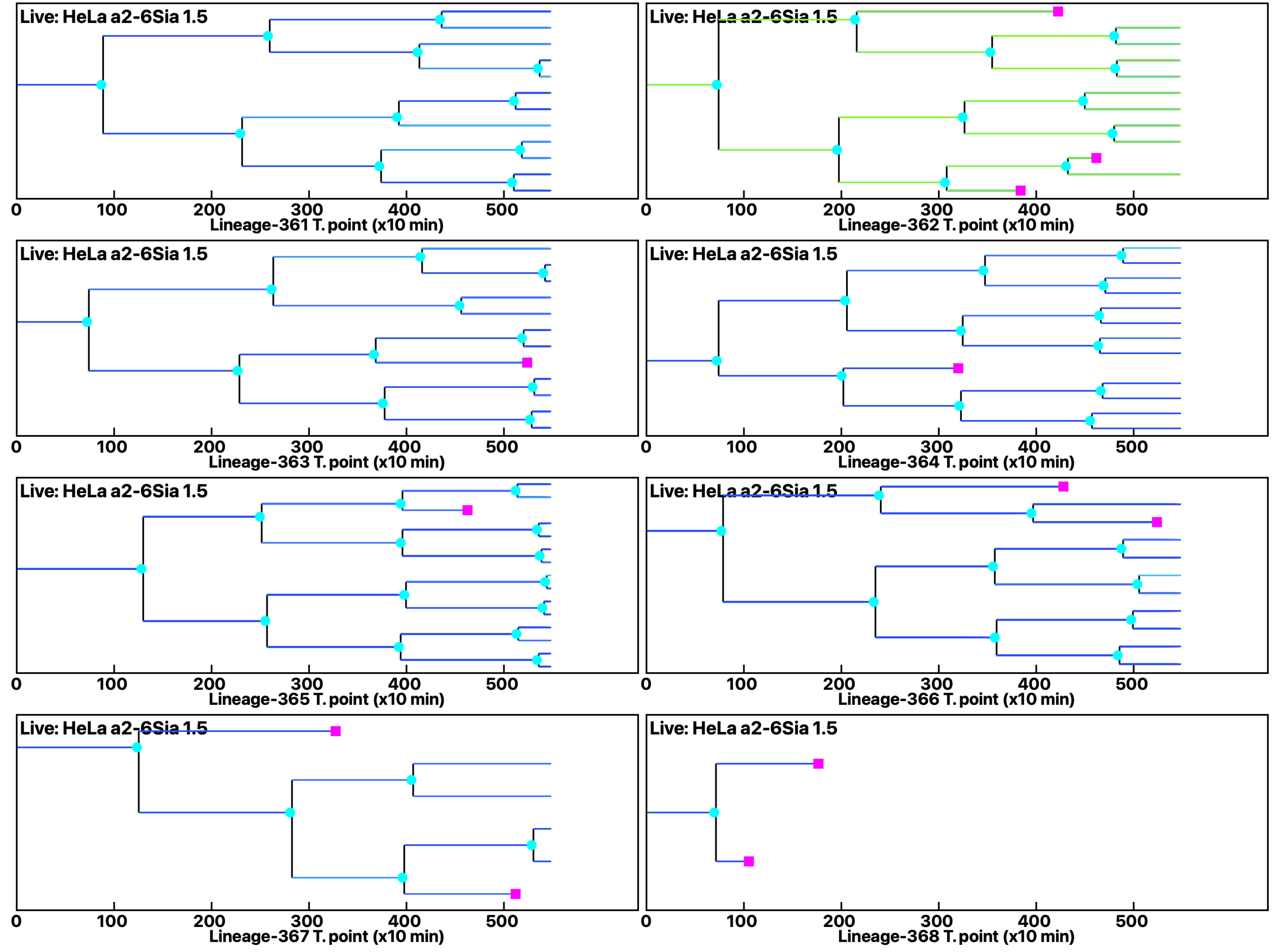

Analysis: Simulation, Treat.: HeLa a2-6Sia 1.5, Cell: HeLa-Simulation

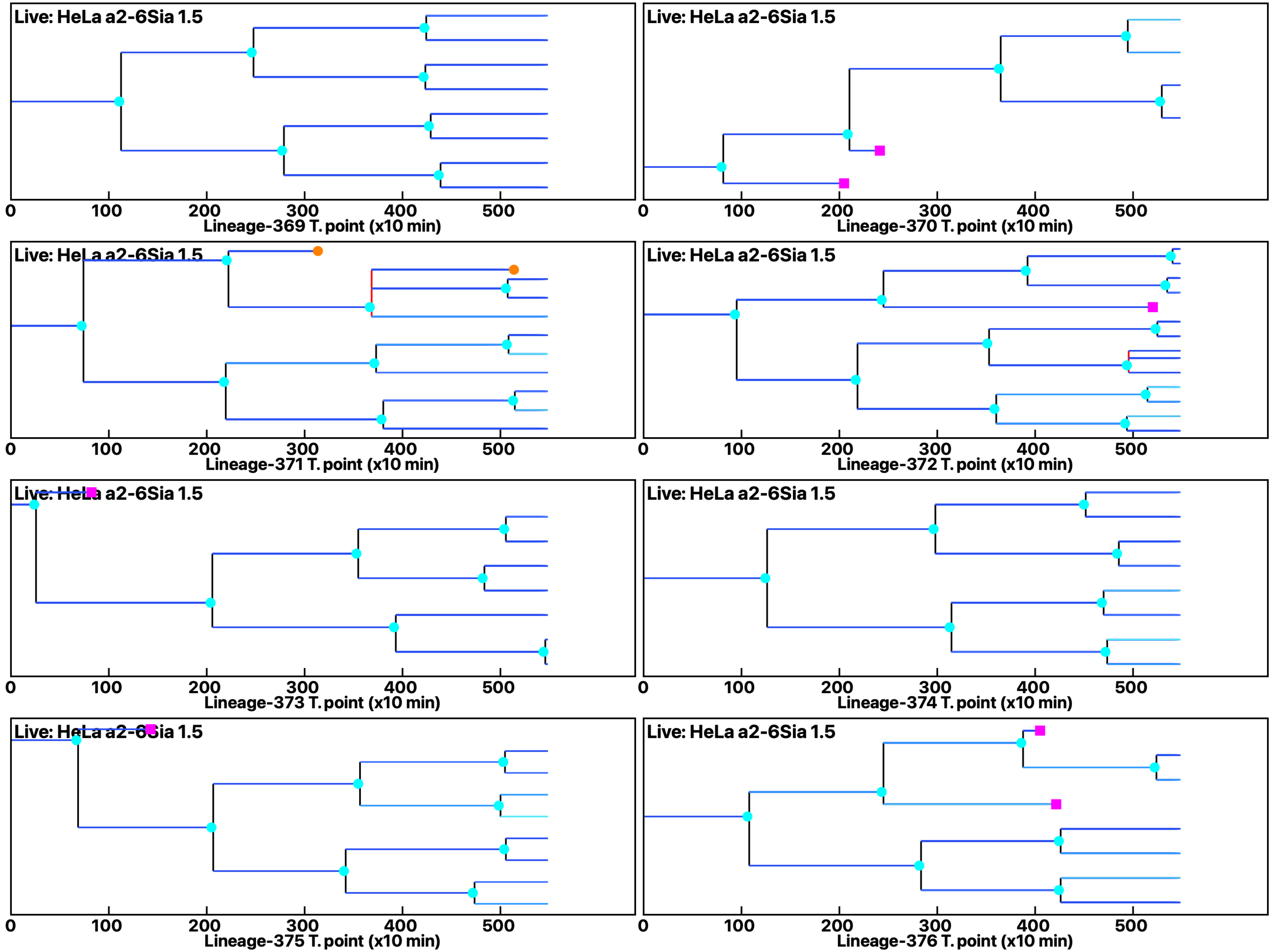

Analysis: Simulation, Treat.: HeLa a2-6Sia 1.5, Cell: HeLa-Simulation

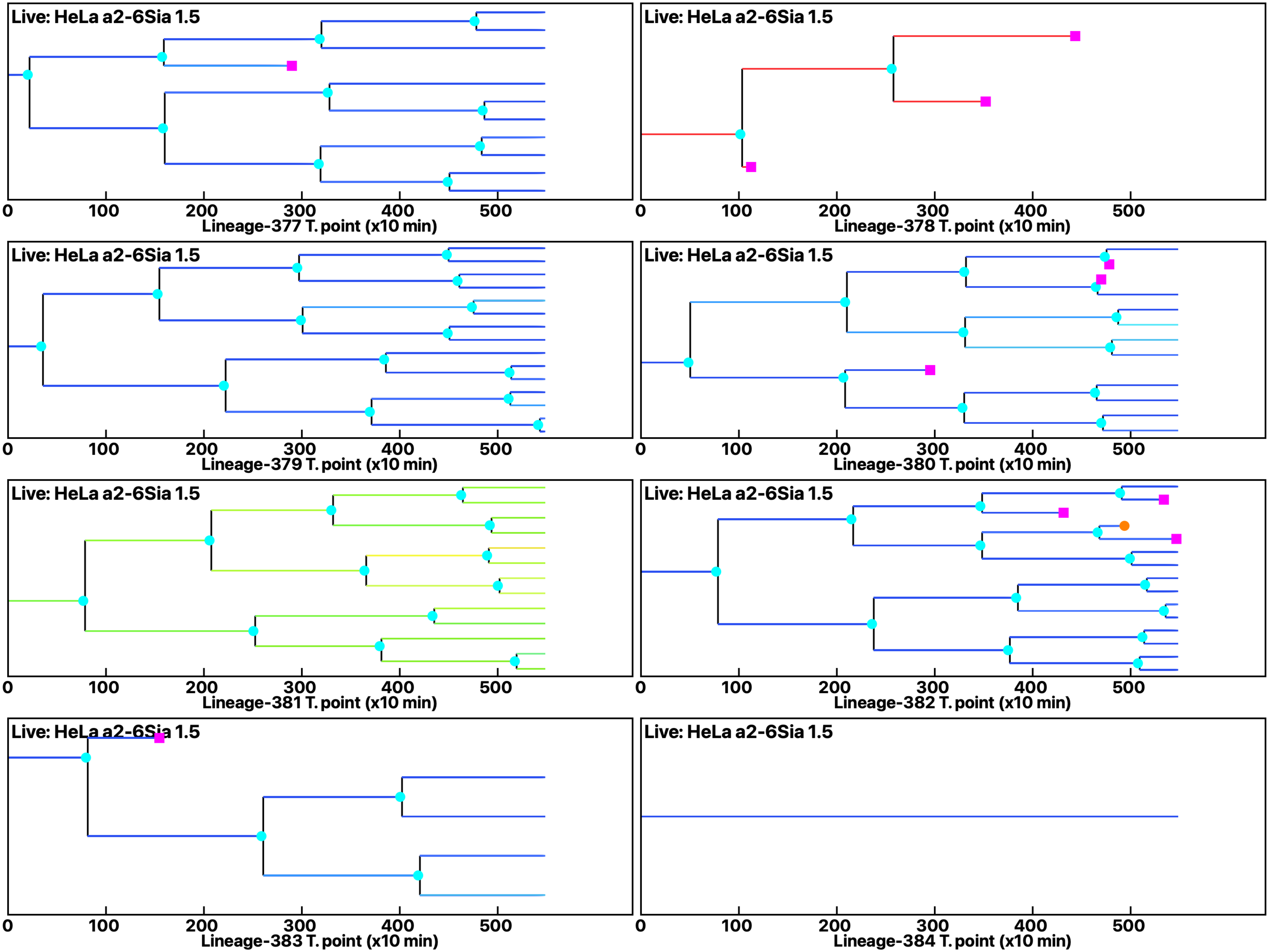

Analysis: Simulation, Treat.: HeLa a2-6Sia 1.5, Cell: HeLa-Simulation

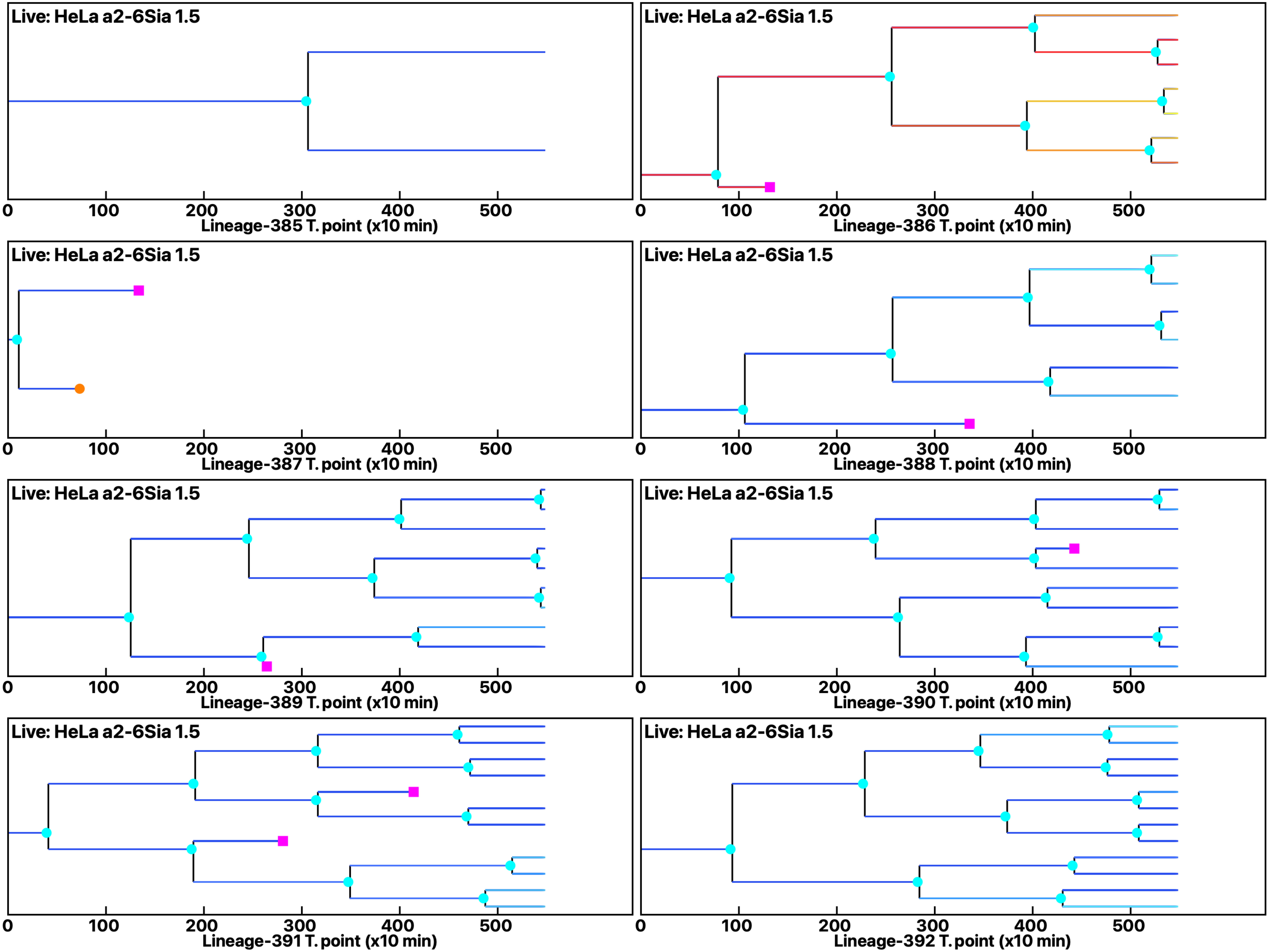

Analysis: Simulation, Treat.: HeLa a2-6Sia 1.5, Cell: HeLa-Simulation

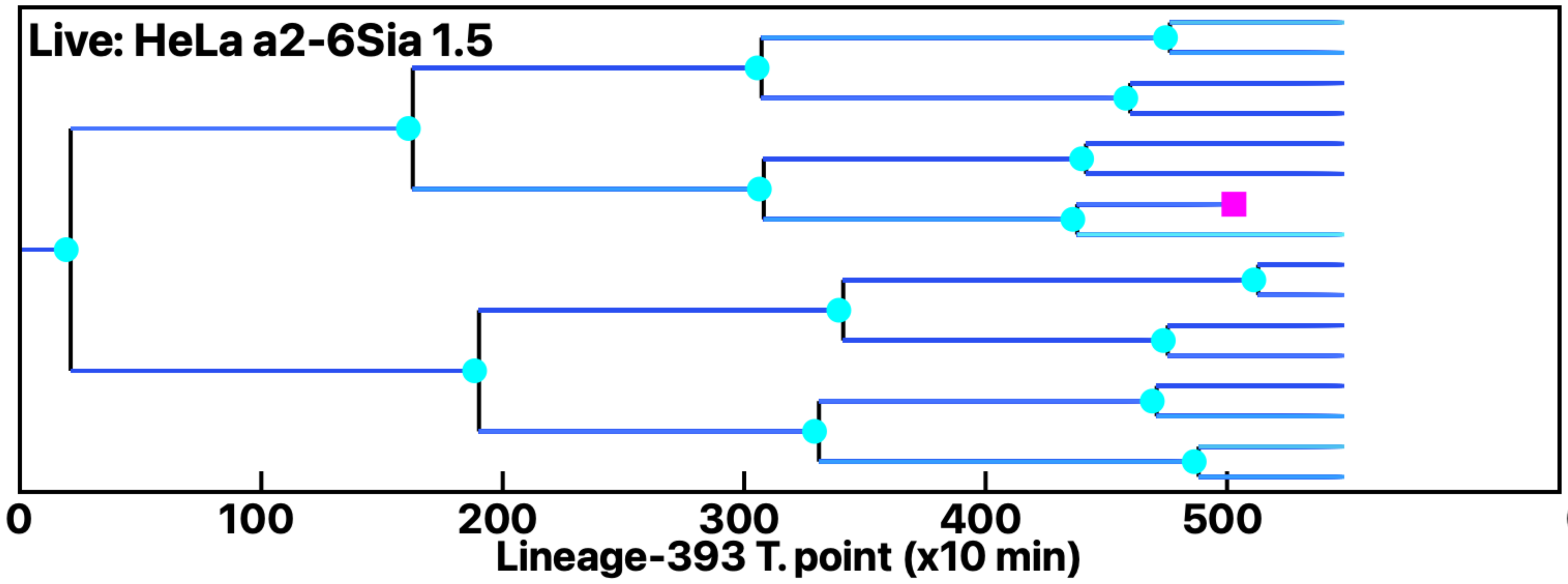

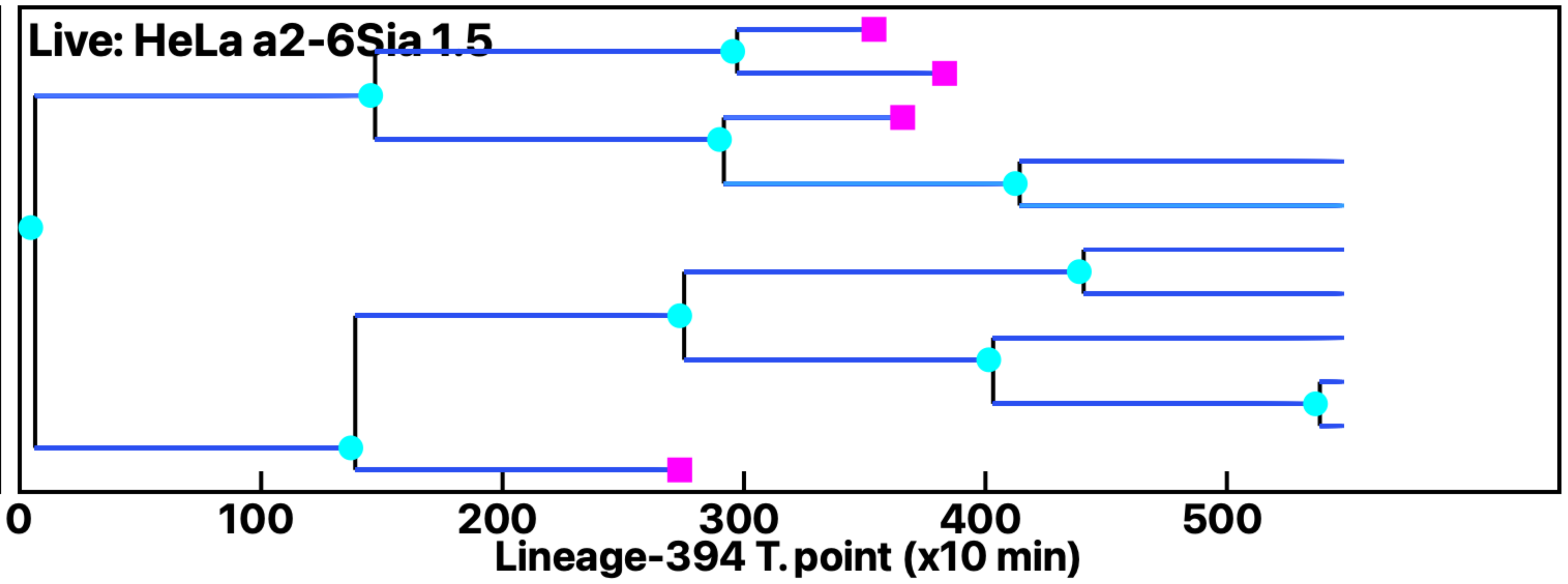

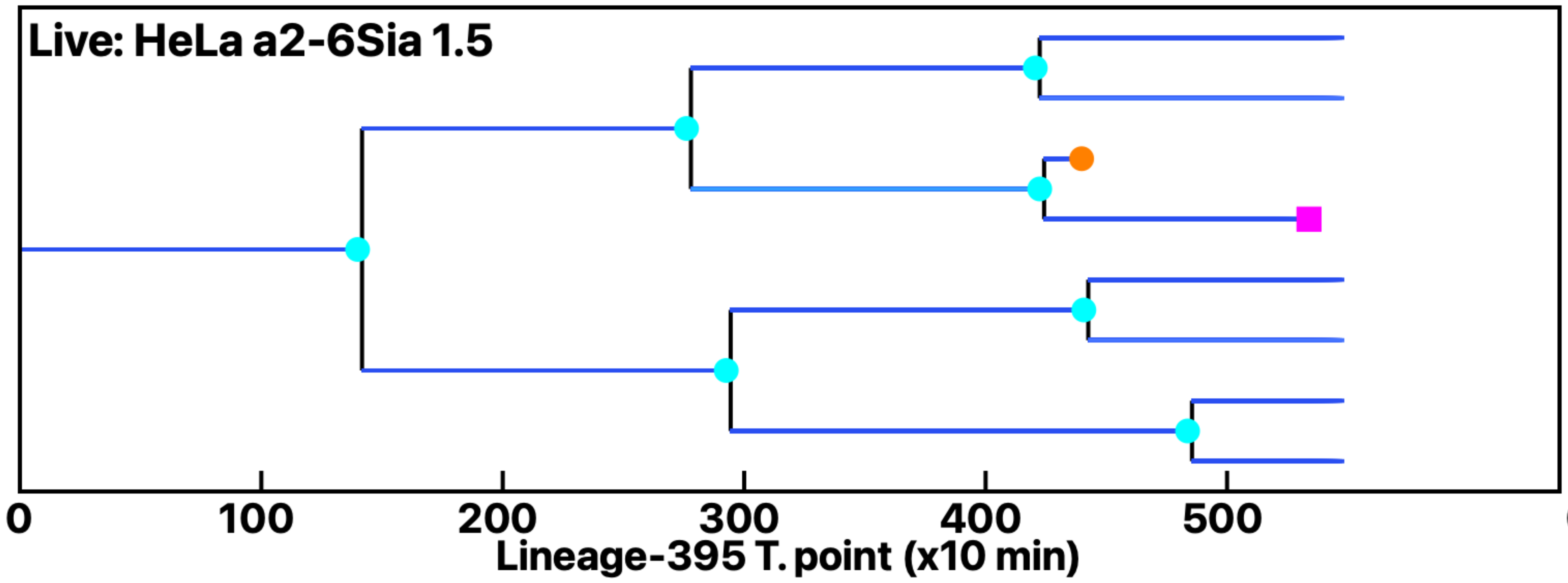

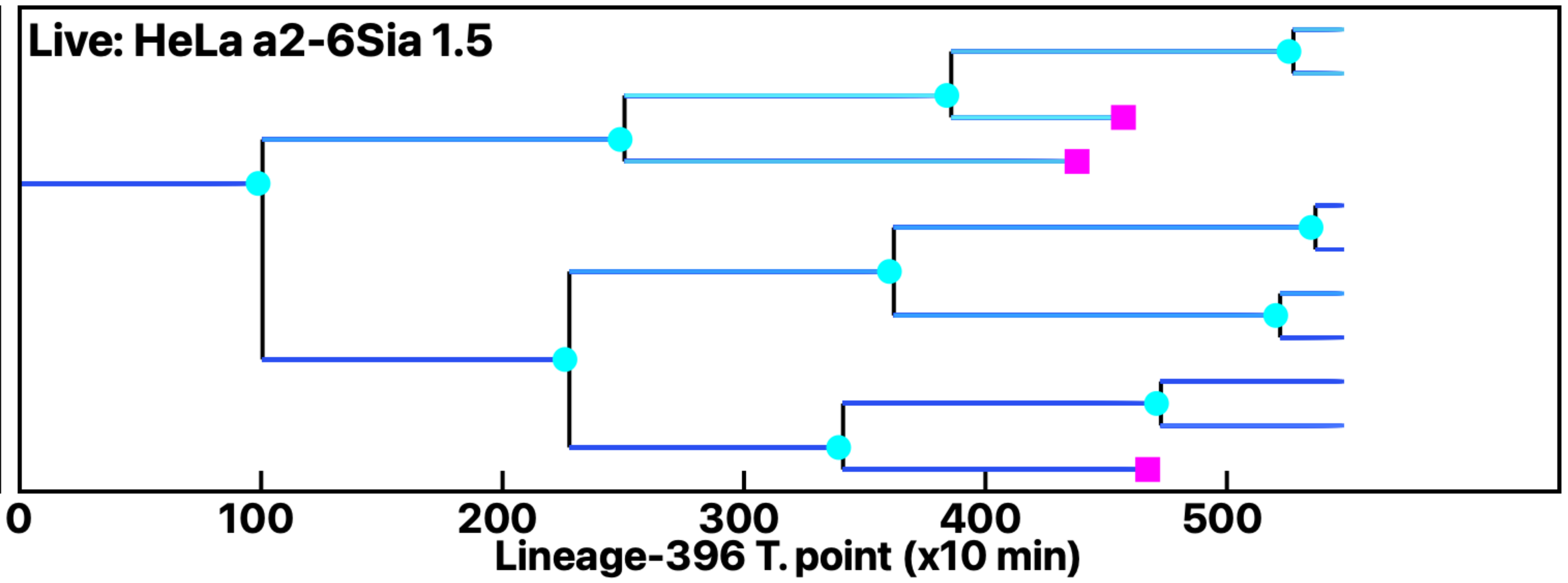

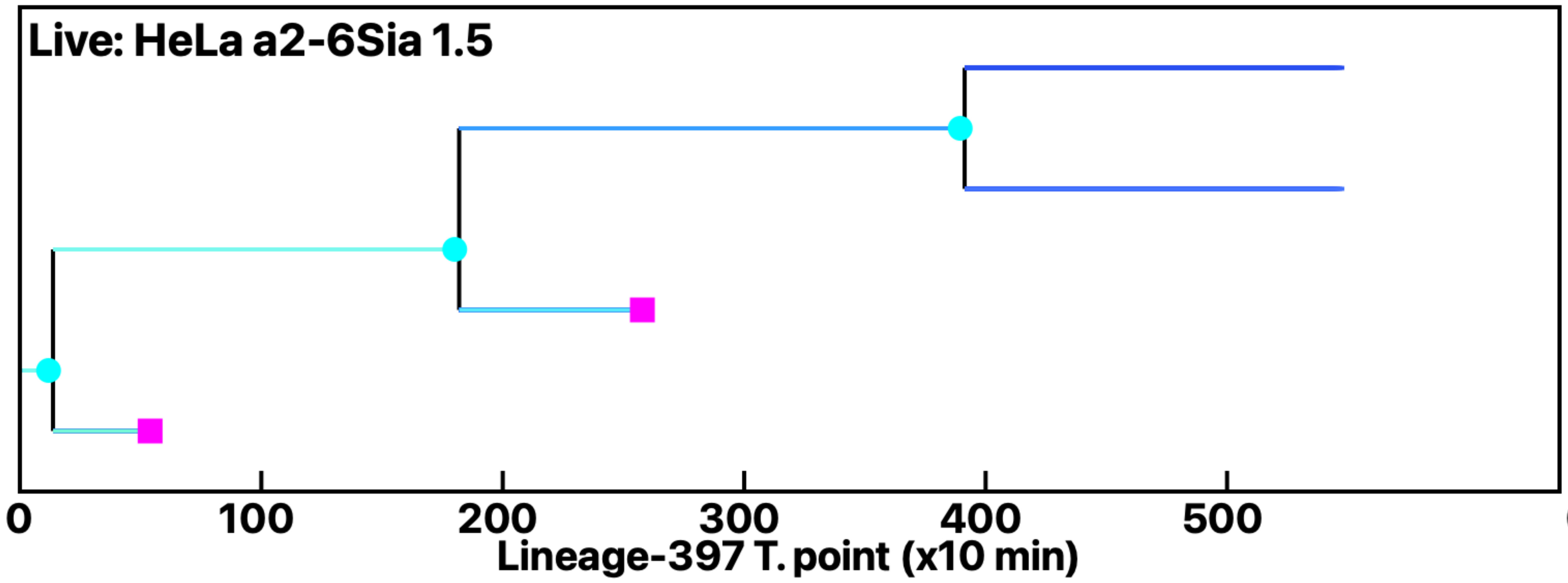

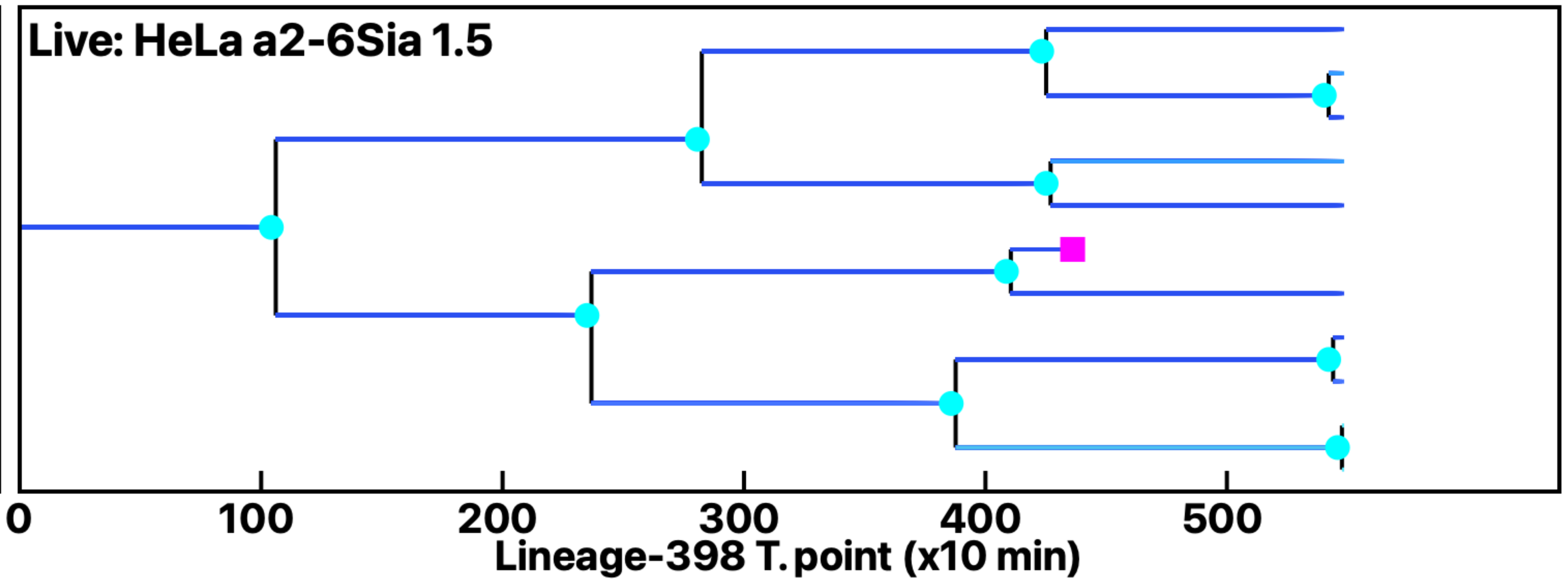

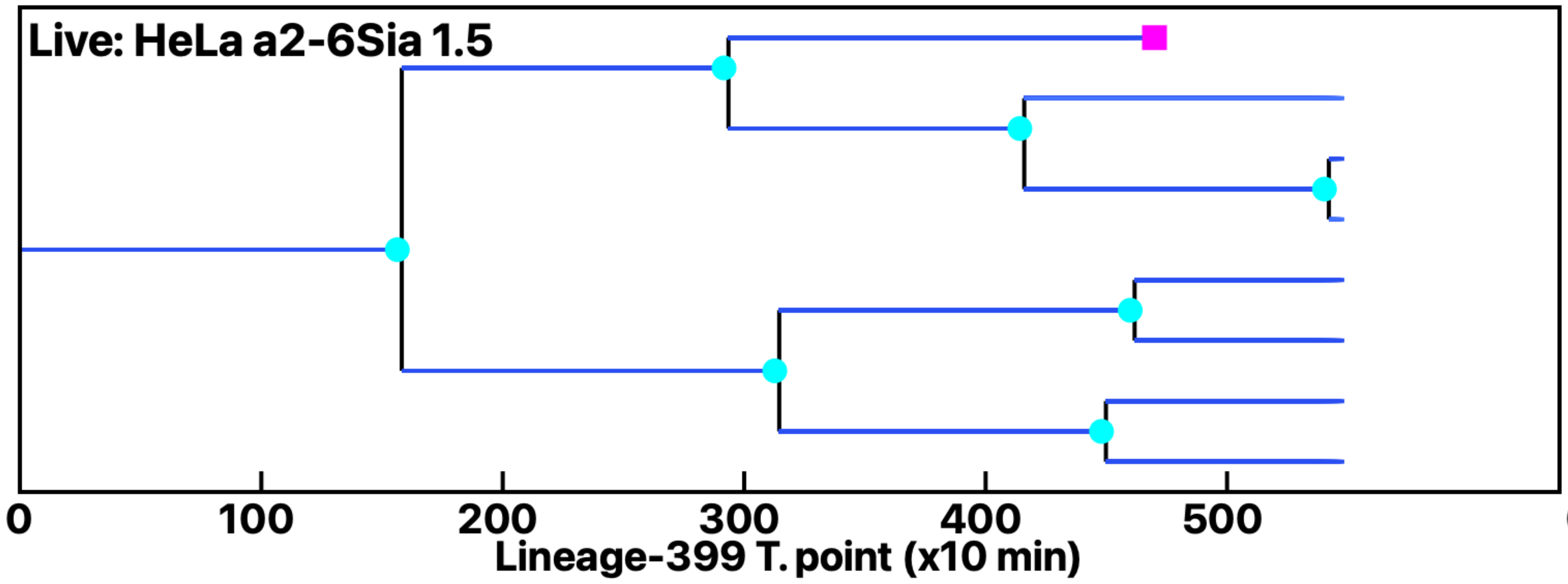

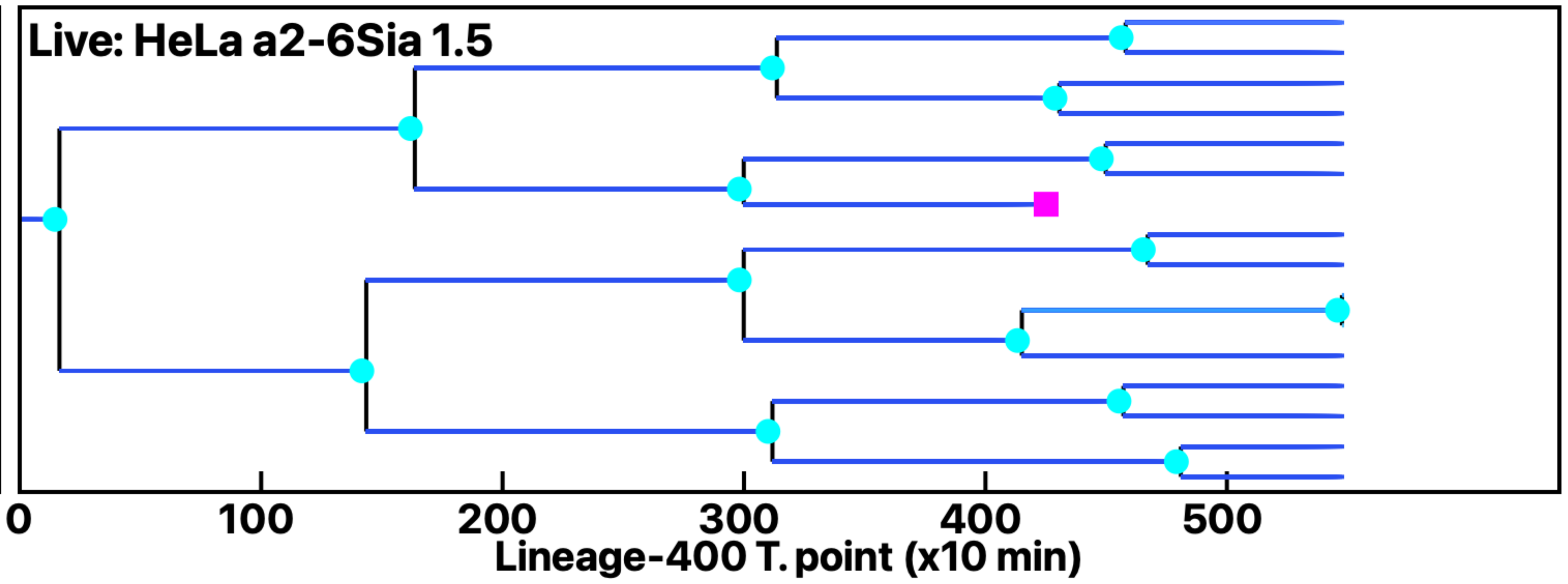

Analysis: Simulation, Treat.: HeLa a2-6Sia 1.5, Cell: HeLa-Simulation

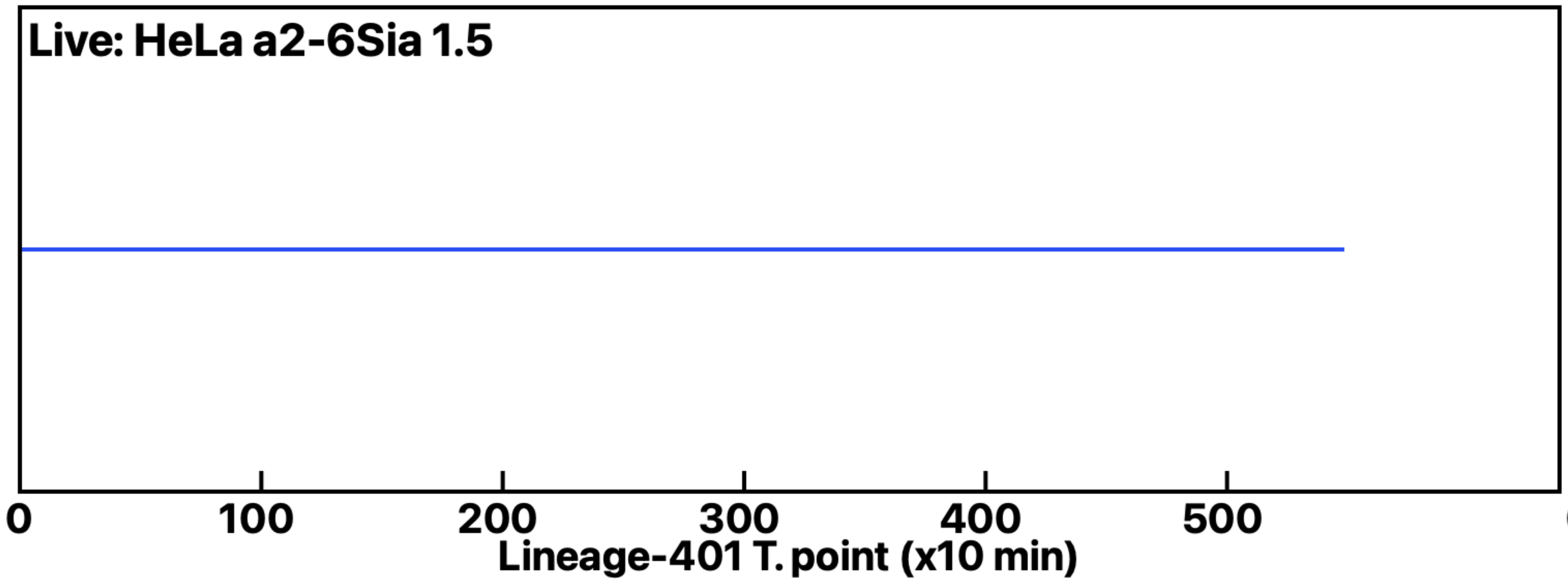

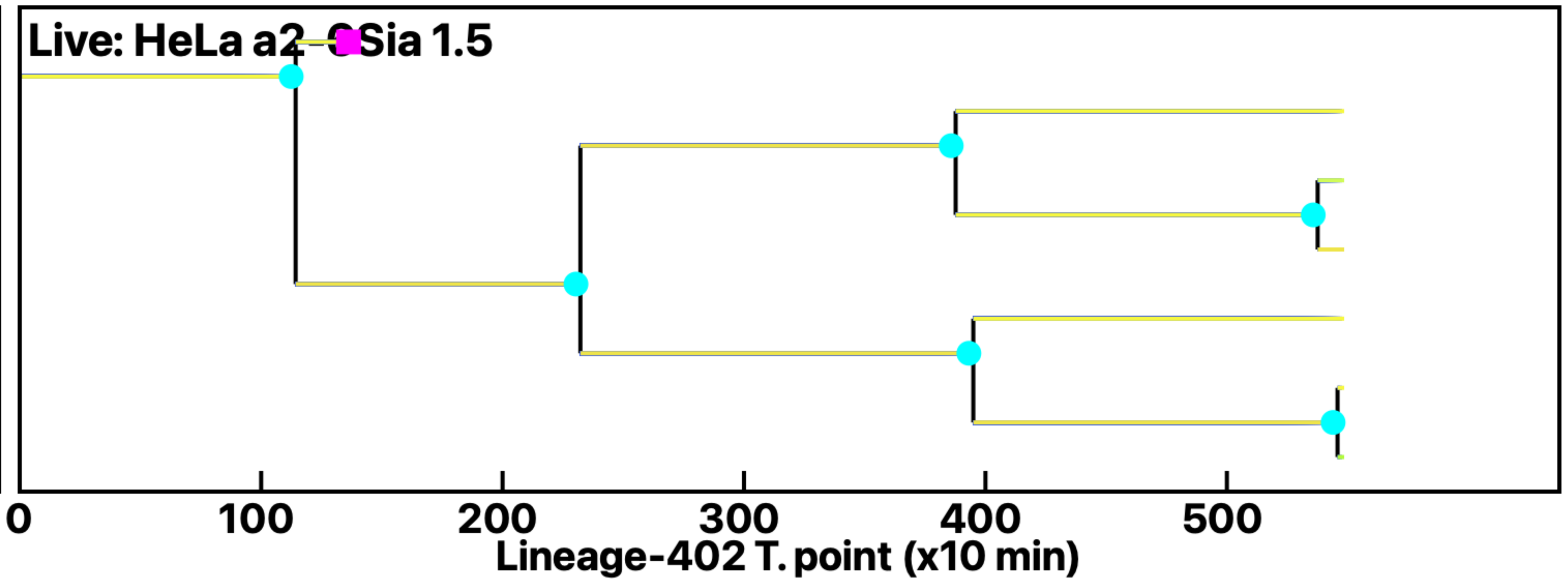

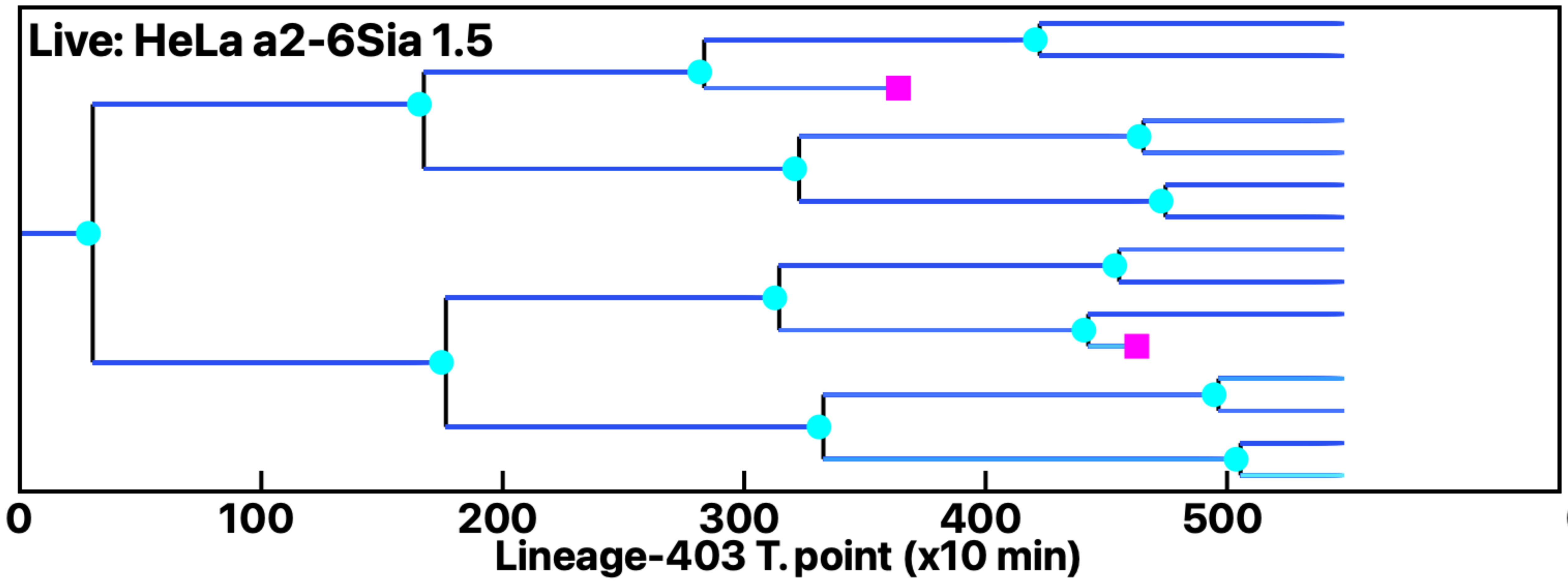

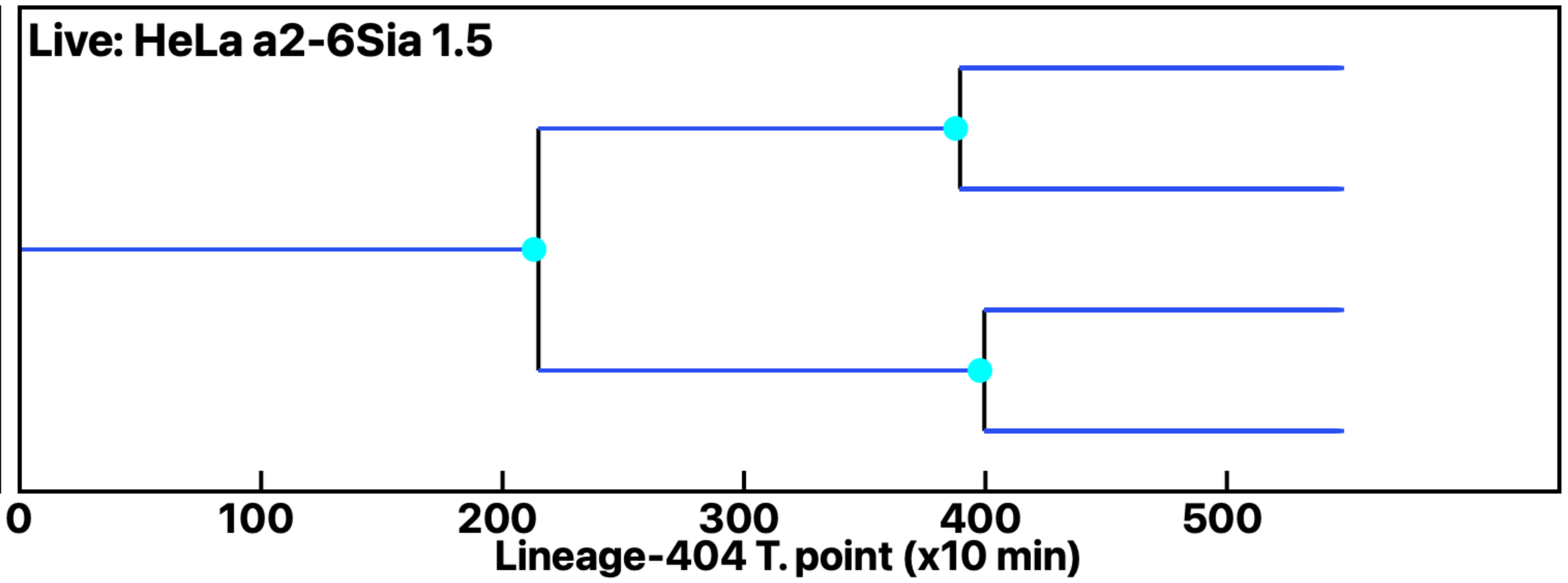

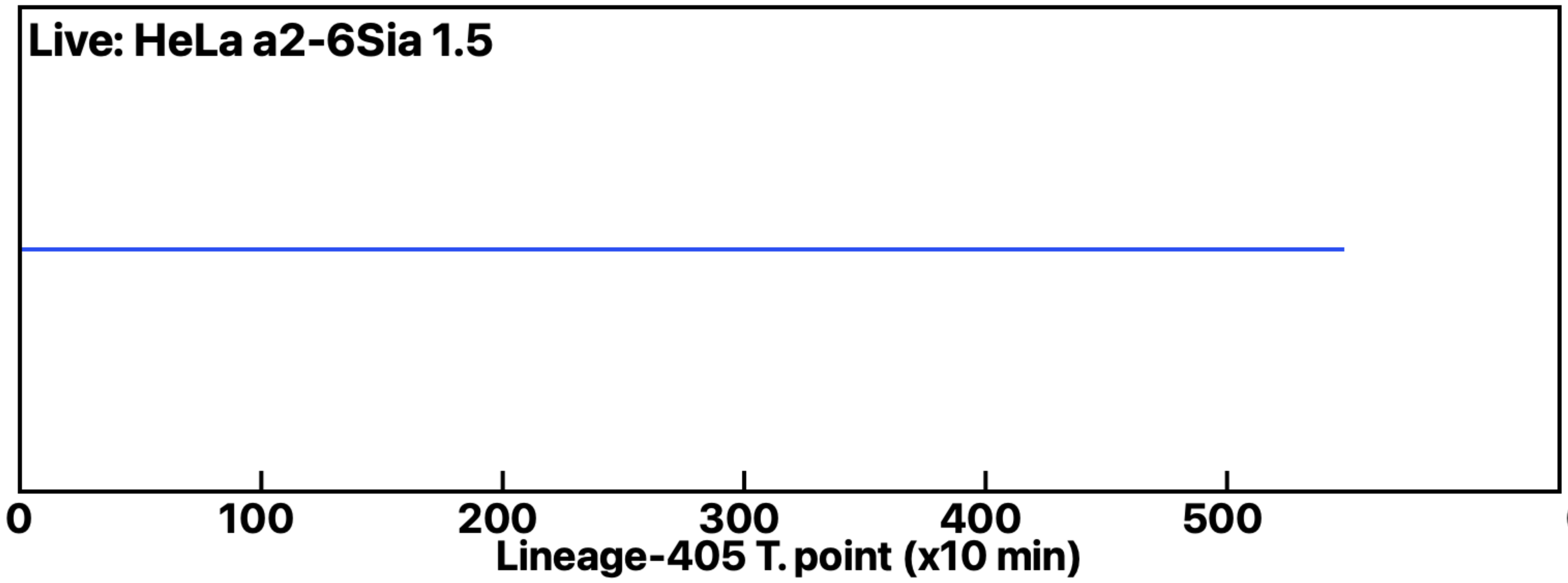

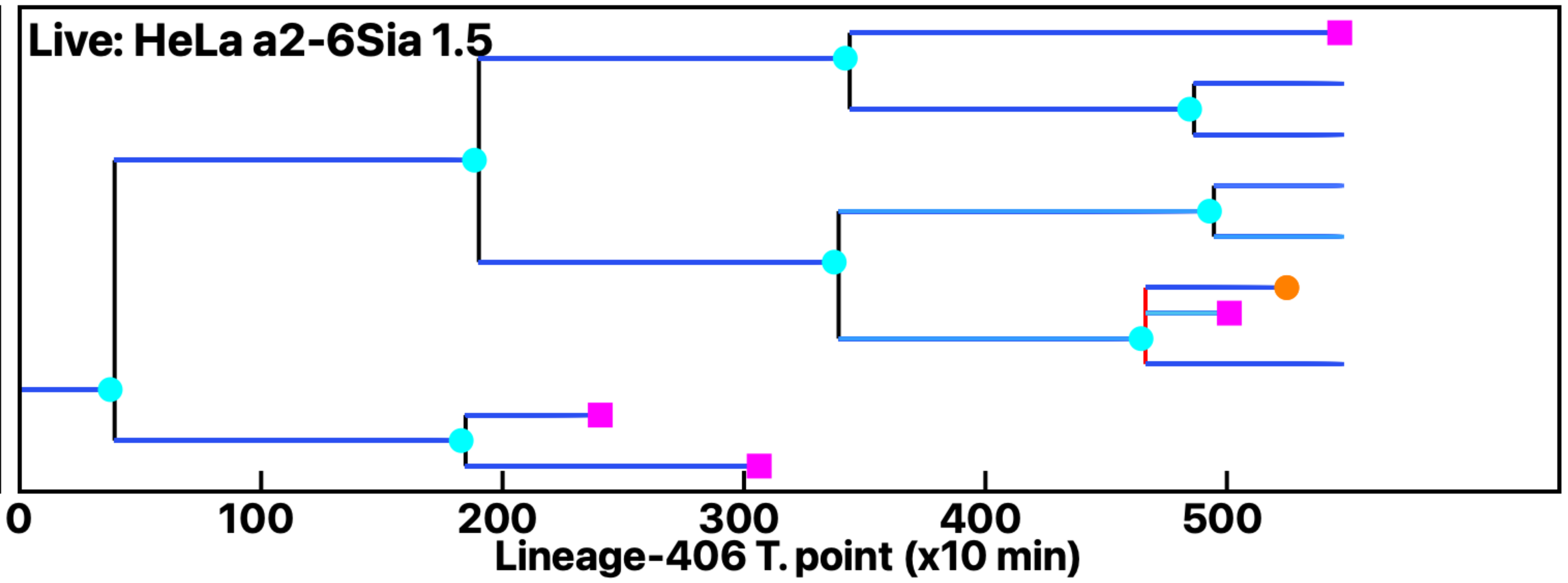

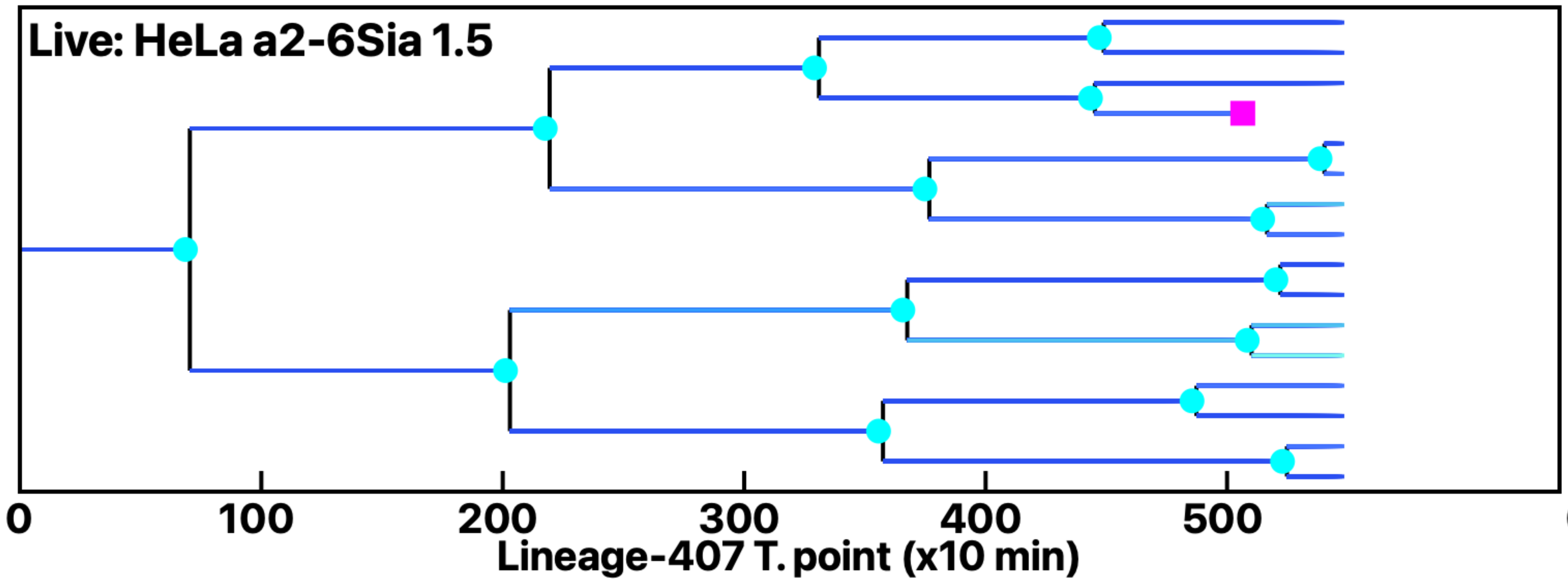

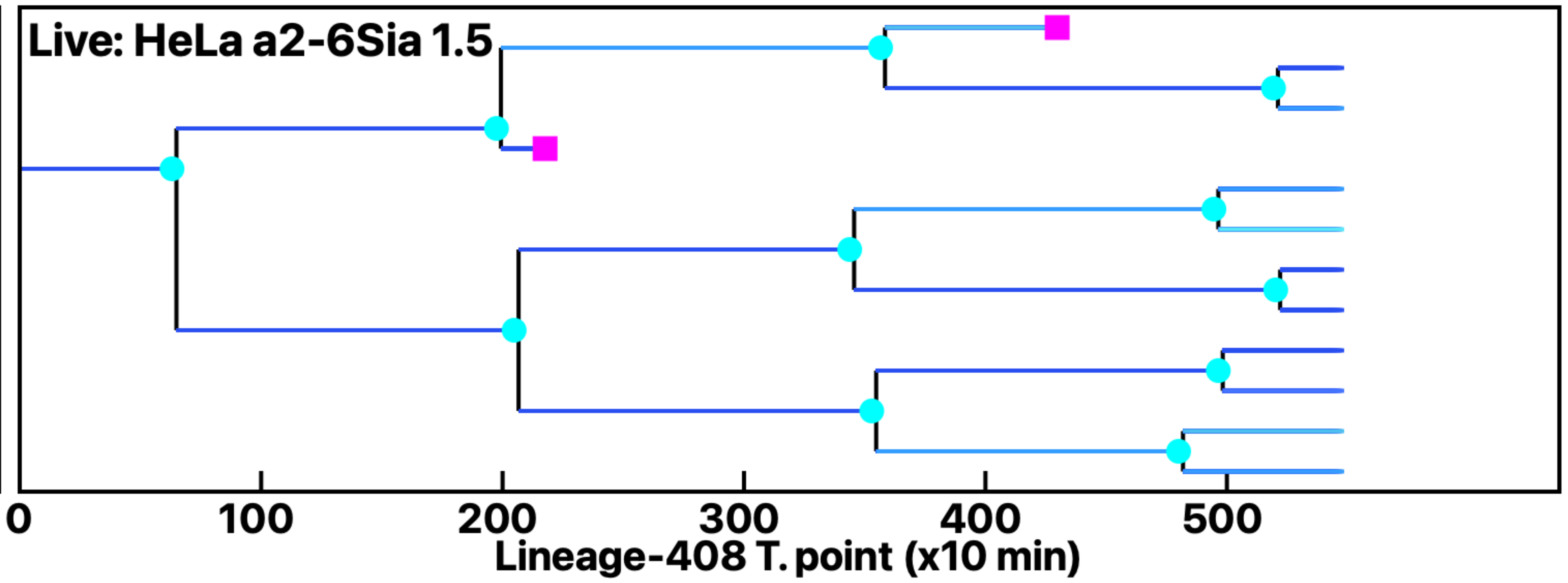

Analysis: Simulation, Treat.: HeLa a2-6Sia 1.5, Cell: HeLa-Simulation

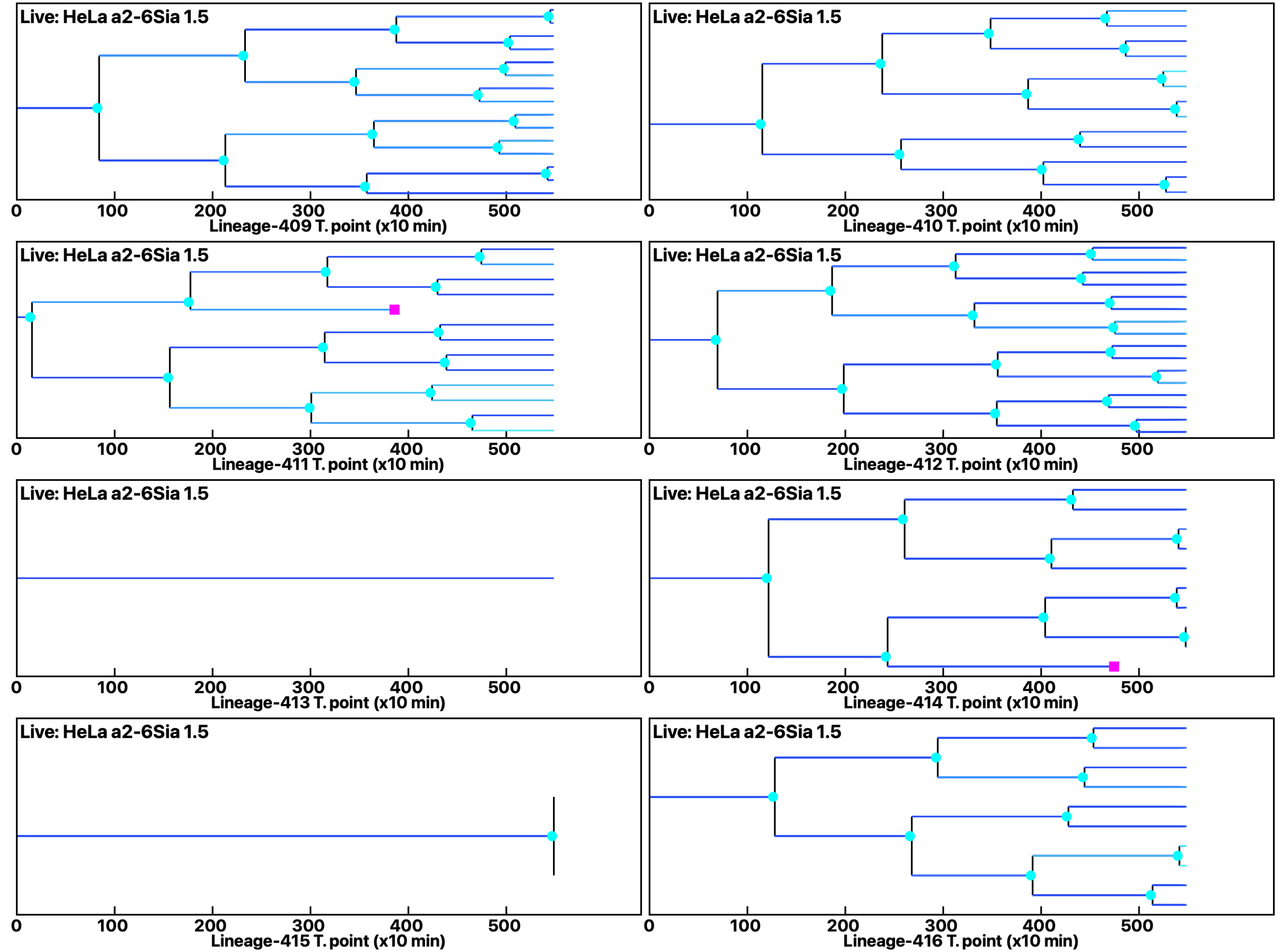

Analysis: Simulation, Treat.: HeLa a2-6Sia 1.5, Cell: HeLa-Simulation

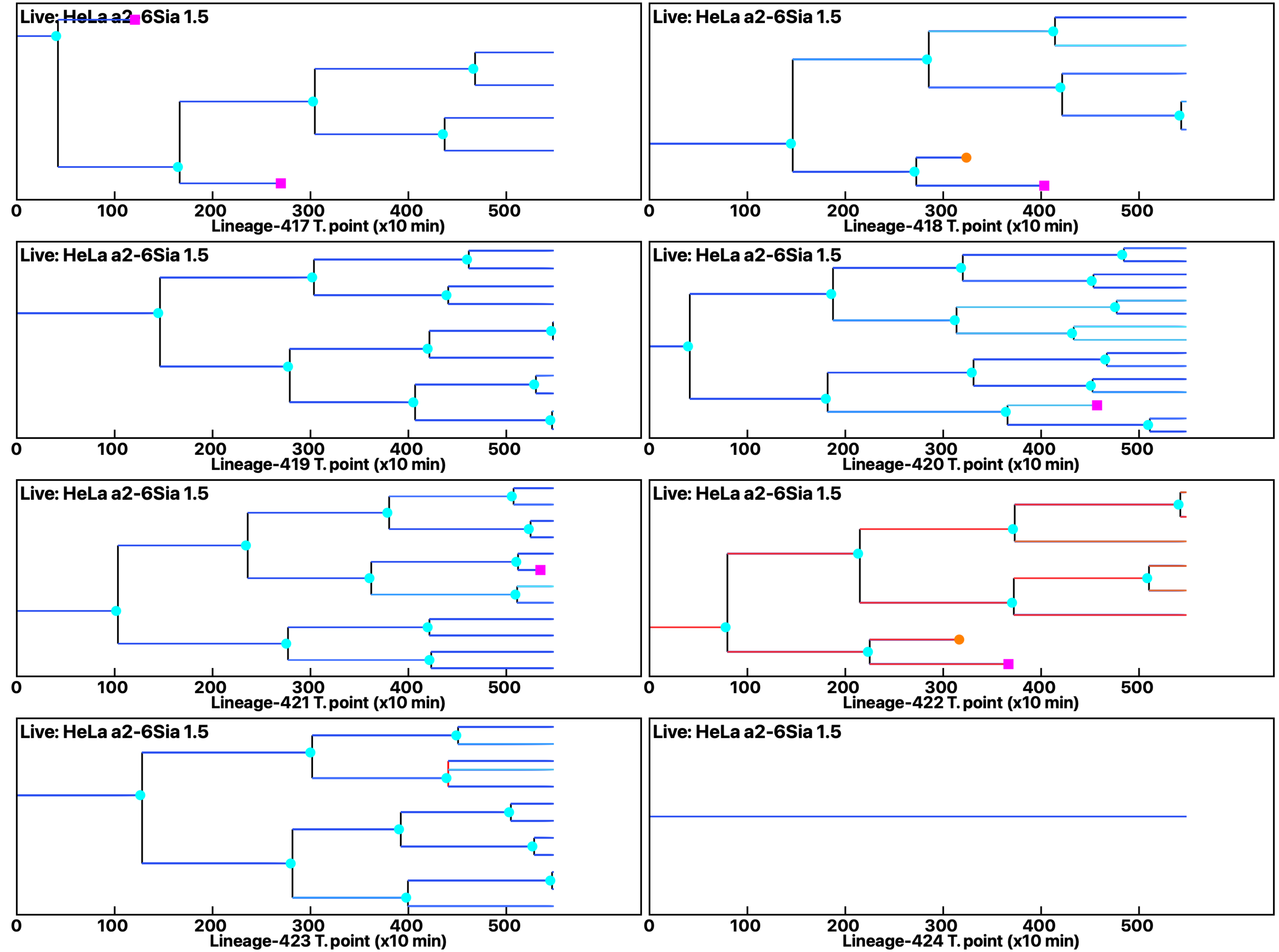

Analysis: Simulation, Treat.: HeLa a2-6Sia 1.5, Cell: HeLa-Simulation

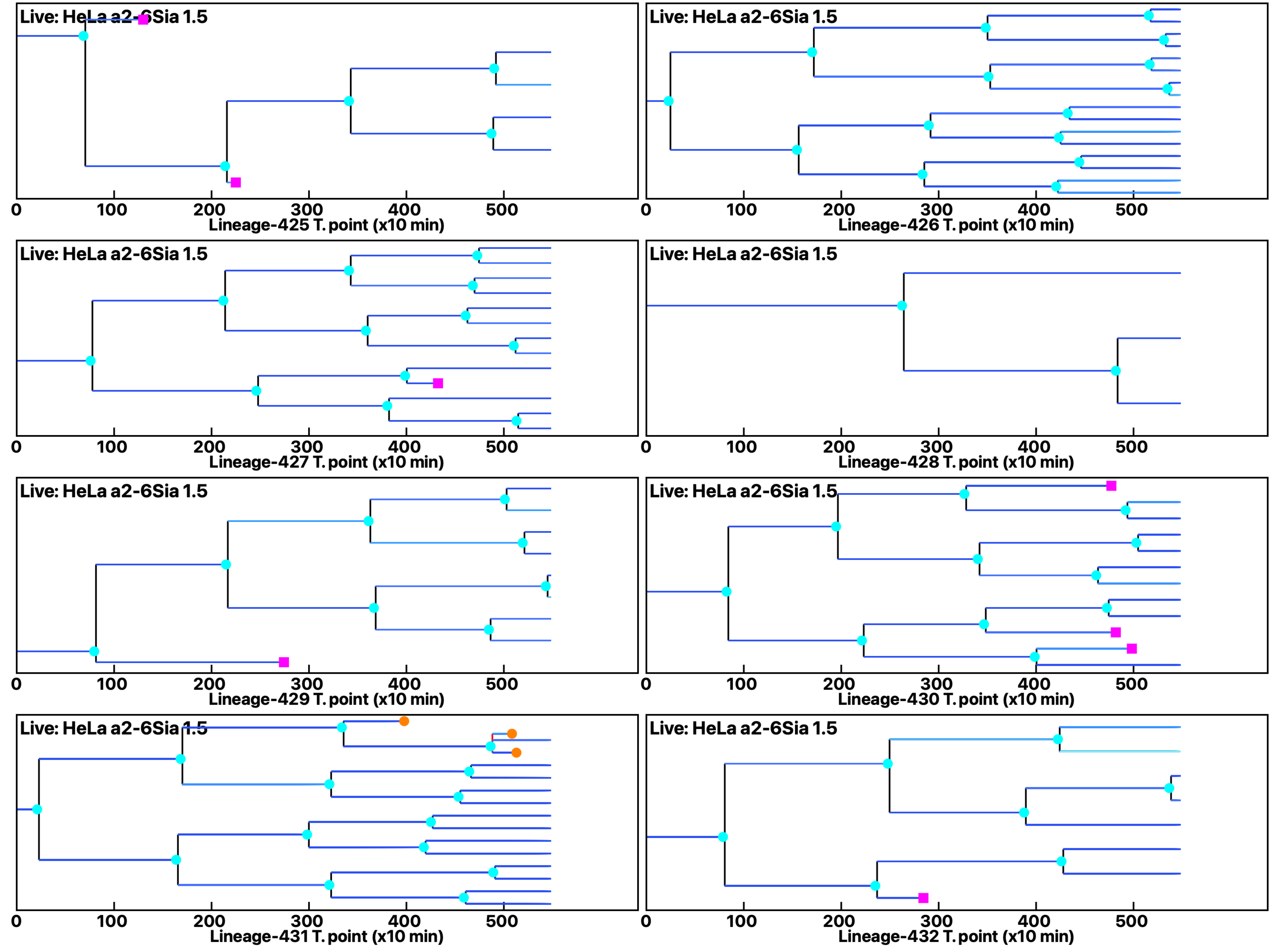

Analysis: Simulation, Treat.: HeLa a2-6Sia 1.5, Cell: HeLa-Simulation

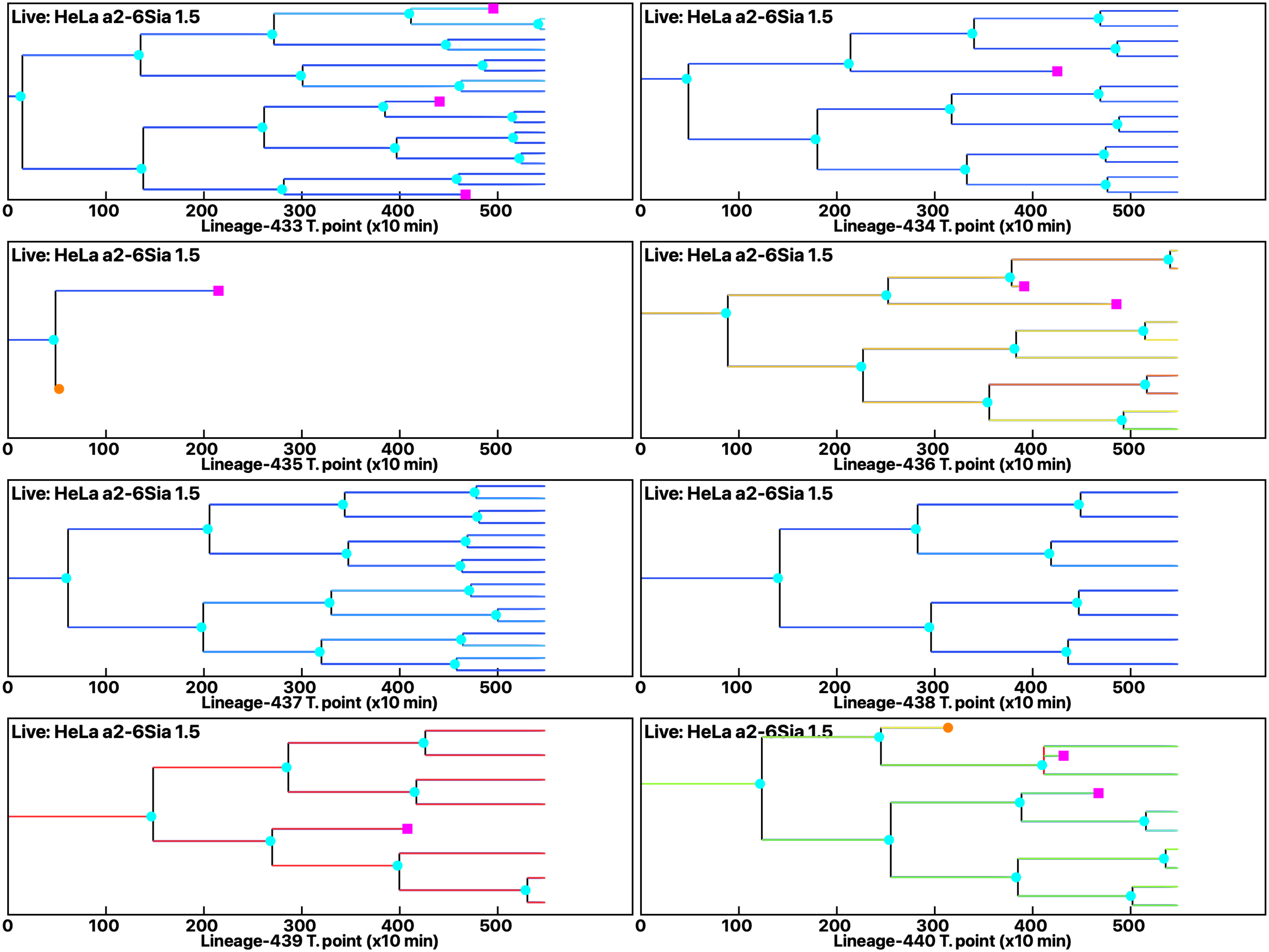

Analysis: Simulation, Treat.: HeLa a2-6Sia 1.5, Cell: HeLa-Simulation

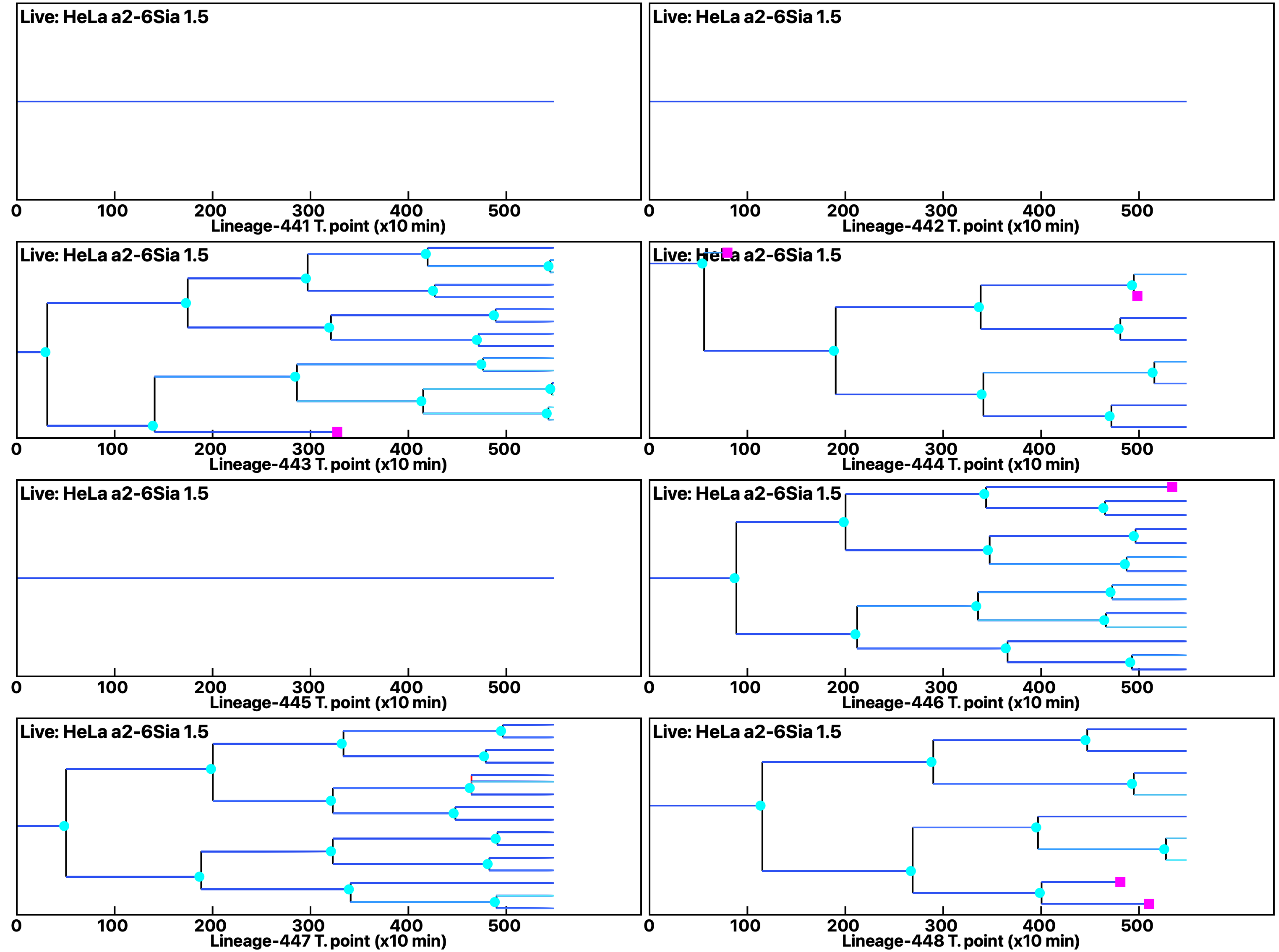

Analysis: Simulation, Treat.: HeLa a2-6Sia 1.5, Cell: HeLa-Simulation

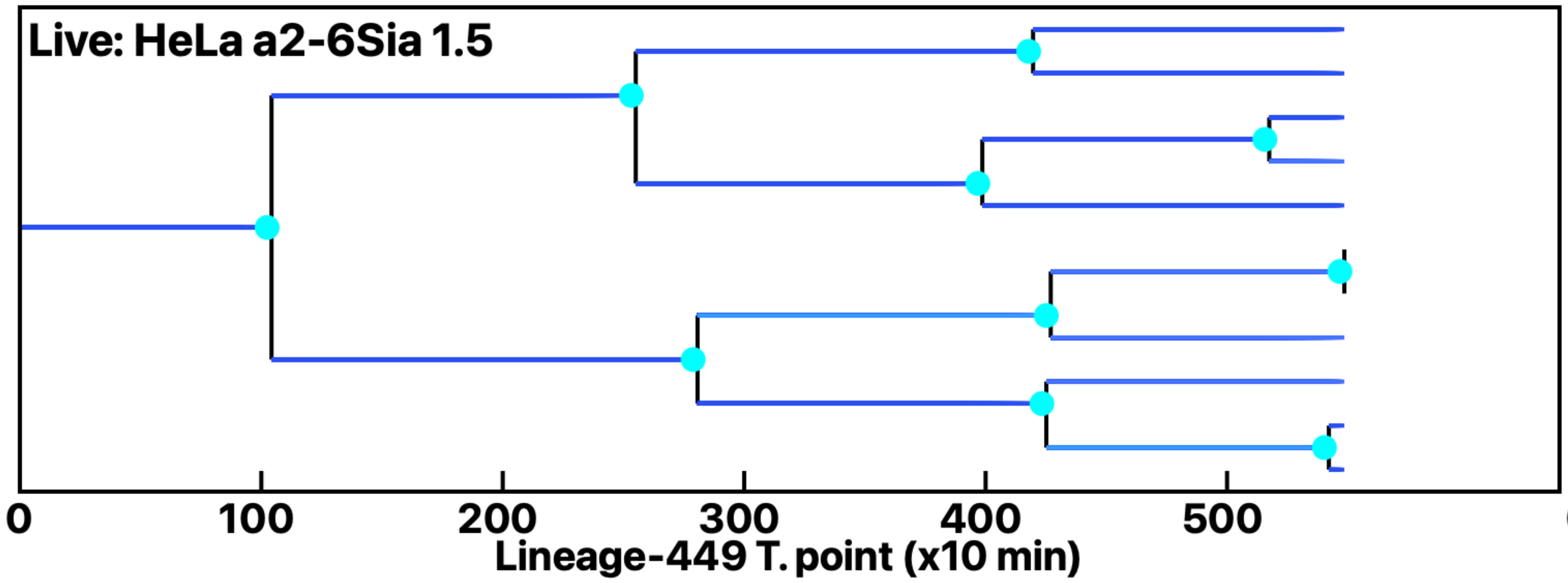

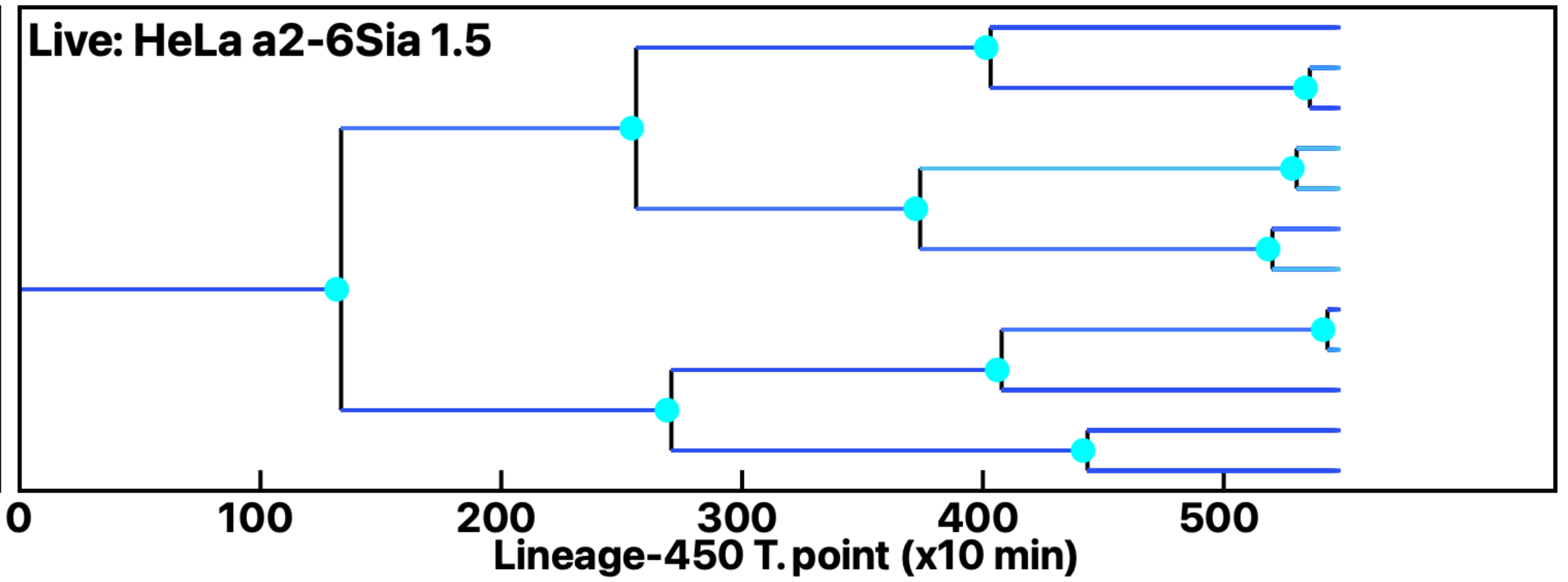

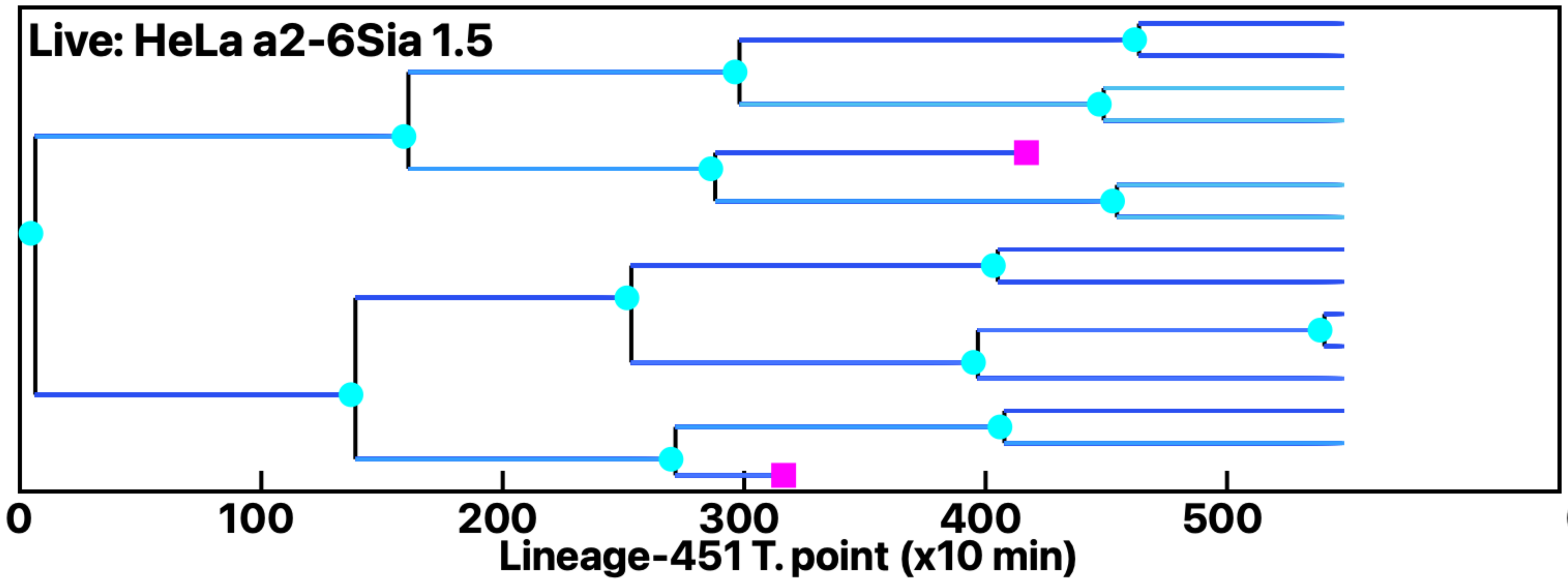

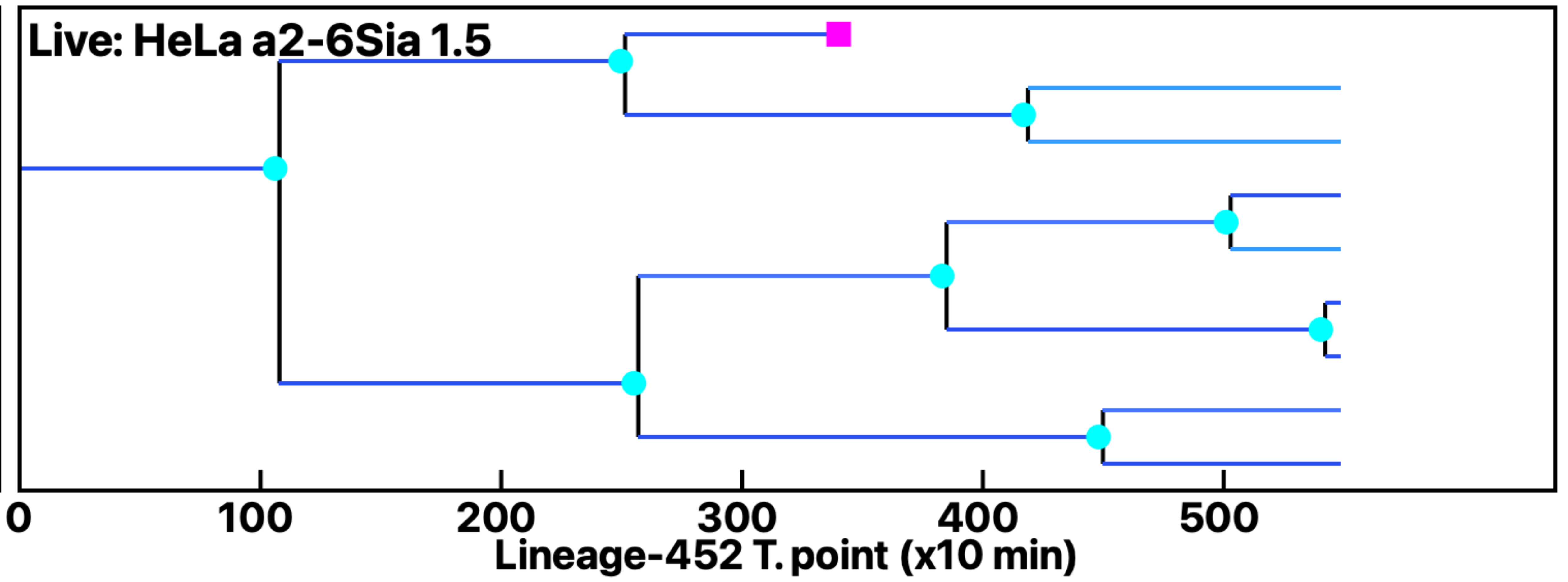

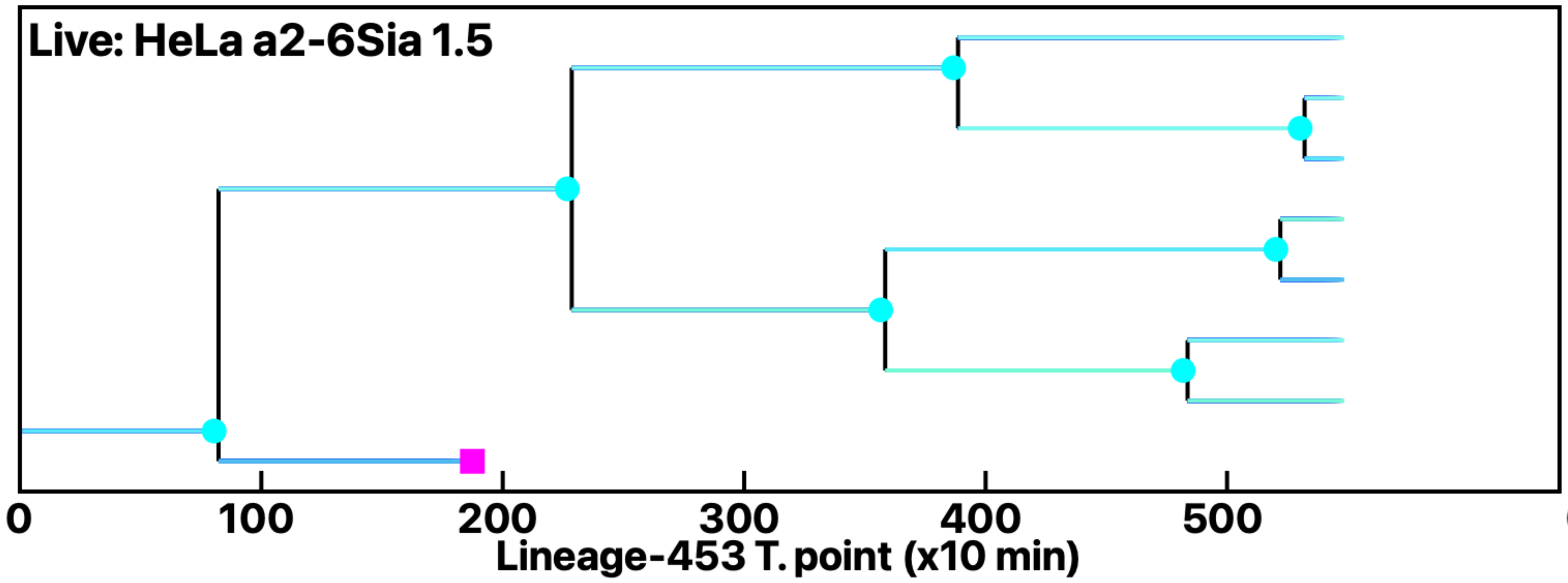

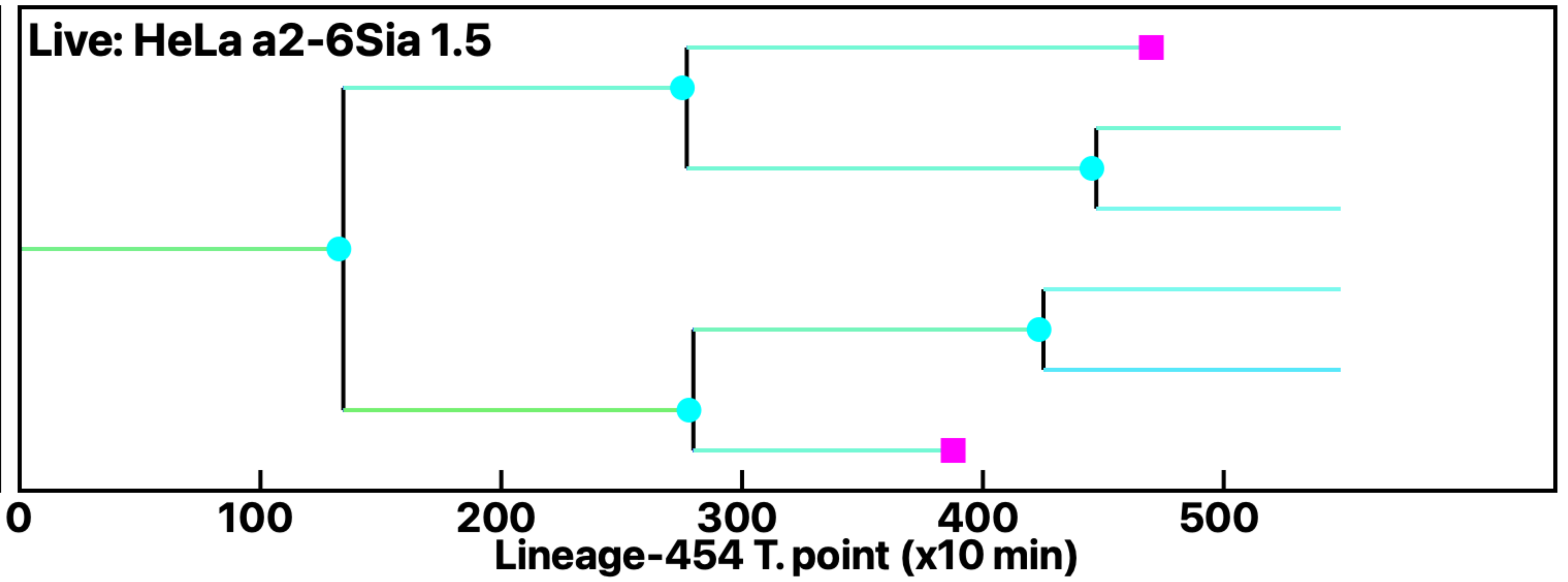

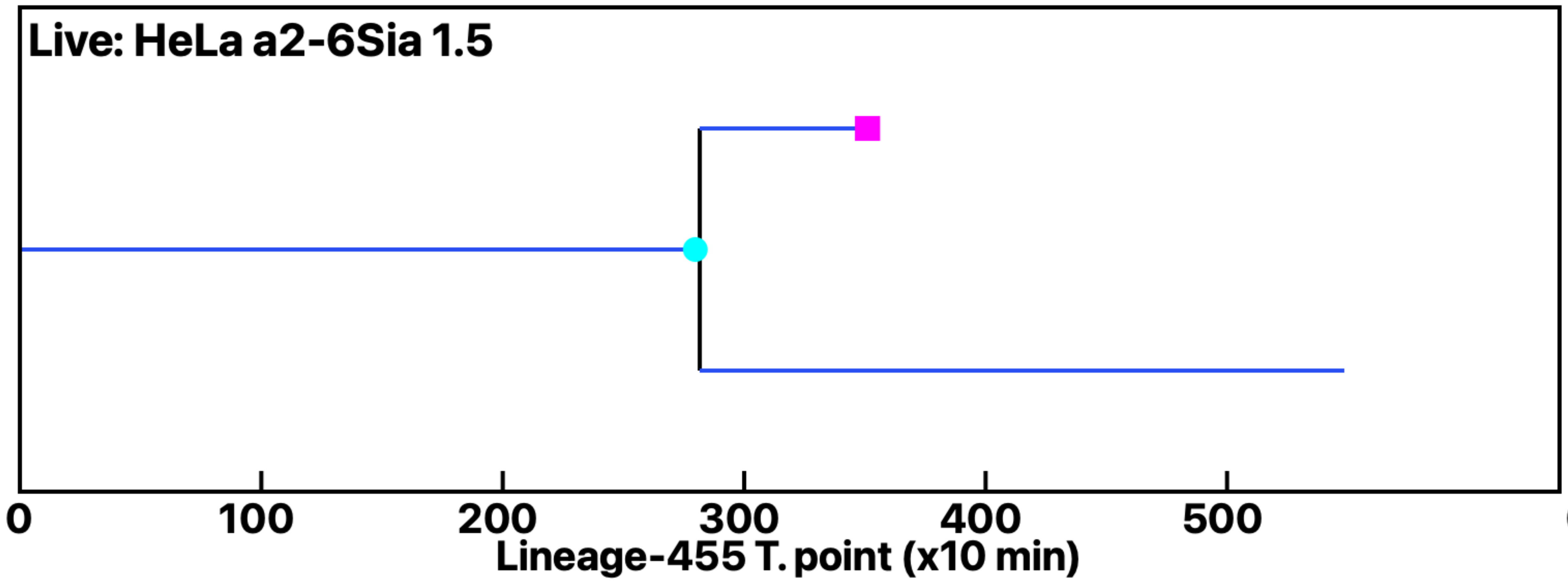

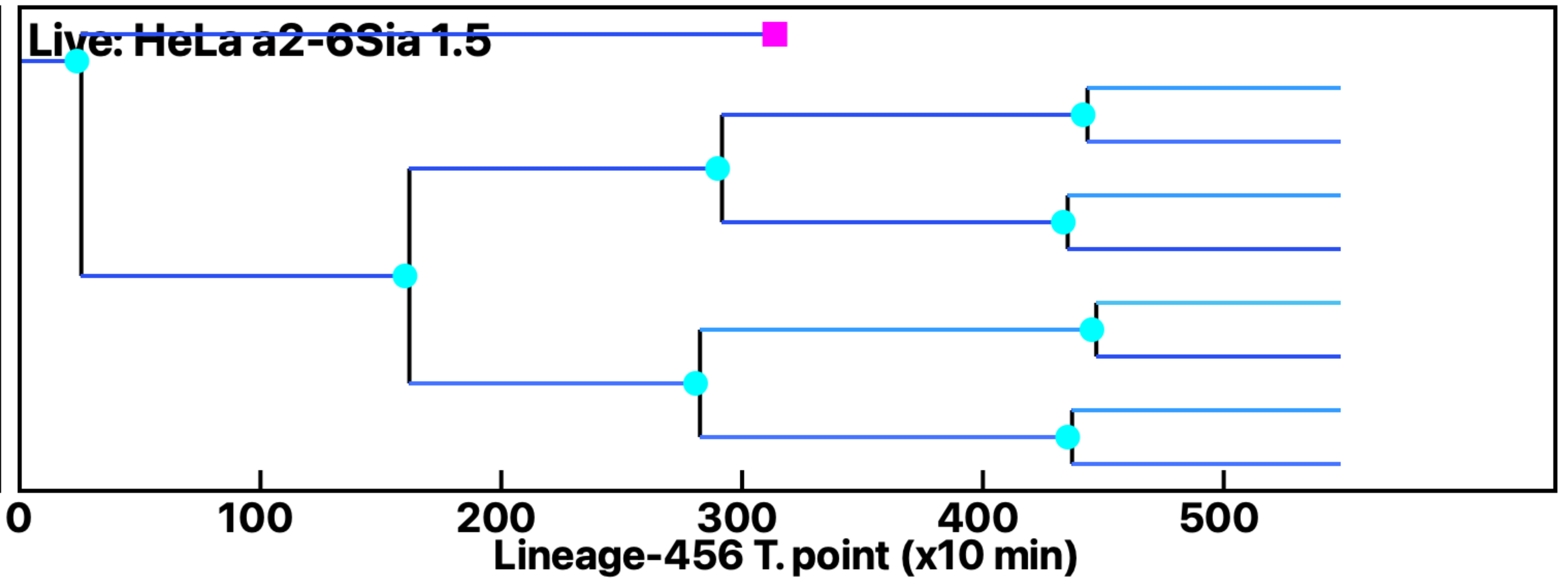

Analysis: Simulation, Treat.: HeLa a2-6Sia 1.5, Cell: HeLa-Simulation

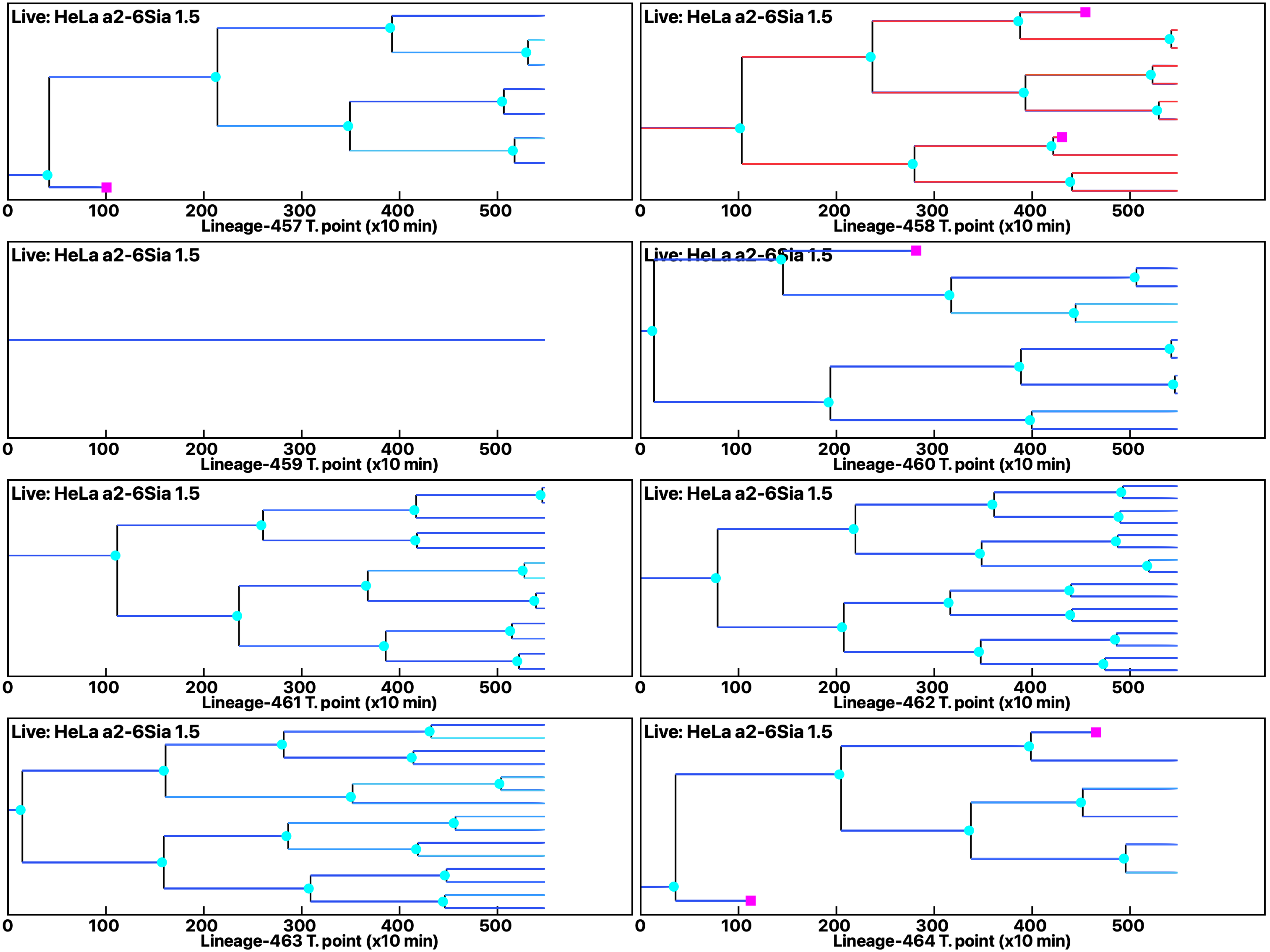

Analysis: Simulation, Treat.: HeLa a2-6Sia 1.5, Cell: HeLa-Simulation

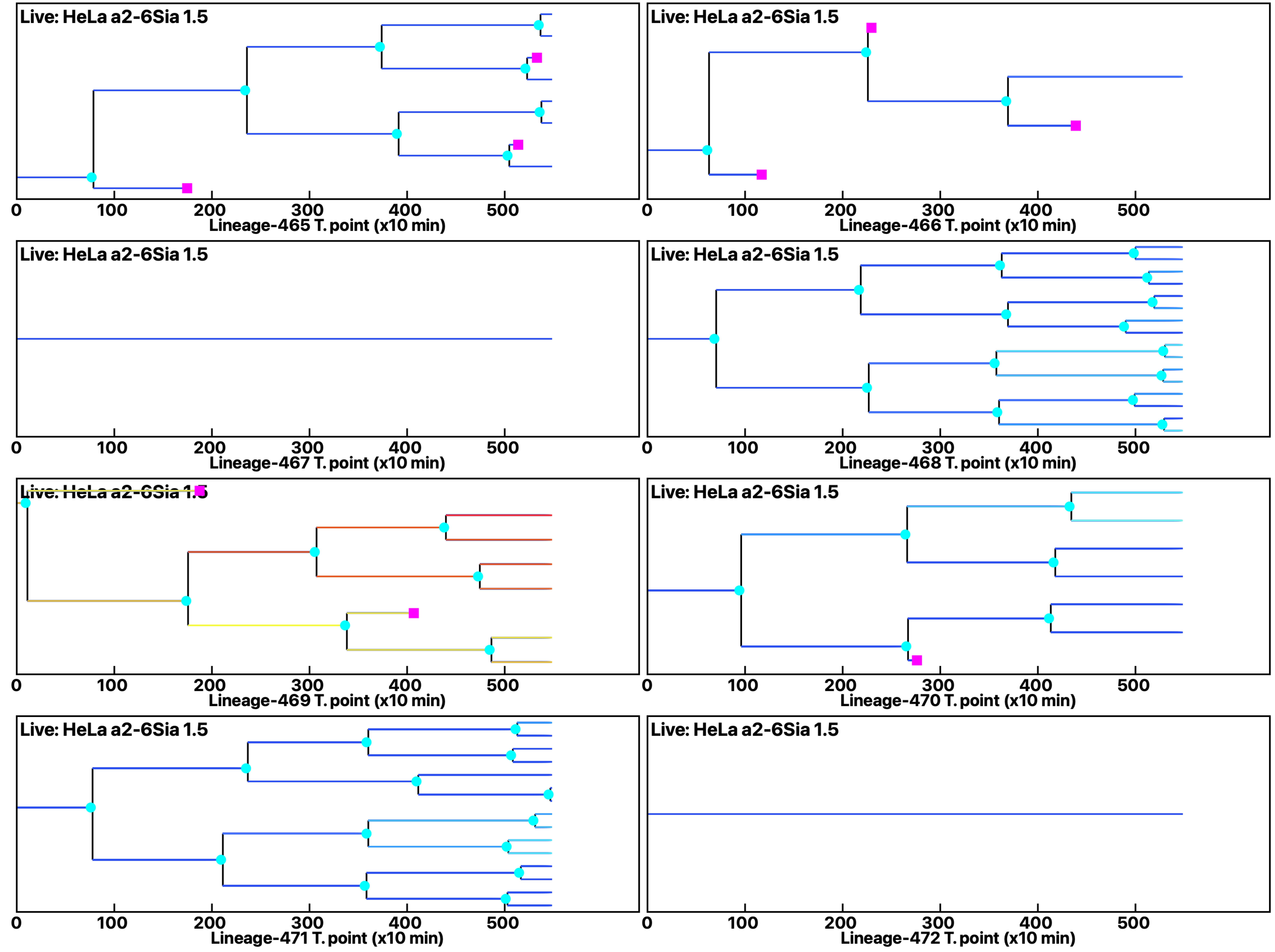

Analysis: Simulation, Treat.: HeLa a2-6Sia 1.5, Cell: HeLa-Simulation

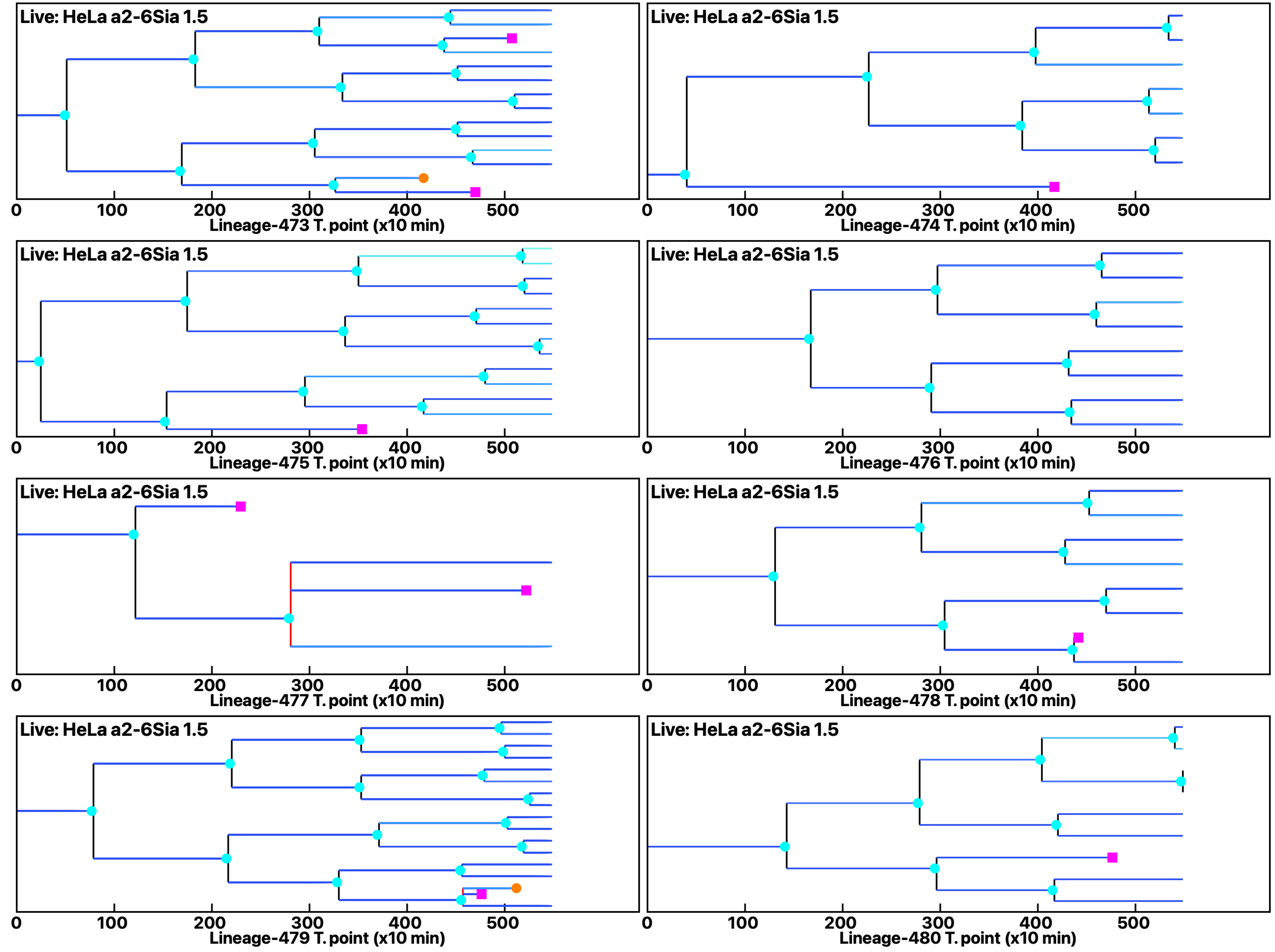

Analysis: Simulation, Treat.: HeLa a2-6Sia 1.5, Cell: HeLa-Simulation

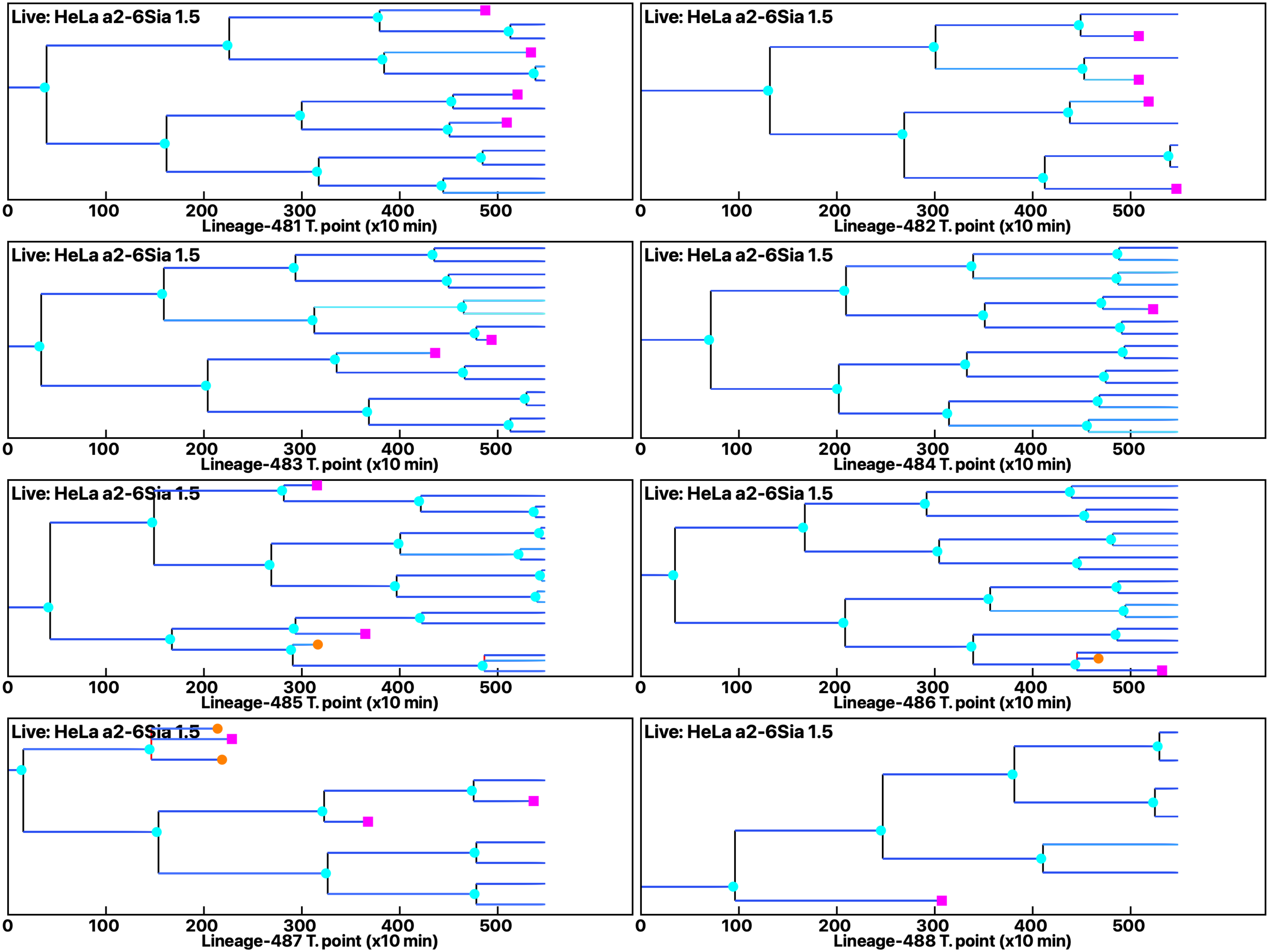

Analysis: Simulation, Treat.: HeLa a2-6Sia 1.5, Cell: HeLa-Simulation

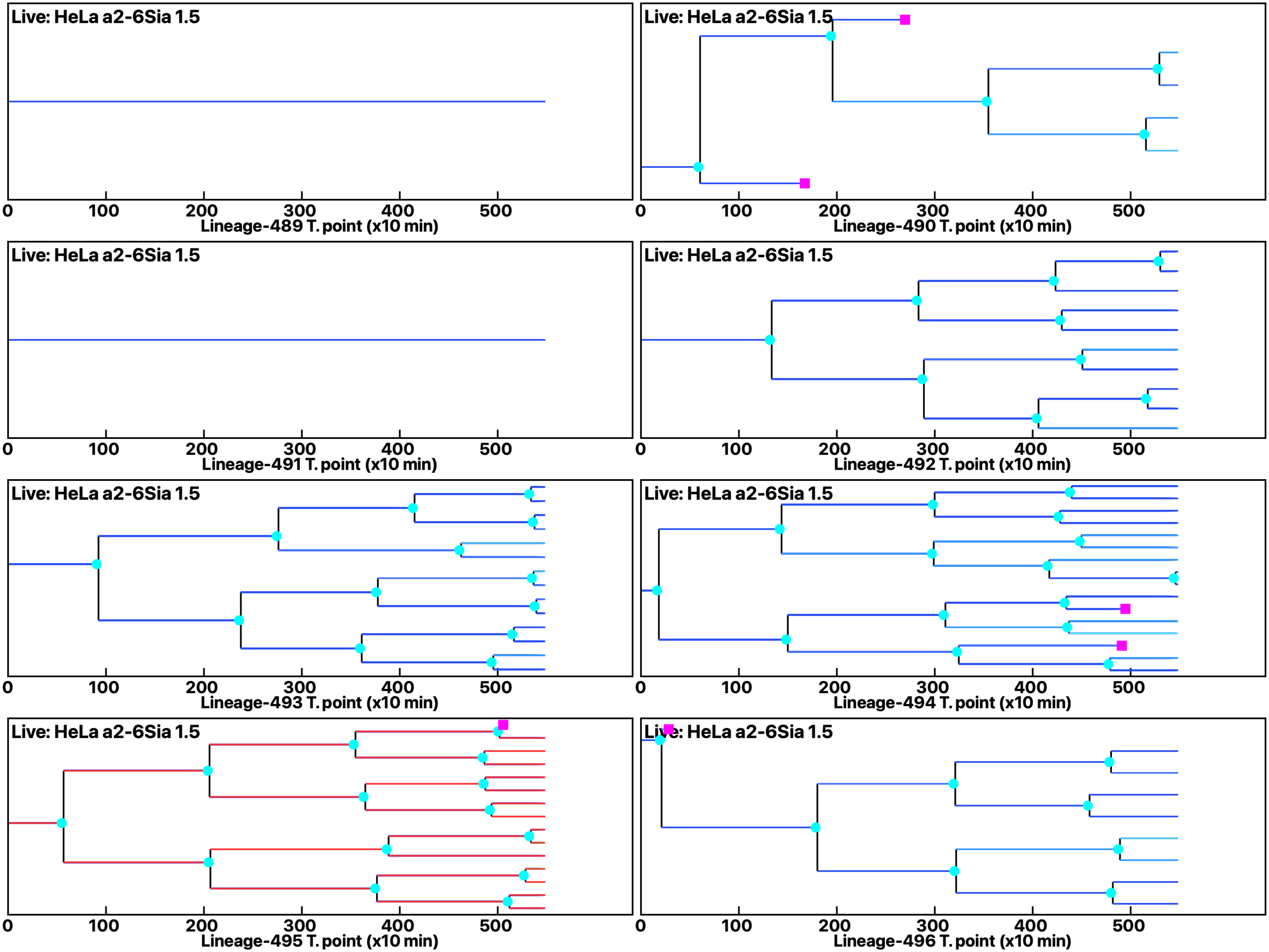

Analysis: Simulation, Treat.: HeLa a2-6Sia 1.5, Cell: HeLa-Simulation

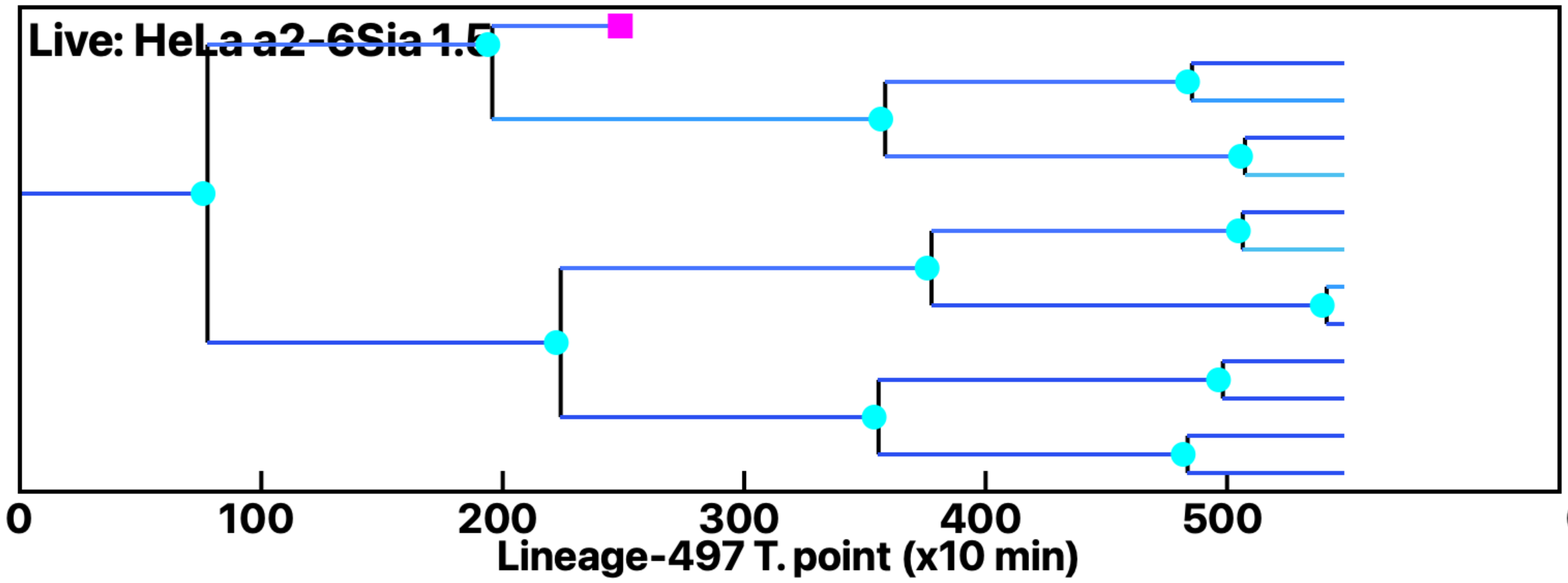

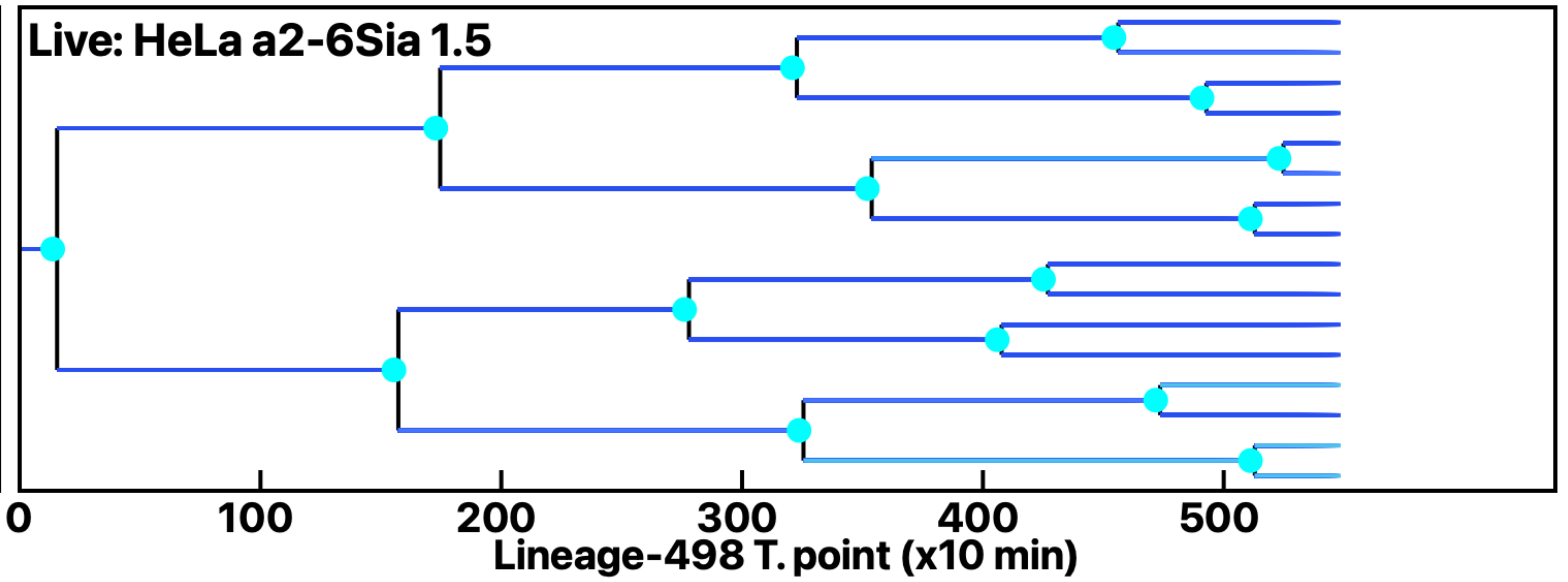

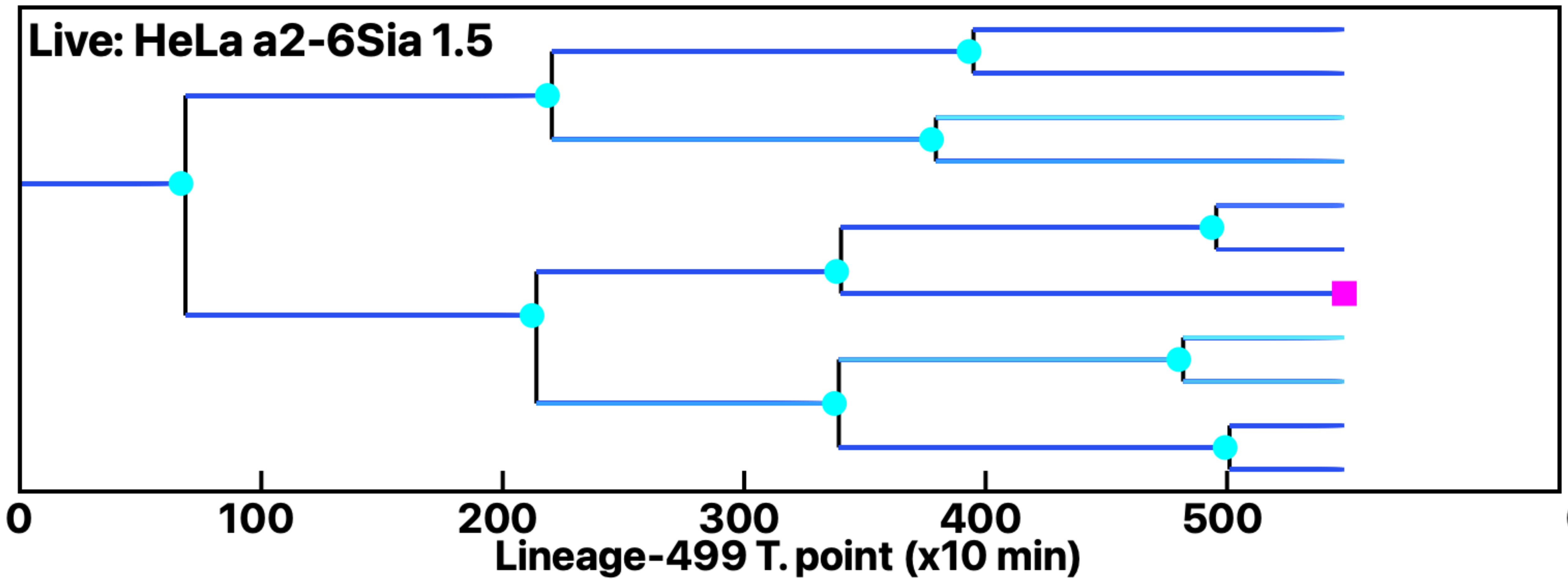

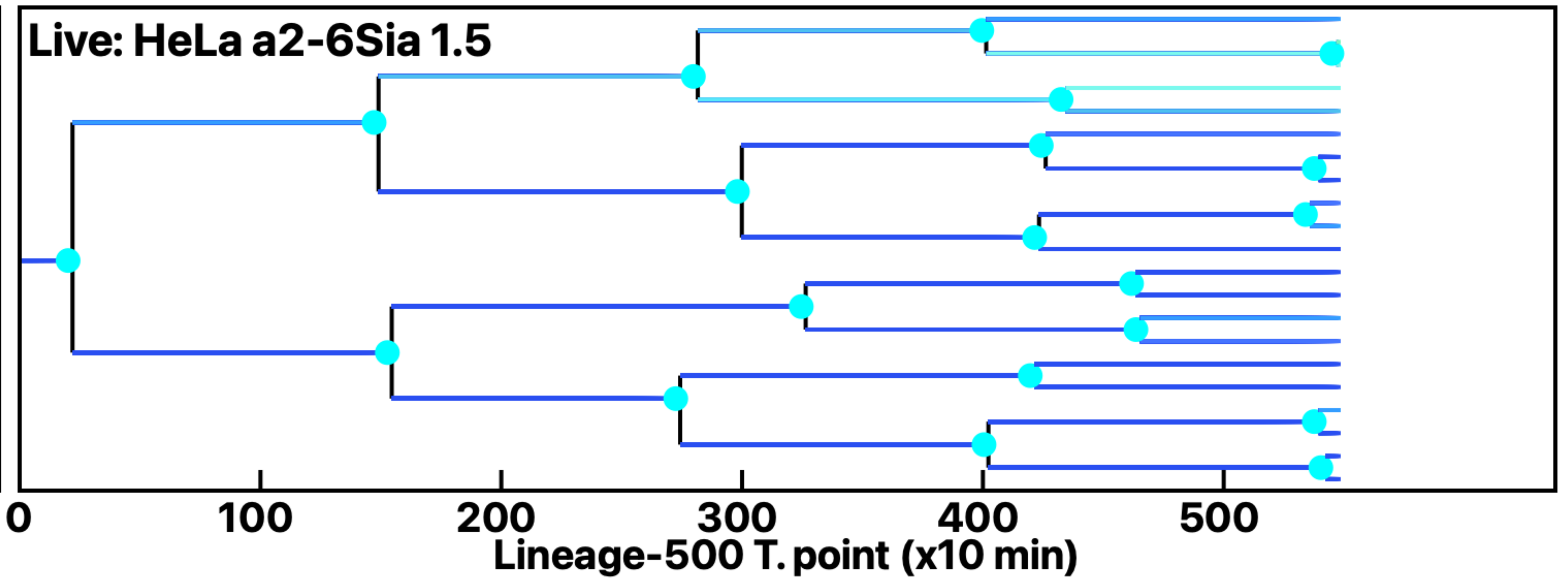

Supplement: Data S5 [file mmc7.pdf]
