# Supplementary material for: Cell fate simulation reveals cancer cell features in the tumor microenvironment
Source: J Biol Chem. 2024 Aug 20;300(9):107697. doi: 10.1016/j.jbc.2024.107697 (PMC11419826; doi:10.1016/j.jbc.2024.107697)

Analysis: Simulation, Treat.: MiaPaCa2 a2-6Sia 1.5, Cell: MiaPaCa2-Simulation

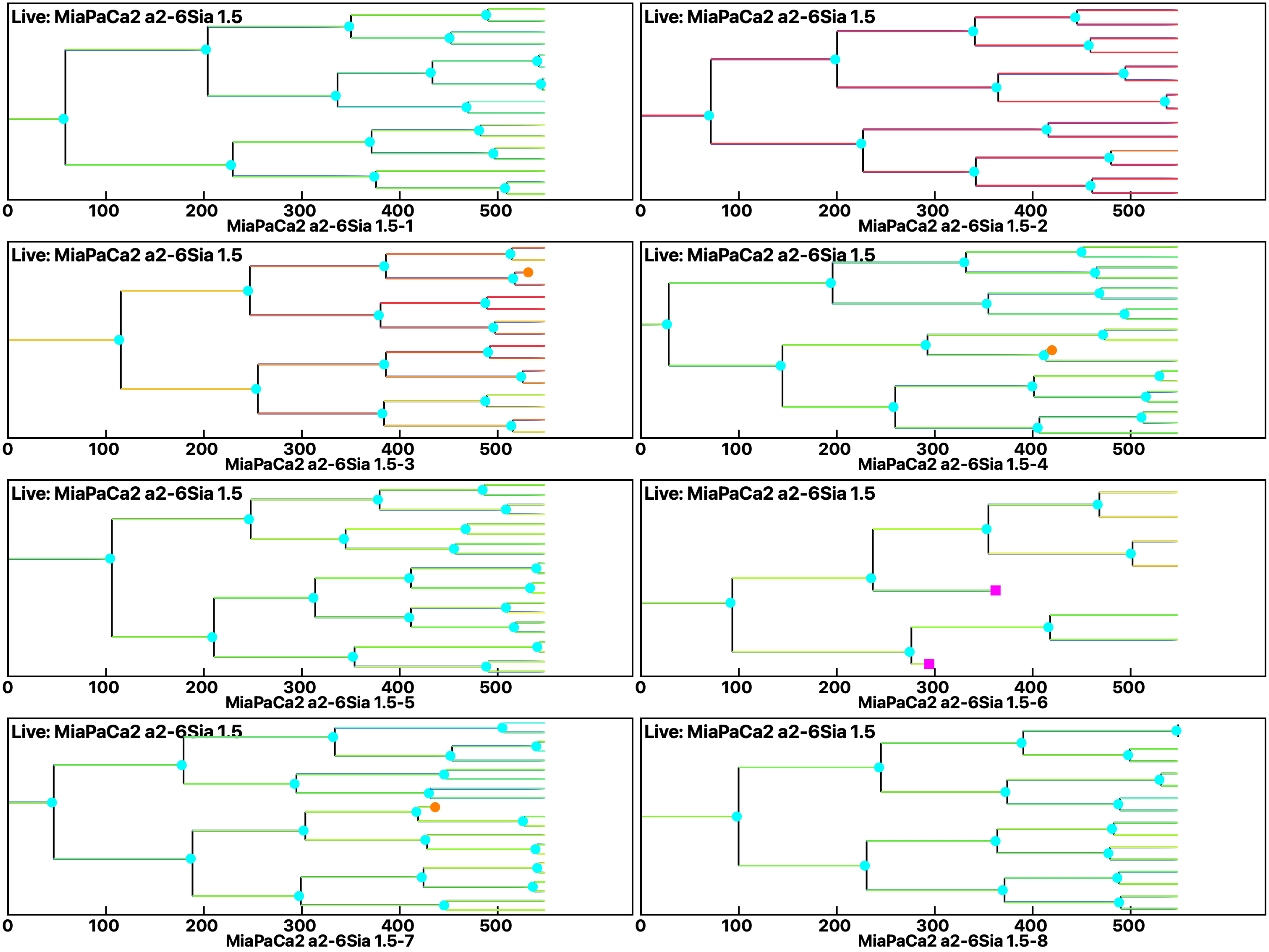

Analysis: Simulation, Treat.: MiaPaCa2 a2-6Sia 1.5, Cell: MiaPaCa2-Simulation

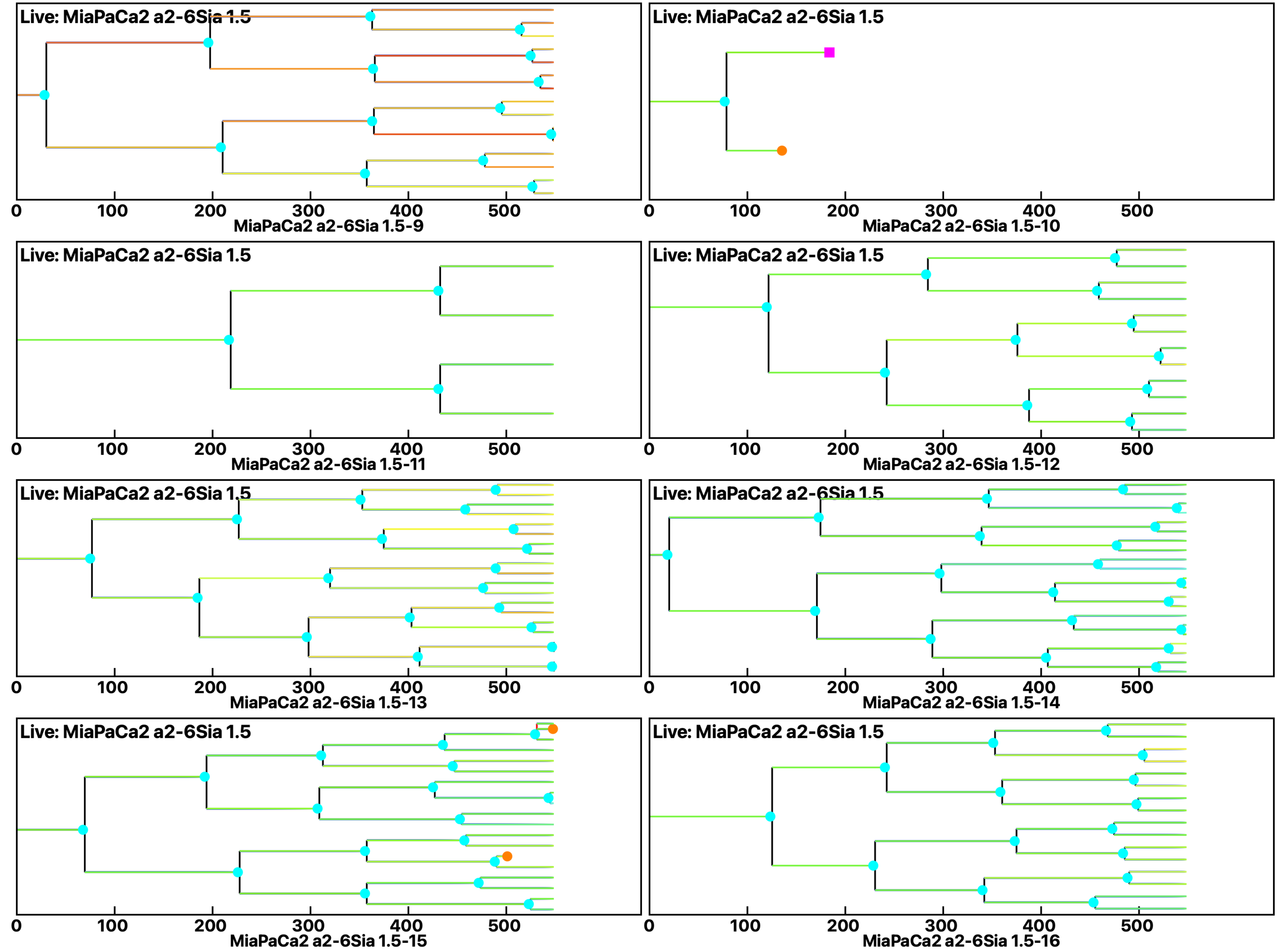

Analysis: Simulation, Treat.: MiaPaCa2 a2-6Sia 1.5, Cell: MiaPaCa2-Simulation

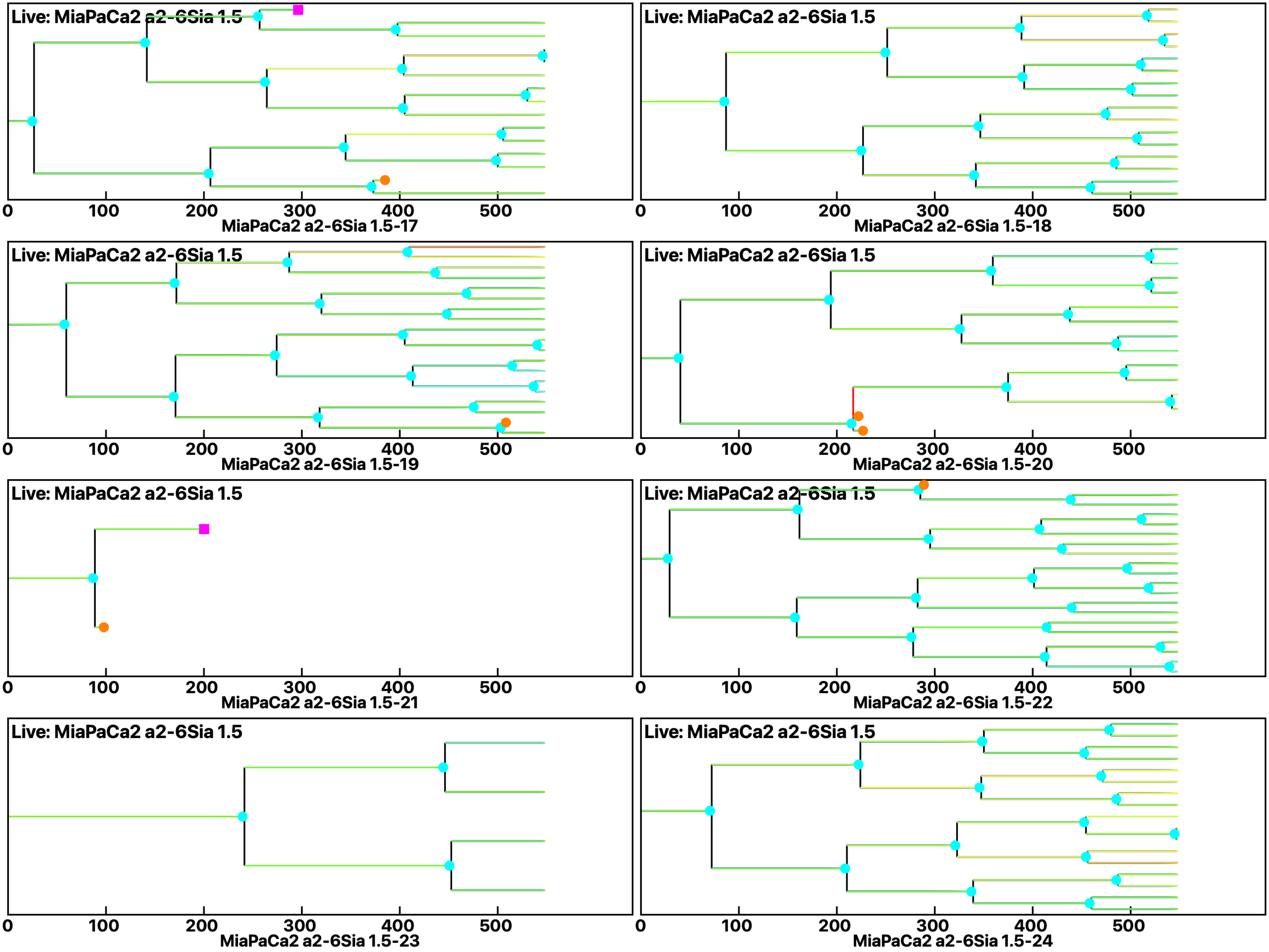

Analysis: Simulation, Treat.: MiaPaCa2 a2-6Sia 1.5, Cell: MiaPaCa2-Simulation

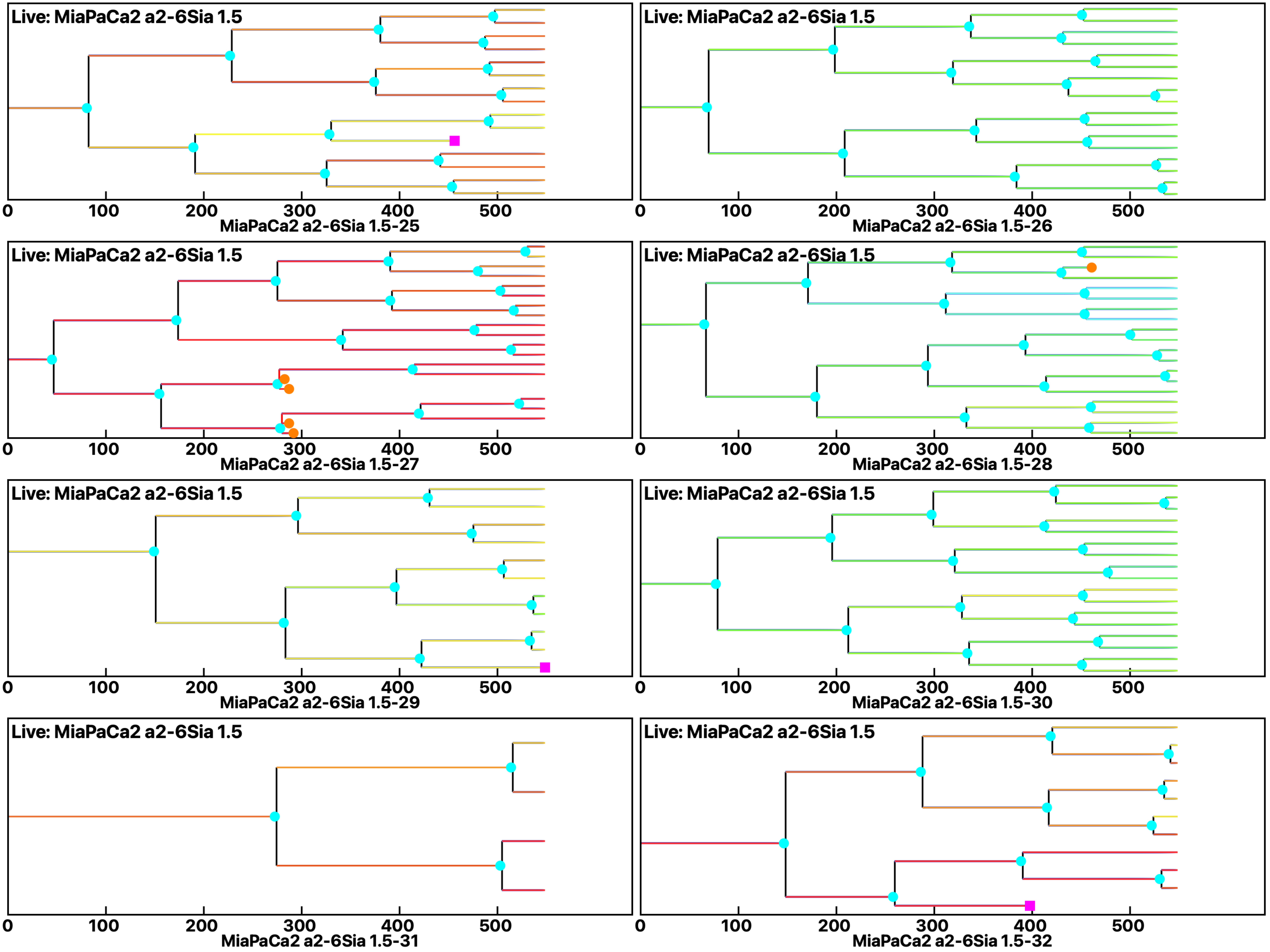

Analysis: Simulation, Treat.: MiaPaCa2 a2-6Sia 1.5, Cell: MiaPaCa2-Simulation

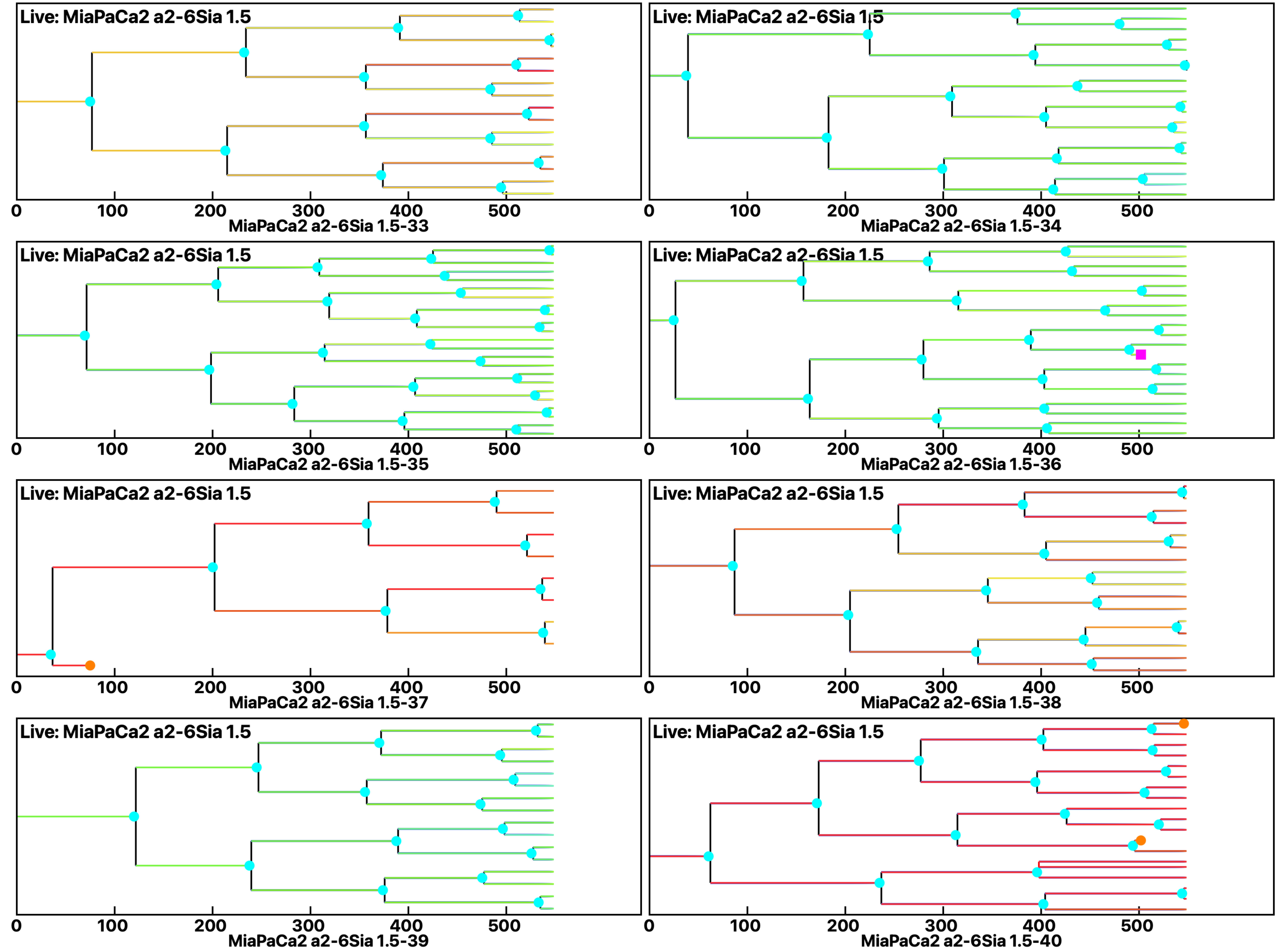

Analysis: Simulation, Treat.: MiaPaCa2 a2-6Sia 1.5, Cell: MiaPaCa2-Simulation

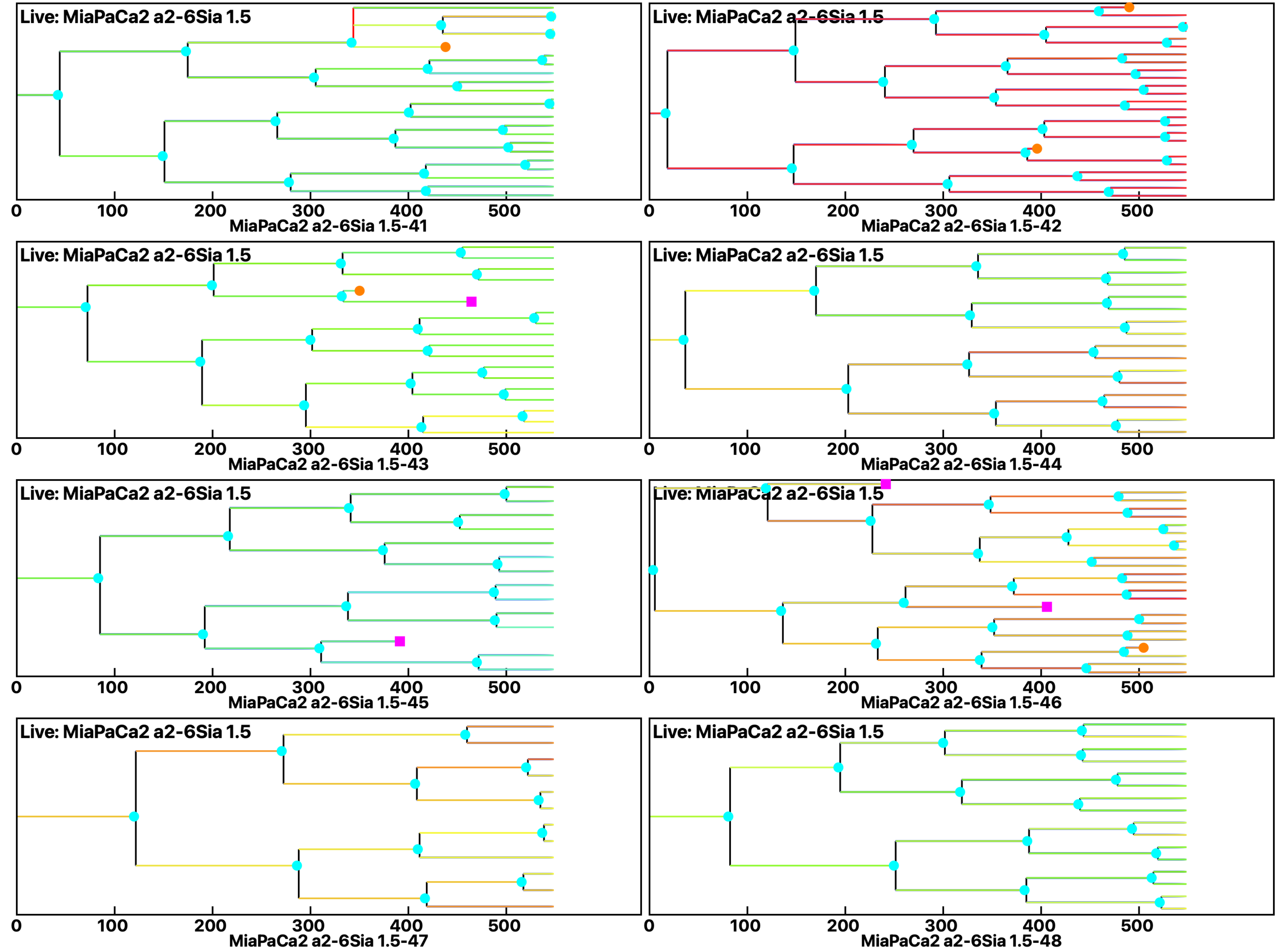

Analysis: Simulation, Treat.: MiaPaCa2 a2-6Sia 1.5, Cell: MiaPaCa2-Simulation

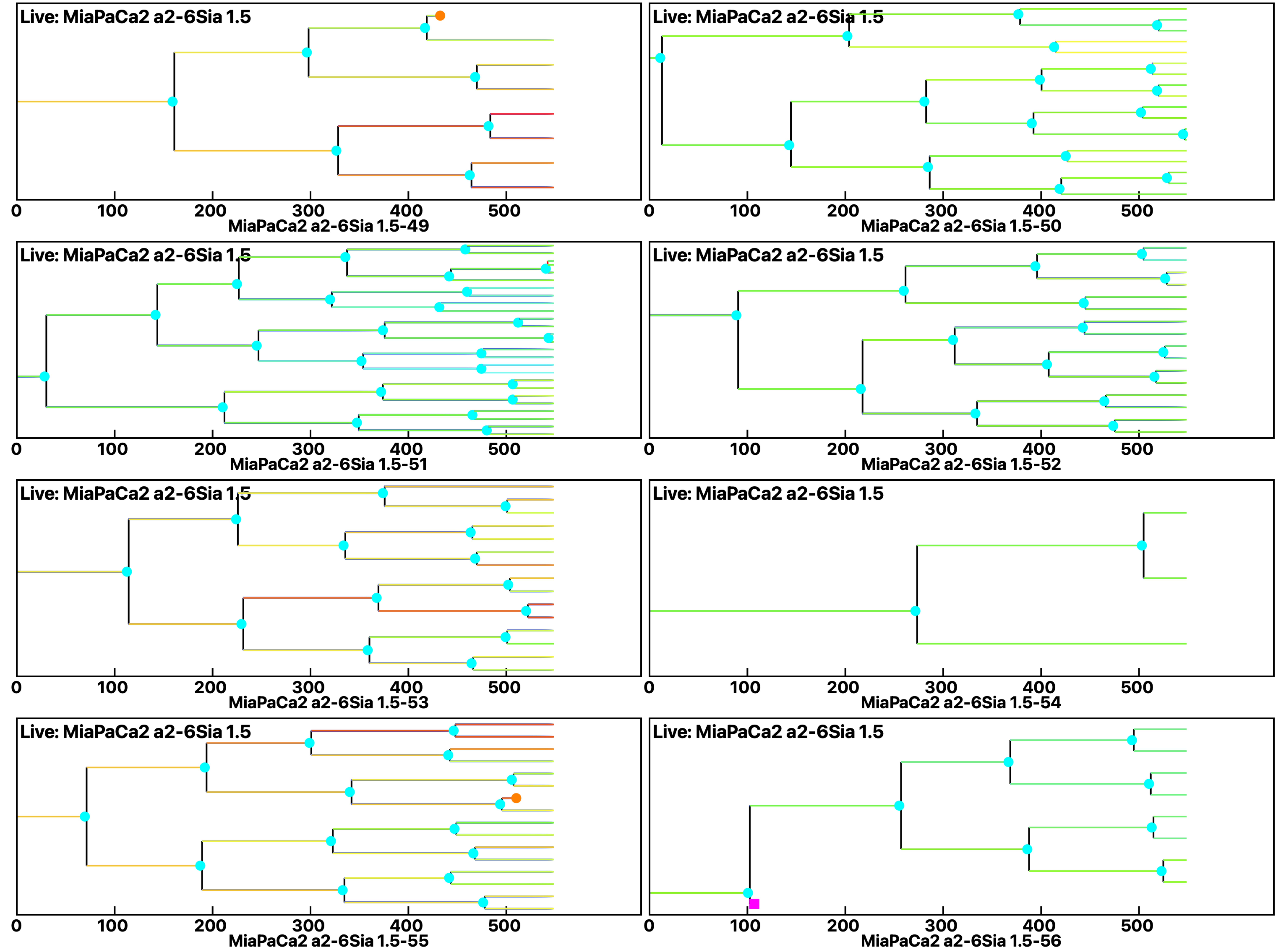

Analysis: Simulation, Treat.: MiaPaCa2 a2-6Sia 1.5, Cell: MiaPaCa2-Simulation

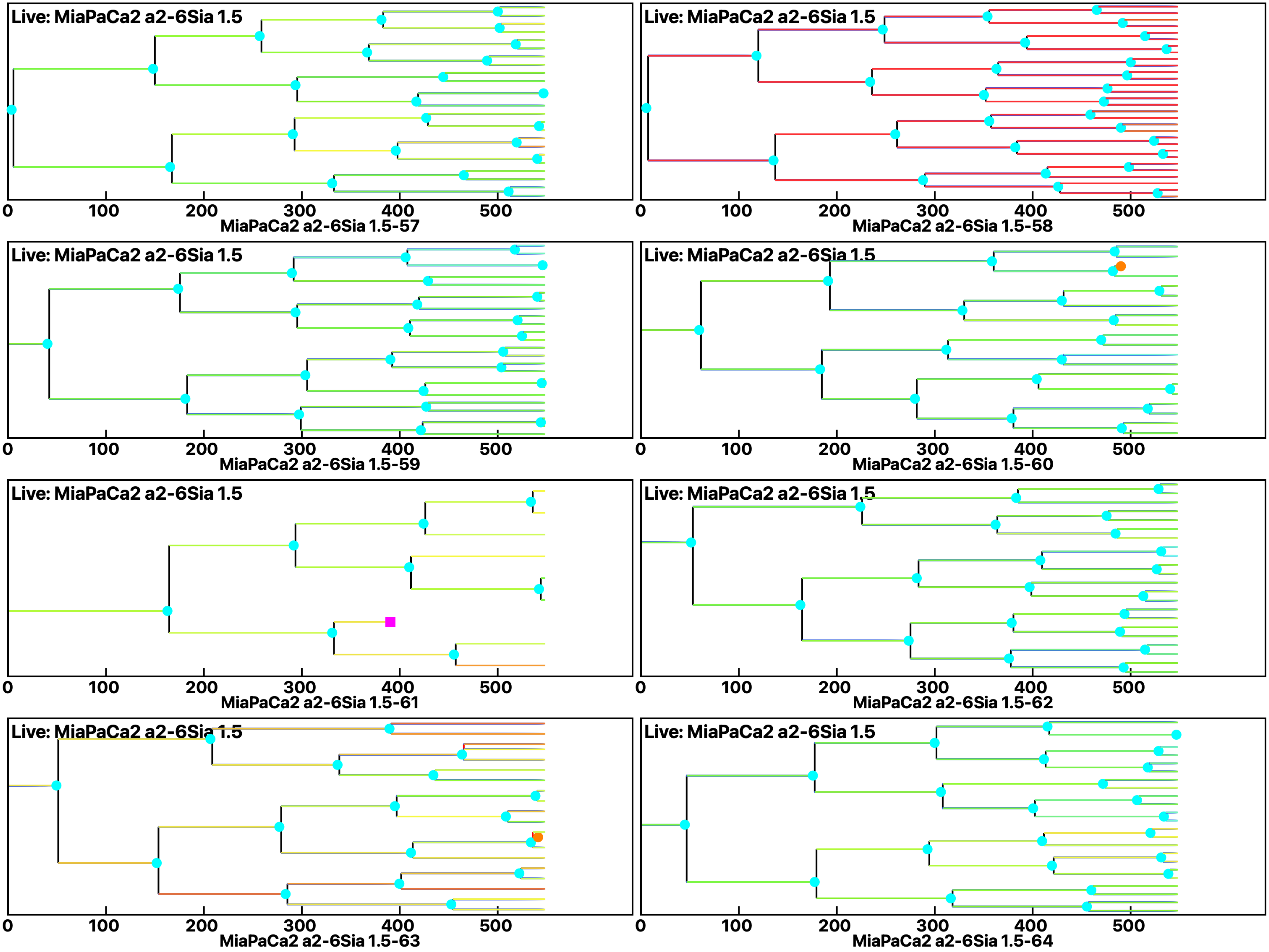

Analysis: Simulation, Treat.: MiaPaCa2 a2-6Sia 1.5, Cell: MiaPaCa2-Simulation

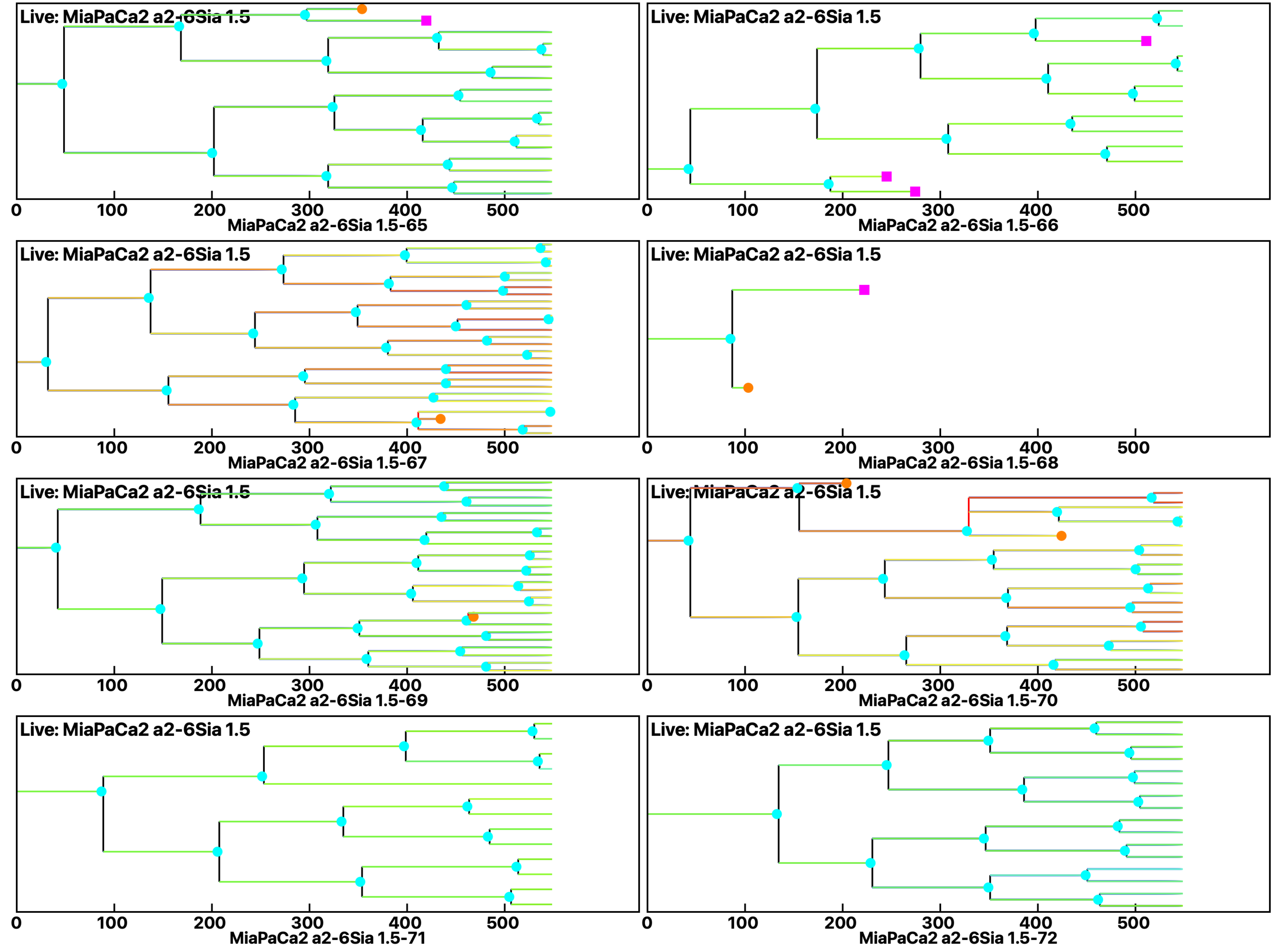

Analysis: Simulation, Treat.: MiaPaCa2 a2-6Sia 1.5, Cell: MiaPaCa2-Simulation

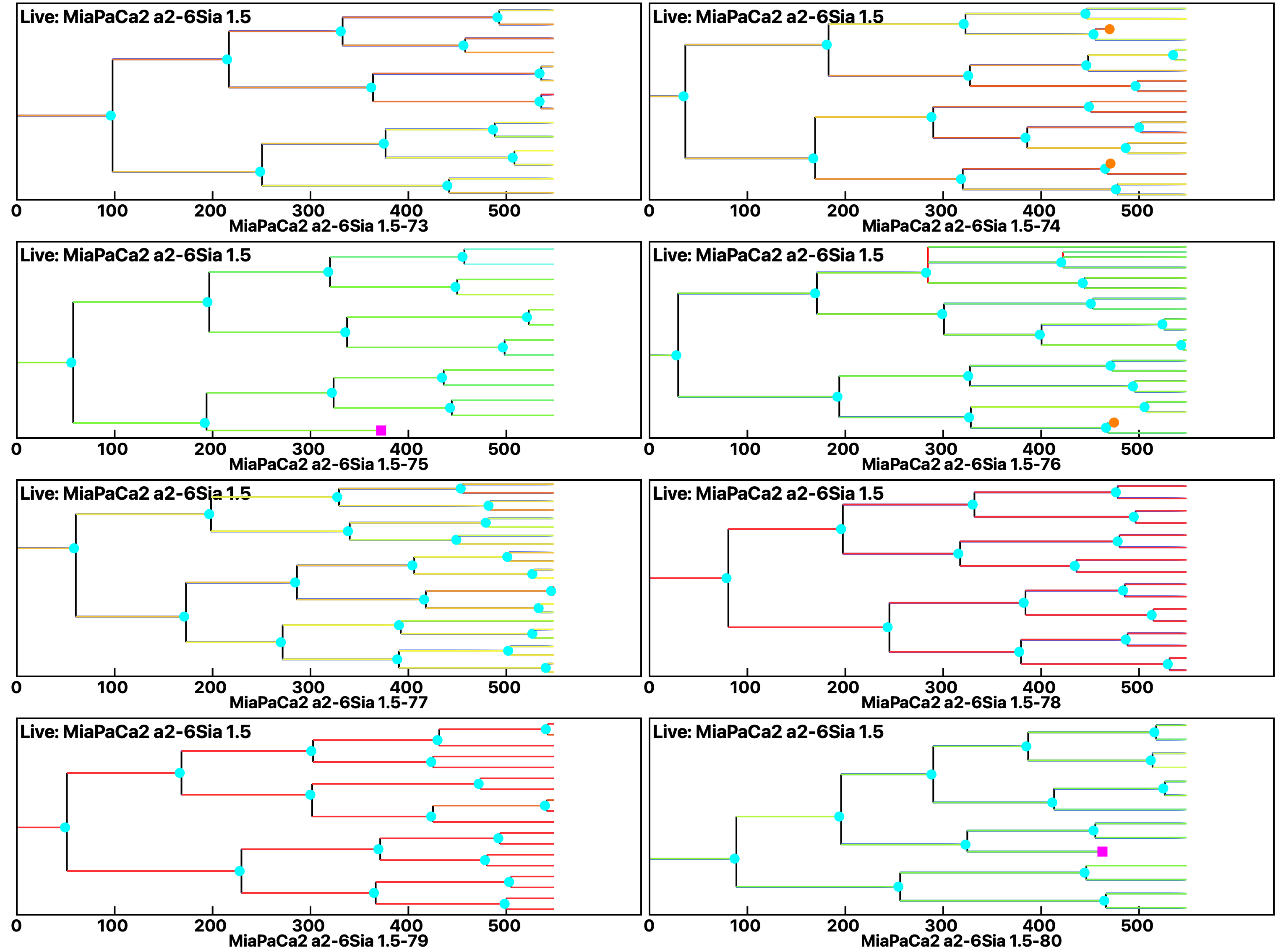

Analysis: Simulation, Treat.: MiaPaCa2 a2-6Sia 1.5, Cell: MiaPaCa2-Simulation

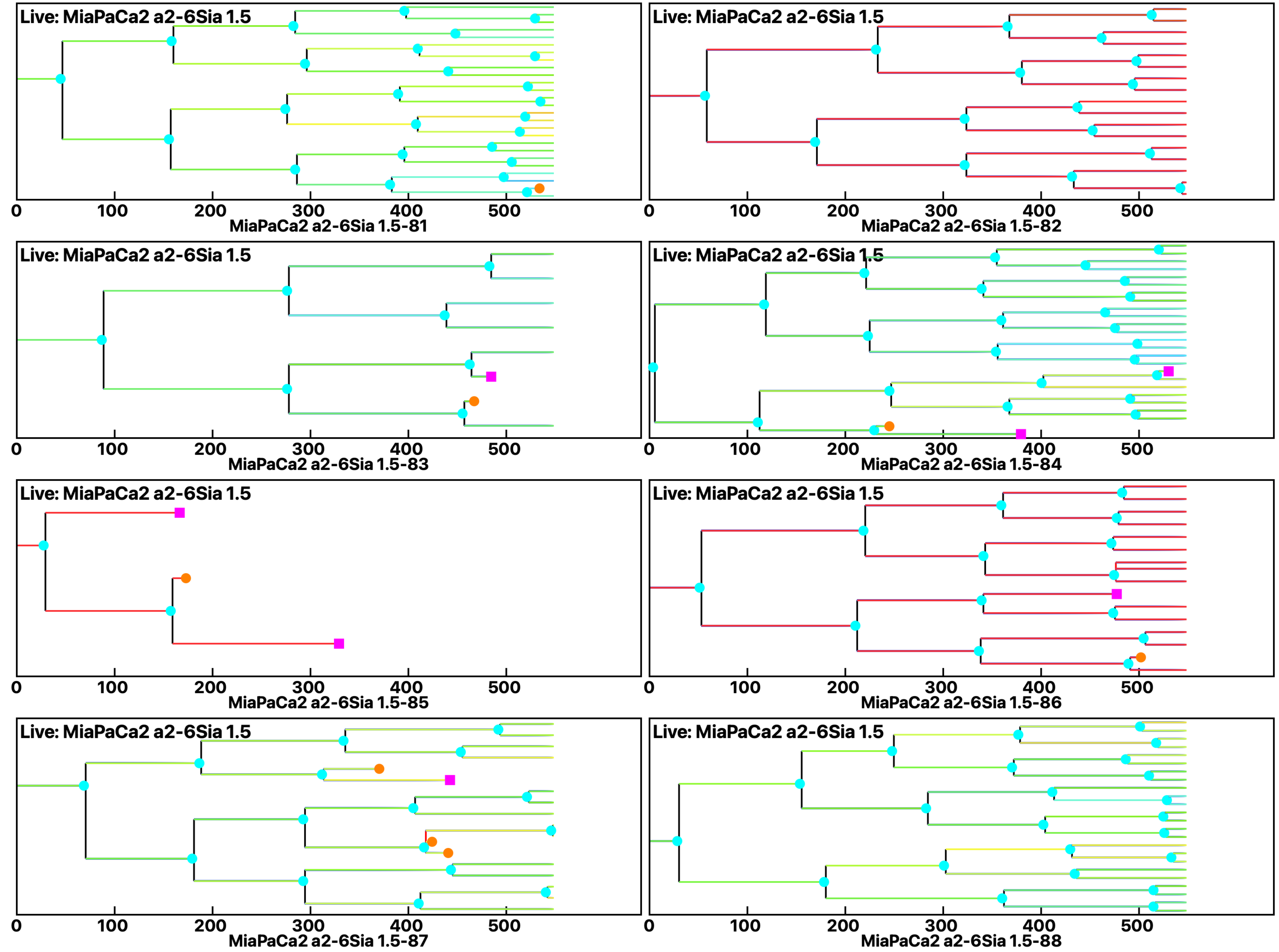

Analysis: Simulation, Treat.: MiaPaCa2 a2-6Sia 1.5, Cell: MiaPaCa2-Simulation

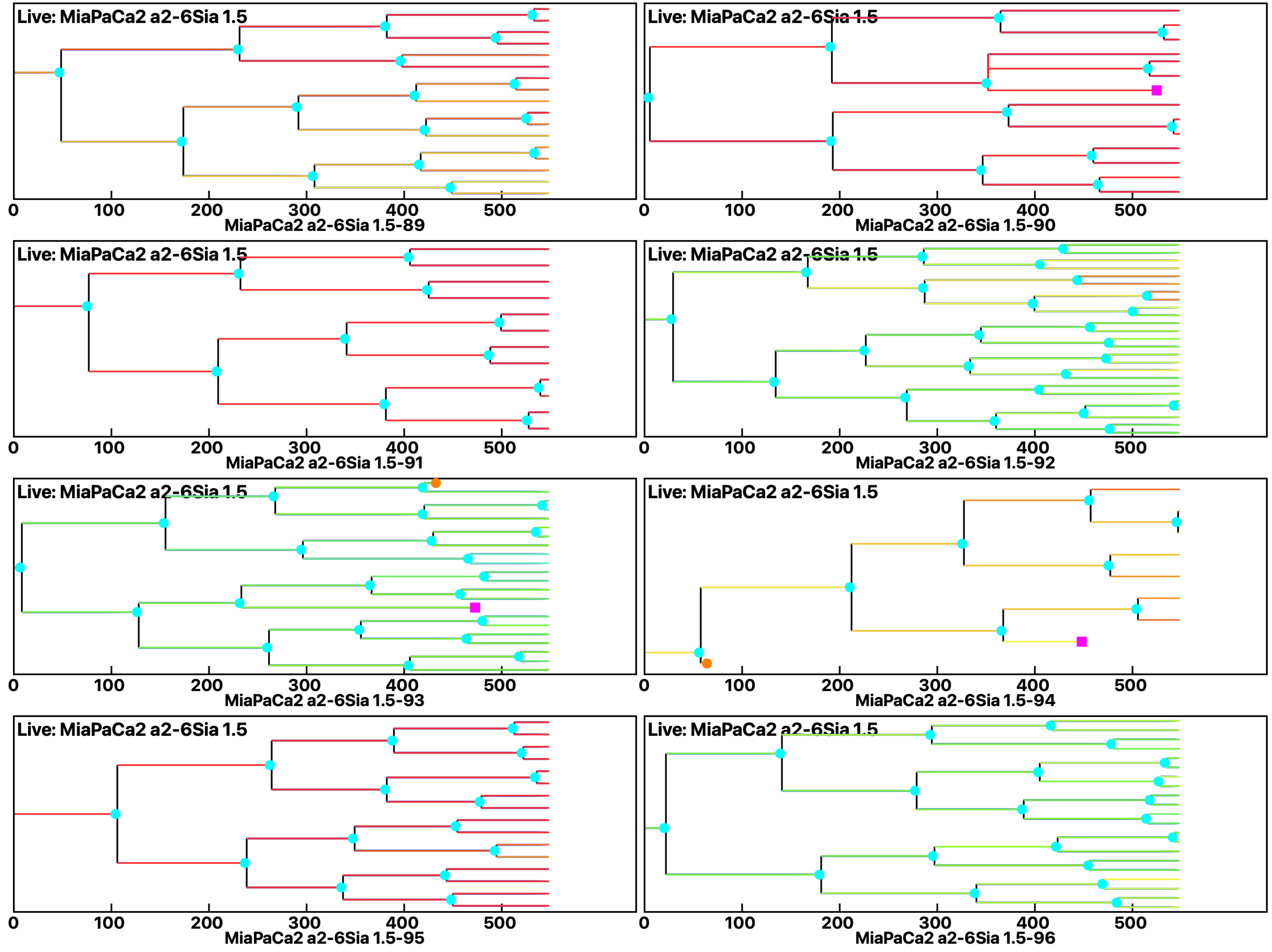

Analysis: Simulation, Treat.: MiaPaCa2 a2-6Sia 1.5, Cell: MiaPaCa2-Simulation

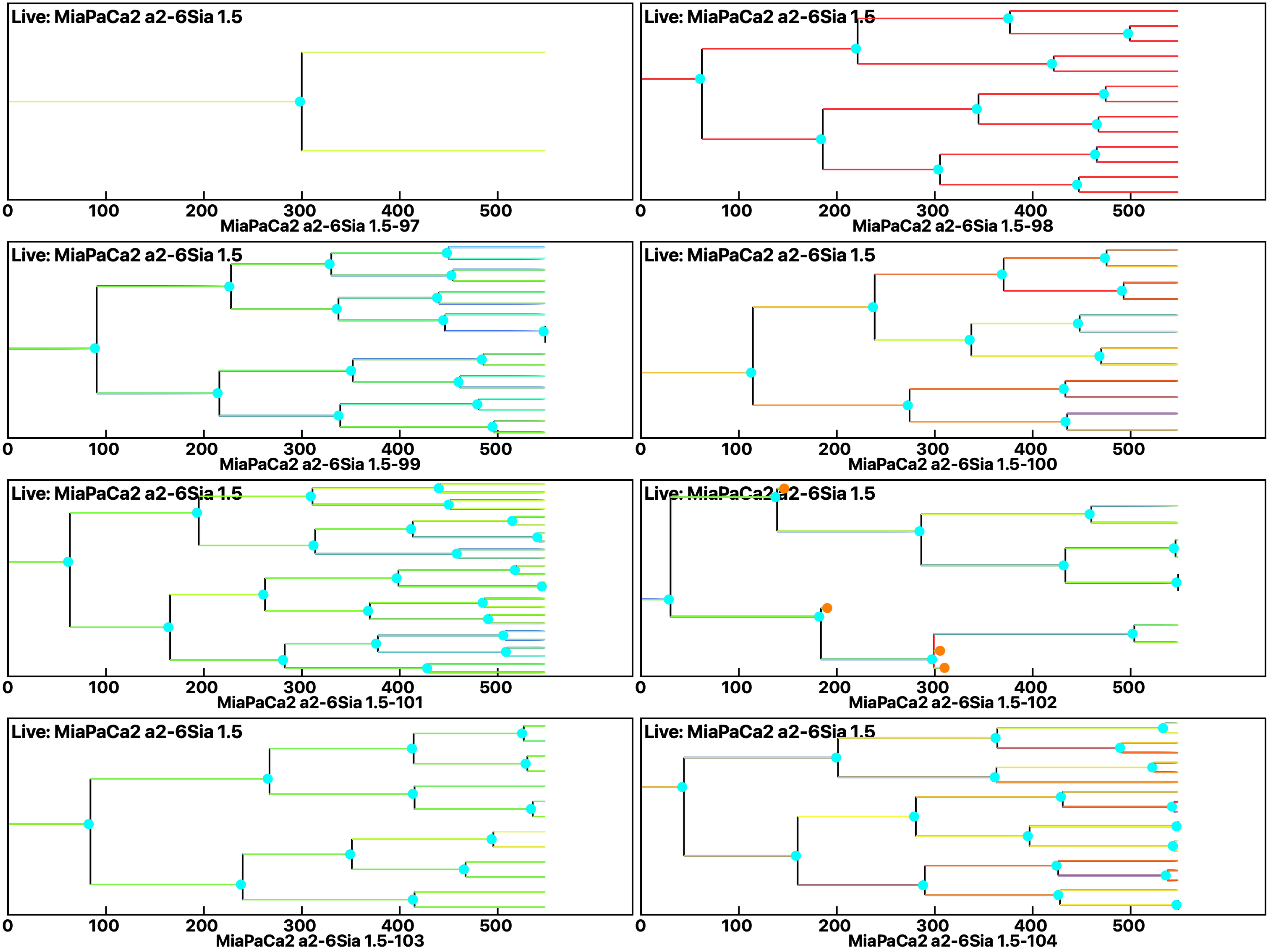

Analysis: Simulation, Treat.: MiaPaCa2 a2-6Sia 1.5, Cell: MiaPaCa2-Simulation

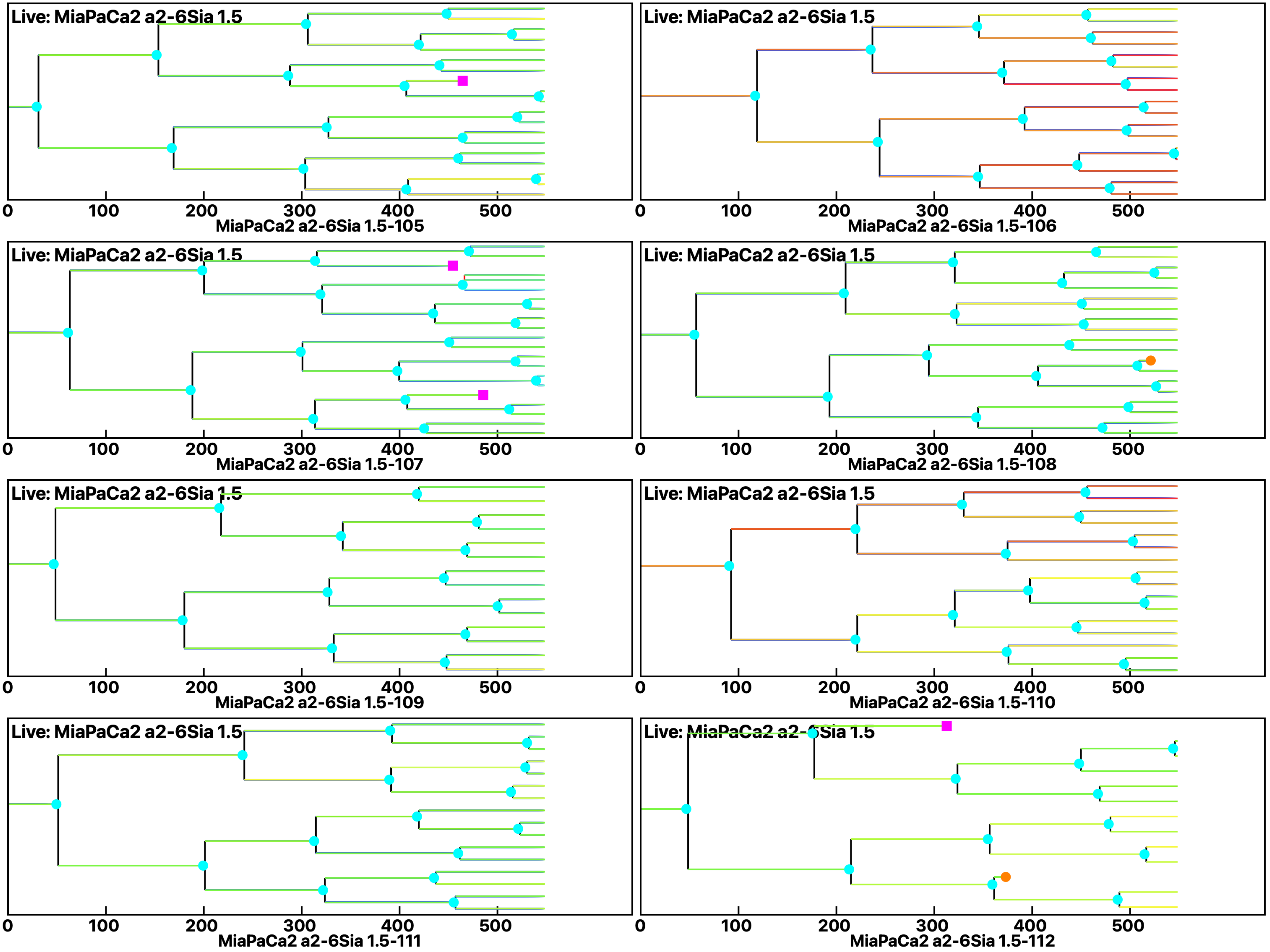

Analysis: Simulation, Treat.: MiaPaCa2 a2-6Sia 1.5, Cell: MiaPaCa2-Simulation

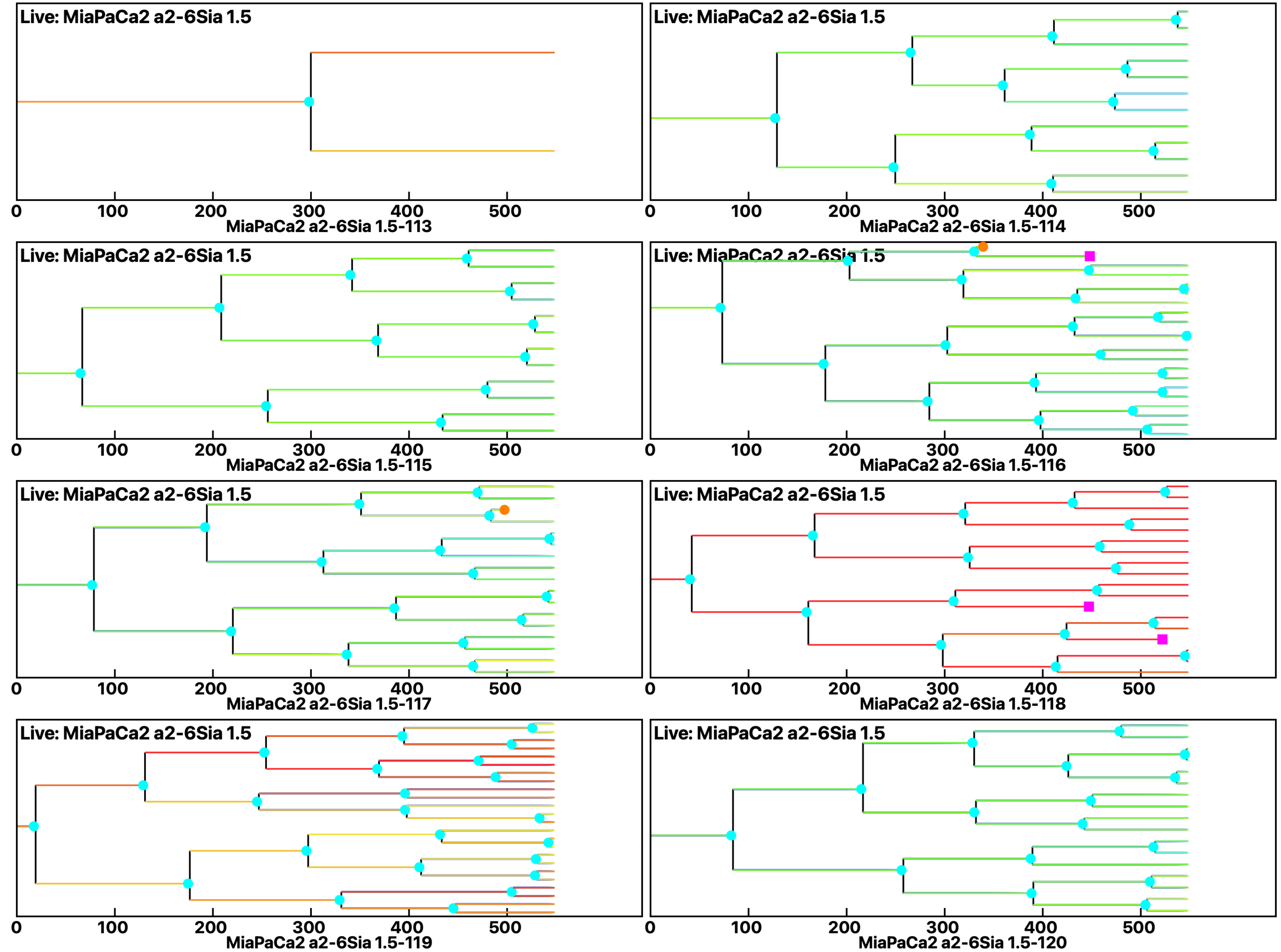

Analysis: Simulation, Treat.: MiaPaCa2 a2-6Sia 1.5, Cell: MiaPaCa2-Simulation

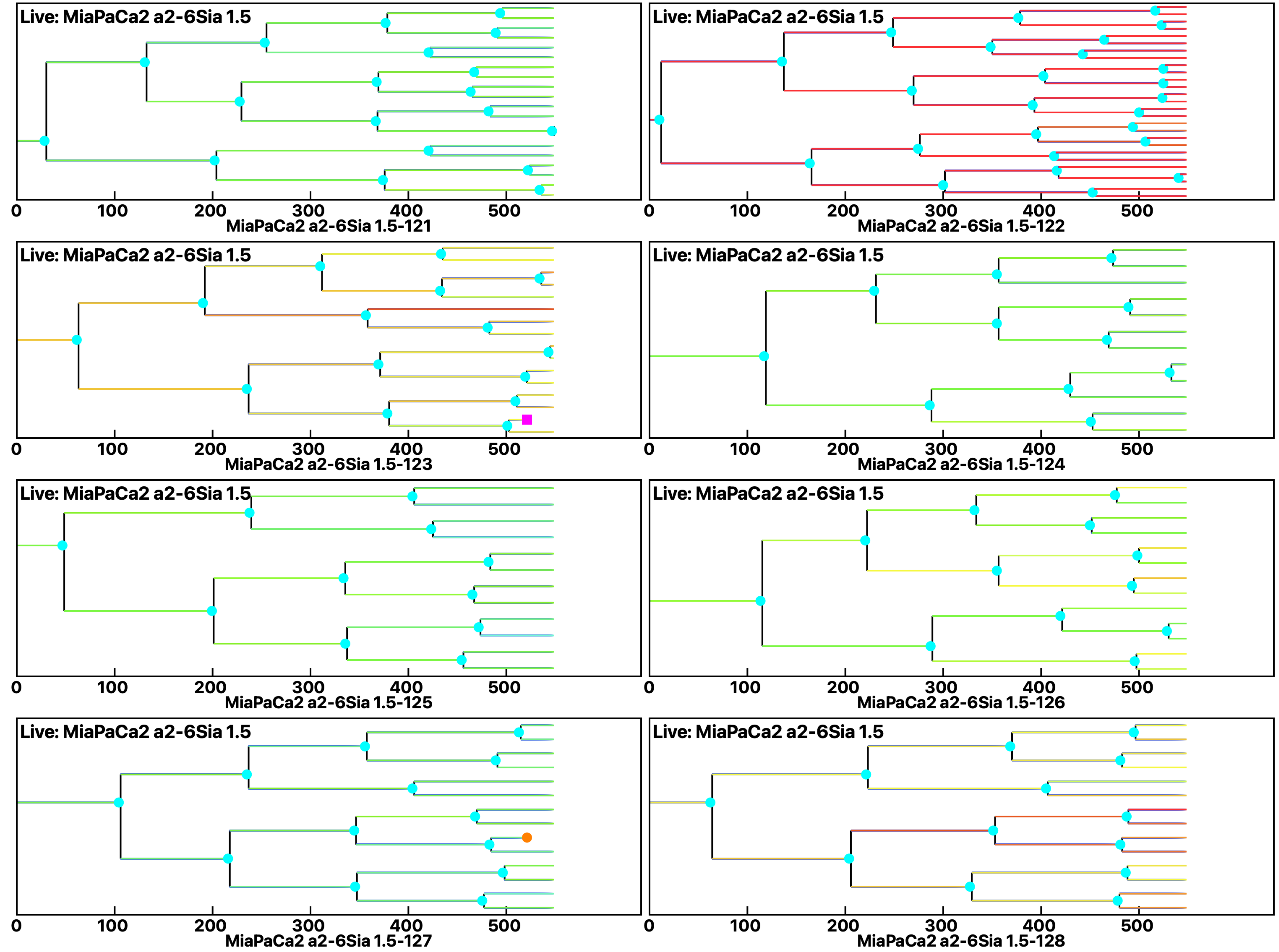

Analysis: Simulation, Treat.: MiaPaCa2 a2-6Sia 1.5, Cell: MiaPaCa2-Simulation

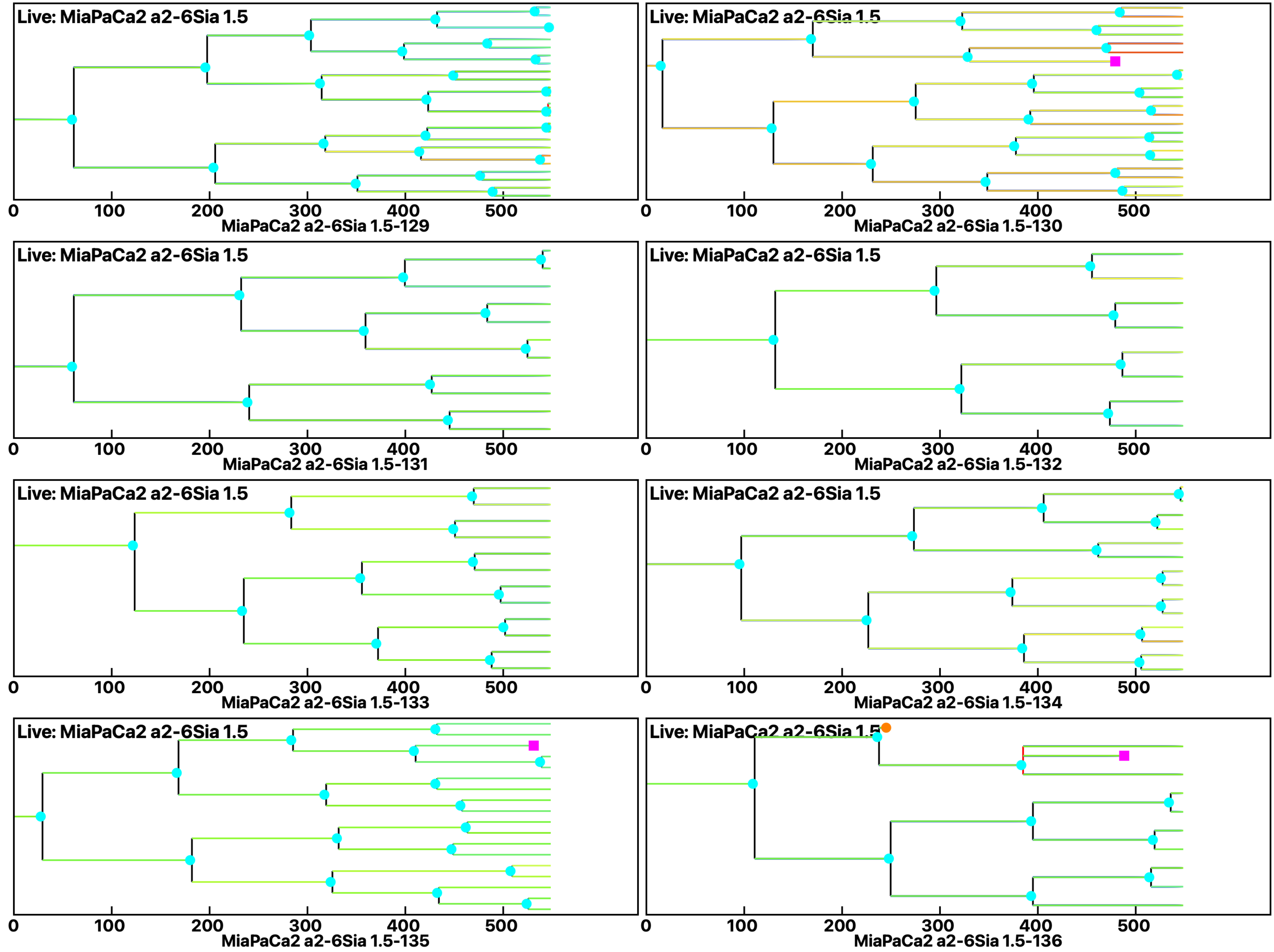

Analysis: Simulation, Treat.: MiaPaCa2 a2-6Sia 1.5, Cell: MiaPaCa2-Simulation

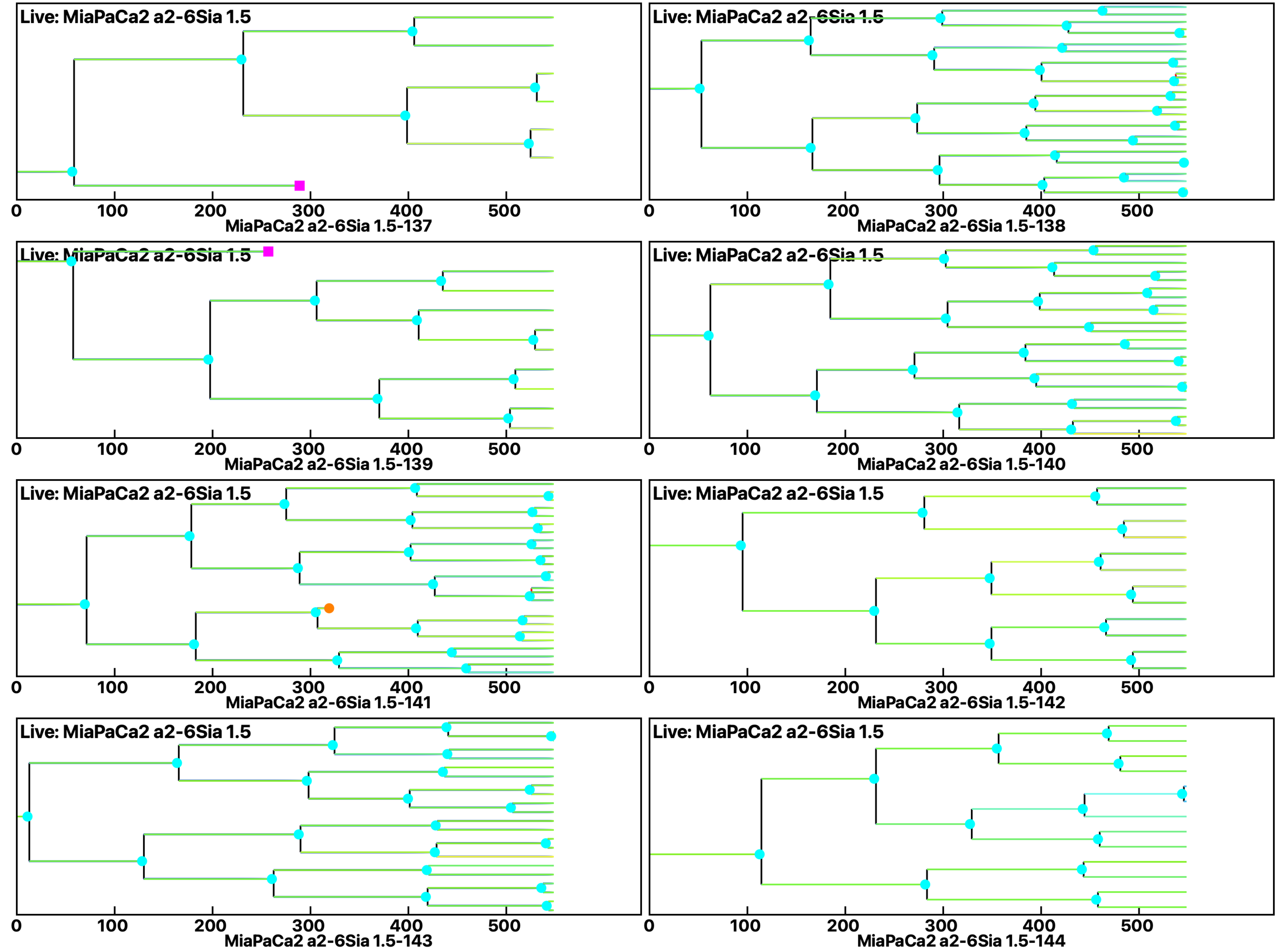

Analysis: Simulation, Treat.: MiaPaCa2 a2-6Sia 1.5, Cell: MiaPaCa2-Simulation

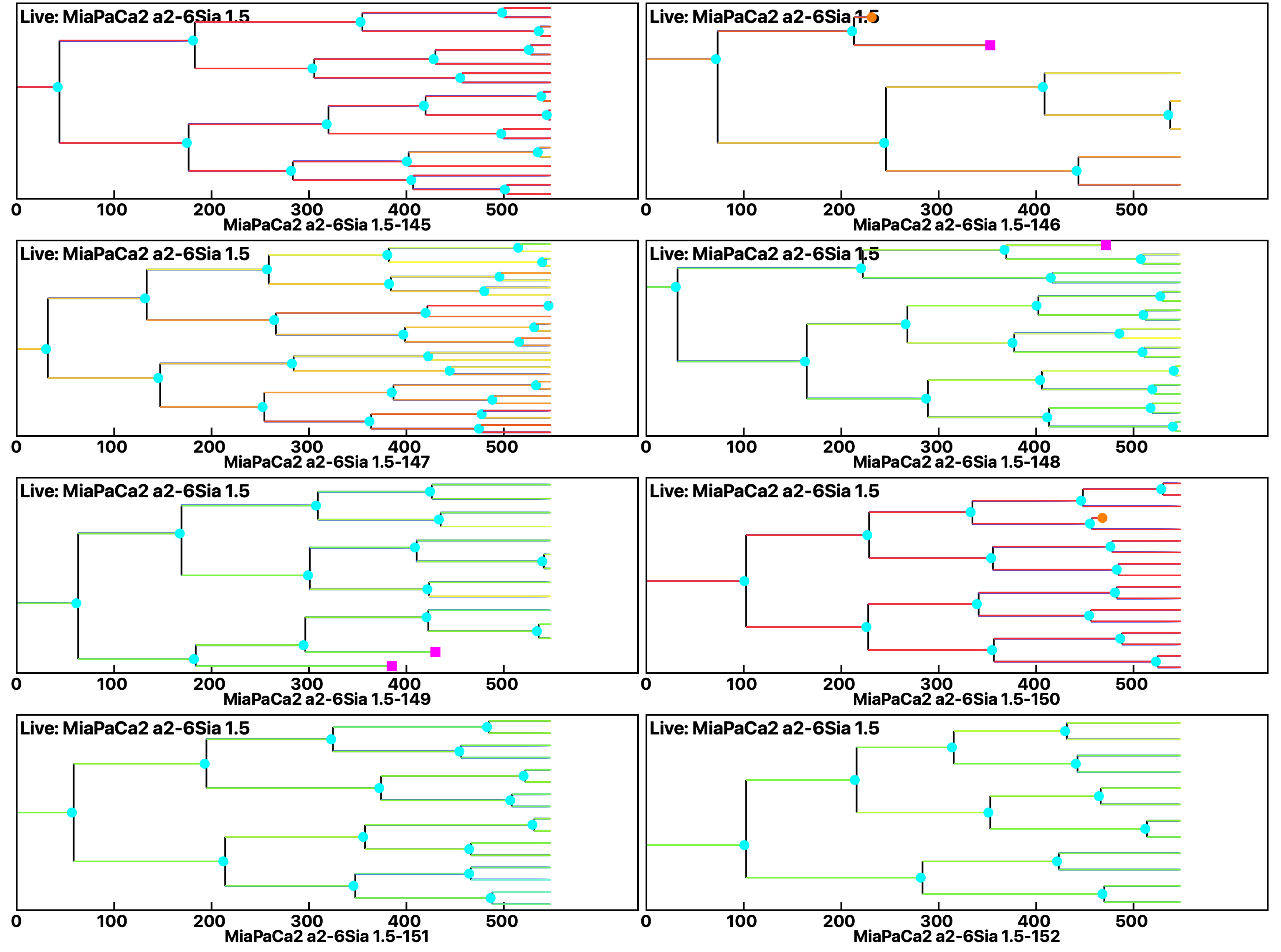

Analysis: Simulation, Treat.: MiaPaCa2 a2-6Sia 1.5, Cell: MiaPaCa2-Simulation

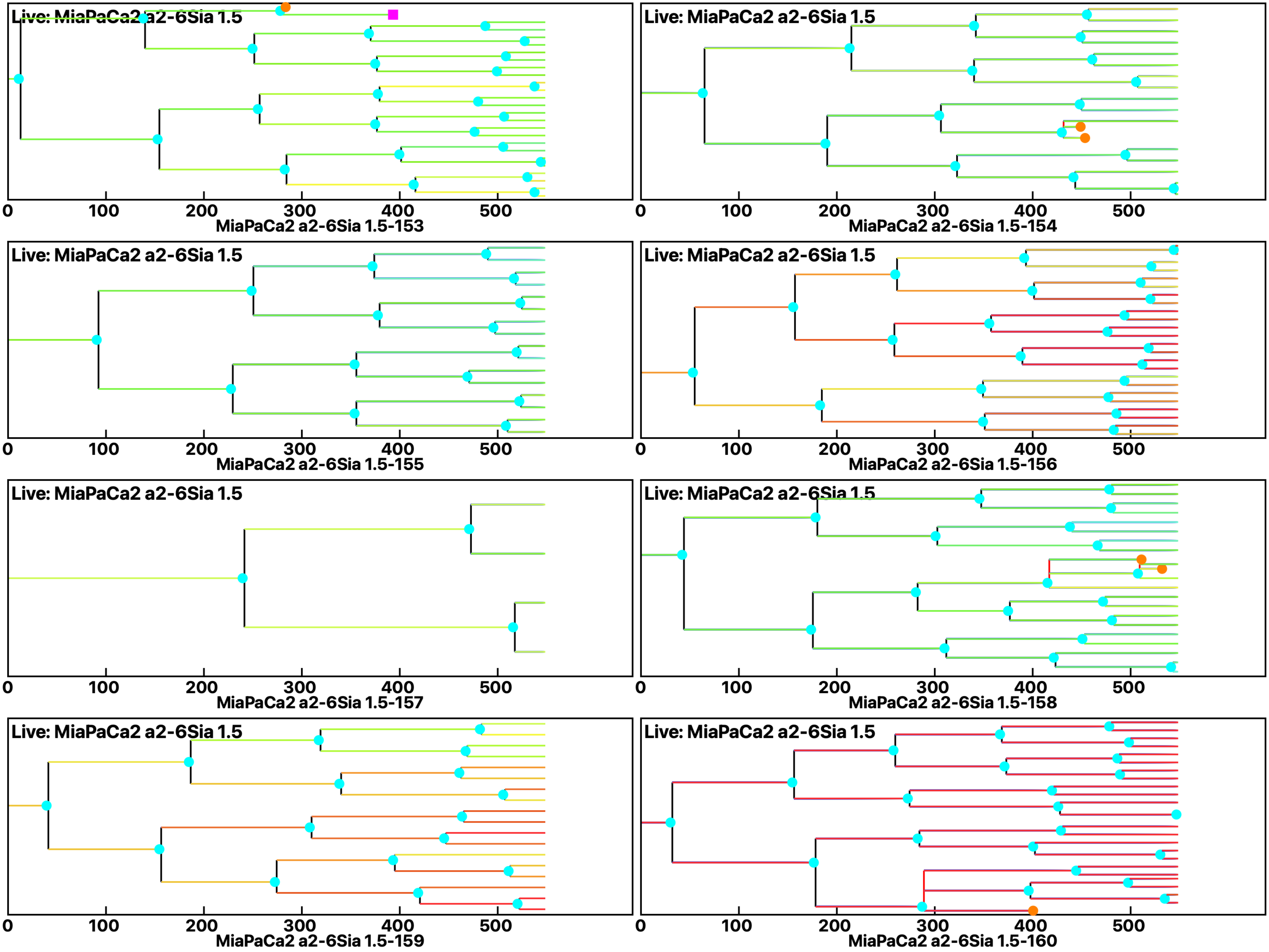

Analysis: Simulation, Treat.: MiaPaCa2 a2-6Sia 1.5, Cell: MiaPaCa2-Simulation

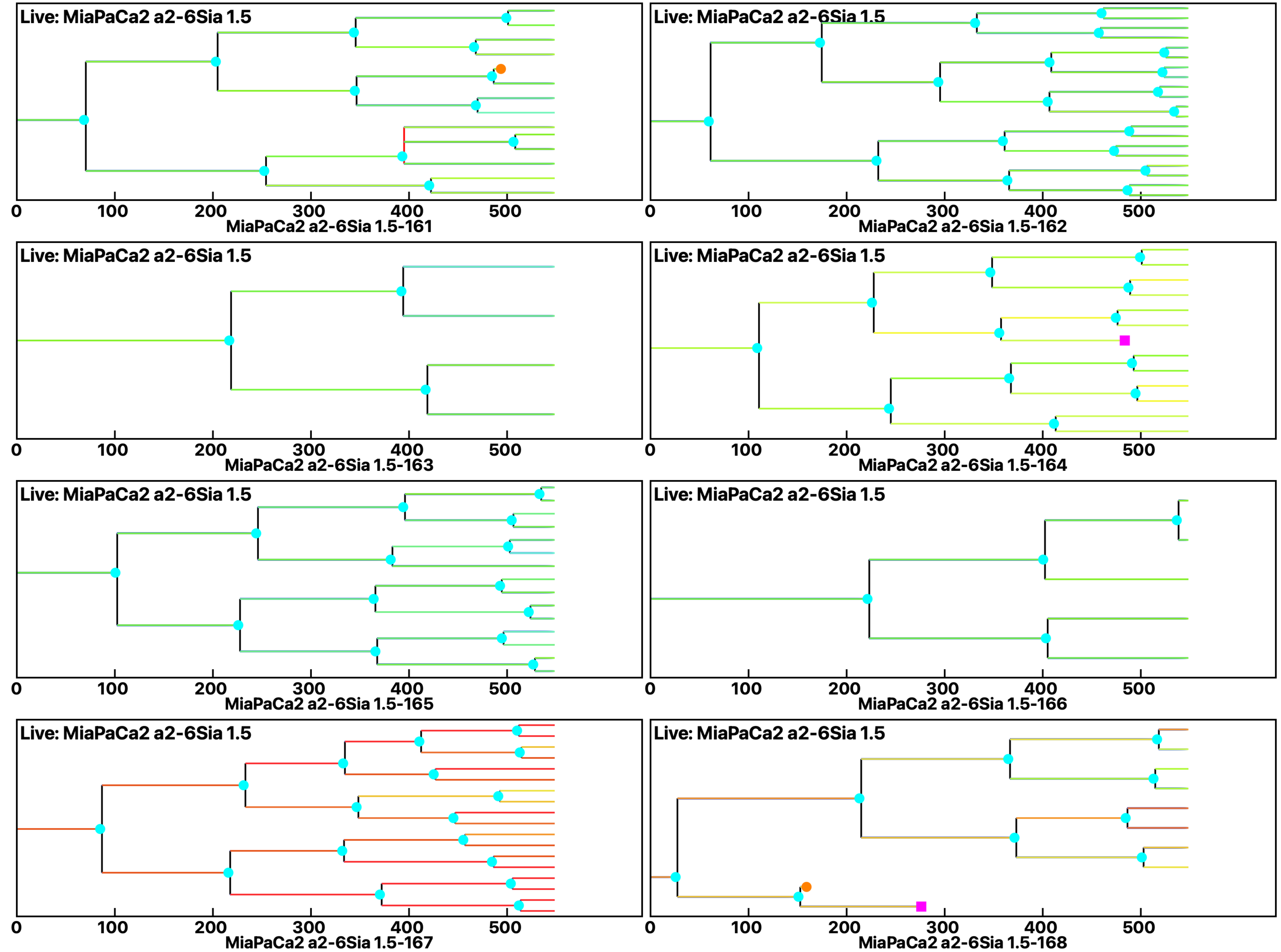

Analysis: Simulation, Treat.: MiaPaCa2 a2-6Sia 1.5, Cell: MiaPaCa2-Simulation

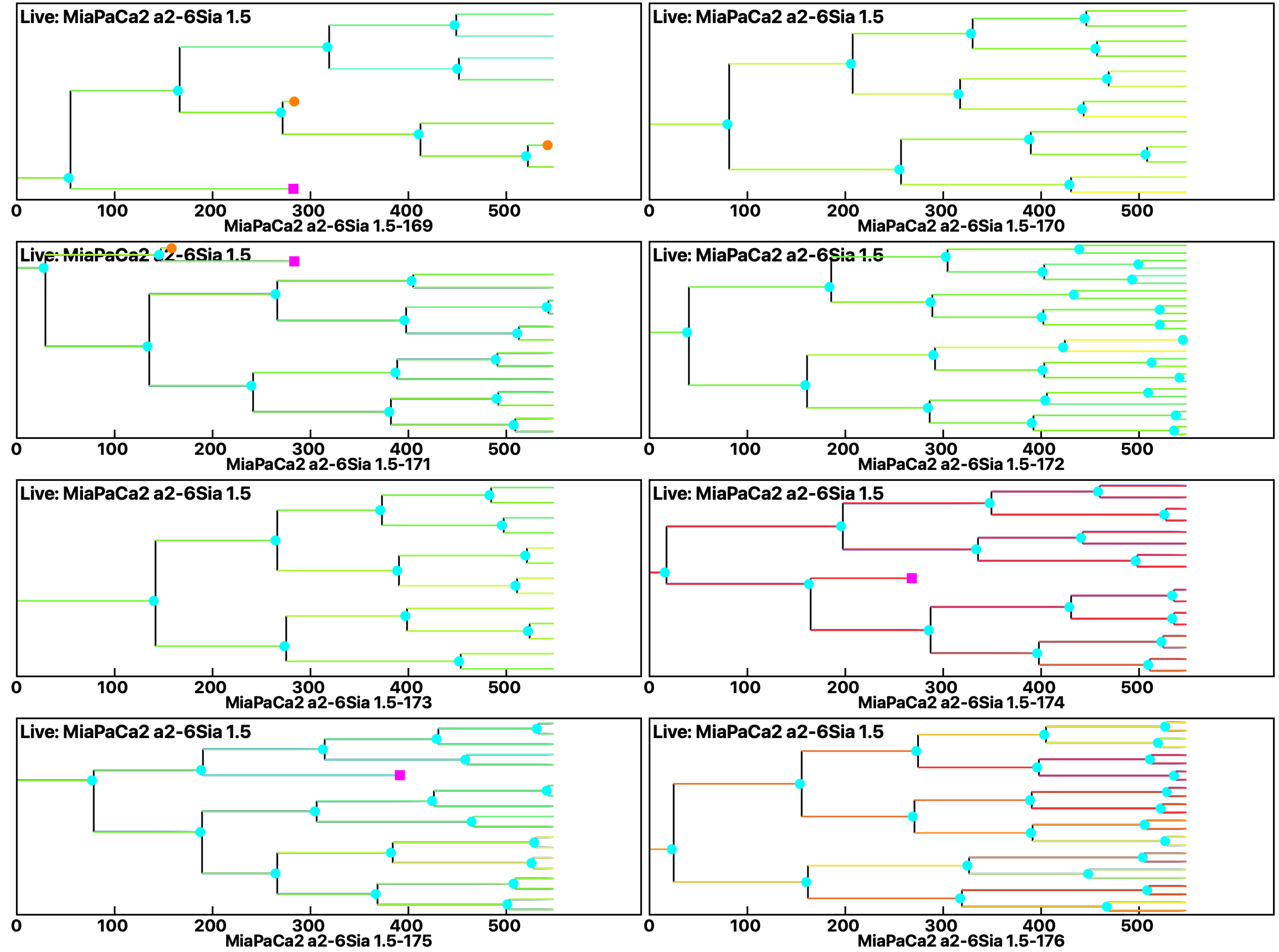

Analysis: Simulation, Treat.: MiaPaCa2 a2-6Sia 1.5, Cell: MiaPaCa2-Simulation

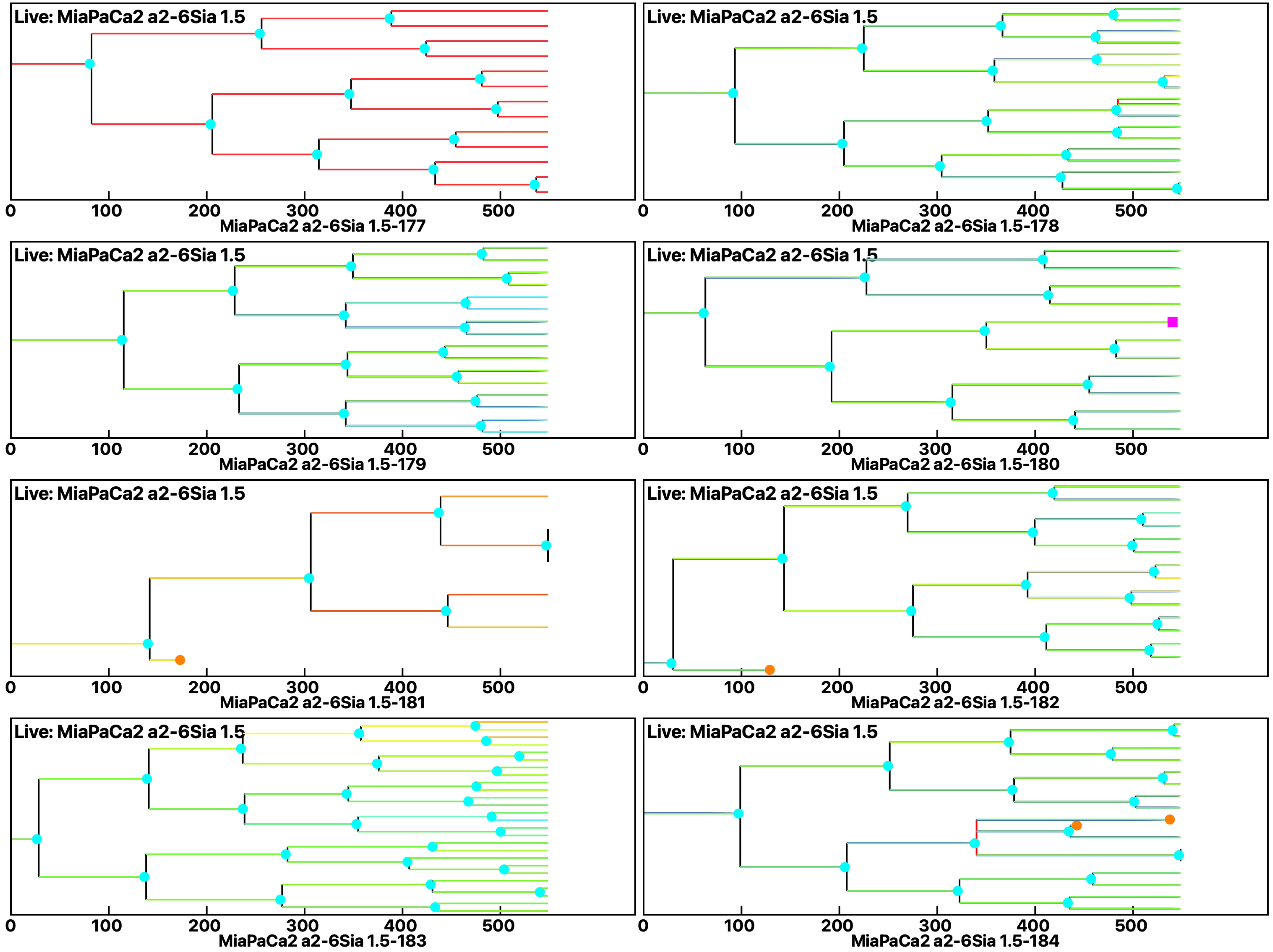

Analysis: Simulation, Treat.: MiaPaCa2 a2-6Sia 1.5, Cell: MiaPaCa2-Simulation

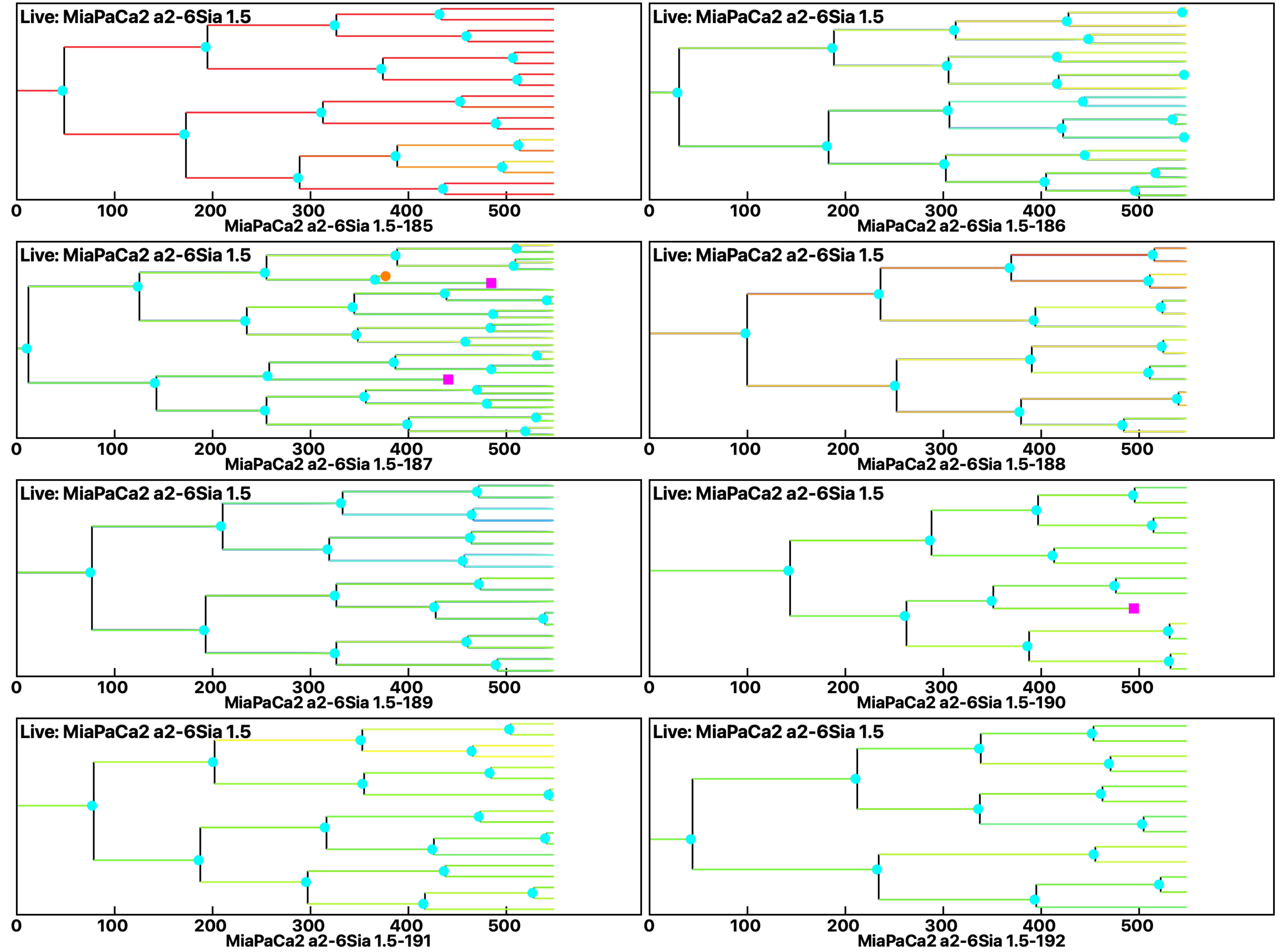

Analysis: Simulation, Treat.: MiaPaCa2 a2-6Sia 1.5, Cell: MiaPaCa2-Simulation

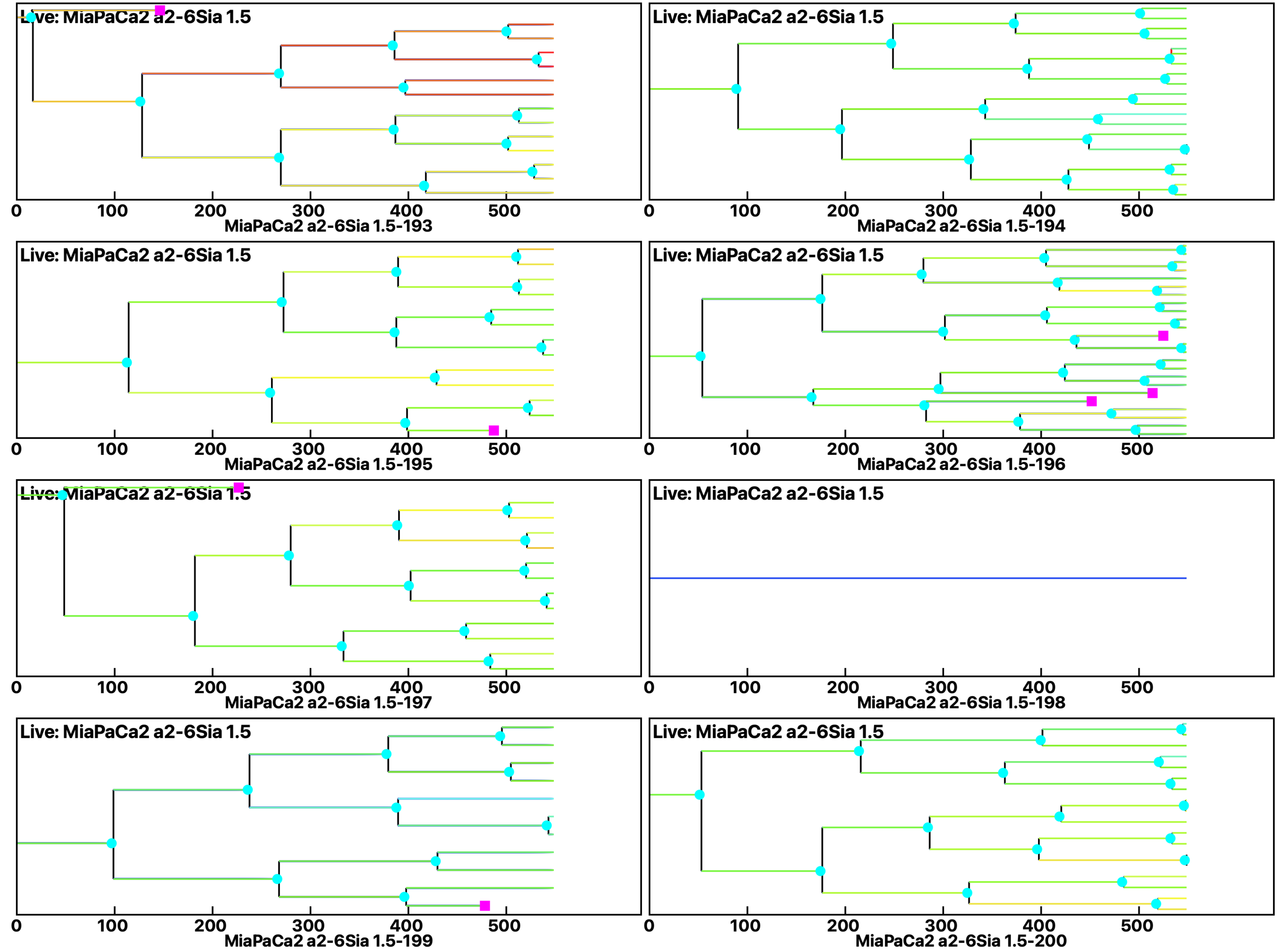

Analysis: Simulation, Treat.: MiaPaCa2 a2-6Sia 1.5, Cell: MiaPaCa2-Simulation

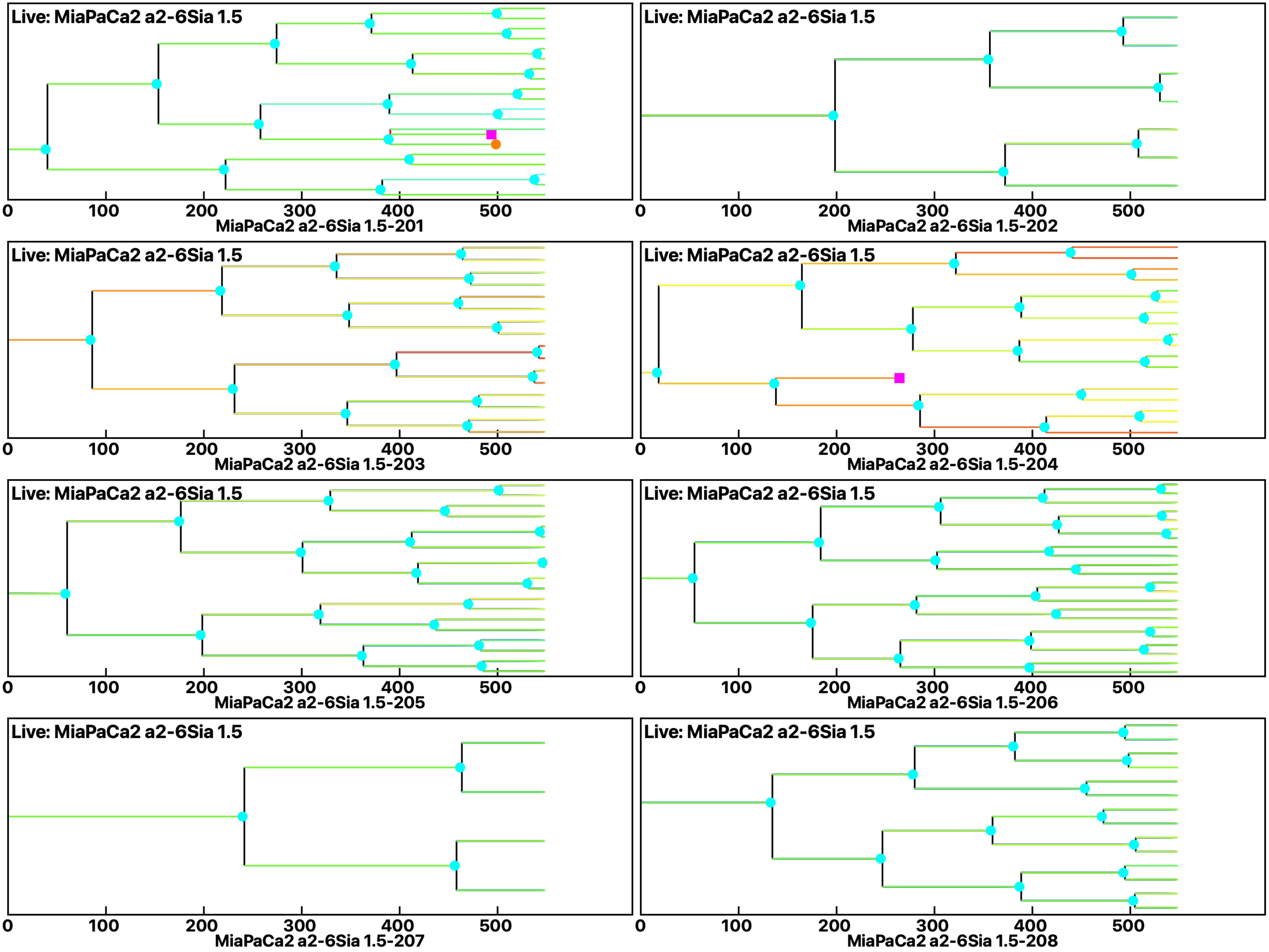

Analysis: Simulation, Treat.: MiaPaCa2 a2-6Sia 1.5, Cell: MiaPaCa2-Simulation

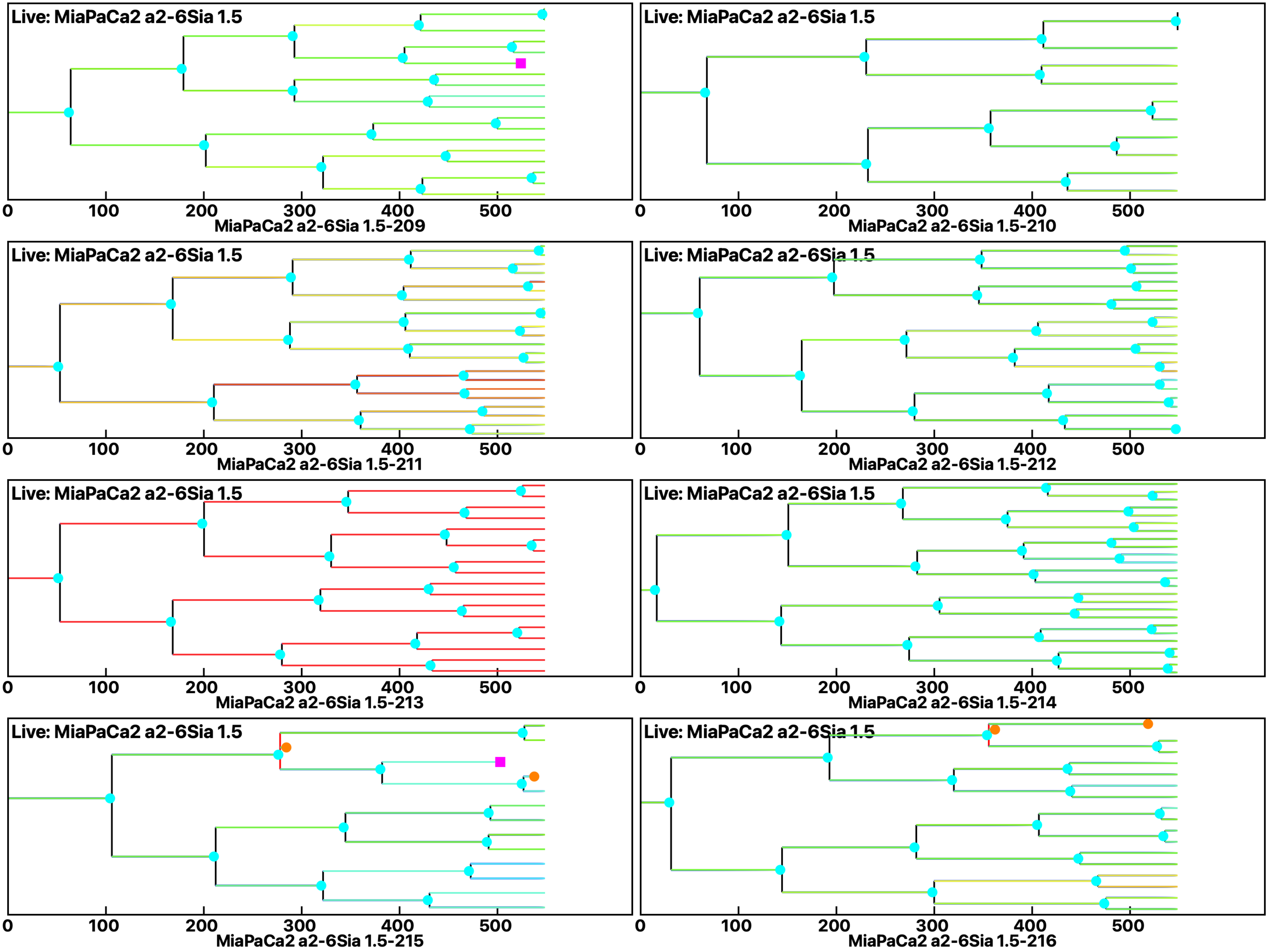

Analysis: Simulation, Treat.: MiaPaCa2 a2-6Sia 1.5, Cell: MiaPaCa2-Simulation

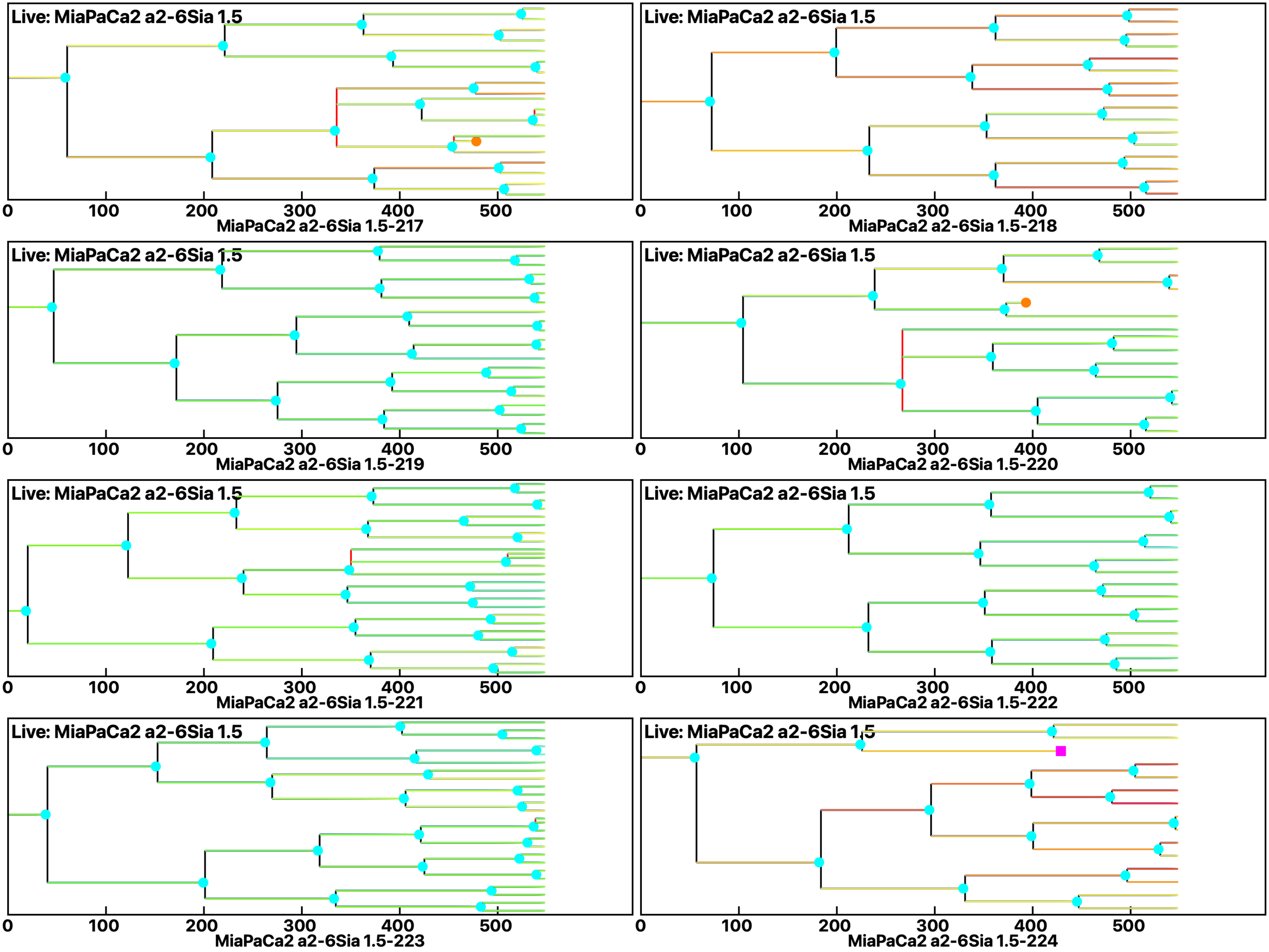

Analysis: Simulation, Treat.: MiaPaCa2 a2-6Sia 1.5, Cell: MiaPaCa2-Simulation

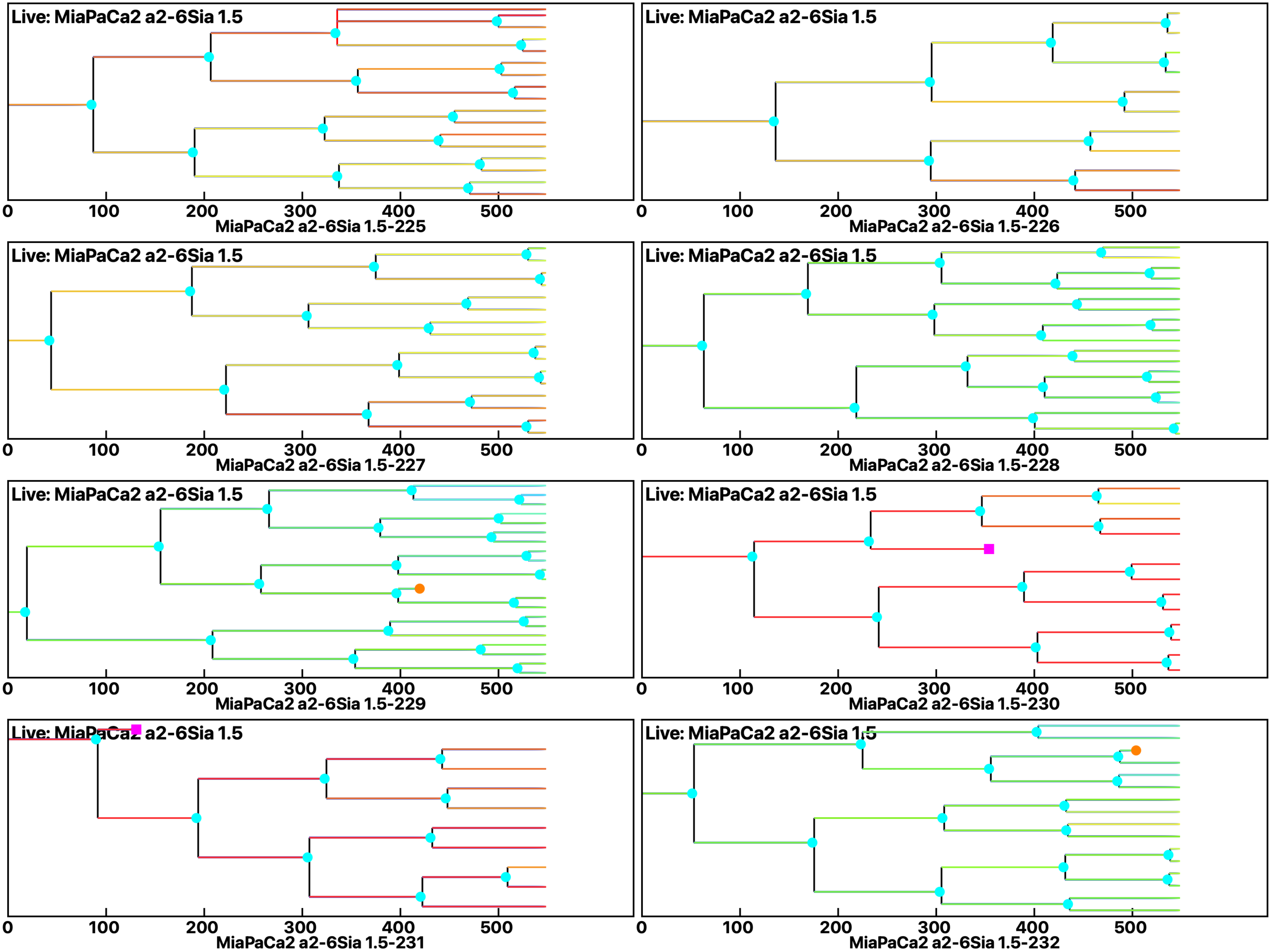

Analysis: Simulation, Treat.: MiaPaCa2 a2-6Sia 1.5, Cell: MiaPaCa2-Simulation

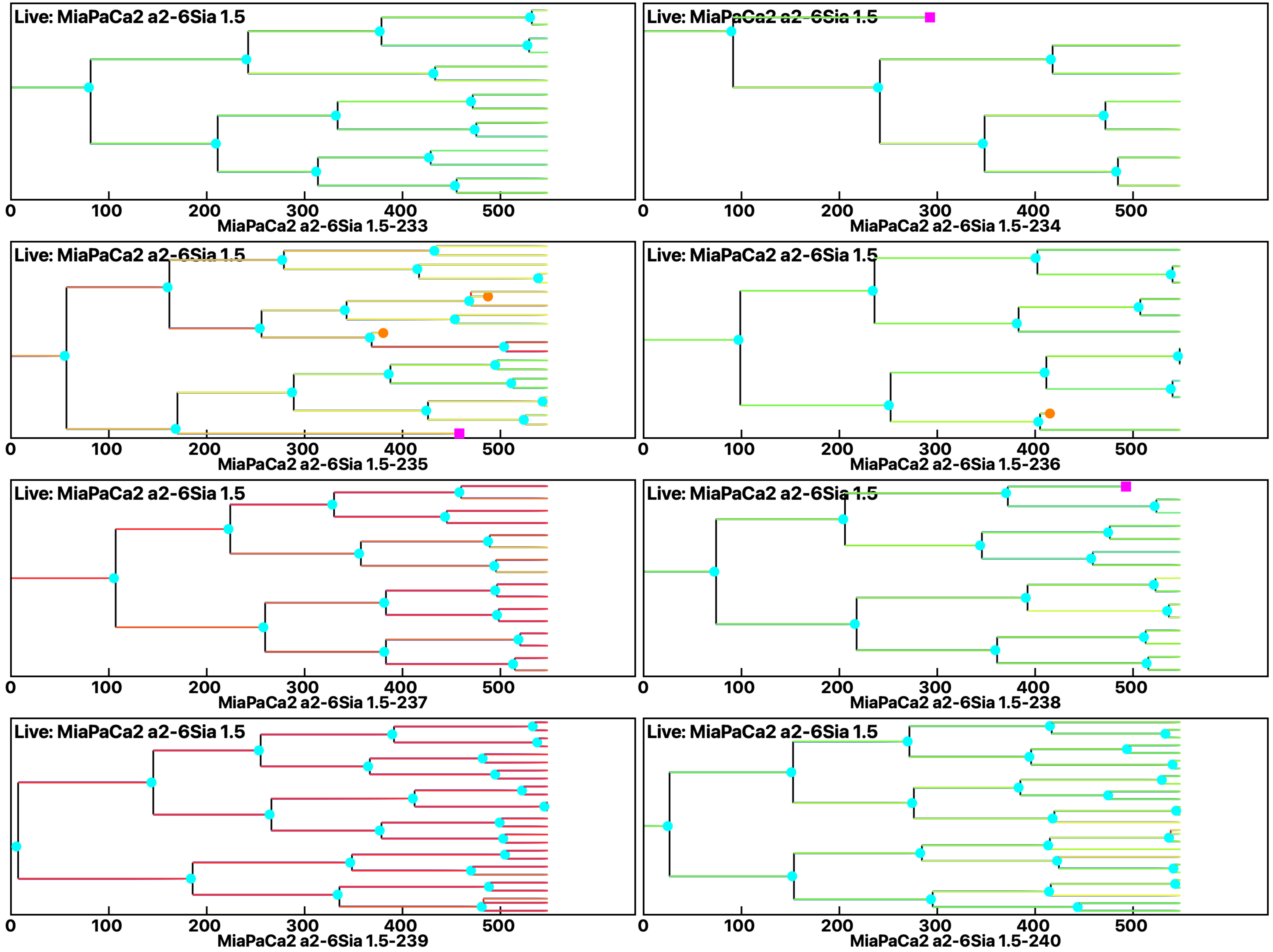

Analysis: Simulation, Treat.: MiaPaCa2 a2-6Sia 1.5, Cell: MiaPaCa2-Simulation

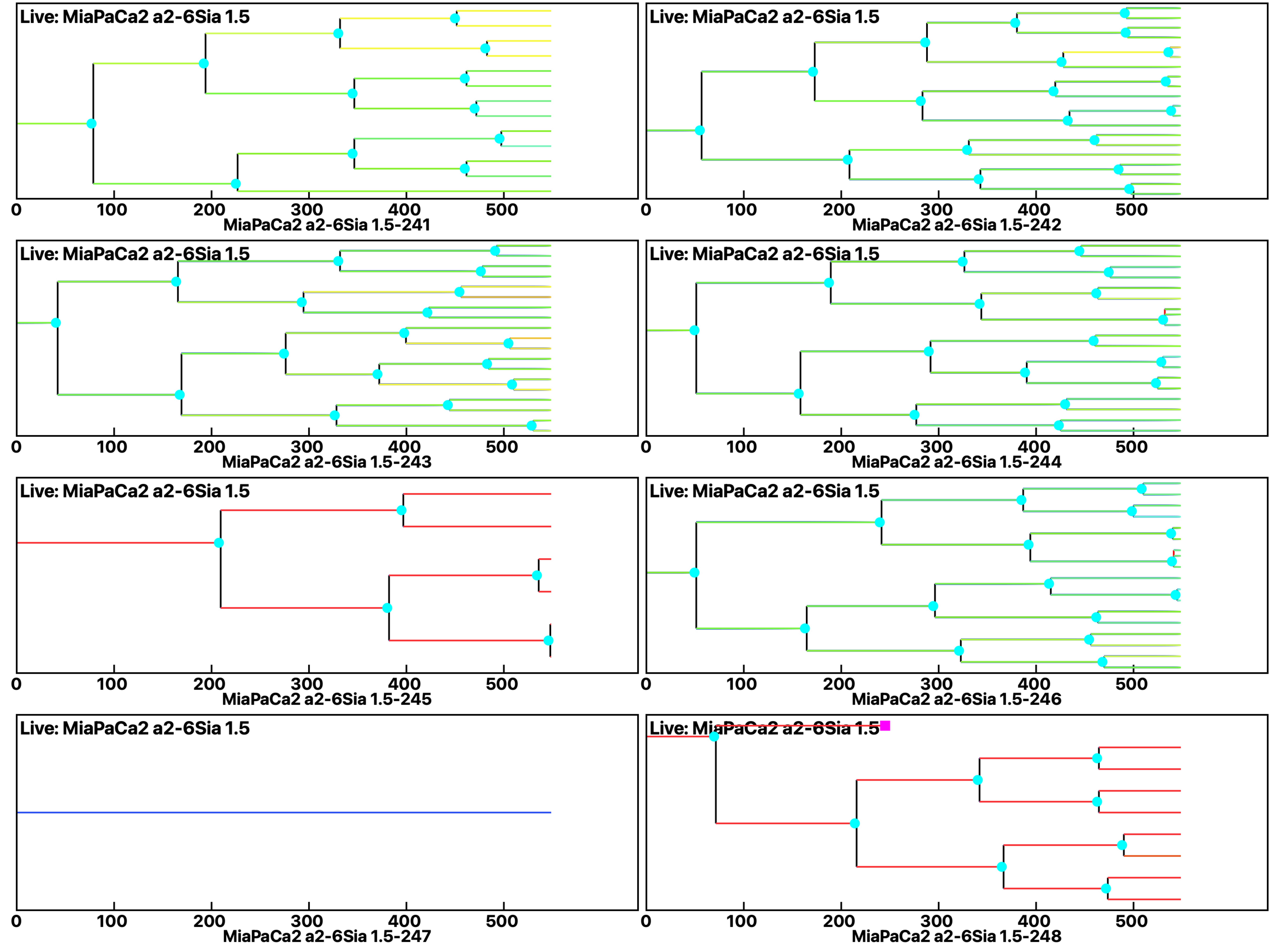

Analysis: Simulation, Treat.: MiaPaCa2 a2-6Sia 1.5, Cell: MiaPaCa2-Simulation

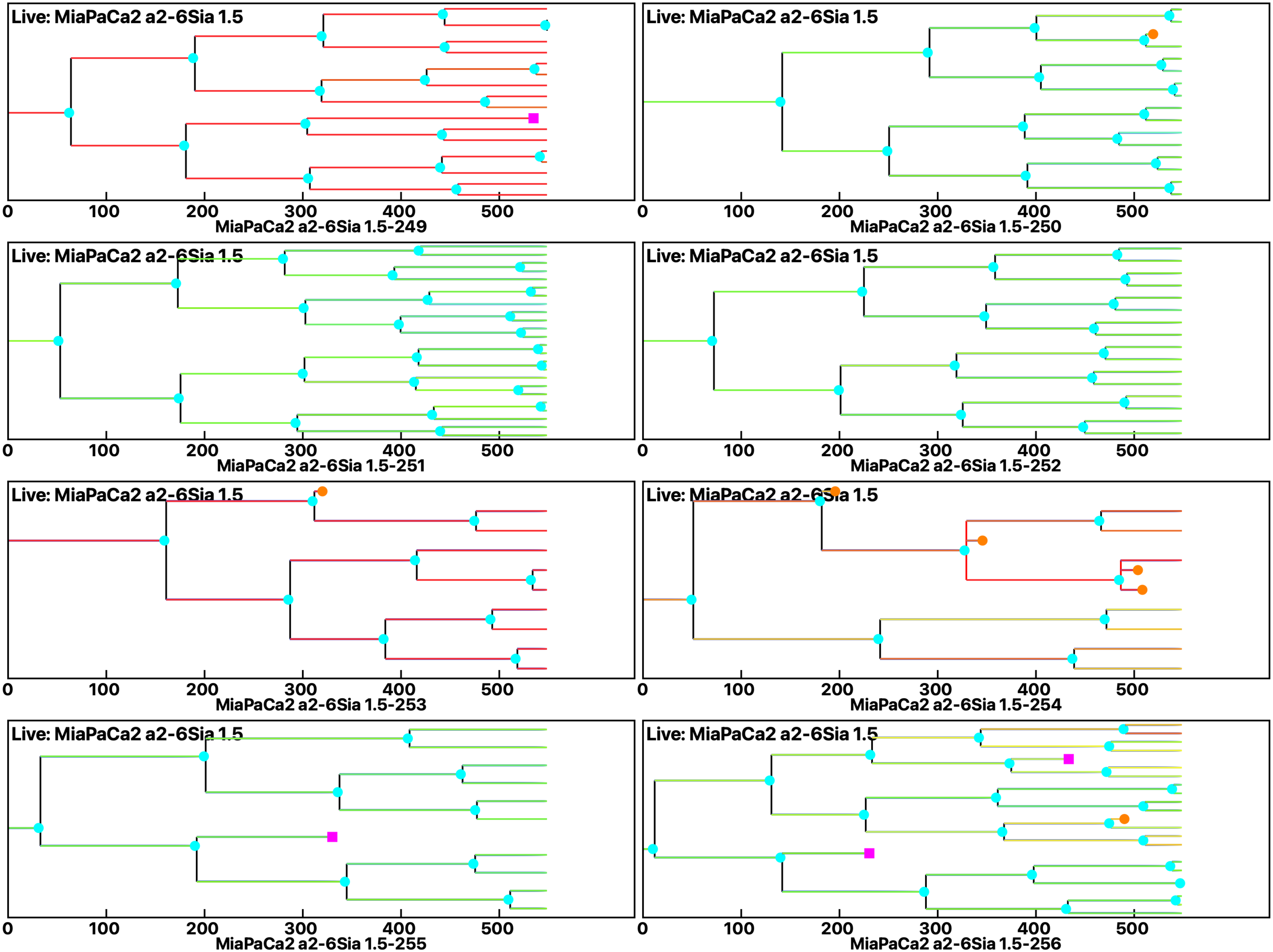

Analysis: Simulation, Treat.: MiaPaCa2 a2-6Sia 1.5, Cell: MiaPaCa2-Simulation

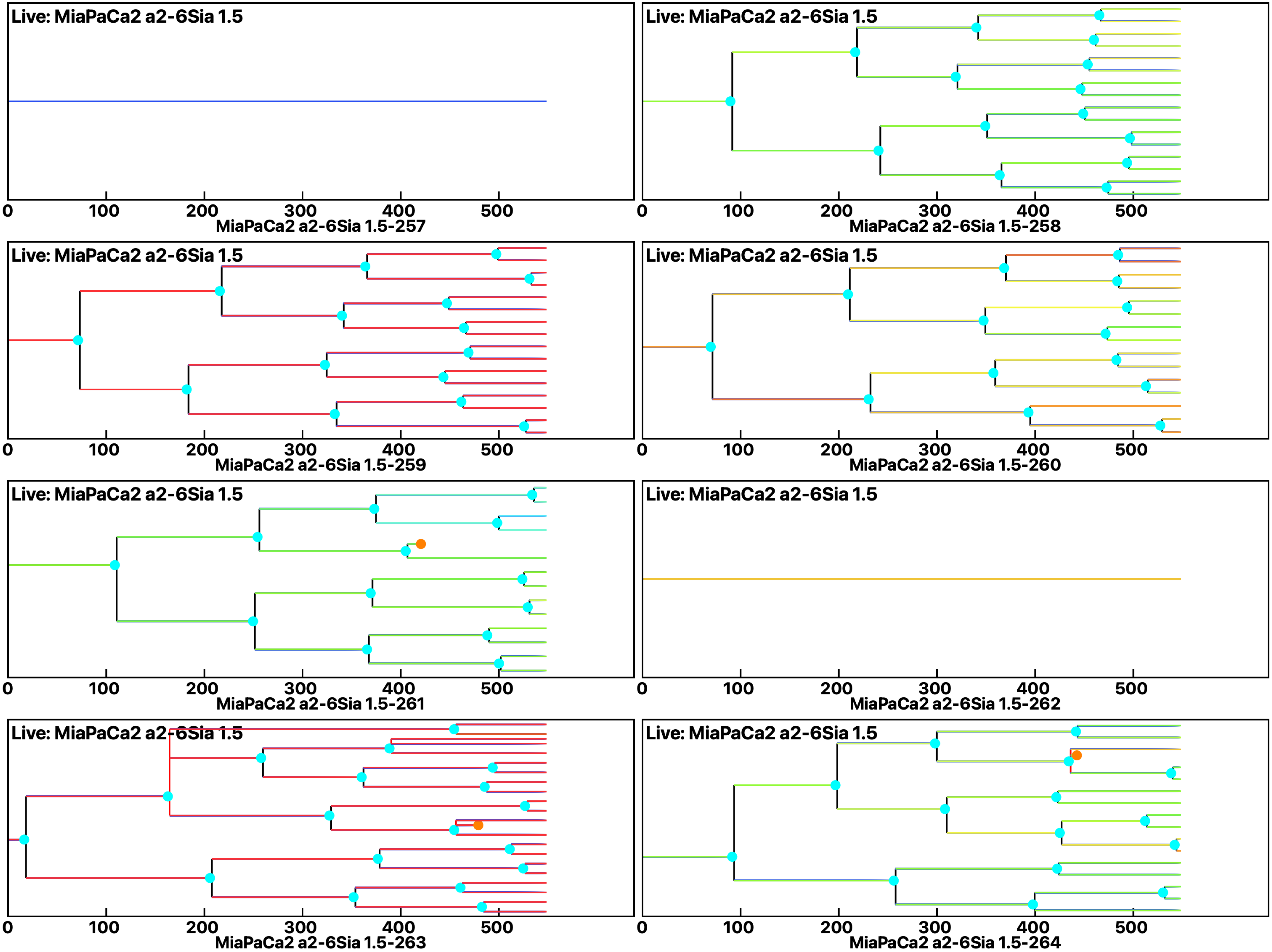

Analysis: Simulation, Treat.: MiaPaCa2 a2-6Sia 1.5, Cell: MiaPaCa2-Simulation

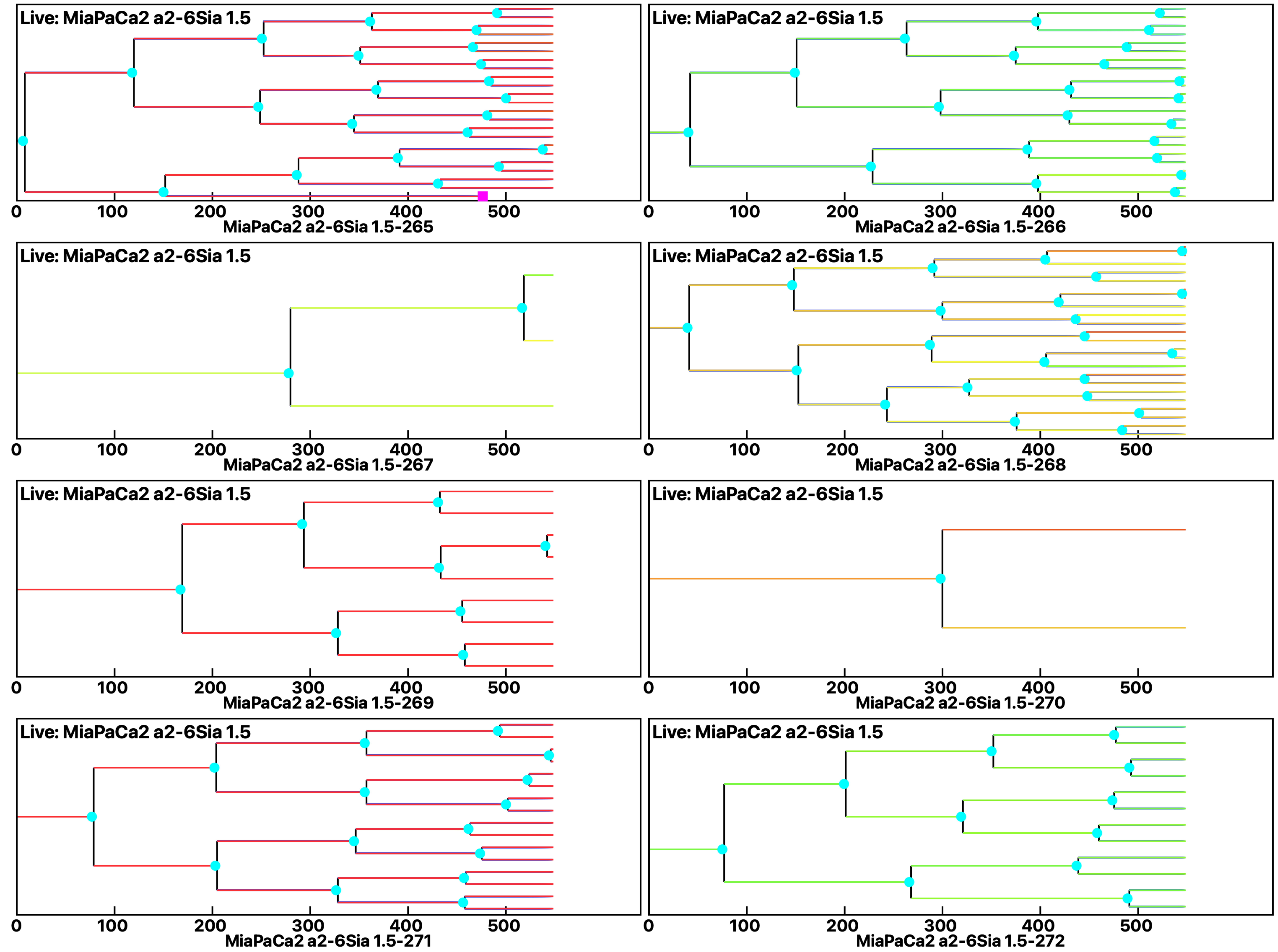

Analysis: Simulation, Treat.: MiaPaCa2 a2-6Sia 1.5, Cell: MiaPaCa2-Simulation

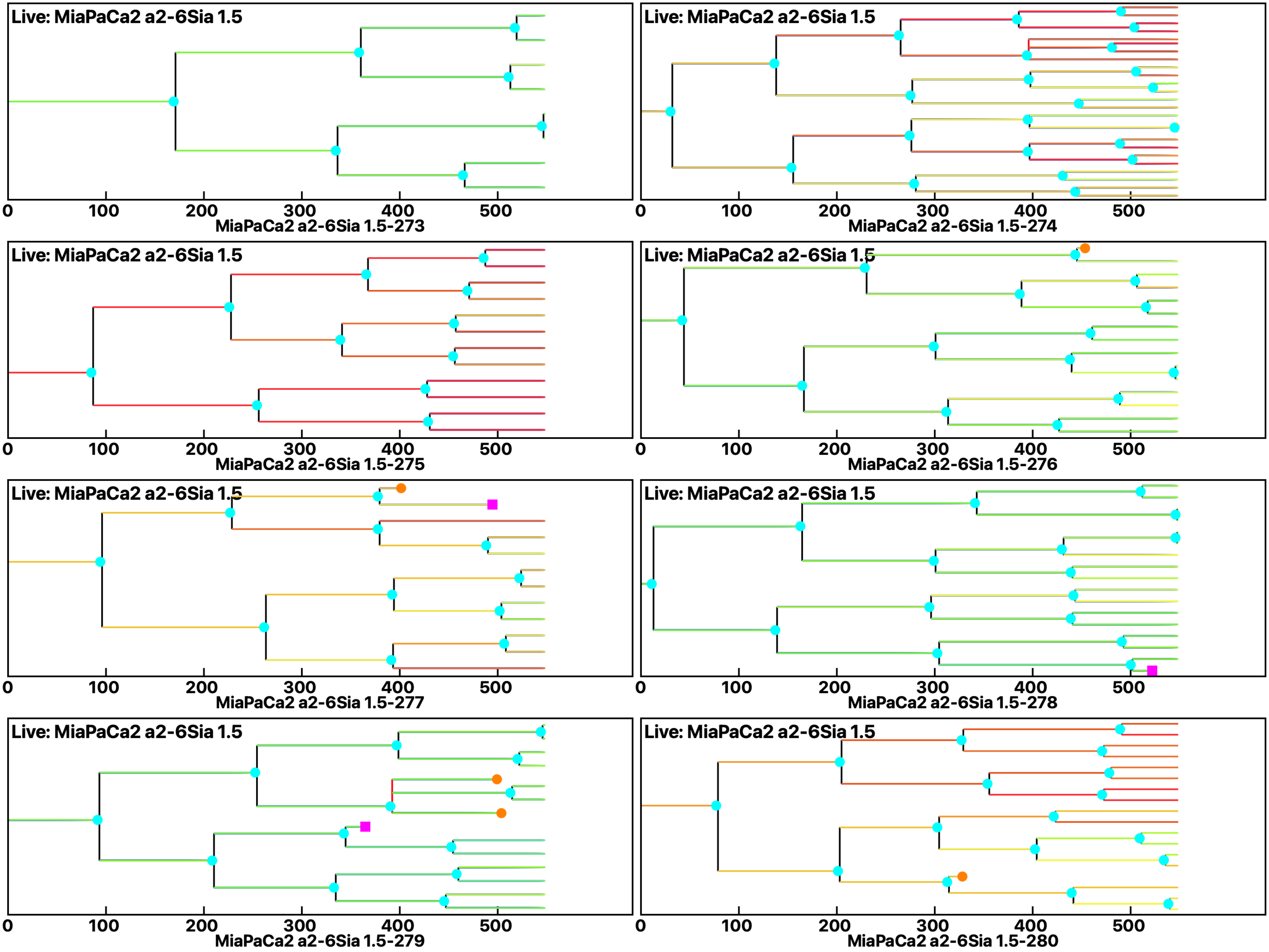

Analysis: Simulation, Treat.: MiaPaCa2 a2-6Sia 1.5, Cell: MiaPaCa2-Simulation

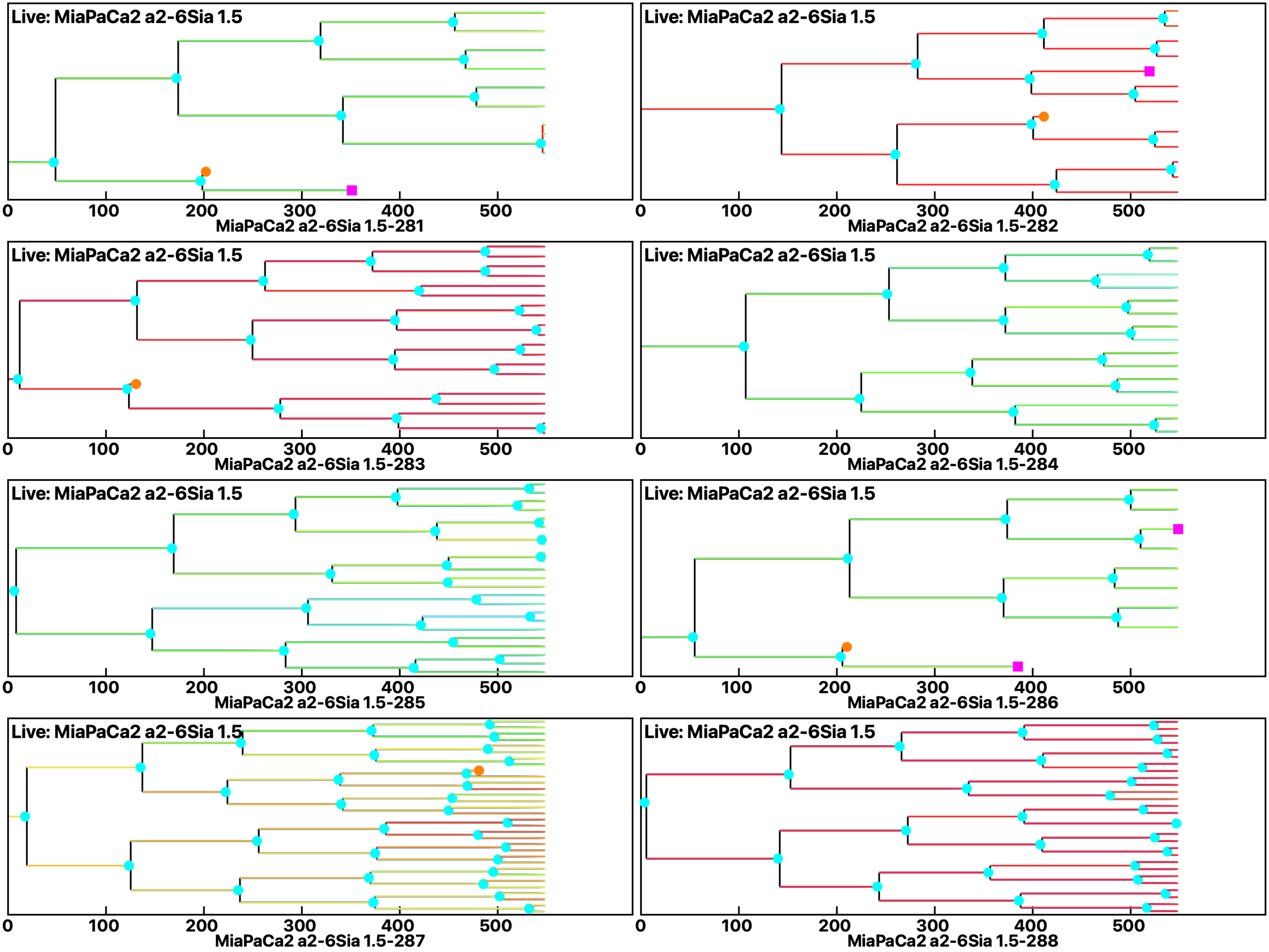

Analysis: Simulation, Treat.: MiaPaCa2 a2-6Sia 1.5, Cell: MiaPaCa2-Simulation

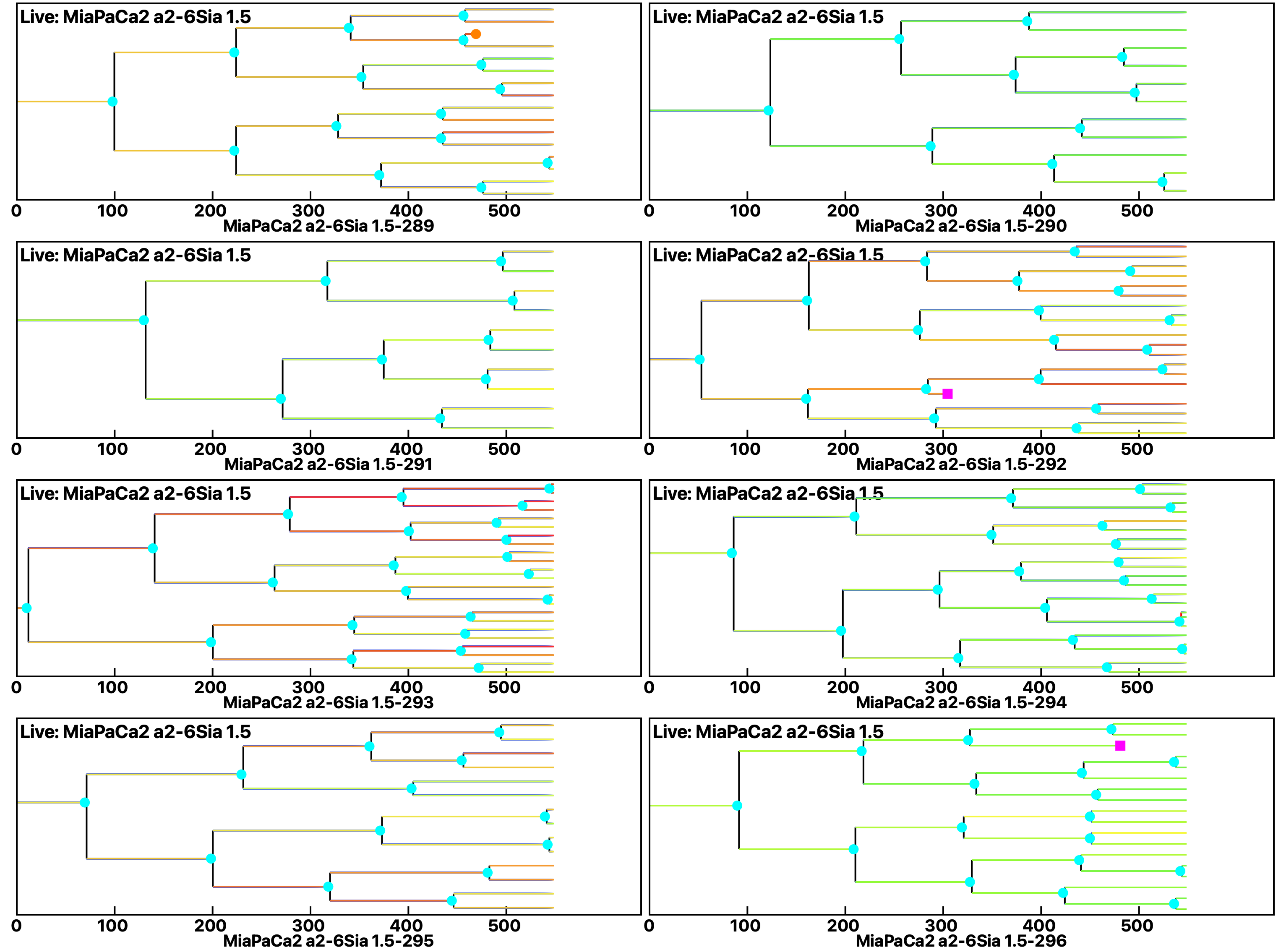

Analysis: Simulation, Treat.: MiaPaCa2 a2-6Sia 1.5, Cell: MiaPaCa2-Simulation

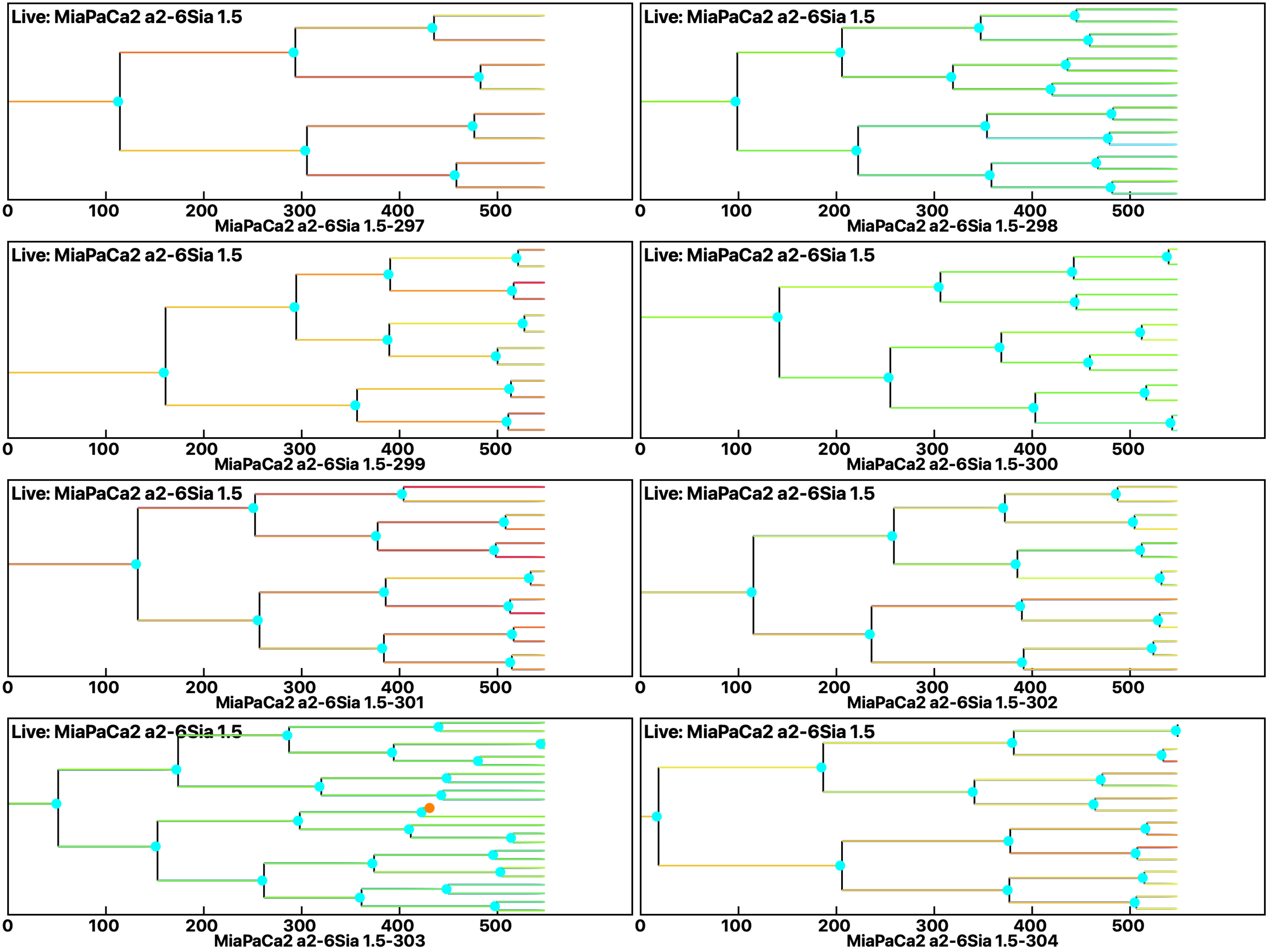

Analysis: Simulation, Treat.: MiaPaCa2 a2-6Sia 1.5, Cell: MiaPaCa2-Simulation

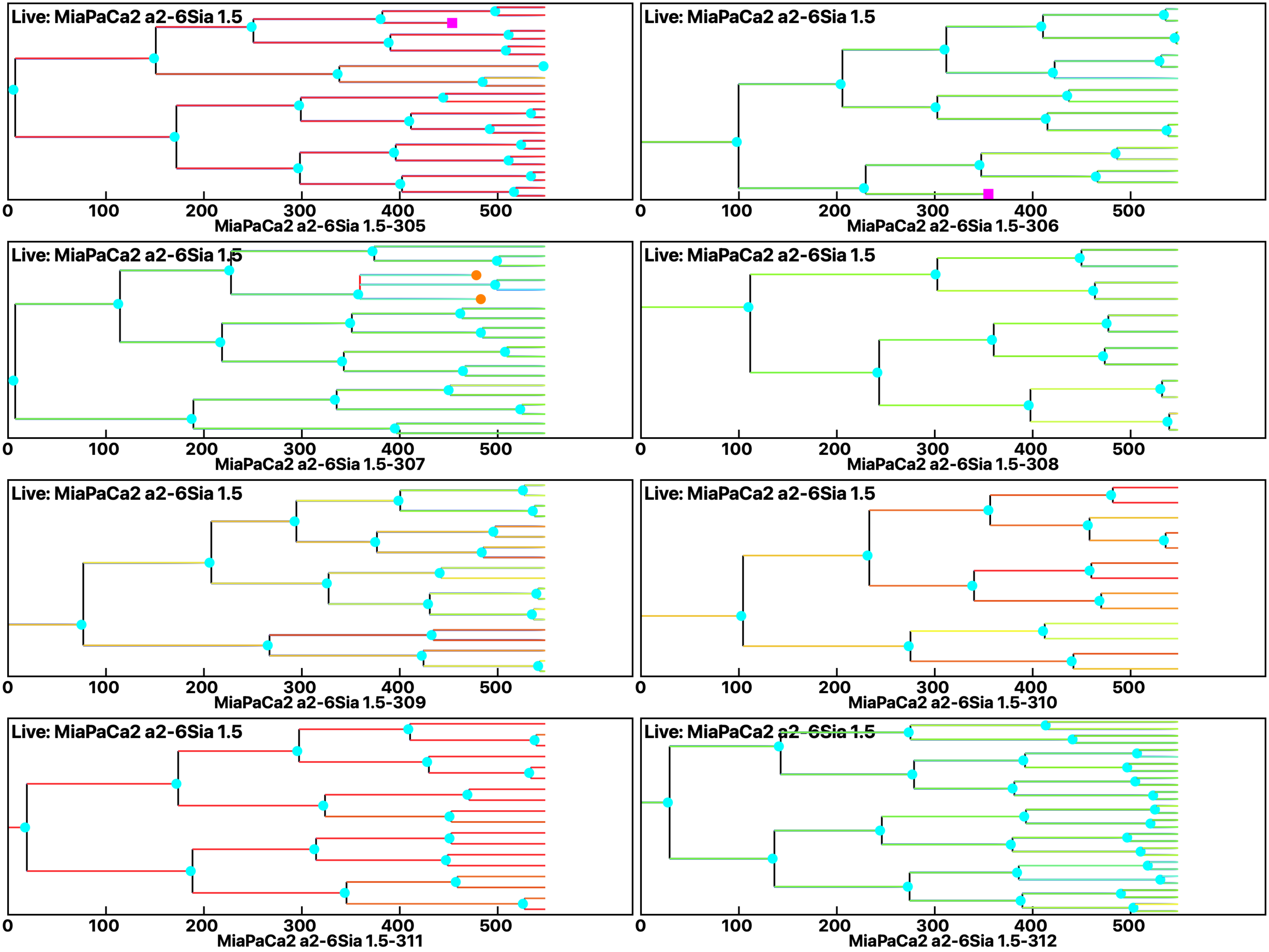

Analysis: Simulation, Treat.: MiaPaCa2 a2-6Sia 1.5, Cell: MiaPaCa2-Simulation

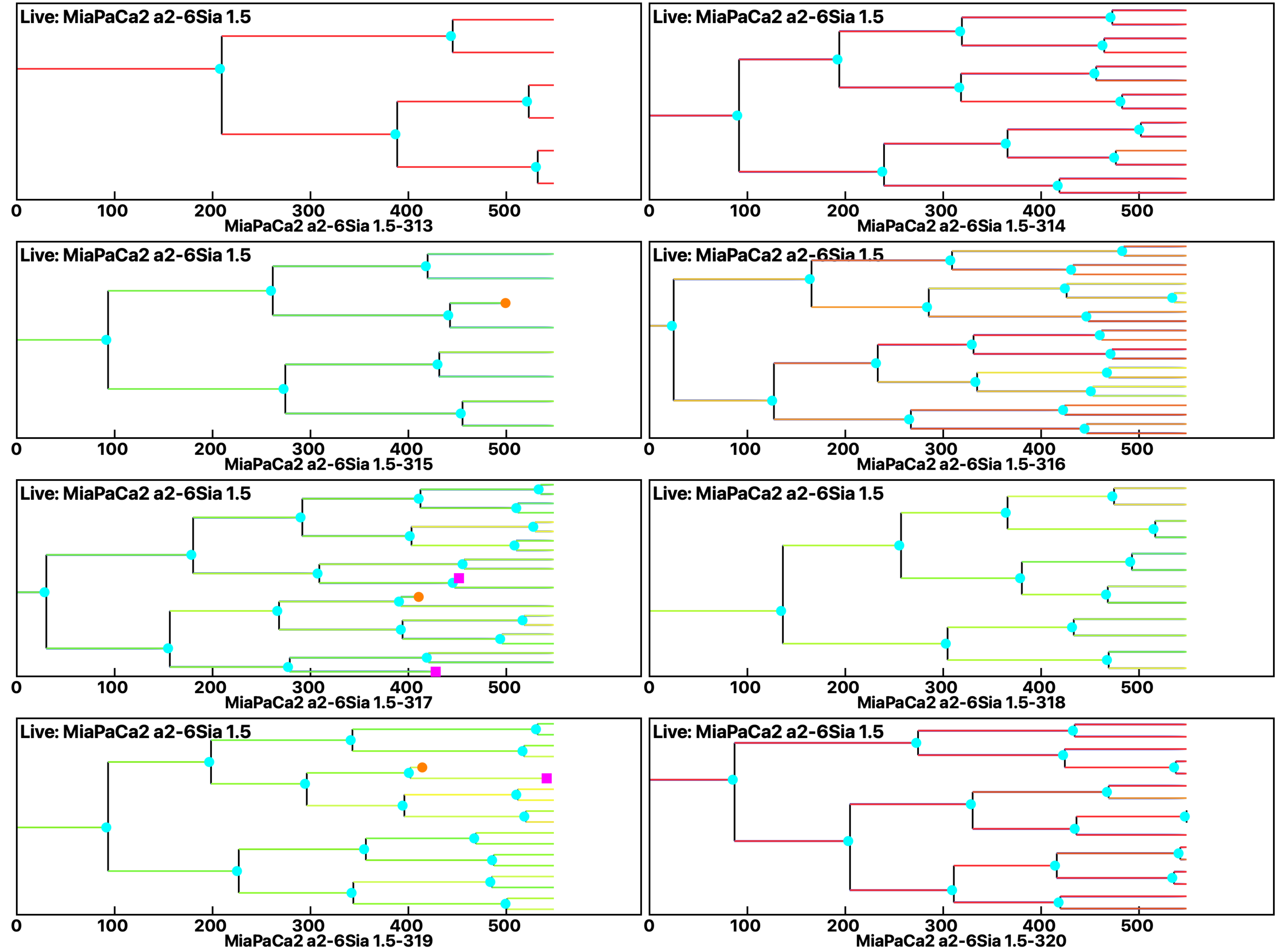

Analysis: Simulation, Treat.: MiaPaCa2 a2-6Sia 1.5, Cell: MiaPaCa2-Simulation

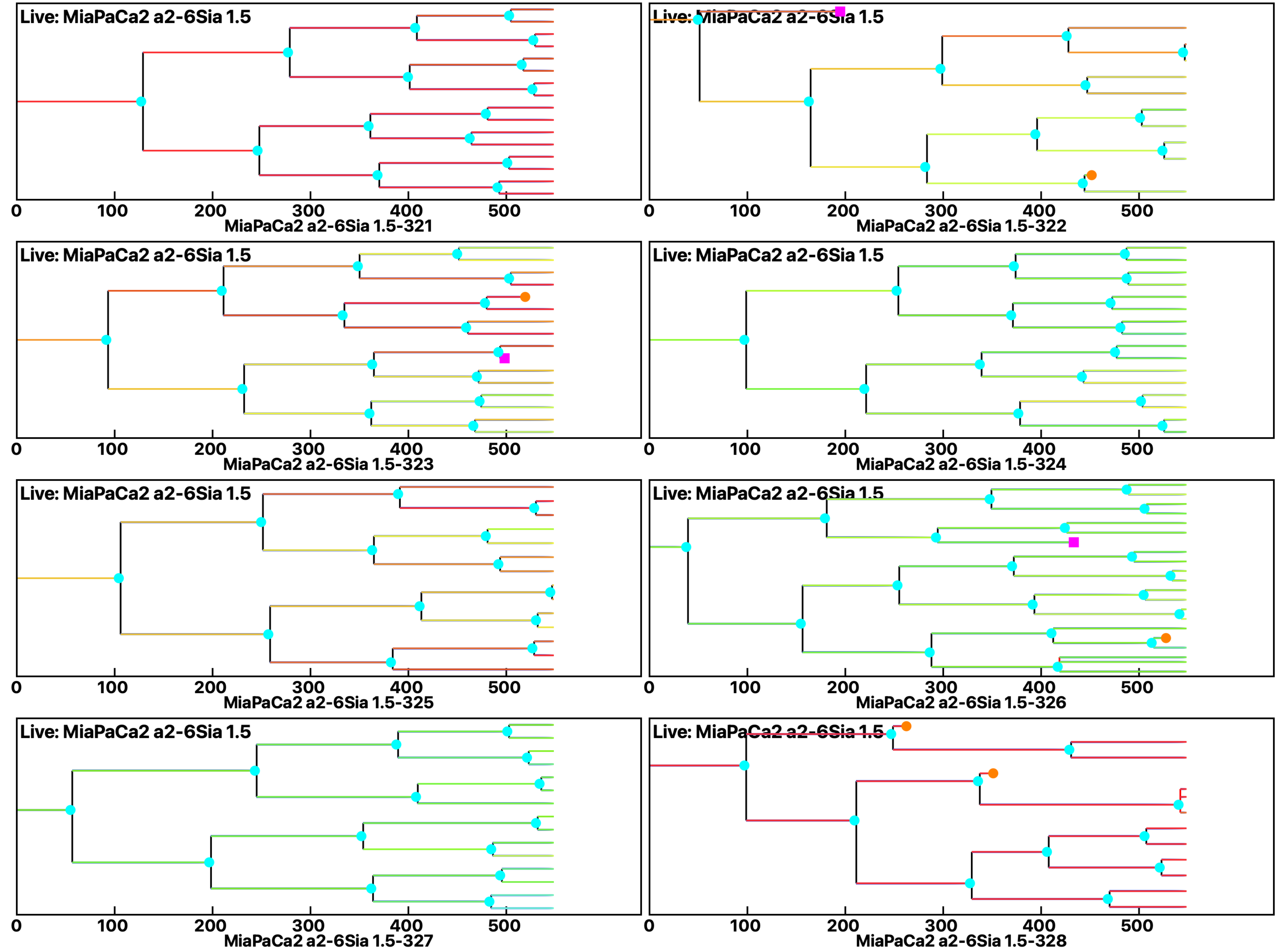

Analysis: Simulation, Treat.: MiaPaCa2 a2-6Sia 1.5, Cell: MiaPaCa2-Simulation

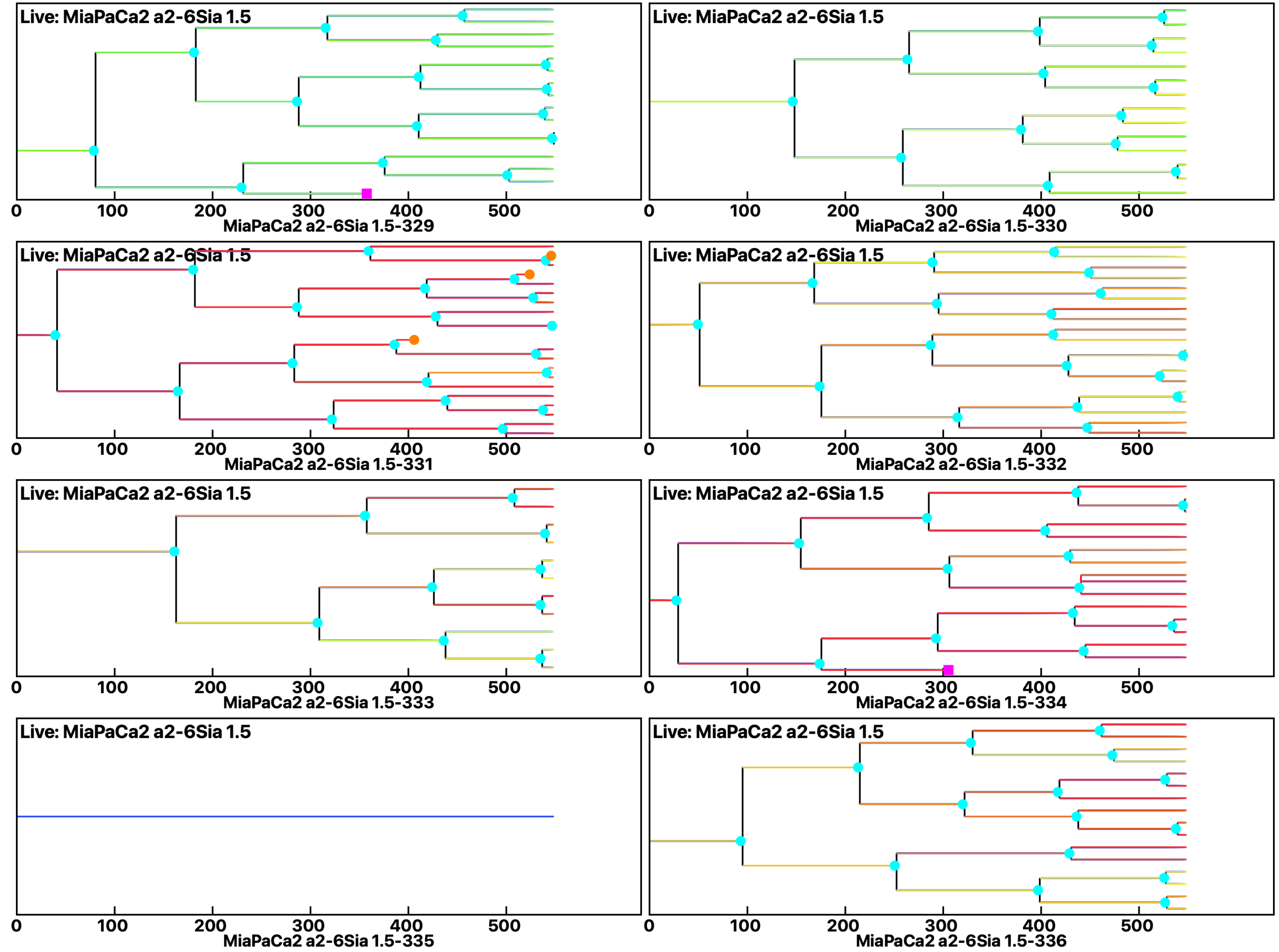

Analysis: Simulation, Treat.: MiaPaCa2 a2-6Sia 1.5, Cell: MiaPaCa2-Simulation

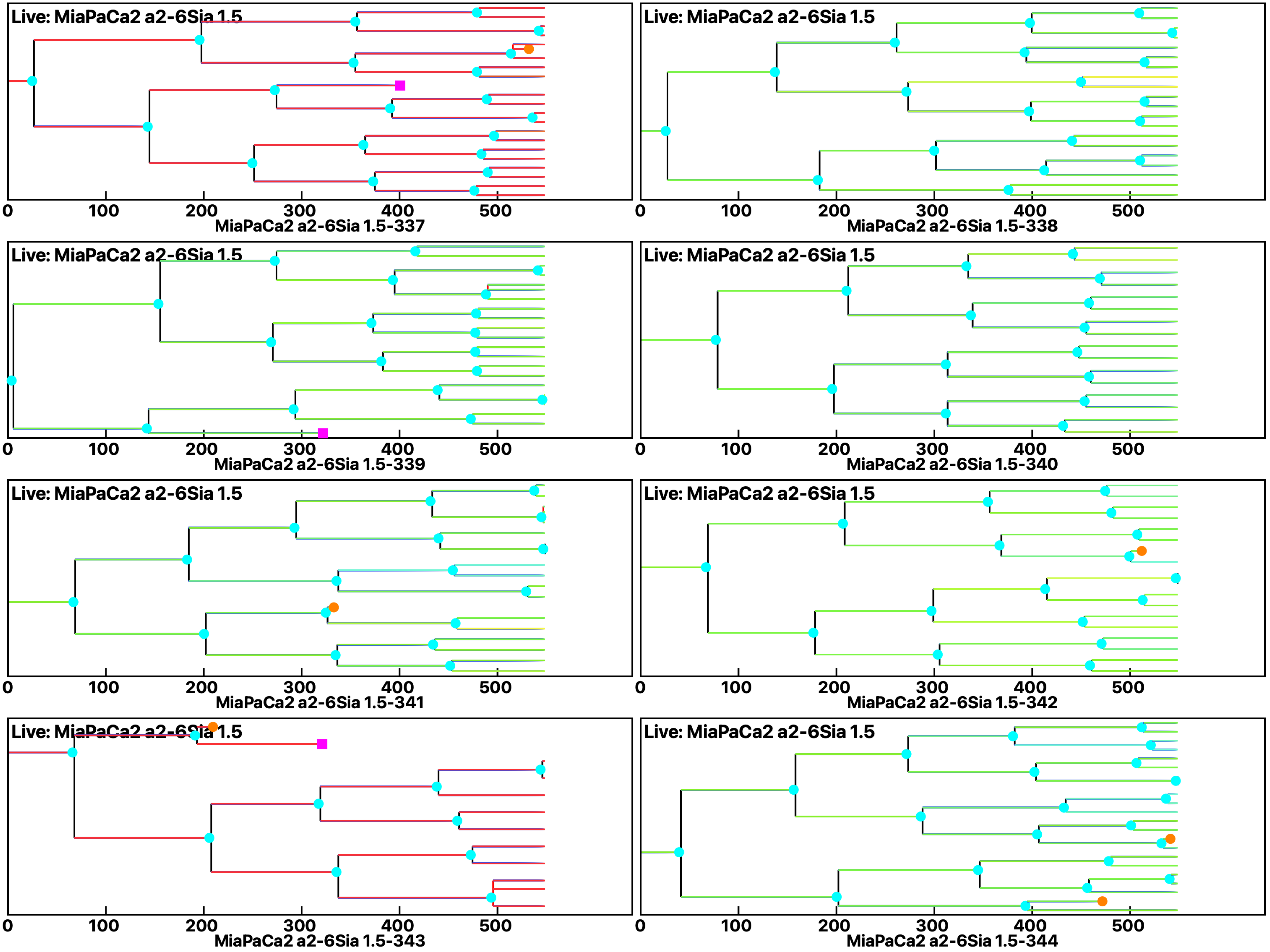

Analysis: Simulation, Treat.: MiaPaCa2 a2-6Sia 1.5, Cell: MiaPaCa2-Simulation

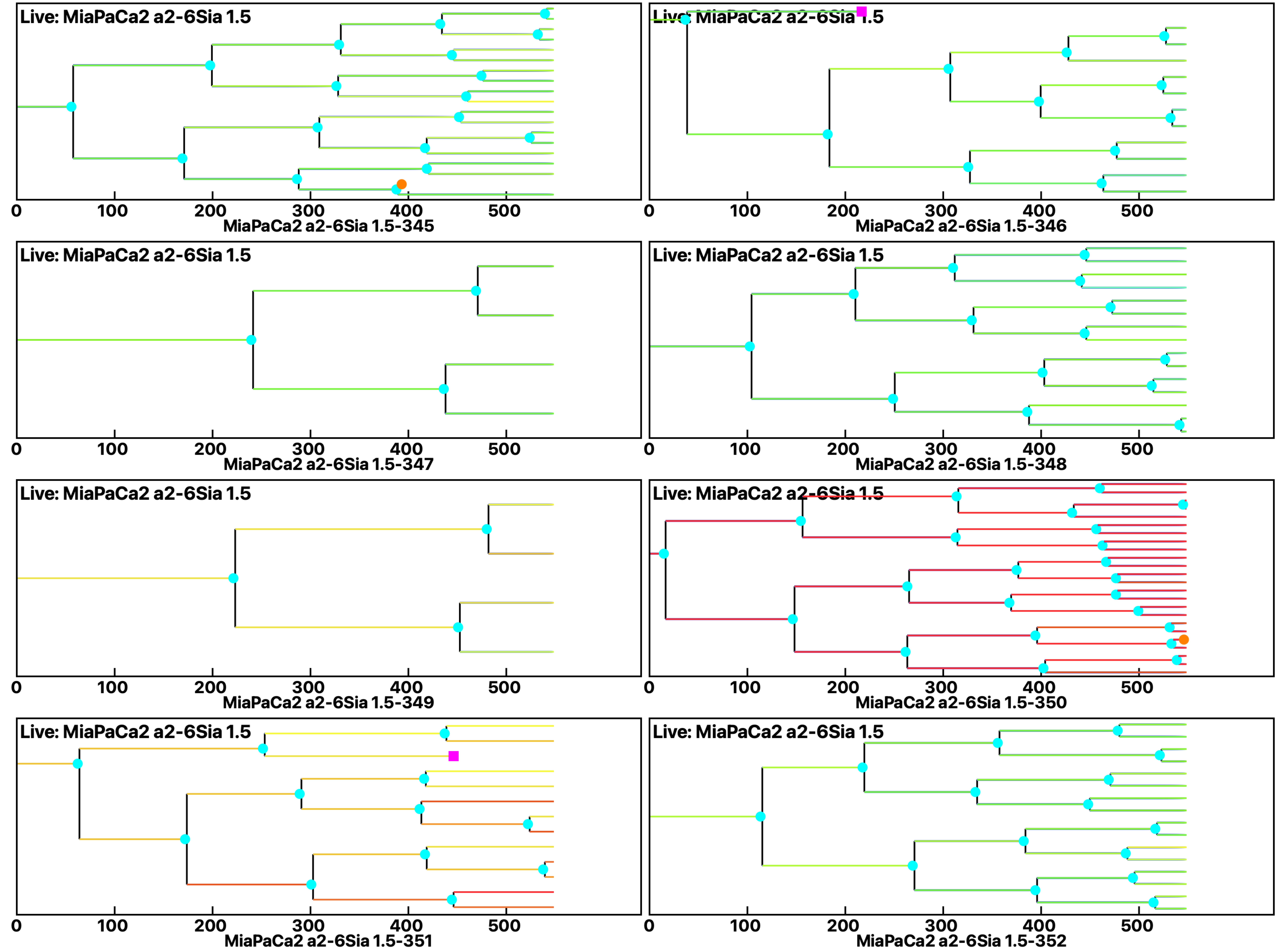

Analysis: Simulation, Treat.: MiaPaCa2 a2-6Sia 1.5, Cell: MiaPaCa2-Simulation

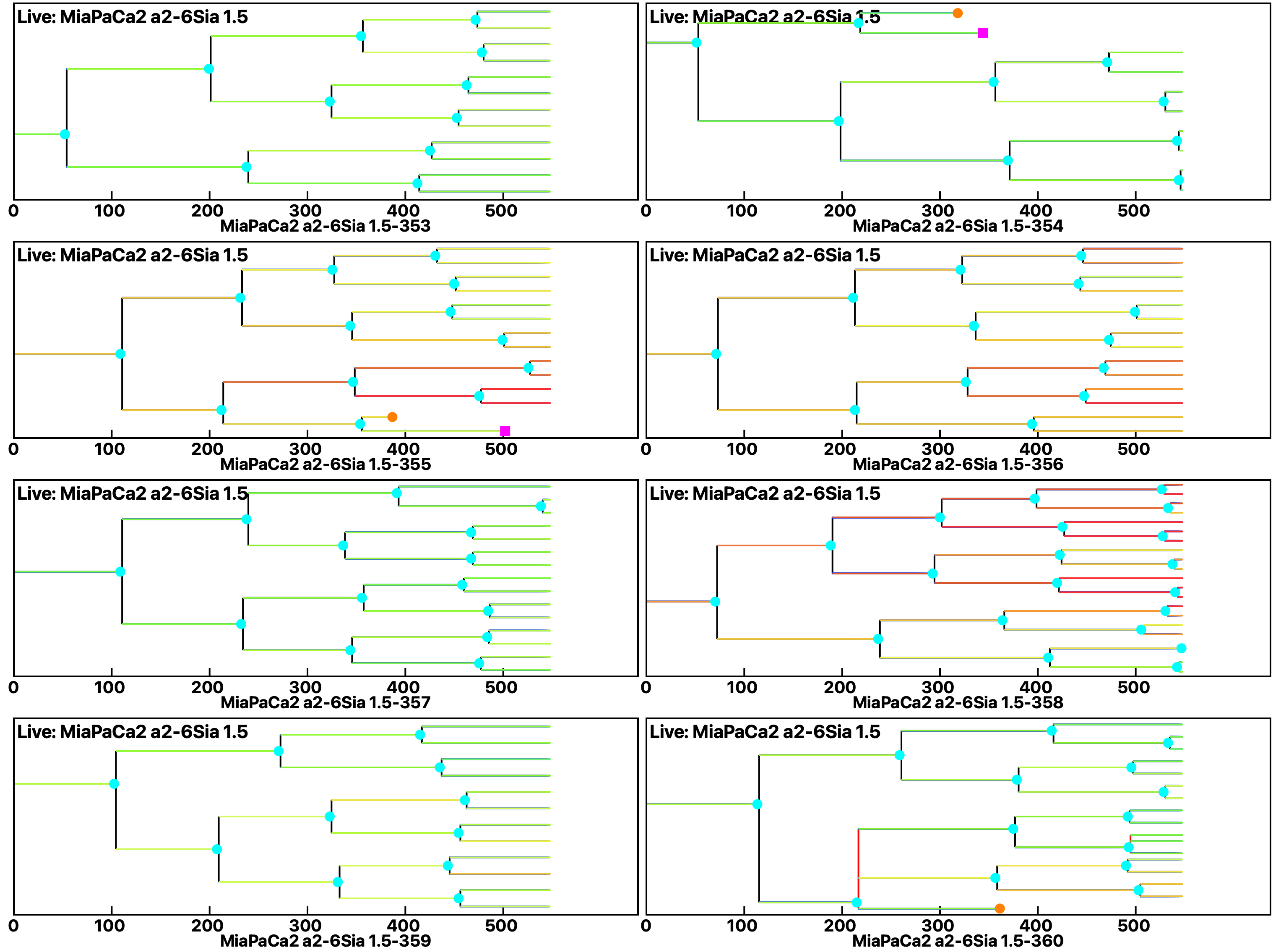

Analysis: Simulation, Treat.: MiaPaCa2 a2-6Sia 1.5, Cell: MiaPaCa2-Simulation

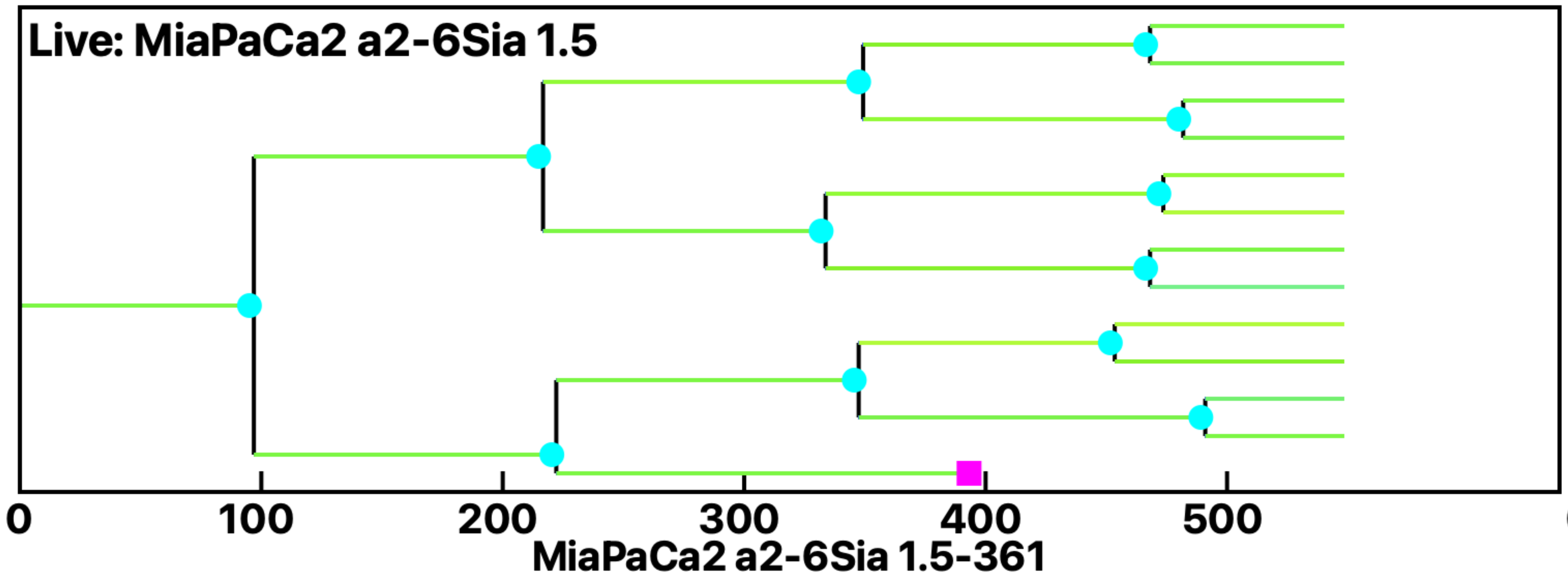

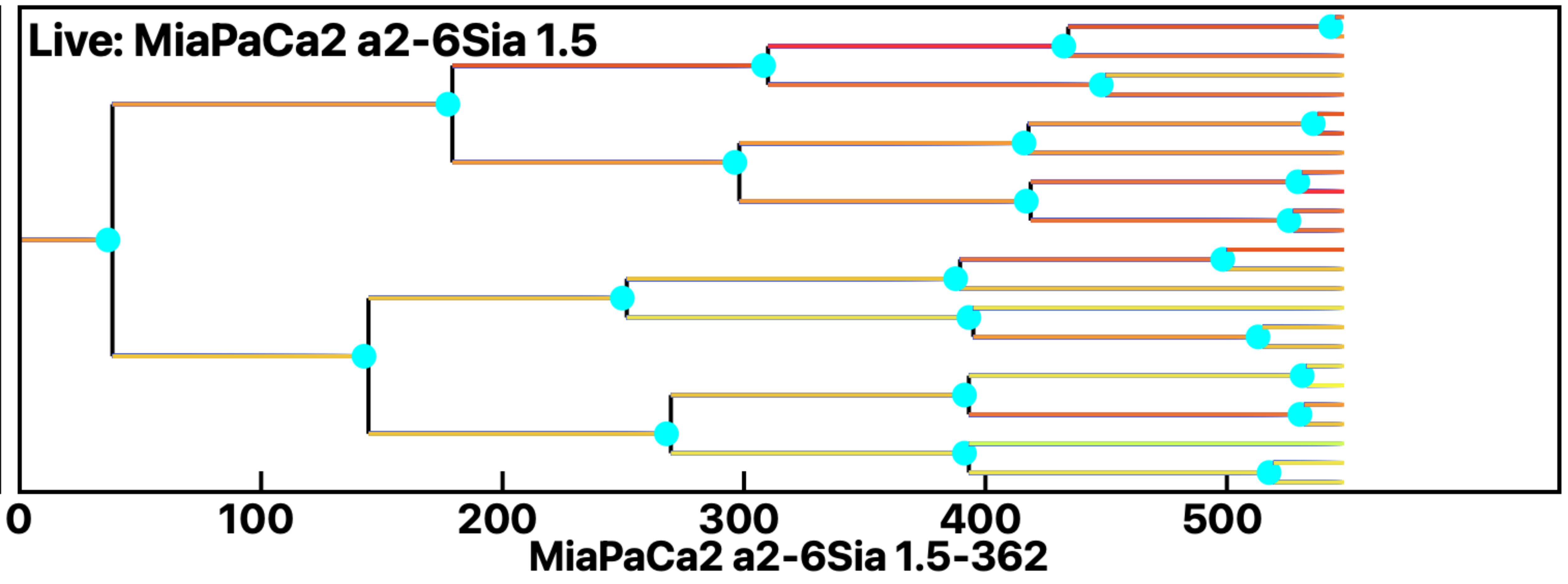

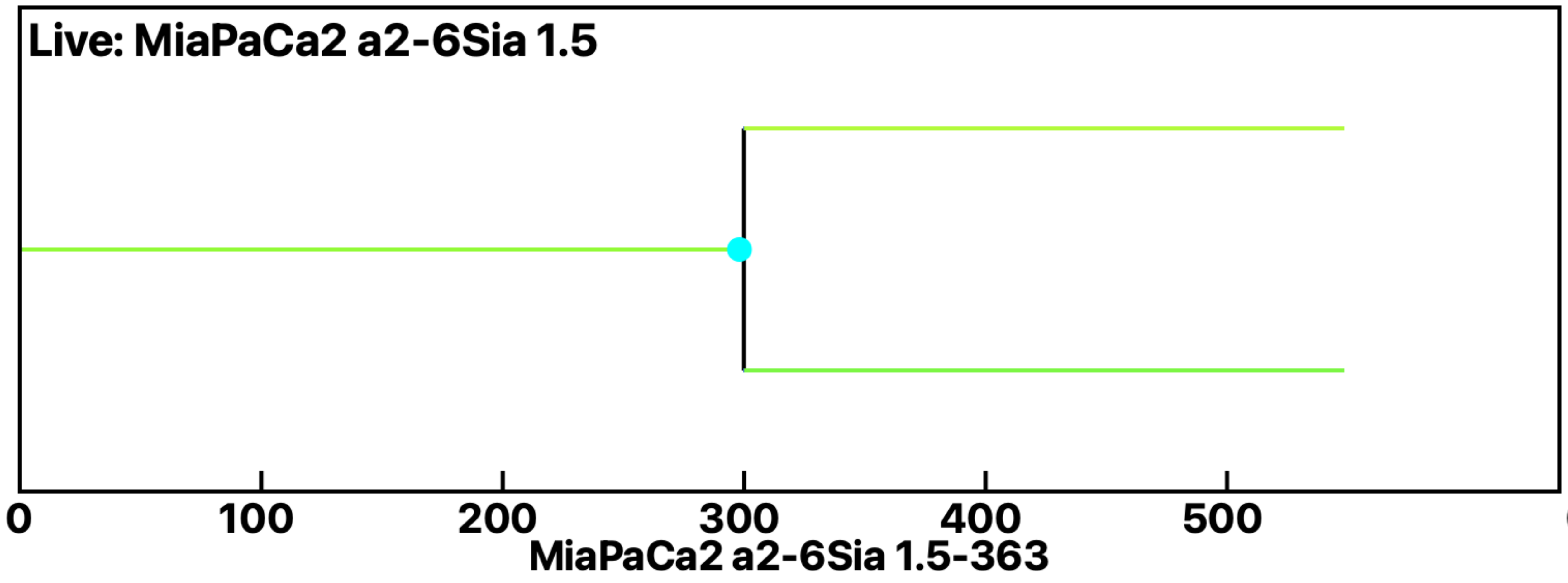

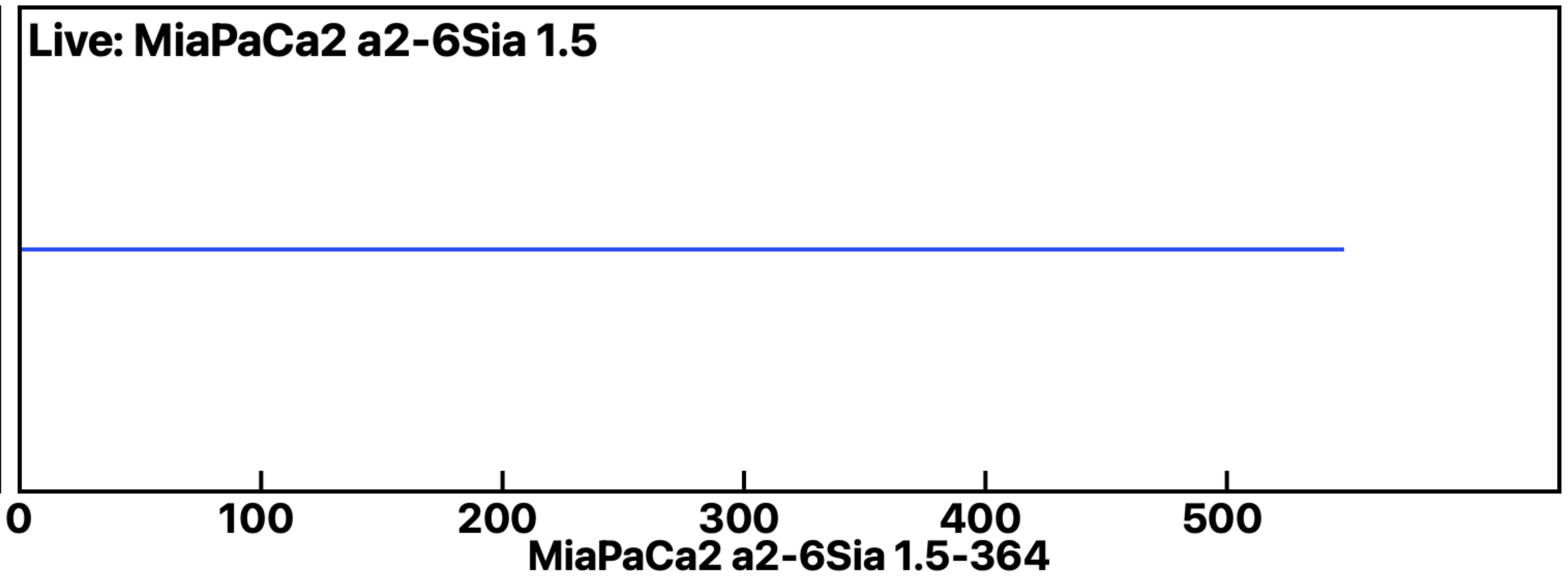

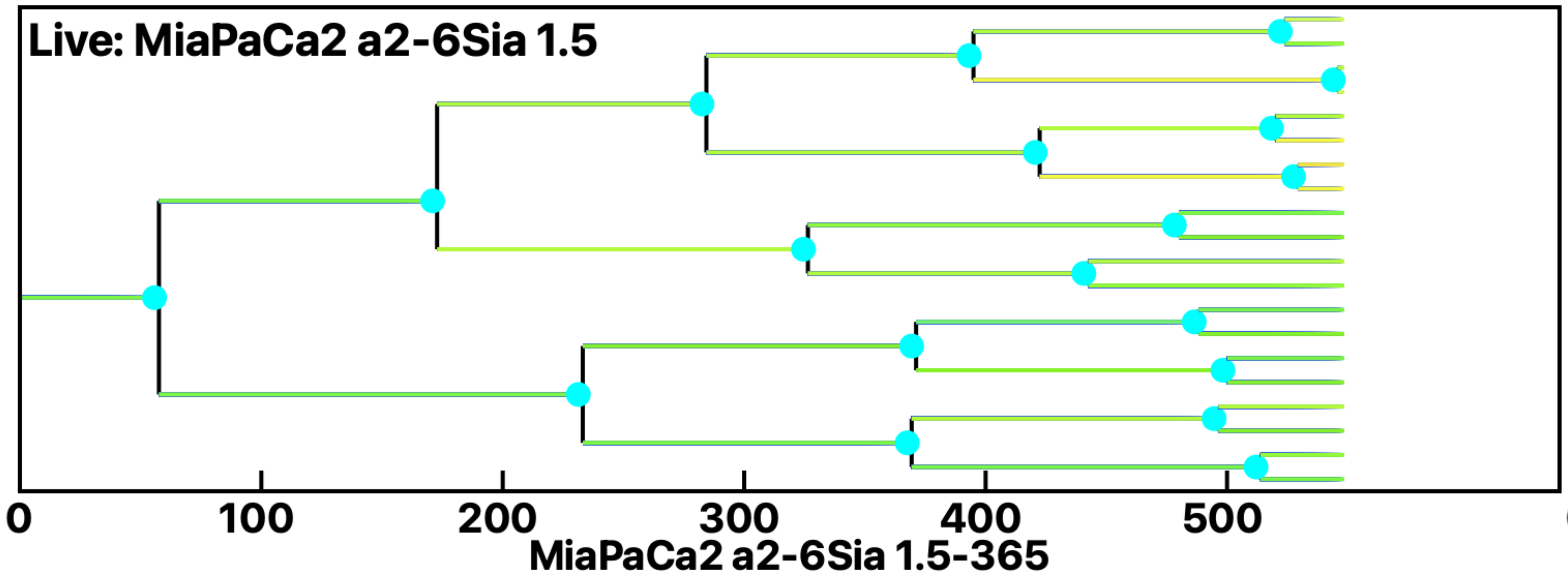

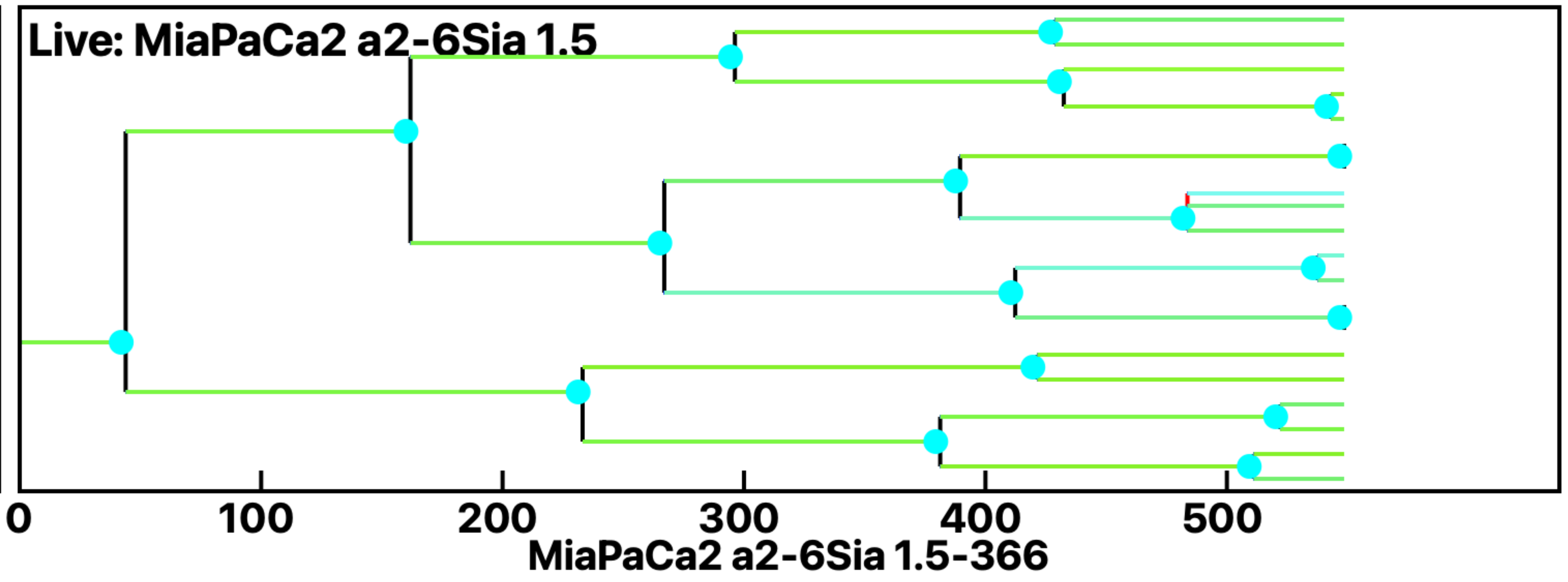

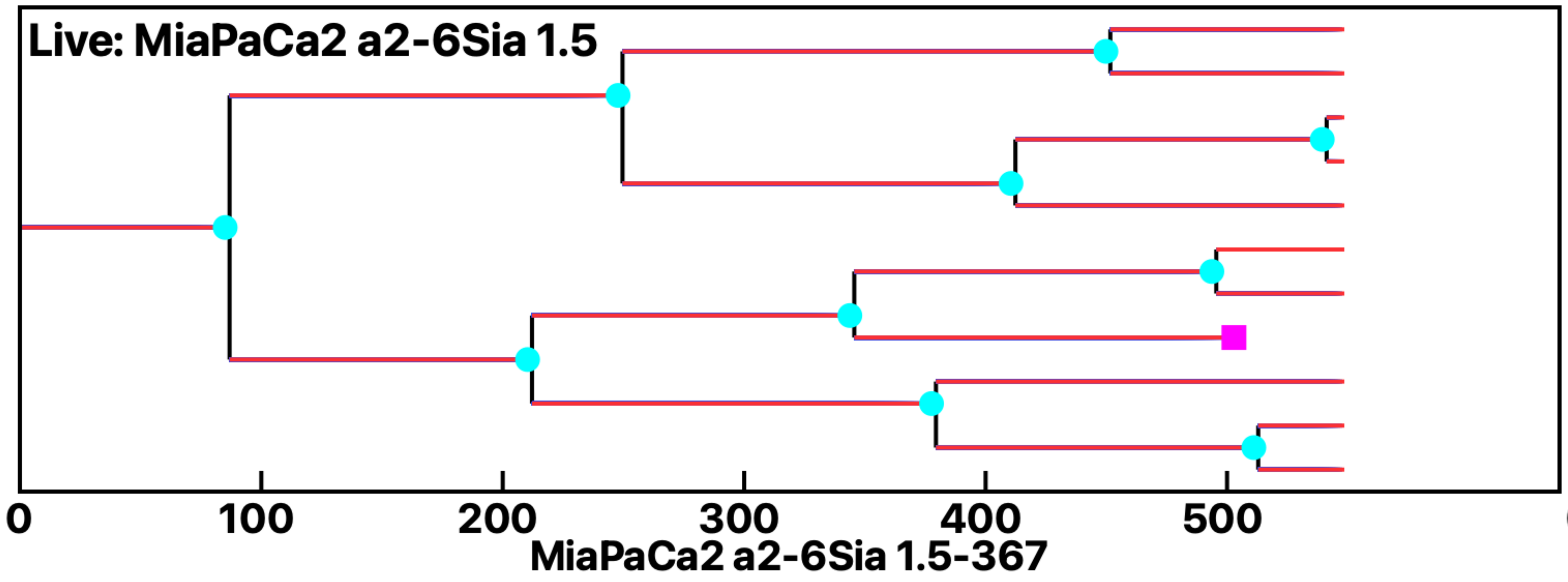

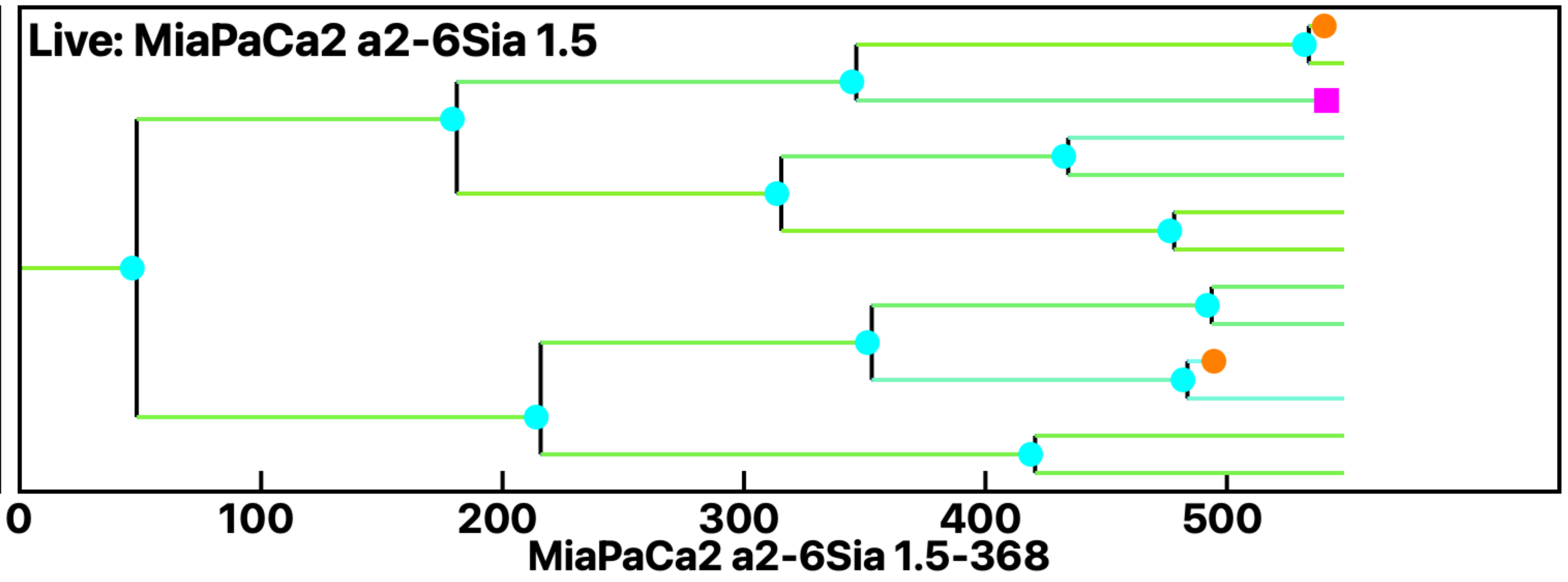

Analysis: Simulation, Treat.: MiaPaCa2 a2-6Sia 1.5, Cell: MiaPaCa2-Simulation

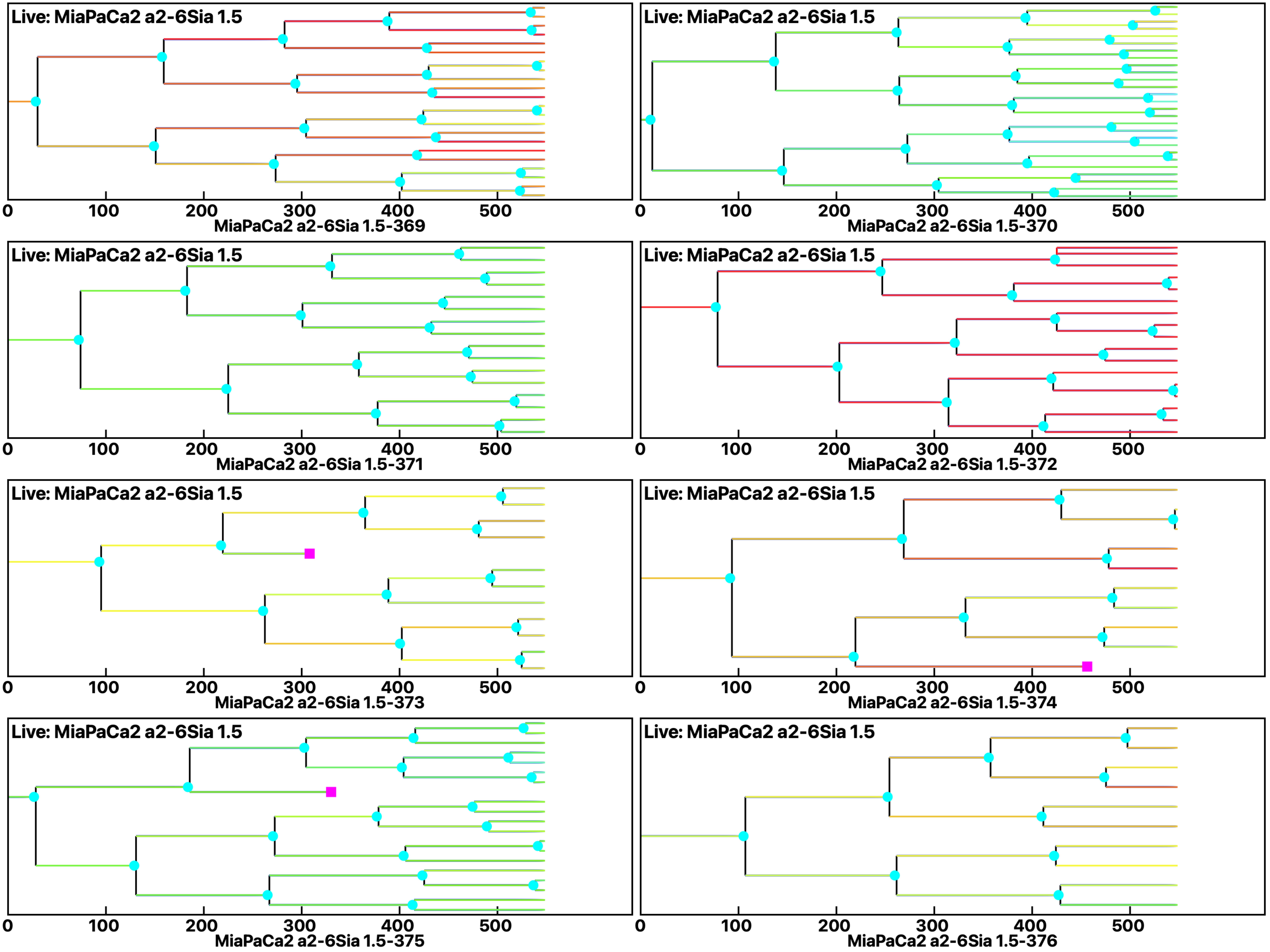

Analysis: Simulation, Treat.: MiaPaCa2 a2-6Sia 1.5, Cell: MiaPaCa2-Simulation

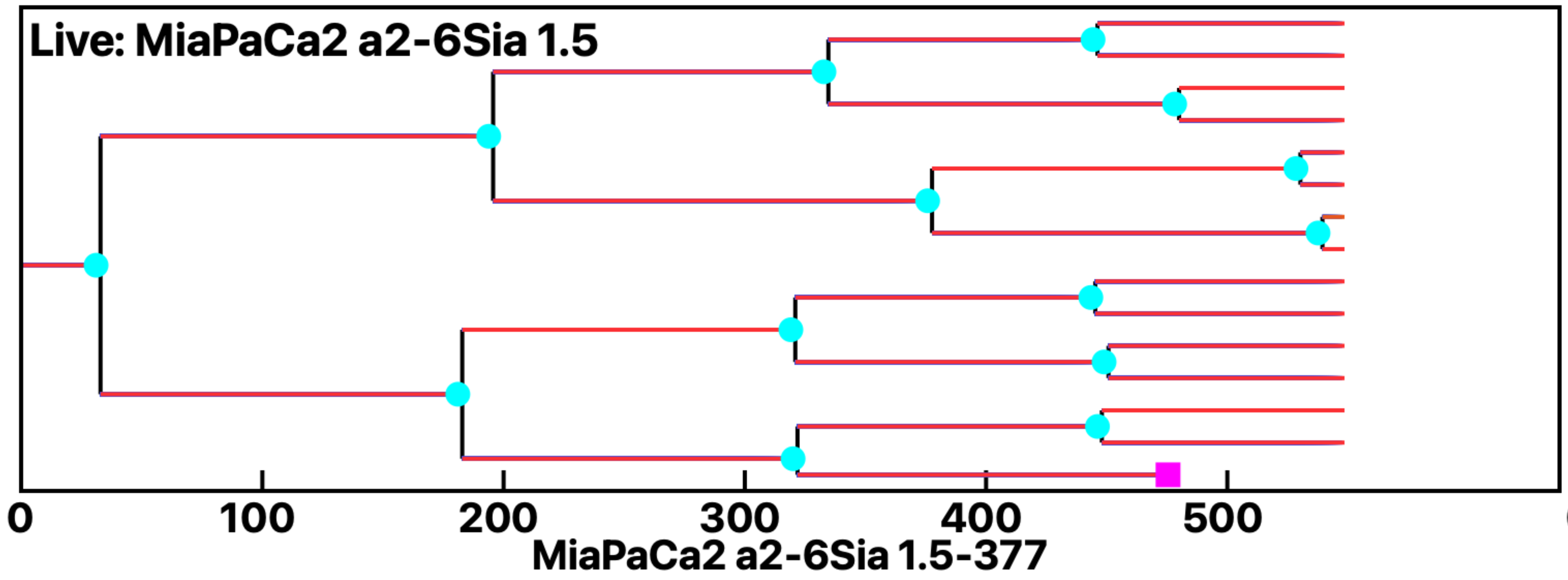

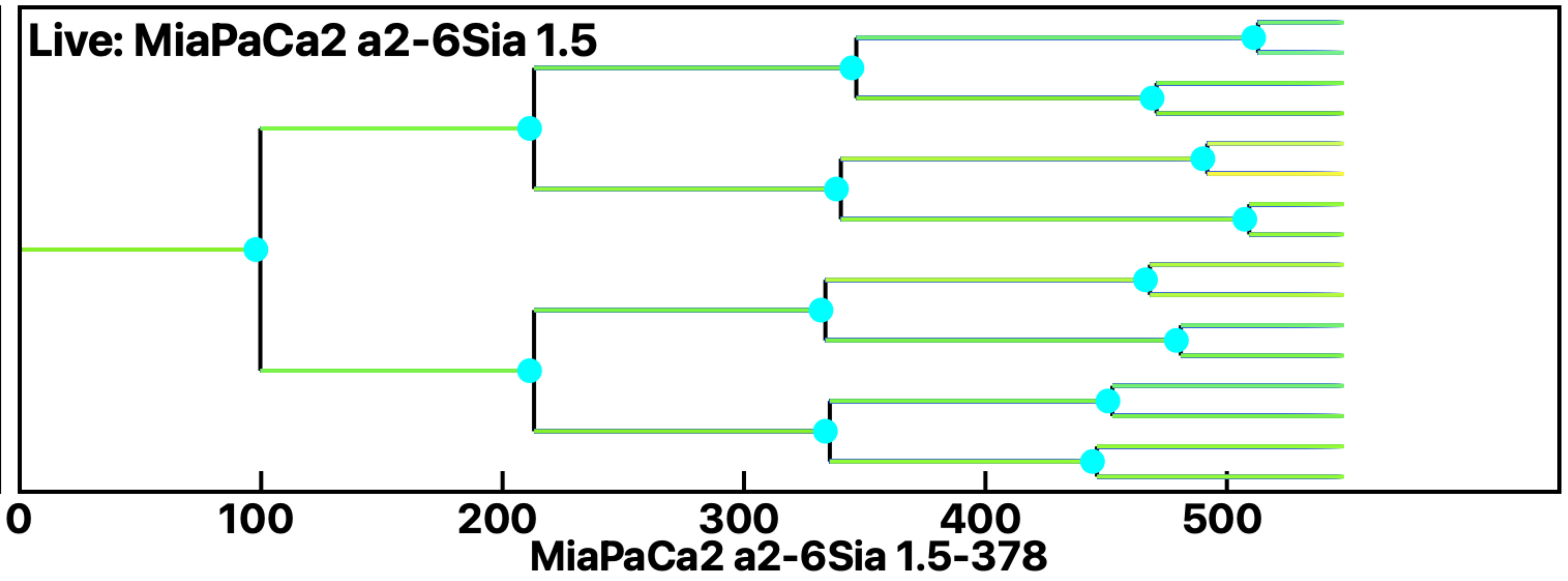

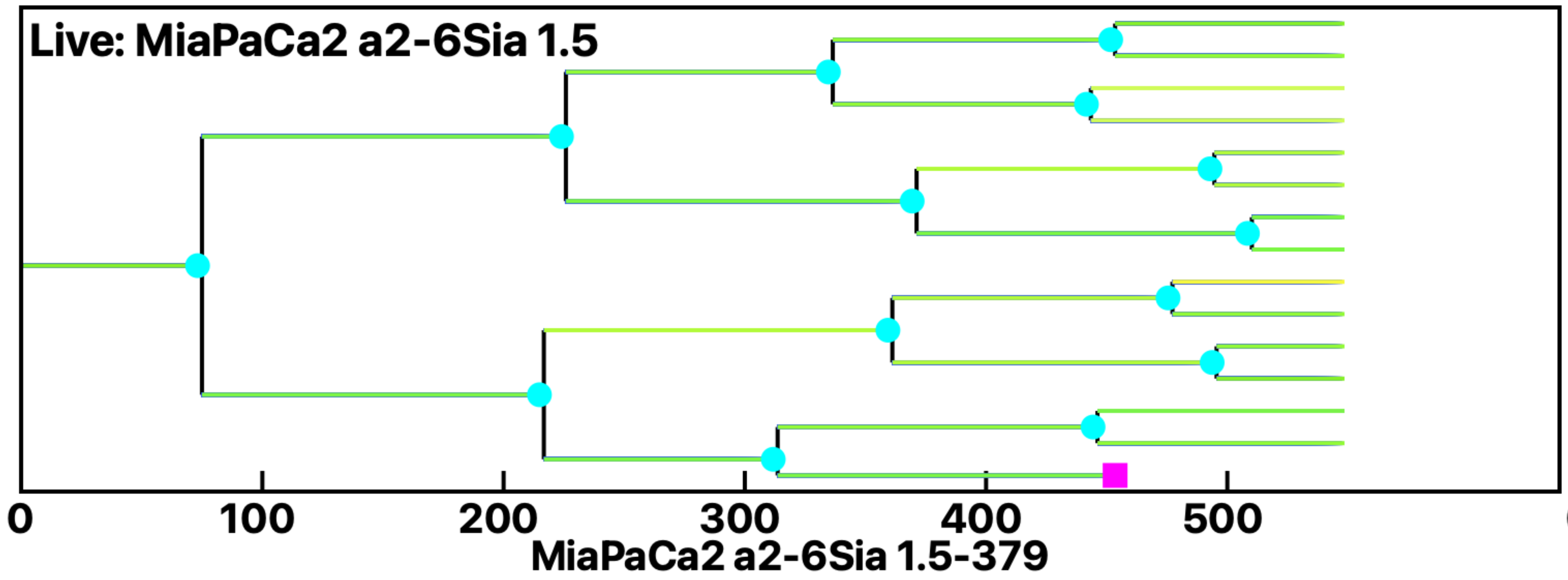

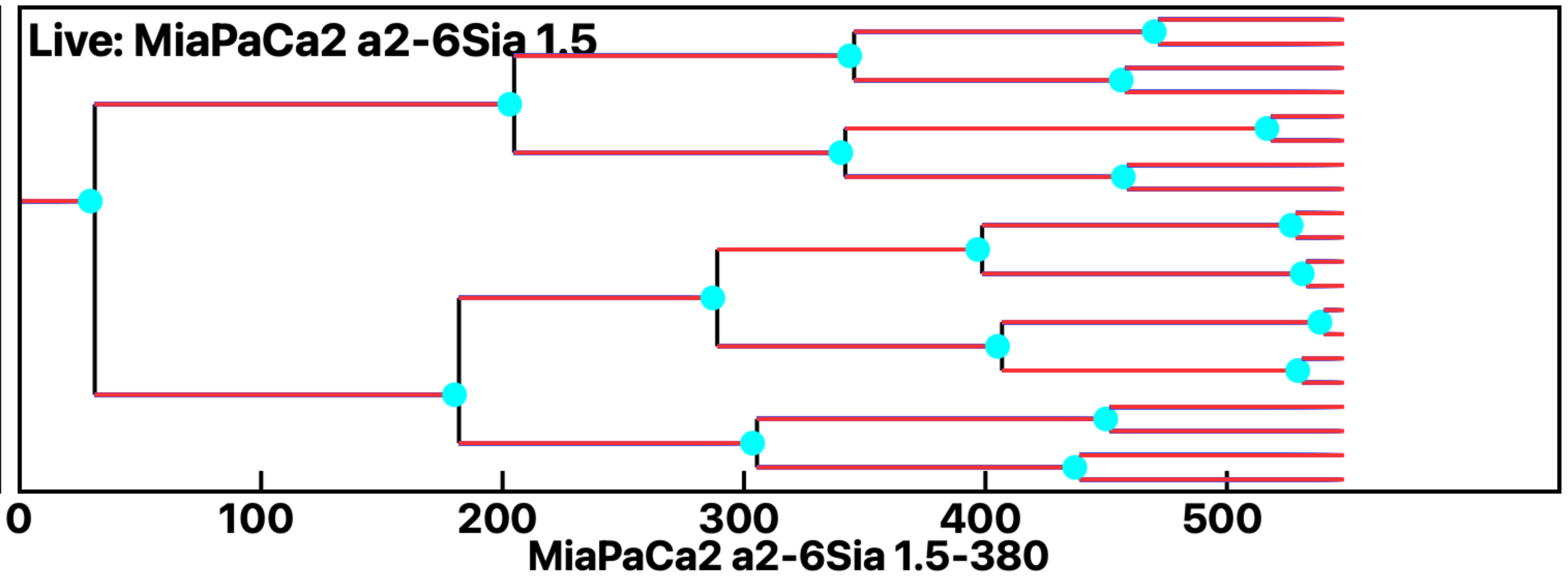

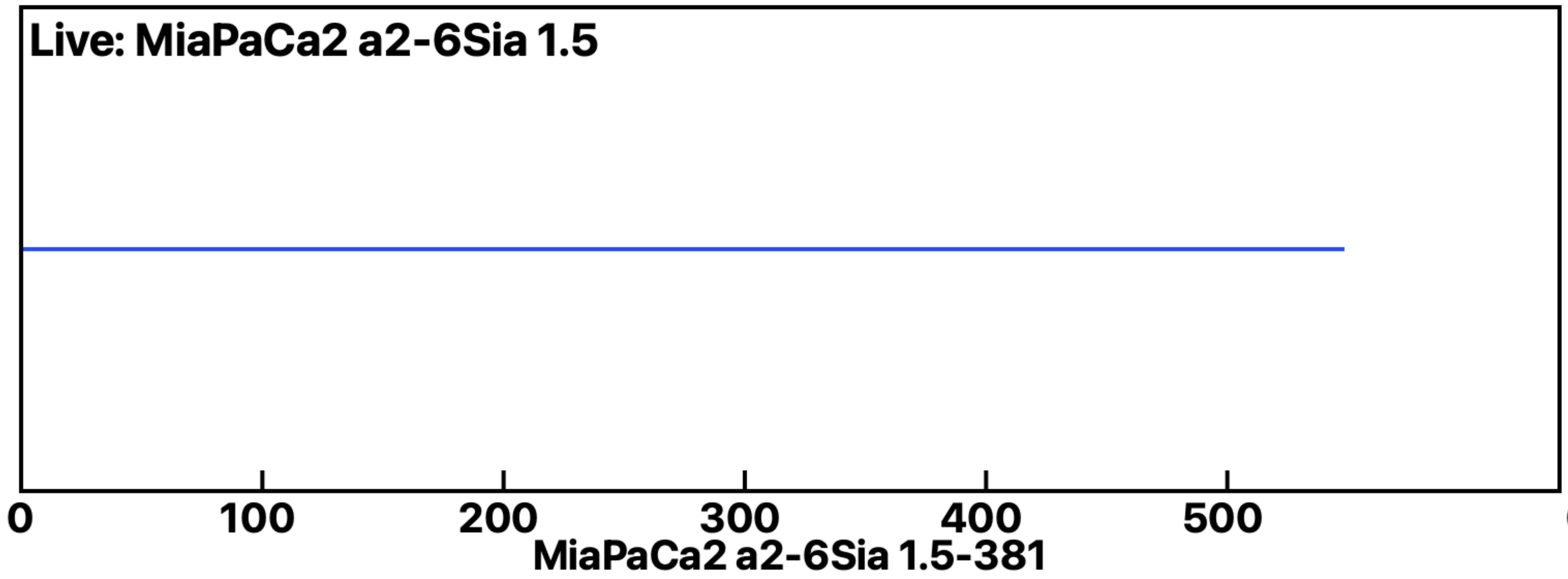

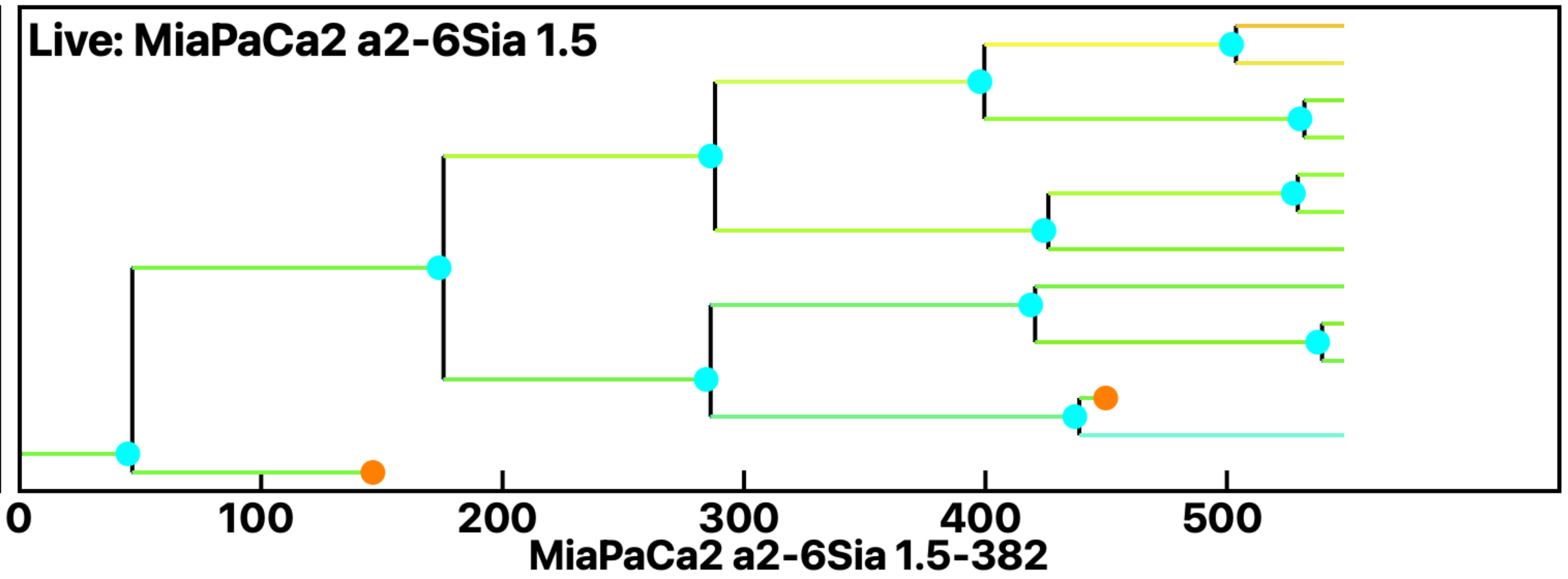

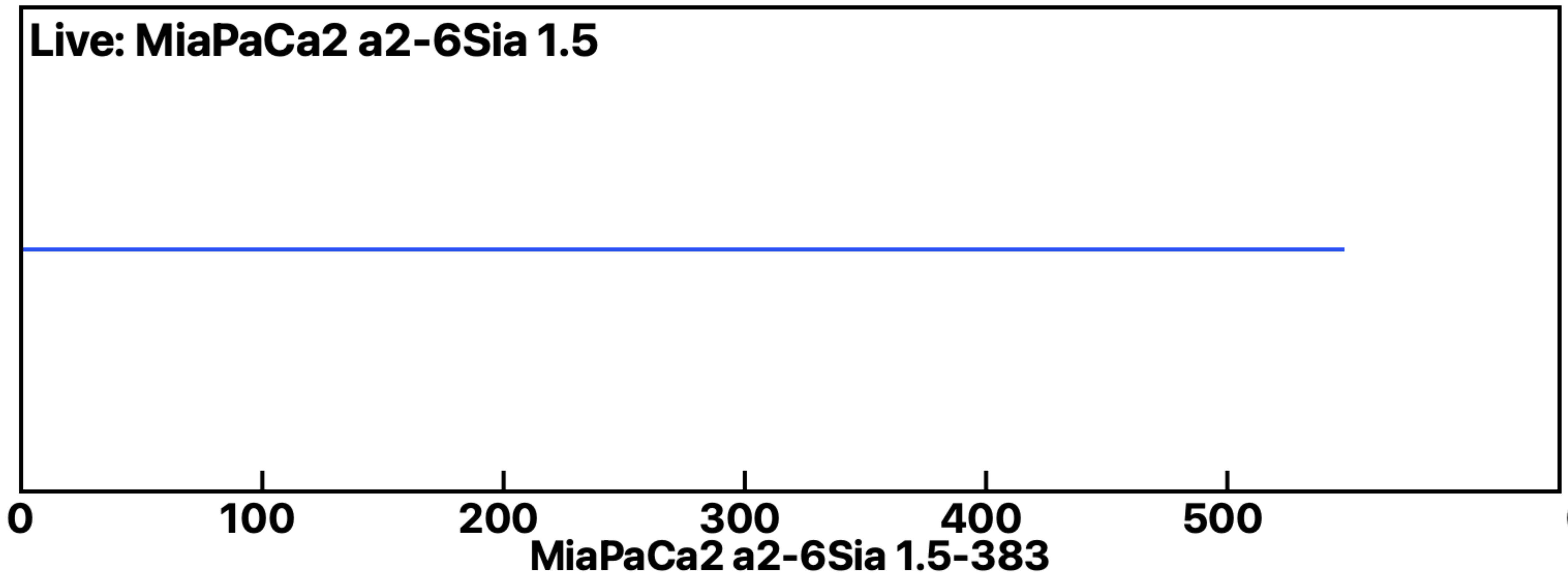

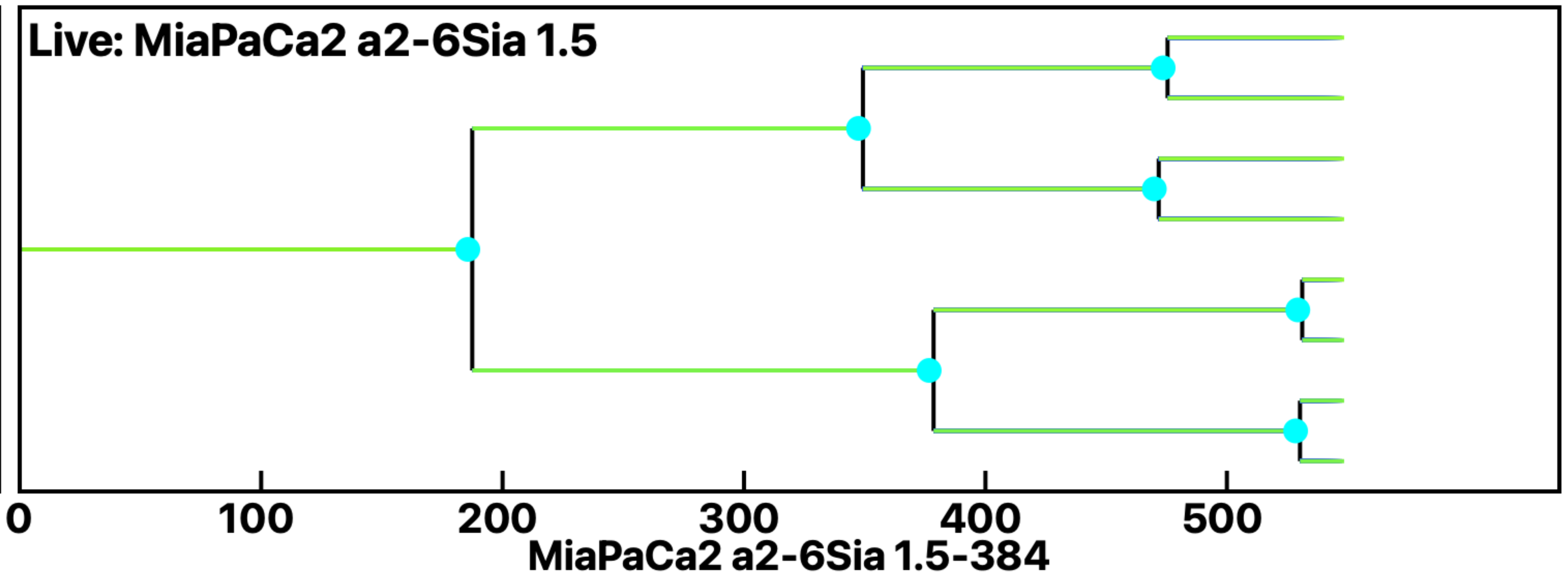

Analysis: Simulation, Treat.: MiaPaCa2 a2-6Sia 1.5, Cell: MiaPaCa2-Simulation

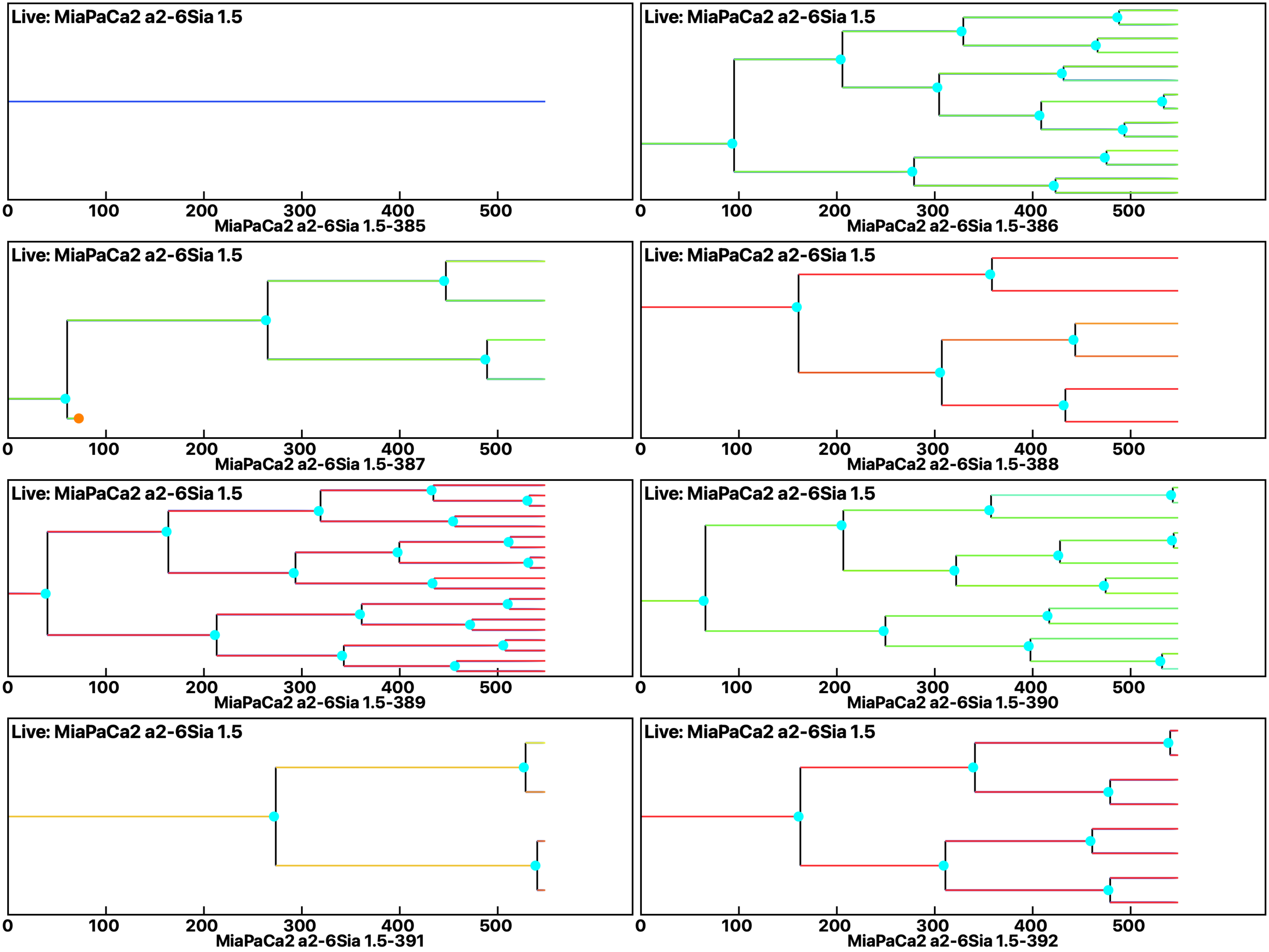

Analysis: Simulation, Treat.: MiaPaCa2 a2-6Sia 1.5, Cell: MiaPaCa2-Simulation

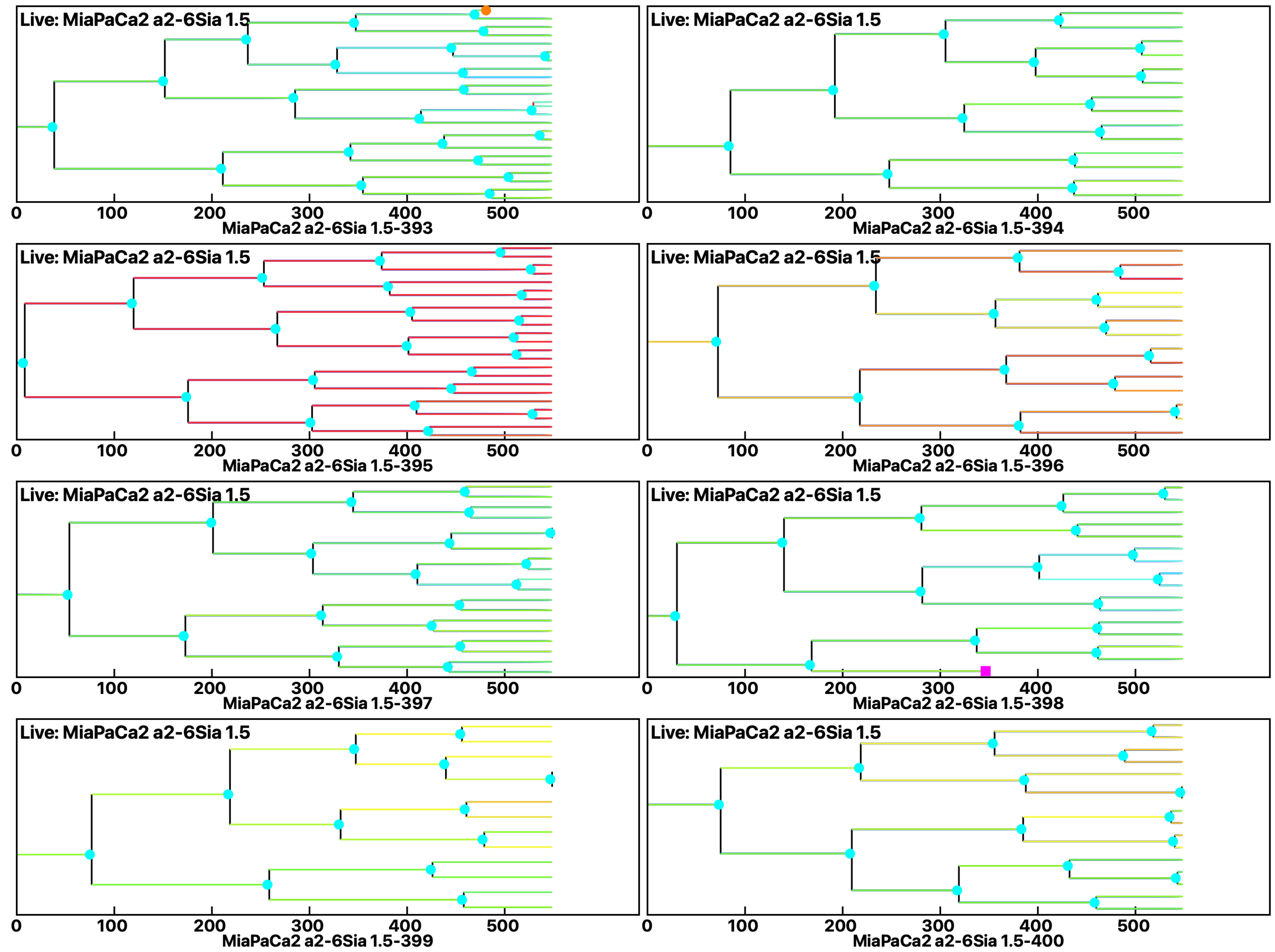

Analysis: Simulation, Treat.: MiaPaCa2 a2-6Sia 1.5, Cell: MiaPaCa2-Simulation

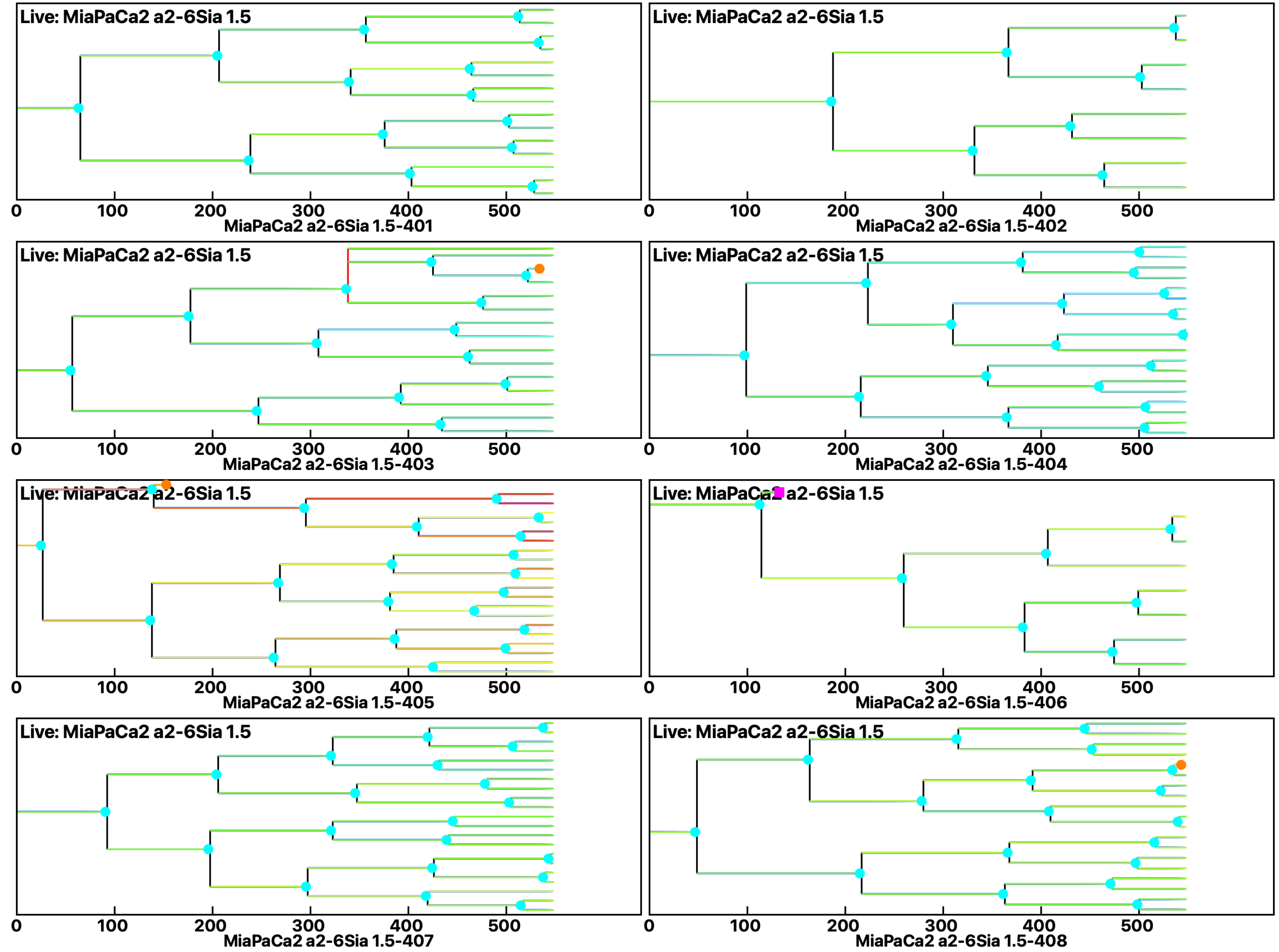

Analysis: Simulation, Treat.: MiaPaCa2 a2-6Sia 1.5, Cell: MiaPaCa2-Simulation

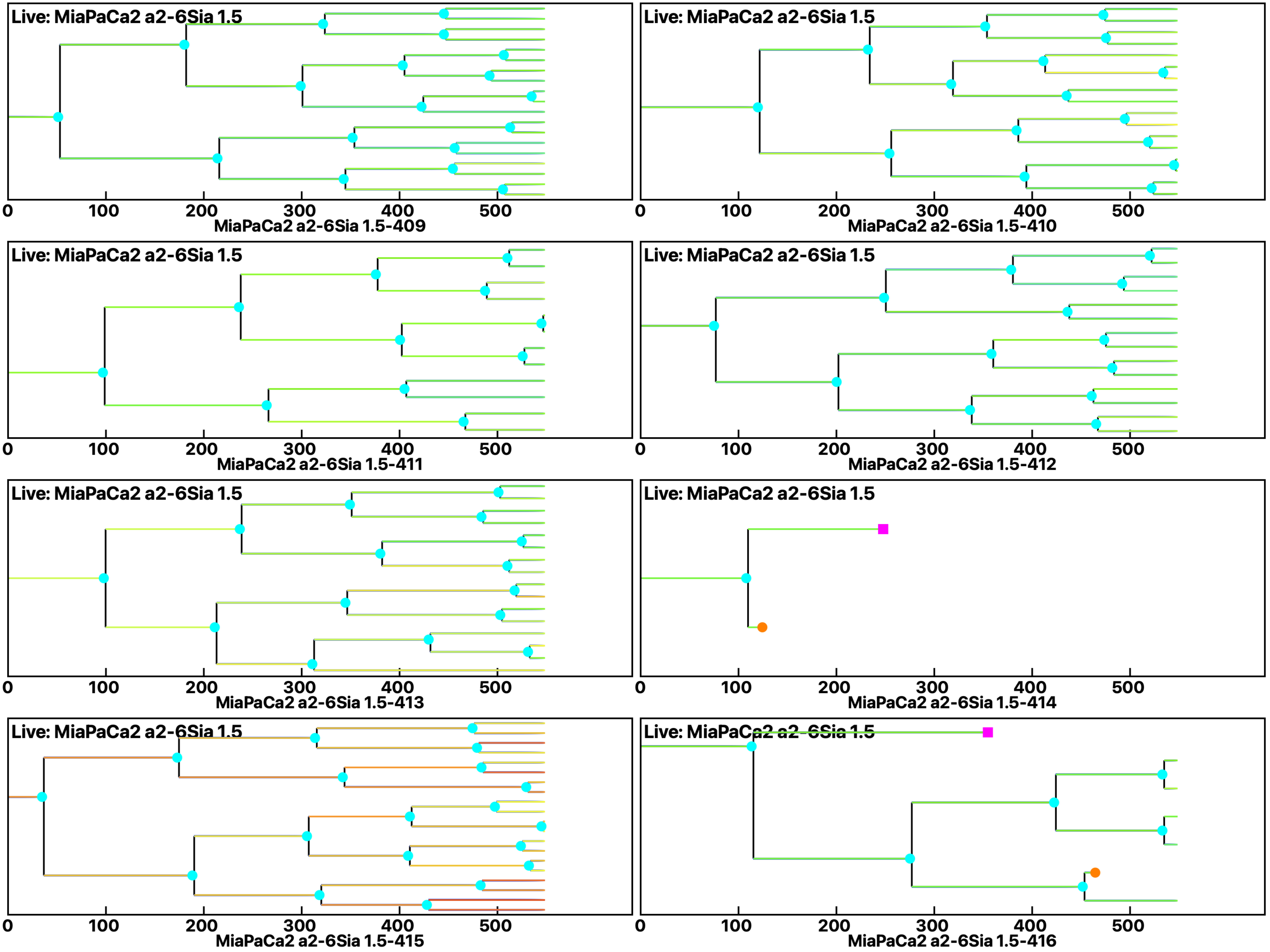

Analysis: Simulation, Treat.: MiaPaCa2 a2-6Sia 1.5, Cell: MiaPaCa2-Simulation

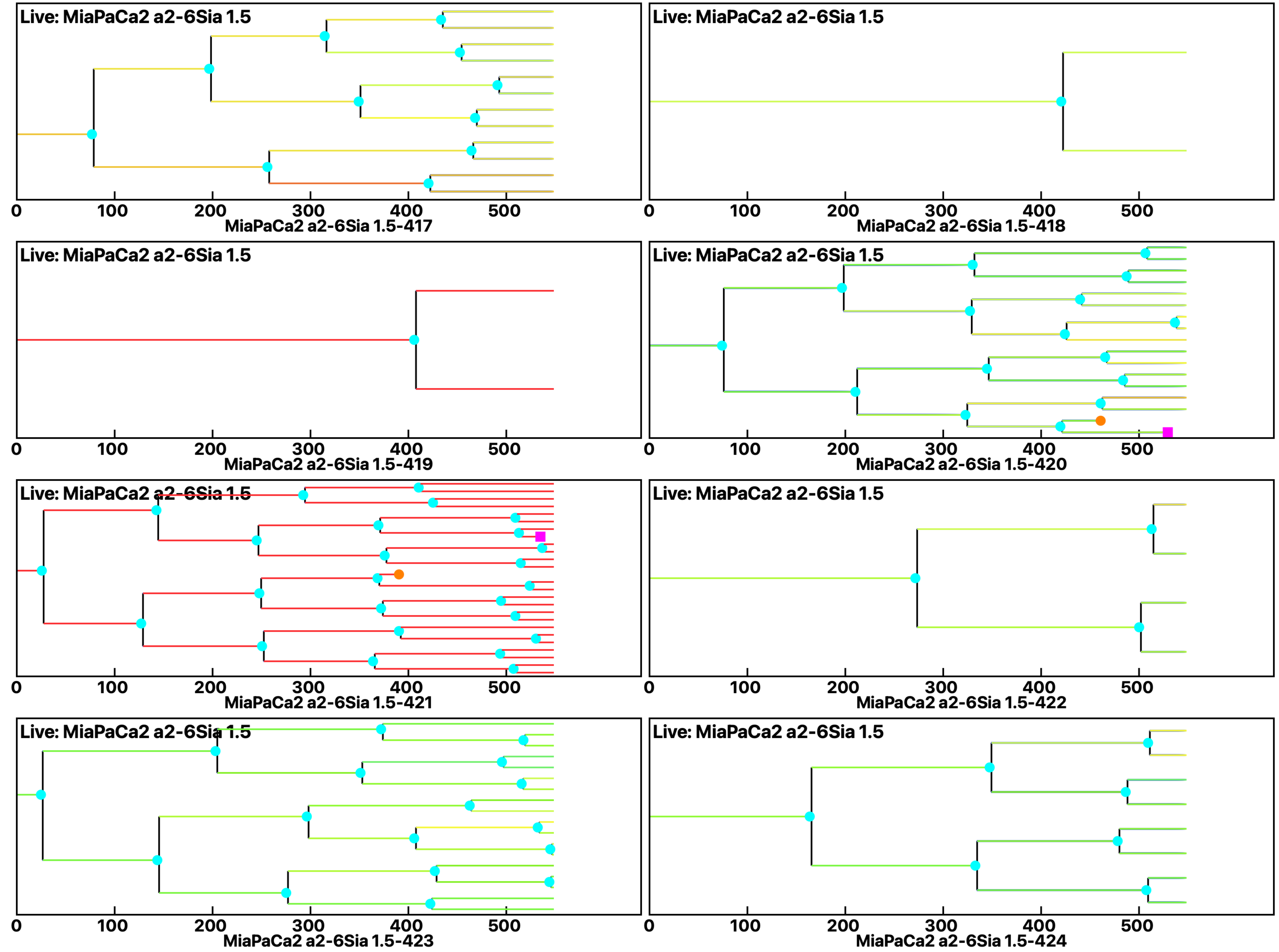

Analysis: Simulation, Treat.: MiaPaCa2 a2-6Sia 1.5, Cell: MiaPaCa2-Simulation

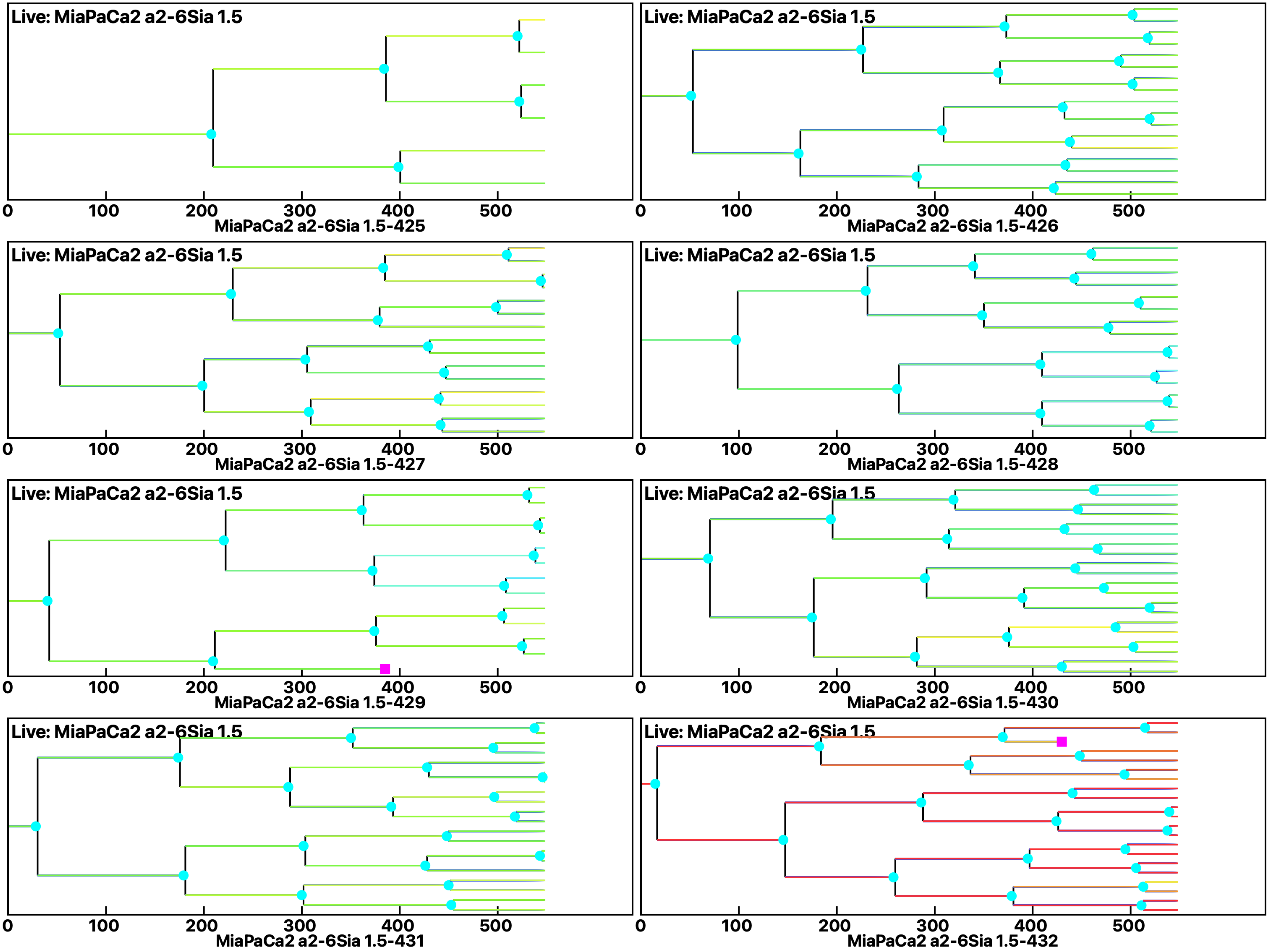

Analysis: Simulation, Treat.: MiaPaCa2 a2-6Sia 1.5, Cell: MiaPaCa2-Simulation

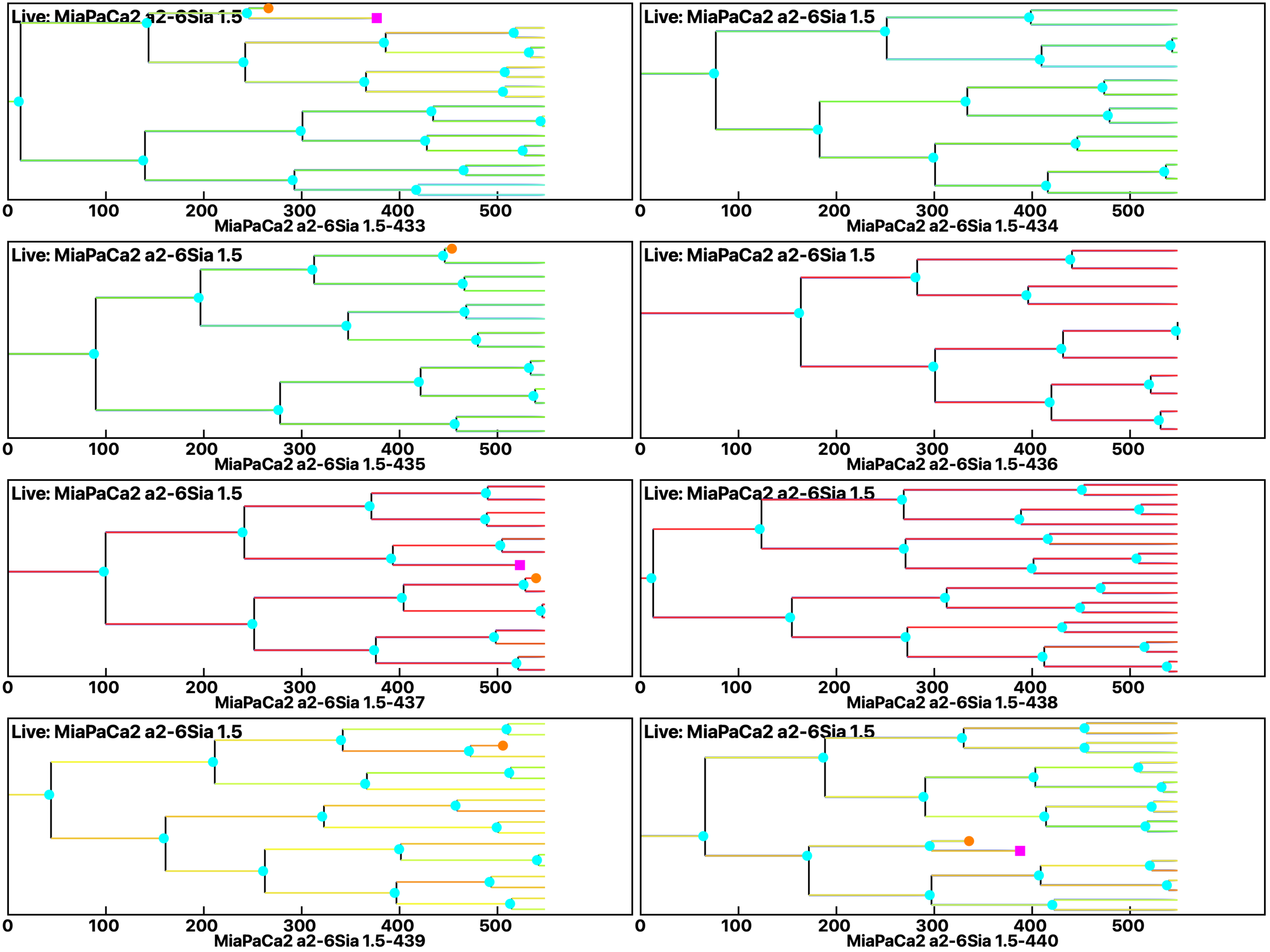

Analysis: Simulation, Treat.: MiaPaCa2 a2-6Sia 1.5, Cell: MiaPaCa2-Simulation

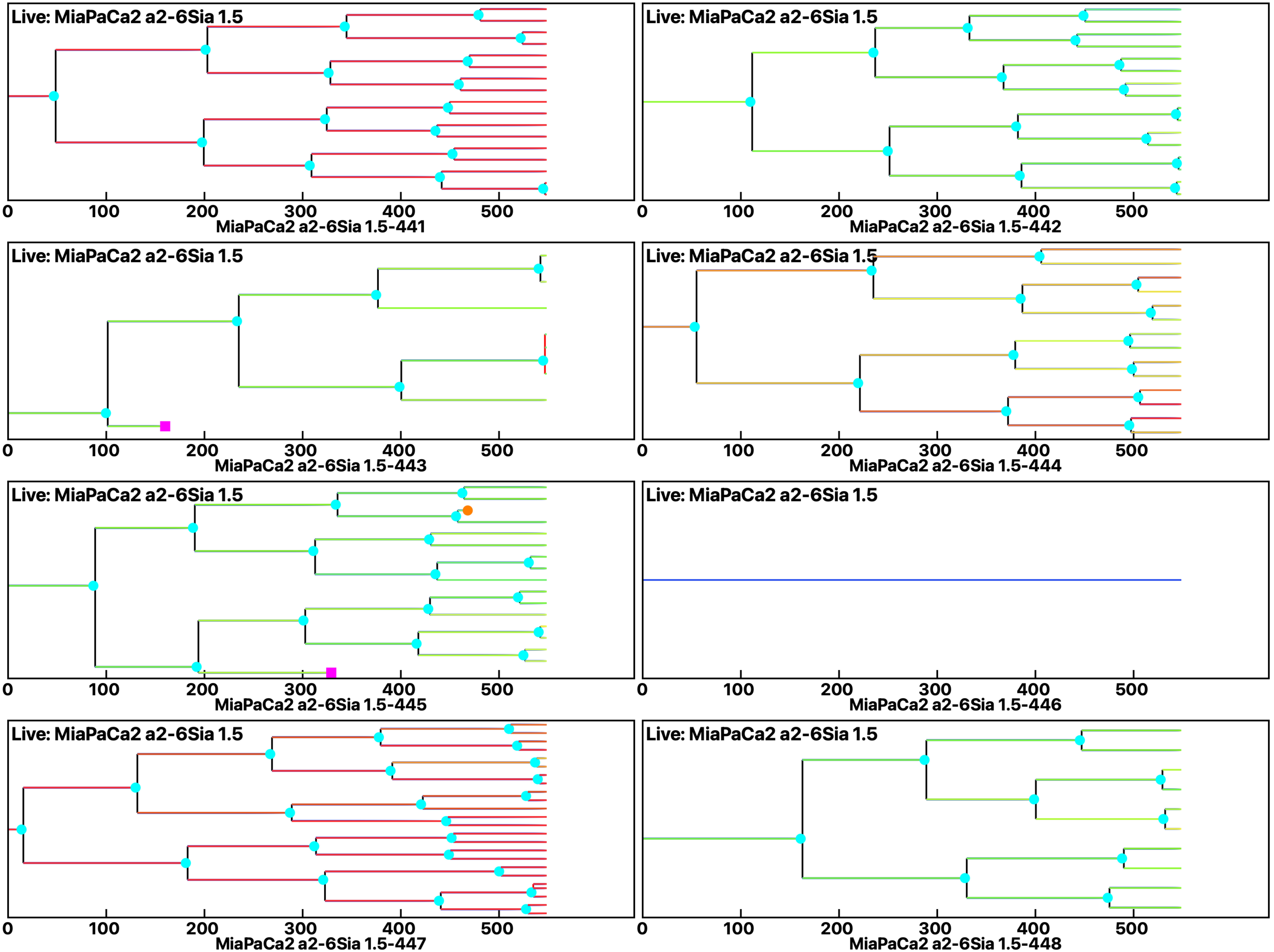

Analysis: Simulation, Treat.: MiaPaCa2 a2-6Sia 1.5, Cell: MiaPaCa2-Simulation

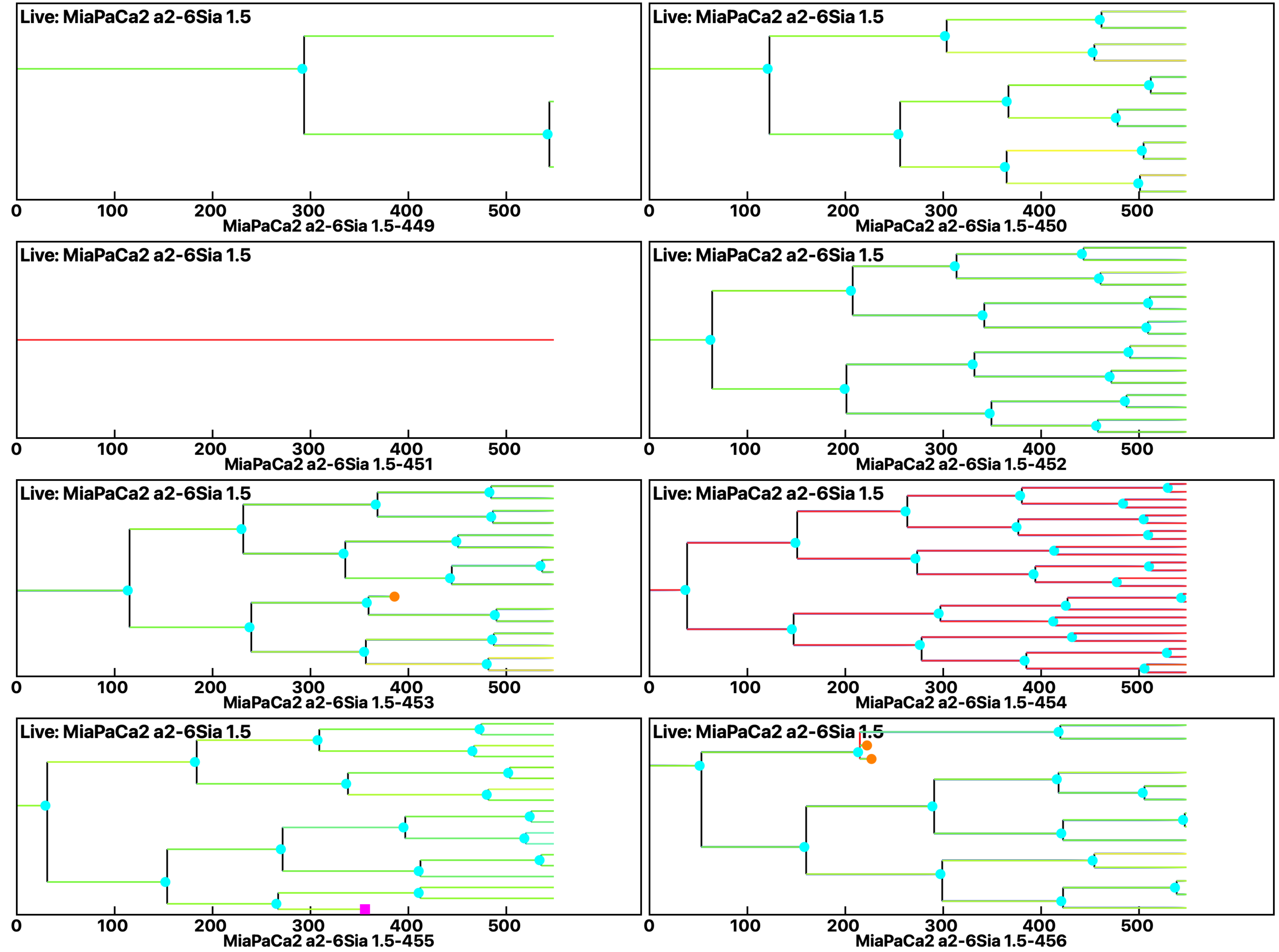

Analysis: Simulation, Treat.: MiaPaCa2 a2-6Sia 1.5, Cell: MiaPaCa2-Simulation

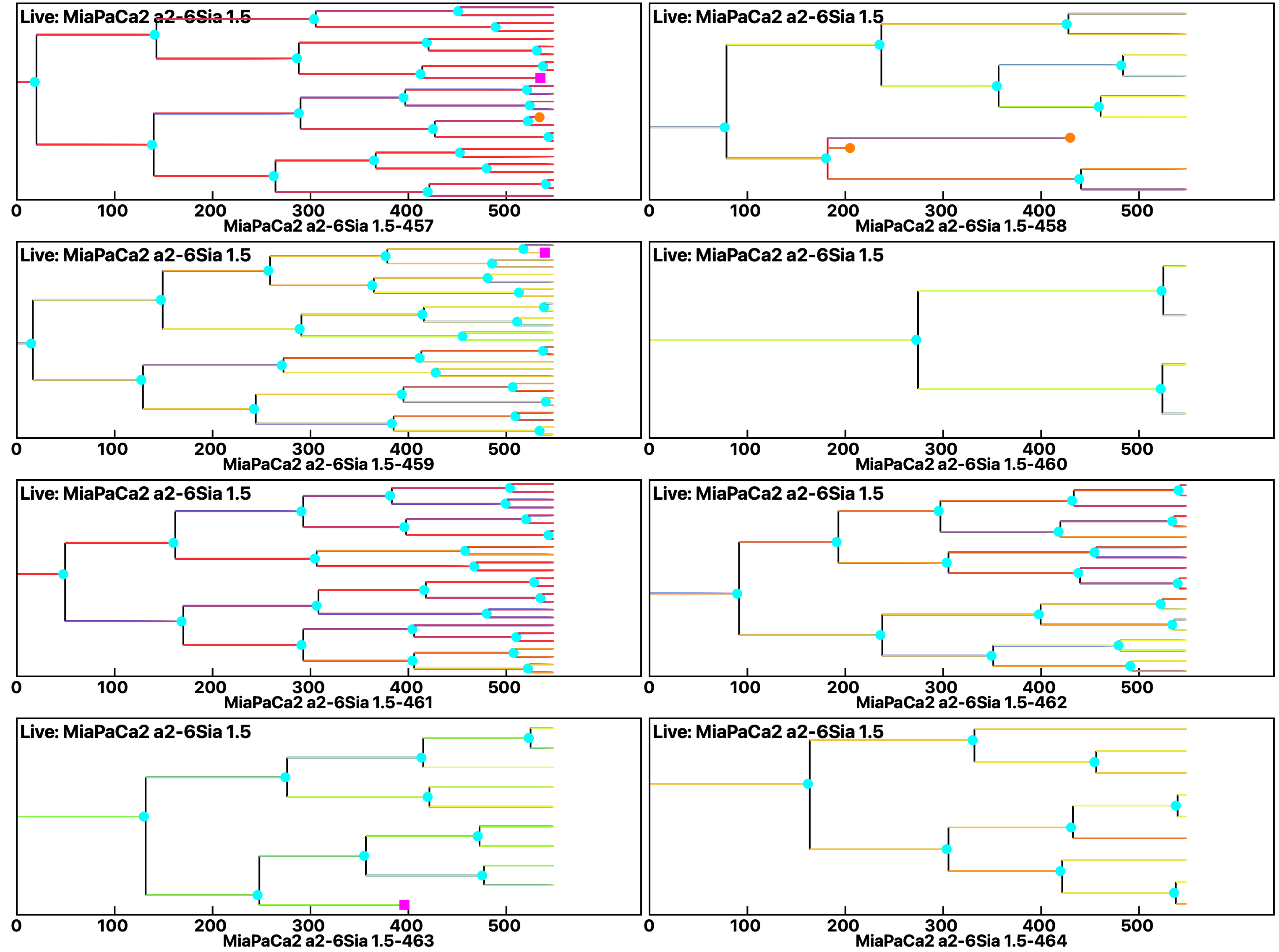

Analysis: Simulation, Treat.: MiaPaCa2 a2-6Sia 1.5, Cell: MiaPaCa2-Simulation

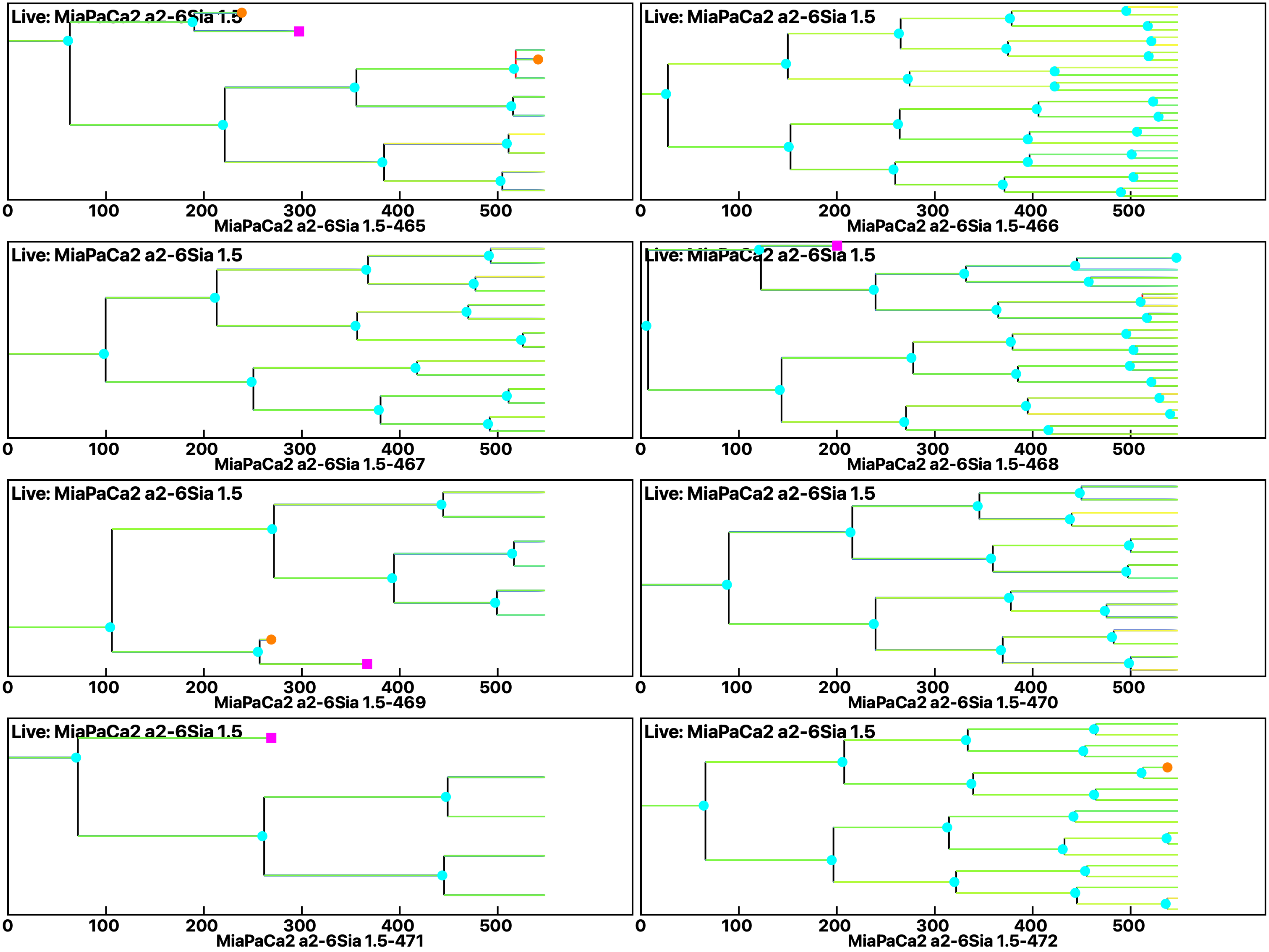

Analysis: Simulation, Treat.: MiaPaCa2 a2-6Sia 1.5, Cell: MiaPaCa2-Simulation

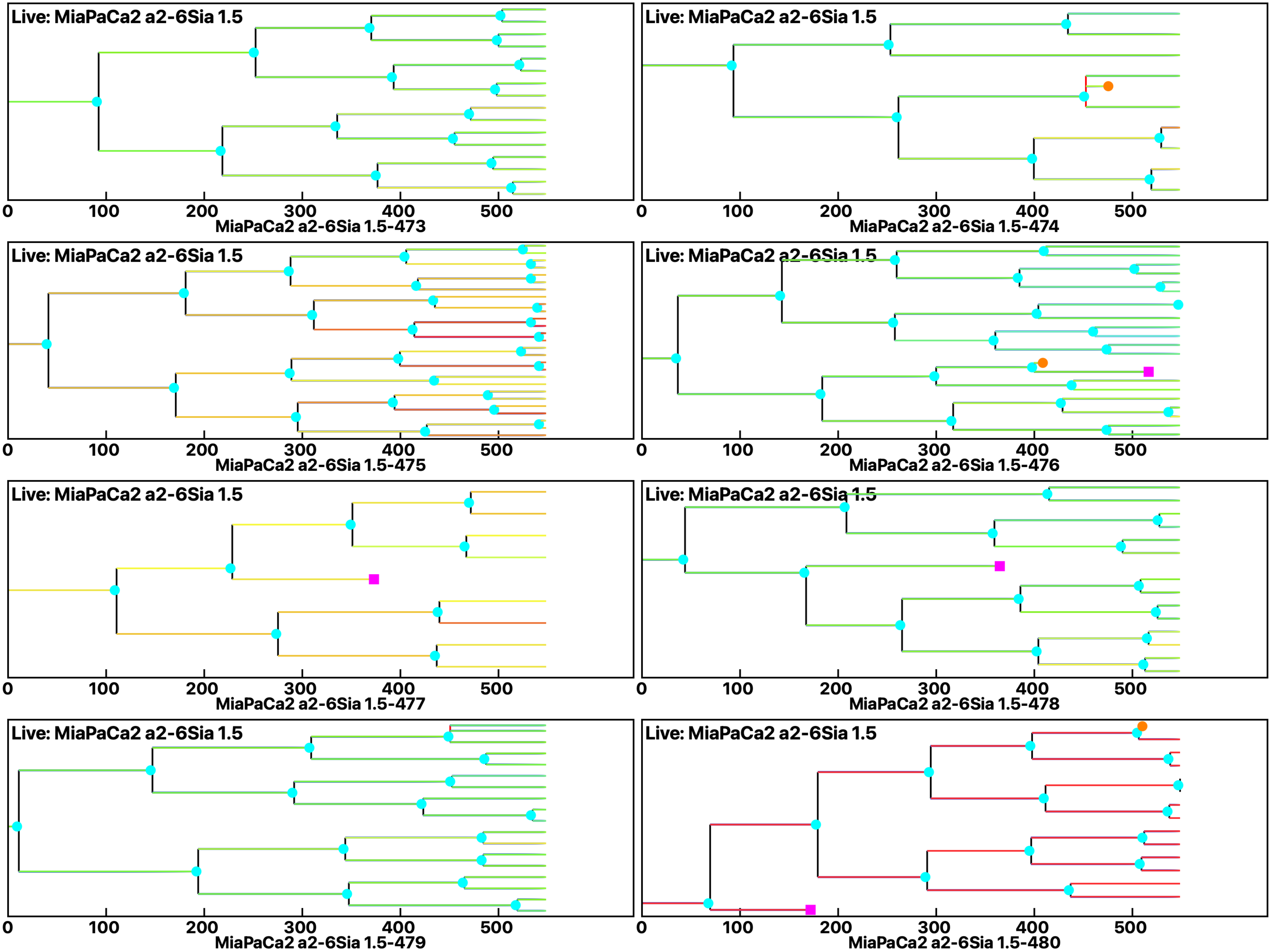

Analysis: Simulation, Treat.: MiaPaCa2 a2-6Sia 1.5, Cell: MiaPaCa2-Simulation

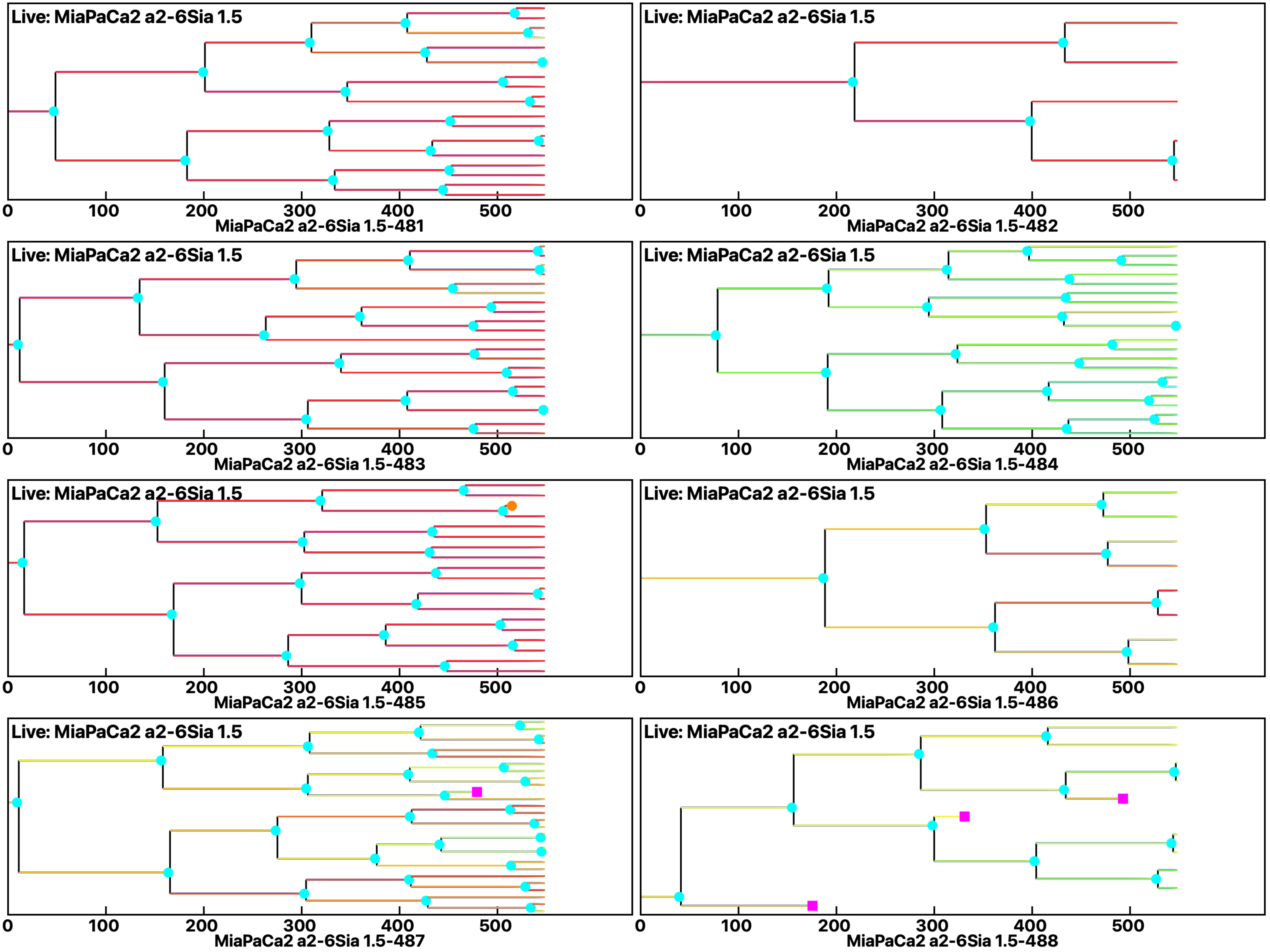

Analysis: Simulation, Treat.: MiaPaCa2 a2-6Sia 1.5, Cell: MiaPaCa2-Simulation

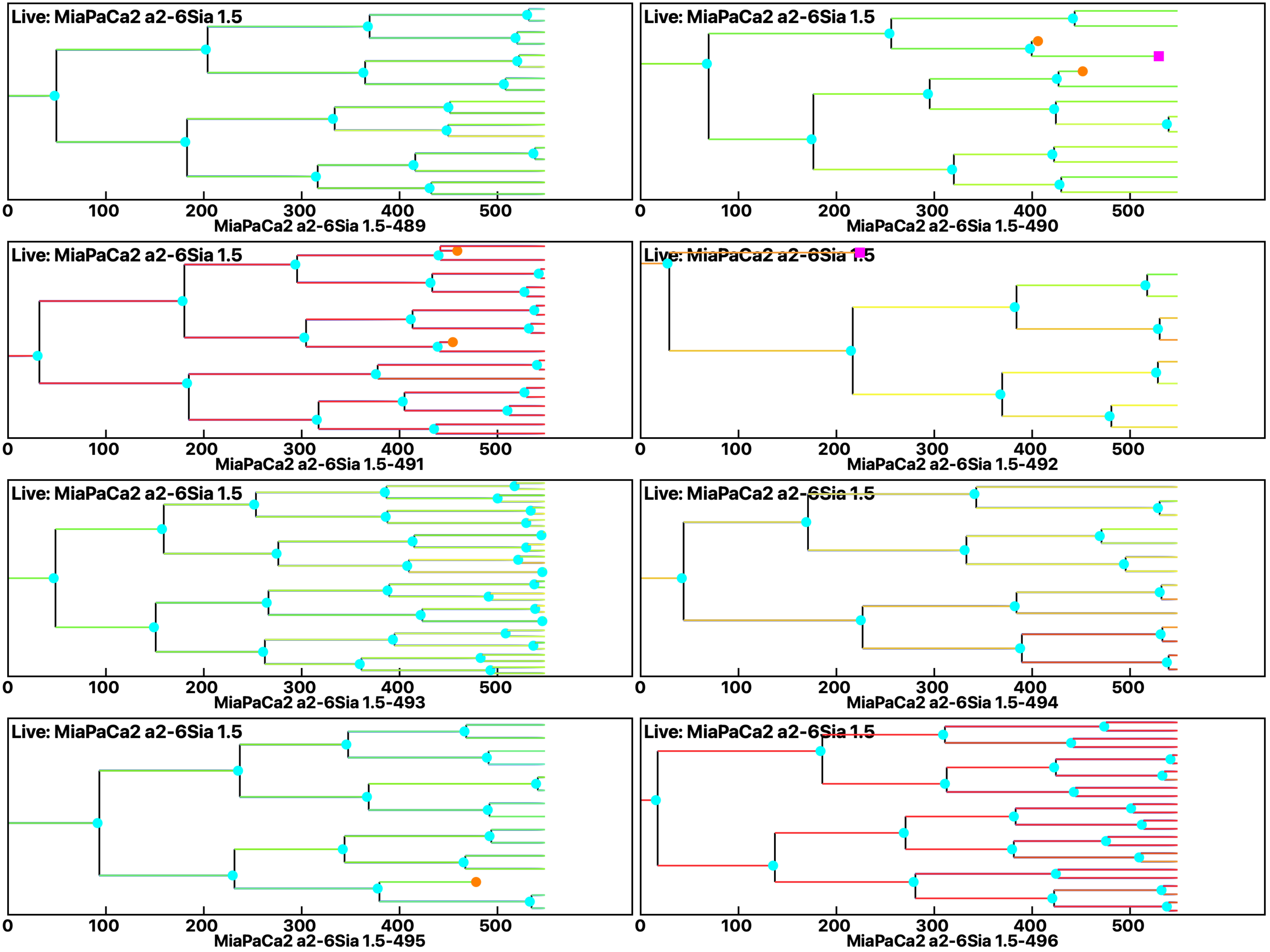

Analysis: Simulation, Treat.: MiaPaCa2 a2-6Sia 1.5, Cell: MiaPaCa2-Simulation

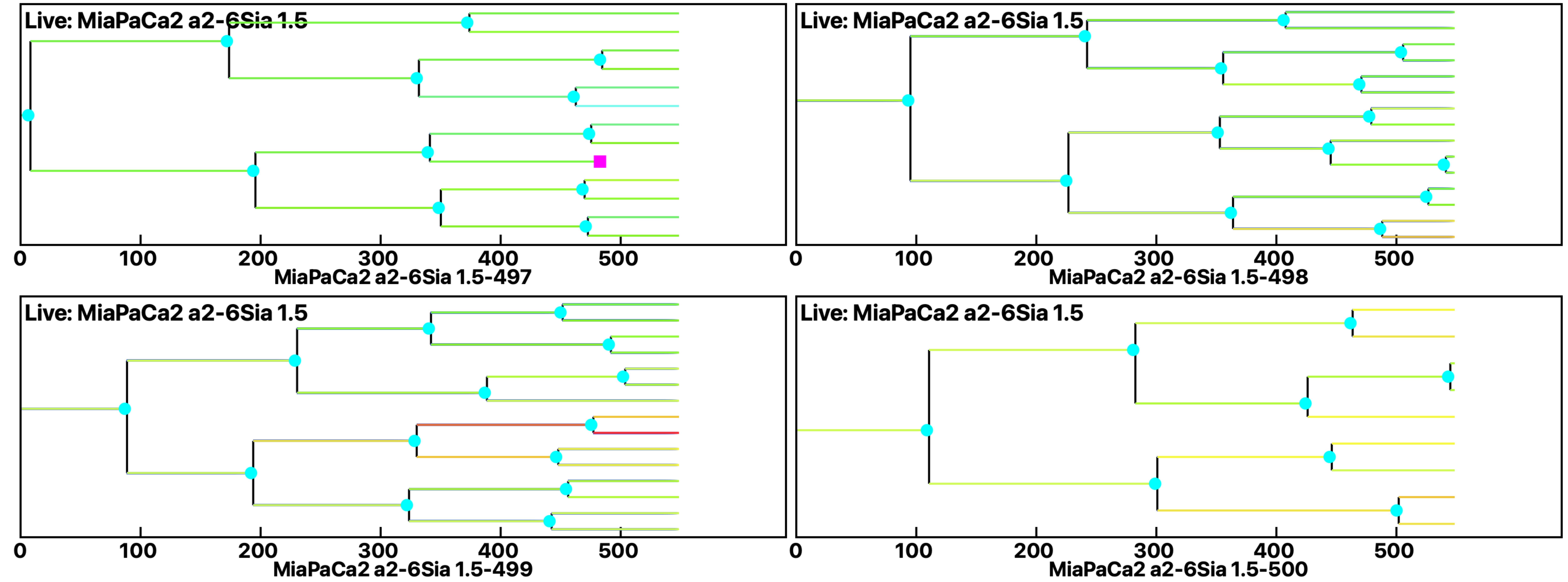

Supplement: Data S6 [file mmc8.pdf]
