# Supplementary material for: Cell fate simulation reveals cancer cell features in the tumor microenvironment
Source: J Biol Chem. 2024 Aug 20;300(9):107697. doi: 10.1016/j.jbc.2024.107697 (PMC11419826; doi:10.1016/j.jbc.2024.107697)
Supplement: Data S7 [file mmc9.pdf]

Cervical cancer

ID: Patients ID
PFS: Progress-free survival

			Cell population size				2-6Sia levels				Number of reproductive cells produced through multipolar cell division				Supressive	Permissive	Lethal
ID	Best call	PFS	α2-6Sia 1.5	α2-6Sia 1.0	α2-6Sia 0.5	α2-6Sia 0.25	α2-6Sia 1.5	α2-6Sia 1.0	α2-6Sia 0.5	α2-6Sia 0.25	α2-6Sia 1.5	α2-6Sia 1.0	α2-6Sia 0.5	α2-6Sia 0.25	%	%	%
TCGA-EK-A2H1	1	540	2176	2177.096168	2384.441946	2628.693638	34998	13186.3196	9252.458323	0	0	2.90021692	7.843559032	2.773858921	30.33849818	11.93476046	28.94180725
TCGA-EK-A2H0	1	463	2193	2137.45987	2244.238328	2690.643154	34245	15717.24223	9010.288438	0	0	1.933477946	0	1.849239281	30.7022515	13.48080487	27.18338285
TCGA-ZJ-A8QQ	1	0	2414	2439.08243	2622.690051	2750.74343	37911	15435.92119	10127.01516	0	0	0	3.921779516	1.849239281	29.83843431	11.61566898	29.45263663
TCGA-ZJ-AB0I	1	186	2250	2093.956616	2267.769005	2873.717842	33002	13092.54591	8670.074065	0	0	2.90021692	3.921779516	0.92461964	27.78347746	14.64302895	29.71588081
TCGA-DS-A0VL	1	4483	2205	2247.668113	2591.315815	2722.080221	35274	14765.00434	10070.14935	0	0	2.90021692	7.843559032	0.92461964	31.68663093	13.70892797	28.61811037
TCGA-EK-A2RE	1	4879	2966	3080.997108	3063.890247	3293.495159	42673	21029.47289	12002.60621	0	0	4.833694866	7.843559032	0.92461964	25.47337226	4.100999081	37.05061236
TCGA-C5-A7XC	1	2226	2147	2471.951555	2522.684674	2807.145228	32717	16279.88431	9703.462967	0	0	6.767172813	3.921779516	2.773858921	32.74730454	12.98062972	28.71909585
TCGA-GH-A9DA	1	1221	2375	2378.177874	2625.631386	2680.472337	37938	18028.71511	10286.82767	0	0	1.933477946	0	0.92461964	30.79158269	11.63143511	29.31645221
TCGA-MU-A51Y	1	90	2309	2245.734635	2217.766316	2712.834025	34772	12863.42878	8456.337081	0	0	0	0	1.849239281	32.5259247	11.53004488	29.36711107
TCGA-EK-A2IP	1	586	2046	2129.725958	2445.229528	2858.923928	34070	14300.00289	9862.295038	0	0	1.933477946	7.843559032	1.849239281	30.54236825	14.23850954	29.0754164
TCGA-MY-A913	1	8	2227	2455.516992	2533.469567	2832.109959	34876	16493.53362	10040.73601	0	0	3.866955893	3.921779516	0	32.4327025	11.79084418	27.44243884
TCGA-VS-A9V2	1	27	2311	2473.885033	2621.709606	2827.48686	36213	16162.90889	10459.38597	0	0	0	3.921779516	0.92461964	33.15386356	12.25671674	28.17945213
TCGA-FU-A2QG	1	607	2181	2067.854664	2459.936201	2301.378285	30983	13716.09255	9775.035444	0	0	1.933477946	0	1.849239281	31.16821966	14.93721811	26.90592879
TCGA-VS-A9U7	1	1203	2202	2296.005061	2624.650941	2841.356155	35679	14761.13738	10046.61868	0	0	4.833694866	0	0.92461964	32.54301547	12.50718686	28.10264631
TCGA-C5-A2LY	1	954	2078	2190.630513	2316.791249	2730.401798	35245	16453.89732	8992.64043	0	0	1.933477946	3.921779516	0.92461964	33.23481428	12.98721631	26.75662146
TCGA-EA-A44S	1	3442	2090	2174.195951	2455.033977	2753.517289	31075	15428.18727	9653.460279	0	0	2.90021692	7.843559032	0.92461964	31.92937332	12.92343469	28.44025512
TCGA-4J-AA1J	1	747	2443	2498.053507	2769.756783	2955.084371	37116	16667.54664	11039.80934	0	0	0	3.921779516	0	27.14530849	9.60784202	34.21711052
TCGA-C5-A1BI	1	803	2174	2376.244396	2535.430457	2952.310512	34244	16379.45842	9990.733317	0	0	4.833694866	7.843559032	0	33.09910184	12.1053141	28.28115737
TCGA-FU-A5XV	1	2496	2462	1987.615329	2627.592276	2557.497925	36036	12802.52422	10384.87216	0	0	1.933477946	7.843559032	1.849239281	31.87702226	11.97996963	28.69888763
TCGA-EX-A3L1	1	27	2696	2754.239335	2942.315082	3254.661134	40591	18020.01446	11722.19897	0	0	2.90021692	3.921779516	2.773858921	29.75013669	8.25914556	30.75505468
TCGA-VS-A9UC	1	1367	2264	2540.590022	2733.480323	2972.652144	33995	17761.89516	10563.31313	0	0	3.866955893	3.921779516	2.773858921	31.44545543	12.94355405	29.38687861
TCGA-C5-A1BL	1	2669	2134	2173.229212	2504.056221	3024.430844	33856	12910.79899	9757.387436	0	0	4.833694866	0	1.849239281	32.89790901	13.25998053	26.45197765
TCGA-EK-A2RC	1	103	2159	2115.224873	2585.433146	2812.692946	35592	15148.79971	10261.3361	0	0	1.933477946	0	1.849239281	32.7092618	12.54130103	29.44700006
TCGA-MA-AA41	1	1415	2276	2116.191612	2641.318504	2690.643154	33935	13084.812	10056.42312	0	0	1.933477946	0	0.92461964	31.74764131	13.23347534	28.22639492
TCGA-ZJ-AAXN	1	3988	2147	2116.191612	2574.648252	2772.009682	33046	13933.60882	10130.93693	0	0	4.833694866	3.921779516	1.849239281	29.2209327	15.15682212	29.83625946
TCGA-C5-A1MP	1	652	2421	2322.107014	2649.162063	2769.235823	41081	16276.98409	10400.55928	0	0	0	3.921779516	0.92461964	32.41320077	10.7060203	29.97655822
TCGA-RA-A741	1	828	2477	2290.204628	2476.603764	2665.678423	38005	16110.70499	9809.351014	0	0	0	0	0	33.32960046	12.18143922	27.97270065
TCGA-C5-A1BJ	1	986	2319	2295.038322	2500.134441	2768.311203	36250	15553.86334	9697.580298	0	0	1.933477946	7.843559032	0	32.40929731	11.17189762	29.67567896
TCGA-ZJ-AAXD	1	940	2363	2467.11786	2624.650941	2859.848548	39326	16234.44758	10213.2943	0	0	1.933477946	0	2.773858921	32.1456771	10.43881404	32.65452534
TCGA-C5-A902	1	1667	2135	1988.582068	2146.19384	2672.150761	31763	13398.03543	8188.675629	0	0	0	0	0	34.50771631	16.18073318	26.45427862
TCGA-LP-A4AU	1	43	2066	2405.246565	2408.953068	2790.502075	31156	16672.38033	9184.807626	0	0	1.933477946	7.843559032	0	31.67354537	13.09201111	29.99062987
TCGA-JW-A5VG	1	383	1889	2274.736804	2288.358348	2557.497925	31100	15765.57918	9115.19604	0	0	1.933477946	7.843559032	1.849239281	32.02385653	14.76268519	25.68657625
TCGA-FU-A3YQ	1	632	2114	2371.410701	2623.670496	2857.074689	32968	15525.82791	10825.09191	0	0	2.90021692	7.843559032	0.92461964	32.9422711	11.1562859	28.54852531
TCGA-EA-A3HS	1	1103	2371	2513.52133	2316.791249	2922.722683	35278	15498.75922	9077.939135	0	0	3.866955893	2.941334637	1.849239281	31.03619608	12.42984073	29.66192166
TCGA-C5-A1MQ	1	954	2454	2374.310918	2715.832315	2662.904564	34888	15504.55965	10291.72989	0	0	2.90021692	0	3.698478562	33.76061185	11.18855693	27.5061572
TCGA-EK-A2PL	1	621	1927	1945.078814	2518.762894	2658.281466	29448	12008.83153	9670.127842	0	0	2.90021692	3.921779516	0.92461964	32.47172567	14.56256506	27.53848444
TCGA-C5-A8YT	1	45	2019	1810.702097	2439.346859	2797.899032	33679	10733.70282	9549.533121	0	0	4.833694866	1.960889758	2.773858921	29.64538147	15.90339355	32.07579391
TCGA-VS-A8EG	1	18	2271	2312.439624	2450.131753	2758.140387	39022	15537.42878	9601.4967	0	0	2.90021692	3.921779516	2.773858921	33.04200038	11.71371141	30.96190538
TCGA-C5-A2LV	2	799	2205	2271.836587	2533.469567	2795.125173	31750	14607.42589	9945.632853	0	0	2.90021692	0	0.92461964	30.26941198	10.86132442	29.45322725
TCGA-C5-A1BK	2	1847	2087	2263.135936	2470.721095	2789.577455	29146	15925.09111	9725.032755	0	0	6.767172813	0	0	34.11522991	12.56667215	26.74457668
TCGA-Q1-A73O	2	2056	2213	2043.686189	2714.85187	2656.432227	35352	15172.96819	11023.14177	0	0	0	7.843559032	0	32.83511598	14.47158682	26.94382538
TCGA-JW-A5VL	2	0	2317	2127.79248	2528.567343	2981.89834	31087	12622.71077	9969.16353	0	0	1.933477946	3.921779516	0.92461964	32.2629079	13.57474898	27.34155187
TCGA-JX-A5QV	2	81	2258	2431.348518	2318.752139	2761.838866	37634	17536.64497	8874.0066	0	0	1.933477946	0	0.92461964	31.84993942	13.18372702	26.63275646
TCGA-C5-A1M7	2	57	2349	2274.736804	2526.606453	2907.004149	33808	14675.09761	9992.694207	0	0	1.933477946	7.843559032	0.92461964	33.11035206	9.58197506	29.44003469
TCGA-IR-A3L7	2	1551	2289	2260.235719	2618.768272	2919.024205	35396	15243.54013	10658.41628	0	0	3.866955893	3.921779516	2.773858921	27.42942599	10.54119341	39.28542376
TCGA-C5-A905	2	854	2440	2641.130875	2475.623319	2826.562241	34458	15480.39118	9884.84527	0	0	3.866955893	3.921779516	0.92461964	32.84330133	10.70096349	32.22740206
TCGA-EA-A97N	2	202	2589	2443.916124	2605.042044	3220.450207	39928	18362.24006	10230.94231	0	0	6.767172813	3.921779516	2.773858921	28.47465538	8.893505622	34.53961605
TCGA-VS-A8EI	2	524	2503	2223.499638	2607.002933	2721.155602	37082	15122.69776	9714.247861	0	0	1.933477946	7.843559032	0	33.40722008	12.59827122	28.38645148
TCGA-HG-A2PA	2	555	2264	2311.472885	2834.466145	3030.903181	33354	16595.04121	11069.22268	0	0	1.933477946	3.921779516	0	31.74036296	12.79010339	29.50965826
TCGA-VS-A94Z	2	579	1917	2026.284888	2380.520166	2901.456432	29667	13305.22849	9604.438035	0	0	0	0	0	34.39027332	13.67720067	26.9393107
TCGA-VS-A8EL	2	1472	1987	2279.570499	2617.787827	2640.713693	30168	15736.57701	10483.89709	0	0	1.933477946	7.843559032	0.92461964	32.29792962	12.93489444	27.1118619
TCGA-C5-A2LT	2	2383	2389	2174.195951	2405.031288	2758.140387	38211	12970.7368	9633.851381	0	0	1.933477946	7.843559032	0	31.82672761	12.82390351	28.40803351
TCGA-EA-A5O9	2	369	2106	2233.167028	2254.042777	2631.467497	33099	15769.44613	8945.579076	0	0	1.933477946	3.921779516	0.92461964	31.41980531	13.21721481	28.80405231
TCGA-VS-A94W	2	542	2178	2300.838756	2674.65363	2778.482019	30104	14785.30586	10741.75409	0	0	2.90021692	3.921779516	1.849239281	33.31492389	11.25472809	27.97175901
TCGA-LP-A5U3	2	1112	2140	2180.963124	2413.855292	2738.723375	32258	13508.24367	9759.348326	0	0	0.966738973	0	0.92461964	31.28513252	13.29862475	27.11831269
TCGA-EK-A2RM	2	321	2709	2583.126537	2808.974578	3139.083679	37485	18640.66088	11137.85383	0	0	4.833694866	3.921779516	0.92461964	26.86775976	7.362703332	34.24972903
TCGA-EA-A5ZD	2	825	2531	2302.772234	2788.385236	3018.883126	38241	14252.63268	10637.82694	0	0	1.933477946	3.921779516	0	30.47898046	9.935049391	30.59958621
TCGA-EA-A3Y4	2	5271	2102	1995.349241	2445.229528	2651.809129	27000	14401.51048	9921.121731	0	0	1.933477946	3.921779516	0.92461964	33.47148572	14.77642416	25.57758388
TCGA-IR-A3LC	2	129	2225	2229.300072	2447.190418	2916.250346	36654	15209.70427	9370.111709	0	0	2.90021692	0	0.92461964	29.92863752	13.1411537	30.0836358
TCGA-EX-A69L	2	279	2415	2502.887202	2547.195796	2951.385892	34374	14737.93565	9774.054999	0	0	4.833694866	1.960889758	0.92461964	33.48929347	12.14111061	26.97683605
TCGA-C5-A8ZZ	2	0	2788	2751.339118	2872.703495	3263.907331	43890	18523.68547	11275.11611	0	0	6.767172813	3.921779516	3.698478562	27.69910731	5.951282539	38.21838372
TCGA-XS-A8TJ	2	109	2183	2323.073753	2410.913957	2847.828492	32793	16691.71511	9894.649719	0	0	1.933477946	0	0.92461964	30.52313069	11.03020526	30.74638387
TCGA-JW-A852	2	444	2368	2196.430947	2688.379858	2715.607884	35435	14337.70571	10743.71498	0	0	3.866955893	7.843559032	1.849239281	33.47338199	11.99144424	28.98472772
TCGA-VS-A9UU	2	4385	2245	2401.37961	2371.696162	2800.672891	30674	14349.30658	9344.620142	0	0	2.90021692	3.921779516	2.773858921	34.35938696	12.28588547	28.16638861
TCGA-R2-A69V	2	149	2292	2294.071584	2354.048154	2848.753112	31494	14063.15184	9322.06991	0	0	2.90021692	0	2.773858921	33.04467556	11.97804536	26.81446518
TCGA-MY-A5BE	2	343	2370	2121.992046	2436.405524	2872.793223	33765	13428.97108	9499.530433	0	0	6.767172813	0	0.92461964	34.95327206	12.93776051	25.81199891
TCGA-VS-A950	2	834	2545	2302.772234	2810.935468	3085.45574	37787	15098.52928	11123.14715	0	0	0	3.921779516	1.849239281	32.27059809	9.950473634	30.5798252
TCGA-VS-A8QF	2	861	2289	2315.339841	2721.714984	2821.014523	31291	17209.8872	10775.08922	0	0	1.933477946	7.843559032	1.849239281	33.32518737	10.54157562	28.59036616
TCGA-ZJ-AAXJ	2	959	2247	2239.934201	2560.922024	2926.421162	37533	16153.2415	9752.485211	0	0	1.933477946	7.843559032	2.773858921	33.82767781	12.43012242	27.48023197
TCGA-EK-A2PK	2	1031	2184	2222.532899	2558.961134	2616.673582	33517	15909.62328	10036.81423	0	0	1.933477946	3.921779516	0.92461964	33.75510124	13.98674027	26.86543672
TCGA-PN-A8MA	2	13	2304	2093.956616	2600.139819	2958.782849	34482	14793.03977	10052.50134	0	0	1.933477946	0	0.92461964	32.79152924	12.4811243	31.06470086
TCGA-FU-A3WB	2	1386	2526	2314.373102	2535.430457	2661.979945	39785	15026.9906	10299.57345	0	0	0	0	0.92461964	30.73080634	12.94966182	33.57861208
TCGA-MA-AA3Z	2	2234	2284	2115.224873	2410.913957	2611.125864	35359	16036.26609	9269.125886	0	0	1.933477946	3.921779516	0.92461964	33.50495121	14.74788316	25.57678523
TCGA-EA-A43B	2	5385	2157	2087.189443	2469.74065	3102.098893	32263	14957.38539	9399.525055	0	0	1.933477946	0	2.773858921	32.78754545	12.12809892	27.87768888
TCGA-C5-A1M8	2	428	1895	2573.459147	2699.164752	2721.155602	32623	15700.80766	10694.69274	0	0	2.90021692	3.921779516	0	29.67485673	10.06458541	33.2602726
TCGA-EA-A3HU	2	474	2195	1976.9812	2489.349548	2556.573306	33849	11826.11786	9729.934979	0	0	1.933477946	3.921779516	0.92461964	34.17258383	14.01467867	26.31758126
TCGA-C5-A7CH	2	636	2532	2730.07086	3073.694696	3103.023513	41181	20021.16414	12649.69983	0	0	3.866955893	7.843559032	1.849239281	27.79963529	8.641608847	32.6336826
TCGA-EX-A8YF	2	1409	2147	2256.368764	2341.302371	2806.220609	34188	15811.01591	8726.939868	0	0	4.833694866	7.843559032	2.773858921	34.21497129	13.35677999	26.70926514
TCGA-Q1-A6DW	2	11	2473	2104.590745	2665.829626	2960.632089	39787	14777.57195	10424.08995	0	0	4.833694866	0	0.92461964	31.30151859	13.13594888	30.32397069
TCGA-C5-A3HE	2	729	2139	2290.204628	2546.215351	2750.74343	33684	15721.10918	9962.300416	0	0	1.933477946	3.921779516	2.773858921	33.05487743	13.09006002	27.69119695
TCGA-EK-A2PI	2	773	2470	2481.618944	2737.402102	3026.280083	38590	14244.89877	10791.75678	0	0	1.933477946	7.843559032	0	27.40873944	11.43469531	36.55509315
TCGA-VS-A8QA	2	1015	2180	2127.79248	2226.59032	2427.126556	33739	13471.50759	8927.931068	0	0	0	0	2.773858921	33.78379712	15.58063066	26.23834162
TCGA-EA-A50E	2	1992	2678	2625.663051	2873.68394	2791.426694	37794	16465.49819	11175.11073	0	0	4.833694866	3.921779516	0	28.16761252	9.031360927	33.37801333
TCGA-EK-A2R7	2	788	2212	2427.481562	2497.193107	2686.020055	36404	17822.79971	9864.255928	0	0	4.833694866	7.843559032	0	33.69960623	12.31738787	29.00965027
TCGA-FU-A3NI	2	1243	2218	2369.477223	2549.156685	2808.069848	31990	15562.56399	10126.03471	0	0	4.833694866	0	0.92461964	30.80982479	11.1464883	31.14207551
TCGA-EK-A2RN	2	25	2619	2383.978308	2720.734539	2793.275934	45109	14100.85466	10782.93278	0	0	0	3.921779516	0.92461964	31.15054512	10.18514238	29.32745516
TCGA-LP-A4AX	2	50	1863	2102.657267	2571.706918	2925.496542	24761	15792.64787	10144.66316	0	0	3.866955893	3.921779516	1.849239281	33.12914712	13.25083255	27.11126448
TCGA-JW-A5VI	2	830	2205	2278.60376	2568.765583	2798.823651	36084	16941.13377	10373.10682	0	0	0	7.843559032	0	32.49884068	12.75928629	29.55090954
TCGA-EA-A3QE	2	1122	2227	2115.224873	2415.816182	2865.396266	32611	12698.11641	9440.70374	0	0	1.933477946	0	2.773858921	34.53875422	13.55213985	25.91320461
TCGA-C5-A7UE	2	3935	2404	2466.151121	2520.723784	2702.663209	37066	17437.07086	9784.839892	0	0	4.833694866	0	0	34.22119207	11.20434613	29.17649539
TCGA-Q1-A73Q	2	602	2517	2380.111352	2767.795893	2938.441217	39120	15756.87852	10745.67587	0	0	1.933477946	7.843559032	2.773858921	33.38281248	9.015911022	31.41001987
TCGA-JW-AAVH	2	636	2042	1846.471439	2240.316549	2466.885201	30843	11640.50398	8429.86507	0	0	2.90021692	7.843559032	0.92461964	32.96878829	16.62538528	29.99224588
TCGA-MU-A8JM	2	890	2209	2109.42444	2566.804693	2836.733057	34868	15946.35936	10122.11293	0	0	0	0	0.92461964	32.37792447	12.52782623	28.57783044
TCGA-DS-A3LQ	2	156	2502	2590.860448	2712.89098	3122.440526	38441	18178.55965	10775.08922	0	0	1.933477946	0	1.849239281	29.65488597	8.742917424	32.64909915
TCGA-EA-A1QS	2	442	2462	2623.729573	2714.85187	2874.642462	37907	18240.43095	10764.30433	0	0	3.866955893	0	0.92461964	28.82471102	9.235196564	35.19153575
TCGA-VS-A958	2	596	2044	2213.832249	2282.475678	2926.421162	30475	14465.31526	8713.21364	0	0	3.866955893	3.921779516	2.773858921	33.77140796	13.13682797	25.72332417
TCGA-DG-A2KM	2	1066	2246	2077.522054	2406.992178	2522.362379	34076	12283.38539	9498.549988	0	0	0	0	0	34.17214943	12.84376595	25.39314002
TCGA-EK-A3GN	2	1800	2462	2754.239335	2698.184307	3004.089212	38023	17612.05061	10796.65901	0	0	1.933477946	3.921779516	1.849239281	30.11264529	9.275004858	35.36878015
TCGA-DS-A1OC	2	0	2144	2190.630513	2646.220728	2959.707469	34260	14924.51627	10452.52286	0	0	4.833694866	0	0.92461964	31.92382937	12.63209095	27.78521344
TCGA-C5-A907	2	12	2341	2400.412871	2528.567343	2803.44675	37432	16809.65727	9914.258616	0	0	4.833694866	0	2.773858921	30.29701773	14.0387666	28.62217443
TCGA-FU-A3HY	2	491	2578	2296.005061	2519.743339	2963.405947	39975	13978.07881	9886.80616	0	0	1.933477946	3.921779516	0.92461964	29.25734373	11.92705902	30.52843962
TCGA-EA-A1QT	2	595	2224	2426.514823	2257.964556	2093.338866	34104	14658.66305	8890.674163	0	0	4.833694866	3.921779516	0	30.76373986	11.44599945	30.11679464
TCGA-DS-A0VN	2	791	2276	2465.184382	2022.657785	2173.780775	35543	16124.23933	7972.977756	0	0	1.933477946	0	0	31.07739488	12.93822931	28.54172209
TCGA-ZJ-AB0H	2	919	2225	2564.758496	2422.679296	2443.76971	34173	16583.44035	9183.827182	0	0	3.866955893	3.921779516	0	33.1271483	10.86552682	28.56761386
TCGA-EK-A2RB	2	1013	2314	2353.042661	1957.948423	2245.901107	34116	14380.24223	7220.976534	0	0	1.933477946	3.921779516	0	31.97662164	14.46547719	29.10928433
TCGA-EK-A2R8	2	4694	2377	2611.161967	2275.612564	2367.950899	38882	16567.00578	9048.525788	0	0	6.767172813	0	0.92461964	29.76557375	12.88793459	29.12241327
TCGA-MA-AA43	2	473	2597	2748.438901	2594.25715	2350.383126	35860	18407.67679	10593.70692	0	0	4.833694866	0	0	29.51545969	7.98467648	33.02411517
TCGA-MA-AA3Y	2	534	2593	2496.120029	2306.006355	2087.791148	37405	16538.97035	8977.933757	0	0	6.767172813	0	0.92461964	32.14520412	11.97260783	27.86946642
TCGA-BI-A0VS	2	548	2179	2535.756327	2348.165485	2367.950899	33010	16922.76573	9112.254705	0	0	3.866955893	0	0	32.41137775	10.62897027	28.99483974
TCGA-EK-A2IR	2	1099	3201	3117.733189	3151.149841	3285.173582	46416	21164.81634	12770.29455	0	0	6.767172813	7.843559032	2.773858921	23.29121455	2.778127164	46.42190849
TCGA-BI-A20A	2	44	2033	2671.099783	2115.800049	2322.644537	32310	16617.27621	7921.014177	0	0	1.933477946	3.921779516	0.92461964	33.30244217	11.6365437	28.93448773
TCGA-EA-A6QX	2	380	2055	2430.381779	2245.218773	2151.589903	34118	17091.94505	8858.319482	0	0	4.833694866	0	0.92461964	33.80528361	11.96110144	28.25071736
TCGA-C5-A1MF	2	747	2365	2510.621114	2379.539721	1989.781466	37252	15594.46638	9574.044243	0	0	4.833694866	0	0	33.58811979	12.47245964	27.48216562
TCGA-IR-A3LH	2	761	2176	2314.373102	2442.288194	2281.961272	31809	12124.8402	9360.30726	0	0	4.833694866	2.941334637	0	32.87552838	11.73441675	27.2680393
TCGA-DS-A1OD	2	4738	2123	2411.046999	2219.727206	2426.201936	32031	14868.44541	8437.708629	0	0	1.933477946	0	0	34.61793618	13.54021601	26.41745946
TCGA-MA-AA3X	2	284	2581	2719.436732	2499.153997	2381.820194	39378	18994.48735	10058.38401	0	0	4.833694866	3.921779516	0	29.53895215	9.082128141	30.66794605
TCGA-EA-A411	2	552	2398	2835.445409	2720.734539	2568.593361	32984	19679.90528	10677.04473	0	0	1.933477946	7.843559032	0.92461964	30.45403175	8.719002282	33.85765198
TCGA-EA-A439	2	699	2403	2497.086768	2194.235639	2451.166667	40526	17045.54158	8348.488145	0	0	2.90021692	3.921779516	0	28.20380304	12.46962343	33.17733877
TCGA-DG-A2KJ	2	1525	2306	2195.464208	2331.497922	2451.166667	36057	13928.77513	9000.483989	0	0	1.933477946	3.921779516	0.92461964	34.78947417	12.44013592	27.70472398
TCGA-EK-A3GJ	2	1946	2172	2299.872017	2095.210706	2124.775934	30698	14654.7961	7959.251528	0	0	4.833694866	0	0.92461964	31.49867087	13.47318065	26.25106292
TCGA-ZJ-AAXU	2	27	2015	2504.82068	2278.553899	2112.755878	33198	16201.57845	8805.375458	0	0	3.866955893	0	0.92461964	36.35008692	12.97150854	26.15729628
TCGA-EK-A2RJ	2	376	2310	2571.525669	2236.394769	2253.298064	34128	17685.52278	8595.560254	0	0	4.833694866	3.921779516	0.92461964	32.34228063	13.39659399	27.07599904
TCGA-HM-A4S6	2	448	2192	2193.53073	2436.405524	2071.147994	38023	12799.62401	9331.874358	0	0	2.90021692	7.843559032	0	31.30071286	12.78009906	28.57802702
TCGA-EA-A410	2	1243	2399	2652.731743	2082.464923	2415.106501	36945	17526.97758	8237.697873	0	0	3.866955893	3.921779516	0.92461964	29.07152102	11.90985435	31.63060067
TCGA-C5-A3HD	2	3609	2271	2283.437455	2249.140552	2498.322268	32453	13721.89299	8266.130775	0	0	3.866955893	0	0.92461964	30.12736791	13.47235832	28.10175811
TCGA-DG-A2KK	2	0	2227	2547.357195	2260.905891	2403.086445	32338	15142.03254	8488.691762	0	0	2.90021692	7.843559032	0	31.62332533	12.2572522	28.67731593
TCGA-DS-A0VM	2	9	2568	2238.967462	2407.972623	2531.608575	37523	12185.74476	9299.519677	0	0	0	3.921779516	0	29.36268233	11.46612833	31.37138412
TCGA-LP-A4AW	2	44	2326	2666.266088	2605.042044	2298.604426	38017	18269.43312	10486.83843	0	0	6.767172813	0	0	30.23718192	8.925840251	35.11544035
TCGA-VS-A9U6	2	346	1931	2351.109183	2171.685407	2210.76556	25195	13743.16124	8353.390369	0	0	1.933477946	3.921779516	0	31.83315919	12.30833286	29.53878667
TCGA-DG-A2KL	2	542	2447	2607.295011	2445.229528	2440.995851	33839	17777.36298	9628.949157	0	0	4.833694866	7.843559032	0.92461964	28.85063747	9.685101565	32.03716827
TCGA-VS-A9UH	2	1735	2662	2556.057845	2269.729895	2234.805671	38924	15379.85033	8823.023466	0	0	2.90021692	0	0	32.78190527	11.6649806	28.2541623
TCGA-BI-A0VR	2	720	2251	2459.383948	2344.243706	2329.116874	33941	17141.24873	9133.824493	0	0	4.833694866	7.843559032	0.92461964	33.24673121	12.5644056	27.90952032
TCGA-DR-A0ZL	2	730	2569	2564.758496	2532.489122	2505.719225	35491	16194.81128	9708.365192	0	0	4.833694866	7.843559032	1.849239281	33.4939372	8.739059885	30.43831469
TCGA-ZJ-AAXT	2	1617	2573	2562.825018	2517.782449	2279.187414	41907	15535.4953	10135.83916	0	0	0	3.921779516	0	31.16313793	11.06790089	28.92595461
TCGA-EA-A78R	2	2394	2258	2580.22632	2502.095331	2080.394191	37699	15977.29501	9717.189196	0	0	4.833694866	0	0	31.36895886	10.82165997	29.78432566
TCGA-MA-AA3W	2	3874	2209	2320.173536	2096.191151	2247.750346	35816	13039.37527	7778.84967	0	0	1.933477946	0	0.92461964	33.54140647	12.76965422	26.92607983
TCGA-EA-A5ZE	2	617	2376	2660.465654	2373.657052	2367.026279	31396	16169.67607	9117.15693	0	0	3.866955893	0	0	31.95883753	11.39401027	30.71948773
TCGA-VS-A9UB	2	965	2142	2420.714389	2192.274749	2152.514523	36849	16510.93492	8570.068687	0	0	2.90021692	3.921779516	1.849239281	32.79686145	12.88751207	25.35791564
TCGA-C5-A2LX	2	2893	2358	2485.4859	2324.634808	2220.011757	32286	17675.85539	8917.146175	0	0	6.767172813	0	0	34.62157997	12.44418144	26.56876229
TCGA-Q1-A6DT	2	3	2561	2738.771511	2720.734539	2490.000692	40359	18574.92263	10815.28746	0	0	4.833694866	7.843559032	0	29.49483075	8.910886977	31.43124535
TCGA-FU-A3TQ	2	5	2457	2430.381779	2342.282816	2409.558783	37474	15181.66884	9258.340992	0	0	1.933477946	0	0	32.38060968	10.75019718	29.86224097
TCGA-VS-A8EC	2	53	2533	2752.305857	2544.254461	2551.025588	36923	18351.60593	10532.91934	0	0	4.833694866	2.941334637	1.849239281	26.16058949	9.591135468	36.72790822
TCGA-C5-A7UH	2	454	2261	2511.587852	2182.470301	2342.061549	35187	18111.85466	8411.236617	0	0	4.833694866	3.921779516	0.92461964	31.49693944	11.22065683	29.87836703
TCGA-C5-A7CG	2	1582	2320	2592.793926	2302.084576	1948.173582	35336	15002.82213	8679.878514	0	0	2.90021692	3.921779516	0.92461964	34.50568019	13.29535754	26.08134148
TCGA-JW-A5VJ	2	3589	2081	2565.725235	2464.838426	2372.573997	29645	16403.6269	9582.868247	0	0	0	3.921779516	0.92461964	32.42653136	10.12982778	29.71625946
TCGA-EA-A5ZF	2	1320	2945	3023.959508	2900.155952	2751.66805	42847	21108.74548	11327.07969	0	0	0	3.921779516	0.92461964	25.53945162	6.130061973	38.45543357
TCGA-VS-A8EH	2	1427	2255	2404.279826	2357.969934	2223.710235	32342	14611.29284	9010.288438	0	0	2.90021692	3.921779516	0	30.5166704	13.20554827	30.68493078
TCGA-EA-A3HR	2	1505	2466	2485.4859	2307.967245	2459.488243	38885	16284.718	8698.506967	0	0	0	2.941334637	0.92461964	30.06101815	11.6174413	32.43122065
TCGA-VS-A9U5	2	0	2334	2522.221981	2070.699584	2120.152835	35230	16078.8026	8100.43559	0	0	1.933477946	3.921779516	1.849239281	34.5942268	12.84307149	26.65153474
TCGA-ZJ-AAX8	2	410	2500	2351.109183	2332.478367	2319.870678	42588	16404.59364	8647.523833	0	0	1.933477946	0	0.92461964	32.38652438	11.79284558	29.70408452
TCGA-EK-A2R9	2	685	2302	2396.545915	2226.59032	2388.292531	36099	15404.98554	8811.258128	0	0	4.833694866	0	1.849239281	31.35161558	12.55227898	30.10467142
TCGA-EX-A1H5	2	829	2454	2098.790311	2451.112198	2137.720609	36747	11883.15546	9512.276216	0	0	6.767172813	3.921779516	0	32.88558934	12.04360685	31.88186735
TCGA-EK-A2RO	2	911	2651	2392.678959	2614.846492	2391.06639	38766	16450.03037	10479.97531	0	0	6.767172813	0	0	27.85724105	12.68268262	32.65740998
TCGA-EA-A3QD	2	2526	2571	2207.065076	2286.397458	2465.035961	38282	13491.80911	9168.140064	0	0	2.90021692	3.921779516	0	33.80883748	12.00747943	27.65740625
TCGA-FU-A23L	2	795	2666	2235.100506	2689.360303	2335.589212	38054	16308.88648	10520.17355	0	0	1.933477946	0	2.773858921	33.15780195	13.44540856	27.35384537
TCGA-MU-A5YI	2	6408	2599	2615.028923	2470.721095	2196.896266	38089	16488.69993	9385.798827	0	0	4.833694866	7.843559032	0	30.68930838	11.00267243	33.30962144
TCGA-IR-A3LL	2	1535	2235	2065.921186	2454.053532	2004.57538	32215	14606.45915	9391.681496	0	0	1.933477946	3.921779516	2.773858921	33.00425037	13.50512704	25.8550914
TCGA-EA-A3HQ	2	0	2538	2346.275488	2811.915913	2490.000692	38425	16693.64859	10931.9604	0	0	4.833694866	0	0	30.57663337	10.59747582	30.15535817
TCGA-DR-A0ZM	2	4	2542	1816.502531	2520.723784	2234.805671	38416	12222.48084	9867.197262	0	0	1.933477946	3.921779516	0	33.38791958	13.34822076	27.12634152
TCGA-ZJ-AAXF	2	619	2484	2288.27115	2522.684674	1984.233748	36982	17177.01808	9912.297727	0	0	6.767172813	5.882669274	0.92461964	34.35241917	11.58517974	28.92773764
TCGA-EX-A69M	2	2	2274	2194.497469	2223.648986	2214.464039	34681	15051.15907	8504.37888	0	0	1.933477946	3.921779516	0	32.773634	13.12666276	26.80627086
TCGA-VS-A9V3	2	397	2585	2123.925524	2355.028599	2268.091978	39458	9853.970354	9542.670007	0	0	0	3.921779516	0.92461964	33.52480866	11.57165662	27.95363469
TCGA-MY-A5BD	2	725	2354	2251.535069	2194.235639	2055.429461	34286	13265.59219	8492.613542	0	0	4.833694866	3.921779516	0	32.52642503	14.68846707	28.89406204
TCGA-LP-A4AV	2	1053	2371	2352.075922	2440.327304	2414.181881	34521	15497.79248	9549.533121	0	0	2.90021692	7.843559032	0.92461964	33.45985569	12.11677195	28.21328463
TCGA-JX-A3Q0	2	1106	2653	2126.825741	2278.553899	2318.946058	36134	11236.40709	8724.978978	0	0	0	7.843559032	1.849239281	32.96751693	12.06082345	27.26081579
TCGA-ZJ-AAXA	2	1136	3024	2731.037599	3083.499144	2832.109959	44675	18110.88792	12577.14691	0	0	6.767172813	3.921779516	3.698478562	26.99797372	6.162985519	37.18187385
TCGA-ZX-AA5X	2	1791	2702	2224.466377	2756.030555	2415.106501	36291	14783.37238	10792.73723	0	0	1.933477946	5.882669274	0.92461964	31.03012467	10.26233365	31.8115422
TCGA-EK-A2GZ	2	0	2549	1956.679682	2283.456123	2302.302905	37884	11773.91396	8665.171841	0	0	1.933477946	0	0	32.39116141	13.64153352	26.7520388
TCGA-HM-A3JK	2	186	2502	2542.5235	2483.466879	2433.598893	36736	17703.89082	9597.574921	0	0	0	3.921779516	0.92461964	31.18118178	9.952627604	33.13470301
TCGA-FU-A3HZ	2	540	2481	1959.579899	2374.637497	2103.509682	40600	13889.13883	9728.954534	0	0	0	3.921779516	0	30.41617621	13.55744154	29.48092768
TCGA-VS-A957	2	0	2740	2304.705712	2647.201173	2268.091978	39845	17432.23717	10318.20191	0	0	4.833694866	3.921779516	0.92461964	30.91707598	13.38105357	28.63391841
TCGA-ZJ-AAXI	2	6375	2452	2268.93637	2169.724517	2090.565007	38544	14959.31887	8362.214373	0	0	4.833694866	0	0.92461964	32.62495674	13.76277449	28.0104221
TCGA-EK-A2RK	2	119	2477	2286.337672	2453.073087	2082.24343	35744	15513.2603	9434.821071	0	0	0	3.921779516	0	31.83590125	13.02544326	28.43489715
TCGA-VS-A954	2	1688	2776	2304.705712	2648.181618	2305.076763	41616	13573.98192	10310.35835	0	0	6.767172813	0	0.92461964	32.19585521	11.27043104	29.86240049
TCGA-MY-A5BF	2	0	2599	2376.244396	2847.211929	2448.392808	39329	15599.30007	11487.87265	0	0	6.767172813	7.843559032	0.92461964	31.01686129	9.475001516	34.12572379
TCGA-EA-A3HT	2	13	2635	2563.791757	2720.734539	2336.513831	39055	17497.97542	11141.7756	0	0	4.833694866	0	0.92461964	29.48063027	9.6430898	32.19438322
TCGA-C5-A7CO	2	1714	2455	2310.506146	2565.824248	2192.273167	32330	16231.54736	10050.54045	0	0	1.933477946	0	0	32.52802709	13.0960407	29.6824521
TCGA-C5-A8XI	2	4482	2459	2082.355748	2588.374481	2018.444675	34402	12620.7773	9890.727939	0	0	2.90021692	3.921779516	0	34.17336294	13.19300112	27.88475138
TCGA-IR-A3LK	2	254	2689	2172.262473	2671.712295	2216.313278	34790	14443.08026	10170.15473	0	0	1.933477946	0	0.92461964	30.87195433	12.29663263	27.23782872
TCGA-VS-A9UD	2	808	2630	2023.384671	2401.109509	2126.625173	36362	11835.78525	9330.893913	0	0	1.933477946	7.843559032	0	33.92218771	12.84747604	25.36685321
TCGA-EK-A2RA	2	739	2571	2183.863341	2440.327304	1859.410097	39019	12575.34056	9308.343681	0	0	0	7.843559032	0	33.56739584	13.89927536	25.41121682
TCGA-C5-A8XJ	2	1246	2650	2630.496746	2575.628697	2351.307746	38309	16668.51338	10224.0792	0	0	1.933477946	3.921779516	1.849239281	31.1583291	12.36061092	32.94506338
TCGA-MA-AA42	2	4467	2291	2187.730296	2321.693473	2132.172891	32829	13184.38612	8974.011978	0	0	2.90021692	0	0	34.77351053	12.93296635	25.28059994
TCGA-DS-A1O9	2	259	2488	2343.375271	2548.176241	2027.690871	34475	15785.88069	10032.89245	0	0	4.833694866	3.921779516	0.92461964	30.4009783	12.14448747	29.04917793
TCGA-C5-A3HL	2	266	2575	2001.149675	2573.667807	2309.699862	36932	13265.59219	10220.15742	0	0	1.933477946	7.843559032	0	31.55424748	12.95932731	29.23767682

**TCGA-VS-A9UI**	2	1246	2284	2275.703543	2427.58152	2188.574689	30231	14874.24584	9582.868247	0	0	0	3.921779516	0	33.41414328	12.65640915	26.40934741
**TCGA-VS-A959**	2	1561	2407	2367.543745	2567.785138	2305.076763	36702	16018.86479	9909.356392	0	0	0	0	1.849239281	34.39283576	11.20608428	29.09861877
**TCGA-DS-A1OA**	2	879	2481	2228.333333	2498.173552	2246.825726	38816	15574.16486	9994.655097	0	0	6.767172813	3.921779516	0	32.40921525	11.88473755	29.00356337
**TCGA-FU-A3TX**	2	1630	2504	2315.339841	2693.282083	2310.624481	38317	14339.63919	10514.29088	0	0	1.933477946	7.843559032	0.92461964	31.84795809	12.32382552	28.67281574
**TCGA-VS-A8Q9**	2	512	2263	2246.701374	2451.112198	2126.625173	38637	14474.01591	9499.530433	0	0	4.833694866	0	0	32.03359761	14.04210966	29.48605704
**TCGA-DS-A5RQ**	2	21	2558	2287.304411	2456.994867	2391.99101	38025	15639.90311	9658.362503	0	0	1.933477946	0	0.92461964	31.57633695	11.11202271	29.60882135
**TCGA-EK-A2PM**	3	634	2706	2572.492408	2819.759472	2746.120332	42174	18148.59074	11361.39526	0	0	3.866955893	0	0.92461964	23.30803134	8.178960896	40.03061148
**TCGA-ZJ-AAX4**	4	71	2604	2295.038322	2414.835737	2432.674274	37697	15318.94577	9188.729406	0	0	0	3.921779516	1.849239281	32.11411107	11.46388715	28.51478309

Pancreatic cancer

ID: Patients ID
PFS: Progress-free survival

			Cell population size				2-6Sia levels				Number of reproductive cells produced through multipolar cell division				Supressive	Permissive	Lethal
ID	Best call	PFS	α2-6Sia 1.5	α2-6Sia 1.0	α2-6Sia 0.5	α2-6Sia 0.25	α2-6Sia 1.5	α2-6Sia 1.0	α2-6Sia 0.5	α2-6Sia 0.25	α2-6Sia 1.5	α2-6Sia 1.0	α2-6Sia 0.5	α2-6Sia 0.25	%	%	%
TCGA-IB-AAUU	1	245	3966	4280.076923	4278.570156	4952.843532	277387	170021.25	160732.4749	71111.17454	15	21.57692308	10.27267745	33.86735113	29.89011735	7.429147003	30.01257632
TCGA-2J-AABO	1	440	4148	4265.365385	4550.796109	4807.111294	291324	181840.5	157545.8904	68174.97782	11	19.61538462	5.136338723	27.70965092	31.29081708	8.580841068	31.10844998
TCGA-L1-A7W4	1	164	4208	4192.788462	4450.12387	4519.751951	276949	179931.9231	171153.0789	81257.01191	24	15.69230769	15.40901617	42.07761807	27.99380983	7.178287366	32.73325875
TCGA-M8-A5N4	1	584	4296	3671.019231	4392.596876	5010.3154	303470	196921.7885	163313.9988	77246.29651	31	13.73076923	2.054535489	35.91991786	28.55434189	7.276509973	30.3261032
TCGA-HV-AA8V	1	920	4372	4391.884615	4382.324198	4868.688296	287217	159542.7115	170615.8179	75046.97125	26	25.5	10.27267745	28.73593429	29.85488041	7.896689025	31.29864269
TCGA-HZ-A77Q	1	33	4397	4285.961538	4917.530693	4747.586858	293022	163724.7115	155540.6637	76239.51253	11	0	16.43628391	33.86735113	32.29981541	8.506914758	28.8545926
TCGA-2J-AABI	1	969	4485	4029.980769	4609.35037	4785.559343	277901	167740.9615	155065.0388	70318.88378	22	1.961538462	14.38174842	23.60451745	34.16900693	8.514206874	27.67717548
TCGA-XD-AAUL	1	498	4492	4760.653846	4755.22239	4878.951129	262779	176055.9231	159942.506	70803.28953	18	21.57692308	13.35448068	40.02505133	29.29031119	6.866850609	30.89753108
TCGA-2J-AABP	1	463	4530	4202.596154	4472.72376	4447.912115	276775	178841.3077	152629.387	71877.80821	19	9.807692308	15.40901617	20.52566735	31.31856404	10.10282728	28.92161399
TCGA-YB-A89D	1	350	4554	4459.557692	4306.306385	4741.429158	298325	181633.5577	138126.4209	73013.9039	32	17.65384615	11.29994519	22.57823409	29.83053827	7.539121593	30.86877956
TCGA-HZ-7922	1	4	4648	4092.75	4757.276925	4741.429158	298042	181093.1538	156613.1313	74745.24394	22	19.61538462	13.35448068	34.8936345	31.03534901	7.691443362	30.75071923
TCGA-US-A776	1	1216	4726	4464.461538	4054.625788	4892.292813	284385	180230.0769	154093.2435	66318.43121	29	24.51923077	7.190874212	28.73593429	31.59872486	9.392065036	34.13535068
TCGA-2J-AABH	1	1287	4690	4660.615385	4278.570156	4892.292813	312670	188660.7692	177760.4651	76227.19713	28	34.32692308	11.29994519	49.26160164	29.2315704	6.593129116	31.15891177
TCGA-IB-A6UF	1	666	5428	4160.423077	4563.123321	5141.679671	334552	169605.4038	160758.1566	76240.53881	19	15.69230769	8.218141957	32.84106776	27.24562214	6.607858086	32.72318529
TCGA-Q3-AA2A	1	95	4689	4683.173077	4911.367087	4409.93963	294739	190136.8269	174134.2099	68022.0616	16	34.32692308	26.70896136	26.68336756	29.93939647	8.190302557	30.6098214
TCGA-LB-A8F3	1	379	5379	5243.192308	5205.165662	5074.971253	338426	208523.3077	186485.05	73950.90062	32	19.61538462	42.11797753	42.07761807	27.75848123	7.212174783	32.50262822
TCGA-IB-7644	1	347	4692	5013.692308	4670.986435	4302.179877	295044	203618.4808	161125.9185	65308.56838	21	22.55769231	32.87256783	32.84106776	29.76433425	8.938592974	30.99377121
TCGA-HZ-7925	1	361	5153	4834.211538	5173.320362	4408.913347	323031	190136.8269	177477.9664	64549.11869	13	11.76923077	40.06344204	40.02505133	28.42068484	7.035027232	31.32003336
TCGA-XD-AAUG	1	420	4939	4659.634615	4800.42217	4970.290349	311585	183728.4808	172516.2632	72731.67598	34	11.76923077	34.92710332	34.8936345	30.95842064	7.325939352	32.32457631
TCGA-XN-A8T3	1	951	5202	4789.096154	4609.35037	4229.313758	325537	193669.5577	172178.2921	63209.81889	32	13.73076923	13.35448068	13.34168378	29.76514022	9.857865996	30.74057841
TCGA-HZ-8317	1	16	5325	5301.057692	5737.290354	4461.253799	336881	213906.75	199163.5885	66070.07064	32	14.71153846	29.79076459	29.76221766	27.84439325	7.085873526	32.88866983
TCGA-HZ-A9TJ	1	603	4639	4869.519231	4964.78501	4923.081314	286499	194727.8077	176294.554	74493.80452	15	11.76923077	34.92710332	34.8936345	28.39468683	6.941976906	32.0377688
TCGA-FB-A78T	1	1	5325	5084.307692	5163.047684	4735.271458	331983	203786.1923	181017.9311	71350.29856	21	34.32692308	36.98163881	36.94620123	30.1612677	7.42229371	29.97302007
TCGA-2J-AABE	1	676	4973	4985.25	5325.355988	4818.400411	308775	199621.8462	189088.1465	72289.34784	15	30.40384615	35.95437106	35.91991786	30.18215598	6.462224462	30.7336045
TCGA-OE-A75W	1	110	4727	4522.326923	4262.133872	4101.028337	295958	184255.1538	154245.2791	59774.84846	13	9.807692308	32.87256783	32.84106776	31.15935493	11.16487138	28.30934131
TCGA-3A-A9J0	1	743	4738	4562.538462	4834.322006	4214.945791	297568	192476.9423	171982.084	63314.49979	29	7.846153846	45.19978076	45.15646817	31.53977163	8.035432872	30.30637322
TCGA-F2-A8YN	1	517	5141	5359.903846	5200.029323	4947.712115	323038	215357.3077	178837.0417	76261.06448	25	17.65384615	24.65442587	24.63080082	28.82966266	7.317749781	31.99563229
TCGA-HZ-A49I	1	0	4729	4808.711538	4684.340915	3841.378645	295316	195450.6346	160024.6874	58643.88419	29	13.73076923	9.245409701	9.236550308	32.2603867	7.895347534	28.71070786
TCGA-HZ-A77O	1	11	4704	5046.057692	4774.740477	4509.489117	291427	205962.5192	168359.9379	67096.354	25	11.76923077	10.27267745	10.26283368	29.86086219	7.926743944	29.84888618
TCGA-HZ-7289	1	240	5188	4893.057692	4744.949712	4048.687885	329142	189654.2885	169942.9575	61779.17988	21	34.32692308	22.59989038	22.57823409	29.18694851	10.7833755	30.633683
TCGA-F2-A44G	1	153	5117	5360.884615	5017.175665	5207.361807	316777	212228.6538	180071.8175	78199.71376	27	31.38461538	40.06344204	40.02505133	28.15913426	6.324853767	31.24909183
TCGA-HZ-8638	1	91	4786	4743	4478.887366	3986.0846	305121	189173.7115	158638.9033	59674.27269	26	24.51923077	19.51808715	19.49938398	31.33326693	10.45172072	28.97540494
TCGA-FB-AAQ6	1	NA	4293	4965.634615	4087.498356	4695.246407	274022	199567.9038	147568.0388	70373.2768	14	14.71153846	8.218141957	38.99876797	30.77389813	8.233091931	32.36104526
TCGA-IB-7890	1	NA	5141	5130.403846	4276.515621	4818.400411	326296	199842.5192	154038.7983	74936.13265	19	22.55769231	17.46355166	44.1301848	29.1321963	6.857182651	32.55446916
TCGA-IB-A5SQ	1	NA	4783	4821.461538	4384.378734	4395.571663	297204	196952.1923	156196.0606	67652.59959	41	16.67307692	13.35448068	21.55195072	31.37111198	7.647634835	30.18805256
TCGA-IB-AAUN	1	NA	4513	4543.903846	4282.679227	5276.122793	282875	178785.4038	154877.0488	79213.68172	28	29.42307692	15.40901617	44.1301848	30.89794203	8.055428596	30.37066876
TCGA-FB-AAPU	1	NA	4587	5117.653846	4563.123321	4984.658316	286530	201519.6346	165055.2176	74023.76674	22	33.34615385	19.51808715	35.91991786	32.46975966	7.294868936	31.88030455
TCGA-IB-7893	1	NA	4655	4596.865385	4652.495615	5309.990144	294495	184164.9231	169515.6141	78886.29733	22	5.884615385	16.43628391	42.07761807	28.66110098	7.501073755	31.79046582
TCGA-2J-AABA	1	NA	4678	4810.673077	4209.743217	4838.926078	295966	191565.8077	152571.86	71245.61766	43	28.44230769	16.43628391	36.94620123	32.44776263	8.680938047	31.81724957
TCGA-2J-AABU	1	NA	4539	4774.384615	4227.206769	4775.296509	285558	191470.6731	156165.2425	70574.42834	28	26.48076923	15.40901617	12.31540041	32.13022052	9.677329899	30.27963097
TCGA-2L-AAQA	1	NA	4665	5133.346154	4252.888463	5632.243121	297960	200577.1154	154806.1673	83997.1885	32	19.61538462	11.29994519	46.18275154	28.81920473	7.152442689	31.77570085
TCGA-2L-AAQI	1	NA	4390	4509.576923	3798.836119	4790.69076	277345	174378.8077	136846.4453	72843.54086	18	29.42307692	0	36.94620123	29.49816724	7.86537024	31.57899069
TCGA-2L-AAQL	1	NA	4490	5298.115385	4088.525623	5065.734702	277471	211467.5769	143342.8865	75504.69363	41	18.63461538	11.29994519	43.10390144	30.47241411	9.542421246	29.95843613
TCGA-3A-A9IB	1	NA	4520	5292.230769	4754.195122	4938.475565	283239	211378.3269	170510.0093	73905.74415	22	36.28846154	20.54535489	34.8936345	28.24652284	7.497462317	32.31030559
TCGA-3A-A9IC	1	NA	4521	5028.403846	4469.641957	4989.789733	284695	199800.3462	160464.358	75734.58111	27	18.63461538	16.43628391	37.9724846	29.21102009	8.668229052	31.17773114
TCGA-3A-A9IZ	1	NA	4496	5321.653846	4753.167854	4711.66694	284220	212713.1538	167847.3313	72501.7885	15	28.44230769	20.54535489	21.55195072	27.94022502	8.643797245	30.91038995
TCGA-FB-AAPP	1	NA	4331	4610.596154	4304.25185	5032.893634	270272	182656.5	157610.6082	74615.93224	27	9.807692308	3.081803234	35.91991786	28.76749203	10.01352154	33.68825392
TCGA-FB-AAPQ	1	NA	4494	4923.461538	4524.087147	5039.051335	283364	200369.1923	158719.0301	72417.63326	17	25.5	23.62715813	50.28788501	29.7612634	8.2832864	31.07368358
TCGA-FB-AAQ2	1	NA	4450	4929.346154	4129.616333	4903.58193	280941	193701.9231	147661.5201	73928.32238	26	8.826923077	12.32721294	32.84106776	32.22870359	7.318334603	31.00870862
TCGA-HZ-8005	1	NA	4351	4577.25	4845.621951	4844.057495	278954	183167.4808	168887.9535	73386.44476	20	24.51923077	16.43628391	41.0513347	29.20559076	9.90535508	29.03783451
TCGA-IB-7654	1	NA	4820	4792.038462	4397.733215	4970.290349	303532	192261.1731	161295.4176	73194.52977	35	21.57692308	16.43628391	38.99876797	29.97191027	7.68210619	32.56121102
TCGA-IB-7887	1	NA	4335	4472.307692	4504.56906	4881.003696	276201	181606.0962	160701.6569	73938.58522	37	16.67307692	9.245409701	29.76221766	30.71728685	9.885785719	29.4839193
TCGA-IB-A5SS	1	NA	4766	4633.153846	4305.279118	5180.678439	300847	188084.0769	157067.1836	77862.06653	19	18.63461538	14.38174842	29.76221766	29.07003923	8.240734958	30.57295327
TCGA-IB-A7LX	1	NA	4537	4743.980769	4139.889011	4856.372895	284461	198973.5577	150216.335	70283.99014	35	22.55769231	19.51808715	51.31416838	30.2763498	9.018408334	30.33256142
TCGA-US-A779	1	NA	5079	4976.423077	5309.946972	5250.465708	319269	193907.8846	191524.8256	75883.3922	29	13.73076923	14.38174842	46.18275154	29.49354613	5.949562187	33.36408769
TCGA-FB-A7DR	2	166	4364	4873.442308	4168.652508	5305.88501	277752	190962.6346	161017.0281	67891.72361	20	20.59615385	15.40901617	32.84106776	28.7974898	8.516566054	31.75549504
TCGA-2J-AAB4	2	729	4274	4651.788462	4496.350918	4993.894867	294089	163332.4038	160093.5144	77939.03778	18	15.69230769	18.4908194	27.70965092	30.33816461	9.098097622	29.44444348
TCGA-HZ-7919	2	20	4374	4240.846154	4251.861195	4628.537988	259397	180803.8269	159052.8922	77852.82998	16	14.71153846	16.43628391	23.60451745	30.71892127	9.378164081	30.01356524
TCGA-IB-8127	2	522	4603	4540.961538	4493.269115	4705.50924	286657	168951.2308	155954.6526	68714.80287	20	21.57692308	10.27267745	19.49938398	31.46651273	9.192549898	29.08051056
TCGA-YY-A8LH	2	2016	4822	4205.538462	3924.162784	4912.81848	275395	161518.9615	160425.3219	65532.29815	33	23.53846154	10.27267745	21.55195072	33.08638	9.104097798	29.46011086
TCGA-HV-A5A6	2	1953	5104	4254.576923	5018.202932	4477.674333	318426	172605.5769	176250.3815	66495.97823	41	21.57692308	44.17251302	44.1301848	27.71716494	8.174065897	31.93227586
TCGA-3A-A9IH	2	1021	4755	4637.076923	5303.783365	4747.586858	300216	189964.2115	188674.1576	72699.86119	20	19.61538462	22.59989038	22.57823409	29.92655836	7.013371587	31.62514977
TCGA-3E-AAAY	2	2285	4708	4838.134615	5012.039326	4382.229979	294593	189236.4808	174953.9696	64321.28378	13	16.67307692	27.7362291	27.70965092	30.68309427	8.999248384	29.83448326
TCGA-HV-A7OL	2	252	5187	4170.230769	5074.702658	4279.601643	325597	165666.6346	179000.3772	62459.60575	25	7.846153846	43.14524527	43.10390144	34.25067019	9.224527412	26.61237814
TCGA-2J-AAB8	2	80	4992	5054.884615	4547.714305	4445.859548	317525	201770.7115	160379.0948	64962.71088	15	30.40384615	21.57262264	21.55195072	30.67602773	8.046069747	31.15461797
TCGA-IB-A5SO	2	329	4780	4563.519231	4957.594135	4300.12731	300416	183609.8077	175282.6953	67006.04107	31	36.28846154	28.76349685	28.73593429	33.1455411	9.015828899	29.4846951
TCGA-2J-AABK	2	484	4376	4739.076923	5180.511236	4095.89692	273457	184984.8462	180292.68	63786.59014	11	31.38461538	34.92710332	34.8936345	30.97576232	9.047447053	30.0065865
TCGA-HV-A5A4	2	232	5010	4857.75	4839.458345	4301.153593	311594	193296.8654	172665.217	64564.51294	22	34.32692308	13.35448068	13.34168378	30.25758957	8.874979693	30.66398964
TCGA-FB-A545	2	385	5003	4829.307692	4808.640312	4078.450103	317610	201354.8654	165841.0774	64191.97207	24	15.69230769	18.4908194	18.47310062	28.63191729	7.70167418	35.40022553
TCGA-LB-A7SX	2	127	4557	4988.192308	5063.402713	4327.836961	284816	196527.5192	179824.246	66514.45133	21	11.76923077	29.79076459	29.76221766	33.79259701	8.905109206	28.85847685
TCGA-FB-A5VM	2	449	5075	4438.961538	4565.177857	4005.583984	319429	179056.0962	161616.9525	60980.73142	22	36.28846154	29.79076459	29.76221766	32.56532675	9.623066919	28.04639384
TCGA-HZ-A4BH	2	194	4951	4493.884615	4815.831187	4387.361396	307465	179490.5769	172725.8258	64998.6308	35	11.76923077	26.70896136	26.68336756	32.14180208	9.144649339	29.92662451
TCGA-S4-A8RO	2	525	5444	5132.365385	5090.111674	4988.76345	338122	198511.6154	182049.3079	74973.07885	30	18.63461538	44.17251302	44.1301848	29.12024669	6.709794897	31.1411216
TCGA-HZ-8637	2	0	4950	4871.480769	5139.420526	4169.789322	311350	196647.1731	178166.2358	65274.70103	31	27.46153846	27.7362291	27.70965092	33.02190954	9.752902736	26.99533499
TCGA-IB-A7M4	2	483	4614	4282.038462	4283.706495	4071.266119	287400	169781.9423	154338.7605	61721.70801	19	5.884615385	34.92710332	34.8936345	33.55581571	9.551187319	27.97780421
TCGA-2J-AAB6	2	NA	4982	4952.884615	4734.677035	4863.556879	306905	200203.4423	169158.125	70906.94415	35	30.40384615	9.245409701	42.07761807	28.99055612	6.613353898	31.0282532
TCGA-FB-AAQ0	2	NA	4434	4453.673077	4326.85174	4757.849692	280007	177251.4808	152411.6062	68938.53265	12	16.67307692	17.46355166	22.57823409	28.89176181	8.680244494	33.92013139
TCGA-2J-AABF	2	NA	4650	5578.615385	4400.815018	4929.239014	291472	223736.0192	153897.0354	72071.77577	24	23.53846154	9.245409701	23.60451745	30.63040706	8.42281576	28.93386493
TCGA-2L-AAQE	2	NA	4898	4726.326923	4290.897369	4461.253799	306275	195947.8846	154073.7254	65703.68747	25	15.69230769	14.38174842	23.60451745	34.13786504	8.968947188	30.11649186
TCGA-3A-A9IU	2	NA	4370	5292.230769	4431.63305	5001.07885	275484	206151.8077	154302.8061	73082.66489	24	23.53846154	9.245409701	31.81478439	30.48011156	7.641090821	30.74336881
TCGA-F2-6879	2	NA	5210	4793.019231	5146.6114	5038.025051	329150	193378.2692	186204.6059	74159.23614	26	34.32692308	24.65442587	34.8936345	27.83331958	7.839593627	32.33073095
TCGA-F2-A7TX	2	NA	4000	5130.403846	4371.024253	4199.55154	254851	199481.5962	153352.5834	59808.71581	17	31.38461538	14.38174842	41.0513347	32.25987632	10.45630706	27.90664091
TCGA-FB-AAQ3	2	NA	4626	4739.076923	4110.098246	4406.86078	297176	187446.5769	149534.2292	65640.05791	35	13.73076923	19.51808715	31.81478439	29.38498983	9.411252054	30.13648755
TCGA-HV-AA8X	2	NA	4678	5341.269231	4689.477254	5312.04271	294600	213327.1154	166008.5221	79589.30144	26	17.65384615	17.46355166	34.8936345	29.59997934	6.275588684	32.72829356
TCGA-IB-7646	2	NA	4487	4623.346154	4829.185667	4662.405339	289310	180558.6346	169737.504	70244.99138	24	36.28846154	12.32721294	36.94620123	28.27795655	8.337642181	30.23182381
TCGA-IB-7651	2	NA	4400	4571.365385	4322.742669	5279.201643	278041	175821.5192	159454.5539	80141.44189	25	24.51923077	19.51808715	37.9724846	32.00332114	9.327657831	29.26182687
TCGA-IB-7886	2	NA	4637	5198.076923	4202.552343	4813.268994	294015	204733.6154	149420.2025	73460.33717	24	17.65384615	16.43628391	21.55195072	29.89512386	9.311235169	29.70176136
TCGA-IB-AAUO	2	NA	4136	4979.365385	4539.496163	4638.800821	261269	200722.2692	162725.3743	70595.98029	29	23.53846154	13.35448068	26.68336756	30.60634964	10.02645083	27.88139877
TCGA-IB-AAUQ	2	NA	4790	4492.903846	4281.651959	4868.688296	301569	174871.1538	157389.7457	73572.20205	23	17.65384615	12.32721294	55.41930185	30.08640991	7.858922944	32.10161729
TCGA-US-A77E	2	NA	4909	5245.153846	4825.076596	5254.570842	303252	211528.3846	170840.7895	77617.81109	27	24.51923077	3.081803234	30.78850103	29.00614553	5.403653811	33.59989773
TCGA-US-A77G	2	NA	4620	5145.115385	4638.113867	4976.448049	285170	206164.5577	162797.2831	74683.66694	15	19.61538462	23.62715813	26.68336756	29.59404672	7.409395237	32.19616359
TCGA-3A-A9IL	3	2741	4044	4442.884615	3920.053713	4498.2	282986	190798.8462	170978.4434	70924.39097	27	15.69230769	5.136338723	40.02505133	28.16411388	7.765630546	34.8896889
TCGA-3A-A9IX	3	1037	4117	4300.673077	4237.479446	4864.583162	258243	182595.6923	140364.8374	68785.61643	21	13.73076923	14.38174842	10.26283368	33.28475516	9.1961448	29.21787622
TCGA-RB-AA9M	3	286	4132	4393.846154	4424.442176	5216.598357	288783	184430.7115	155645.4451	71147.09446	26	30.40384615	14.38174842	24.63080082	30.62834743	8.915756264	31.05281103
TCGA-IB-AAUV	3	404	4318	4499.769231	3836.845026	4719.877207	270420	150222.4615	156085.1156	73842.11458	15	7.846153846	9.245409701	28.73593429	32.47262707	9.606066785	29.36830316
TCGA-IB-7652	3	1116	4357	4029	4320.688134	4382.229979	300384	192007.1538	152957.0854	71728.99713	28	36.28846154	4.109070978	36.94620123	30.08433808	6.698709814	30.68452739
TCGA-XD-AAUI	3	202	4385	4455.634615	4001.207865	4784.53306	282901	161488.5577	159211.0914	71379.0345	26	23.53846154	17.46355166	30.78850103	33.37034354	7.787073626	29.92853174
TCGA-2L-AAQM	3	1383	4452	4415.423077	4812.749383	5461.880082	275776	163720.7885	154920.194	64453.67433	34	22.55769231	18.4908194	32.84106776	30.26485495	12.27486249	31.41585819
TCGA-XN-A8T5	3	720	4436	4531.153846	4538.468896	4711.66694	303139	171761.1346	136884.4542	74230.04969	26	21.57692308	14.38174842	34.8936345	34.39527641	10.12691163	29.52841843
TCGA-F2-7273	3	360	4388	4109.423077	4014.562346	4948.738398	269744	198859.7885	153636.1093	78960.18973	28	24.51923077	18.4908194	45.15646817	30.59761734	8.389501403	30.3720342
TCGA-H6-8124	3	392	4533	3885.807692	4581.614141	4823.531828	295290	176427.6346	142821.0345	73680.98809	9	11.76923077	14.38174842	33.86735113	30.37718238	9.348520313	29.59564715
TCGA-HZ-7926	3	8	4469	4447.788462	4095.716498	4433.544148	279933	168883.5577	153277.5929	71502.1885	13	29.42307692	8.218141957	32.84106776	33.16468771	8.636553079	29.02294753
TCGA-XD-AAUH	3	395	4529	4173.173077	4789.122225	4955.922382	281569	156575.8846	168621.8912	73256.10678	12	28.44230769	7.190874212	31.81478439	34.01938151	9.464060457	29.73863084
TCGA-HZ-8636	3	5	4541	4415.423077	4272.40655	5009.289117	280819	173688.3462	176204.1544	68376.12936	33	12.75	19.51808715	22.57823409	29.75074485	7.407910171	30.29520209
TCGA-HZ-A49G	3	660	4569	3993.692308	4777.82228	5075.997536	279558	193152.6923	162709.9653	72923.59097	10	13.73076923	15.40901617	27.70965092	30.62236431	8.341465364	29.85104531
TCGA-3A-A9IV	3	1103	4696	4024.096154	4310.415456	4927.186448	313352	197859.4038	173755.1481	74236.20739	9	16.67307692	14.38174842	18.47310062	26.34983897	6.660530708	36.33255935
TCGA-RL-AAAS	3	9	4561	4046.653846	4538.468896	5220.703491	266216	168512.8269	142942.2521	67101.48542	23	9.807692308	9.245409701	21.55195072	33.40501138	10.23504374	28.78417345
TCGA-2J-AABR	3	438	4665	4248.692308	4035.107701	4945.659548	259386	175916.6538	146654.7978	71435.48008	23	9.807692308	18.4908194	47.20903491	32.06992904	9.987054127	28.81894766
TCGA-HZ-7924	3	840	4720	5043.115385	4149.13442	4573.118686	267060	170040.8654	146770.879	70679.10924	18	13.73076923	7.190874212	43.10390144	32.87707903	9.020041578	29.3847927
TCGA-HZ-8002	3	24	4699	4437.980769	4459.369279	4920.002464	269487	173883.5192	162211.7405	70685.26694	19	20.59615385	14.38174842	35.91991786	30.93928257	9.278804728	30.1259306
TCGA-3A-A9IN	3	2084	4823	4496.826923	4308.360921	5328.463244	304832	183459.75	153838.4811	78662.56756	23	12.75	10.27267745	43.10390144	28.87321765	8.376052497	34.6241698
TCGA-IB-AAUW	3	179	4922	4860.692308	4877.467251	4938.475565	295374	207734.7692	146230.5362	68337.1306	17	19.61538462	13.35448068	10.26283368	32.13557449	8.963704023	29.77368541
TCGA-IB-A5ST	3	635	4578	4775.365385	4978.13949	4619.301437	288670	183817.7308	182634.8505	69704.14004	26	10.78846154	33.89983557	33.86735113	34.05708423	8.720221991	28.45579148
TCGA-3A-A9IR	3	1542	5123	4920.519231	4962.730474	4104.107187	322570	197142.4615	182455.0787	60962.25832	19	32.36538462	25.68169361	25.65708419	29.88440129	8.203067405	32.81746282
TCGA-HZ-8003	3	21	4737	4777.326923	5203.111126	4331.942094	297509	195474.1731	183107.3937	66474.42628	20	24.51923077	34.92710332	34.8936345	32.02339464	9.284552224	28.48955749
TCGA-IB-AAUT	3	287	4570	4536.057692	5086.002603	4101.028337	286380	180699.8654	181919.8722	61898.22875	25	18.63461538	21.57262264	21.55195072	31.82815942	10.25008279	29.40432086
TCGA-HZ-7923	3	314	4126	4976.423077	4491.214579	4326.810678	256325	198263.4808	156150.8608	65994.12567	3	13.73076923	17.46355166	17.44681725	32.1427509	9.278955237	29.11313296
TCGA-3A-A9IJ	3	1854	5697	4965.634615	5247.283639	4801.979877	356745	195374.1346	182197.2344	73439.8115	13	26.48076923	49.30885174	49.26160164	27.2968537	5.970034067	33.88537661
TCGA-S4-A8RM	3	737	5017	4917.576923	4681.259112	4066.134702	315178	197230.7308	167949.0308	60963.2846	19	25.5	18.4908194	18.47310062	31.09528519	8.860459746	31.4312022
TCGA-3A-A9IS	3	998	5108	4835.192308	5410.619211	4220.077207	317952	194354.1346	189083.0101	63361.70883	16	20.59615385	43.14524527	43.10390144	28.5023821	7.982911374	32.84678723
TCGA-IB-AAUP	3	431	4433	4867.557692	4468.614689	4340.152361	275051	192162.1154	159224.4459	66856.2037	25	17.65384615	34.92710332	34.8936345	35.47559354	10.07179704	27.852181
TCGA-HZ-A49H	3	491	4950	4255.557692	4369.996985	3951.190965	311471	169947.6923	158831.0023	59443.35893	30	13.73076923	20.54535489	20.52566735	32.20685578	9.866256845	28.66785425
TCGA-F2-6880	3	295	4730	4468.384615	4954.512332	4807.111294	297744	182378.9423	174633.462	72150.79959	29	19.61538462	39.03617429	38.99876797	28.25887715	8.971849114	34.06280894
TCGA-HZ-7920	3	0	4979	4886.192308	5002.793916	4056.898152	311758	191589.3462	177868.3282	61959.80575	36	22.55769231	29.79076459	29.76221766	32.45582752	9.47381399	29.30196024
TCGA-2J-AABT	3	319	4946	4670.423077	4685.368183	4725.008624	308531	187616.25	162266.1857	72382.73963	2	11.76923077	30.81803234	30.78850103	29.93415476	8.224769092	30.72705826
TCGA-HZ-7918	3	969	4307	3759.288462	4917.530693	3947.085832	273238	159200.4231	174476.2901	58447.86407	9	16.67307692	10.27267745	10.26283368	32.38009299	10.41486469	28.45289607
TCGA-HZ-8519	3	454	4834	4998.980769	4511.759934	4515.646817	300011	199347.2308	159269.6457	66135.75277	38	23.53846154	16.43628391	16.42053388	30.32420054	9.696797329	30.94218352
TCGA-HZ-A8P0	3	0	4604	4830.288462	5028.47561	4397.62423	287016	198390.9808	177784.0922	65240.83368	6	17.65384615	26.70896136	26.68336756	30.659223	7.767042623	31.66941585
TCGA-IB-AAUM	3	8	4577	4727.307692	4200.497808	4208.78809	290419	190867.5	155401.9826	65092.02259	17	21.57692308	45.19978076	45.15646817	32.35711612	9.762178223	30.1413985
TCGA-HZ-A77P	3	330	5141	5241.230769	4914.44889	4358.625462	321573	204942.5192	174870.7609	64876.50308	32	28.44230769	26.70896136	26.68336756	29.21503314	8.871140394	31.79115362
TCGA-IB-7885	3	1257	4373	4878.346154	4855.894629	4296.022177	278913	194199.1731	175846.6653	63456.1269	17	21.57692308	25.68169361	25.65708419	31.57270827	8.619584453	29.39466191
TCGA-IB-8126	3	462	4584	4915.615385	4286.788298	4374.019713	286583	196870.7885	151657.5917	67177.43039	4	30.40384615	39.03617429	38.99876797	32.37778426	10.83877026	29.29869985
TCGA-HZ-A8P1	3	7	4900	5112.75	4968.894081	4653.168789	305410	204412.9038	173615.4397	67744.96509	26	32.36538462	35.95437106	35.91991786	29.11418285	7.409242344	33.06540304
TCGA-3A-A9I9	3	NA	4635	4679.25	4058.734859	4592.61807	293905	190585.0385	146580.8345	67558.18152	20	27.46153846	18.4908194	17.44681725	29.93787144	8.9806442	29.63393584
TCGA-3E-AAAZ	3	NA	4541	5144.134615	4888.767196	5069.839836	287989	206632.3846	170457.6187	76537.1347	20	14.71153846	12.32721294	33.86735113	30.15637318	7.468693187	32.83143783
TCGA-HV-A5A3	3	NA	4231	4714.557692	4363.833379	4957.974949	269895	183748.0962	155992.6616	72963.61602	24	26.48076923	20.54535489	29.76221766	30.99867624	9.274084943	29.04246233
TCGA-IB-7888	3	NA	4230	4803.807692	4205.634146	4691.141273	267187	194616	152051.0352	71689.99836	13	22.55769231	5.136338723	31.81478439	33.11902467	8.978042555	29.04691679
TCGA-2J-AABV	3	NA	3959	4928.365385	4705.913538	4453.043532	247432	195587.9423	162851.7283	65498.4308	15	25.5	16.43628391	31.81478439	31.10433	10.21320246	29.54142842
TCGA-F2-7276	3	NA	4200	4686.115385	4423.414908	4626.485421	268028	186137.25	161021.1372	68754.82793	30	13.73076923	7.190874212	47.20903491	31.9835869	9.006071789	29.40444123
TCGA-IB-7645	3	NA	4305	4778.307692	4427.523979	4680.878439	276144	192091.5	157469.8726	69976.10513	24	19.61538462	6.163606468	19.49938398	31.68878473	8.751951982	29.65859935
TCGA-IB-7649	3	NA	4747	4616.480769	3906.699233	5118.075154	302030	181421.7115	136720.0914	78231.52854	12	20.59615385	11.29994519	30.78850103	31.65946977	9.247344343	29.69857405
TCGA-IB-7889	3	NA	4042	4739.076923	4188.170595	4523.857084	256034	192015.9808	149873.2276	65542.56099	29	15.69230769	9.245409701	29.76221766	32.92449104	9.503634792	28.70030962
TCGA-IB-7897	3	NA	4014	4925.423077	4110.098246	4242.655441	251483	197125.7885	152707.4593	65253.14908	7	34.32692308	11.29994519	12.31540041	31.061377	9.753432469	29.81038003
TCGA-IB-A6UG	3	NA	4141	4316.365385	4051.543985	4924.107598	261497	174495.5192	149013.4045	74553.32895	14	11.76923077	13.35448068	38.99876797	33.44969528	8.671381328	29.581299
TCGA-US-A77J	3	NA	3945	4780.269231	4455.260208	4254.970842	253021	192129.75	157780.1074	62546.83984	23	21.57692308	19.51808715	32.84106776	34.7355653	9.447369278	28.1865324
TCGA-2L-AAQJ	3	NA	4630	4887.173077	4563.123321	4970.290349	292930	199657.1538	159899.3608	75135.23162	13	28.44230769	10.27267745	12.31540041	32.58521794	8.111485501	30.52293891
TCGA-HV-A7OP	4	978	4509	4625.307692	4846.649219	4065.108419	285169	185424.2308	171538.3043	59632.19507	11	18.63461538	24.65442587	24.63080082	33.19777048	10.4307878	26.60550314
TCGA-PZ-A5RE	6	247	4143	4164.346154	3999.15333	4463.306366	298024	177824.25	166335.1932	72406.34415	16	13.73076923	17.46355166	33.86735113	30.42571895	7.619202579	29.04938544
TCGA-3A-A9I7	6	1323	4247	4190.826923	4088.525623	4720.903491	276866	169425.9231	155083.5296	68632.70021	12	7.846153846	19.51808715	27.70965092	34.13752564	9.501411589	28.82685724
TCGA-IB-7891	6	648	4293	4143.75	4396.705947	4564.908419	273383	174163.0385	136623.5282	72697.80862	8	29.42307692	8.218141957	25.65708419	31.7496018	8.991122115	29.33897306
TCGA-H8-A6C1	6	671	4438	4304.596154	4435.742121	4920.002464	285509	171143.25	154059.3437	72808.64723	25	11.76923077	16.43628391	32.84106776	31.77104955	7.151721322	30.12803451
TCGA-HZ-8315	6	28	4496	4103.538462	4344.315292	4761.954825	295988	165841.2115	171134.5881	72856.88255	14	7.846153846	14.38174842	36.94620123	30.78201428	9.192603719	29.17719305
TCGA-FB-AAPZ	6	716	4468	4720.442308	4408.005892	4844.057495	276466	181445.25	142974.0974	71760.81191	12	16.67307692	11.29994519	25.65708419	30.92262745	9.296281781	29.28652676
TCGA-FB-A4P6	6	767	4417	4085.884615	4331.988079	4690.11499	254171	174312.1154	150867.6228	74763.71704	8	16.67307692	12.32721294	38.99876797	31.13519976	8.819898647	30.90271014
TCGA-HV-A5A5	6	289	4655	4517.423077	4222.07043	5050.340452	284927	168929.6538	175961.7192	74295.73183	20	21.57692308	14.38174842	43.10390144	30.97885546	8.156333173	30.6824316
TCGA-FB-AAPS	6	228	4821	4720.442308	4608.323102	5319.226694	279272	171843.5192	144565.3352	73991.95195	22	24.51923077	3.081803234	22.57823409	32.46151759	9.067945619	30.39848263
TCGA-LB-A9Q5	6	155	4894	4725.346154	4824.049329	5143.732238	272146	189027.5769	157989.67	73578.35975	26	19.61538462	15.40901617	31.81478439	29.87257913	8.076276095	29.29856659
TCGA-F2-A44H	6	586	4562	4902.865385	5222.629213	4628.537988	287850	196061.6538	179530.4474	71859.33511	24	17.65384615	15.40901617	15.39425051	28.84813551	7.245938425	31.22620323
TCGA-IB-AAUR	6	338	4344	4711.615385	4632.977528	3953.243532	271629	188971.6731	168473.9646	61532.87187	28	15.69230769	17.46355166	17.44681725	35.20239301	10.1479839	27.85548446
TCGA-FB-A4P5	6	4	4891	4432.096154	4203.579611	4143.105955	305900	177782.0769	153161.5116	63388.3922	31	19.61538462	22.59989038	22.57823409	34.06945786	10.32913733	28.38259603
TCGA-HZ-8001	6	706	5172	4701.807692	4734.677035	3862.930595	326321	190708.6154	168439.0375	60336.22546	33	19.61538462	24.65442587	24.63080082	32.7079779	9.355052944	28.56394317
TCGA-HZ-A4BK	6	657	5027	5101.961538	5004.848452	4220.077207	314617	208240.8462	173851.7113	64025.71417	31	20.59615385	14.38174842	14.36796715	31.80624315	8.884678836	29.62312185
TCGA-IB-A5SP	6	482	4900	4459.557692	4673.04097	4595.69692	306390	174926.0769	160948.2012	70361.98768	23	19.61538462	29.79076459	29.76221766	29.50883791	8.594774386	29.89083346
TCGA-RB-A7B8	6	36	4783	4817.538462	4682.28638	4416.097331	304908	185050.5577	168377.4015	68106.21684	12	27.46153846	18.4908194	18.47310062	30.72958251	8.776536339	30.66906799
TCGA-IB-AAUS	6	225	4942	4608.634615	4781.931351	5027.762218	309880	180995.0769	171750.9488	77441.29035	38	17.65384615	32.87256783	32.84106776	30.82800689	8.858422774	29.30361857
TCGA-H6-A45N	6	233	4637	4724.365385	4749.058783	4234.445175	294002	195009.2885	171642.0584	65274.70103	23	18.63461538	21.57262264	21.55195072	31.82852818	9.541146258	29.74300606
TCGA-2J-AAB1	6	NA	4415	4875.403846	3909.781036	4892.292813	282500	192581.8846	140847.6532	75372.30308	41	9.807692308	16.43628391	36.94620123	31.24133146	9.302112493	30.01333494
TCGA-2J-AAB9	6	NA	4527	4466.423077	4411.087695	4921.028747	289181	174595.5577	160404.7765	71003.41478	30	17.65384615	7.190874212	44.1301848	30.98586334	8.822151795	30.6480145
TCGA-FB-AAPY	6	NA	4318	4616.480769	3522.501096	4805.058727	269908	185798.8846	121765.1276	70423.56468	11	10.78846154	2.054535489	32.84106776	33.26975256	9.012225256	28.49666112
TCGA-FB-AAQ1	6	NA	4926	4717.5	4704.88627	4744.508008	311563	192527.9423	165331.5526	71092.70144	31	25.5	5.136338723	29.76221766	30.00853282	7.791140438	30.24324631
TCGA-S4-A8RP	6	NA	4593	4835.192308	3900.535626	4514.620534	291175	199814.0769	140686.3722	65124.86366	24	17.65384615	9.245409701	13.34168378	29.65573476	8.819958892	30.58658291
TCGA-US-A774	6	NA	4194	4403.653846	4094.68923	5074.971253	263515	175134	146535.6347	77367.39795	12	7.846153846	13.35448068	57.47186858	32.17189191	9.046874158	28.92797847
